# Abstracts from the 17th European Headache Congress (EHC)

**DOI:** 10.1186/s10194-024-01793-4

**Published:** 2024-08-01

## AL001 Metabolic dysfunction in new-onset IIH: Identification of diagnostic and prognostic biomarkers

### J. J. Korsbæk^1^, R. H. Jensen^1^, D. Beier^2^, E. A. Wibroe^3^, S. M. Hagen^3^, L. D. Molander^4^, M. P. Gillum^5^, K. Svart^1^, L. J. Kogelman^1^, T. F. Hansen^1^*,* C. S. J. Westgate^1^

#### ^1^Rigshospitalet Glostrup, University of Copenhagen, Danish Headache Center, Glostrup, Denmark; ^2^Odense University Hospital, Department of Neurology, Odense, Denmark; ^3^Rigshospitalet Glostrup, University of Copenhagen, Department of Ophthalmology, Glostrup, Denmark; ^4^Odense University Hospital, Department of Ophthalmology, Odense, Denmark; ^5^Novo Nordisk A/S, Global Obesity and Liver Disease Research, Måløv, Denmark

##### **Correspondence:** C. S. J. Westgate

*The Journal of Headache and Pain 2024,* **25(Suppl 1)**: AL001

**Objective:** The pathological underpinnings of idiopathic intracranial hypertension (IIH), a disease that primarily affects young obese women of reproductive age, are unknown. Although metabolic dysregulation occurs later in the disease course of IIH, it is unclear if this is present at the point of diagnosis, or if this dysfunction could be pathogenic or a consequence of IIH. Moreover, given the lack of prognostic markers in IIH, it is vital to understand if potential metabolic dysfunction has prognostic value.

**Methods:** In a prospective cohort of newly diagnosed IIH patients with follow up at ocular remission (*N*=60) we performed non-targeted liquid chromatography tandem mass-spectrometry metabolomics on serum and cerebrospinal fluid (CSF) with age, sex and BMI matched healthy controls (*N*=35).

**Results:** The lipid sphingosine-1-phosphate (S1P) is lower in newly diagnosed IIH patients (IIH vs control, fold change 0.47, *P*<0.0001) where this normalizes following ocular remission (remission vs IIH, fold change 2.23, *P*=0.044). Lower S1P at diagnosis, is predictive of a lower chance of developing bilateral optic nerve atrophy (*P*=0.02). Serum adenosine (*P*<0.01), glutamate (*P*<0.0001) and CSF LysoPC (C18:0) (*P*<0.0001) and LysoPC (C16:0) (*P*<0.0001) were lower in new onset IIH but show no visual prognostic value. In newly diagnosed IIH patients, pathway analysis identifies dysregulated carbohydrate metabolism in the CSF. Additionally, we identify dysregulated eicosanoid, neuroprostane and vitamin E metabolism in IIH serum. Finaly, we identify dysregulated amino acid, lipid and steroid hormone metabolism in the serum of new onset IIH.

**Conclusion:** We demonstrate in a rigorously phenotyped cohort of new onset IIH, that metabolic dysfunction is a feature of early IIH. We identify S1P as a candidate pathogenic moiety that could have neuro-ophthalmological prognostic value as well as dysregulated carbohydrate, lipid and steroid metabolism. Future work is required to see if S1P has true prognostic value.

## AL002 Neurofilament light chain is elevated in newly diagnosed idiopathic intracranial hypertension

### K. Svart^1^, J. Juhl Korsbaek^1^, R. H. Jensen^1,2^, T. Parkner^3,4^, C. Soendersoe Knudsen^3,4^, S. Gregers Hasselbalch^2,5^, S. Malm Hagen^2,6^, E. Arnberg Wibroe^2,6^, L. Dehghani Molander^7^, D. Beier^8,9,10^

#### ^1^Danish Headache Center, Copenhagen University Hospital – Rigshospitalet Glostrup, Neurology, Glostrup, Denmark; ^2^University of Copenhagen, Copenhagen, Denmark; ^3^Aarhus University Hospital, Department of Clinical Biochemistry, Aarhus, Denmark; ^4^University of Aarhus, Clinical Medicine, Aarhus, Denmark; ^5^Danish Dementia Research Center, Rigshospitalet-Glostrup, Neurology, Copenhagen, Denmark; ^6^Rigshospitalet Glostrup, University of Copenhagen, Ophthalmology, Glostrup, Denmark; ^7^Odense University Hospital, Ophthalmology, Odense, Denmark; ^8^Odense University Hospital, Headache Clinic, Neurology Department, Odense, Denmark; ^9^University of Southern Denmark, Clinical Research, Odense, Denmark; ^10^Odense University Hospital, OPEN (Odense Patient Data Explorative Network), Odense, Denmark

##### **Correspondence:** J. Juhl Korsbaek

*The Journal of Headache and Pain 2024,* **25(Suppl 1)**: AL002

**Objective:** Neurofilament light chain (NfL) is elevated in Idiopathic Intracranial Hypertension (IIH). It is unclear whether this reflects optic nerve damage or unspecific axonal damage limiting its use as a prognostic marker. Further investigations of neurodegenerative biomarkers in IIH are needed. Here, we investigate NfL along with amyloid-beta 42 (Aβ-42), total-tau (t-tau), and phosphorylated-tau (p-tau) in new-onset IIH.

**Methods:** This prospective case-control study included newly diagnosed IIH patients and age, sex, and BMI matched healthy controls. CSF was analyzed for NfL, Aβ-42, t-tau, and p-tau, and plasma for NfL. Biomarker levels were compared between patients and controls and correlated with papilledema grade, visual fields and opening pressure (OP). We evaluated cNfL and pNfL levels at diagnosis in relation to visual field defects and optic nerve atrophy at ocular remission.

**Results:** We included 37 IIH patients and 35 controls. IIH patients showed higher levels of age-adjusted cNfL (1.4 vs. 0.6 pg/mL, q<0.001) and pNfL (0.5 vs. 0.3 pg/mL, q<0.001) compared to controls. The t-tau/Aβ-42 ratio was elevated in IIH (0.12 vs. 0.11, q=0.04). Significant positive linear correlations were observed between cNfL, pNfL, t-tau/Aβ-42 and OP. High cNfL was associated with severe papilledema and perimetric mean deviation was inversely correlated with cNfL. No associations were found between cNfL/pNfL and ophthalmological outcomes at remission.

**Conclusion:** Elevated cNfL, pNfL, and t-tau/Aβ-42 were found in new-onset IIH compared to controls. cNfL was associated with OP, papilledema severity and visual field defects at diagnosis. There were no signs of global axonal damage. Our findings indicate early, pressure-induced optic nerve damage in IIH, which is associated with cNfL. This confirms cNfL as a potential biomarker for assessing disease severity and optic nerve damage in IIH.

## AL003 Raised intracranial pressure alters cortical spreading depression responses, induces neurovascular uncoupling and reduces trigeminal sensitivity thresholds which is rescued by a GLP-1R agonist

### O. Grech^1^, E. Rubio-Beltran^1,2^, E. C. Stanyer^2,3^, A. Labastida-Ramirez^2^, L. Hill^1,4^, P. R. Holland^2^, A. Sinclair^1^

#### ^1^University of Birmingham, Translational Brain Science, Birmingham, United Kingdom; ^2^King's College London, Headache Group, London, United Kingdom; ^3^University of Oxford, Sleep and Circadian Neuroscience Institute, Oxford, United Kingdom; ^4^University of Birmingham, Institute of Clinical Sciences, Birmingham, United Kingdom

##### **Correspondence:** O. Grech

*The Journal of Headache and Pain 2024,* **25(Suppl 1)**: AL003

**Objective:** Raised intracranial pressure (ICP) is a feature of secondary headaches, however, pathological mechanisms underlying headache are unknown, and targeted therapies are lacking. This study investigated the impact of ICP on headache pathology and the effects of reducing ICP with glucagon-like peptide-1 receptor agonist (GLP1-RA).

**Methods:** Kaolin (or saline) was injected into the cisterna magna of male Sprague-Dawley rats to increase ICP. After 7 days following injection mechanical thresholds were assessed. Changes in direct current shift [DC] and cerebral blood flow (CBF) were measured following KCL-induced CSD. Behavioral and CSD measurements were repeated in kaolin animals treated with GLP-1RA exenatide (20ug/kg) or vehicle.

**Results:** ICP was significantly higher in kaolin animals (saline mean (SD) ICP=5.46mmHg(1.22) *n*=6, kaolin=17.72mmHg(10.124) *n*=8 *p*=0.001). Mechanical thresholds were decreased (periorbital; saline=6.43g (1.88) *n*=10, kaolin=2.35g(1.91) *n*=12 *p*<0.001, hind paw; saline=5.16g(1.40), kaolin=3.34g(2.21) *p*<0.001). CSD responses were drastically different in kaolin animals (depolarization duration saline=62.18s (42.13) *n*=9, kaolin=126.72s(76.12) *n*=11 *p*=0.038). %CBF change was significantly lower (saline=217.65% (37.70) *n*=8, kaolin=85.55%(30.84) *n*=9 *p*<0.001).

In kaolin animals GLP-1RA reduced ICP (vehicle=18.27mmHg (6.67) n=16, exenatide=9.74mmHg(6.09) *n*=19 *p*<0.001). GLP-1RA rescued mechanical sensitivity (periorbital; vehicle =4.67g (2.81), kaolin=5.65g(2.08) *p*=0.0010, hind paw; vehicle=2.53g(1.24), kaolin=6.78g(1.20) *n*=12 *p*<0.0001). Exenatide improved CSD responses (repolarization duration vehicle=986.25s (691.82) *n*=6, kaolin=257.55s(194.56) *n*=7 *p*=0.002).

**Conclusion:** Raised ICP altered trigeminal sensitivity thresholds, neurovascular uncoupling and altered CSD responses which were reversed by reducing ICP with GLP-1RA. ICP may be directly related to headache pathophysiology and reducing ICP with GLP-1RA may provide a targeted therapeutic for headache.

## AL004 Migraine impact on patient's life in terms quality of life and mental health (2020 national health and wellness survey)

### D. García Azorín^1^, C. Moya-Alarcón^2^, B. Armada^2^, M. Sánchez del Río^3^

#### ^1^Hospital Clínico Universitario de Valladolid, Headache Unit, Department of Neurology, Valladolid, Spain; ^2^Pfizer S.L.U., Alcobendas, Spain; ^3^Clínica Universidad de Navarra, Department of Neurology, Madrid, Spain

##### **Correspondence:** D. García Azorín; M. Sánchez del Río

*The Journal of Headache and Pain 2024,* **25(Suppl 1)**: AL004

**Objective:** This study evaluated patient-reported outcomes from people with migraine compared to controls regarding quality of life (QoL) and depression in Spain.

**Methods:** A cross-sectional study including 7,074 respondents to the 2020 National Health and Wellness Survey (NHWS) captured patient-reported outcomes about QoL using SF-12 health survey and EQ-5D questionnaire, and associated depression using the patient health questionnaire PHQ-9. Migraine patients included respondents who reported a physician-confirmed diagnosis of migraine (*n*=1,020) and ≥1 monthly headache days (MHD) in the preceding 30 days (*n*= 595). Control group included respondents that reported no having history of migraine (*n*=5,490), matched by propensity score based on 11 demographic and clinical characteristics (*n*=1,190).

**Results:** The mean age of the study population was 41 years and 67% were females. All analyses revealed significant differences (*p*<0.001) between the two cohorts in SF-12, EQ-5D and PHQ-9. Patients with migraine reported worse mean SF-12 mental component summary scores (41.88 vs 44.73) and worse SF-12 physical component scores (48.62 vs 51.53). EQ-5D showed greater burden in the migraine group compared to the control group in utility score (0.765 vs 0.844) and visual analog scale (VAS) (65.80 vs 73.54). Regarding depression, 70.4% of patients with migraine vs 50.5% of control group individuals had PHQ-9 mean index scores ≥5 and twice more patients with migraine (18.82% vs 9.92%) showed scores within the moderate-severe depression range. It was remarkable that 18% of patients with ≥15 MHD presented severe depression scores.

**Conclusion:** In 2020, during COVID pandemic, individuals suffering from migraine in Spain had significantly lower QoL (both mental and physical) and more depression compared to non-migraineurs. Globally, migraineurs reported more depression than controls, especially significant if they suffered from ≥15 MHD.

**Fig. 1 (Abstract AL004):**
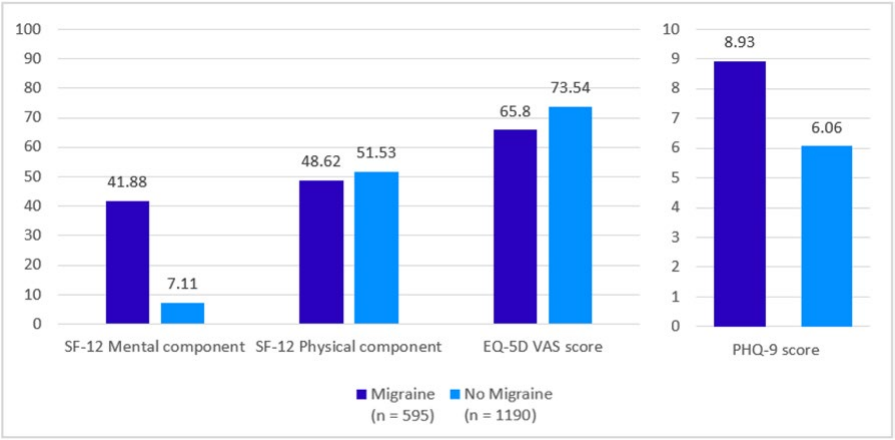
Mean score comparison of the patient reported outcomes in SF-12 health survey, EQ-5D questionnaire and patient health questionnaire PHQ-9 among people with migraine matched to no migraine controls

## AL005 Effectiveness of galcanezumab vs. traditional oral migraine preventive medications: interim 3-Month results from real-world TRIUMPH study

### R. B. Lipton^1,2^, M. Láinez^3^, Z. Ahmed^4^, T. Kurth^5^*,* M. Vincent^6^, D. Novick^6^, C. Vallarino^6^, L. Viktrup^6^, R. L. Robinson^6^

#### ^1^Albert Einstein College of Medicine, Department of Neurology, Bronx, MA, United States; ^2^Montefiore Medical Center, Headache Center, Bronx, MA, United States; ^3^Universidad Católica de Valencia, Hospital Clínico Universitario, Valencia, Spain; ^4^Cleveland Clinic, Cleveland, OH, United States; ^5^Charité – Universitätsmedizin Berlin, Institute of Public Health, Berlin, Germany; ^6^Eli Lilly and Company, Indianapolis, IN, United States

##### **Correspondence:** M. Vincent

*The Journal of Headache and Pain 2024,* **25(Suppl 1)**: AL005

**Objective:** To compare the 3-month effectiveness of galcanezumab and traditional oral migraine preventive medications (TOMP) in patients (pts) with migraine.

**Methods:** Pts with ≥4 monthly migraine headache days reported 30 days prior to taking galcanezumab or a TOMP were included (02/2020-02/2023). The effectiveness was assessed as reduction from baseline in physician-reported monthly migraine headache days at 3 months (response): ≥30% in chronic migraine (CM) and ≥50% in episodic migraine (EM). The difference in the proportion of responders between treatments was assessed using a weighted Chi-squared test. The weights were derived from a least absolute shrinkage and selection operator (LASSO) model fit of propensity scores using 65 baseline covariates. The response rates were also reported by migraine subtype (CM or EM). Sensitivity analysis was performed using Frequentist Model Averaging and the primary endpoint was compared between groups at 5% alpha level (two-sided).

**Results:** At baseline, most pts were diagnosed with CM, and had high disability, with mean monthly migraine headache days of 14.4, and 11.4 (Table 1). [FJ1] [RLR2] At 3 months, the galcanezumab and TOMP cohorts reported mean (SD) monthly migraine headache days of 8.2 (7.8) and 7.3 (5.8). The change from baseline in monthly migraine headache days with acute medication use was –4.98 for galcanezumab and –3.47 for TOMP. Overall, the weighted response rate for galcanezumab was significantly greater vs TOMP (45.8% vs. 34.1% *p*<0.0001). In both migraine subtypes, the weighted response rate for galcanezumab was greater vs TOMP (CM: 44.3% vs. 36.9, *p*=0.0127; EM: 49.7% vs. 29.1%, *p*<0.0001).

**Conclusion:** Pts with migraine initiating/switching to galcanezumab had statistically significantly better response rates vs TOMP at 3 months, despite greater disability at baseline. The results were consistent in both CM and EM. [FJ1] Changes in mean monthly headache days also resulted in greater reduction of acute medications for pts on galcanezumab.

Previously presented at American Headache Society - 65th Annual Scientific Meeting 2023.

**Table 1 (Abstract AL005):**
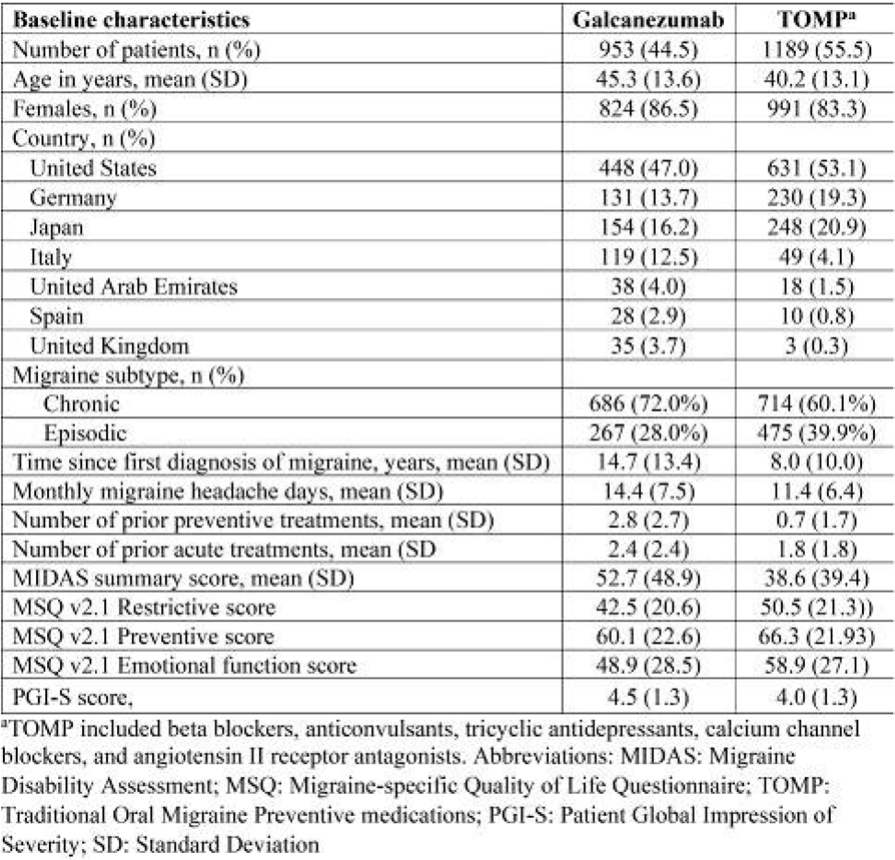
Baseline charateristics of patients before switching to or initiating on galcanezumab or TOMP

**Fig. 1 (Abstract AL005):**
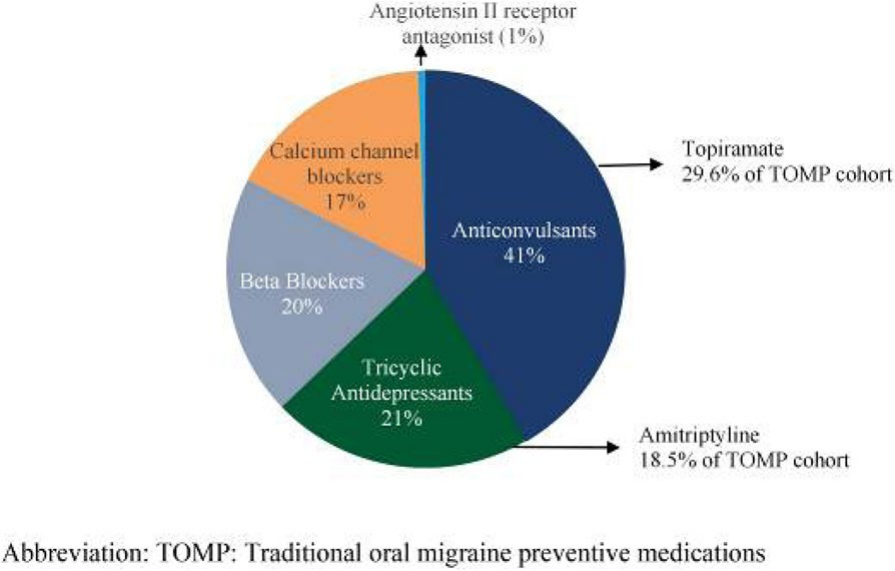
Types of traditional oral migraine preventive (n=1,189). Abbreviation: TOMP: Traditional oral migraine preventive medications

## AL006 Headwork as innovative tool for monitoring mAbs efficacy in migraine and their influence on work activity: a multicentric experience

### D. A. Montisano^1^, G. Vaghi^2^, A. Raggi^1^, C. Altamura^3^, F. Vernieri^3^, C. Tassorelli^2^, G. Sances^2^, L. Grazzi^1^

#### ^1^Foundation IRCCS C. Besta Institute, Milano, Italy; ^2^Foundation IRCCS Mondino Institute, Pavia, Italy; ^3^Campus Bio-Medico University, Rome, Italy

##### **Correspondence:** D. A. Montisano

*The Journal of Headache and Pain 2024,* **25(Suppl 1)**: AL006

**Objective:** Aim of this study is to test the validity of HeadWork (HW), new work related evaluation tools, and to compare its performance with usually used clinical indexes.

**Methods:** We enrolled 108 patients receiving treatment with mAbs at the Headache Centres of C. Besta, C. Mondino and Campus-Biomedico.Patients enrolled were followed up on a three-month basis, at each time point they filled in diaries about headache frequency (MMD),medication intake (MMI) and HW.HW questionnaire consists of two sections: “Work-related difficulties"(HW1),11 items dealing with the degree of difficulty in general skills, problems solving or starting new task; “Factors contributing to work-related difficulties"(HW2),6 items to address the degree to which some factors, such as noise and brightness of the workplace, negatively impact work-related tasks. Friedman and Wilcoxon repeated measure tests were used for the analysis(*p*<.005). We also assessed the presence of a correlation between T0-T3 and T0-T6 deltas with SpearmanRho.

**Results:** 108 patients (79%females, average age 50y±9, disease duration 16±8, age at onset of disease 18y±8) completed the 6-months evaluation with valid data. For each of the parameters a significant reduction was observed in the first three months of treatment (*p*<.001), maintained at six months. MMD decreased from 16.8±6.5 days to 8.1±6.2; MMI from 17.8±9.7 to 7.8±6.5; HW1scale from 23.8±10.5 to 15.2±10.0; HW2scale from 11.3±6.3 to 8.1±5.4. 60 patients out of 108(56%) reduced monthly headaches by 50% or more, and 29(27%) by 75% or more. The correlation for HW1 is moderate and significant with deltas 0-3 and 0-6 for MMD and MMI; for HW2 it is significant for 0-3 changes for MMD, and only with MMI for 0-6 change.

**Conclusion:** HW has a parallel reduction with indexes usually used to monitor treatment efficacy in clinical practice, suggesting good reliability and fidelity. We observed that the reductions of MMD, MMI and HW scales were correlated: although a causal direction cannot be stated, so that improvement in work-related activities might be due to improved clinical course. With HW we can highlight and evaluate the effectiveness of these treatments in relation to work productivity.

## AL007 Increased iron deposition in nucleus accumbens associated with disease progression and chronicity in migraine

### K. Liu, X. Xu, M. Zhou, X. Wu, F. Zhao, X. Luo, K. Li, Q. Z. Zeng, J. He, H. Cheng, X. Guan, P. Huang, M. Zhang

#### The Second Affiliated Hospital, Zhejiang University School of Medicine, Department of Neurology, Hangzhou, China

##### **Correspondence:** K. Liu

*The Journal of Headache and Pain 2024,* **25(Suppl 1)**: AL007

**Objective:** Migraine is one of the world’s most prevalent and disabling diseases. Despite huge advances in neuroimaging research, more valuable neuroimaging markers are still urgently needed to provide important insights into the brain mechanisms that underlie migraine symptoms. We therefore aim to investigate the regional iron deposition in subcortical nuclei of migraineurs as compared to controls, and its association with migraine related pathophysiological assessments.

**Methods:** A total of 200 migraineurs (56 chronic migraine [CM], 144 episodic migraine [EM]) and 41 matched controls were recruited. All subjects underwent MRI and clinical variables including frequency/duration of migraine, intensity of migraine, 6-item Headache Impact Test (HIT-6), Migraine Disability Assessment (MIDAS), and Pittsburgh Sleep Quality Index (PSQI) were recorded. Quantitative susceptibility mapping was employed to quantify the regional iron content in subcortical regions. Associations between clinical variables and regional iron deposition were studied as well.

**Results:** Increased iron deposition in putamen, caudate, and nucleus accumbens (NAC) was observed in migraineurs than controls. Meanwhile, patients with CM had significantly higher volume of iron deposits compared to EM in multiple subcortical nuclei, especially in NAC. Volume of iron in NAC can be used to distinguish patients with CM from EM with a sensitivity of 85.45% and specificity of 71.53%. As the most valuable neuroimaging markers in all of the subcortical nuclei, higher iron deposition in NAC was significantly associated with disease progression, and higher HIT-6, MIDAS, and PSQI.

**Conclusion:** These findings provide evidence that iron deposition in NAC may be a biomarker for migraine chronicity and migraine-related dysfunctions, thus may help to understand the underlying vascular and neural mechanisms of migraine.

**Keywords**: migraine, iron deposition, nucleus accumbens, disease burden, chronicity, neuroimaging biomarkers

**Fig. 1 (Abstract AL007):**
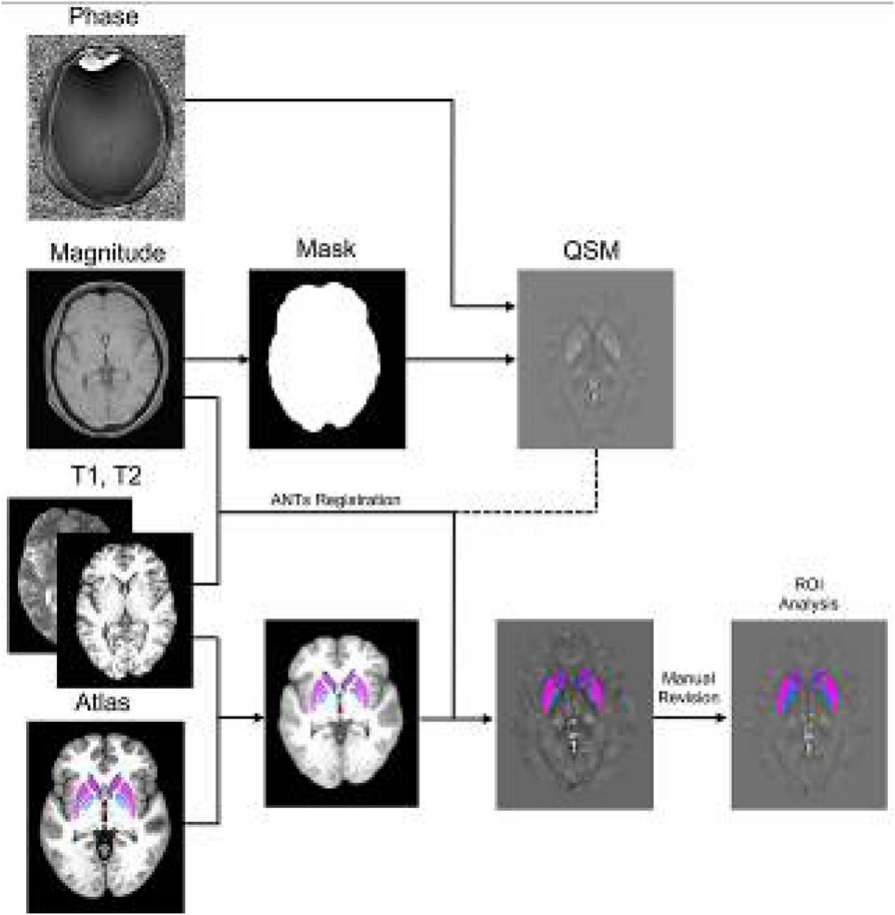
See text for description

**Fig. 2 (Abstract AL007):**
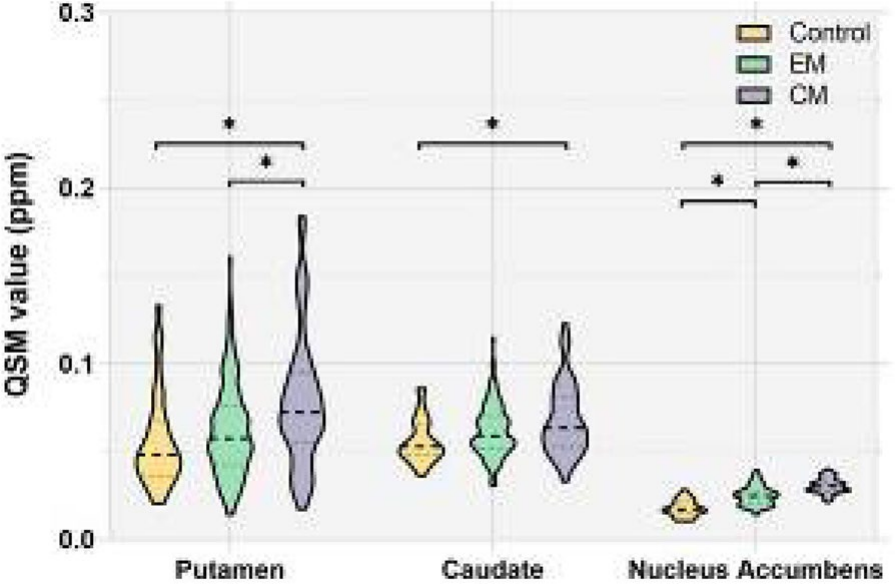
See text for description

**Fig. 3 (Abstract AL007):**
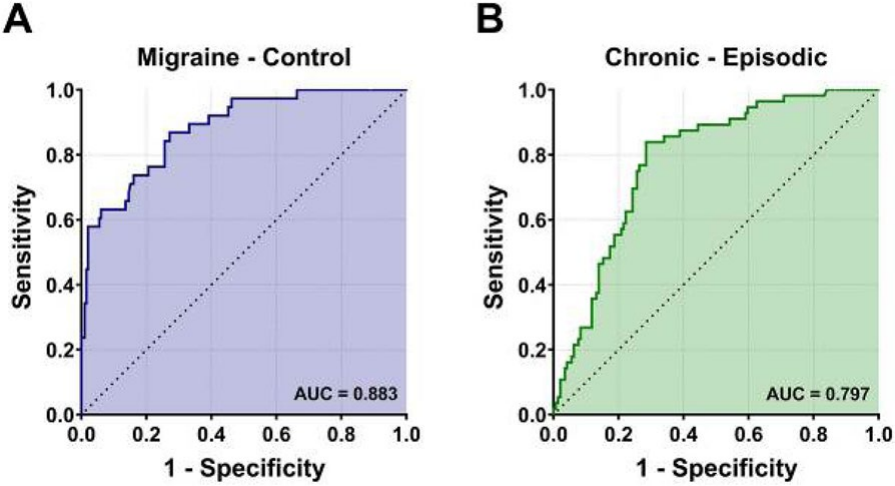
See text for description

## AL008 Extended phenotyping of migraine in children: a prospective study in a specialist children's headache clinic

### N. Karsan^1,2^, P. Prabhakar^2^, P. J. Goadsby^1,2,3^

#### ^1^NIHR-King's Clinical Research Facility, King's College Hospital, London, United Kingdom; ^2^Great Ormond Street Hospital for Children, Department of Neurology, London, United Kingdom; ^3^University of California, Department of Neurology, Los Angeles, CA, United States

##### **Correspondence:** N. Karsan

*The Journal of Headache and Pain 2024,* **25(Suppl 1)**:AL008

**Objective:** We aimed to perform prospective extended migraine phenotyping in children, to characterise migraine-related symptoms in this patient group.

**Methods:** Consecutive patients presenting to the clinic between 8/1/22 and 8/2/23 (*n*=105) were included in a service evaluation. Data was collected prospectively from the first clinical encounter, including age, gender, diagnosis, disease duration, gestational age, migraine markers, triggers, headache laterality and features, premonitory symptoms (PS), vertigo, allodynia, cranial autonomic symptoms (CAS), attack duration and preventive use. Data were analysed using descriptive statistics, Cohen's kappa (*k*) and a linear regression model. Bonferroni correction was applied so significance was assessed at *P*<0.01.

**Results:** Patients were 65% female and aged 5-17 years (median 14, IQR 11-15). Mean disease duration was 4.7 years (SD 2.8), headache frequency 1-30 days/month (median 30, IQR 12-30), attack duration 2-168 hours (median 12, IQR 5-72) and 81% had bilateral headache. PS were reported by 93% (range 0-7; mood change and tiredness the most common), CAS by 58% (range 0-6; pallor and lacrimation the most common), and premonitory CAS by 23%. Vertigo (53%) and craniofacial allodynia (16%) were also present. Headache and CAS laterality moderately agreed (*k*=0.5, *P*<0.001). Average disease duration significantly predicted the number of PS, *F* (1,103) = 15.408, *P*<0.001). There was slight agreement between loud sounds as a trigger and attack-related phonophobia (*k*=0.2, *P*=0.002). Allodynia and vertigo (*k*=0.2, *P*=0.009) and allodynia and neck stiffness (*k*=0.2, *P*=0.007) showed slight agreement.

**Conclusion:** The extended paediatric migraine phenotype includes PS, CAS, vertigo and allodynia. CAS lateralise with headache and should not deter from a migraine diagnosis, despite shorter attack durations in children. There is a more enriched PS phenotype with disease chronicity. The perception of loud sounds as a trigger may be the misattribution of attack-related phonophobia. The co-reporting of some migraine symptoms may allude to shared neural substrates. Further systematic evaluation of similar potential trigger-symptom associations in larger samples is required.

## AL009 Ubrogepant for the acute treatment of migraine when administered during the prodrome (premonitory phase): results from a phase 3, randomized, double-blind, placebo-controlled, crossover study

### D. W. Dodick^1^, P. J. Goadsby^2,3^, T. J. Schwedt^1^, R. B. Lipton^4^, C. Liu^5^, K. Lu^5^, S. Y. Yu^5^, L. Severt^5^, M. Finnegan^5^, J. M. Trugman^5^

#### ^1^Mayo Clinic, Phoenix, AZ, United States; ^2^King's College London, London, United Kingdom; ^3^University of California, Los Angeles, CA, United States; ^4^Albert Einstein College of Medicine, Bronx, MA, United States; ^5^AbbVie, Madison, NJ, United States

##### **Correspondence:** P. J. Goadsby

*The Journal of Headache and Pain 2024,* **25(Suppl 1)**:AL009

**Objective:** To evaluate the efficacy, safety, and tolerability of ubrogepant 100 mg when administered during the prodrome (premonitory phase) of a migraine attack. Ubrogepant is a calcitonin gene-related peptide (CGRP) receptor antagonist approved for the acute treatment of migraine. The prodrome is the earliest phase of the migraine attack. This study examined the potential of ubrogepant, when administered during the prodrome, to prevent or attenuate headache and disability.

**Methods:** PRODROME (NCT04492020) was a multicenter, randomized, double-blind, placebo-controlled, crossover trial. Eligible participants treated 2 "qualifying prodrome events," defined as a migraine attack with prodromal symptoms in which the participant was confident a headache would follow within 1-6 hours. The primary endpoint was absence of moderate/severe intensity headache within 24 hours post-dose. Secondary endpoints were absence of moderate/severe intensity headache within 48 hours, ability to function normally over 24 hours, and absence of a headache of any intensity within 24 hours post-dose.

**Results:** The safety population included 480 participants and the modified intent-to-treat population included 477 participants. Absence of moderate/severe intensity headache within 24 hours was achieved following 45.5% of ubrogepant-treated qualifying prodrome events vs 28.6% of placebo-treated events (*P*<0.0001). Absence of moderate/severe intensity headache within 48 hours (40.7% vs 24.6%; *P*<0.0001), ability to function normally over 24 hours (OR=1.66; *P*<0.0001), and absence of headache of any intensity within 24 hours (23.7% vs 13.9%; *P*<0.0001) were achieved at significantly greater rates following ubrogepant-treated events vs placebo.

**Conclusion:** Treatment with ubrogepant 100 mg during the prodrome prevented the development of moderate/severe headache for 24 and 48 hours post-dose and headache of any intensity within 24 hours and reduced functional disability compared with treatment with placebo.

## AL010 Peripheral monocytes differentiation and cytokines inflammatory profile in episodic and chronic migraine: association with disease severity

### R. De Icco^1,2^, R. Greco^2^, M. Corrado^1,2^, F. Bighiani^1,2^, F. Cammarota^1,2^, G. Vaghi^1,2^, A. Zanaboni^1,2^, C. Demartini^1,2^, M. Francavilla^1,2^, S. Facchetti^1,2^, M. Allena^2^, D. Martinelli^2^, E. Guaschino^2^, N. Ghiotto^2^, G. Sances^2^, C. Tassorelli^1,2^

#### ^1^University of Pavia, Department of Brain and Behavioral Sciences, Pavia, Italy; ^2^IRCCS Mondino Foundation, Headache Science and Neurorehabilitation Centre, Pavia, Italy

##### **Correspondence:** R. De Icco

*The Journal of Headache and Pain 2024,* **25(Suppl 1)**:AL010

**Objective:** Studies examining differences in peripheral inflammatory markers between episodic (EM) and chronic migraine patients did not provide definite consensus. The present study aims to compare cytokines gene expression and monocytes differentiation in EM and chronic migraine with medication overuse headache (CM) patients.

**Methods:** We enrolled 50 EM patients (41.6±10.4 years, 75% female, 6.4±3.7 MMDs), 34 CM patients (46.5±11.3 years, 85% female, 22.6±6.3 MMDs), and 30 healthy controls (HCs, 42.9±14.8, 67% female). We assessed in peripheral blood monocytes the interictal gene expression of pro-inflammatory (IL-1β and TNF-α) and anti-inflammatory (IL-10) cytokines (rtPCR - Relative Quantification). In a subset of patients (18 EM, 24 CM, and 17 HCs), we differentiated monocytes in 4 phenotypes by means of FACS Melody sorter (expressed as number of events for 1 million monocytes): classical (CD14+) / non-classical (CD16+) and M1 (pro-inflammatory – CD80+) / M2 (anti-inflammatory – CD163+).

**Results:** IL-1β and TNF-α expression was higher in EM (IL-1β: 1.17±0.3, TNF-α: 1.11±0.3) and CM (IL-1β: 1.82±1.0, TNF-α: 1.11±0.27) when compared to HCs (IL-1β: 0.39±0.1, TNF-α: 0.39±0.2) (*p*=0.001). In addition, expression of IL-1β and TNF-α was higher in CM when compared to EM (*p*=0.001). IL-10 expression was lower in EM (0.81±0.3) and CM (0.64±0.2) when compared to HCs (1.54±0.6, *p*=0.001), without differences between EM and CM (*p*=0.132). CD16+/163+ and CD14+/80+ monocytes percentage Sorter-related events were higher in migraine patients when compared to HCs (*p*<0.003), with CD14+/80+ being higher in CM when compared to EM (*p*=0.001). CD16+/80+ and CD14+/163+ monocytes percentage events were higher in CM when compared to EM and HCs (*p*=0.001 for all comparisons).

**Conclusion:** Our cohort of migraine patients was characterized by a more pro-inflammatory oriented profile, as demonstrated by: i) increased expression of IL-1β and TNF-α; ii) inhibited expression of IL-10; and iii) increased differentiation of classical and non-classical monocytes toward M1 and M2 phenotypes. Noteworthy, most of these features were more pronounced in CM when compared to EM patients.

## AL011 The impact of hypocretin receptor 1 rs2271933 polymorphism on sleep parameters in chronic migraine patients

### H. Genç^1^, E. U. Özçelik^2^, A. Özge^3^

#### ^1^Gaziantep Dr. Ersin Arslan Training and Research Hospital, Neurology, Gaziantep, Turkey; ^2^Istanbul Kanuni Sultan Süleyman Training and Research Hospital, Neurology, İstanbul, Turkey; ^3^Mersin University Medical Faculty, Neurology, Mersin, Turkey

##### **Correspondence:** E. U. Özçelik

*The Journal of Headache and Pain 2024,* **25(Suppl 1)**:AL011

**Objective:** Studies conducted better to understand long-reported comorbidity between migraine and sleep disorders show that genetic factors and certain gene variations may play a role in the relationship between sleep quality and migraine. This study aimed to examine the relationship of the hypocretin receptor 1 (HCRTR1) rs2271933 gene with sleep parameters.

**Methods:** The present study was designed prospectively in the Mersin University Neurology Clinic between January 2000 and February 2018. Patients aged 18-75 years with chronic migraine (CM) according to the International Classification of Headache Disorders-3 (ICHD-3) criteria were included. The Turkish version of the Pittsburgh Sleep Quality Index (PSQI) was used to evaluate the sleep quality of the patients. Genotyping was performed for the HCRTR1 rs2271933 gene.

**Results:** This study examined 67 patients with CM who have poor sleep quality. The mean age of patients with poor sleep quality was 40.9±11.8%, and the female rate was 89.6%. It was observed that the time to fall asleep increased (*p*=0.369), and the rate of poor sleep quality increased (*p*=0.461) with the increase of G allele carrier, but there wasn't a statistical difference (*p*=0.369). Finally, it was determined that the sleep duration shortened as the ratio of carriers of the G allele increased, and this difference was statistically significant (*p*=0.016).

**Conclusion:** As the G allele carrier of the HCRTR1 rs2271933 gene increased, a shorter sleep duration was observed. As the G allele carrier of the HCRTR1 rs2271933 gene increased, worsening sleep quality and prolonging sleep latency was remarkable, although not statistically significant. We think, based on these results, that these findings may contribute to studies on the physiological roles of orexins.

**Keywords:** Chronic migraine, HCRTR1 rs2271933, poor sleep quality, sleep latency, sleep duration

## AL012 FGF-21 and GDF-15 are increased in Migraine and associated with the severity of migraine-related disability

### K. Liu, J. He, M. Zhou, F. Zhao

#### The Second Affiliated Hospital, Zhejiang University School of Medicine, Department of Neurology, Hangzhou, China

##### **Correspondence:** K. Liu

*The Journal of Headache and Pain 2024,* **25(Suppl 1)**:AL012

**Objective:** Migraine is a highly prevalent disorder that imposes a significant socioeconomic burden. The diagnosis of migraine is based on clinical features and lacks specific biomarkers. Some research has found that mitochondria play an important role in the pathogenesis of migraine. Fibroblast growth factor-21 (FGF-21) and growth differentiation factor-15 (GDF-15) are considered biomarkers for mitochondrial disorders. The aim of this study was to determine whether the serum levels of FGF-21 and GDF-15 in migraine sufferers differ from those in healthy controls and are associated with the severity of migraine-related disability.

**Methods:** The serum concentrations of FGF-21 and GDF-15 were measured using an ELISA-based approach. Clinical variables, including monthly headache days, peak headache pain intensity, the 6-item Headache Impact Test (HIT-6), and the Migraine Disability Assessment (MIDAS), were also addressed. The associations between the clinical variables of migraine patients and serum levels of FGF-21 and GDF-15 were studied.

**Results:** We collected serum samples from 221 migraine patients (153 episodic migraineurs and 68 chronic migraineurs) and 124 healthy controls. In the multiple regression that corrected for identified confounders, we found that the serum levels of FGF-21 and GDF-15 were significantly higher in migraine sufferers than in healthy controls. Regarding quality of life, higher scores on the HIT-6 and MIDAS were associated with higher levels of FGF-21 and GDF-15. For the ROC analysis, the diagnosis of migraine using GDF-15 showed that the area under the ROC curve (AUC) was 0.801 and the AUC of chronic migraine was 0.880.

**Conclusion:** The results revealed that FGF-21 and GDF-15 levels were significantly higher in migraine sufferers than in healthy controls and were strongly associated with the severity of migraine-related disability. Our findings may contribute to a better understanding of the underlying mechanisms of brain mitochondrial metabolism dysfunction in migraine.

**Fig. 1 (Abstract AL012):**
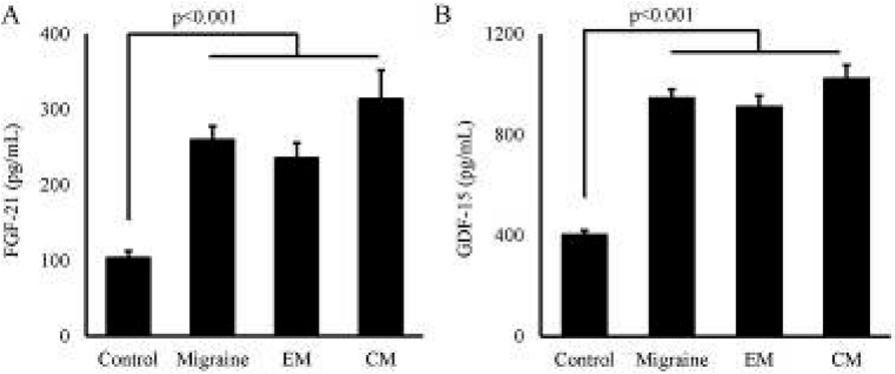
See text for description

**Fig. 2 (Abstract AL012):**
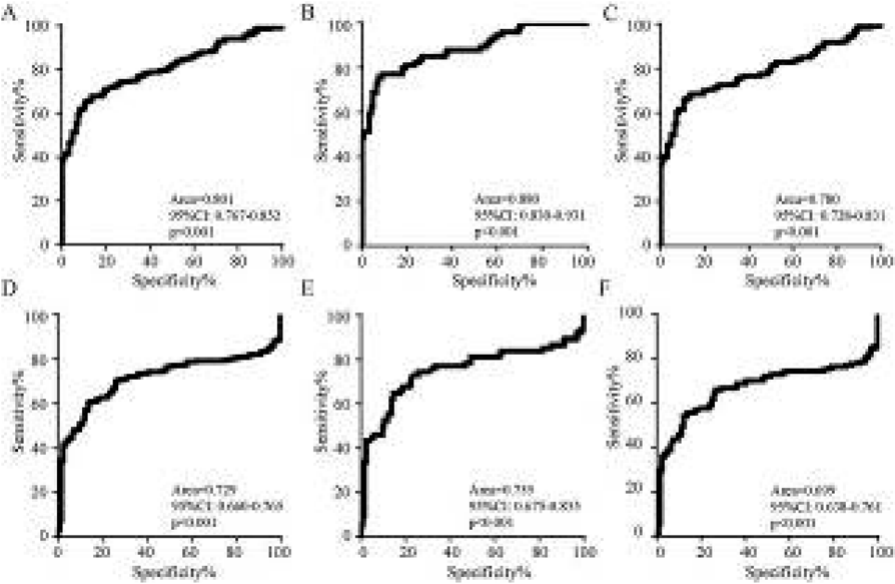
See text for description

**Fig. 3 (Abstract AL012):**
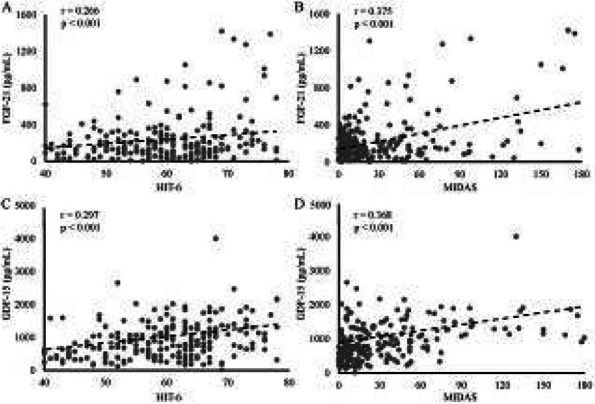
See text for description

## AL013 RE-START STUDY: Multicenter study to assess response after reintroduction of anti-CGRP antibodies in patients with migraine

### A. González Martínez^1^, C. Romero del Rincón^1^, S. Quintas Gutierrez^1^, D. García Azorín^2^, I. Fernández Lázaro^1^, Á. L. Guerrero Peral^2^, Y. González Osorio^2^, F. Iglesias Díez^3^, A. Echavarría Íñiguez^3^, S. Gil Luque^4^, M. Huerta Villanueva^5^, S. Campoy Díaz^5^, A. Muñoz Vendrell^5^, F. Velasco Juanes^5,6^, A. Lozano Ros^7^, A. Sánchez Soblechero^7^, N. Morollón Sánchez-Mateos^8^, R. Belvis^8^, I. Kortazar Zubizarreta^9^, A. Echeverria Urabayen^9^, J. S. Rodrígue-Vico^10^, A. Jaimes Sanchez^10^, A. Gómez García^10^, M. P. Navarro Pérez^11^, N. Montes^1^, A. Gago-Viega^1^

#### ^1^Hospital Universitario de la Princesa, Neurology, Madrid, Spain; ^2^Headache Unit, Neurology Department, Hospital Clínico Universitario de Valladolid, Valladolid,, Valladolid, Spain; ^3^Hospital de Burgos, Neurology Department, Burgos, Spain; ^4^Headache Unit, Neurology Department, Burgos, Spain; ^5^Sección de Neurología, Hospital de Viladecans-IDIBELL, Viladecans, Barcelona, Spain Servicio de Neurología, Unidad de cefaleas, Hospital Universitari de Bellvitge-IDIBELL, L’Hospitalet de Llobregat, Barcelona, Spain, Viladecans, Spain; ^6^Hospital de Cruces, Cruces, Spain; ^7^Headache Unit, Neurology Department, Madrid, Spain; ^8^Hospital de la Santa Creu i Sant Pau, Barcelona, Spain; ^9^Hospital de Álava Department of Neurology, Bioaraba Health Research Institute, Araba University Hospital-Txagorritxu, Vitoria-Gasteiz, Spain, Vitoria-Gasteiz, Spain; ^10^Headache Unit, Neurology Department, Madrid, Spain; ^11^Hospital Obispo Polanco de Teruel, Teruel, Spain

##### **Correspondence:** A. González Martínez

*The Journal of Headache and Pain 2024,* **25(Suppl 1)**: AL013

**Objective:** According to recent European guidelines, suspension of the anti-CGRP antibody should be considered after 12-18 months, although a high percentage of patients may present a significant worsening after that. This study analyzed the effectiveness of the reintroduction of treatment, in absolute terms and in comparison with the first cycle.

**Methods:** Multicenter, observational, prospective study including patients with migraine under anti-CGRP treatment performed at 10 Headache Units. The number of headache days per month (HDM) and migraine days per month (MDM) was recorded at four times: prior to starting anti-CGRP (T-Basal), last month of the first cycle (T-Suspension), at worsening (T -Worsening) and 3 months after reintroduction (T-Reintroduction). The effectiveness of the second cycle (improvement in HDM and MDM at T-Reintroduction compared to T-Worsening >30%) was measured and the response of the second cycle (T-Reintroduction) was compared with the situation at the end of the first cycle (T- Suspension).

**Results:** *N*=152. 85% women, mean age 48 years. The median of HDM and MDM were: 1) T-Basal: 22 (IQR=13), 14 (IQR=8); 2) T-Suspension: 6 (IQR=7), 4 (IQR=5.75); 3) T-Worsening: 16 (IQR=10.25), 11 (IQR=7); 4) T-Reintroduction: 7 (IQR=7.25), 5 (IQR=6.50). In DCM: The 2nd cycle was effective in 151/152 (99%). 79/152(52%) did not obtain the same response: 54/79(68%) worsened 1-5 days, 19/79(24%) 6-10 days and 6/79(7%) >10 days. In DMM it was effective in 131/138 (95%). They did not achieve the same response in 75/152(49%): 55/75(73%) worsened 1-5 days, 12/75(16%) from 6-10 days and 8/75(11%) >10 days.

**Conclusion:** These results suggest that the reintroduction of the treatment is effective in the majority of patients. However, at 3 months half of the sample did not achieve the improvement of the first cycle.

## AL014 Psychological profiles of super responders and non-responders to CGRP-monoclonal antibodies: data from a 6-month follow-up

### S. Bottiroli^1,2^, G. Vaghi^1,3^, R. De Icco^1,3^, D. Martinelli^1^, M. M. Pocora^1,3^, G. Sances^1^, C. Tassorelli^1,3^

#### ^1^IRCCS Mondino Foundation, Pavia, Italy; ^2^Giustino Fortunato University, Benevento, Italy; ^3^University of Pavia, Pavia, Italy

##### **Correspondence:** S. Bottiroli

*The Journal of Headache and Pain 2024,* **25(Suppl 1)**: AL014

**Objective:** To evaluate the psychological predictors of a super response to anti-CGRP monoclonal antibodies (mAbs) in a 6-month follow-up in chronic migraine (CM) or episodic migraine (EM).

**Methods:** One hundred and sixteen patients (age: 48.2±10.5, F: 77%) with CM or EM who had already failed at least three preventive therapies underwent treatment with CGRP-targeting mAbs. At baseline (T0), patients received a full psychological evaluation comprising mood, anxiety, and personality disorders as well as childhood traumas, current stressors and alexithymia. Patients were then followed up at 6 months for their clinical condition.

**Results:** At the 6-month follow-up, 41% of patients (age: 49.7±8.8, F: 81%) reported a reduction of at least 75% in monthly migraine days (MMD) (Super Responder, SR); whereas 16% (age: 49.6±11.9, F: 74%) a ≤ 25% MMD reduction with respect to T0 (Non Responders, NR). When compared to SR, NR patients were characterized by a higher prevalence of anxiety (90% vs 57%, *p*=.012) and personality disorders (94% vs 34%, *p*=.003). They also showed a higher number of very serious current stressors (2.0±3.1 vs 0.2±0.7, *p*<.001) as well as more alexithymic traits (53.6±13.4 vs 43.5±12.9, *p*=.005). The SR and NR groups were instead similar as regards mood disorders and childhood traumas.

**Conclusion:** We confirm the marked effectiveness of CGRP-targeting mAbs also in patients with difficult-to-treat forms of migraine and a high burden of psychological comorbidities. Our results, although preliminary, show that patients who achieve two extreme responses (super response vs. absolute nonresponse) to mAbs also significantly differ in their psychological profiles. In particular, our data highlight the impact of an "anxious-fearful" personality, anxiety, current stressors and alexithymic traits in those particularly refractory to many preventive treatments, including mAbs.

## AL015 Safety and tolerability of combining CGRP monoclonal antibodies with Gepants in patients with migraine: a retrospective study

### T. Alsaadi, V. Santos, I. Al Qaissi, B. Aldaher, A. Al Fardan, Y. Bader, R. Suliman

#### American Center for Psychiatry and Neurology, Neurology, Abu Dhabi, United Arab Emirates

##### **Correspondence:** T. Alsaadi, V. Santos

*The Journal of Headache and Pain 2024,* **25(Suppl 1)**: AL015

**Objective:** The introduction of CGRP based therapies has revolutionized the treatment of migraines. There are many health care providers combining CGRP based therapies in clinical practice. However, very limited data is available regarding the safety and tolerability of this practice. We sought to design this study to evaluate the safety and tolerability of combining CGRP mAbs with Gepants in the management of migraines.

**Methods:** This was a retrospective, real-world, exploratory study. The participants included within the study were adult (≥18 years) patients diagnosed with migraine. We screened for patients who were treated with at least one GCRP mAbs. Data was collected from one site, the American Center for Psychiatry and Neurology, Abu Dhabi UAE. A total of 409 patients taking CGRP mAbs were identified. Extracted data from patients electronic medical records included patient demographics, migraine characteristics, prescribed treatments and AEs. The tolerability and safety of the combination therapy with Gepants was evaluated based on documented AEs.

**Results:** Among the identified 409 patients, 136 were administered Gepants in addition to mAbs (128, Rimegepant and 8, Ubrogepant). Later during the study, 10 out of 128 patients switched from Rimagepant to Urogepant, and similarly, 1 out of 8 patients switched from Urogepant to Zolmitriptan, due to the ineffectiveness of the former medication. Out of all the patients included in this study, 3 AEs were documented. These AEs were generally mild and transient hence, this did not lead to discontinuation of treatment. Moreover, 53 out of the 409 (13.2%) patients were switched from one class of CGRP-mAbs to another, at least once, during this study and while continuing Gepants

**Conclusion:** The finding of this study demonstrates that combining CGRP mAbs with Gepants is a safe and well-tolerated treatment approach for migraine. Future studies are warranted to further validate this finding and explore long-term outcomes.

## AL016 TRPM3 channels as new therapeutic targets for sex dimorphism in migraine

### E. Rivera-Mancilla^1^, A. J. P. Vincent^2^, A. H. J. Danser^1^, A. MaassenVanDenBrink^1^

#### ^1^Erasmus MC University Medical Center, Division of Vascular Medicine and Pharmacology, Department of Internal Medicine, Rotterdam, Netherlands; ^2^Erasmus MC University Medical Center, Department of Neurosurgery, Rotterdam, Netherlands

##### **Correspondence:** E. Rivera-Mancilla

*The Journal of Headache and Pain 2024,* **25(Suppl 1)**: AL016

**Objective:** The mechanisms behind sex dimorphism in migraine are not fully characterized. However, it has been suggested that transient receptor potential (TRP) channels may be involved. We investigated the differential vascular responses of TRP channel agonists in human isolated middle meningeal arteries (HMMAs, a predictive vascular model of antimigraine action) from both women and men.

**Methods:** In segments of HMMAs, concentration-response curves to the agonist cinnamaldehyde (TRPA1), pregnenolone sulfate (PregS, TRPM3), or capsaicin (TRPV1) were constructed to obtain the maximum contractile response (E_max_) in the absence or presence of the antagonists HC-030031 (TRPA1), isosakuranetin (TRPM3) or capsazepine (TRPV1). Moreover, the role of CGRP was evaluated using the antagonist olcegepant. Since NMDA receptors are modulated by PregS, we also evaluated the effect of the antagonist MK-801 in the PregS-induced vascular responses.

**Results:** In HMMAs from both women and men, the TRP channel agonists induced concentration-dependent relaxation responses. The E_max_ to PregS (women: E_max_ 46±4% vs men: E_max_ 28±2%), but not to cinnamaldehyde (women: E_max_ 89±4% vs men: E_max_ 81±5%) or capsaicin (women: E_max_ 107±6% vs men: E_max_ 109±5%), was significantly higher in women than in men. Moreover, the E_max_ to: (i) capsaicin was not modified in the presence of capsazepine or olcegepant; (ii) cinnamaldehyde was reduced only in the presence of olcegepant but not by HC-030031; and (iii) PregS was significantly inhibited in the presence of isosakuranetin or olcegepant; while in the presence of MK-801, it was only inhibited in HMMAs from women, but not in men.

**Conclusion:** Unlike TRPA1 or TRPV1, which seems not to be involved in the cinnamaldehyde- or capsaicin-induced relaxation, targeting TRPM3 may represent a new therapeutic option for sex dimorphism in migraine. Further studies should analyze the differential role of NMDA receptors and their specific role for migraine therapy.

## AL017 MADRE MIA: Monoclonal Antibody Duration of REsponse in MIgraine After treatment interruption: A prospective multicentric study on 2164 patients

### A. Echavarría Íñiguez^1^, Á. L. Guerrero Peral^2^, J. Diaz de Teherán^3^, S. Santos Lasaosa^4^, E. Cuadrado Godia^5^, M. Martín Bujanda^6^, N. Riesco Pérez^7^, F. Velasco Juanes^8^, A. Sánchez Soblechero^9^, F. Iglesias Díez^10^, M. T. Temprano Fernández^11^, V. Obach Baurier^12^, J. C. García-Moncó^13^, S. Aranceta Arilla^14^, A. J. Sánchez^15^, A. Mínguez-Olaondo^16^, A. Ruisanchez Nieva^17^, J. Porta-Etessam^18^, I. Kortazar Zubizarreta^19^, A. López-Bravo^20^, M. Huerta Villanueva^21^, A. Gago-Viega^22^, C. González Oria^23^, A. A. López^24^, N. Morollón Sánchez-Mateos^25^, A. Sanz Castrillo^26^, C. Treviño^27^, Á. Sierra-Mencía^2^, A. Recío García^2^, J. A. Membrilla López^3^, M. P. Navarro Pérez^4,28^, P. Manera Zorrilla-Lequerica^5^, M. d. R. Álvarez Escudero^7^, A. Lozano Ros^9^, S. Gil Luque^10^, M. Alvarez^11^, N. Fabregat Fabra^12^, N. Roncero Colina^13^, J. S. Rodriguez-Vico^15^, M. Ruibal Salgado^16^, N. González García^18^, A. Echeverria Urabayen^19^, A. Muñoz Vendrell^21^, I. Fernández Lázaro^22^, C. Romero del Rincón^22^, R. Lamas Pérez^23^, A. Layos-Romero^24^, R. Belvis Nieto^25^, Y. González Osorio^2^, D. Guisado-Alonso^5^, A. Oterino Durán^7^, J. Trigo López^10^, L. González-Fernández^11^, S. Fernández-Fernández^12^, H. Martín Rodriguez^13^, A. Gómez García^15^, S. Campoy Díaz^21^, S. Quintas Gutierrez^22^, N. Sánchez Rodriguez^23^, M. T. Sánchez Sánchez^2^, T. Marco Galindo^12^, S. Fernández Peña^2^, M. D. L. Ibañez dela Cadiniere^15^, A. González Martínez^22^, D. García Azorín^2^

#### ^1^Hospital Universitario de Burgos, Department of Neurology, Burgos, Spain; ^2^Hospital Clínico Universitario de Valladolid, Valladolid, Spain; ^3^Hospital Universitario La Paz, Madrid, Spain; ^4^Hospital Clinico Universitario Lozano Blesa, Zaragoza, Spain; ^5^Hospital del Mar, Barcelona, Spain; ^6^Hospital Universitario de Navarra, Pamplona, Spain; ^7^Hospital Universitario Central de Asturias, Oviedo, Spain; ^8^Hospital Universitario de Cruces, Bilbao, Spain; ^9^Hospital General Universitario Gregorio Maranon, Madrid, Spain; ^10^Hospital Universitario de Burgos, Burgos, Spain; ^11^Hospital de Cabueñes, Gijón, Spain; ^12^Hospital Clinic de Barcelona, Barcelona, Spain; ^13^Hospital Universitario de Basurto, Bilbao, Spain; ^14^Parc Taulí Hospital Universitari, Barcelona, Spain; ^15^Fundación Jiménez Díaz, Madrid, Spain; ^16^Hospital Universitario de San Sebastian, Donostia, Spain; ^17^Hospital Universitario de Galdakao, Bizkaia, Spain; ^18^Hospital Clinico San Carlos, Madrid, Spain; ^19^Hospital Universitario de Araba, Vitoria-Gasteiz, Spain; ^20^Hospital Reina Sofía, Tudela de Navarra, Spain; ^21^Hospital Universitario de Bellvitge y Viladecans, Barcelona, Spain; ^22^Hospital Universitario La Princesa, Madrid, Spain; ^23^Hospital Universitario Virgen del Rocio, Sevilla, Spain; ^24^Hospital Universitario de Albacete, Albacete, Spain; ^25^Hospital de la Santa Creu i Sant Pau, Barcelona, Spain; ^26^Complejo Asistencial de Segovia, Segovia, Spain; ^27^Hospital Universitario Severo Ochoa, Madrid, Spain; ^28^Hospital Obispo Polanco de Teruel, Teruel, Spain

##### **Correspondence:** D. García Azorín

*The Journal of Headache and Pain 2024,* **25(Suppl 1)**: AL017

**Objective:** How long does it last the clinical benefit of monoclonal antibodies (mAbs) targeting calcitonin gene-related peptide (CGRP) or its receptor in patients with migraine who achieved a positive response after treatment discontinuation?

**Methods:** Prospective cohort multicentric (*n*=27) study including patients with high-frequency episodic migraine (HFEM) or chronic migraine (CM) who were treated with mAbs according to the national criteria prior failure to at least three migraine prophylactic drugs, and achieved a positive response. The study protocol was registered in ClinicalTrials.gov (NCT05232942). A series of demographic and clinical variables were collected. The duration of the response was assessed by survival estimates and Kaplan-Meier curves, and the survival event was defined as the time elapsed until the return of the pre-mAb situation, the re-start of the mAb or the use of another migraine prophylactic treatment. The study was funded by the Regional Health Administration (Gerencia Regional de Salud, SACYL, Castilla y León, GRS 2578/A/2022).

**Results:** A total of 2,164 patients were enrolled, aged 48.4 [inter-quartile range (IQR): 41.6-56.4], 84.7% female. Migraine type corresponded to HFEM in 21.4% and CM in 78.6% cases, and 22% had migraine with aura. The median number of prior prophylactic treatments was 5 [IQR: 4-6]. The median duration of the mAb treatment prior to its discontinuation was 11.9 [IQR: 11-13] months. After mAb treatment, patients presented a statistically significant reduction in the number of headache days per month, migraine days per month, acute treatment days per month, triptan days per month and median intensity of the headache. The employed mAb was galcanezumab (53.5%), fremanezumab (23.5%, 29.9% with quarterly administration), erenumab (22.6%, 57.7% with 140 mg dose) and eptinezumab (0.3%). The median duration of the response was 4.7 [IQR: 3.96-5.56] months.

**Conclusion:** The duration of the benefit of anti-CGRP mAbs was not homogeneous and varied between patients, with some patients presenting a prompt relapse and others with a sustained clinical benefit.

## AL018 The role of purity and frequency in the classification of perimenstrual headache

### W. Na^1,2^, H. Liu^1^, Y. Liu^1^, X. Wang^1^, S. Yu^1^

#### ^1^The First Medical Center, Chinese PLA General Hospital, Department of Neurology, Beijing, China; ^2^Nankai University, School of Medicine, Tianjin, China

##### **Correspondence:** W. Na

*The Journal of Headache and Pain 2024,* **25(Suppl 1)**: AL018

**Objective:** International Classification of Headache Disorders 3nd 3rd edition identified classifies menstrual migraine in aspects ofaccording to headache type, timing (on days -2 to +3 of menstruation), frequency (whether having a headache occurs in at least two out of three menstrual cycles), and purity (whether having a headache occurs at other times of the menstrual cycle) of headache, and it providedprovides a reference for research on other menstruation-associated headache. The role of frequency and purity in the classification of menstruation-associated headache is not clear. The potential risk factor factors for high-frequency and pure headaches ishave not been explored.

**Methods:** The study was a secondary analysis of a survey on menstrual migraine among nurses. Among those nurses who havehad a headache in on days -2 to +3 of menstruation, headache frequency, purity, and type were described. High-frequency vs. low-frequency and pure vs. impure headache were compared in according to headache features, demographics, occupation-related factors, menstruation-related factors, and lifestyle factors.

**Results:** Of all the respondents, 254(18.3%) nurses who have had headachesheadache on days -2 to +3 of menstruation were included in the study. In the 254 nurses with perimenstrual headache, proportion the proportions of migraine, tension type headache (TTH), high-frequency headache, and pure headache were 24.4%, 26.4%, 39.0%, and 42.1%, respectively. High-frequency and impure perimenstrual headache is was more severe and approximate similar to migraine. High-frequency headache is was associated with more perimenstrual extremity swelling and generalized pain. Other variables were not statistically significantly different between the groups.

**Conclusion:** Headache except for menstrual migraine makes upaccounts for a certain proportion of menstruation-associated headache and should not be ignored. The Frequency and purity are related to headache type and should be equally treated considered in the classification of menstruation-associated headache. Perimenstrual extremity swelling and generalized pain are potential indicators of high-frequency headache.

**Fig. 1 (Abstract AL018):**
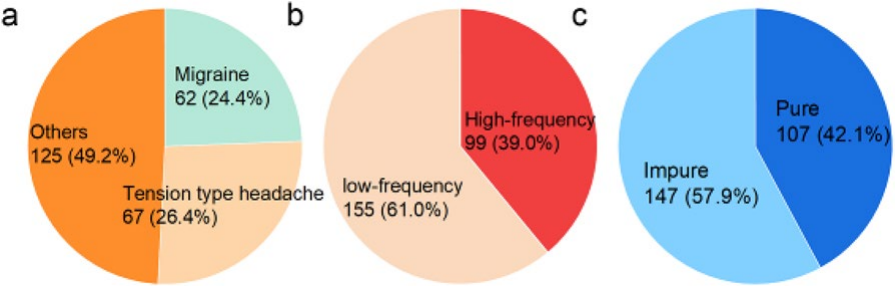
See text for description

**Fig. 2 (Abstract AL018):**
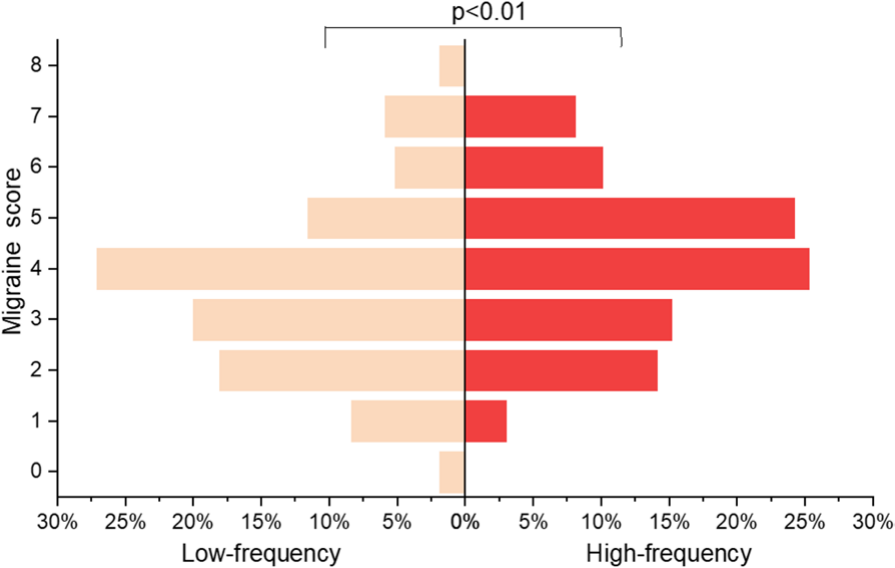
See text for description

**Fig. 3 (Abstract AL018):**
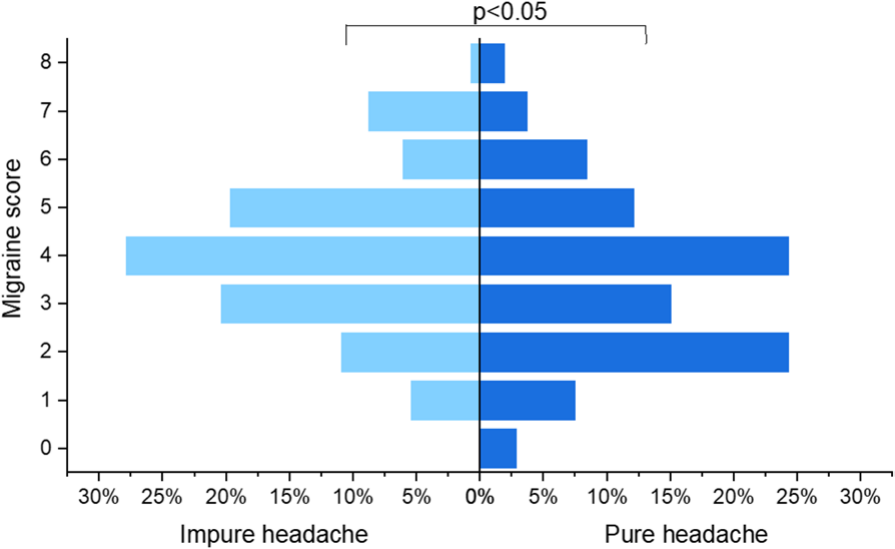
See text for description

## AL019 PPPD and vestibular migraine: How can we differentiate between these entities?

### A. Zubaidy, D. Moreno Ajona, M. D. Villar-Martínez, N. Karsan, P. J. Goadsby

#### King's College London, Institute of Psychiatry, Psychology & Neuroscience Wolfson Centre for Age-Related Diseases, London, United Kingdom

##### **Correspondence:** D. Moreno Ajona

*The Journal of Headache and Pain 2024,* **25(Suppl 1)**: AL019

**Objective:** Persistent postural perceptual dizziness or PPPD has been recently considered as one of the main causes of dizziness if not the main in NeuroOtology Clinics. In essence, PPPD was called functional vertigo or phobic vertigo in the past. As for migraine and vestibular migraine, the diagnosis of PPPD is entirely clinical. Recent research has shown that up to 80% of patients with PPPD associate headaches, particular migraine headache. The Niigata PPPD Questionnaire (NPQ) has been validated for the evaluation of PPPD stablishing cut-off point of 27 and 29 as characteristic. The NPQ evaluates the severity of symptoms in three categories: upright posture/walking, movement, and visual stimulation, for each one of them the cut-off point has been stablished at 9 as characteristic of PPPD. We set to determine if patients with vestibular migraine or migraine experience similar symptoms and to a similar degree to patients with PPPD.

**Methods:** We audited the results of the questionnaires handed to patients in the waiting room of the General Neurology Clinic and Headache Clinic at Queen Elizabeth Hospital and King's College Hospital, London, UK. The questionnaires included the three-item ID migraine and if positive included questions of migraine canonical symptoms as well as the NPQ. Means and medians were compared using Student T test and U of Mann-Whitney.

**Results:** Out of 120 patients who filled the assessments 88 had positive 3-item ID migraine of which 75 filled the NPQ. The median age was 48 (IQR 20, 65) and 73% were females. The total NPQ median value was 29 (IQR= 11, 47) and for the upright posture/walking was 10 (IQR= 4, 16), movement (IQR= 6, 16), and visual stimulation was 10 (IQR= 3, 17). Interestingly, there was a good correlation between the total NPQ values and the number of monthly headache days (NPQ= 29, MHD= 20, *p*=0.02). Additionally, the NPQ was signifficantly higher in those patients who presented with movement sensitivity as one of their migraine canonical symptoms (*p* = 0.036).

**Conclusion:** The previously validated NPQ scale for PPPD showed similar scores in patients with migraine and vestibular migraine suggesting the distinction between both entities might not be clear.

## AL020 The prevalence of headache disorders among epilepsy patients in the Trøndelag Health Study (HUNT)

### H. Engstrand^1,2,3^, E. Revdal^4,5^, K. Hagen^2,5,6^, M. B. Argren^2,3^, J. A. Zwart^1,2,7^, E. Brodtkorb^4,5^, B. S. Winsvold^1,2,3^

#### ^1^Oslo University Hospital, Department og Research and Innovation, Division of Clinical Neuroscience, Oslo, Norway; ^2^Norwegian University of Science and Technology, Norwegian Centre for Headache Research (NorHEAD), Trondheim, Norway; ^3^Oslo University Hospital, Department of Neurology, Oslo, Norway; ^4^St. Olavs University Hospital, Department of Neuromedicine and Movement Science (INB), Trondheim, Norway; ^5^Norwegian University of Science and Technology, Department of Neuromedicine and Movement Science (INB), Trondheim, Norway; ^6^St. Olavs University Hospital, Clinical Research Unit Central Norway, Trondheim, Norway; ^7^Faculty of Medicine, Institute of Clinical Medicine, Oslo, Norway

##### **Correspondence:** H. Engstrand

*The Journal of Headache and Pain 2024,* **25(Suppl 1)**: AL020

**Objective:** Is epilepsy associated with migraine and headache in a large population-based study?

**Methods:** In total 65,408 adult (≥20 years) residents of the Nord-Trøndelag county of Norway participated in the HUNT2 and/or HUNT3 studies, and classified for headache disorders by a validated questionnaire. We used two separate definitions of epilepsy: 1) hospital diagnostic (ICD) codes for epilepsy from a neurological or pediatric department on ≥2 occasions between 1987 and 2019 (*n* = 513), and 2) validated and classified epilepsy by review of the medical records of a selection of those with hospital diagnosed epilepsy (*n* = 364). The remaining population without epilepsy, were used as controls (*n* = 63,299). The association of migraine and non-migraine headache to epilepsy and epilepsy subgroups were analyzed using logistic regression, adjusting for sex and age.

**Results:** The prevalence was 0.78% for hospital record-defined epilepsy. Of 364 patients with validated epilepsy 281 had focal, 44 generalized and 39 unknown/combined epilepsy. Compared to controls, the odds ratio (OR) for having migraine was 0.99 (95% confidence interval (CI) 0.76-1.30, *p* = 0.958) for hospital-diagnosed epilepsy and 0.96 (95% CI 0.69-1.33, *p* = 0.796) for validated diagnosis of epilepsy. The corresponding numbers for non-migraine headache were OR = 1.17 (95% CI 0.95-1.43, *p* = 0.137), and OR = 1.20 (95% CI 0.94-1.52, *p* = 0.140). We found no association of migraine to epilepsy subtype (focal, generalized, or unknown/combined) or to active vs. non-active epilepsy.

**Conclusion:** In this large population-based study, we found no association between hospital-diagnosed epilepsy and migraine. This contrasts with previous clinical-based studies, which may have been flawed by selection bias or lack of control groups.

## AL021 Medication overuse headache vs. medication overuse, phenotypical differences

### K. Burrows, D. Moreno Ajona, S. Maniataki, P. J. Goadsby

#### King's College London, Institute of Psychiatry, Psychology & Neuroscience Wolfson Centre for Age-Related Diseases, London, United Kingdom

##### **Correspondence:** D. Moreno Ajona

*The Journal of Headache and Pain 2024,* **25(Suppl 1)**: AL021

**Objective:** Medication overuse headache (MOH) is a cause of chronic daily headache whose biological explanation is still under debate. Here, we set to determine the phenotypical differences between patients with and without headaches among those with rheumatoid arthritis who overuse medication by ICHD-3 criteria.

**Methods:** Data from Rheumatology patients seen at the Rheumatology Clinic, King"s College Hospital, London were audited. We included 259 patients from the MOH-R cohort (1) with a diagnosis of rheumatoid arthritis whose clinical letters were examined and 10 patients were contacted to complete their headache history.

**Results:** One-hundred-and-four patients were identified as having suspected MO as per the clinical information available and headache (*n* = 104, 88 females). Headache frequency was missing from the Rheumatology letters. The median age was 65 (IQR= 54, 76). Opioids were the most prescribed medication (*n* = 51), followed by paracetamol (*n* = 45) and non-steroidal anti-inflammatory drugs (*n* = 25). The distribution of NSAIDs used was: naproxen 12; ibuprofen, 10; and one each etoricoxib, celecoxib and meloxicam). Out of those who did not take paracetamol (*n* = 56), 33 took an opioid. Out of those who did not take an opioid (*n* = 50), 27 were taking paracetamol. Migraine features documented included canonical symptoms and also presence of aura, cranial autonomic symptoms, premonitory and postdrome symptoms. Migraine features were present in all patients with a definite diagnosis of MOH who were contacted, namely, chronic headache and medication overuse (6/10). However, not all patients with medication overuse and a migrainous features developed a chronic headache (2/10).

**Conclusion:** In a cohort of patients taking analgesics at a frequency that would qualify for the designation of medication overuse, headache is common, although crucially many patients "overusing" analgesics did not develop a headache problem. Patients with medication overuse and headache generally had migrainous symptoms.

**Reference**

1. Moren-Ajona D, Villar-Martinez MD, Futter N, Hoffmann J, Goadsby PJ. Medication overuse headache: Is there a difference between naproxen and paracetamol? Cephalalgia. 2021;41(1S):259.

## AL022 A11 nucleus activation inhibits nitroglycerin-mediated photophobia and nociceptive sensitization in mice

### C. Li, S. Yu

#### Department of Neurology, the First Medical Center of Chinese PLA General Hospital, Medical School of Chinese PLA, Haidian District Beijing, China

##### **Correspondence:** C. Li

*The Journal of Headache and Pain 2024,* **25(Suppl 1)**: AL022

**Objective:** To clarify the role played by the A11 nucleus in the pathophysiology of migraine, we designed experiments to explore

**Methods:** Various techniques such as immunofluorescence staining, western-blot, and neuronal tracing were applied to investigate the role of the A11 nucleus in migraine and its associated structures

**Results:** During acute and chronic attacks in the NTG-mediated mouse migraine model, c-Fos expression of GABAergic neurons in the A11 nucleus was significantly increased, and the inhibition of Dopaminergic (DA) neurons was achieved by binding to GABA A receptors on the surface of DA neurons in the A11 nucleus. In the acute and chronic migraine models, the expression of glutamic acid decarboxylase (GAD) and Tyrosine hydroxylase (TH) protein did not significantly change in the of the A11 nucleus in the hypothalamus. Specific damage to Dopaminergic neurons in the A11 nucleus of mice resulted in severe nociceptive sensitization and photophobic behavior. Injection of cis-tracer viruses into A11 resulted in the observation of projection fibers in the SP5C and LP. Injection of retrograde tracer virus into SP5C and LP regions could capture labeled DA neurons in the A11 region. The expression levels of dopamine receptor in the SP5C region of the chronic migraine model were increased compared with those in the acute migraine model as well as the control group, while there was no significant change in LP region. By activation of the D2DR in the SP5C of the model mouse, it could slow down the pain sensitization and photophobia behavior of mouse skin, while activation of D1DR can reverse this behavioral change. Activation of D2DR in the LP attenuates the flinching behavior of mice to bright light, and activation of D1DR makes mice more sensitive to bright light.

**Conclusion:** GABAergic neurons in the A11 are activated in the NTG-mediated mouse migraine model, and secreted GABA binds to GABA A receptors on the surface of DA neurons in the A11, exerting postsynaptic inhibitory effects, which leads to a decrease in the amount of DA secreted by the A11 nucleus in SP5C and LP, and the reduced DA binds preferentially to the D2DR, which exerts a protective effect against glare and pain resistance

## AL023 Investigating neuropeptides as predictors of early efficacy of monoclonal antibodies therapy in migraine

### E. Rubino^1^, A. Marcinnò^1^, E. Gallo^2^, S. Boschi^1^, F. Roveta^1^, E. M. Piella^1^, F. Ferrandes^1^, A. Grassini^1^, I. Rainero^1^

#### ^1^University of Torino, Department of Neuroscience "Rita Levi Montalcini", Torino, Italy; ^2^“Cardinal Massaia” Hospital, S.O.C. Neurologia, Asti, Italy

##### **Correspondence:** I. Rainero

*The Journal of Headache and Pain 2024,* **25(Suppl 1)**: AL023

**Objective:** Innovative therapies targeting the CGRP signaling opened and established a new era in preventive treatment of migraine. Emerging evidences suggest a role of circulating factors to unravel the neurobiological causes of variability in treatment response. This study aims to combine the use of baseline plasmatic neuropeptides and clinical factors to develop a prediction model of response to anti-CGRP monoclonal antibodies (mAbs).

**Methods:** 41 patients (34 females, 7 males; mean age 52.16 ± 12.47 years; 24 with chronic migraine, and 17 with episodic migraine) started mAbs therapy (7 Erenumab, 17 Galcanezumab and 17 Fremanezumab). During the first visit (T0), plasmatic dosage of CGRP, Orexin-A, and PACAP-38 were performed. Clinical characteristics were collected. The clinical course was re-evaluated at 3 months (T3), based on monthly migraine days (MMD), monthly medication use, mean pain intensity (NRS) and MIDAS. Data were analyzed by Structural Equation Modeling to develop a predictive model of treatment response based on T0 clinical and biochemical characteristics. This multivariate analysis defines new composite variables through quantitative relationships with the observed variables. Thus, we obtained the latent variables NeuP, i.e. "Neuropeptides" (CGRP, PACAP-38 and Orexin-A) and MigBurd T0 i.e. "Migraine burden" (MIDAS and MMD at T0). Then, we correlated them with T3 clinical outcome.

**Results:** CGRP plasmatic concentrations at T0 emerged as a unique independent predictor of therapeutic response at T3 through direct correlation with MMD (100 pg/ml per 1,7 MMD at T3; *p*= 0.032). Through S.E.M. we also found a similar correlation with monthly medication use and MIDAS at T3. The latent variable MigBurd T0 was directly correlated with all three above mentioned parameters, while baseline CGRP prevailed over NeuP latent variable as predictor of MMD and MIDAS at T3.

**Conclusion:** The neurobiological setting may be crucial in the variability of clinical response to mAbs therapy even in the short term. Though further data are needed to generalize these results, the present study supports the predictive role of baseline GCRP plasmatic concentrations in mAbs anti-CGRP therapeutic response.

## AL024 Efficacy of mindfulness added to treatment as usual in patients with chronic migraine and medication overuse headache: a randomized clinical trial, early results

### L. Grazzi, D. A. Montisano, D. D'Amico, E. Guastafierro, B. Del Corso, A. Raggi

#### Foundation IRCCS C. Besta Institute, Milano, Italy

##### **Correspondence:** L. Grazzi, D. A. Montisano

*The Journal of Headache and Pain 2024,* **25(Suppl 1)**: AL024

**Objective:** To assess the efficacy of a six-session mindfulness-based treatment added to treatment as usual (TaU) on headache frequency reduction and medication intake.

**Methods:** This is a phase-III single blind RCT single-center study, carried out at the third-level Italian headache center IRCCS "C.Besta". Patients were enrolled between November 2018 and December 2021, and followed-up for 12 months. 177 patients with Chronic Migraine and Medication Overuse Headache (CM and MOH) were randomized 1:1 to either TaU or mindfulness added to TaU (TaU+MIND). Exclusion criteria were: psychiatric comorbidities; pregnancy; secondary headaches; withdrawal from MOH at least twice in the previous two years; previous experience with mindfulness. TaU consisted of withdrawal from overused drugs, patients" education, and prescription of prophylaxis. Patients attending mindfulness sessions were taught to focus their attention on the present and enhance awareness of body sensations, which enabled tackling the pain-pill automatism, and were encouraged to engage in a 7-10 minute/day self-practice. The primary endpoint was the achievement, at 12 months of ≥50% headache frequency reduction compared to baseline. Secondary endpoints included medication intake.

**Results:** Out or the 177 participants (median age 47.9 years [Q1-Q3: 40.1-54.2]; 19 [11.3%] males; median CM duration 14.6 years [Q1-Q3: 4.9-22.2]) 89 were randomized to TaU and 88 to TaU+MIND. Patients in the TaU+MIND group outperformed those in TaU for the primary endpoint, achievement of ≥50% headache frequency reduction (78.4% vs 48.3%; *p*<0.0001). They also showed superiority in some secondary endpoints, namely headache frequency and medication intake.

**Conclusion:** These findings show that a six-week mindfulness-based treatment as add on to TaU is superior to TaU for the treatment of patient with CM and MOH

## P001 Effects of US-Guided trigger point interventions on ophthalmic artery doppler parameters and eye symptoms in patients with migraine and headache

### R. Bubnov

#### Clinical Hospital Pheophania, Kyiv, Ultrasound, Kyiv, Ukraine

*The Journal of Headache and Pain 2024,* **25(Suppl 1)**: P001

**Objective:** Migraine and headache disorders affect a significant portion of the population, leading to substantial disability and impaired quality of life. The etiology ofis multifactorial, and emerging research suggests the involvement of myofascial pain, abnormal posture, and vascular dysregulation. This study aimed to explore the effects of Ultrasound (US)-guided trigger point interventions on ophthalmic artery Doppler parameters and their potentiabiomarkers for stroke prevention.

**Methods:** A cohort of 13 patients with diagnosed migraine and headache disorders underwent clinical assessments and US-guided trigger point interventions targetmuscles, pterygoid muscles, and shoulder correction. Patients received dry needling of muscle trigger points under US guidance according to the approach by R. Bubnov [EPMA J2012, 3(1):13.]. Doppler analysis of retinal artery, ciliary artery, and ophthalmic artery parameters was conducted pre- and postintervention. Imeye symptoms were evaluated using standardized scales.

**Results:** Following the interventions, significant alterations in ophthalmic artery Doppler parameters were observed. Postintervention, retinal artery exhibited incresystolic velocity (PSV) (11 cm/sec) and decreased resistivity index (RI) from 0.82 to 0.64 (*p* < 0.01). Ciliary artery showed increased PSV (12 cm/sec) afrom 0.78 to 0.67-0.7 (*p* < 0.01). Ophthalmic artery demonstrated a substantial increase in PSV (35 cm/sec) and decreased RI (0.64). These changes coreported improvements in eye symptoms, reduced frequency, duration, and intensity of migraines and headaches.

**Conclusion:** US-guided trigger point interventions may contribute to the normalization of ophthalmic artery Doppler parameters. These findings suggest a potential link between myofascial pain, posture, Flammer syndrome, and the pathophysiology of migraine and headache disorders.

**Fig. 1 (Abstract P001):**
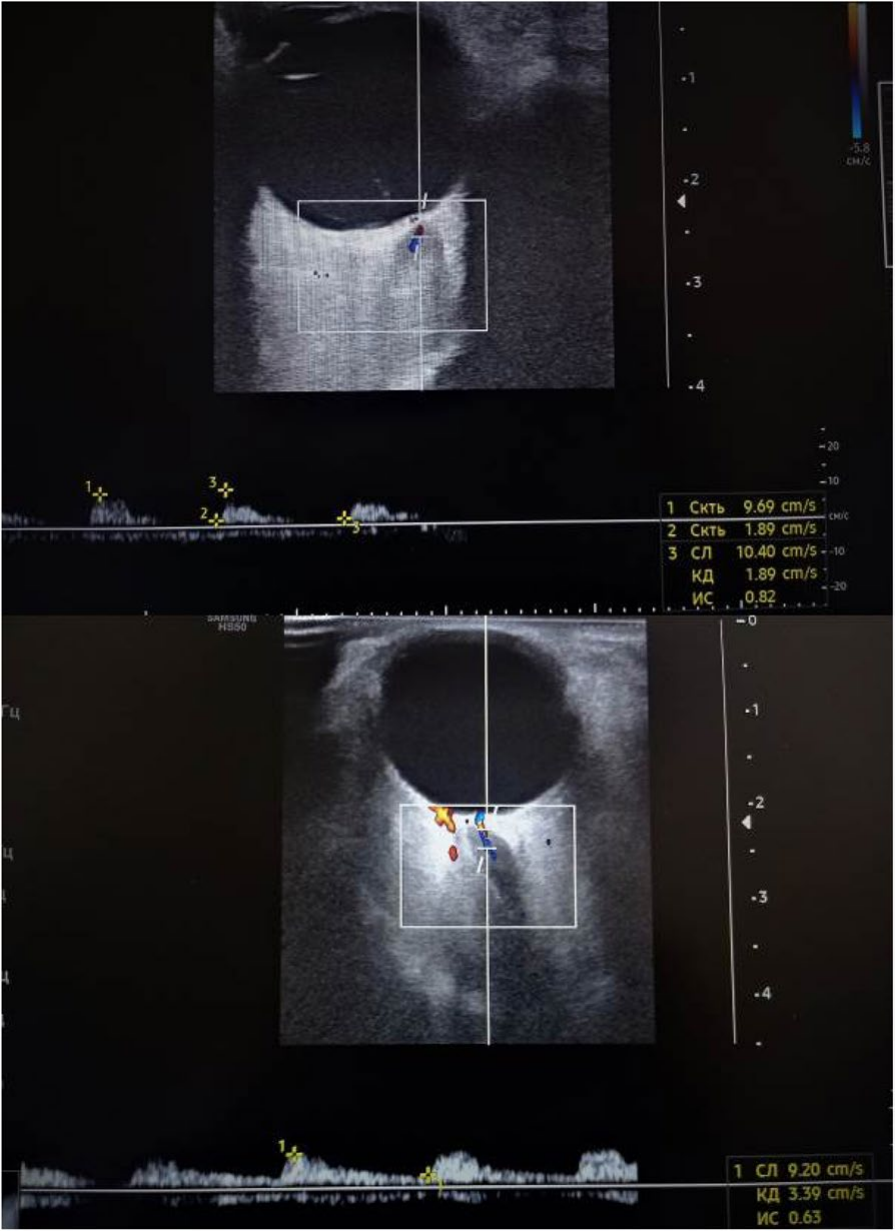
See text for description

## P002 Pre-Treatment cephalic interictal allodynia predicts outcome of prophylactic treatment of chronic and episodic migraine patients with Galcanezumab: a prospective quantitative sensory testing study

### S. Ashina^1,2,3,4,5^, A. Melo-Carrillo^3,4,5^, E. Szabo^3,5^, D. Borsook^6,5^, R. Burstein^3,4,5^

#### ^1^Beth Israel Deaconess Medical Center, Neurology, Boston, MA, United States; ^2^Faculty of Health Sciences, University of Copenhagen, Clinical Medicine, Copenhagen, Denmark; ^3^Beth Israel Deaconess Medical Center, Anesthesia, Critical Care and Pain Medicine, Boston, MA, United States; ^4^BIDMC Comprehensive Headache Center, Brookline, MA, United States; ^5^Harvard Medical School, Boston, MA, United States; ^6^Massachusetts General Hospital, Psychiatry and Radiology, Boston, MA, United States

##### **Correspondence:** S. Ashina

*The Journal of Headache and Pain 2024,* **25(Suppl 1)**: P002

**Objective:** To investigate whether the interictal (rather than ictal) cutaneous allodynia may determine the migraine state, including the outcome of migraine prevention with migraine prophylactic drugs.

**Methods:** Quantitative Sensory testing (QST) was used to determine whether it is possible to predict patients" response to prophylactic treatment with galcanezumab, a monoclonal antibody against calcitonin gene-related peptide (CGRP-mAb), based on the presence of or absence of cephalic and/or extracephalic allodynia during the pre-treatment interictal phase of migraine (i.e., before treatment initiation). We used strict criteria for allodynia (lower than 40^o^C for heat, higher than 20^o^C for cold, lower than 60g for mechanical).

**Results:** We found that the incidence of pre-treatment cephalic interictal allodynia was 21% in the 24 responders (>50% decrease in monthly migraine days, MMD) and 85% in the 19 non-responders. Heat, cold, and mechanical interictal allodynia was detected in 37%, 68%, and 53% of the non-responders, respectively. The incidence of extracephalic interictal allodynia did not distinguish responders from non-responders while the incidence of cephalic interictal allodynia was similar in the chronic migraine and high-frequency episodic migraine groups. The incidence of interictal allodynia was unrelated to the amount of time patients were headache-free before being tested.

**Conclusion:** Clinically, the findings suggest that it is possible and simple to predict galcanezumab responders with nearly 80% accuracy and galcanezumab non-responders with nearly 85% accuracy. Mechanistically, the findings suggest that the establishment of activity-independent central sensitization – the neural correlates of interictal allodynia - is unrelated to the frequency of migraine attacks or the number of hours/days after the termination of the last attack or initiation of the next attack. It is possible that the state of interictal allodynia may be attributed to peripheral and central molecular, cellular and/or physiological properties of central trigeminovascular neurons that are inherent to the genetic load of the individual patient rather than the pathophysiological state of the disease.

## P003 Pharmacokinetics and safety of eptinezumab in children and adolescents with migraine

### A. D. Hershey^1,2^, J. Areberg^3^, L. P. Boserup^3^, A. Lindsten^3^, M. Rosen^3^

#### ^1^Department of Pediatrics, University of Cincinnati, Cincinnati, OH, United States; ^2^University of Cincinnati College of Medicine, Cincinnati, OH, United States; ^3^H. Lundbeck A/S, Copenhagen, Denmark

##### **Correspondence:** L. P. Boserup

*The Journal of Headache and Pain 2024,* **25(Suppl 1)**: P003

**Objective:** To characterize the pharmacokinetic (PK) profile of eptinezumab and explore the safety and tolerability of eptinezumab in pediatric patients with migraine.

**Methods:** This open-label PK study (NCT04537429) evaluated a single intravenous infusion of eptinezumab in children and adolescents (aged 6–17y) with migraine. Eligible patients had a history of ≥4 migraine days/month for ≥3 months prior to screening. The study included a 20-week evaluation period and a 44-week extension period (ongoing). The dose levels (by body weight: ≤20kg, 100mg; >20 to ≤40kg, 150mg; >40kg, 300mg) were based on simulations using a population PK (popPK) model of eptinezumab in adults that was adapted to a pediatric population. Primary PK endpoints, estimated by popPK analysis, were area under the curve (AUC) and maximum concentration (Cmax) of eptinezumab. Safety/tolerability assessments included adverse events (AEs), clinical laboratory tests, vital signs, height, and weight. Exploratory endpoints included change from baseline in Pediatric Migraine Disability Assessment (PedMIDAS).

**Results:** A total of 28 patients were enrolled. Patients were primarily female (20/28) and White (24/28), with a mean age of 12.6y and body weight ranging 21–79kg. A robust and reliable popPK model was created and used for PK parameter estimations. After a single eptinezumab infusion, AUC and Cmax were similar across dose levels and weight groups and overlapped with adult-based prediction models. Four (14.3%) patients experienced ≥1 infusion-related AE. No treatment-emergent AEs were severe or serious nor led to withdrawal. The mean PedMIDAS score decreased from 52.7 (severe) at baseline to 25.1 (mild) at Week 12.

**Conclusion:** This robust and reliable popPK model for eptinezumab in children and adolescents showed appropriate weight-based dose levels and support the weight-based dosing approach in pediatric efficacy studies. Eptinezumab was generally well tolerated, with no new safety signals observed.

## P005 A prokinetic effect of corydalis tuber and pahrbitidis semen extract (DA 9701) improves nausea and headache in migraine neurological disorders: a real world observational study

### M. Kim^1^, S. H. Lee^2^, H. Son^1^, M. J. Lee^1^

#### ^1^Seoul National University Hospital, Neurology, Seoul, South Korea; ^2^Jeonbuk University College of Medicine, Jeonju, South Korea

##### **Correspondence:** M. Kim

*The Journal of Headache and Pain 2024,* **25(Suppl 1)**: P005

**Objective:** Nausea is frequent in migraineurs. D2 antagonist had been used to symptomatic control but adverse event such as exrapyramidal signs are occacionally encountered. DA 9701 is known extract of Corydalis tuber and Pahrbitidis semen. In gastrointestinal system, and proven efficacy and safety are known as D2 antagonist and agonism of 5-HT1A, Adrenergic α2, and 5-HT4.In this study, we attempted to apply DA 9701 on migraine with nausea, and assess the safety and efficacy on headache frequency with nausea improvement.

**Methods:** 110 subjects (102 women, 8 men) with migraineurs with nausea were asked to take DA9701 90 mg divided three times a day for 4 weeks, from March 2021 through February 2022. The primary end point was a nausea frequency change following DA9701 at week 4 from baseline. Headache days and acute rescue medicine frequency change was the secondary outcome. These parameters were evaluated using Headache diaries. Safeties were performed by questionarre from DIEPS (Drug-induced EPS) scale. Subgroups (epidosodic migraine (EM) vs chronic migraine (CM)) were further explored (58 EM, 52 CM).

**Results:** The primary end point (nausea frequency change) showed a decrease of 53.7% (4.65 days) (*p* <0.001) ; Headache and acute rescue medicine frequency change as secondary end points, was 52.2% (8.18 days) decrease (*p* <0.001) and 44.8% (3.46 days) decrease (*p* <0.001), respectfully. In the subgroup analysis in EM and CM, whereas nausea changed 2.21 (*p* <0.001) and headache days decrease 2.89 (*p* <0.001) in EM, in CM, 7.12 (*p* <0.001) for nausea and 13.47 (*p* <0.001) days for headache days decrease observed. No side effect including extrapyramidal symptoms (EPS) were reported.

**Conclusion:** DA9701 can reduce the nausea effectively, and additional effect on migraine headache in CM without nausea, suggesting the independent prophylactic effect of DA9701 on migraine headache disorders.

Randomized controlled trial would be warranted.

## P006 Triptan failure in specialized headache care: cross-sectional data from the DMKG Headache Registry

### R. Ruscheweyh^1,2^, G. Gossrau^3^, V. Ruschil^4^, C. Gaul^5^, T. Kraya^6^, J. Scheidt^7^, T. P. Jürgens^8^

#### ^1^LMU Hospital, LMU Munich, Neurology, Munich, Germany; ^2^German Migraine and Headache Society, Frankfurt a. M., Germany; ^3^Universitätsklinikum Dresden, Universitätsschmerzcentrum, Dresden, Germany; ^4^Universitätsklinikum Tübingen, Neurologische Klinik, Tübingen, Germany; ^5^Headache Center Frankfurt, Frankfurt a. M., Germany; ^6^Krankenhaus Sankt Georg Leipzig, Leipzig, Germany; ^7^Hochschule Hof, Institute of information systems, Hof, Germany; ^8^KMG Krankenhaus Güstrow, Neurologische Klinik, Güstrow, Germany

##### **Correspondence:** R. Ruscheweyh

*The Journal of Headache and Pain 2024,* **25(Suppl 1)**: P006

**Objective:** Triptans are effective for many migraine patients, but some do not experience adequate efficacy and tolerability, or have contraindications. The European Headache Federation (EHF) has proposed that patients with lack of efficacy and/or tolerability of ≥2 triptans ("triptan resistance") could be considered eligible for treatment with the novel, effective but more expensive medications from the ditan and gepant groups. There is little evidence on how frequent triptan resistance is.

**Methods:** We applied the EHF criteria for triptan response and triptan (efficacy and/or tolerability) failure to data from the German Migraine and Headache Society (DMKG) Headache Registry.

**Results:** 2284 adult migraine patients (females: 85.4%, age: 39.4 ± 12.8 years) were included. 42.5% (*n* = 970) had failed ≥1 triptan, 13.1% (*n* = 300) had failed ≥2 triptans ("triptan resistant"), and 3.9% (*n* = 88) had failed ≥3 triptans (Fig. 1). Compared to triptan responders (current use, no previous failure, *n* = 597), triptan non-responders had significantly more severe migraine (higher frequency and disability, *p* < 0.001 and higher intensity, *p* < 0.05), that further increased with the level of triptan failure (Fig. 2). Highest proportions of responders were found for nasal and oral zolmitriptan, oral eletriptan, and subcutaneous sumatriptan.

**Conclusion:** In the present specialized headache care setting, a significant number of patients (13.1%) were classified as "triptan resistant" according to the EHF criteria. Triptan resistance was associated with significantly higher migraine severity and migraine-related disability, emphasizing the importance of establishing an effective and tolerable acute (and preventive) migraine medication. Acute migraine treatment optimization might include evaluation of one of the triptans with the highest responder rates (nasal and oral zolmitriptan, oral eletriptan, and subcutaneous sumatriptan) and/or switching to a different acute medication class.

**Fig. 1 (Abstract P006):**
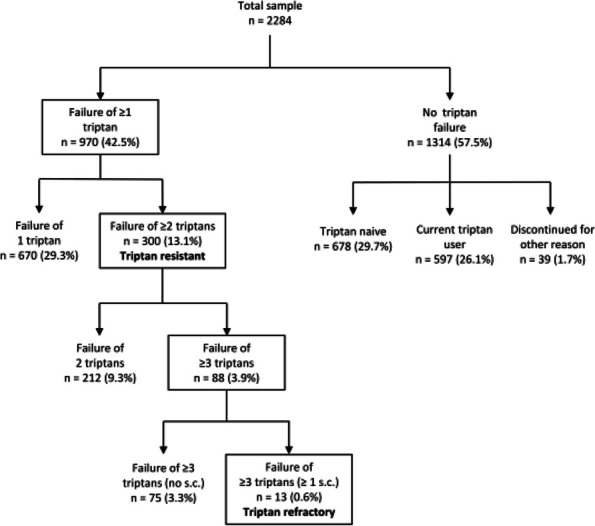
See text for description

**Fig. 2 (Abstract P006):**
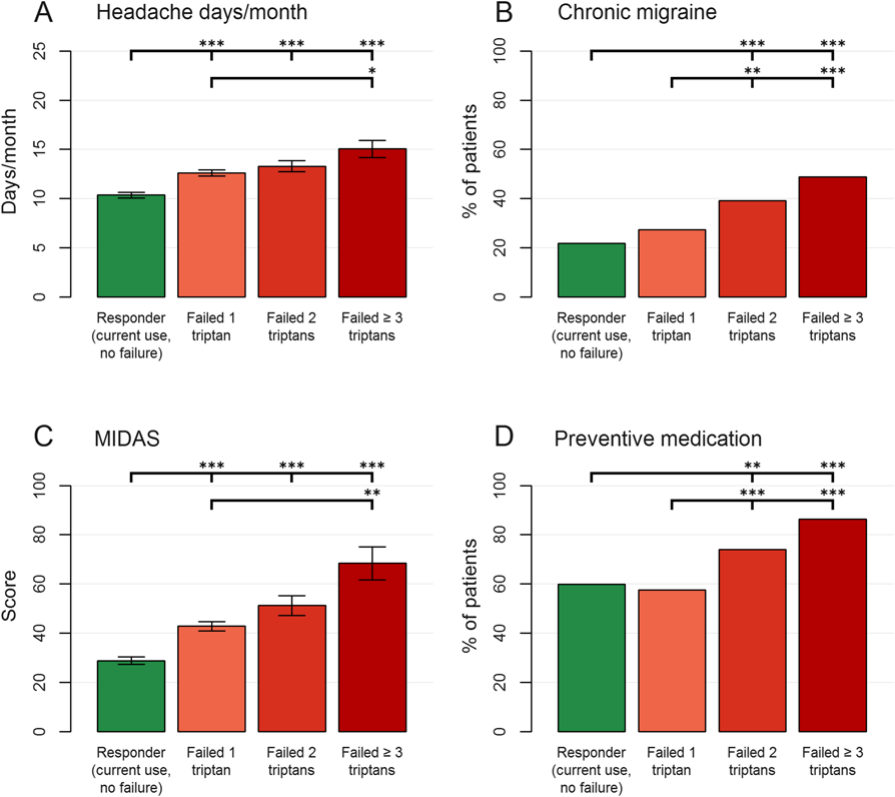
See text for description

## P007 Reduction in barbiturate prescription fills and quantity dispensed following initiation of rimegepant treatment: a real-world administrative claims study

### N. Rosen^1^, A. Mohajer^2^, L. Abraham^3^, J. Brown^4^, K. Hygge Blakeman^5^, A. Jenkins^3^, L. Harris^6^, G. L'Italien^6^

#### ^1^Northwell Health Institute for Neurology and Neurosurgery, Headache Center, Great Neck, NY, United States; ^2^Qral Group, New Orleans, LA, United States; ^3^Pfizer R&D UK Ltd., Tadworth, United Kingdom; ^4^Pfizer, Inc., New York, NY, United States; ^5^Pfizer AB, Stockholm, Sweden; ^6^Biohaven Pharmaceuticals Inc., New Haven, CT, United States

##### **Correspondence:** L. Abraham

*The Journal of Headache and Pain 2024,* **25(Suppl 1)**: P007

**Objective:** This study evaluated real-world changes in barbiturate prescribing patterns and usage after initiation of migraine therapy with rimegepant, a CGRP antagonist, as measured by monthly quantity dispensed, monthly prescriptions filled, and discontinuation rate.

**Methods:** Prescription claims data from 9/15/2019 through 11/30/2022 were collected from a longitudinal pharmacy commercial claims database for 689,425 migraine patients treated with rimegepant. To determine study eligibility, we required at least 12 months of total time in the dataset, at least one barbiturate fill in a 6-month baseline period prior to rimegepant initiation, and at least 2 rimegepant fills during a 6-month follow-up. Monthly barbiturate prescription fills and barbiturate milligrams dispensed were tabulated in the baseline and follow-up periods. Discontinuation was defined as the absence of a barbiturate prescription fill after rimegepant initiation. The study was repeated with similar endpoints over 6 and 18 months of total observation and according to triptan use status.

**Results:** We observed 34,486 migraine patients who initiated treatment with rimegepant and used butalbital during the 6 months prior to rimegepant initiation (age 46.8±12.6 years; 89.2% female). A total of 288,053 patients had 2+ rimegepant fills and met time-in-data requirements, but had no baseline butalbital Rx fill, so the study cohort represents a 10.7% period-prevalence of barbiturate exposure in the 6-month period immediately preceding rimegepant initiatio. A 26.7% decrease in mean monthly butalbital milligrams dispensed and a 32.0% decrease in total butalbital Rx fills was observed in the study cohort after rimegepant initiation. A barbiturate discontinuation rate of 49.4% was observed after rimegepant initiation. These effects were similar in direction and magnitude for patients with or without triptan exposure over observation periods of 6 and 18 months.

**Conclusion:** In migraine patients who use barbiturates, a significant decrease in barbiturate use, measured by mean monthly butalbital Rx fills, mean monthly butalbital milligrams dispensed, and butalbital discontinuation, was observed in the months following initiation of rimegepant therapy.

**Fig. 1 (Abstract P007):**
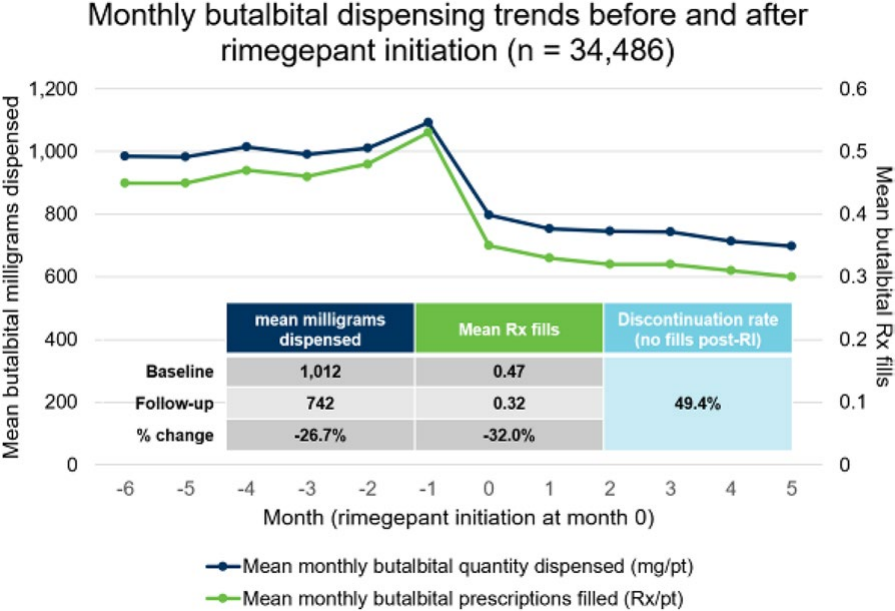
See text for description

**Fig. 2 (Abstract P007):**
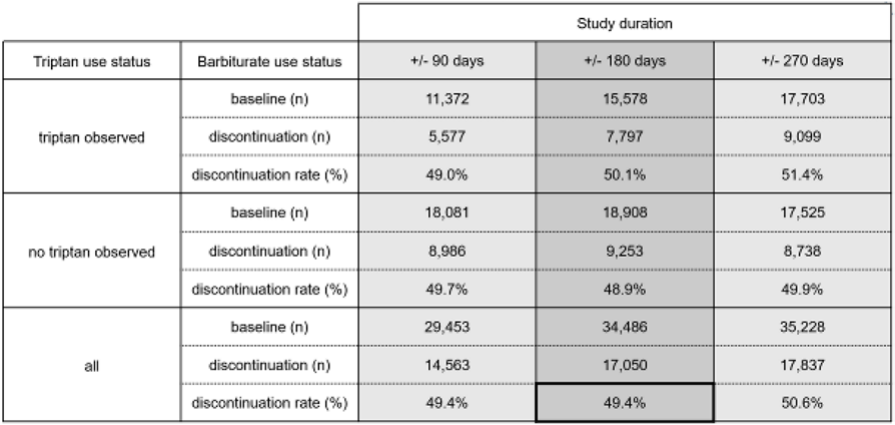
See text for description

**Fig. 3 (Abstract P007):**
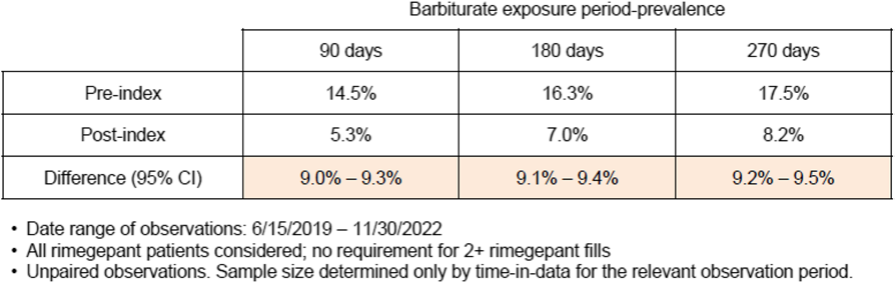
See text for description

## P008 BACT – Botulinum toxin A in frequent and chronic tension-type headache with the FollowTheSutures paradigm. A triple blind, randomized, placebo-controlled cross-over trial

### K. Devik

#### Norwegian University of Science and Techologu, Namsos, Norway

*The Journal of Headache and Pain 2024,* **25(Suppl 1)**: P008

**Objective:** The primary aim is to determine the efficacy of treatment with botulinum toxin with a FollowTheSutures injection-regime in frequent and chronic tension type headache (TTH).

Secondary aims are the assessment of safety, difference in number of responders, in headache severity, in duration of headache, in use of pain medication and in quality-of-life measures.

**Methods:** Randomized, placebo-controlled, triple blind, cross-over trial.

The primary efficacy variable is the difference in monthly headache days in the active period versus the placebo period.

The target population is male and female patients 18 to 75 years of age with TTH, with 10 or more headache days per month. They will be preferably recruited from the outpatient clinics at the Department of Neurology at Namsos Hospital, but also from all of Norway through advertisement in social media and referrals from other treatment centres A total of 66 patients will be included.

In this study, injections will follow a FollowTheSutures injection protocol. Study duration is 36 weeks long. A 4 weeks screening/baseline period with a daily electronic headache diary (eDiary) follows inclusion and informed consent. After completing the 4-week screening/baseline period, participants will be re-screened and eligible participants will enter the randomized phase of the study consisting of two treatment periods. The treatment periods are either injections with verum (onabotulinum toxin suspended in saline (Botox® and NaCl 0,9% Braun) or placebo (only NaCl 0,9% Braun). The participants are randomized to the order with which they enter the two periods, and a triple-blinding regime is implemented. Each treatment period lasts 12 weeks, separated by a 4 week washout period. Participants continue to keep a daily eDiary and register adverse events in the entire randomized period.

**Results:** Trial status: study has started 03.10.23 and alleredy inrolled 51 patients and 39 of them are randomized.

**Conclusion:** Study finishes in medio 2024.

## P009 OnabotulinumtoxinA add-on to monoclonal anti-CGRP antibodies in treatment-refractory chronic migraine

### E. Dermitzakis^1^, A. Argyriou^2^, G. Xiromerisiou^3^, M. Vikelis^4^

#### ^1^Euromedica General Clinic, Thessaloniki, Greece; ^2^“Agios Andreas” State General Hospital of Patras, Neurology Department, Patras, Greece; ^3^Medical School, University of Thessaly, Larisa, Greece; ^4^Mediterraneo Hospital, Headache Clinic, Athens, Greece

##### **Correspondence:** A. Argyriou

*The Journal of Headache and Pain 2024,* **25(Suppl 1)**: P009

**Objective:** We sought to assess the effectiveness of combining dual therapy with ΟnabotulinumtoxinA (BTX) add-on to anti-calcitonin gene-related peptide (CGRP) monoclonal antibodies (anti-CGRP MAbs) in treatment-refractory patients with chronic migraine (CM), who failed to respond to all available monotherapies

**Methods:** We retrospectively reviewed the medical files of 19 treatment-refractory patients with CM who failed to two oral preventatives; at least three consecutive BTX cycles (less than 30% response rate); at least three consecutive sessions with either fremanezumab or erenumab (less than 30% response rate) and were eventually switched to dual therapy with BTX add-on to any of the already-given anti-CGRP MAb. We then assessed from baseline to each monotherapy or dual intervention efficacy pre-defined follow-up any occurring changes in the following efficacy outcomes: (i) monthly headache days (MHD) (ii) monthly days with moderate/severe peak headache intensity (iii) monthly days with intake of any acute headache medication. Response (50% reduction in MHD) rates as also safety-tolerability were also determined.

**Results:** In most cases (*n*=14), dual targeting proved effective and was associated with clinically meaningful improvement in all efficacy variables; 50% response rates as also disability and QOL outcomes, coupled with favorable safety/tolerability.

**Conclusion:** Our results advocate in favor of the view that dual therapy is indeed effective and should be considered in difficult-to-treat CM patients who failed all available monotherapies.

## P010 Predictors of response to fremanezumab in migraine patients with at least three previous preventive failures: post-hoc analysis of a prospective, multicenter, real-world Greek registry

### A. Argyriou^1^, E. Dermitzakis^2^, G. Xiromerisiou^3^, D. Rallis^4^, P. Soldatos^5^, P. Litsardopoulos^1^, M. Vikelis^6^

#### ^1^“Agios Andreas” State General Hospital of Patras, Neurology Department, Patras, Greece; ^2^Euromedica General Clinic, Thessaloniki, Greece; ^3^Medical School, University of Thessaly, Larisa, Greece; ^4^Tzaneio General Hospital of Piraeus, Department of Neurology, Athens, Greece; ^5^Private Praxis, Kalamata, Greece; ^6^Mediterraneo Hospital, Headache Clinic, Athens, Greece

##### **Correspondence:** A. Argyriou

*The Journal of Headache and Pain 2024,* **25(Suppl 1)**: P010

**Objective:** To define in a real-world population of patients with high-frequency episodic (HFEM) or chronic migraine (CM) the predictive role of socio-demographic or phenotypic profiling of responders to fremanezumab.

**Methods:** Two-hundred and four adult fremanezumab-treated patients with either HFEM or CM, who failed to at least 3 preventives, provided data at baseline on several individual socio-demographic and phenotypic variables. These variables were analyzed for their ability to independently predict the response (50-74% response rates) or super-response (≥ 75% response rates) to fremanezumab. Patients were followed from 3-18 months of fremanezumab exposure

**Results:** The main finding to emerge from univariate analyses was that threebaseline socio-demographic/clinical variables, i.e., age group 41-70 years (*p*=0.02); female gender (*p*=0.03); patients with HFEM (*p*=0.001) and three clinical phenotypic variables, i.e., strict unilateral pain (*p*=0.05); pain in the ophthalmic trigeminal branch (*p*=0.04) and the "imploding" quality of pain (*p*=0.05), were significantly related to fremanezumab response. However, in multivariate analysis, only HFEM (*p*=0.02); the presence of strict unilateral (*p*=0.03); and pain location in the ophthalmic trigeminal branch (*p*=0.036) were independently associated with good fremanezumab response. Allodynia (*p*=0.04) was the only clinical predictive variable of super-responsiveness to fremanezumab.

**Conclusion:** A precise phenotypic profiling with identification of pain characteristics consistent with peripheral and/or central sensitization might reliably predict the responsiveness to fremanezumab in migraine prophylaxis.

## P011 Efficacy and safety of fremanezumab for migraine prophylaxis in patients with at least three previous preventive failures: prospective, multicenter, real-world data from a Greek registry

### A. Argyriou^1^, M. Vikelis^2^, G. Xiromerisiou^3^, P. Soldatos^4^, D. Rallis^5^, P. Litsardopoulos^1^, E. Dermitzakis^6^

#### ^1^“Agios Andreas” State General Hospital of Patras, Neurology Department, Patras, Greece; ^2^Mediterraneo Hospital, Headache Clinic, Athens, Greece; ^3^Medical School, University of Thessaly, Larisa, Greece; ^4^Private Praxis, Kalamata, Greece; ^5^Tzaneio General Hospital of Piraeus, Department of Neurology, Athens, Greece; ^6^Euromedica General Clinic, Thessaloniki, Greece

##### **Correspondence:** A. Argyriou

*The Journal of Headache and Pain 2024,* **25(Suppl 1)**: P011

**Objective:** To prospectively assess the efficacy and safety of fremanezumab for migraine prophylaxis in patients with failure of at least three previous preventive treatments. Changes in disability as also quality-of-life outcomes after fremanezumab treatment were also examined.

**Methods:** Two hundred and four patients with either high frequency EM (HFEM) or chronic migraine (CM), who attained at least three consecutive monthly sessions with fremanezumab 225mg and otherwise met the inclusion criteria, were included in the study. The crude response (at least 50% reduction in monthly headache days [MHD]) rates to fremanezumab were assessed. Scores in the following efficacy outcomes were then compared from baseline to the last efficacy evaluation follow-up: (i) MHD (ii) monthly days with moderate/severe peak headache intensity (iii) monthly days with intake of abortive medication. The disability was evaluated with the "Migraine Disability Assessment"; the quality of life (QOL) status was assessed with the "Headache Impact-6 Test" and the "EQ-5D questionnaire".

**Results:** In the majority of HFEM cases (*n*=81/97; 83.5%) and CM patients (*n*=67/107; 62.6%), fremanezumab proved effective in reducing the MHDs by at least 50% and was associated with clinically meaningful improvement in all other efficacy variables. The migraine-related disability experienced by our patients decreased and their QOL increased. We recorded just 36 cases reporting mild adverse events, including pain, rush or pruritus (*n*=26), flue-like symptoms (*n*=8) and hair loss (*n*=2).

**Conclusion:** With our prospective results, we further provide real-world data to support the favourable benefit/risk profile of fremanezumab in the prophylaxis of both HFEM and CM.

## P012 Effects of fremanezumab on psychiatric comorbidities in difficult-to-treat patients with chronic migraine: post-hoc analysis of a prospective, multicenter, real-world Greek registry

### M. Vikelis^1^, A. Argyriou^2^, G. Xiromerisiou^3^, D. Rallis^4^, P. Soldatos^5^, D. Rikos^6^, P. Litsardopoulos^2^, E. Dermitzakis^7^

#### ^1^Mediterraneo Hospital, Headache Clinic, Athens, Greece; ^2^“Agios Andreas” State General Hospital of Patras, Neurology Department, Patras, Greece; ^3^Medical School, University of Thessaly, Larisa, Greece; ^4^Tzaneio General Hospital of Piraeus, Department of Neurology, Athens, Greece; ^5^Private Praxis, Kalamata, Greece; ^6^404 Greek Military Hospital, Department of Neurology, Larisa, Greece; ^7^Euromedica General Clinic, Thessaloniki, Greece

##### **Correspondence:** A. Argyriou

*The Journal of Headache and Pain 2024,* **25(Suppl 1)**: P012

**Objective:** This post hoc analysis aimed to evaluate the efficacy of fremanezumab in difficult-to-treat chronic migraine (CM) patients with and without psychiatric comorbidities (PCs); mainly anxiety and/or depression.

**Methods:** We assessed data from CM patients with and without PCs, who failed to at least 3 preventives and eventually attained at least three consecutive monthly sessions with fremanezumab 225mg. Outcomes included the crude response (≥50% reduction in monthly headache days [MHD]) rates to fremanezumab from baseline to the last clinical follow-up. The changes in MHD; MHD of moderate/greater severity; monthly days with intake of abortive medication and the proportion of patients" changing status from with to decreased/without PCs were also compared. Disability and quality of life (QOL) outcomes were also assessed.

**Results:** Of 107 patients enrolled, 65 (60.7%) had baseline PCs. The percentage of patients with (*n*=38/65; 58.5%) and without PCs (*n*=28/42; 66.6%) that achieved a ≥50% reduction in MHD with fremanezumab was comparable (*p*=0.41), whereas MHDs were significantly reduced (difference vs baseline) in both patients with PCs (mean –8.9 [standard error: 6.8]; *p*<0.001) and without PCs (–9.8 [7.5]; *p*<0.001). Both groups experienced significant improvements in all other efficacy, disability and QOL outcomes at comparable rates, including in MHD reduction. A significant proportion of fremanezumab-treated patients with baseline PCs de-escalated in corresponding severities or even reverted to no PCs (28/65; 43.1%), post-fremanezumab.

**Conclusion:** Fremanezumab appears effective as preventive treatment in difficult-to treat CM patients with and without PCs, while also being beneficial in reducing the severity of comorbid anxiety and/or depression.

## P013 Real-world effectiveness of Anti-CGRP monoclonal antibodies compared to OnabotulinumtoxinA. The RAMO study: early results

### D. A. Montisano^1^, R. Giossi^1^, M. Canella^1^, F. Vernieri^2^, C. Altamura^2^, L. Grazzi^1^

#### ^1^Foundation IRCCS C. Besta Institute, Milano, Italy; ^2^Campus Bio-Medico University, Rome, Italy

##### **Correspondence:** D. A. Montisano, L. Grazzi

*The Journal of Headache and Pain 2024,* **25(Suppl 1)**: P013

**Objective:** Aim of this study is to compare the effectiveness and safety of anti-CGRP mAbs and BoNT-A after 6 and 12 months of treatment.

**Methods:** We enrolled patients from IRCCS Neurologic Institute C. Besta and Bio-Medic Campus University. Inclusions criteria:diagnosis of CM,received anti-CGRP mAbs or BoNT-A,with at least 6 months follow-up,age 18-65y,≥2 preventive treatment failures, starting MIDAS ≥11. Exclusion criteria:serious psychiatric diseases, received BoNT-A before anti-CGRP mAbs treatment (for mAbs arm). Study outcomes: difference from baseline in monthly migraine days (MHD), number of monthly acute medications (MAM) and MIDAS. Safety assessment:report of serious adverse events (SAE), treatment discontinuation. Wilcoxon rank-sum and Fisher"s exact tests were used for the analyses(*p*<0.05).

**Results:** At the time of this interim analysis, we screened 122 patients:92 included,25 mAbs arm,67 BoNT-A arm. Population:mean age 51.1(8.6)y,80 female,90 medication overuse (non-significant differences between groups). At baseline the BoNT-A group presented significantly higher mean MHD(23.0[6.3]vs17.4[32.2]), MAM(24.1[13.7]vs16.5 [2.8]), and MIDAS(93.0[66.8]vs55.6[36.8]) compared to mAbs group. MHD reduction was significantly greater in the mAbs group (-12.4[4.8] vs -9.0[8.8]) at 6 months compared to BoNT-A. Adverse events"(AE) discontinuation: 4.8% patients mAbs arm, 4.5% patients BoNT-A arm.

**Conclusion:** Our preliminary results show a comparable effectiveness between mAbs and BoNT-A at 12months follow-up, with significantly higher efficacy for mAbs at 6 months. Discontinuation due to AE were similar. From these preliminary data the effectiveness and sustainability of the two treatments appear to be overlapping

## P014 Randomized controlled studies evaluating Topiramate, Botulinum toxin type A, and mABs targeting CGRP in patients with chronic migraine and medication overuse headache: a systematic review and meta-analysis

### S. Giri^1,2^, E. Tronvik^1,2,3^, M. Linde^2,3^, S. A. Pedersen^4^, K. Hagen^1,2,5^

#### ^1^Norwegian University of Science and Technology, Department of Neuromedicine and Movement Science (INB), Trondheim, Norway; ^2^NorHEAD-Norwegian centre for Headache Research, Trondheim, Norway; ^3^Norwegian Advisory Unit on Headache , St. Olavs Hospital, Department of Neurology and Clinical Neurophysiology, Trondheim, Norway; ^4^The Medicine and Health Library, NTNU, Library Section for Research Support, Data and Analysis, Trondheim, Norway; ^5^Clinical Research Unit Central Norway, St. Olavs University Hospital, Trondheim, Norway

##### **Correspondence:** S. Giri

*The Journal of Headache and Pain 2024,* **25(Suppl 1)**: P014

**Objective:** To describe and evaluate the relative effects of preventive treatment of topiramate, botulinum toxin type A (BoNTA), and calcitonin gene-related peptide (CGRP) in chronic migraine patients with medication overuse headache (MOH).

**Methods:** A systematic search was conducted in the databases CENTRAL, MEDLINE, Embase and Web of Science until May 2022. We included randomized controlled trials reporting the outcomes of change in monthly headache/migraine days, ≥50% response rates and change in medication overuse status. Studies were excluded if response rates were not reported. Risk of bias assessment was performed using the Cochrane RoB2 tool. The quality of evidence for outcomes across included studies were evaluated according to the five factors outlined in Cochrane GRADE approach.

**Results:** The initial search resulted in 1599 records. Ten studies met our inclusion criteria, while seven studies with sufficient data were included in the meta-analysis. Studies assessing BoNTA included 1139 patients and showed a mean reduction in headache frequency by 1.92 days per month compared to placebo (-1.92; 95% CI -2.68 to -1.16). Studies assessing human monoclonal antibodies (mABs) included 1982 patients, and showed significant positive effects compared to placebo for all measured outcomes. The overall odds ratio for the ≥50% response rate was 2.90 (95% CI, 2.23 to 3.78). No significant difference was observed in the frequency of adverse effect for both BoNTA and low dose of mABs compared to placebo. There is currently insufficient evidence to determine the impact of topiramate in chronic migraine patients with MOH.

**Conclusion:** BoNTA and mABs targeting CGRP(r) were beneficial in reducing monthly migraine days and ≥50% response rate, but uncertainties remained for BoNTA regarding response rate. The effect size for mABs was greater with relatively lower drop-out rate. High-quality randomized trials are required to evaluate the effect of topiramate in chronic migraine patients with MOH.

**Fig. 1 (Abstract P014):**
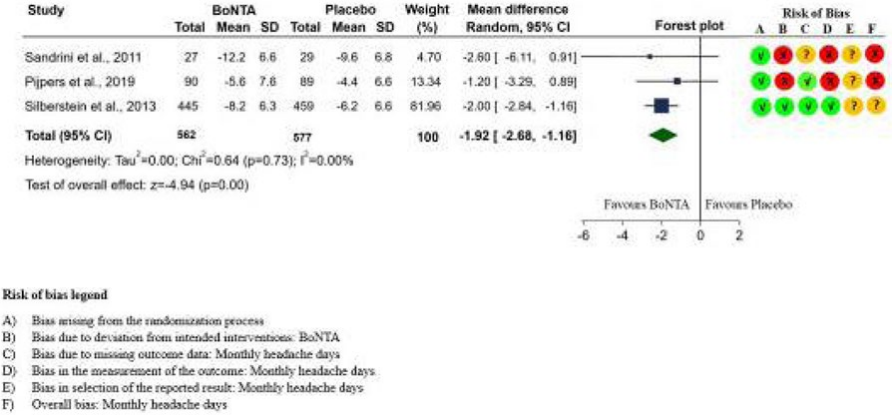
See text for description

**Fig. 2 (Abstract P014):**
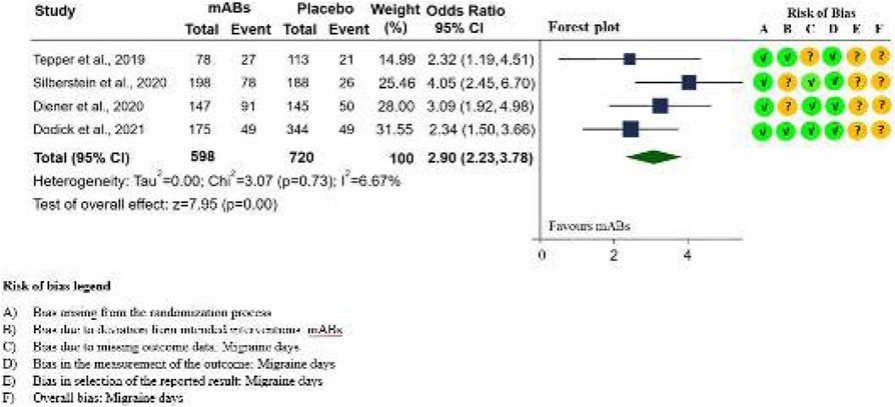
See text for description

## P015 Real-world evidence for frequency of constipation with the use of anti-CGRP monoclonal antibodies in chronic migraine

### S. Uzun^1,2^, U. Frejvall^1^, G. Özkaya Sahin^3,4^, G. Sahin^1,2^

#### ^1^Skåneuro Neurology Clinic, Lund, Sweden; ^2^Lund University, Department of Clinical Sciences of Malmö and Lund, Lund, Sweden; ^3^Lund University, Department of Laboratory Medicine, Lund, Sweden; ^4^Lund University, Department of Translational Medicine, Lund, Sweden

##### **Correspondence:** S. Uzun

*The Journal of Headache and Pain 2024,* **25(Suppl 1)**: P015

**Objective:** Constipation is increasingly being reported as a common side effect of anti-calcitonin gene related peptide (CGRP) monoclonal antibodies (mAbs). This can be attributed to the role of CGRP in peristalsis and regulating ions and water secretion in the bowels. Our study aims to analyze the differential frequency of constipation among anti-CGRP mAbs.

**Methods:** We included chronic migraine patients treated with anti-CGRP mAbs between 1 Jan 2019 and 31 Dec 2022 at SkåNeuro neurology clinic. Charts that include demographic data, migraine features, medical history, and information regarding constipation were reviewed. The severity of treatment-induced/worsened constipation was categorized on a scale of 0 to 3, with 3 is leading to treatment discontinuation.

**Results:** A total of 345 patients were included, 212 receiving erenumab, 100 receiving fremanezumab, and 33 receiving galcanezumab as their initial anti-CGRP mAbs. The constipation rates were 51.4% for erenumab, 3.3% for fremanezumab, and 12.1% for galcanezumab. Among patients treated with erenumab, 24.5% switched to another anti-CGRP mAbs because of constipation, 21.2 % due to lack and/or disappearance of effect. Patients who switched treatments experienced significantly milder symptoms (*p*<0.0001). Even those who switched due to lack and/or disappearance of effect reported an improvement in their constipation (*p*=0.011). These rates were extremely low for fremanezumab and galcanezumab.

**Conclusion:** This analyzes shows that erenumab has much higher constipation frequency than reported rates from clinical trials and compared to fremanezumab and galcanezumab. This differing side effect profiles could be explained by mechanism of actions i.e., receptor vs ligand. Clinicians should take constipation into account while selecting treatment and try to switch to another anti-CGRP mAbs if severe constipation occurs.

## P016 Onabotulinum Toxin A (OnA) as a preventive treatment for non-migraine headaches: a retrospective analysis

### C. Nieves Castellanos, M. A. Olivier, L. Ferré González, S. Díaz Insa

#### Hospital Universitari i Politécnic la Fe, Neurology, Valencia, Spain

##### **Correspondence:** C. Nieves Castellanos

*The Journal of Headache and Pain 2024,* **25(Suppl 1)**: P016

**Objective:** Non-migraine headaches often pose challenges in terms of finding effective preventive treatments. As a result, off-label use of onabotulinum toxin A (OnA) has emerged as a therapeutic option. In this study, we aimed to analyze the outcomes of patients with non-migraine headaches who were treated with OnA at our headache unit.

**Methods:** We conducted a retrospective observational study to analyze patients treated with OnA over the past 6 months. The study focused on various factors: type of headache, previous/current treatments, duration of OnA therapy, dosage, injection sites, efficacy, and side effects.

**Results:** We collected 84 patients, 57 patients with primary headaches and 27 with secondary headaches. An average of 10,8 years from headache onset and 37.7 months of treatment with OnA. Patients had failed 4 previous preventive treatments.

The diagnosis and the average doses of OnA were: 31 patients with trigeminal neuralgia (85 UI), 14 with Persistent idiopathic facial pain (124UI), 7 with cluster headache (229UI), 7 with occipital neuralgia (69 UI), 6 with nummular headache (47UI), 5 with persistent headache attributed to craniotomy (110UI), 4 with tension type headache (135UI) and 10 with other headaches (157UI).

Most of injection strategies (84% of patients) followed a "following the pain" approach. In 59.5% of patients, the injections were performed unilaterally, while only 15.5% underwent infiltration using the amplified PREEMPT protocol.

11 (13%) reported mild efficacy, 33 (39%) medium efficacy, and 38 (45.3%) reported high efficacy. Eight patients experienced mild aesthetic side effects, while no other side effects were reported.

**Conclusion:** Onabotulinum toxin A (OnA), originally approved for chronic migraine, demonstrates potential efficacy and minimal side effects in various headaches and neuralgias. Early consideration of OnA usage, particularly in trigeminal neuralgia and persistent idiopathic facial pain, may help address unmet needs in pain management.

## P017 Real-World effectiveness and safety of fremanezumab in migraine: 3rd interim analysis of the pan-european PEARL study

### M. Ashina^1,2^, D. D. Mitsikostas^3^, F. Mohammad Amin^1,4^, P. Kokturk^5^, C. J. Schankin^6^, G. Sahin^7^, P. Pozo-Rosich^8^, P. Dorman^9^, T. Nežádal^10^, A. Christine Poole^11^, I. Pavão Martins^12^, M. L. Sumelahti^13^, V. Ramirez Campos^14^, X. Ning^14^, L. Lyras^5^, C. Tassorelli^15,16^

#### ^1^Danish Headache Center, Copenhagen University Hospital – Rigshospitalet Glostrup, Department of Neurology, Copenhagen, Denmark; ^2^University of Copenhagen, Department of Clinical Medicine, Copenhagen, Denmark; ^3^National and Kapodistrian University of Athens, Department of First Neurology, Aeginition Hospital, Athens, Greece; ^4^Rigshospitalet Glostrup, University of Copenhagen, Department of Neurorehabilitation/Traumatic Brain Injury, Copenhagen, Denmark; ^5^Teva Netherlands B.V., Amsterdam, Netherlands; ^6^Inselspital, University Hospital Bern, University of Bern, Department of Neurology, Bern, Switzerland; ^7^Lund University, Department of Clinical Sciences of Lund, Lund, Sweden; ^8^Vall d’Hebron Hospital & Research Institute, Universitat Autonoma de Barcelona, Headache Unit & Research Group, Barcelona, Spain; ^9^The Newcastle upon Tyne Hospitals NHS Foundation Trust, Newcastle upon Tyne, United Kingdom; ^10^Charles University, Institute of Neuropsychiatric Care, 1st School of Medicine, Prague, Czech Republic; ^11^Private Practice: Oslo Headache Centre, Oslo, Norway; ^12^University of Lisbon, Centro de Estudos Egas Moniz, Faculty of Medicine, Lisbon, Portugal; ^13^University of Tampere, Faculty of Medicine and Health Technology, Tampere, Finland; ^14^Teva Branded Pharmaceutical Products R&D, Inc., West Chester, PA, United States; ^15^University of Pavia, Department of Brain and Behavioral Sciences, Pavia, Italy; ^16^IRCCS Mondino Foundation, Pavia, Italy

##### **Correspondence:** M. Ashina

*The Journal of Headache and Pain 2024,* **25(Suppl 1)**: P017

**Objective:** PEARL (EUPAS35111) is an observational, prospective, Phase 4 study of fremanezumab effectiveness and safety for episodic and chronic migraine (EM, CM) prevention. This 3rd interim analysis was conducted when all enrolled patients had completed ≥6 months of treatment.

**Methods:** Participants are adults with EM or CM receiving fremanezumab treatment for migraine prevention, who maintain a daily headache diary prior to and throughout the 24-month observational period. Primary endpoint: proportion of patients with ≥50% reduction in monthly migraine days (MMD) during the 6 months post-fremanezumab initiation. Secondary endpoints: mean change from baseline across Months 1–12 in MMD, days of acute migraine medication use, and headache-related disability (MIDAS and HIT-6). Safety was measured through adverse events (AEs) reported in clinical practice.

**Results:** Of 1140 patients enrolled in PEARL, 968 were included in the effectiveness analysis (EM, 33.1%; CM, 66.9%). In patients with available data, 428/732 (58.5%) achieved ≥50% MMD reduction during the 6 months post-fremanezumab initiation (Figure 1). The proportions of patients reaching ≥50% reduction in MMD were sustained at Months 1–12 (Figure 2), as were reductions in mean MMD (Figure 3), days of acute medication use and headache-related disability. Overall, 267/1140 (23.4%) patients reported AEs relating to treatment, 2/1140 (0.2%) reported serious AEs relating to treatment and 33/1140 (2.9%) reported AEs that led to discontinuation.

**Conclusion:** Over half of patients achieved ≥50% MMD reduction during the 6 months post-fremanezumab initiation, with sustained reductions in acute medication use and disability observed over 12 months, and few AEs leading to discontinuation reported. Outcomes from this large, real-world population can guide informed migraine management.

**Fig. 1 (Abstract P017):**
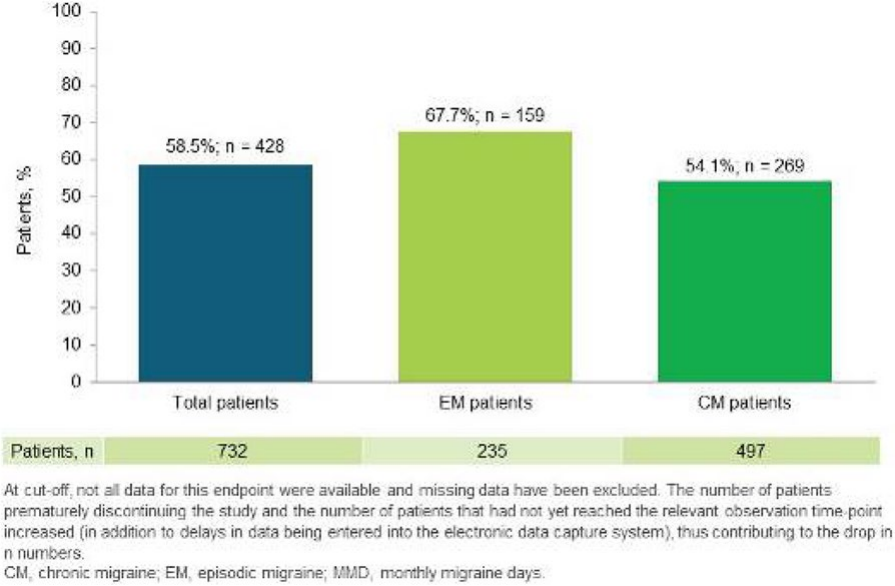
Proportion of patients reaching ≥50% reduction in MMD from baseline by migraine type during the 6 months post-fremanezumab initiation (primary endpoint)

**Fig. 2 (Abstract P017):**
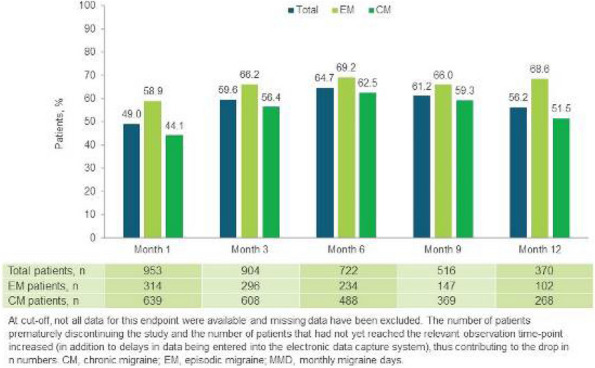
Proportion of patients reaching ≥50% reduction in MMD from baseline by migraine type at months 1, 3, 6, 9, and 12

**Fig. 3 (Abstract P017):**
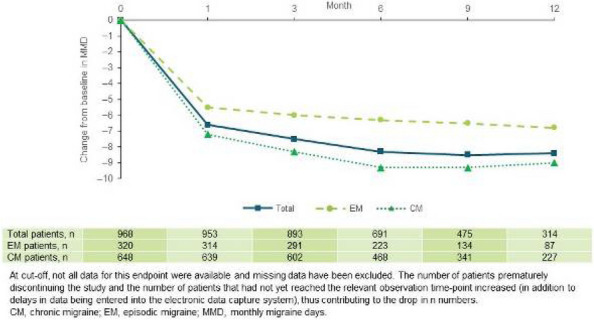
Mean change from baseline in MMD at months 1, 3, 6, 9, and 12 after fremanezumab treatment initiation, by migraine type

## P018 Primary bilateral trigeminal neuralgia: controlling double trouble pain with onabotulinumtoxinA

### G. Cabral^1^, R. Pelejão^1^, M. Viana Baptista^1,2^

#### ^1^Hospital Egas Moniz, Neurology, Lisbon, Portugal; ^2^NOVA Medical School, Universidade NOVA de Lisboa,, Chronic Diseases Research Center (CEDOC), Lisbon, Portugal

##### **Correspondence:** G. Cabral

*The Journal of Headache and Pain 2024,* **25(Suppl 1)**: P018

**Objective:** Bilateral trigeminal neuralgia (BTN) is an uncommon finding, occurring in up to 6% of patients in most series. In BTN, the pain rarely begins concurrently on both sides and is often more severe on one side. Although is important to exclude secondary causes, the vast majority of cases are attributed to vascular compression or are idiopathic.

**Methods:** Case report

**Results:** A 63-year-old man presented with typical right-side trigeminal neuralgia (TN) for 7 years, involving the second and third divisions. The neurological examination was normal. He underwent a course of medical therapy with carbamazepine but due to adverse effects, it was switched to gabapentin up to 2400mg/day with good pain control. After 3 years of being asymptomatic, the patient complained about the newly begun paroxysmal electrical pain on the second and third divisions of the bilateral trigeminal nerve. MRI excluded secondary cases but revealed bilateral neurovascular contacts. The patient tried to control his pain with oxcarbazepine (up to 900mg/day) and gabapentin (up to 2400mg/day) but the pain remained refractory. After discussed with the other therapeutic modalities, the patient started onabotulinumtoxinA (total units: 40). After 9 months of treatment the patient remains without uni or bilateral pain.

**Conclusion:** BTN is a rare entity and the treatment could be challenging. Patients may develop side effects from centrally acting drugs, have contraindications or complications for neurosurgical procedures, or experience relapse during conventional therapies. This case highlights that onabotulinumtoxinA could be a safe and effective option, in patients who are refractory to pharmacological treatment and an alternative to surgery.

*Disclosure statement*: Informed consent to publish this case study and its potentially identifiable information of the patient was obtained from the individual involved. The patient gave explicit permission for the publication of this case report, including any relevant clinical details.

## P019 Benefit-Risk assessment of atogepant vs. placebo in participants with episodic migraine who had prior inadequate response to 2–4 classes of oral preventive treatment: a post-hoc analysis of the ELEVATE trial

### U. Reuter^1^, A. Lalla^2^, S. Sacco^3^, D. Holle-Lee^4^, P. Pozo-Rosich^5,6^, K. Nagy^7^, L. Luo^8^, K. Carr^9^, P. Gandhi^8^, C. Tassorelli^10^

#### ^1^Charité – Universitätsmedizin Berlin, Berlin, Germany; ^2^AbbVie, Irvine, CA, United States; ^3^University of L'Aquila, L'Aquila, Italy; ^4^West German Headache and Vertigo Center Essen, Department of Neurology, Essen, Germany; ^5^Vall d’Hebron Hospital & Research Institute, Universitat Autonoma de Barcelona, Barcelona, Spain; ^6^Vall d’Hebron Hospital & Research Institute, Universitat Autonoma de Barcelona, Barcelona, Spain; ^7^AbbVie, Budapest, Hungary; ^8^AbbVie, Madison, NJ, United States; ^9^AbbVie, North Chicago, IL, United States; ^10^Headache Science & Neurorehabilitation Centre, C. Mondino Foundation and University of Pavia, Pavia, Italy

##### **Correspondence:** U. Reuter

*The Journal of Headache and Pain 2024,* **25(Suppl 1)**: P019

**Objective:** To determine the number needed to treat (NNT) and number needed to harm (NNH) for atogepant vs placebo for the preventive treatment of episodic migraine (EM) in participants with prior inadequate response to 2-4 classes of oral preventive treatment.

**Methods:** ELEVATE was a global, multicenter, randomized, double-blind, placebo-controlled study in participants with EM with prior inadequate response to 2-4 classes of oral preventive treatment. Participants were randomized to atogepant 60 mg once daily or placebo in a 1:1 ratio. The NNT was calculated based on achievement of a ≥50% decrease from baseline in mean monthly migraine days (MMDs) (NNT_MMD≥50%_) across 12 weeks. A second NNT was calculated based on achievement of a ≥10.9-point clinically relevant improvement from baseline in Migraine-Specific Quality of Life questionnaire version 2.1 Role Function-Restrictive (MSQ-RFR) score (NNT_MSQ-RFR_; Dodick. *Headache* 2007;47:1398) at week 12. NNH was calculated using the proportion of participants reporting a treatment-emergent adverse event (TEAE) leading to discontinuation.

**Results:** Of atogepant participants, 50.6% experienced a ≥50% reduction in MMDs from baseline vs 18.1% of placebo participants across the 12-week double-blind treatment period (Figure 1). The calculated NNT_MMD≥50%_ for atogepant was 3.1, indicating that 3 participants needed to be treated with atogepant for 1 additional participant to experience a ≥50% treatment response. The NNT_MSQ-RFR_ for atogepant was 3.2 (Figure 2). TEAEs leading to discontinuation were reported by 1.9% of atogepant vs 1.3% of placebo participants. The NNH was 167 for atogepant, indicating that 167 participants would need to be treated before 1 would observe TEAEs leading to discontinuation.

**Conclusion:** Atogepant demonstrated a positive benefit-risk profile vs placebo for the preventive treatment of EM based on the low NNT and high NNH in participants with prior inadequate response to 2-4 classes of oral preventive treatment.

**Fig. 1 (Abstract P019):**
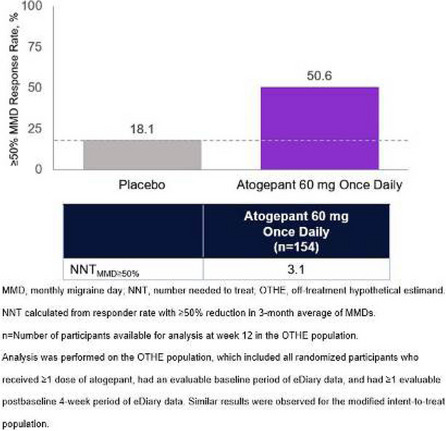
Number needed to treat: monthly migraine days

**Fig. 2 (Abstract P019):**
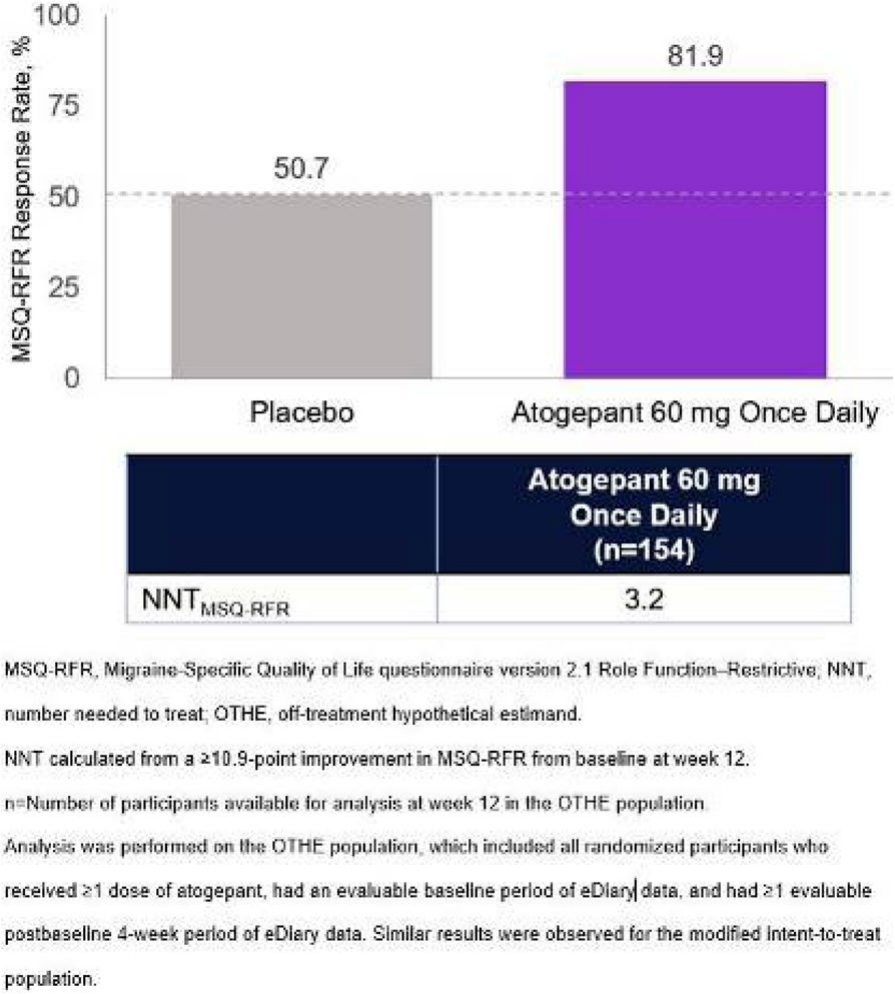
Number needed to treat: MSQ role function-restrictive

## P021 Predictors of ≥50% and ≥75% response to anti-CGRP monoclonal antibodies for migraine prevention in real life: results from a european multicenter observational study including 5818 patients

### E. Caronna^1^, V. J. Gallardo^1^, A. Alpuente^1^, M. Torres-Ferrús^1^, M. Huerta Villanueva^2^, A. Muñoz-Vendrell^3^, S. Campoy Díaz^2^, V. Obach Baurier^4^, N. Fabregat Fabra^4^, A. Gago-Viega^5^, A. Sánchez Soblechero^6^, A. Lozano Ros^6^, J. Díaz de Terán^7^, J. A. Membrilla López^7^, J. S. Rodriguez-Vico^8^, Á. L. Guerrero Peral^9^, D. García Azorín^9^, J. Pascual^10^, G. Gárate^10^, E. Cuadrado Godia^11^, L. Dorado^12^, J. Sanahuja^13^, A. Echeverria Urabayen^14^, P. Irimia Sieira^15^, M. Sánchez del Río^16^, A. López-Bravo^17^, R. Álvarez Escudero^18^, F. Velasco Juanes^19^, S. Santos Lasaosa^20^, S. Díaz Insa^21^, A. Layos-Romero^22^, A. Andrés López^22^, J. Viguera Romero^23^, I. Beltrán Blasco^24^, C. González Oria^25^, S. Zapata^26^, B. Flores Pina^12^, I. Fernández Lázaro^5^, S. Quintas Gutierrez^5^, A. González Martínez^5^, A. Jaimes Sanchez^8^, A. García Gómez^8^, Y. González Osorio^9^, V. González-Quintanilla^10^, I. Kortazar Zubizarreta^14^, P. Manera Zorrilla-Lequerica^11^, I. Miró Muñoz^11^, D. Guisado-Alonso^11^, A. Oterino Durán^18^, N. Riesco Pérez^18^, B. Venegas Pérez^18^, W. Sifontes^19^, J. Rodriguez Montolio^20^, M. P. Navarro Pérez^20^, C. Nieves Castellanos^21^, M. A. Olivier^21^, F. Sánchez Caballero^23^, Y. Vaamonde Esteban^24^, P. Ros Arlanzón^24^, R. Lamas Pérez^25^, M. Millán Vázquez^25^, B. Nunes Vicente^27^, I. Pavão Martins^27^, L. Pereira^28^, E. Martins-Silva^28^, M. Rodrigues^28^, G. Cabral^29^, A. Caetano^29^, M. Viana Baptista^29^, I. Luzeiro^30^, C. Fernandes^30^, I. Brás Marques^31^, E. Parreira^31^, R. Oliveira^31^, M. Waliszewska-Prosół^32^, C. Heidemann Sundal^33^, M. Ghadiri-Sani^34^, H. Basedau^35^, A. May^35^, C. Thunstedt^36^, A. Straube^36^, V. Caponnetto^37^, R. Ornello^37^, G. Vaghi^38^, R. De Icco^38^, R. Ruscheweyh^36^, R. Gil-Gouveia^31^, G. Egeo^39^, S. Cevoli^40^, P. Torelli^41^, S. Sacco^37^, C. Tassorelli^38^, P. Barbanti^39^, P. Pozo-Rosich^1^

#### ^1^Vall d’Hebron Hospital & Research Institute, Universitat Autonoma de Barcelona, Headache Clinic, Neurology Department, Barcelona, Spain; ^2^Hospital Villadecans, Barcelona, Spain; ^3^Hospital de Bellvitge, Barcelona, Spain; ^4^Hospital Clinic, Barcelona, Spain; ^5^Hospital de la Princesa, Madrid, Spain; ^6^Hospital Gregorio Marañon, Madrid, Spain; ^7^Hospital Universitario La Paz, Madrid, Spain; ^8^Fundación Jiménez Díaz, Madrid, Spain; ^9^Hospital Clínico Universitario de Valladolid, Valladolid, Spain; ^10^University Hospital Marqués de Valdecilla, Santander, Spain; ^11^Hospital del Mar, Barcelona, Spain; ^12^Hospital Universitario Germans Trias i Pujol, Barcelona, Spain; ^13^Hospital Arnau de Villanova, Lleida, Spain; ^14^H Universitario de Álava, Álava, Spain; ^15^Clínica Universidad de Navarra, Pamplona, Spain; ^16^Clinica Universidad de Navarra, Madrid, Spain; ^17^Hospital Reina Sofía, Tudela, Spain; ^18^Hospital Universitario Central de Asturias, Oviedo, Spain; ^19^Hospital Universitario Cruces, Bilbao, Spain; ^20^Hospital Universitario Lozano Blesa, Zaragoza, Spain; ^21^Hospital Universitario La Fe, Valencia, Spain; ^22^Hospital General Universitario de Albacete, Albacete, Spain; ^23^Hospital Virgen de la Macarena, Sevilla, Spain; ^24^Hospital General Universitario Dr. Balmis, Alicante, Spain; ^25^Hospital Virgen del Rocío, Sevilla, Spain; ^26^Universidad de Antioquia, Antioquia, Colombia; ^27^Hospital de Santa Maria, Lisbon, Portugal; ^28^Hospital Garcia de Orta, Almada, Portugal; ^29^Hospital de Egas Moniz – Centro Hospitalar de Lisboa Ocidental, Lisbon, Portugal; ^30^Hospitalar and University Center of Coimbra, Coimbra, Portugal; ^31^Hospital da Luz, Lisbon, Portugal; ^32^Wrocław Medical University, Wrocław, Poland; ^33^University of Gothenburg, Gothenburg, Sweden; ^34^The Walton Centre NHS Foundation Trust, Liverpool, United Kingdom; ^35^University Hospital Hamburg-Eppendorf, Hamburg, Germany; ^36^Klinikum der LMU München, Munich, Germany; ^37^Department of Biotechnological and Applied Clinical Sciences University of L'Aquila, L'Aquila, Italy; ^38^IRCCS Fondazione Istituto Neurologico Nazionale C. Mondino, Pavia, Italy; ^39^IRCCS San Raffaele Roma, Rome, Italy; ^40^IRCCS Istituto delle Scienze Neurologiche Bologna, Bologna, Italy; ^41^University of Parma, Parma, Italy

##### **Correspondence:** E. Caronna

*The Journal of Headache and Pain 2024,* **25(Suppl 1)**: P021

**Objective:** To identify predictors of ≥50% and ≥75% response to anti-CGRP monoclonal antibodies (mAbs) in real life in Europe.

**Methods:** European multicenter, observational study based on prospective registries of adult patients with high-frequency episodic (HFEM) or chronic (CM) migraine treated with anti-CGRP mAbs since March 2018. We collected demographic data, efficacy variables (monthly headache days-MHD; monthly migraine days-MDM, monthly acute medication days-MAMD). Response and excellent response were defined as ≥50% and ≥75% reduction in MHD, respectively; non-responders as <30% reduction in MHD. We used a generalized mixed-effect regression model (GLMM) to identify variables independently associated with treatment response rate (≥50% and ≥75%).

**Results:** We included a total of 5818 patients (84.2% females; median [IQR] age 47.0y [39.0-53.0]). 72.2% (4,198/5,818) had CM. At month 6, we observed a median reduction in MHD of -9.0 [-15.0, -3.0] days/month and the response rate was: 30.3% (1,503/4,963) non-responders, 13.2% (656/4,963) partial-responders (30-50% reduction MHD) and 56.5% (2,804/4,963) responders (≥50%). With GLMM, older age (OR [95% CI], 1.08 [1.01-1.16]; *p*=0.023), CM diagnosis at baseline (1.39 [1.16-1.66], *p*<0.001), higher ratio of MMD at baseline (1.02 [1.01-1.03], *p*<0.001) and presence of unilateral pain (1.52 [1.31-1.75], *p*<0.001) were predictors associated with higher likelihood of ≥50% response. The model presented an accuracy [95% CI] 0.635 [0.620-0.650], Kappa 0.428 and AUC 0.669 [0.653-0.686]. We repeated the previous analysis between non-responders and excellent-responders (26.7%; 1,324/4,963) at month 6. In the GLMM, statistically significantly independent variables were similar as with the excellent responders.

**Conclusion:** Older age and migraine-specific clinical features are associated with an excellent response to anti-CGRP mAbs, in a large real-world European cohort

## P022 Atogepant demonstrates early functional and quality of life improvements for the preventive treatment of episodic migraine after prior inadequate response to treatment: results from the ELEVATE trial

### D. Holle-Lee^1^, P. Gandhi^2^, U. Reuter^3,4^, J. Ailani^5^, R. B. Lipton^6^, K. Nagy^7^, Y. Liu^8^, K. Carr^8^, J. Stokes^2^, C. Tassorelli^9,10^

#### ^1^University of Essen, Department of Neurology, West German Headache and Vertigo Center Essen, Essen, Germany; ^2^AbbVie, Madison, NJ, United States; ^3^Charité – Universitätsmedizin Berlin, Department of Neurology, Berlin, Germany; ^4^Universitätsmedizin Greifswald, Greifswald, Germany; ^5^Georgetown University Hospital, Washington, DC, United States; ^6^Albert Einstein College of Medicine, Bronx, MA, United States; ^7^AbbVie, Budapest, Hungary; ^8^AbbVie, North Chicago, IL, United States; ^9^University of Pavia, Pavia, Italy; ^10^C. Mondino Foundation and University of Pavia, Headache Science Centre, Pavia, Italy

##### **Correspondence:** D. Holle-Lee

*The Journal of Headache and Pain 2024,* **25(Suppl 1)**: P022

**Objective:** Evaluate the impact of atogepant (ATO) on daily functioning and quality of life (QoL) in the first month of treatment among participants with episodic migraine (EM) who experienced prior inadequate response to treatment.

**Methods:** ELEVATE was a phase 3, multicenter, randomized, double-blind, placebo (PBO)-controlled, parallel-group trial conducted in Europe and North America. Adults with EM (4-14 mean monthly migraine days during the 28-day baseline period) who had previously experienced an inadequate response to 2-4 classes of conventional oral preventive migraine treatments were randomized to ATO 60 mg once daily (QD) or PBO. Patient-reported outcome measures included the Performance of Daily Activities (PDA) and Physical Impairment (PI) domains of the 11-item Activity Impairment in Migraine – Diary (AIM-D), and the 5-level European Quality of Life – 5 Dimension (EQ-5D-5L) descriptive system and visual analogue scale (VAS), implemented via daily diary. Changes from baseline in weekly PDA and PI scores were calculated for weeks 1, 2, 3, and 4. Changes from baseline in EQ-5D-5L were evaluated for weeks 1-2 and at week 4 using diary data at these time points in the trial.

**Results:** Of the 313 participants who received treatment, 309 were included in the Off-treatment Hypothetical Estimand population (ATO 60 mg QD, *n*=154; PBO, *n*=155). ATO 60 mg QD demonstrated greater improvements in PDA (Figure 1) and greater reductions in PI (Figure 2) as early as week 1, through weeks 2, 3, and 4, relative to PBO (nominal *P*<.05). ATO 60 mg QD showed greater improvements for the EQ-5D-5L descriptive system and VAS scores at weeks 1-2 and 4 (Figure 3), relative to PBO (nominal *P*<.05).

**Conclusion:** Participants with EM and prior inadequate response to conventional oral preventive treatment taking ATO 60 mg QD demonstrated rapid improvement in function as early as week 1 and QoL by week 2, compared to PBO.

**Fig. 1 (Abstract P022):**
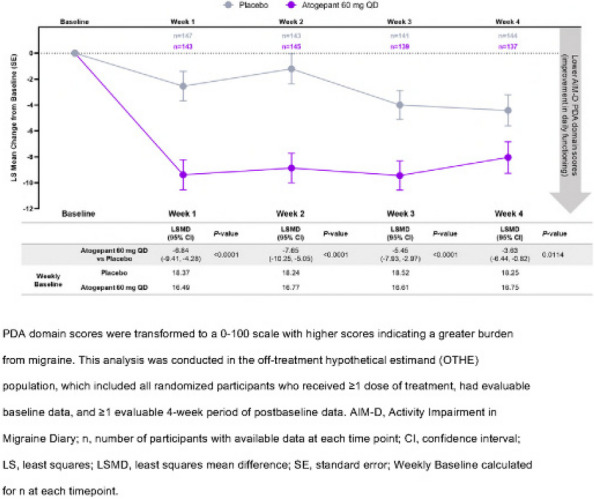
Change from baseline in the Perfomance of Daily Activities (PDA) domain of the AIM-D at weeks 1, 2, 3, and 4 (OTHE population)

**Fig. 2 (Abstract P022):**
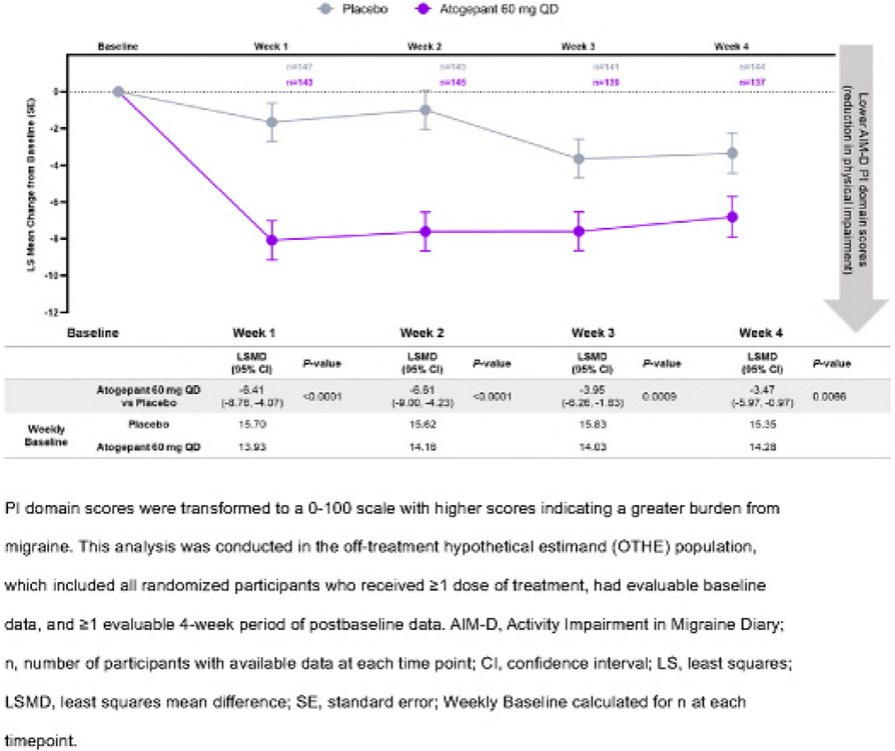
Change from baseline in the Physical Impairment (PI) domain of the AIM-D at weeks 1, 2, 3, and 4 (OTHE population)

**Fig. 3 (Abstract P022):**
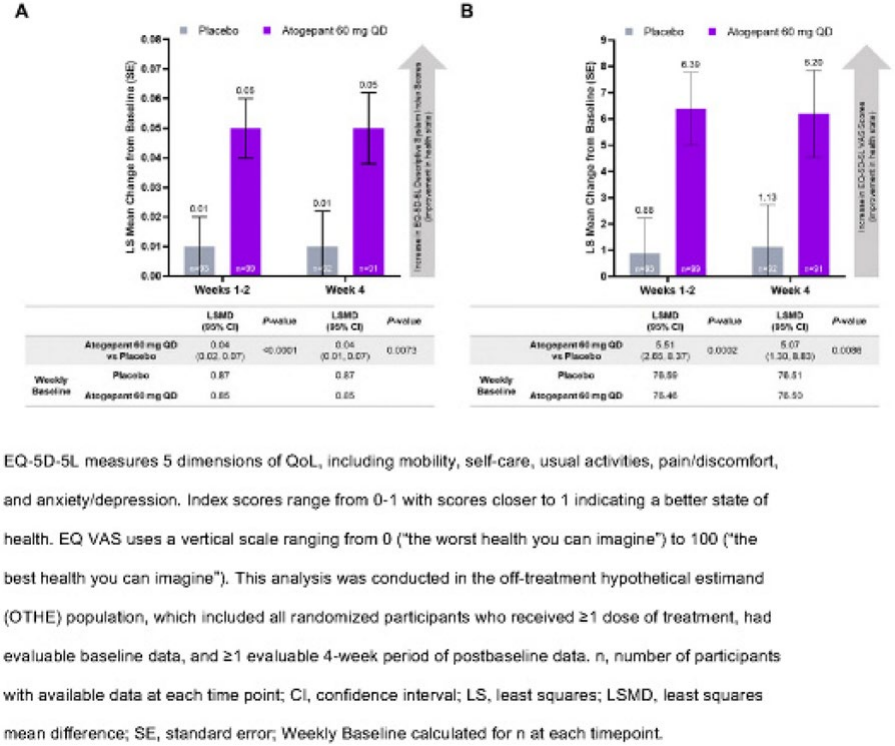
Change from baseline in European quality of life – 5 dimensional (EQ-5D-5L) (**A**) descriptive system index score and (**B**) Visual Analogue Scale (VAS) score at weeks 1-2 and 4 (OTHE population)

## P023 Effectiveness of galcanezumab versus traditional oral migraine preventive medications: 3-month findings from the TRIUMPH observational study

### P. Pozo-Rosich^1^, C. Tassorelli^2^, M. Vincent^3^, C. Vallarino^4^, L. Viktrup^3^, R. L. Robinson^5^

#### ^1^Vall d’Hebron Hospital & Research Institute, Universitat Autonoma de Barcelona, Neurology, Barcelona, Spain; ^2^University of Pavia, Department of Brain and Behavioral Sciences, Pavia, Italy; ^3^Eli Lilly and Company, Medical Affairs Migraine/Headache, Indianapolis, IN, United States; ^4^Eli Lilly and Company, Real World and Access Analytics, Indianapolis, IN, United States; ^5^Eli Lilly and Company, Value Evidence Outcomes - Research & Neuroscience, Indianapolis, IN, United States

##### **Correspondence:** P. Pozo-Rosich

*The Journal of Headache and Pain 2024,* **25(Suppl 1)**: P023

**Objective:** Among patients (pts) with migraine who initiated or switched to a new migraine preventive pharmacological treatment, what is the 3-month real-world effectiveness of galcanezumab versus (vs.) traditional oral migraine preventive medications (TOMP)?

**Methods:** Data from the TRIUMPH study were analyzed; adults with ICHD-3 diagnosis of migraine were included. The primary endpoint was to determine the proportion of patients with clinically meaningful response (responders with ≥50% for episodic migraine [EM]; ≥30% for chronic migraine [CM]) in monthly migraine headache days (MHD) at month 3 by galcanezumab vs. TOMP cohorts. Secondary endpoints included mean change from baseline in function (using MSQ v2.1 Role Function-Restrictive [RFR]) and monthly MHD, "enhanced response" (EM: ≥75%, CM: ≥50% reduction in MHD), and responders in subsets with EM or CM. Inverse probability of treatment weights were derived from a LASSO propensity score model and were used to compare cohorts. Change in outcomes were assessed at 3-months from baseline.

**Results:** Of the 2879 pts at baseline, 1123 (39.0%) initiated or switched to galcanezumab (mean ± SD age: 44.9 ± 13.4 years); 1314 (45.6%) pts initiated or switched to TOMP (mean ± SD age: 39.8 ± 13.1 years). In the galcanezumab cohort, 48.6% of pts achieved clinically meaningful response at 3-months vs. 35.7% in TOMP cohort (*p*<.0001). The galcanezumab cohort had improved functioning vs. TOMP cohort [mean and 95% confidence interval: 19.5 (17.6, 21.3) vs. 10.0 (8.4, 11.6), *p*<.0001], and had reductions in monthly MHDs [mean and 95% confidence interval: -5.7 (-6.2, -5.2) vs. -4.2 (-4.6, -3.8), *p*<.0001]. The proportion of responders and "enhanced responders" for each type of migraine were significantly higher for galcanezumab vs. TOMP (responders: EM: 50.5% vs. 30.3%, *p*<.0001; CM: 47.8% vs. 39.0%, *p*=.001; enhanced responders: EM: 28.2% vs. 13.2%, *p*<.0001; CM: 37.3% vs. 31.4%, *p*=.02).

**Conclusion:** Across multiple measures of 3-month effectiveness, galcanezumab treatment yielded greater improvements than TOMP among patients with migraine in this real-world study.

## P024 Three-and-six month effectiveness of CandeSpartan in the treatment of migraine: the CandeSpartan study (NCT: 04138316)

### C. Martinez-Badillo^1^, J. Camina-Muniz^2^, A. Gago-Viega^3^, N. Morollón Sánchez-Mateos^4^, V. González-Quintanilla^5^, J. Porta-Etessam^6^, A. Recío García^1^, Y. González Osorio^1^, Á. Sierra-Mencía^1^, Á. L. Guerrero Peral^1^, D. García Azorín^1^

#### ^1^Hospital Clínico Universitario de Valladolid, Neurology, Valladolid, Spain; ^2^Hospital Universitari Son Espases, Neurology, Palma de Mallorca, Spain; ^3^Hospital Universitario de la Princesa, Neurology, Madrid, Spain; ^4^Hospital de la Santa Creu i Sant Pau, Neurology, Barcelona, Spain; ^5^University Hospital Marqués de Valdecilla, Neurology, Santander, Spain; ^6^Hospital Clinico San Carlos, Neurology, Madrid, Spain

##### **Correspondence:** C. Martinez-Badillo, Á. L. Guerrero Peral, D. García Azorín

*The Journal of Headache and Pain 2024,* **25(Suppl 1)**: P024

**Objective:** To assessed the effectiveness of candesartan in a real-world setting.

**Methods:** Observational, multicenter, prospective cohort study including adult patients with episodic and chronic migraine, according to The International Classification of Headache Disorders treated with candesartan. Patients were excluded if 1) they had failed to >3 preventive drugs, 2) received another drug with effectiveness as migraine preventive, 3) had history of another headache disorder, 4) had previously used candesartan, 5) had any severe disorder. The effectiveness was evaluated by the 50%, 75% and 30% responder rates the reduction in monthly migraine days (MMD), between weeks 8-12 and weeks 20-24, compared with the baseline. All results were calculated per-protocol (PP) and by intention-to-treat (ITT), using baseline-carried-forward approach in the case of missing data.

**Results:** Eighty-six patients were included, 68 (79.1%) females, aged 40 [inter-quartile range (IQR) 26.1-49.9], 37 (43.0%) with chronic migraine, 48 (55.8%) with medication overuse headache and a median number of prior preventive treatments of 2 [IQR: 0.75-3[U1] ]. At baseline patients had 14 [10-24] headache days per month, 10 [5-15] days of use of acute medication, and 5 [0-8] days of triptan use. At 3-months, 30%, 50% and 75% responder rates were 52.4%; 36.6% and 15.9% in the PP analysis; and 50%; 34.9% and 15.1% in the ITT analysis. At 6-months, 30%, 50% and 75% responder rates were 63.6%, 47% and 24.2% (PP) and 48.8%, 36%, and 18.6% (ITT). The reduction of MMD was 4.5 [IQR:-1 – 4] days (PP) and 4 [0-9.25] days (ITT) at 3-months, and 5 [0.7-14.2] (PP) days and 3 [0-10.5] days (ITT).

**Conclusion:** The effectiveness of candesartan in the preventive of migraine in a real-world setting was in line with the observed efficacy from the RCTs.

## P025 6-Month Real-World Effectiveness of Fremanezumab in Patients with Migraine who switched from another mAb targeting the CGRP pathway (subgroup analysis from FINESSE)

### A. Straube^1^, G. Broessner^2^, C. Gaul^3^, X. Hamann^4^, J. Hipp^4^, T. Kraya^5^, L. Neeb^6^

#### ^1^University Hospital LMU Munich, Munich, Germany; ^2^Innsbruck Medical University, Department of Neurology, Innsbruck, Austria; ^3^Headache Center Frankfurt, Frankfurt a. M., Germany; ^4^Teva GmbH, Ulm, Germany; ^5^Hospital Sankt Georg Leipzig gGmbH, Headache Center Halle, Leipzig, Germany; ^6^Helios Global Health, Charité Universitätsmedizin Berlin, Berlin, Germany

##### **Correspondence:** A. Straube

*The Journal of Headache and Pain 2024,* **25(Suppl 1)**: P025

**Objective:** To evaluate effectiveness and tolerability of fremanezumab administered in migraine patients who switched from a previous anti-CGRP pathway mAb (aCGRP mAb) as part of their routine disease management.

**Methods:** FINESSE is an ongoing prospective, non-interventional study in adults with episodic or chronic migraine (EM, CM). Observation period: 24 months. Primary endpoint: proportion of patients reaching ≥50% reduction in average number of monthly migraine days (MMD) during the 6-month period after the first dose of fremanezumab. Further measures: monthly average number of migraine days, MIDAS (Migraine Disability Assessment), HIT-6 (6-Item Headache Impact Test), acute migraine medication (AMM) use. In this subgroup analysis, 6-month-data in patients who experienced poor effectiveness or tolerability with a prior anti-CGRP pathway mAb and therefore switched to fremanezumab are presented.

**Results:** 140 patients with prior exposure to another aCGRP mAb were included (47.6 ± 11.5 years, 84.7% female); 56.4% had EM, 43.6% CM. 126 patients had been previously treated with erenumab, 14 with galcanezumab or galcanezumab and erenumab. The main reason for discontinuation of prior aCGRP mAb therapy was lack of efficacy (LOE) in 110 patients (78.6%). MMD decreased from 13.3 ± 6.42 at baseline (B), by 6.1 ± 5.47 (month 6). 54 (38.6%) achieved a MMD reduction of ≥ 50% over 6 months (EM 44.3%, CM 31.2%, in patients with LOE 40%). AMM was used on 9.5 ± 4.92 days/month at baseline and decreased to 5.0 ± 3.86 days/month (month 6). MIDAS: 71.1 ± 57.1 (B), 43.7 ± 44.4 (month 6), HIT-6: 65.8 ± 4.8 (B), 59.6 ± 7.9 (month 6).

**Conclusion:** In this interim analysis of the FINESSE non-interventional study, about 38.6% of anti-CGRP pathway mAb-non-responder benefit (≥ 50% response) from switching to fremanezumab. These results suggest that switching to fremanezumab may be a promising option for patients experiencing inadequate efficacy or poor tolerability with prior other anti-CGRP pathway mAb use.

## P026 Effectiveness of galcanezumab vs. traditional oral migraine preventive medications: 3-month results from Real-World TRIUMPH study for Europe [Germany, Italy, Spain, and the United Kingdom]

### C. Tassorelli^1^, D. Novick^2^, S. Gonderten^2^, M. Vincent^2^, R. L. Robinson^2^, G. Martimianaki^2^, P. Pozo-Rosich^3^

#### ^1^University of Pavia, Department of Brain and Behavioral Sciences, Pavia, Italy; ^2^Eli Lilly and Company, Indianapolis, IN, United States; ^3^Vall d’Hebron Hospital & Research Institute, Universitat Autonoma de Barcelona, Barcelona, Spain

##### **Correspondence:** S. Gonderten

*The Journal of Headache and Pain 2024,* **25(Suppl 1)**: P026

**Objective:** It is important to assess the real-world impact of migraine preventive therapies. TRIUMPH, an ongoing observational study, assesses outcomes of such treatments with the primary objective of comparing the effectiveness of galcanezumab to traditional oral migraine preventive medications (TOMP) in patients with migraine switching/initiating preventive treatment in clinical practice. We present TRIUMPH [Europe] results for 3-month outcomes.

**Methods:** TRIUMPH [Europe] enrolled patients ≥18 years old with migraine from Germany, Italy, Spain, and the UK. Patients were enrolled 06/2020-08/2022 and taking on-label galcanezumab or TOMP. Effectiveness was defined as reduction from baseline in patient-reported and physician-recorded monthly migraine headache days (MHD) at 3 months (response): ≥30% / ≥50% in chronic / episodic migraine. Differences in proportion of responders across treatments were assessed using a weighted Chi-squared test. Weights were derived using Least Absolute Shrinkage and Selection Operator (LASSO) model fit of propensity scores with 65 baseline covariates.

**Results:** 630 patients were enrolled, 53% in the galcanezumab and 47% in the TOMP cohorts. Patients were more often women, with a mean age of 44.4/36.9 years. Chronic migraine was more represented in the galcanezumab cohort (61%/33%). Mean (SD) MHD were 14.8 (7.1)/10.4 (5.3) days. At 3 months, the galcanezumab and TOMP cohorts" mean (SD) MHD were 7.7 (7.4)/6.9 (5.5) days. Change from baseline in the number of mean MHD with acute medication use was –6.5 for galcanezumab and –2.7 for TOMP (*P*<0.0001). The 3-month weighted proportion of responders for galcanezumab was higher than that for TOMP (62.0% vs. 34.2% *p*<0.0001). The weighted proportion of responders in the galcanezumab cohort was greater than that of the TOMP cohort also for the chronic and episodic migraine subsets, 64.5% vs. 38.2%, *p*<0.0001 and 58.7% vs 31.6%, *p*<0.0001, respectively.

**Conclusion:** After 3 months of migraine preventive treatment**,** patients with migraine initiating/switching to galcanezumab had a substantially higher proportion of responders than those receiving TOMP.

## P027 Efficacy of rimegepant for the acute treatment of migraine in chinese and korean adults receiving concurrent preventive medication

### S. Yu^1^, Z. Lu^2^, Y. Liu^3^, Y. Zou^4^, Y. Sun^5^, J. Atkinson^6^

#### ^1^Chinese PLA General Hospital, Beijing, China; ^2^Pfizer (China) Research and Development Ltd, Shanghai, China; ^3^Pfizer, Inc., Beijing, China; ^4^Pfizer, Inc., Shanghai, China; ^5^Pfizer CRDC, Shanghai, China; ^6^Pfizer, LTD, Tadworth, United Kingdom

##### **Correspondence:** J. Atkinson

*The Journal of Headache and Pain 2024,* **25(Suppl 1)**: P027

**Objective:** To investigate the efficacy of rimegepant, an orally administered small molecule calcitonin gene-related peptide receptor antagonist, for the acute treatment of migraine in a subgroup of Chinese and Korean adults receiving concurrent preventive medication.

**Methods:** These subgroup analyses were from a phase 3, double-blind study conducted in China and Korea (NCT04574362) of single-dose rimegepant 75 mg or placebo for the acute treatment of moderate or severe migraine. Eligibility criteria included 2–8 moderate or severe migraine attacks per month. Co-primary endpoints at 2 hours (h) postdose were pain freedom and freedom from the most bothersome symptom (MBS), analyzed using Cochran-Mantel-Haenszel tests. Subgroup analyses were based on concurrent preventive medication use (yes/no).

**Results:** Of the 1340 participants analyzed (rimegepant *n*=666, placebo *n*=674), 81.2% were female, 80.1% were from China, 19.9% were from Korea, and 5.4% (rimegepant *n*=38, placebo *n*=35) were taking concurrent preventive medication. In the small subgroup taking preventive medication, response rates for pain freedom at 2 h were greater for rimegepant than placebo (18.4% vs 0%, respectively; *P*=0.0103); for freedom from the MBS at 2 h the difference between rimegepant and placebo (57.9% vs 37.1%; *P*=0.0726) showed similar trends but did not reach statistical significance (Figure). In the larger subgroup not taking preventive medication, response rates for pain freedom at 2 h (19.9% vs 11.3%; *P*<0.0001) and freedom from the MBS at 2 h (50.0% vs 35.7%; *P*<0.0001) were greater with rimegepant than placebo (Figure). Rimegepant was well tolerated.

**Conclusion:** Among participants who were taking concurrent preventive medication, more experienced pain freedom at 2 h after taking rimegepant 75 mg for the acute treatment of migraine compared with placebo. Rimegepant was effective compared with placebo in participants who were not taking concurrent preventive medication. The subgroup taking preventive medication was small so findings should be interpreted cautiously. Funded by Pfizer.

**Fig. 1 (Abstract P027):**
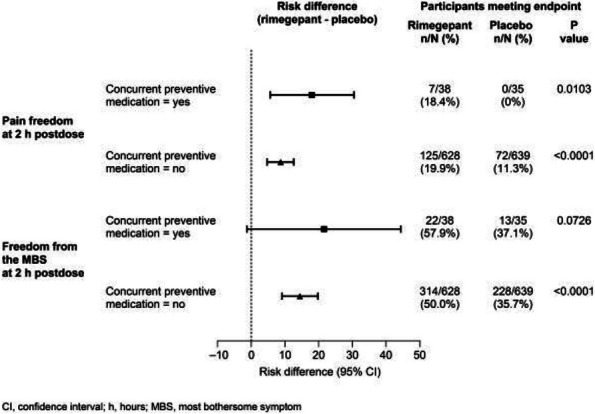
Paticipants meeting co-primary endpoints. CI, confidence interval; h, hours; MBS, most bothersome symptom

## P028 UNITE: Efficacy and impact of fremanezumab in patients with migraine and major depressive disorder

### V. Ramirez Campos^1^, R. B. Lipton^2^, L. J. Krasenbaum^1^, X. Ning^1^, P. Barbanti^3,4^, Z. Roth-Ben Arie^5^, M. Galic^6^, L. Denysenko^7,8^, M. Marmura^7^, P. McAllister^9^, D. D. Mitsikostas^10^

#### ^1^Teva Branded Pharmaceutical Products R&D, Inc., West Chester, PA, United States; ^2^Albert Einstein College of Medicine, Departments of Neurology, Psychiatry & Behavioral Sciences, New York, NY, United States; ^3^IRCCS San Raffaele Roma, Headache & Pain Unit, Rome, Italy; ^4^San Raffaele University, Rome, Italy; ^5^Teva Pharmaceutical Industries Ltd., Petah Tikva, Israel; ^6^TEVA-PHARMA, Produtos Farmacêuticos, Lda., Porto Salvo, Portugal; ^7^Thomas Jefferson University Hospital, Jefferson Headache Center, Department of Neurology, Philadelphia, PA, United States; ^8^Thomas Jefferson University Hospital, Department of Psychiatry & Human Behavior, Philadelphia, PA, United States; ^9^New England Institute for Neurology and Headache, Stamford, CT, United States; ^10^National and Kapodistrian University of Athens, Department of First Neurology, Aeginition Hospital, Athens, Greece

##### **Correspondence:** P. Barbanti

*The Journal of Headache and Pain 2024,* **25(Suppl 1)**: P028

**Objective:** To evaluate the efficacy and impact of fremanezumab in patients with migraine and major depressive disorder (MDD).

**Methods:** A 12-week randomized (1:1), Phase 4 study with a 12-week open-label extension (OLE). Patients (12 months MDD [DSM-V, PHQ-9 score ≥ 10]) received monthly fremanezumab (225 mg) or matched placebo for 12 weeks. All patients in the OLE received quarterly fremanezumab (675 mg). Primary endpoint: mean change (baseline to Week 12) in average number of monthly migraine days (MMD). Secondary endpoints: mean change from baseline in symptoms of depression (HAMD-17, PHQ-9), disability (HIT-6, CGI-S), and patients achieving ≥50% MMD reduction.

**Results:** 330/353 patients (fremanezumab, *n* = 164; placebo, *n* = 166) completed the 12-week double-blind period. Fremanezumab showed statistically significant reductions in MMD, and in depression and disability scores (Table). All results were maintained through the OLE. Despite the different patient population included in this study, the safety profile was consistent with previous pivotal fremanezumab randomized controlled trials.

**Conclusion:** During the 12-week double blind period, fremanezumab treatment resulted in a significant reduction in MMD, along with improvements in depression and disability outcomes, which were maintained over the longer term. Fremanezumab may reduce the burden of these comorbid diseases and improve quality of life.

**Table 1 (Abstract P028):**
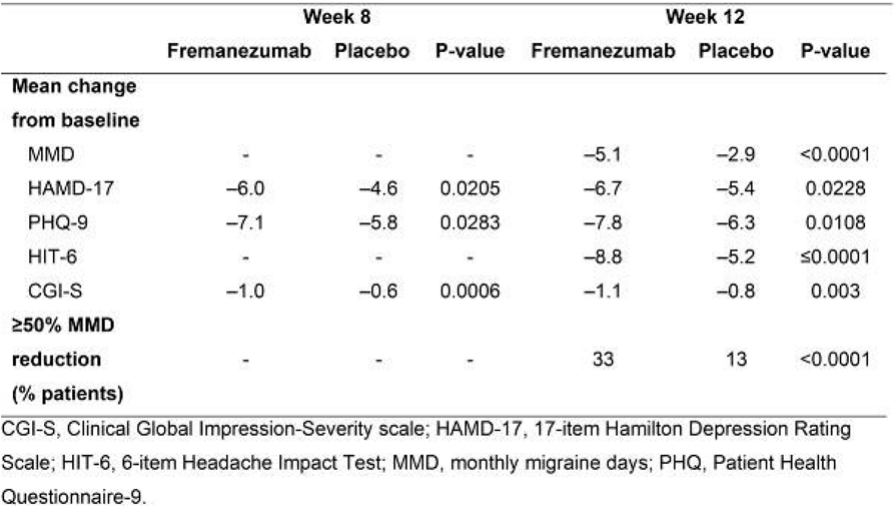
Primary and secondary endpoints

## P029 Benefit-Risk assessment based on number needed to treat and number needed to harm: Atogepant vs. calcitonin gene–related peptide monoclonal antibodies

### S. Sacco^1^, A. Lalla^2^, R. B. Halker Singh^3^, D. Holle-Lee^4^, P. Pozo-Rosich^5,6^, K. Nagy^7^, K. Kelton^8^, C. Piron^8^, P. Gandhi^9^, J. Ailani^10^

#### ^1^University of L'Aquila, L'Aquila, Italy; ^2^AbbVie, Irvine, CA, United States; ^3^Mayo Clinic, Scottsdale, AZ, United States; ^4^West German Headache and Vertigo Center Essen, Department of Neurology, Essen, Germany; ^5^Vall d’Hebron Hospital & Research Institute, Universitat Autonoma de Barcelona, Barcelona, Spain; ^6^Vall d’Hebron Hospital & Research Institute, Universitat Autonoma de Barcelona, Barcelona, Spain; ^7^AbbVie, Budapest, Hungary; ^8^Medical Decision Modeling Inc., Indianapolis, IN, United States; ^9^AbbVie, Madison, NJ, United States; ^10^MedStar Georgetown University Hospital, Washington, DC, United States

##### **Correspondence:** S. Sacco

*The Journal of Headache and Pain 2024,* **25(Suppl 1)**: P029

**Objective:** To evaluate the benefit-risk assessment of atogepant and calcitonin gene–related peptide (CGRP) monoclonal antibodies (mAbs) vs placebo based on the number needed to treat (NNT) and the number needed to harm (NNH) in a blended episodic and chronic migraine population (EM+CM).

**Methods:** The NNT was calculated based on achievement of a ≥50% reduction in mean monthly migraine days (MMDs) from baseline across 12 weeks. A random effects model was used in the meta-analysis of trials to calculate the median ≥50% response rates, NNTs, and credible intervals (CrI) versus placebo at 12 weeks. The NNH was calculated using the proportion of participants reporting a discontinuation due to adverse events (AEs). A cloglog link model was used to estimate median discontinuation rates due to AEs at 12 weeks and calculate NNH and CrI. The base-case analysis included data from core studies of atogepant 60 mg, erenumab 70 mg and 140 mg, galcanezumab 120 mg, eptinezumab 100 mg and 300 mg, and fremanezumab 225 mg and 675 mg. Additional scenario analyses were conducted, including dose-response relationship studies, Asian studies (ie, conducted in China, Japan, and other Asian countries), and dedicated inadequate prior treatment studies (only for NNH).

**Results:** Based on the base-case scenario, the calculated NNT for atogepant 60 mg vs placebo was 4.2, which was comparable with CGRP mAbs in the blended EM+CM population (Table 1). Participants who received atogepant 60 mg or fremanezumab (225 mg or 675 mg) demonstrated lower rates of discontinuation due to AEs compared with those receiving placebo, resulting in negative NNH values (Table 2). Comparable NNT and NNH values were observed for atogepant 60 mg relative to the mAbs across all scenario analyses.

**Conclusion:** Atogepant demonstrated a favorable benefit-risk profile, with NNT and NNH values comparable with those of CGRP mAbs across all scenarios.

**Table 1 (Abstract P029):**
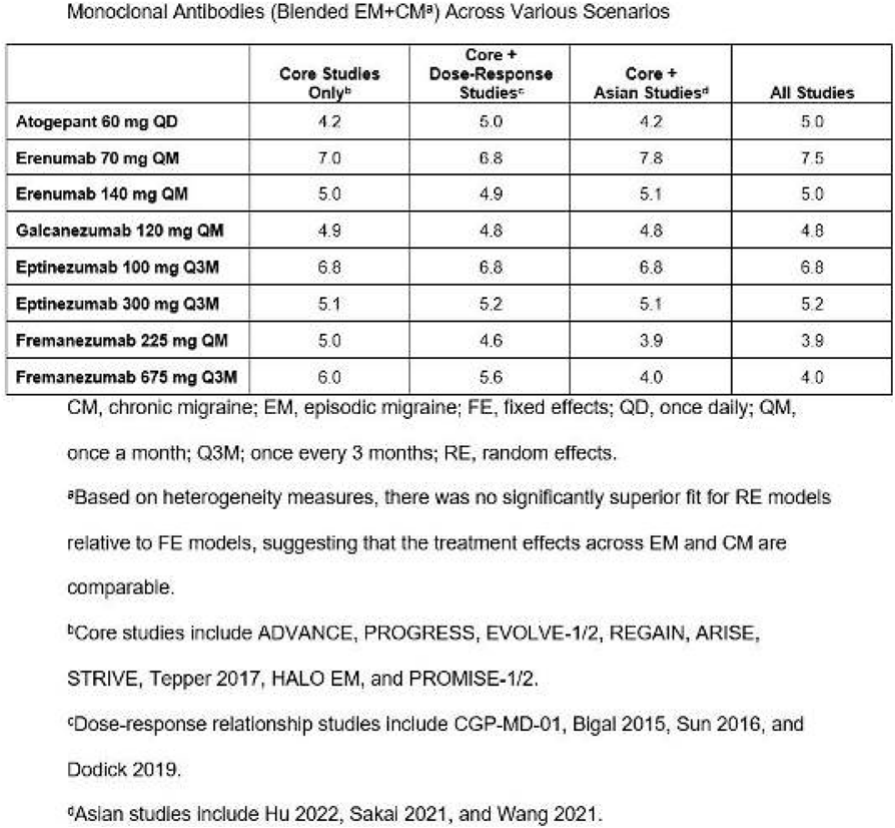
Number Needed to Treat for Atogepant and Calcitonin Gene-Related Peptide Monoclonal Antibodies (Blended EM+CM^a^) Across Various Scenarios

**Table 2 (Abstract P029):**
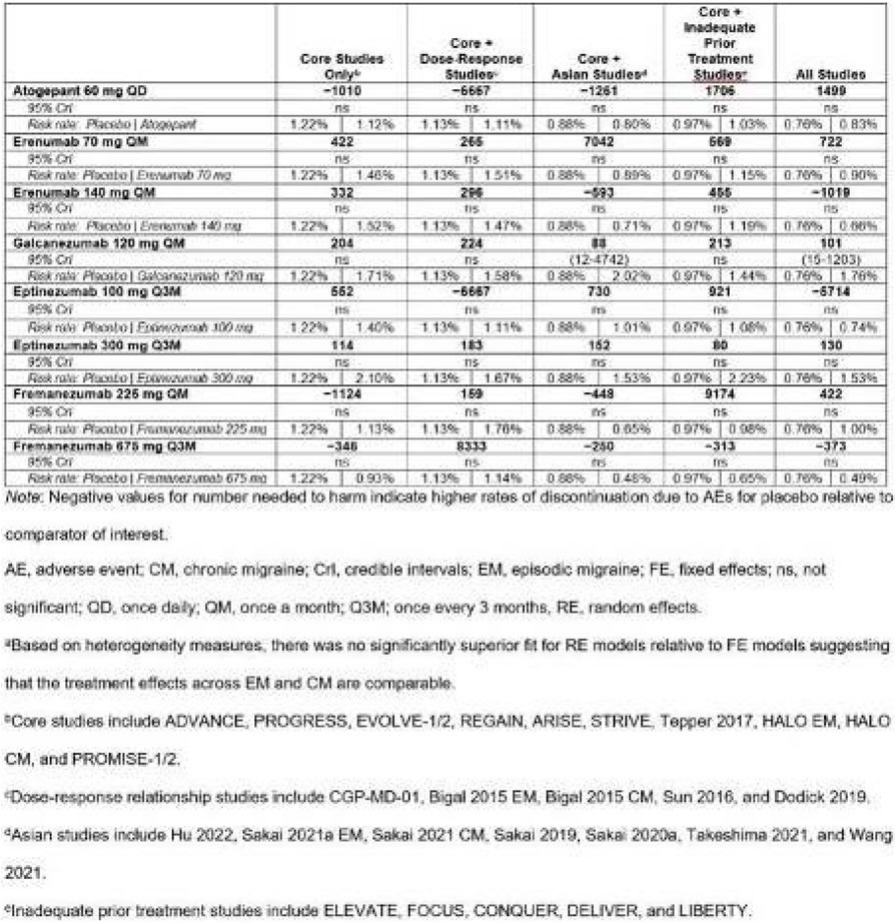
Number Needed to Harm for Atogepant and Calcitonin Gene-Related Peptide Monoclonal Antibodies (Blended EM+CM^a^) Across Various Scenarios

## P030 Rimegepant for the acute treatment of migraine in adults from China

### S. Yu^1^, A. Guo^2^, M. Zhang^1^, Z. Wang^3^, J. Liu^4^, G. Tan^5^, Q. Yang^6^, Y. Liu^7^, Q. Zhao^8^, Z. Lu^9^, J. Atkinson^10^

#### ^1^Chinese PLA General Hospital, Beijing, China; ^2^Yan'an University Xianyang Hospital, Xianyang, China; ^3^Changsha Central Hospital, Changsha, China; ^4^Wuhan Third Hospital, Wuhan, China; ^5^The First Affiliated Hospital of Chongqing Medical University, Chongqing, China; ^6^Shanxi Provincial Hospital, Xi’an, China; ^7^Pfizer, Inc., Beijing, China; ^8^Pfizer, Inc., Chengdu, China; ^9^Pfizer (China) Research and Development Ltd, Shanghai, China; ^10^Pfizer, LTD, Tadworth, United Kingdom

##### **Correspondence:** J. Atkinson

*The Journal of Headache and Pain 2024,* **25(Suppl 1)**: P030

**Objective:** To evaluate the efficacy and safety of rimegepant, an oral small molecule calcitonin gene-related peptide receptor antagonist, for the acute treatment of migraine in adults from China.

**Methods:** This is a subgroup analysis of Chinese patients from a double-blind, randomized, placebo-controlled, phase 3 trial in Chinese and Korean adults with migraine (NCT04574362). Subjects received rimegepant 75 mg or placebo for a single migraine attack of moderate or severe intensity. Coprimary endpoints were pain freedom and freedom from the most bothersome symptom (MBS) at 2 hours post-dose.

**Results:** Rimegepant (*n*=537) was superior to placebo (*n*=537) for the co-primary endpoints of pain freedom (18.2% vs 10.6%, *P*=.0004) and freedom from the MBS (48.0% vs 31.8%, *P*<.0001), as well as all key secondary endpoints (Table). The incidence of treatment-emergent adverse events (AEs) was similar in the rimegepant (15.2%) and placebo (16.4%) groups. No drug-related serious AEs were reported in rimegepant-treated subjects.

**Conclusion:** Rimegepant 75 mg demonstrated rapid and sustained efficacy, with safety and tolerability similar to placebo, for the acute treatment of migraine in adults from China.

**Table 1 (Abstract P030):**
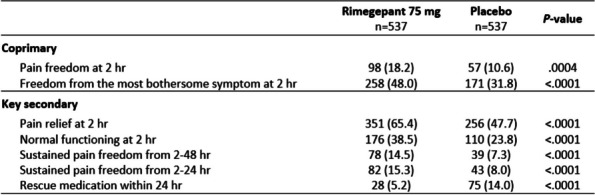
N (%) of subjects meeting coprimary and key secondary endpoints

## P031 Impact of atogepant on migraine day reduction, response rate, and patient-reported outcomes in participants with episodic migraine and comorbid depressive symptoms: subgroup analysis from the ELEVATE trial

### F. Vernieri^1^, P. Gandhi^2^, M. Ashina^3,4^, E. Seng^5^, R. B. Lipton^5^, K. Nagy^6^, Y. Liu^7^, K. Carr^7^, J. Stokes^2^, P. Pozo-Rosich^8,9^

#### ^1^Università Campus Bio-Medico di Roma, Rome, Italy; ^2^AbbVie, Madison, NJ, United States; ^3^Danish Headache Center, Copenhagen University Hospital – Rigshospitalet Glostrup, Department of Neurology, Copenhagen, Denmark; ^4^University of Copenhagen, Department of Clinical Medicine, Copenhagen, Denmark; ^5^Albert Einstein College of Medicine, Bronx, MA, United States; ^6^AbbVie, Budapest, Hungary; ^7^AbbVie, North Chicago, IL, United States; ^8^Vall d’Hebron Hospital & Research Institute, Universitat Autonoma de Barcelona, Barcelona, Spain; ^9^Vall d’Hebron Hospital & Research Institute, Universitat Autonoma de Barcelona, Barcelona, Spain

##### **Correspondence:** F. Vernieri

*The Journal of Headache and Pain 2024,* **25(Suppl 1)**: P031

**Objective:** Evaluate the effect of atogepant on monthly migraine day (MMD) reduction, response rate, and patient-reported outcomes among people with episodic migraine (EM) and depressive symptoms.

**Methods:** This was a subgroup analysis of ELEVATE (NCT04740827), a 12-week, phase 3, multicenter, randomized, double-blind, placebo-controlled trial of atogepant 60 mg QD for preventing EM among participants with prior inadequate response to 2-4 classes of oral preventives. Participants in the off-treatment hypothetical estimand (OTHE) population with depressive symptoms (Patient Health Questionnaire [PHQ-9] score ≥5 [mild/moderate/severe]) were included. Outcomes were change from baseline in 3-month average MMDs; response rates (≥50% and ≥75% reductions in MMDs); and Migraine-Specific Quality of Life questionnaire (MSQ v2.1), Headache Impact Test-6 (HIT-6), and PHQ-9 scores at week 12. Outcomes were analyzed using a mixed-effects model for repeated measures (MMDs, MSQ, and HIT-6), logistic regression (response rates over 12 weeks of treatment), generalized linear mixed model (response rates by 4-week interval), or analysis of covariance (PHQ-9).

**Results:** In the OTHE population (*n*=309), 108 (70%) in the placebo arm and 113 (74%) in the atogepant arm had PHQ-9 ≥5. Among participants with EM and depressive symptoms, relative to placebo, the atogepant group demonstrated greater improvement from baseline in MSQ domain scores (Figure 1); greater reduction from baseline in MMDs (Figure 2); higher response rates (≥50% reduction, odds ratio [OR] [95% CI]: 4.2 [2.3, 7.9], *P*<0.0001; ≥75%, OR [95% CI] = 19.7 [4.6, 84.7], *P*<0.0001 [nominal]); and greater reduction from baseline in HIT-6 total score and PHQ-9 total score (Figure 2). Safety results were consistent with the known safety profile of atogepant.

**Conclusion:** Among people with EM and depressive symptoms, atogepant demonstrated treatment benefits in reduced MMDs and improved response rates and patient-reported outcomes vs placebo.

**Fig. 1 (Abstract P031):**
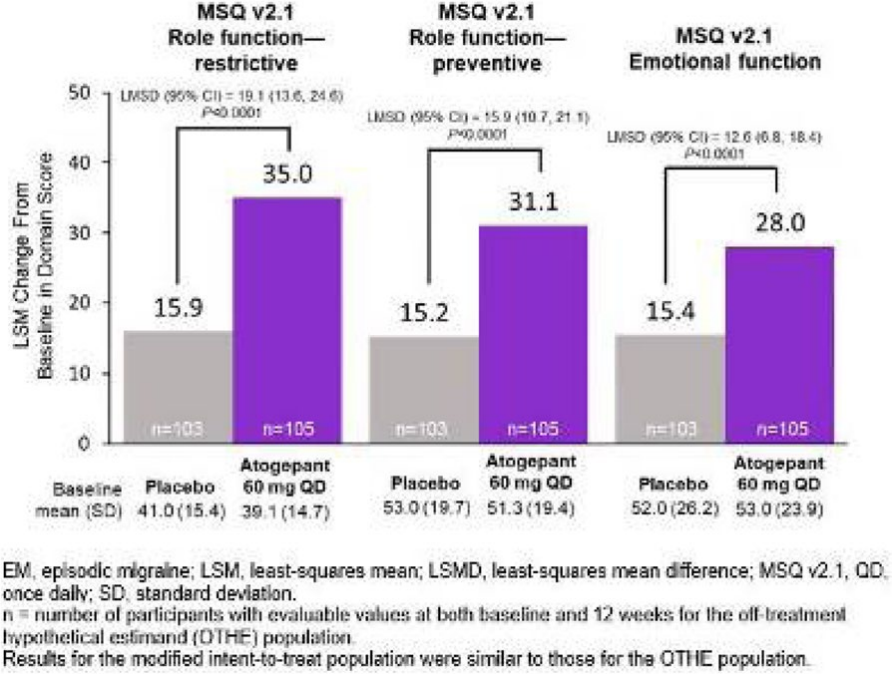
Change from baseline in MSQ v2.1 domain scores at week 12 among participants with EM and depressive symptoms

**Fig. 2 (Abstract P031):**
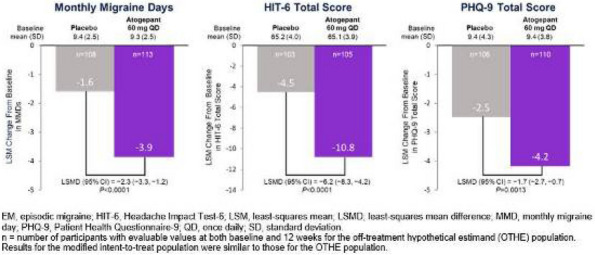
Change from baseline in 3-month average MMDs and in HIT-6 and PHQ-9 total scores at week 12 among participants with EM and depressive symptoms

## P032 Drug-resistant epicrania fugax: responding to onabotulinumtoxinA

### J. Moral Rubio, F. J. Alberola Amores

#### Hospital General Universitario Elche, Neurología, Alicante, Spain

##### **Correspondence:** J. Moral Rubio

*The Journal of Headache and Pain 2024,* **25(Suppl 1)**: P032

**Objective:** Epicrania fugax (EF) is a primary headache consisting of brief paroxysms of pain, lasting 1–10s, that move through different nerve territories of one hemicranium with a linear or zigzag trajectory, although there are some clinical variants.

**Methods:** Preventive therapy with anti-seizure medication such as gabapentin and lamotrigine are most commonly used in patients presenting with frequent and non-remitting attacks. In some cases, greater occipital nerve blockades are used for short-or long-term relief. However, there is still a lack of evidence supporting the efficiency of onabotulinumtoxinA in this group of patients.

**Results:** Here, we report three patients with a paroxismal EF-type pain who meet the criteria for EF of the International Classification of Headache Disorders, 3rd edition, with clear triggers and autonomic ocular signs and who failed multiple preventive treatments, but had a sustained response to onabotulinumtoxinA.

**Conclusion:** Despite this use being absent from the current literature, we think that onabotA could be an excellent treatment for this condition due to its peripheral sensitization inhibition.

## P033 Long-term reductions in acute headache medication use after eptinezumab treatment in patients with prior preventive treatment failures

### A. Gryglas-Dworak^1^, J. Schim^2^, A. Ettrup^3^, L. P. Boserup^3^, M. K. Josiassen^3^, K. Ranc^3^, B. Sperling^3^, M. Ashina^4,5^

#### ^1^MIGRE Polish Migraine Center, Wrocław, Poland: ^2^Neurology Center of Southern California, Carlsbad, CA, United States: ^3^H. Lundbeck A/S, Copenhagen, Denmark: ^4^University of Copenhagen, Department of Neurology, Copenhagen, Denmark: ^5^University of Copenhagen, Department of Clinical Medicine, Copenhagen, Denmark

##### **Correspondence:** A. Gryglas-Dworak

*The Journal of Headache and Pain 2024,* **25(Suppl 1)**: P032

**Objective:** To evaluate long-term reductions in acute headache medication (AHM) use with eptinezumab vs placebo (pbo) in patients with prior preventive migraine treatment failures and medication overuse (MO).

**Methods:** DELIVER (NCT04418765) randomized adults with migraine and 2–4 prior preventive failures to eptinezumab 100mg, 300mg, or pbo every 12 weeks; patients initially given pbo received 100mg or 300mg in the extension period. MO was defined based on modified ICHD-3 criteria and baseline diary reports. Post hoc analysis included change from baseline in AHM days/month (total and class-specific) and percent of patients without MO in the subgroup with MO at baseline.

**Results:** Of 890 patients in the full analysis set (FAS), 438 (49%) had MO at baseline. Baseline AHM days/month were ~11 (FAS) and ~15 (MO). Eptinezumab resulted in greater reductions than pbo in total AHM days/month over Weeks 1–24 (FAS and MO; *p*<0.0001 all comparisons), with triptans showing the largest reduction among classes. Patients switching from pbo to eptinezumab experienced reductions in AHM days/month to similar levels as initial eptinezumab treatment in the FAS (Weeks 1–4, -4.6 [100mg], -4.8 [300mg]; Weeks 25–28: -4.8 [pbo-to-100mg], -5.5 [pbo-to-300mg]) and MO subgroup (-6.5, -6.6, -7.1, and -8.0, respectively). All treatment arms sustained or further reduced AHM use across 18 months of treatment (Weeks 69–72 range: FAS, -4.7 to -5.7; MO, -7.0 to -7.9). In the MO subgroup, the percent of patients with MO at Weeks 1–4 was 31% (100mg), 25% (300mg), 62% (pbo-to-100mg), and 51% (pbo-to-300mg), further decreasing to 12%, 15%, 16%, and 21%, respectively, at Weeks 69–72.

**Conclusion:** Eptinezumab reduced total AHM use more than pbo over Weeks 1–24 with the largest reductions observed for triptans. The robust reductions in AHM use after eptinezumab were sustained or further reduced with up to 18 months of treatment. About 80% of patients with MO at baseline did not meet MO criteria at the end of study.

## P034 Efficacy and safety of minidosing lysergic acid diethylamide (LSD) for chronic cluster headache: protocol for a randomized placebo-controlled study

### J. Jansen^1^, R. Fronczek^2^, R. ter Heine^3^, K. Kramers^1,3^, W. Mulleners^1^

#### ^1^Canisiu-Wilhelmina Hospital, Neurology, Nijmegen, Netherlands; ^2^Leiden University Medical Center, Neurology, Leiden, Netherlands; ^3^Radboud University Medical Center, Nijmegen, Netherlands

##### **Correspondence:** J. Jansen

*The Journal of Headache and Pain 2024,* **25(Suppl 1)**: P034

**Objective:** Chronic cluster headache (cCH) is a severe and debilitating condition that poses significant therapeutic challenges, as current prophylactics are prescribed off-label and are limited in their utility due to associated side effects. Despite treatment, many (notably chronic) cluster headache patients continue suffering headache attacks, necessitating the need for more effective and tolerable treatment options. As a consequence, numerous patients turn to illicit drug use, such as cannabinoids and hallucinogens. In the outpatient clinic, patients frequently attest to a beneficial effect of LSD. However, the evidence for the efficacy of LSD is limited, with the majority of data originating from case reports or uncontrolled and retrospective (internet) surveys. This study aims to provide clinical evidence for the efficacy of LSD in cCH and to evaluate its tolerability and safety.

**Methods:** In this three-week double-blind, placebo-controlled intervention trial a non-hallucinogenic dose of LSD (25μg) will be employed. Treatment will be preceded by a four-week baseline period and followed by a five week post-treatment observation to explore safety and sustainability of effect. A total of 52 patients diagnosed with cCH, according to the ICHD-3 criteria, will be recruited from two headache centres. Patients who meet in- and exclusion criteria will receive oral LSD or matching placebo once every 3 days for 3 weeks.

**Results:** The study's projected outcomes include clinical endpoints, such as change in headache frequency and severity, as well as safety endpoints, the pharmacokinetics and pharmacodynamics of LSD (exposure-response relationship) and cost-effectiveness.

**Conclusion:** If the study findings are positive, LSD should be further studied before use in routine clinical practice. Non-hallucinogenic low-dosed LSD may provide an alternative or adjunctive option for patients who do not respond to or cannot tolerate currently available treatments.

## P035 Efficacy and safety of fremanezumab in patients with migraine and obesity: post-hoc analysis of the phase 3 HALO-LTS and FOCUS clinical trials

### P. Irimia Sieira^1^, B. Torphy^2^, M. Ortega^3^, S. Barash^3^, L. J. Krasenbaum^3^, H. Akcicek^4^, M. Smith^2^, X. Ning^3^, V. Ramirez Campos^3^

#### ^1^Clinica Universidad de Navarra, Neurology Department, Pamplona, Spain; ^2^Chicago Headache Center and Research Institute, Chicago, IL, United States; ^3^Teva Branded Pharmaceutical Products R&D, Inc., West Chester, PA, United States; ^4^Teva Netherlands B.V., Amsterdam, Netherlands

##### **Correspondence:** M. Ortega

*The Journal of Headache and Pain 2024,* **25(Suppl 1)**: P035

**Objective:** Migraine and obesity are both prevalent disabling diseases, with obesity a known migraine comorbidity. A higher body mass index (BMI) is frequently associated with increased migraine prevalence and severity, and an increased number of adverse events (AEs). This subgroup analysis evaluated the efficacy and safety of fremanezumab, a humanized monoclonal antibody targeting the calcitonin gene-related peptide (CGRP) pathway, in patients with migraine and obesity (BMI ≥30 kg/m^2^ [BMI-high]).

**Methods:** This post hoc analysis included data from a subgroup of patients with migraine and obesity from two randomized, placebo-controlled trials: the 12-month Phase 3 HALO-LTS (NCT02638103) study and the 12-week Phase 3b FOCUS (NCT03308968) study. In each study, eligible patients with episodic or chronic migraine (EM, CM) were randomized to receive either monthly (CM: 675/225/225 mg; EM: 225/225/225 mg) or quarterly (675 mg) fremanezumab, or monthly matched placebo during the respective treatment periods (Figure 1). Key efficacy outcomes were change from baseline in mean monthly migraine days (MMD) and in headache days of at least moderate severity.

**Results:** A total of 2437 patients (BMI-high, *n* = 578; BMI <30 kg/m^2^ [BMI-normal], *n* = 1859) received fremanezumab for migraine prevention. BMI-high patients had more self-reported comorbidities vs BMI-normal. Mean MMD at baseline were 13.7 vs 13.6 for BMI-high vs BMI-normal patients, respectively. At Month 6, BMI-high patients had a greater mean change from baseline in MMD vs BMI-normal patients (–6.9 vs –5.9) (Figure 2). In BMI-high vs BMI-normal subgroups, mean headache days were 13.2 vs 13.4 at baseline, and 7.0 vs 7.4 at Month 6, with a mean change from baseline of –6.2 vs –5.6 at Month 6 (**Figure 3**). Similar proportions of patients experienced AEs in the BMI-high (*n* = 462 [80%]) and BMI-normal (*n* = 1459 [78%]) subgroups.

**Conclusion:** This analysis demonstrates that fremanezumab is efficacious and well tolerated over 6 months in patients with both migraine and obesity, consistent with outcomes from other pivotal fremanezumab studies. These data support the use of fremanezumab for migraine prevention in a wide population of patients.

**Fig. 1 (Abstract P035):**
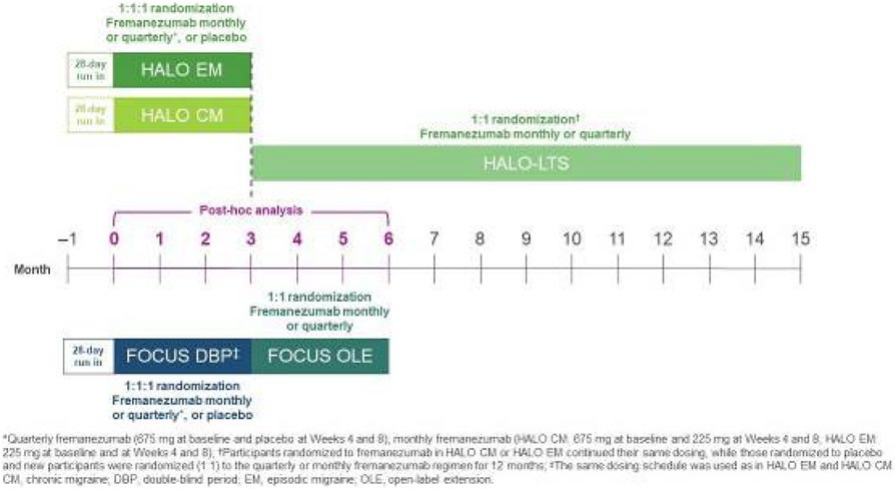
Study design of HALO-LTS and FOCUS clinical trials

**Fig. 2 (Abstract P035):**
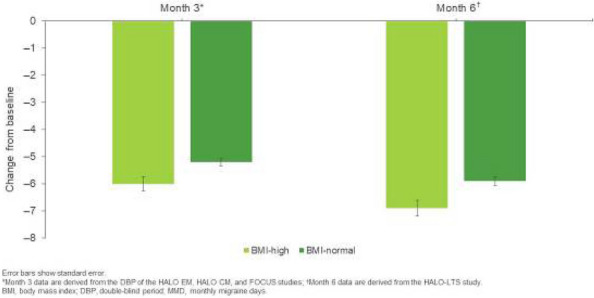
Mean change from baseline in MMD at months 3 and 6 in patients with BMI-high and BMI-normal

**Fig. 3 (Abstract P035):**
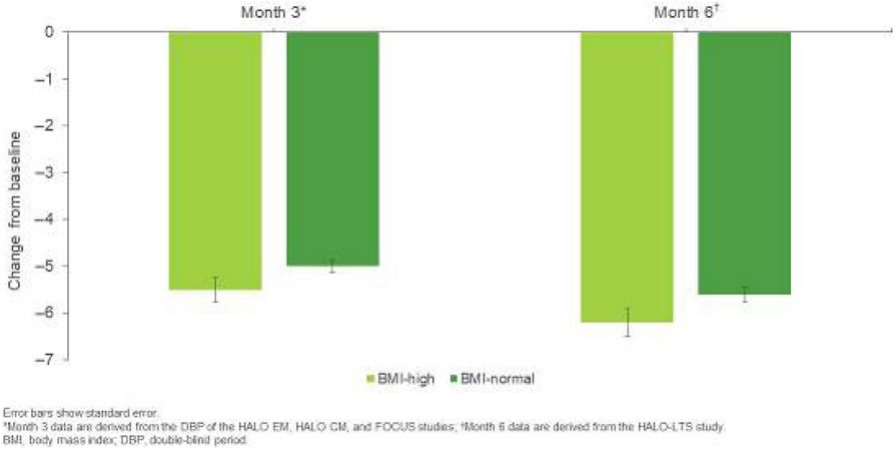
Mean change from baseline in monthly headache days at months 3 and 6 in patients with BMI-high and BMI-normal

## P036 Sustained response to atogepant in individuals with episodic migraine and prior inadequate response to preventive treatment and in individuals with chronic migraine: post-hoc analyses from ELEVATE and PROGRESS

### R. B. Lipton^1^, N. Chalermpalanupap^2^, Y. Tatsuoka^3^, K. Nagy^4^, Y. Liu^5^, K. Carr^5^, P. McAllister^6^, S. J. Nahas^7^, J. M. Trugman^2^, S. Sacco^8^

#### ^1^Albert Einstein College of Medicine, Bronx, MA, United States; ^2^AbbVie, Madison, NJ, United States; ^3^Tatsuoka Neurology Clinic, Kyoto, Japan; ^4^AbbVie, Budapest, Hungary; ^5^AbbVie, North Chicago, IL, United States; ^6^New England Institute for Neurology and Headache, Stamford, CT, United States; ^7^Thomas Jefferson University Hospital, Philadelphia, PA, United States; ^8^University of L'Aquila, L'Aquila, Italy

##### **Correspondence:** S. Sacco

*The Journal of Headache and Pain 2024,* **25(Suppl 1)**: P036

**Objective:** Atogepant is a calcitonin gene-related peptide receptor antagonist approved in the U.S. for the preventive treatment of migraine. This analysis evaluated the proportion of clinical trial participants (in the episodic migraine (EM), ELEVATE and chronic migraine (CM), PROGRESS trials) who experienced sustained response thresholds over the 12 weeks of atogepant treatment following their initial, month 1 response.

**Methods:** In separate phase 3, 12-week, double-blind, randomized, placebo-controlled trials, ELEVATE (NCT04740827), for preventive treatment of EM with inadequate response to previous preventive classes and PROGRESS (NCT03855137), for preventive treatment of CM, participants were randomized to receive atogepant 60 mg once daily or placebo in EM and atogepant 30 mg twice daily, 60 mg once daily or placebo in CM. This post hoc analysis conducted in the Off-treatment Hypothetical Estimand Population (OTHE), focused on the sustained response to atogepant 60 mg once daily. Sustained response was calculated by assessing the proportion of participants with a reduction from baseline in MMDs in month 1, who then experienced at least that threshold of response in months 2 and 3. Initial response thresholds of ≥50%, ≥75%, and 100% reduction (ELEVATE) and ≥30%, ≥50%, ≥75%, and 100% reduction (PROGRESS) from baseline in MMDs were evaluated.

**Results:** Of the 315 (EM) and 778 (CM) participants randomized, the OTHE populations of 309 (EM) and 760 (CM) were included in the analysis. Of participants who experienced an initial ≥50% MMD reduction in month 1, 61.3% (49/80, EM) and 72.6% (69/95, CM) experienced sustained ≥50% response throughout the trial. Of participants who experienced an initial ≥75% MMD reduction in month 1, 52.3% (23/44, EM) and 51.2% (21/41, CM) experienced sustained ≥75% response throughout the trial.

**Conclusion:** In both ELEVATE and PROGRESS trials, most atogepant-treated participants who experienced an initial response in month 1 experienced sustained response throughout the 12-week treatment period.

## P037 Optimizing prophylactic therapy: withdrawal strategies for medication overuse headache in migraine

### A. Uzhakhov^1^, N. Vashchenko^2^, D. Korobkova^2^, J. Azimova^2^, K. Skorobogatykh^2^

#### ^1^Darmed University Clinic, Astana, Kazakhstan; ^2^University Headache Clinic, Moscow, Russian Federation

##### **Correspondence:** A. Uzhakhov

*The Journal of Headache and Pain 2024,* **25(Suppl 1)**: P037

**Objective:** With the advent of monoclonal antibodies to cGRP or to the cGRP receptor in migraine prophylaxis, the typical course of migraine has changed, especially when it is accompanied by medication overuse headache. We asked the question of whether detoxification therapy is necessary for such patients.

**Methods:** The study included 103 patients who came to the University Headache Clinic between April 2021 and May 2022. All patients had migraine and were treated with erenumab. From this group of patients, 41 patients with medication-overuse headache were selected. We considered a 50% reduction in headache days to be a positive effect of erenumab therapy. A reduction in the use of NSAIDs to 14 or fewer days per month or triptans to 9 or fewer days per month was considered a positive outcome for MOH

**Results:** We included 41 patients with chronic migraine. Eight patients suffered from migraine with aura. After 3 months of treatment with erenumab, a positive effect on headache frequency was observed in 28.6% of patients. However, in 24 patients (58.5%) MOH had already resolved. Approximately two-thirds of patients with MOH (71.4%) did not respond to treatment with anti-CGRP monoclonal antibodies after 3 months of therapy (the mean number of headache days per month was 16.6). Eleven of them were switched to fremanezumab and after 3 months, 7 patients had a positive effect on headache frequency and MOH. There was no significant difference in baseline headache frequency between erenumab responders (22.6 days per month) and nonresponders (25.3 days per month).

**Conclusion:** Based on our clinic experience, anti-CGRP monoclonal antibody monotherapy can be started in patients with MOH without withdrawing triptans or NSAIDs. Detoxification therapy should be added if there is no significant benefit after 3 months of anti-CGRP monoclonal antibody therapy. Predictors of response to MH therapy without detoxification remain to be clarified in future studies.

## P038 Cost-effectiveness of rimegepant oral lyophilisate compared to best supportive care for the acute treatment of migraine in the UK

### K. Johnston^1^, L. Powell^1^, E. Popoff^1^, G. L'Italien^2^, R. Pawinski^3^, A. Ahern^3^, S. Large^3^, T. Tran^4^, A. Jenkins^3^

#### ^1^Broadstreet Health Economics and Outcomes Research, Vancouver, Canada; ^2^Biohaven Pharmaceuticals Inc., New Haven, CT, United States; ^3^Pfizer Ltd., Tadworth, United Kingdom; ^4^Pfizer Canada, Kirkland, Canada

##### **Correspondence:** A. Jenkins

*The Journal of Headache and Pain 2024,* **25(Suppl 1)**: P038

**Objective:** Rimegepant 75 mg, an oral lyophilisate calcitonin gene-related peptide antagonist, is the first acute migraine medication approved in over 20-years, and the first and only for both acute and preventative treatment. The aim of this analysis was to assess the cost-effectiveness of rimegepant compared with best supportive care (BSC) in the UK.

**Methods:** A de novo economic model was developed to estimate incremental costs and quality-adjusted life years (QALYs), structured as a hybrid decision tree followed by Markov model. The target population comprised adults who had failed ≥2 triptans. Patients received rimegepant or BSC for a migraine attack and were assessed for response (pain relief at 2-hours). Responders and non-responders followed different pain trajectories over 48-hour cycles. Non-responders discontinued treatment while responders continued treatment for subsequent attacks, with a proportion discontinuing over time. Data sources included a post-hoc pooled analysis of three Phase 3 acute rimegepant trials (NCT03235479, NCT03237845, NCT03461757) and a 52-week safety study, which informed discontinuation rates and reduced monthly migraine days with long-term rimegepant acute treatment (NCT03266588). Areas of uncertainty were minimised by clinical independent external expert consensus. The analysis was conducted from the UK National Health Service perspective over a 20-year time horizon.

**Results:** Rimegepant had higher incremental QALYs and higher incremental costs compared to BSC and was cost-effective at a willingness-to-pay threshold of £30,000/QALY. Improved QALYs for rimegepant were a result of less time spent with moderate or severe migraine pain. Base case results were robust to deterministic sensitivity and scenario analyses.

**Conclusion:** This study highlights the economic value of rimegepant which was found to be cost-effective for acute treatment of migraine in adults with inadequate response, intolerance, or contraindication to triptans in the UK.

## P039 Effectiveness and tolerability of monoclonal antibodies targeting CGRP pathway for migraine prevention: Real-world data from the Migraine Registry (ReMig) in the Czech Republic

### T. Nežádal^1^, T. Doležal^2^, D. Pejřilová^2^, B. Turková^2^, J. Marková^3^, A. Bártková^4^, L. Klečka^5^, C. Z. ReMig study group^2^

#### ^1^Military University Hospital , Prague, Czech Republic; ^2^Value Outcomes, Prague, Czech Republic; ^3^University Thomayer Hospital, Department of Neurology, Prague, Czech Republic; ^4^Palacký University Hospital, Department of Neurology, Olomouc, Czech Republic; ^5^Municipal Hospital, Department of Neurology, Ostrava, Czech Republic

##### **Correspondence:** T. Nežádal

*The Journal of Headache and Pain 2024,* **25(Suppl 1)**: P039

**Objective:** First evaluation and cohort characteristics of participants enrolled in the Czech Registry of patients with Migraine on biological treatment (ReMig).

**Methods:** A prospective evaluation of patients enrolled in the ReMig: detailed demographics, past prophylactic therapy, monthly migraine days (MMD), days of acute migraine medication, treatment persistence and safety.

**Results:** A total of 2 269 patients were analysed. Most of the patients were women (87.6%), the mean age was 46.5 years, mean age at diagnosis was 19.1 years. Mean BMI was 25.2 kg/m^2^.

Mean duration from diagnosis to first anti CGRP therapy was 26.3 years. Positive family history of migraine was recorded in 64.6% of patients. A total of 28.1% of patients had chronic migraine (CM), 71.9% episodic migraine (EM).

Most of the patients (60.1%) did not remember any migraine triggering event, 12.5% reported stress and 11.4% menarche. 57.2% of the patients had at least one comorbidity.

Before biological treatment, 98.9% of patients were treated with antiseizure medication, 53.4% with calcium channel blockers and 45.5% with antidepressants.

Out of 2 346 treatment series were 41.6% erenumab, 36.4% fremanezumab, 21.3% galcanezumab and 0.6% eptinezumab. Mean duration of therapy was 12 months and only 10.8% of treatment series were discontinued.

Mean MMD decreased from 12.0 to 2.9 after 12 months of treatment, from 17.6 to 4.1 and from 9.8 to 2.6 in patients with CM and EM, respectively.

A total of 98.0% of patients required acute medication for migraine at the start of treatment. The mean number of days of acute treatment decreased after 12 months from 8.2 to 2.1, from 3.8 to 1.1 and from 1.7 to 0.4 for triptans, NSAIDs and analgesics, respectively.

Medication overuse headache decreased from 37.6% to 0.7%.

A total of 123 adverse events were recorded, 2 of which were serious.

**Conclusion:** Collecting long-term data on the effectiveness and tolerability of migraine biological treatment can contribute to its further correctly selected and safe administration.

## P040 Fremanezumab effectiveness and tolerability in clinical routine: interim real-world-data of the observational FINESSE study

### A. Straube^1^, G. Broessner^2^, C. Gaul^3^, X. Hamann^4^, T. Kraya^5,6^, L. Neeb^7,8^

#### ^1^University Hospital LMU Munich, Department of Neurology, Munich, Germany; ^2^Innsbruck Medical University, Department of Neurology, Innsbruck, Austria; ^3^Headache Center Frankfurt, Frankfurt a. M., Germany; ^4^Teva GmbH, Ulm, Germany; ^5^Hospital Sankt Georg Leipzig gGmbH, Headache Center Halle, Department of Neurology, Leipzig, Germany; ^6^Headache Center Halle, University Hospital Halle, Department of Neurology, Halle (Saale), Germany; ^7^Helios Global Health, Berlin, Germany; ^8^Charité – Universitätsmedizin Berlin, Department of Neurology, Berlin, Germany

##### **Correspondence:** A. Straube

*The Journal of Headache and Pain 2024,* **25(Suppl 1)**: P040

**Objective:** To evaluate effectiveness and tolerability of fremanezumab administered in migraine patients as part of their routine disease management.

**Methods:** FINESSE is an ongoing prospective, non-interventional study in adults with episodic or chronic migraine (EM, CM). Observation period: 24 months. Primary endpoint: proportion of patients reaching ≥ 50% reduction in the average number of monthly migraine days (MMD) during the 6-month period after the first dose of fremanezumab. Further measures: monthly average number of migraine days, MIDAS (Migraine Disability Assessment), HIT-6 (6-Item Headache Impact Test), acute medication use. Adverse events as reported in routine clinical practice.

**Results:** Of 826 patients (intention-to-treat analysis), 444 (53.8%) achieved a MMD reduction of ≥ 50% in the 6 months post-initial fremanezumab dose (EM: 58.4%, CM: 47.4%). Table 1: Effectiveness data for months 6 and 12.

Of 1076 patients (safety analysis), 523 (48.6%) reported any adverse event. Injection site reactions: 186 (17.3%), COVID-19: 170 (15.8%), drug ineffective: 130 (12.1%), constipation: 31 (2.9%).

**Conclusion:** 53.8% of patients achieved the primary endpoint. Current results of the FINESSE study substantiate continuous MMD reduction, and sustained decrease in disability and acute medication use. Presented real-world-data on tolerability are in line with the expected favourable safety profile of fremanezumab demonstrated in the pivotal studies.

**Table 1 (Abstract P040):** Effectiveness (missing data omitted)

≥ 50% MMD reduction over 6 months (*N*=826)		444 (53.8%)
EM (*N*=474)		277 (58.4%)
CM (*N*=352)		167 (47.4%)
MMD	Baseline (*N*=926)	12.6
	Month 6 (*N*=712)	5.3
	Month 12 (*N*=484)	5.0
MIDAS	Baseline (*N*=545)	75.2
	Month 6 (*N*=500)	32.0
	Month 12 (*N*=296)	27.3
HIT-6	Baseline (*N*=581)	65.9
	Month 6 (*N*=510)	57.2
	Month 12 (*N*=303)	57.0
Acute medication use (days)	Baseline (*N*=901)	9.6
	Month 6 (*N*=712)	3.7
	Month 12 (*N*=484)	3.7

## P041 A prospective, observational study to evaluate the long-term safety, including cardiovascular safety, of fremanezumab in patients with migraine in routine clinical practice

### N. Kahan^1^, L. J. Krasenbaum^2^, D. Braverman^1^, S. Barash^2^, S. Colilla^2^, K. Jennissen^3^, R. Nellailingam^4^, C. Rainville^2^, V. Ramirez Campos^2^, J. Frain^2^, H. Akcicek^5^, P. JM^6^

#### ^1^Teva Branded Pharmaceutical Industries Ltd., Netanya, Israel; ^2^Teva Branded Pharmaceutical Products R&D, Inc., West Chester, PA, United States; ^3^Teva Pharmaceuticals International GmbH, Rapperswil-Jona, Switzerland; ^4^Teva UK Ltd., Harlow, United Kingdom; ^5^Teva Netherlands B.V., Amsterdam, Netherlands; ^6^Teva Pharmaceutical Industries Ltd., Karnataka, India

##### **Correspondence:** H. Akcicek

*The Journal of Headache and Pain 2024,* **25(Suppl 1)**: P041

**Objective:** This post-authorization safety study was developed to evaluate the long-term safety, including cardiovascular (CV) safety, of fremanezumab in real-world clinical practice.

**Methods:** In this controlled prospective cohort study in the US, Spain, and Canada, patients with migraine aged ≥18 years were grouped into three cohorts based on their preventive migraine medication: fremanezumab, other calcitonin gene-related peptide (CGRP) pathway medications (that target CGRP or its receptor), and other migraine preventive medications. Each cohort was divided into two sub-populations: CV compromised (patients with a history or current diagnosis of major CV disease and/or hypertension [HTN]) and non-CV compromised patients. The aim was to enroll a total of 6000 patients, 725 non-CV compromised and 1275 CV compromised in each cohort. Follow-up was planned for at least 3 years. Concomitant medications, comorbidities, and all adverse events (AEs) were electronically recorded. To assess worsening of pre-existing HTN, blood pressure (BP) values measured during office visits were documented. Patients were consented and enrolled at the physician visit when the cohort drug was initially prescribed. Rates of AEs were calculated for all cohorts stratified by the CV subpopulations.

**Results:** Over a period of 30 months, only 1077 patients were enrolled. The study was terminated due to low enrollment rates, primarily of CV compromised patients (*N* = 342). Mean duration of follow-up in the fremanezumab cohort (*N* = 374) was 396.9 days (minimum; maximum: 23; 857). No new safety trends or previously undocumented AEs were observed. Routine BP measurement was generally not part of standard of care which impeded evaluation of the BP outcomes.

**Conclusion:** No new safety findings in patients treated with fremanezumab were detected. With the current interest in the effects of CGRP pathway targeted medications on BP, measurements of BP should be encouraged as part of a routine migraine office visit.

## P042 Efficacy of fremanezumab in reducing Monthly Migraine Days (MMD) and depression severity in patients from european countries with Migraine and Major Depressive Disorder (MDD)

### C. Tassorelli^1,2^, D. D. Mitsikostas^3^, P. Barbanti^4,5^, H. Akcicek^6^, L. J. Krasenbaum^7^, V. Ramirez Campos^7^

#### ^1^University of Pavia, Brain & Behavioral Science, Pavia, Italy; ^2^IRCCS C. Mondino Foundation, Pavia, Italy; ^3^National and Kapodistrian University of Athens, Department of First Neurology, Aeginition Hospital, Athens, Greece; ^4^IRCCS San Raffaele Roma, Headache & Pain Unit, Rome, Italy; ^5^San Raffaele University, Rome, Italy; ^6^Teva Netherlands B.V., Amsterdam, Netherlands; ^7^Teva Branded Pharmaceutical Products R&D, Inc., West Chester, PA, United States

##### **Correspondence:** C. Tassorelli

*The Journal of Headache and Pain 2024,* **25(Suppl 1)**: P042

**Objective:** This post hoc analysis of the UNITE study evaluated the efficacy of fremanezumab, a humanized monoclonal antibody that selectively targets calcitonin gene-related peptide, in reducing MMD and the severity of depression in patients enrolled in European countries with migraine and MDD.

**Methods:** UNITE (NCT04041284) was a multicenter, randomized, placebo-controlled, Phase 4 study with a 12-week treatment period, followed by a 12-week open-label extension (OLE), conducted in 12 countries. Patients with ≥12 months of diagnosed migraine, 12-month history of MDD (DSM-V criteria) prior to screening, and active symptoms of depression (Patient Health Questionnaire-9 score ≥10) were randomized (1:1) to monthly fremanezumab (225 mg) or matched placebo. A single concomitant medication for depression was permitted if the dose was stable ≥8 weeks prior to screening, with no anticipated changes. Key endpoints were: mean change from baseline in MMD during the 12-week treatment period and mean change from baseline in symptoms of depression as measured by the 17-item Hamilton Depression Rating Scale (HAMD-17), where a reduction from baseline of ≥50% or by 3–8 points is considered clinically meaningful.

**Results:** Of the 353 total patients randomized, 226 were enrolled in European countries (fremanezumab, *n* = 114; placebo, *n* = 112). Mean change from baseline in MMD during the double-blind period was –4.5 for fremanezumab and –2.3 for placebo (Figure 1). The mean change from baseline in HAMD-17 score was –3.6 vs –3.5 at Week 4, –6.0 vs –4.7 at Week 8, and –6.3 vs –5.4 at Week 12 for fremanezumab and placebo, respectively (Figure 2).

**Conclusion:** Fremanezumab was associated with reductions in MMD and HAMD-17 scores at Week 12. These data suggest that reduced severity of depression is an additional potential benefit of using fremanezumab to reduce MMD in European patients with migraine and MDD.

**Fig. 1 (Abstract P042):**
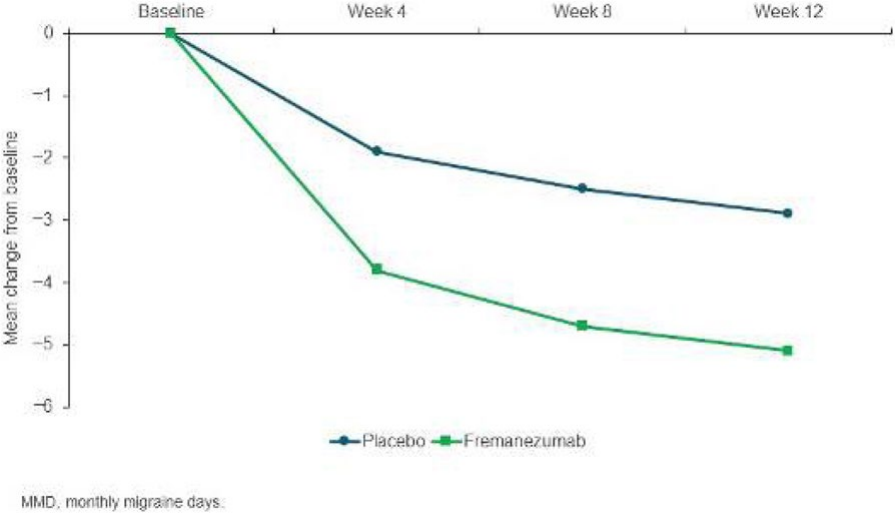
Change from baseline in average MMD in the double-blind period in European patients

**Fig. 2 (Abstract P042):**
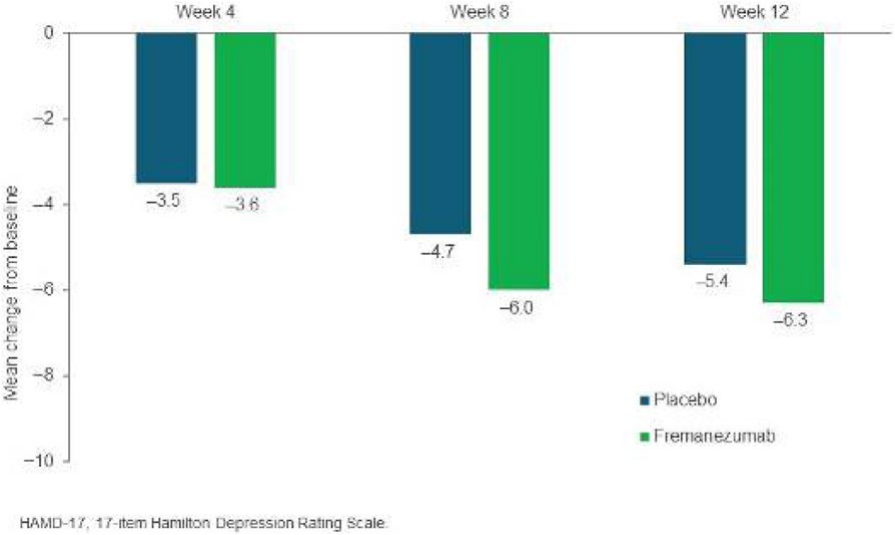
Reduction from baseline in HAMD-17 scores in the double-blind period in European patients

## P043 The possible role of onabotulinum toxin A in the preventive treatment of chronic migraine during pregnancy

### C. Fernandes^1^, J. M. Alves^1^, M. Coelho^1^, I. Costa^1^, F. Magalhães^1^, H. Gens^1^, I. Luzeiro^1,2^

#### ^1^Hospitalar and University Center of Coimbra, Neurology, Coimbra, Portugal; ^2^Coimbra Health School, Coimbra, Portugal

##### **Correspondence:** C. Fernandes

*The Journal of Headache and Pain 2024,* **25(Suppl 1)**: P043

**Objective:** Chronic Migraine (CM) is a condition that predominantly affects women of childbearing age. However, the safety regarding the application of onabotulinum toxin A (OnaBotA) during pregnancy in the preventive treatment of CM is still not well studied. Our objective was to assess the impact of using OnaBotA during pregnancy, particularly in the early stages.

**Methods:** Description of 3 clinical cases and literature review according to Haynes's "5 S" model.

**Results:** The first case, a 38-year-old female diagnosed with migraine with aura (MA) underwent OnaBotA treatment in the 5th week of pregnancy. During pregnancy, she had a diagnosis of gestational diabetes (GD) and in the end a cesarean delivery due to induction failure. Second, a 39-year-old woman with migraine without aura. She underwent eight OnaBotA treatments before pregnancy, with the last treatment in the first week of pregnancy. No complications during pregnancy. Third, a 32-year-old woman with MA was submitted to OnaBotA treatment 8 weeks before conception. During pregnancy with the diagnosis of GD. In the three patients, a total dose of 195U was administered, and during pregnancy, it was necessary to block the greater and lesser occipital nerves due to the worsening of the number/intensity of the headache attacks. No fetal malformations were detected. All pregnancies resulted in full-term and normal-weight newborns.

There are no systematic reviews or meta-analyses published on the subject. It was possible to find results at the "Studies" level with a case study and two retrospective studies, one of which included 45 patients who did not present worse obstetric outcomes.

**Conclusion:** In these cases, the exposure to OnaBotA in the pre-conception period or first trimester of pregnancy did not show malformations or relevant obstetric complications. We concluded that scientific evidence is scarce on this subject, but previously published studies do not seem to associate OnaBotA with significant secondary effects.

*Disclosure statement:* Informed consent to publish this case study and its potentially identifiable information of the patients was obtained from the individuals involved. The patients gave explicit permission for the publication of this case report, including any relevant clinical details.

## P044 The impact in migraine postdrome phase of monoclonal antibody therapy against CGRP

### C. Fernandes^1^, J. M. Alves^1^, I. Costa^1^, H. Gens^1^, I. Luzeiro^1,2^

#### ^1^Hospitalar and University Center of Coimbra, Neurology, Coimbra, Portugal; ^2^Coimbra Health School, Coimbra, Portugal

##### **Correspondence:** C. Fernandes

*The Journal of Headache and Pain 2024,* **25(Suppl 1)**: P044

**Objective:** The migraine attack is typically divided into four nonobligatory phases, which include the postdrome phase. Although common and disabling, the postdrome corresponds to the final phase of migraine and is the least studied and most poorly characterized phase. Our study aimed to determine the prevalence of the postdrome, characterize its clinical manifestations, evaluate the impact of these symptoms on patients' quality of life, and investigate the effects of monoclonal antibody therapy against CGRP (mAbs) in the population studied.

**Methods:** We conducted a cross-sectional study of migraine patients treated with mAbs between January and June 2023. Patients completed a questionnaire with demographic and clinical information about postdrome symptoms and mAbs response.

**Results:** Nineteen patients with a median age of 49.0 ± 11.8 [25-75] years were surveyed, 68.4% were women, 84.2% were diagnosed with chronic migraine and 94.7% reported at least one postdrome symptom. The most frequent symptoms were sensitivity to light (77.8%), sensitivity to sound (72.2%) and fatigue (61.1%). 83.3% of patients reported an impact on their daily activities. Abortive medication drugs seem to influence the postdrome phase's duration in 77.8% of patients. The frequency of migraine attacks does not seem to influence the presence of the postdrome phase (*p*=0.526). An improvement after starting mAbs therapy was noticed by 88.9% of the patients, nine (52.9%) with improvement in frequency, and fourteen (82.4%) in the duration of the postdrome phase. At this point, only 47.1% of patients reported a negative impact on daily living activities.

**Conclusion:** We conclude that postdrome symptoms are very frequent and light and sound sensitivity were the most reported symptoms. We did not find any factor that can be correlated with the postdrome phase. The mAbs therapy not only improves headache attacks but also seems to influence another attack phase, such as the prodromic period.

## P045 Effect of atogepant on the migraine disability assessment, work productivity and activity impairment questionnaire, and patient global impression of change: results from the ELEVATE trial

### S. Christie^1^, P. Gandhi^2^, F. Vernieri^3^, D. Holle-Lee^4^, K. Nagy^5^, L. Luo^2^, J. Stokes^2^, C. Tassorelli^6^

#### ^1^University of Ottawa, Neurology, Ottawa, ON, Canada; ^2^AbbVie, Madison, NJ, United States; ^3^Università Campus Bio-Medico di Roma, Rome, Italy; ^4^West German Headache and Vertigo Center Essen, Department of Neurology, Essen, Germany; ^5^AbbVie, Budapest, Hungary; ^6^Headache Science & Neurorehabilitation Centre, C. Mondino Foundation and University of Pavia, Pavia, Italy

##### **Correspondence:** P. Gandhi

*The Journal of Headache and Pain 2024,* **25(Suppl 1)**: P045

**Objective:** Evaluate atogepant on measures of disability, work productivity, and patient-rated overall change in people with episodic migraine (EM) previously experiencing inadequate response to oral migraine preventive medications (OMPMs).

**Methods:** ELEVATE (NCT04740827) was a 12-week, phase 3, multicenter, randomized, double-blind, placebo-controlled trial of atogepant 60 mg once daily for preventing EM among participants with prior inadequate response to 2–4 OMPM classes. Outcomes included change from baseline on the Migraine Disability Assessment (MIDAS) and Work Productivity and Activity Impairment Questionnaire: Migraine v2.0 (WPAI), and the Patient Global Impression of Change (PGIC) responder rate (ie, participants with responses of "much better" or "very much better"). These were analyzed (off-treatment hypothetical estimand population) using analysis of covariance (MIDAS), a restricted maximum likelihood–based mixed model for repeated measures (WPAI), or logistic regression (PGIC).

**Results:** Compared to placebo, atogepant demonstrated greater reduction from baseline in MIDAS total (least-squares mean difference [LSMD] [95% CI]: −20.2 [−31.4, −8.9], nominal *P*=0.0005), absenteeism (LSMD [95% CI]: −9.1 [−16.1, −2.1], *P*=0.0110), and presenteeism scores (LSMD [95% CI]: −11.0 [−16.0, −6.0], *P*<0.0001) at week 12, and greater reduction from baseline in WPAI presenteeism, overall impairment, and activity impairment at weeks 4, 8, and 12 (Figure). PGIC responder rate at week 12 was greater for atogepant vs placebo (70% vs 31%; odds ratio [95% CI]: 5.3 [3.2, 8.7], nominal *P*<0.0001). Overall safety results were consistent with the known safety profile of atogepant.

**Conclusion:** Atogepant demonstrated nominally significant reduction of migraine-related disability, including absenteeism and presenteeism; improved work productivity; reduced activity impairment; and greater global impression of change among people with EM and prior inadequate response to 2–4 classes of OMPMs.

**Fig. 1 (Abstract P045):**
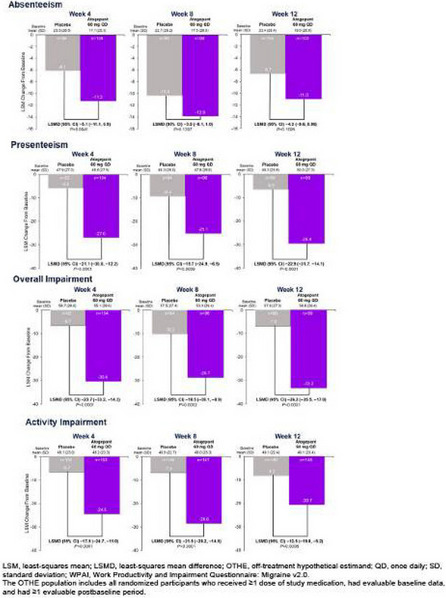
Change form baseline in WPAI: migraine v2.0 scores among participants with episodic migraine (OTHE Population)

## P046 Real-life study of migraine burden modification with Botulinum toxin type A introduction in a group I hospital centre in Portugal

### A. S. Pereira^1^, I. Macedo^2^, I. Alves^1^

#### ^1^Centro Hospitalar Tâmega e Sousa, E.P.E., Neurology, Penafiel, Portugal; ^2^USF Hygeia, ACeS Tâmega III - Vale do Sousa Norte, Vila Cova da Lixa, Portugal

##### **Correspondence:** A. S. Pereira

*The Journal of Headache and Pain 2024,* **25(Suppl 1)**: P046

**Objective:** Migraine is a common and disabling health problem, representing high direct and indirect costs. Botulinum toxin type A (BTX-A) is one of the prophylactic therapies indicated in chronic migraine (CM). The present studyevaluates the impact of initiating BTX-A intervention in patients with chronic migraine or non-controlled high frequency episodic migraine in a group I Hospital Centre in Portugal.

**Methods:** A retrospective longitudinal study was performed and included patients with migraine (according to diagnostic criteria published in ICHD-3), age ≥18-years-old, follow-up data available for the first 6 months of Interventional Neurology outpatient clinic in a group I Hospital Centre in Portugal.

**Results:** The 26 patients analysed presented a mean age of 43,1-years-old, a female prevalence of 96% and family history of migraine in 54%. A mean failure number of 4 preventive drugs was reported. Topiramate, propranolol and amitriptyline were the most previously tried drugs and valproate wasn"t picked in almost all patients due to fertile age. The evaluation 3 months after the first treatment session with BTX-A was associated with a reduction of 29,8% in days per month with headache and a mean reduction of 2.7 points in HIT-6 score, (*P*=0,000004 and *P*=0,01 respectively). At 6 months evaluation, after two treatments sessions, the total reduction compared to the baseline were 40,9% (*P*= 0,00015) in days per month with headache and a mean reduction of 6 points in HIT-6 score (*P*=0,34). Five patients wanted to discontinue the treatment after no improvement with one or two BTX-A sessions.

**Conclusion:** In our previous non-responsive migraine population, two sessions of BTX-A were associated to a gradual improvement of the frequency and burden of headache. Despite the size of the population, our data are as close as possible to reality and suggest future investigation in our setting to analyse the extension and adaptation of our population to new opportunities of treatment.

## P047 Erenumab 70mg every 15 days: avoiding the "wearing-off" effect in the treatment of pharmacorresistent chronic migraine

### I. M. Lucas Requena, F. J. Alberola Amores

#### Hospital General Universitario de Elche, Neurología, Elche, Spain

##### **Correspondence:** I. M. Lucas Requena

*The Journal of Headache and Pain 2024,* **25(Suppl 1)**: P047

**Objective:** The launch of monoclonal antibodies directed against CGRP has been a revolution in the management of migraine, being the first specific preventive treatment for this disease. Throughout the experience with the use of these drugs, there has been evidence of a decrease in efficacy towards the dosing interval, known as the "wearing off" effect. Only three observational studies have been published with a wearing-off effect range of 9-35%. This effect seems to be greater in patients with chronic migraine than in episodic migraine and also in those patients treated with Erenumab. In a study of 145 patients treated with Erenumab, up to 40% of the total experienced wearing-off effect between injections. The underlying mechanism is not clear, although it is thought that it may be related to its pharmacokinetic characteristics, associating a decrease in serum drug levels at the end of the monthly cycle.

**Methods:** We present the case of a 66-year-old woman under follow-up for chronic migraine refractory to multiple preventive treatments, including botulinum toxin. Treatment was initiated with Erenumab 70mg, achieving a good initial response that decreased progressively. Afterwards, the dose was increased up to 140mg every 28 days, with no clinical response. The patient was switched to Galcanezumab with good clinical response but progressively worsened, which was repeated in the same way with Fremanezumab.

**Results:** Given the suspicion of a wearing-off effect probably due to rapid proteolysis in this patient, it was decided to start a regimen of Enerumab 70 mg every 15 days as compassionate use with the patient's consent, in order to achieve constant minimum plasma levels. With this regimen an optimal control of the disease is achieved, presenting a single episode per month, being this response constant and maintained in a year of follow-up, without presenting adverse effects.

**Conclusion:** Despite the revolution that anti-CGRP treatment has brought about, we are still facing patients with a partial effect or "wearing off" effect that we don't know why it occurs, and the role that a readjustment in the treatment dosing intervals could play.

*Disclosure statement:* Informed consent to publish this case study and its potentially identifiable information of the patients was obtained from the individuals involved. The patients gave explicit permission for the publication of this case report, including any relevant clinical details.

## P048 Patient responses to atogepant 60 mg once daily: a p*ost hoc* analysis of the ADVANCE trial

### J. J. Y. Ong^1^, Y. Liu^2^, J. H. Smith^2^, K. Carr^2^, P. J. Goadsby^3,4^

#### ^1^National University Hospital, Division of Neurology, Department of Medicine, & Yong Loo Lin School of Medicine, Singapore, Singapore; ^2^AbbVie, North Chicago, IL, United States; ^3^King's College London, London, United Kingdom; ^4^University of California, Los Angeles, CA, United States

##### **Correspondence:** P. J. Goadsby

*The Journal of Headache and Pain 2024,* **25(Suppl 1)**: P048

**Objective:** To evaluate individual responses to atogepant among people with episodic migraine (EM).

**Methods:** ADVANCE was a 12-week, double-blind, randomized, phase 3 trial evaluating atogepant 10 mg, 30 mg, and 60 mg once daily compared with placebo for the preventive treatment of EM. In this *post hoc* analysis, changes from baseline in monthly migraine days (MMDs) and monthly headache days (MHDs) at weeks 9-12 were assessed for each participant. The proportions of participants who reported a given change in MMDs and MHDs at weeks 9-12 were calculated, as well as those with ≥15 MHDs at weeks 9-12. Categories were defined as an increase in MMDs or MHDs, a reduction of ≥0 to <4 MMDs or MHDs, and a reduction of ≥4 MMDs or MHDs.

**Results:** A total of 436 participants (placebo, *n*=214; atogepant 60 mg, *n*=222) in the modified intent-to-treat population from ADVANCE were included. Mean (SD) MMDs and MHDs at baseline were 7.8 (2.3) and 9.0 (2.6) for participants randomized to atogepant 60 mg and 7.5 (2.4) and 8.4 (2.6) for participants randomized to placebo, respectively. An increase in MMDs at weeks 9-12 compared with baseline occurred in 7.2% vs 16.8% of atogepant- vs placebo-treated participants (Table). A reduction of ≥4 MMDs from baseline to weeks 9-12 occurred in 63.1% vs 39.3% of atogepant- vs placebo-treated participants. Additionally, an increase in MHDs at weeks 9-12 occurred in 9.2% vs 14.8% of atogepant- vs placebo-treated participants. A reduction of ≥4 MHDs at weeks 9-12 occurred in 64.1% vs 40.3% of atogepant- vs placebo-treated participants. Three participants (1.5%) in each arm had ≥15 MHDs at weeks 9-12.

**Conclusion:** Higher proportions of participants with EM experienced an increase in MMDs and MHDs on placebo compared with atogepant at weeks 9-12, with no increased risk of medication overuse headache. These results further demonstrate the efficacy of atogepant 60 mg once daily, on a per patient basis, for the preventive treatment of EM.

**Table 1 (Abstract P048):**
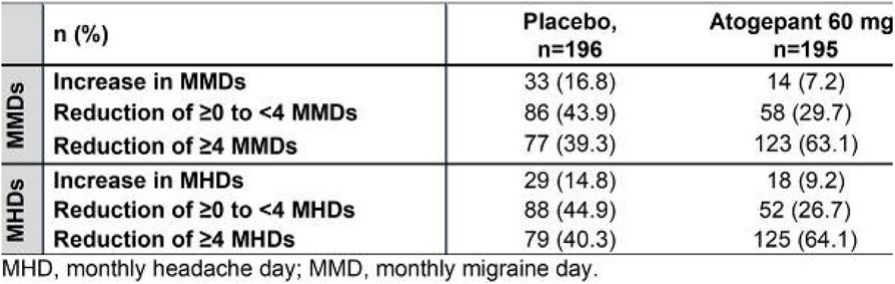
Change from baseline in monthly migraine days and monthly headache days at weeks 9-12

## P049 Evaluation of rimegepant utilization patterns for acute and preventive treatment of migraine in a commercially insured population

### J. Ailani^1^, J. Brown^2^, A. Jenkins^2^, K. Hygge Blakeman^2^, M. Lewis^2^, J. Cirillo^2^, R. B. Lipton^3^

#### ^1^Georgetown University Hospital, MedStar Georgetown Headache Center, Washington, DC, United States; ^2^Pfizer, Inc., New York, NY, United States; ^3^Montefiore Medical Center and Albert Einstein College of Medicine, Bronx, MA, United States

##### **Correspondence:** J. Brown

*The Journal of Headache and Pain 2024,* **25(Suppl 1)**: P049

**Objective:** To assess the utilization and patient characteristics of rimegepant, an oral calcitonin gene-related peptide (CGRP) antagonist approved for migraine treatment and prevention, in the United States.

**Methods:** This was a retrospective cohort study using the MarketScan administrative claims databases in the United States. Patients (≥18 years old) who newly initiated rimegepant and had ≥1 refill between March 1, 2020 to January 31, 2023 were included. Patients were then divided into acute treatment (quantity=8 tablets) or prevention (quantity=15 or 16 tablets) cohorts based on the index quantity dispensed and stratified by time periods before/after June 1, 2021 (addition of the prevention indication). Utilization periods were defined as the time between the first and last observed prescription fills plus a standard 90-day addition to account for "as needed" use. Patient demographic, clinical, and treatment history characteristics were identified during the 6-month pre-index period. Tablet utilization was standardized to the quantity dispensed per 30-days.

**Results:** A total of 16,177 rimegepant users were identified. Among "acute treatment" users, the utilization period (mean ± standard deviation) was 4.5 ± 2.2 tablets per 30 days over a use period of 340 ± 187 days. "Prevention" users had a follow-up of 225 ± 90 days and tablet utilization of 8.7 ± 2.8 tablets per 30 days. Rimegepant users (age 43 ± 11.5 years; 88.4% female) commonly used triptans (58.3%), non-steroidal anti-inflammatory drugs (35%), anti-CGRP monoclonal antibodies (30.5%), and opioids (30.3%) prior to initiation.

**Conclusion:** Tablet utilization for rimegepant acute treatment users was consistent over time and similar to literature benchmarks for migraine frequency. Lower than expected utilization in the assumed prevention user groups may be due to variable use patterns and requires further investigation.

## P050 The impact of fremanezumab treatment on depression severity in patients with concomitant antidepressant use: post hoc analysis of the UNITE study

### P. Barbanti^1,2^, C. Tassorelli^3,4^, V. Ramirez Campos^5^, L. J. Krasenbaum^5^, H. Akcicek^6^, M. Ortega^4^, R. Burstein^7^

#### ^1^IRCCS San Raffaele Roma, Headache & Pain Unit, Rome, Italy; ^2^San Raffaele University, Rome, Italy; ^3^University of Pavia, Department of Brain and Behavioral Sciences, Pavia, Italy; ^4^IRCCS C. Mondino Foundation, Pavia, Italy; ^5^Teva Branded Pharmaceutical Products R&D, Inc., West Chester, PA, United States; ^6^Teva Netherlands B.V., Amsterdam, Netherlands; ^7^Harvard Medical School, Department of Anaesthesia, Critical Care and Pain Medicine, Beth Israel Deaconess Medical Center, Boston, MA, United States

##### **Correspondence:** P. Barbanti

*The Journal of Headache and Pain 2024,* **25(Suppl 1)**: P050

**Objective:** The UNITE study demonstrated that treatment with fremanezumab, a humanized monoclonal antibody targeting calcitonin gene-related peptide, results in significant reductions in monthly migraine days (MMD), and improvements in depression outcomes in patients with migraine and major depressive disorder (MDD). This post hoc analysis evaluated the impact of fremanezumab on depression scores in patients with and without concomitant antidepressant treatment.

**Methods:** UNITE (NCT04041284) was a 12-week randomized (1:1), Phase 4, double-blind study with a 12-week open-label extension (OLE). Patients diagnosed with migraine and a history of MDD according to the DSM-V criteria ≥12 months prior to screening, and with active moderate-to-severe symptoms of depression (Patient Health Questionnaire-9 score ≥10) received monthly fremanezumab (225 mg) or matched placebo for 12 weeks. A single concomitant medication for depression was allowed if the dose was stable ≥8 weeks prior to screening, with no changes anticipated. A key secondary endpoint was the mean change from baseline in symptoms of depression measured by the 17-item Hamilton Depression Rating Scale (HAMD-17), where a reduction from baseline of ≥50% or 3–8 points is considered clinically meaningful.

**Results:** Of the 353 randomized patients (mean age, 42.9 years; 88% female), 330 completed the 12-week double-blind period. Of the 175 patients receiving fremanezumab, 114 had concomitant antidepressant use. Mean change from baseline in HAMD-17 score was –3.7 vs –3.9 at Week 4, –5.7 vs –6.9 at Week 8, and –6.3 vs –7.7 at Week 12 for patients treated with fremanezumab with and without concomitant antidepressant use, respectively (Figure 1).

**Conclusion:** In this post hoc analysis, clinically meaningful reductions in HAMD-17 scores were observed in patients with migraine and moderate-to-severe MDD, irrespective of concomitant antidepressant use. These results indicate that the reduction in the burden of migraine by fremanezumab can allow symptoms of depression to improve in this patient population.

**Fig. 1 (Abstract P050):**
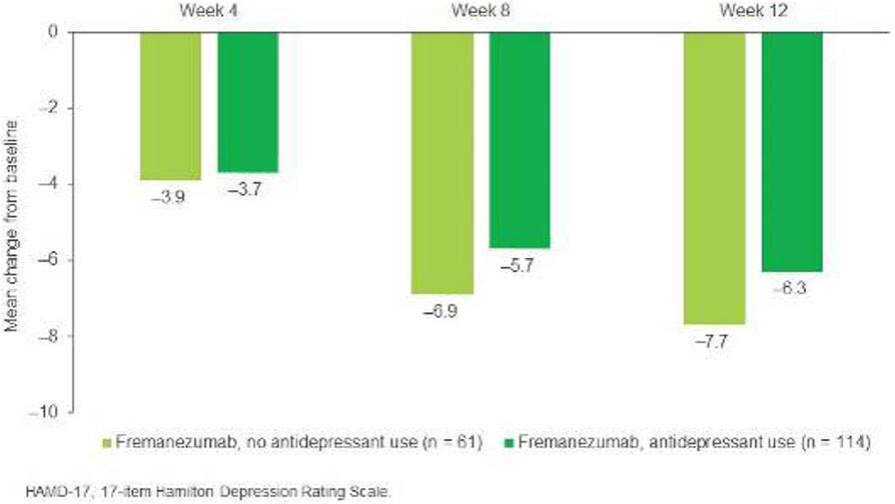
Reduction from baseline in HAM-D 17 in patients with and without concomitant medication use

## P051 Atogepant vs. rimegepant cost per treatment responder analysis for the preventive treatment of episodic migraine

### J. Ailani^1^, P. Gandhi^2^, A. Lalla^3^, R. B. Halker Singh^4^, P. McAllister^5^, J. H. Smith^6^, B. Dabruzzo^2^, N. Chalermpalanupap^2^, S. J. Nahas^7^

#### ^1^MedStar Georgetown University Hospital, Washington, DC, United States; ^2^AbbVie, Madison, NJ, United States; ^3^AbbVie, Irvine, CA, United States; ^4^Mayo Clinic, Scottsdale, AZ, United States; ^5^New England Institute for Neurology and Headache, Stamford, CT, United States; ^6^AbbVie, North Chicago, IL, United States; ^7^Jefferson Headache Center, Thomas Jefferson University, Department of Neurology, Philadelphia, PA, United States

##### **Correspondence:** P. Gandhi

*The Journal of Headache and Pain 2024,* **25(Suppl 1)**: P051

**Objective:** To understand the efficacy and economic value of oral calcitonin gene–related peptide receptor antagonists (gepants) for preventive treatment of episodic migraine (EM), we used a Bucher indirect comparison to assess number needed to treat (NNT) and cost per additional responder (CPR).

**Methods:** Bucher analysis was done to compare efficacy of atogepant (ato) and rimegepant (rime) using published data from registrational trials ADVANCE and BHV3000-305. For base case analysis, responder rate (RR) was defined as proportion of participants (PoPs) with ≥50% reduction in moderate-to-severe (MTS) monthly headache days during wks 9-12 for ato and the PoPs of participants with ≥50% reduction in MTS monthly migraine days (MMDs) during wks 9-12 for rime. In scenario analyses: 1) PoPs with ≥50% reduction in MMDs in wks 1-12 ato were compared to PoPs with ≥50% reduction in MTS MMDs in wks 9-12 rime, and 2) PoPs with ≥50% reduction in MMDs in wks 9-12 ato were compared to PoPs with ≥50% reduction in MTS MMDs in wks 9-12 rime. NNT: 1 ÷ ([probability of prespecified clinical response at prespecified timepoint with treatment]−[probability of same prespecified clinical response at same timepoint with control]). CPR: treatment cost over 12 wks × NNT.

**Results:** In base case analysis, ≥50% RR were 64.9% for ato, 51.8% for rime, vs 44.1% placebo. Median NNT vs placebo was 4.8 for ato vs 13.0 for rime (Figure 1). CPR vs placebo was $15,069 for ato vs $69,551 for rime (Figure 2). Scenario analysis 1: ≥50% RR were 67.5% for ato ,42.7% for rime, vs 35.4% placebo. Median NNT vs placebo was 3.1 for ato vs 13.7 for rime (Figure 1). CPR vs placebo was $9725 for ato vs $73,314 for rime (Figure 2). Scenario analysis 2: ≥50% RR were 70.0% for ato, 50.0% for rime, vs 42.4% placebo. Median NNT vs placebo was 3.6 for ato vs. 13.2 for rime (Figure 1). CPR vs placebo was $11,310 for ato vs $70,420 for rime (Figure 2).

**Conclusion:** These data suggest that ato may be more cost effective than rime for the preventive treatment of EM.

**Fig. 1 (Abstract P051):**
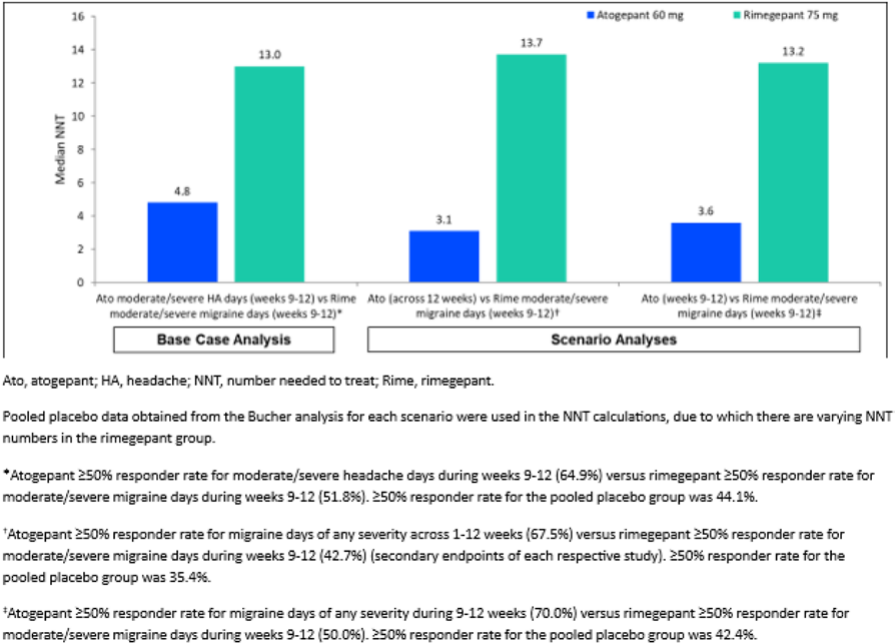
Median number needed to treat per additional ≥50% responder vs placebo in EM population

**Fig. 2 (Abstract P051):**
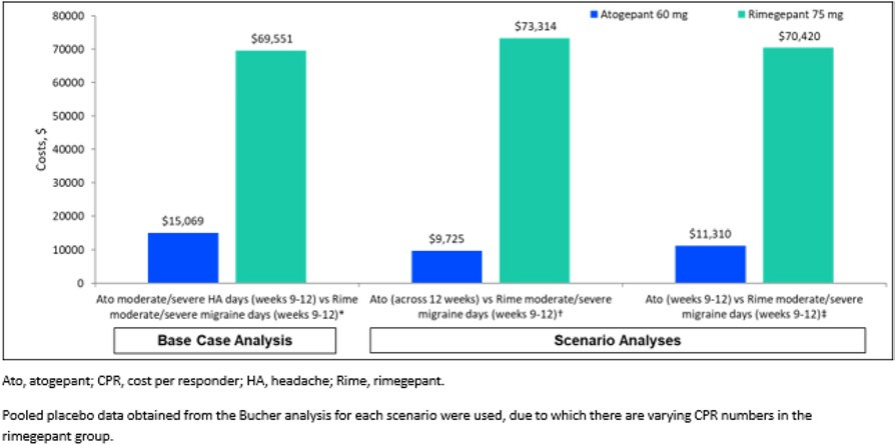
Median cost per additional ≥50% responder vs placeboin EM population

## P052 Efficacy of botulinum toxin type A in high frequency episodic migraine: a preliminary study

### B. Medeiros, M. J. Pinto, M. Pinto, A. Costa

#### Centro Hospitalar Universitário de São João EPE, Neurology, Porto, Portugal

##### **Correspondence:** B. Medeiros

*The Journal of Headache and Pain 2024,* **25(Suppl 1)**: P052

**Objective:** The objective of this study was to evaluate the efficacy of botulinum toxin type A (155/195 units) in the treatment of high frequency episodic migraine.

**Methods:** Data analysis was conducted using SPSS and R. Symptom outcomes were described as proportions with 95% confidence intervals. Binomial tests were employed to compare the estimated proportions of pain improvement against a null hypothesis assuming a 50% improvement rate in days of pain. Associations between treatment improvement and gender were assessed using Fisher's exact test, while age and number of initial episodes were analyzed using t-tests.

**Results:** A total of 55 patients diagnosed with episodic migraine were included in the study, the majority being females (96.4%). The mean age of the participants was 41.1 years, ranging from 24 to 63 years old. Following treatment with botulinum toxin, symptom outcomes were assessed at three, six, and nine months. The dropout rates were 0%, 29.1% (remaining patients: 40) and 63.6% (remaining patients: 20) at each respective time point. Excluding dropouts, symptom improvements were reported by 80.0% of patients at three months, 87.2% at six months, and 95.0% at nine months. Considering the high dropout rate, associations were analyzed with symptom outcomes at three months only. No significant associations were found with gender, age, or number of initial episodes.

**Conclusion:** While the current findings suggest a high prevalence of symptom improvement following treatment with botulinum toxin in high frequency episodic migraine, future studies should include larger samples and incorporate a control group**.**

## P053 How does chronic migraine change during three one-year cycles of CGRP-targeting monoclonal antibodies? A real-life study

### G. Vaghi^1,2^, R. De Icco^1,2^, M. Corrado^1,2^, F. Cammarota^1,2^, F. Bighiani^1,2^, C. Brancaccio^1^, E. Guaschino^1^, N. Ghiotto^1^, C. Tassorelli^1,2^, G. Sances^1,2^

#### ^1^IRCCS Mondino Foundation, Headache Science and Neurorehabilitation Centre, Pavia, Italy; ^2^University of Pavia, Department of Brain and Behavioral Sciences, Pavia, Italy

##### **Correspondence:** G. Vaghi

*The Journal of Headache and Pain 2024,* **25(Suppl 1)**: P053

**Objective:** In Italy an interruption period of at least 1 month is mandatory after 1-year cycle of monoclonal antibodies targeting CGRP (mAbs). Our primary aim was to assess mAbs effectiveness across 3 consecutive 1-year cycles (C1, C2 and C3) and related suspension periods as few evidence is actually available on their effectiveness during consecutive treatments.

**Methods:** Our baseline cohort was composed of 84 chronic migraine (CM) patients (72.6% females, 52.2±11.4years, medication overuse headache 90.5%). Of them, 46 patients were mAbs responders and underwent three 1-year cycles (T0 to T1_end_ for C1, S1-T2_end_ for C2, S2-T3_end_ for C3) separated by a suspension period of at least 3 months (S1,S2). Co-primary outcomes were: i) changes in monthly migraine days (MMDs) during each cycle compared to baseline (namely the 3 months prior to each cycle) and ii) during the related suspensions.

**Results:** MMDs showed an early and comparable reduction during the 3 cycles (C1 T0 22.2±5.1, T1_end_ 6.7±4.1; C2 S1 17.5±5.9, T2_end_ 7.7±4.2; C3 S2 14.0±5.5, T3_end_ 8.0±4.4, factorTIME *p*<0.001, factorGROUP=0.303) with a similar percentage of patients who achieved MMDs reduction >50% (86.7%, 73.3%, 77.8% for C1, C2 and C3)(Fig.1). During both S1 and S2 MMDs showed a worsening without reaching pretreatment values (S1 and S2 *p*<0.001, T1_end_ vs T0 *p*=0.035; T2_end_ vs T0 *p*<0.001). Notably, MMDs reached comparable values at the end of each cycle (*p*=1.000) with nearly half of patients still presenting more than 8 MMDs (57.8% at T1_end_, 48.9% at T2_end_, 44.4% at T3_end_). During the study period 66.7% patients reported at least one mild adverse event (namely constipation, injection site reactions or fatigue) with no discontinuation for adverse events.

**Conclusion:** In our cohort, mAbs induced a sustained reduction in MMDs during each 1-year cycle. MMDs worsened during the suspension periods still not reaching pretreatment levels. At the end of all cycles MMDs reached comparable values suggesting a *floor effect.*

**Fig. 1 (Abstract P053):**
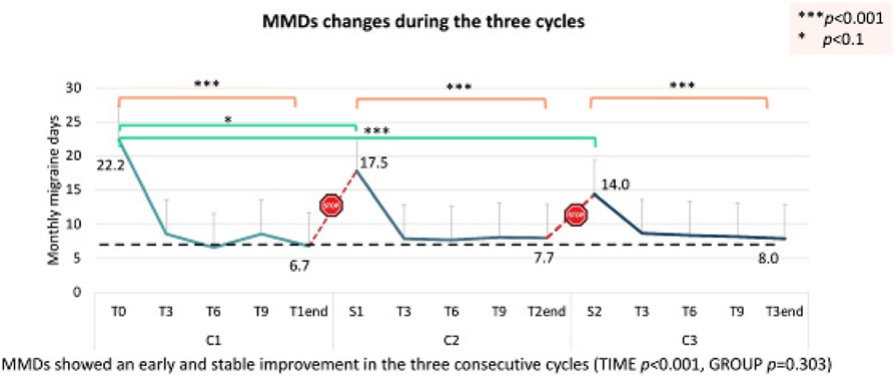
See text for description

## P055 Galcanezumab for migraine prevention in patients above 65: a multicenter study in real-world settings

### J. Peris-Subiza^1^, D. Guisado-Alonso^1^, E. Cuadrado Godia^1^, F. Velasco Juanes^2^, R. Álvarez Escudero^3^, N. Riesco Pérez^3^, L. González-Fernández^3^, A. Oterino Durán^3^, M. Martín Bujanda^4^, S. Aranceta Arilla^5^, A. Ruisanchez Nieva^6^, N. Roncero^7^, J. C. García-Moncó^7^, A. Mínguez-Olaondo^8^, M. Ruibal Salgado^8^, A. Echeverria Urabayen^9^, I. Kortazar Zubizarreta^9^, A. López-Bravo^10^, Á. L. Guerrero Peral^11^, D. García Azorín^11^, N. Fabregat Fabra^12^, S. Fernández-Fernández^12^, V. Obach Baurier^12^

#### ^1^Hospital del Mar, Neurology, Barcelona, Spain; ^2^Hospital Universitario Cruces, Neurology, Bilbao, Spain; ^3^Hospital Universitario Central de Asturias, Neurology, Oviedo, Spain; ^4^Hospital Universitario de Navarra, Neurology, Navarra, Spain; ^5^Hospital Parc Tauli, Neurology, Sabadell, Spain; ^6^Hospital Universitario de Galdakao-Usansolo, Neurology, Bilbao, Spain; ^7^Hospital Universitario de Basurto, Neurology, Basurto, Spain; ^8^Hospital Universitario de Donostia, Neurology, San Sebastian, Spain; ^9^Hospital Universitario de Áraba, Neurology, Osakidetza, Spain; ^10^Hospital Reina Sofía, Neurology, Zaragoza, Spain; ^11^Hospital Clínico Universitario de Valladolid, Neurology, Valladolid, Spain; ^12^Hospital Clínic i Provincial, Neurology, Barcelona, Spain

##### **Correspondence:** J. Peris-Subiza

*The Journal of Headache and Pain 2024,* **25(Suppl 1)**: P055

**Objective:** Galcanezumab is a *monoclonal antibody* used as a preventive treatment for patients with chronic migraine (CM) and high-frequency episodic migraine (HFEM). Elderly patients (≥ 65 years) are underrepresented in galcanezumab clinical trials. Our aim is to describe the efficacy and tolerability in this group of older patients.

**Methods:** Real-life, multicenter cohort study of consecutive patients with CM or HFEM with prior failure to three or more migraine preventive drugs, treated with galcanezumab and followed up for 12 months. Efficacy (measured as the percentage of patients obtaining a reduction of at least 50% (R50%) of monthly headache days (MHD)) at 3, 6 and 12 months and tolerability (measured as drug withdrawal due to side effects) were compared between patients <65 and ≥65 years. We also analyzed reduction of 75% of MHD (R75%).

**Results:** We included 1055 patients, 934 patients (88.5%) <65 and 121 patients (11.5%) ≥65 years old. Patients ≥ 65 years had a higher number of baseline MHD [25 (IQR 15-30) vs 20 (14-30), *p*=0.045] and a lower HIT-6 score [66.5 (IQR 63-72) vs 69 (66-73), *p*<0.001]. There were no differences in terms of R50% between patients over and under 65 years at 3 months (57% vs 48.8%, *p*=0.090), 6 months (54.6%, vs 48.1%, *p*=0.180) or 12 months (52.1%, vs 47.9%, *p*=0.384). However, R75% was more frequent in the ≥65 years subgroup (3 months: 32.23% vs 23.13%, *p*= 0.028; 6 months: 32.2% vs 21.5%, *p*= 0.008; 12 months: 33.1% vs 23.6%, *p*= 0.022). The drug was well tolerated, and no significant differences were found in the rate of drug withdrawal due to side effects through 12 months of follow up (5.8% in patients ≥65 years vs 6.7% in patient <65, *p*=0.837).

**Conclusion:** Galcanezumab in patients ≥65 years was as effective and well tolerated as in younger ones, but a higher percentage of older patients obtained an excellent response to the treatment.

## P058 Evaluation of the accuracy of the predictive algorithm of response to anti-CGRP monoclonal antibodies in patients with migraine older than 65 years: external validation study

### C. Sánchez-Rodríguez^1^, A. Gago-Viega^1^, J. Pagán^2,3^, I. Fernández Lázaro^1^, J. S. Rodrígue-Vico^4^, A. Jaimes Sanchez^4^, A. Gómez García^4^, J. Casas Limón^5^, J. Díaz de Terán^6^, M. Sastre Real^6^, J. A. Membrilla López^6^, G. Latorre^7^, C. Calle de Miguel^7^, S. Gil Luque^8^, C. Trevino-Peinado^9^, S. Quintas Gutierrez^1,9^, P. Heredia^1,9^, D. García Azorín^10^, A. Echavarría Íñiguez^10^, Á. L. Guerrero Peral^10^, Á. Sierra-Mencía^10^, N. González García^11^, J. Porta-Etessam^11^, A. González Martínez^1^

#### ^1^Hospital Universitario de la Princesa, Neurology, Madrid, Spain; ^2^CCS: Center for Computational Simulation, Madrid, Spain; ^3^Electronic Engineering Department, Madrid, Spain; ^4^Headache Unit, Neurology Department, Madrid, Spain; ^5^Headache Unit, Neurology Department, Alcorcón, Spain; ^6^Headache Unit, Neurology Department, Madrid, Spain; ^7^Headache Unit, Neurology Department, Hospital Universitario de Fuenlabrada,, Fuenlabrada, Spain; ^8^Headache Unit, Neurology Department, Burgos, Spain; ^9^Headache Unit, Neurology Department, Madrid, Spain; ^10^Headache Unit, Neurology Department, Hospital Clínico Universitario de Valladolid, Valladolid,, Valladolid, Spain; ^11^Headache Unit, Neurology Department, Madrid, Spain

##### **Correspondence:** A. González Martínez

*The Journal of Headache and Pain 2024,* **25(Suppl 1)**: P058

**Objective:** The prediction of response to recent anti-CGRP therapies is a topic of interest in the field of migraine. Previous studies carried out in our group have developed a predictive tool for response to anti-CGRP using an approach based on machine-learning techniques. The aim of this study was to validate this tool and its usefulness in patients with chronic (CM) and episodic (EM) migraine.

**Methods:** Multicenter, retrospective cohort study, with patients with migraine from 9 Headache Units, different from the model generation cohort. Sensitivity(S), specificity(E) and global positive (PPV) and negative (NPV) predictive values and for the different groups were obtained.

**Results:** 127 patients with migraine were included, 104 (81.88%) with CM, 108 (85.03%) women and mean age 53.73 (SD 13.84) years. In the evaluation of the algorithm of global response greater than 50% at 6 months, the global S was 78.04% and the global E was 80%. The area under the curve (AUC) was 0.790, CI[0.726-0.849) and weighted F1 of 79% in the validation cohort, with an AUC of 0.819 CI[0.762-0.884] and weighted F1 of 81.88% in MC and AUC of 0.592 CI[0.322-0.842] with a weighted F1 of 68.97% in EM.

**Conclusion:** Our study confirms the external validity of the predictive model of response to anti-CGRP in a different cohort from the generation of the algorithm. The S and E of the predictive model were higher in the group of patients with CM. Future models could improve the predictive capacity of this tool in patients with EM.

## P060 Effectiveness and tolerability of CGRP monoclonal antibodies in combination with monoclonal antibodies for other diseases: a multicenter study

### F. Pistoia^1^, L. F. Iannone^2^, A. Russo^3^, G. Saporito^1^, R. Ornello^1^, F. De Santis^1^, M. Albanese^4^, C. Tassorelli^5^, S. Guerzoni^6^, G. Sances^5^, G. Vaghi^5^, A. Casalena^7^, G. Dalla Volta^8^, E. Mampreso^9^, M. Valente^10^, P. Geppetti^2^, S. Sacco^1^

#### ^1^University of L'Aquila, L'Aquila, Italy; ^2^University of Florence, Health Sciences, Florence, Italy; ^3^University of Campania Luigi Vanvitelli, Naples, Italy; ^4^Tor Vergata University of Rome, Rome, Italy; ^5^IRCCS Mondino Foundation, Pavia, Italy; ^6^AOU Policlinico di Modena, Modena, Italy; ^7^G. Mazzini Hospital, Teramo, Italy; ^8^Istituto Clinico Città di Brescia, Brescia, Italy; ^9^Euganea, Health Unit, Padua, Italy; ^10^Azienda Sanitaria Universitaria Friuli Centrale, Presidio Ospedaliero Santa Maria della Misericordia, Udine, Italy

##### **Correspondence:** F. Pistoia

*The Journal of Headache and Pain 2024,* **25(Suppl 1)**: P060

**Objective:** Is it safe and effective combining calcitonin gene-related peptide antibodies (CGRP-mAbs) with monoclonal antibodies for diseases different from migraine?

**Methods:** Outpatients of the "Italian Headache Registry" (RICe), treated with CGRP-mAbs for migraine while simultaneously assuming monoclonal antibodies for other pathologies, were screened and included if they had a 6-month follow-up after the start of the two co-prescribed drugs. Efficacy outcomes were reduction from baseline of Monthly Headache Days (MHDs), MIDAS scores and HIT-6 questionnaire scores, as well as subjective improvement scored through the Patients' Global Impression of Change (PGIC) scale. Safety outcomes included the identification of side effects different frommonotherapy.

**Results:** Twenty-six patients (21 women; age 50.3±9.7) were included. In most of cases (*n*=16; 61%) the CGRP-MAb (erenumab, galcanezumab or fremanezumab) was added to a previously ongoing treatment with another MAb (ie, adalimumab, ocrelizumab, omalizumab, natalizumab, ustekinumab, risankizumab, tocilizumab, etanabercept, denosumab, certolizumab, evolocumab) for psoriatic arthritis (*n*=6; 23%), osteoporosis (6; 23%), ankylosing spondylitis (4; 15%), rheumatoid arthritis (3; 11%), multiple sclerosis (2; 8%), asthma (2; 8%), ulcerative colitis (1; 4%), vasculitis (1; 4%) and dyslipidemia (1; 4%). MHDs (mean±SD, 20.7±6.1 vs 11.8±8.0; *p*<0001), MIDAS scores (75.0±41.9 vs 30.3±27.7; *p*=0.001) and HIT-6 scores (66.5±10.5 vs 53.4±8.7; *p*=0.002) significantly decrease from baseline to 6 months. The PGIC score was high both for anti-CGRP mAbs (5.48±2.6) and other mAbs (5.5±2.9). The introduction of the second mAb resulted in mild gastrointestinal symptoms with the co-prescription of evolocumab and erenumab and in mild alopecia with galcanezumab and ustekinumab.

**Conclusion:** The combination of different mAbs may be considered safe and effective in daily clinical practice.

## P061 Evaluation of the concomitant use of preventive treatments in patients with migraine under anti-CGRP therapies: the PREVENAC study

### A. González Martínez^1^, N. López Alcaide^1^, I. Fernández Lázaro^1^, S. Quintas Gutierrez^1^, J. Casas Limón^2^, C. Calle de Miguel^3^, G. Latorre^3^, N. González García^4^, J. Porta-Etessam^4^, J. S. Rodrígue-Vico^5^, A. Jaimes Sanchez^5^, A. Gómez García^5^, D. García Azorín^6^, Á. L. Guerrero Peral^6^, Á. Sierra-Mencía^6^, A. Lozano Ros^7^, A. Sánchez Soblechero^7^, J. Díaz de Terán^8^, J. A. Membrilla López^8^, C. Trevino-Peinado^9^, A. Gago-Viega^1^

#### ^1^Hospital Universitario de la Princesa, Neurology, Madrid, Spain; ^2^Headache Unit, Neurology Department, Alcorcón, Spain; ^3^Headache Unit, Neurology Department, Hospital Universitario de Fuenlabrada, Fuenlabrada, Spain; ^4^Headache Unit, Neurology Department, Madrid, Spain; ^5^Headache Unit, Neurology Department, Madrid, Spain; ^6^Headache Unit, Neurology Department, Hospital Clínico Universitario de Valladolid, Valladolid,, Valladolid, Spain; ^7^Headache Unit, Neurology Department, Madrid, Spain; ^8^Headache Unit, Neurology Department, Madrid, Spain; ^9^Headache Unit, Neurology Department, Madrid, Spain

##### **Correspondence:** A. González Martínez

*The Journal of Headache and Pain 2024,* **25(Suppl 1)**: P061

**Objective:** Antibodies against calcitonin gene-related peptide (CGRP) are recent preventive therapies for migraine. One of the measures of effectiveness is the withdrawal of other preventive treatments. The objective of this study is to quantify the impact of anti-CGRP drugs on other preventive treatments in patients with migraine.

**Methods:** Observational, retrospective, cross-sectional, multicenter study with patients from the Headache Units of nine Spanish hospitals. Patients with migraine treated for at least 6 months with anti-CGRP antibodies, who received some preventive treatment, were included. Demographic and clinical variables were collected, as well as variables related to headache. Differences according to withdrawal or non-withdrawal were evaluated.

**Results:** 408 patients were included, 86.52% women, 48.79(SD:1.46) years. Preventive treatment was withdrawn in 43.87% patients, 20.83% partially and 23.04% totally. The variables associated with withdrawal were comorbidities (insomnia, arterial hypertension, obesity), type of migraine, excessive use of medication, a greater number of headache/migraine days per month, and a greater number of preventive treatments. In multivariate analysis, more headache/migraine days at baseline, fewer migraine days per month with anti-CGRP, and greater than or equal to 50% response were independently associated with treatment withdrawal (*p*<0.05).

**Conclusion:** Anti-CGRP antibodies allow the withdrawal of preventive treatment in a significant percentage of patients, which supports their role in evaluating the effectiveness of anti-CGRP drugs in real life.

## P062 Headache due to excessive use of medication: prevalence after preventive treatment with antibodies directed against the CGRP pathway

### P. Santamaría Montero, L. Abraira Carballido, L. Ramos Rua, R. Pego Reigosa

#### Hospital Universitario Lucus Augusti, Neurología, Lugo, Spain

##### **Correspondence:** P. Santamaría Montero; L. Abraira Carballido

*The Journal of Headache and Pain 2024,* **25(Suppl 1)**: P062

**Objective:** To know the effectiveness of preventive treatment with antibodies against the CGRP pathway in medication overuse headache (MOH) in patients with migraine.

**Methods:** Retrospective observational study of 45 patients from the Lucus Augusti University Hospital diagnosed with high-frequency episodic migraine (20%) and chronic migraine (80%) according to the International Headache Society, treated for at least three months with anti-CGRP antibodies and who previously met criteria for excessive use of analgesic medication. The efficacy of the treatment is evaluated using the HIT-6 scale and the number of days of headache and analgesic consumption monthly at baseline and at 3, 6 and 12 months. Responders were considered those patients with a decrease of at least 50% in headache days and analgesic intake per month with respect to their baseline.

**Results:** The baseline score of the HIT-6 scale of 68.8 points, with 20 days of headache and 18 days of analgesic consumption per month. The score on the HIT-6 scale was 64.3 at 3 months, 57.2 at 6 months, and 54.3 at 12 months. Headache days were reduced by 48% at 3 months, 62% at 6 months, and 65% at 12 months compared to baseline. The days of analgesic consumption decreased by 45.5%, 56.6%, and 63% at 3, 6, and 12 months, respectively.

**Conclusion:** Treatment with antiCGRP antibodies is effective in the treatment of MOH in patients with chronic migraine and high-frequency episodic migraine.

## P063 Question: Can monoclonal antibodies prevent chronic migraine without aura?

### G. Chakhava^1,2,3^, T. Kakubava^1,3^, M. Demuria^1,3^, *V. Nemsadze*^1^

#### ^1^Multiprofile Clinic Consilium Medulla, Neurology, Tbilisi, Georgia; ^2^D.Tvildiani Medical University, Medical Faculty, Tbilisi, Georgia; ^3^Georgian Association of Medical Specialties, Tbilisi, Georgia

##### **Correspondence:** G. Chakhava

*The Journal of Headache and Pain 2024,* **25(Suppl 1)**: P063

**Objective:** Primary Objective: The primary objective of this study was to evaluate the effectiveness of Eptinezumab in reducing the monthly frequency of migraine headaches by at least 50%.

Secondary Objective: The secondary objective of this study was to assess the impact of Eptinezumab treatment on decreasing the level of emotional distress associated with migraines

**Methods:** In a double-blinded study, 33 adults with an average age of 52.5 years, all of whom had a history of migraine headaches without an aura were randomly assigned in a 2:1 ratio, with 23 patients receiving a one-year treatment of IV Eptinezumab once every three months, and the remaining participants receiving a placebo. The results were assessed using monthly questionnaires per protocol.

**Results:** 78.2% of patients in the Eptinezumab group had at least 50% fewer migraine attacks during 3 month period compared to 10% patients in placebo group, for an estimated absolute risk reduction (ARR) of 68.2% and number needed to treat (NNT) of 1.465 CI [1.035-2.506]. Eptinezumab's efficacy in reducing migraine frequency was statistically significant (*p*=0.0005). In contrast, the study yielded no significant difference in emotional distress reduction between the Eptinezumab and placebo groups. The hazard ratio (HR) was calculated to be 0.9 (*p*= 0.7). Additionally, 5 patients from the treatment group experienced rhinorrhea.

**Conclusion:** Eptinezumab demonstrated statistically and clinically significant effectiveness in reducing migraine frequency by 50% or more in patients with migraine without aura. However, emotional distress reduction did not significantly differ between Eptinezumab and placebo groups.

**Fig. 1 (Abstract P063):**
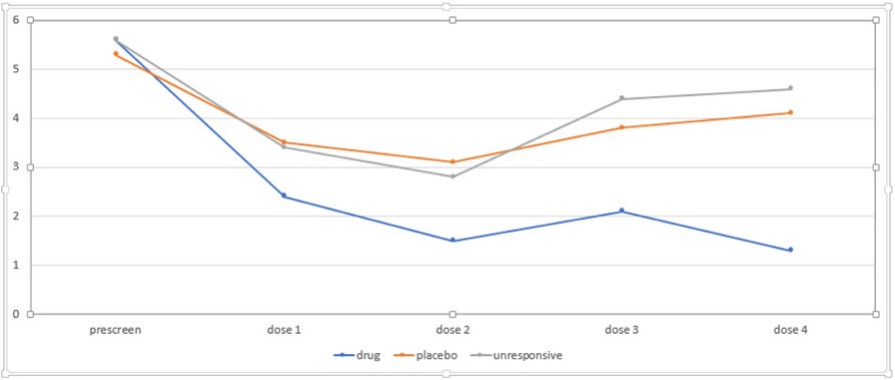
See text for description

**Fig. 2 (Abstract P063):**
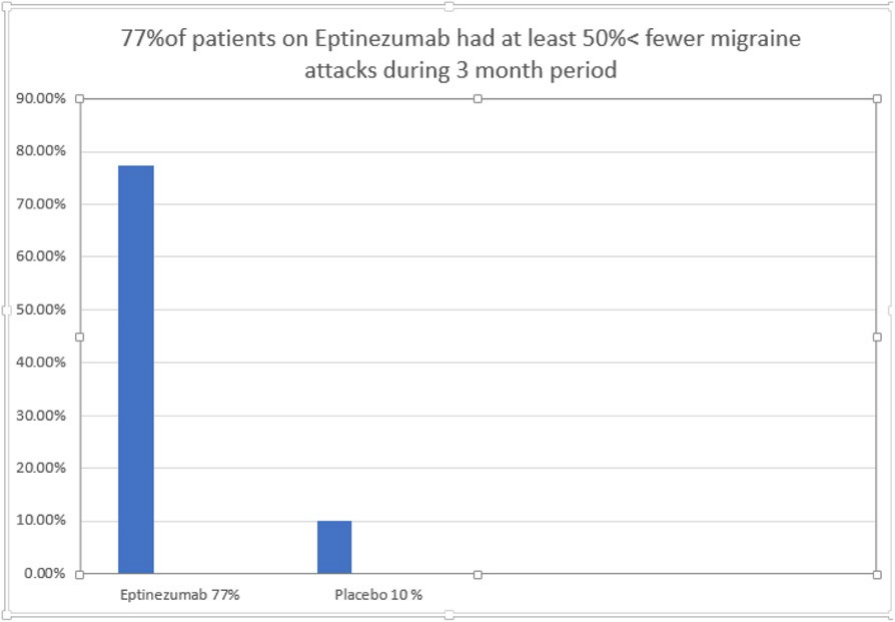
See text for description

## P064 Botulinum toxin as an off-label treatment of other primary and secondary headaches

### B. Nunes Vicente^1^, F. Dourado Sotero^1,2^, I. Pavão Martins^1,2,3^

#### ^1^Centro Hospitalar Universitário Lisboa Norte, Headache Center, Lisbon, Portugal; ^2^Faculdade de Medicina da Universidade de Lisboa, Lisbon, Portugal; ^3^Centro de Estudos Egas Moniz, Faculdade de Medicina da Universidade de Lisboa, Lisbon, Portugal

##### **Correspondence:** B. Nunes Vicente

*The Journal of Headache and Pain 2024,* **25(Suppl 1)**: P064

**Objective:** Botulinum toxin type A (BoNT) has been approved for preventive treatment in chronic migraine. However, it has been used off-label in other headaches, due to its possible modulating effect on the trigeminovascular system. Our aim was to analyze the clinical response to BoNT treatment in patients with other refractory headaches.

**Methods:** A descriptive, retrospective study in adults with headaches who underwent off-label BoNT, identified through the analysis of records from a Headache Center of a tertiary hospital. Demographic variables, diagnosis, the protocol used, and Patient Global Impression of Change (PGIC) and Headache Impact Test-6 Item scales were collected. All patients signed informed consent.

**Results:** A total of 39 patients were included, of whom 66,7 %(*n*=26) were female, with a median of 58,6 (33-87) years of age. The main diagnoses were nummular headache (*n*=18;47%), trigeminal autonomic cephalgias (TAC) (*n*=8;21%), trigeminal neuralgia (*n*=4;11%), and other headaches (*n*=8;21%). The protocol "follow the pain" was used for nummular headaches and trigeminal neuralgia, and the protocol Phase III Research Evaluating Migraine Prophylaxis was adapted for the remaining cases. The median dose used was 50 (25-150) units. Significant improvement (score of 1 or 2 on PGIC) was observed in 38,9% (*n*=7) of the patients with nummular headache, 50% (*n*=2) with trigeminal neuralgia, 37,5% (*n*= 3) with TAC and in 2 patients with daily persistent headache with a chronic migraine phenotype, attributed to a ruptured arteriovenous malformation and a subarachnoid hemorrhage.

**Conclusion:** This uncontrolled case series highlights the efficacy and safety of BoNT in various types of primary and secondary headaches, placing it as a possible therapeutic strategy in refractory headaches. Becomes clear the need for randomized clinical trials for a better selection of patients and protocols.

## P065 Adverse effects of CGRP mAbs: experience from a tertiary center in migraine treatment

### J. M. Alves, C. Fernandes, M. Coelho, I. Costa, G. Cordeiro, C. Machado, F. Magalhães, H. Gens, S. Batista, L. Sousa, I. Luzeiro

#### Centro Hospitalar e Universitário de Coimbra, E P.E., Neurology Department, Coimbra, Portugal

##### **Correspondence:** C. Fernandes

*The Journal of Headache and Pain 2024,* **25(Suppl 1)**: P065

**Objective:** The primary objective of this study was to examine the adverse effects experienced by patients with migraines who received CGRP mAbs and were monitored in a specialized headache clinic at a tertiary hospital.

**Methods:** In this study, we conducted a retrospective analysis on the medical records of 46 patients suffering from uncontrollable migraines despite receiving conventional treatment. These patients were prescribed CGRP mAbs (erenumab, fremanezumab, and galcanezumab) and underwent at least one assessment following the initiation of this medication.

**Results:** Our research included a population consisting of 46 patients. On average, the treatment lasted for 9.9 months (SD 5.2). Among those patients, 28 received erenumab, 11 received fremanezumab, and 7 received galcanezumab. One patient treated with erenumab developed a hypersensitivity reaction 6 days after the first administration, characterized by a sore throat, generalized pruritic rash, and swollen lips. Corticosteroid therapy was necessary for treatment. Other reported side effects under erenumab included complaints of psychomotor slowing and fatigue (3; 10.7%), intestinal constipation (2; 7.1%), worsening of vertigo syndrome (1), and a metallic taste after administration (1). In the case of fremanezumab, 2 patients (18.2%) experienced a self-limited cutaneous reaction at the injection site, leading to the substitution with erenumab in one patient. In one patient who received galcanezumab, falls without vertigo or trauma were documented.

**Conclusion:** The outcomes of this study provide further support for the notion that CGRP mAbs are well-tolerated medications with a diminished occurrence of side effects. The majority of reported adverse events were mild in nature and did not warrant discontinuation of the drug, except in one patient who experienced an idiosyncratic reaction, and another who developed a self-limited cutaneous reaction at the injection site.

**Fig. 1 (Abstract P065):**
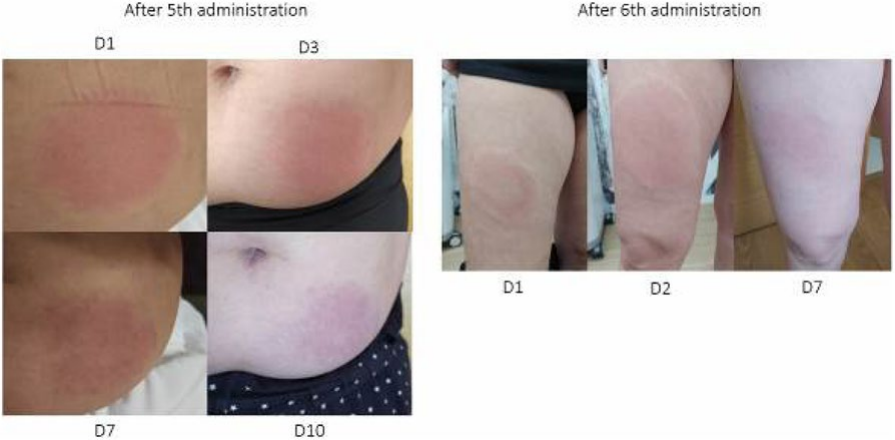
See text for description

**Fig. 2 (Abstract P065):**
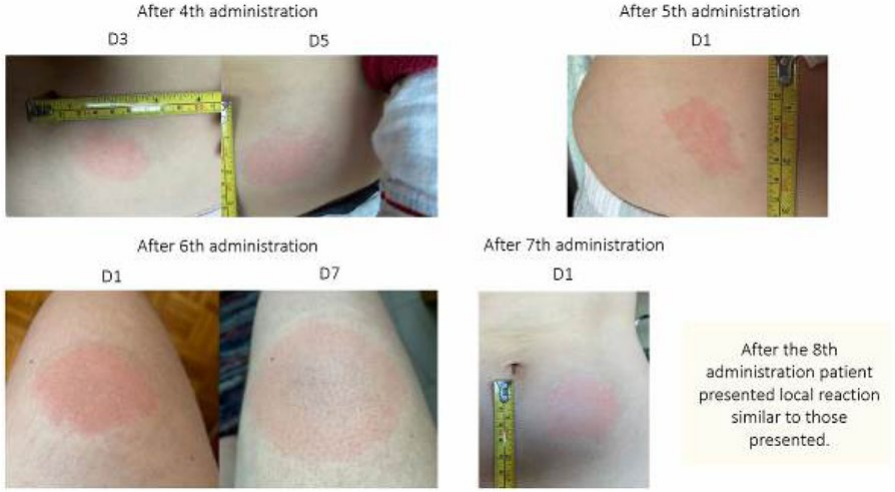
See text for description

## P066 Real world experience after one year of treatment with antiCGRP monoclonal antibodies in patients with chronic migraine

### V. González-Quintanilla, J. Madera Fernandez, G. Gárate, S. Pérez-Pereda, J. Pascual Gómez

#### University Hospital Marqués de Valdecilla, Neurology, Santander, Spain

##### **Correspondence:** V. González-Quintanilla; J. Madera Fernandez; G. Gárate

*The Journal of Headache and Pain 2024,* **25(Suppl 1)**: P066

**Objective:** To analyze the experience with anti-CGRP monoclonal antibodies in patients with refractory chronic migraine (CM) in real clinical practice, and to know the evolution after one year of treatment.

**Methods:** Demographic, efficacy and tolerability parameters were collected prospectively in the first patients with CM who started treatment with anti-CGRP antibodies in the Headache unit of a tertiary level hospital in Spain. Baseline and quarterly data were recorded until a minimum of one year of treatment was completed. Furthermore, we analyzed the migraine recurrence rate 3 months after withdrawal of treatment and its correlation with demographic and clinical evolution parameters.

**Results:** 120 patients with CM have completed at least one year of treatment with anti-CGRP antibodies (87.5% women, mean age 50.5 ± 10.3 years). The headache days per month showed a mean decrease of 11.65 days. 58.04% of the patients had a reduction of more than 50% in headache days per month. Treatment was withdrawn in 52 patients and 54.71% of this patients presented a recurrence of headache frequency. The mean time to migraine recurrence was 1.72 ± 0.70 months. Treatment with antiCGRP was well tolerated and serious adverse effects were not reported.

**Conclusion:** Routine clinical practice results confirm the efficacy of Anti-CGRP antibodies after one year of treatment in patients with CM and indicate that a high percentage of patients who withdraw the treatment after one year recur within 3 months.

## P069 Amitriptyline versus quetiapine for migraine prophylaxis in Saudi population

### A. Alasmari^1,2^, H. Younis^1,2^, F. Alhamaid^1,2^

#### ^1^King Fahad Military Hospital, Neuroscience, Jeddah, Saudi Arabia; ^2^King Fahad Military Hospital, Jeddah, Saudi Arabia

##### **Correspondence:** A. Alasmari

*The Journal of Headache and Pain 2024,* **25(Suppl 1)**: P069

**Objective:** the aim of this study was to evaluate the use of Quetiapine (QTP) in the preventive treatment of migraine through head to head study with one of the most common used medication for migraine prophylaxis and sharing almost the same characters regarding its effect on sleep.

**Methods:** our research study is head to head study where we enrolled all newly diagnosed migraine patients from January 2023 to April 2023 and they randomly divided into 4 equal groups (2 groups are using amitriptyline 10 and 25 mg respectively and 2 groups are using quetiapine 25 and 50 mg respectively) and we followed them for 3 months and we compare their headache diary after 3 months with their initial diary at time of diagnosis .

**Results:** total 112 patients are enrolled in our study 85 female (76%) and 27 male (24%),with mean age SD 32.4 +_ 6.2 ,the change in migraine frequency after 3 months in the amitriptyline groups (10,25 mg ) was 58.4 % and 71 % respectively while in the quetiapine groups (25,50 mg ) was 50.3 and 61.9% respectively (P value 0.002).but the drop out percentage was higher in the quetiapine groups 35.7% in the 25 mg group and 50 % in the 50 mg group.

**Conclusion:** our current study showed that quetiapine was not inferior to amitriptyline regarding the efficacy as a migraine prophylaxis treatment option but the compliance was not the same like in amitriptyline group.

**Fig. 1 (Abstract P069):**
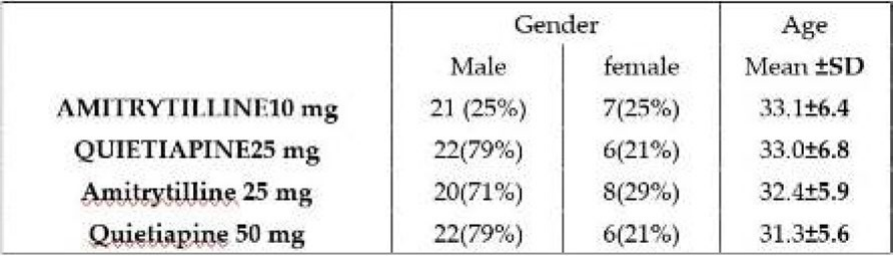
See text for description

**Fig. 2 (Abstract P069):**
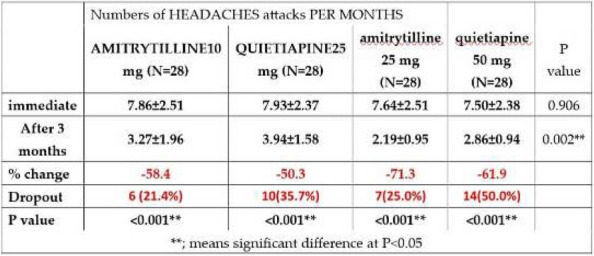
See text for description

**Fig. 3 (Abstract P069):**
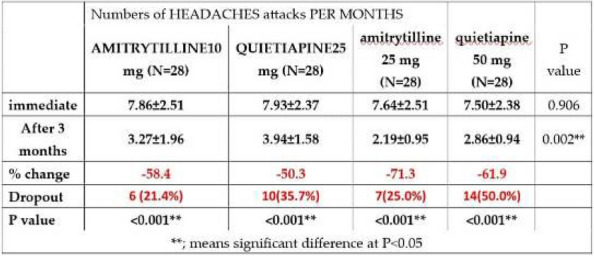
See text for description

## P070 Preventive and therapeutic effects of rimegepant on medication overuse headache: based on a mouse model that is more in line with clinical characteristics

### C. Li, Z. Ma, W. Xie, S. Yu

#### Department of Neurology, the First Medical Center of Chinese PLA General Hospital, Medical School of Chinese PLA, Haidian District Beijing, China

##### **Correspondence:** C. Li; Z. Ma

*The Journal of Headache and Pain 2024,* **25(Suppl 1)**: P070

**Objective:** Rimegepant, a small molecule calcitonin gene-related peptide (CGRP) antagonist, is approved as an oral medication for the acute treatment of migraine. It is currently known from studies that repeated administration of rimegepant does not induce the development of medication overuse headache (MOH) in mice. The aim of this study was to investigate whether rimegepant could prevent the formation of MOH or play a role in the treatment of MOH.

**Methods:** A more clinically characterized mouse model: in this study, mice were injected intraperitoneally with nitroglycerin every other day for 2 weeks. Rizatriptan solution was given daily for 2 weeks to make the mice actively ingest it, and the consumption of rizatriptan was recorded daily. Plantar and cephalo-facial sensory thresholds were measured in the intervals between nitroglycerin injections. After the pain threshold was recovered, low-dose sodium nitroprusside was verified, and staining was performed to screen the differential brain regions.2. Rimegepant (100 mg/kg) was given intraperitoneally every other day during the model formation and drug withdrawal periods, and the mice were monitored for changes in the pain thresholds, and the changes of c-Fos exprssion in the differential brain regions were counted.

**Results:** The novel MOH model was more sensitive to the decrease in pain threshold and the duration of pain sensitization was longer than the other groups, and the active intake of rizatriptan was significantly increased. Compared with the control group, the new MOH model showed significantly higher c-Fos expression in the regions of OFC, CeA, AID/AIV, and VTA. 2. Mice given rimegepant for prevention did not develop MOH due to the overdose of Rizatriptan, whereas MOH mice given rimegepant for treatment left the MOH state faster and did not have nociceptive pain sensitization induced by the injection of sodium nitroprusside.

**Conclusion:** Rimegepant can reduce the number of activated trigeminal ganglia CGRPergic neurons, decrease the activation of OFC, CeA, AID/AIV, and VTA, prevent the formation of MOH, and rapidly reverse the medication overuse state in mice.

**Fig. 1 (Abstract P070):**
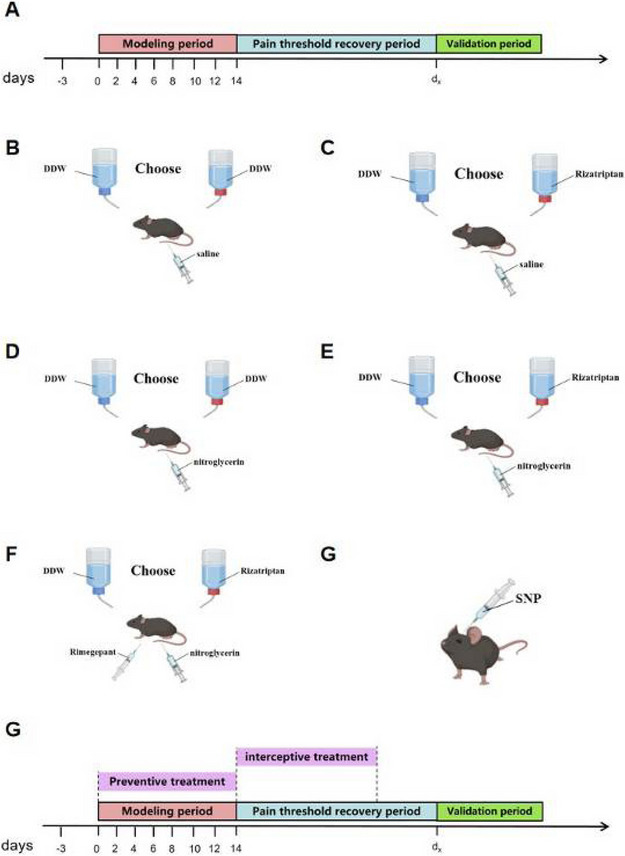
See text for description

## P071 Ubrogepant in the treatment of vestibular migraine: case report

### B. Torphy, C. Medrano, M. Smith, B. Murphy, J. Straube, M. Helle, B. Ranchero

#### Chicago Headache Center and Research Institute, Chicago, IL, United States

##### **Correspondence:** B. Torphy

*The Journal of Headache and Pain 2024,* **25(Suppl 1)**: P071

**Objective:** We present a case of a 59 year-old female with vestibular migraine whose moderate to severe episodes are treated with ubrogepant.

**Methods:** We present a case of a 59 year-old female with a history of migraine for over twenty years prior to the onset of frequent symptoms of vertigo. Two years prior to presentation in our clinic she had spontaneous onset of vertigo, which she described as "room spinning." Vertigo symptoms were constant for one year and were accompanied by a daily headache. She underwent vestibular physical therapy for one year and noted reduction in severity of vertigo but no reduction in daily headache. At her first presentation in our clinic she reported having daily headaches and 1-7 episodes of moderate to severe vestibular migraine per month, with each lasting 1-2 days. In our clinic she began gacanezumab 120 mg subcutaneous injection monthly (after a loading dose of 240 mg). The patient was instructed to take ubrogepant 50 mg at the onset of moderate to severe vestibular migraine symptoms, and she was instructed to take a second dose in two hours if symptoms had not resolved.

**Results:** On the first follow up visit the patient reported one dose of ubrogepant 50 mg resolved her vestibular migraine symptoms within 25 minutes. She continued to take galcanezumab 120 mg monthly, and by the sixth month her daily headaches had reduced to between 3-4 headache days per month. Vestibular migraine episodes have become less frequent, ranging from 1-4 per month, and they continue to be triggered by weather changes. She continues to take ubrogepant 50 mg at the onset of moderate to severe vestibular symptoms, and she reports "the vestibular sensation goes away in 25 minutes, and one pill always does the trick!" She has not required a second dose of ubrogepant 50 mg during any of her vestibular migraine episodes.

**Conclusion:** These results suggest ubrogepant may have utility for patients in the acute treatment of vestibular migraine. Further research, including real-world evidence, is warranted to investigate the use of ubrogepant in this setting.

*Disclosure statement:* Informed consent to publish this case study and its potentially identifiable information of the patient was obtained from the individual involved. The patient gave explicit permission for the publication of this case report, including any relevant clinical details.

## P072 Neck pain and medication overuse headache

### S. J. Cho^1^, H. K. Park^2^, S. Y. Oh^3^, T. J. Song^4^, H. S. Moon^5^, M. K. Chu^6^, M. K. Kang^1^

#### ^1^Hallym University College of Medicine, Neurology, Hwaseong, South Korea; ^2^Inje University Ilsan Paik Hospital, Inje University College of Medicine,, Koyang, South Korea; ^3^Chonbuk National University Hospital, Jeonju, South Korea; ^4^Ewha Womans University Seoul Hospital, Seoul, South Korea; ^5^Kangbuk Samsung Hospital, Seoul, South Korea; ^6^Severance Hospital, Yonsei University College of Medicine, Seoul, South Korea

##### **Correspondence:** S. J. Cho

*The Journal of Headache and Pain 2024,* **25(Suppl 1)**:

**Objective:** To investigate the frequency and severity of neck pain in a prospective registry of medication overuse headache

**Methods:** We analyzed the data of on-going multicenter cooperative registry (Registry for Load and Management of MEdicAtion OveruSE Headache [RELEASE]) since April 2020. Treatment for MOH included withdrawal or tapering of overused medication and preventive medications based on the decision of investigators. Neck pain was assessed by the structured interview at baseline, 1, 3, 6, 12 months of follow-up. Neck pain was classified as mild or severe according to its association with high disability or functional limitation. The proportion of neck pain at baseline and 3 months of follow-up was compared by McNemar"s test and the association with MOH reversal at 3 months was assessed by Chi-square test.

**Results:** A total of 309 patients, 85.1% female, were enrolled. At baseline, 191 patients (61.8%) had neck pain and 78 patients were severe (25.3%). MOH patients with neck pain had more cutaneous allodynia (allodynia symptom checklist-12, 1.0 [0.0-4.0] vs. 0.0 [0.0-2.0], *p*=0.007) and decrease quality of life (migraine-specific quality-of-life, 179.3 [116.0-218.8] vs. 187.9 [137.1-250.0], *p*=0.036) compared to MOH patients without neck pain. A total of 228 patients returned for the 3 months visit and 118 patients (51.7%, *p* < 0.001) had neck pain and 24 patients (10.5%, *p* < 0.001) were severe. At 3 months follow-up, 138 (60.5%) patients were recovery from MOH. The proportion of severe neck pain were 6.5% in patients with recovery from MOH and 16.7% in patients without (*p* = 0.026).

**Conclusion:** About two-thirds of MOH patients had neck pain and one-fourth were severe. Treatment of MOH may reduce neck pain, especially severe neck pain , in patients with MOH.

## P074 Long-term safety and effectiveness of eptinezumab in patients with prior preventive migraine treatment failures

### M. Ashina^1,2^, S. J. Tepper^3^, A. Gendolla^4^, B. Sperling^5^, A. Ettrup^5^, M. K. Josiassen^5^, A. J. Starling^6^

#### ^1^University of Copenhagen, Department of Neurology, Copenhagen, Denmark; ^2^University of Copenhagen, Department of Clinical Medicine, Copenhagen, Denmark; ^3^Dartmouth Hitchcock Medical Center, Lebanon, NH, United States; ^4^Praxis Gendolla, Essen, Germany; ^5^H. Lundbeck A/S, Copenhagen, Denmark; ^6^Mayo Clinic Arizona, Scottsdale, AZ, United States

##### **Correspondence:** A. Ettrup

*The Journal of Headache and Pain 2024,* **25(Suppl 1)**: P074

**Objective:** DELIVER (NCT04418765) evaluated the safety and efficacy of eptinezumab for migraine prevention in patients with prior preventive migraine treatment failures. Here, we report results of the 48-week dose-blinded extension period.

**Methods:** The DELIVER study evaluated eptinezumab 100mg and 300mg vs placebo (pbo) (infusions every 12 weeks) in adults with migraine and 2–4 documented preventive treatment failures. Patients randomized to pbo during the initial 24-week treatment period received eptinezumab 100mg or 300mg in the 48-week extension period; patients initially randomized to eptinezumab continued their assigned dose. Efficacy measures were change from baseline in number of monthly migraine days (MMDs), ≥50% and ≥75% reduction from baseline in MMDs, change from baseline in the 6-item Headache Impact Test (HIT-6), migraine severity, and acute headache medication (AHM) use.

**Results:** After the pbo-controlled period (Weeks 1–24), 782/865 patients (90.4%) completed the 48-week extension. Patients switching from pbo experienced a steep decrease in MMDs over Weeks 25–28 (pbo-to-100mg, –5.8 days; pbo-to-300mg, –7.2 days) compared with the pbo-controlled baseline. All treatment arms had sustained reductions from baseline in MMDs over Weeks 61–72, with >60% of patients experiencing ≥50% MMD reductions and >30% of patients experiencing ≥75% reductions. Patients switched from pbo to eptinezumab had reductions in HIT-6 scores, migraine severity, and AHM use after the first eptinezumab dose. No new safety signals were identified.

**Conclusion:** In DELIVER, the long-term effectiveness and safety of eptinezumab was demonstrated by high completion rates, sustained MMD reductions, and reductions in migraine severity. Similar improvements were observed in patients switched from pbo to eptinezumab in the extension period as well as in those continuing eptinezumab treatment for up to 18 months.

## P075 TAC-tic syndrome secondary to sphenoid sinusitis during pregnancy: a case report

### A. Parejo Olivera^1^, M. Mesa Hernández^1^, N. Valverde Mata^1^, P. Macías Sedas^1^, A. M. Roa Montero^1^, J. M. Ramírez Moreno^1,2^

#### ^1^Servicio Extremeño de Salud. Hospital Universitario de Badajoz., Servicio de Neurología, Badajoz, Spain; ^2^Universidad de Extremadura, Facultad de Medicina, Badajoz, Spain

##### **Correspondence:** A. Parejo Olivera

*The Journal of Headache and Pain 2024,* **25(Suppl 1)**: P075

**Objective:** trigeminal autonomic cephalalgias (TAC) can be associated with trigeminal neuralgia (TN) or TN-like, which is known as TAC-Tic syndrome. The onset may be simultaneous or separated in time and the syndrome is also found in secondary TACs. Causes of secondary TAC-Tics include pituitary adenomas, sinus infection, vascular causes and multiple sclerosis. The incidence of TAC-Tic syndrome is unknown due to the low frequency of cases described. Hemicrania continua associated with TN is extremely uncommon and, in most cases reviewed, the onset was not simultaneous and responded to indomethacin and carbamazepine.

**Methods:** 24-year-old pregnant women with history of hypersensitivity to NSAIDs (non-steroidal anti-inflamatory drugs) and unknown history of headache presented with ptosis, lacrimation and eyelid edema associated with a 3-day continuous right hemichranea and ipsilateral paroxysmal shock-like episodes of pain in the first division of trigeminal nerve with a duration of seconds. She did not present fever at admission nor in previous days. Neurological and ophthalmological examination did not reveal any abnormality. Brain MRI showed sphenoid sinus occupation due to acute sinusitis so intravenous amoxicillin/clavulanic acid and dexamethasone were initiated with complete resolution of symptoms in the first two weeks.

**Conclusion:** most TAC-tic syndromes described in literature are idiopathic, however some of them are related to sinus infection and none of them occurred during pregnancy. Indeed, pregnancy was a challenge in this case because of diagnostic and therapeutic limitations since most drugs used in TAC and TN were contraindicated. The aim of this case report is to highlight the importance of excluding alternative, and potentially severe, causes of headache in pregnant women even when clinical features mimic a primary headache. Consent to publish has been obtained.

**Fig. 1 (Abstract P075):**
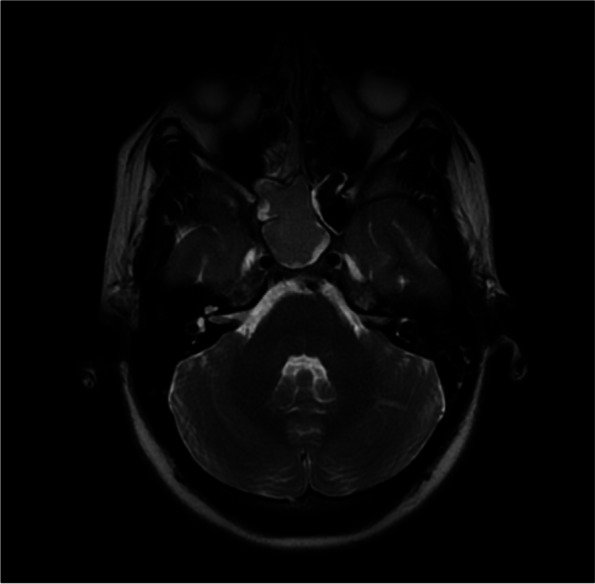
See text for description

**Fig. 2 (Abstract P075):**
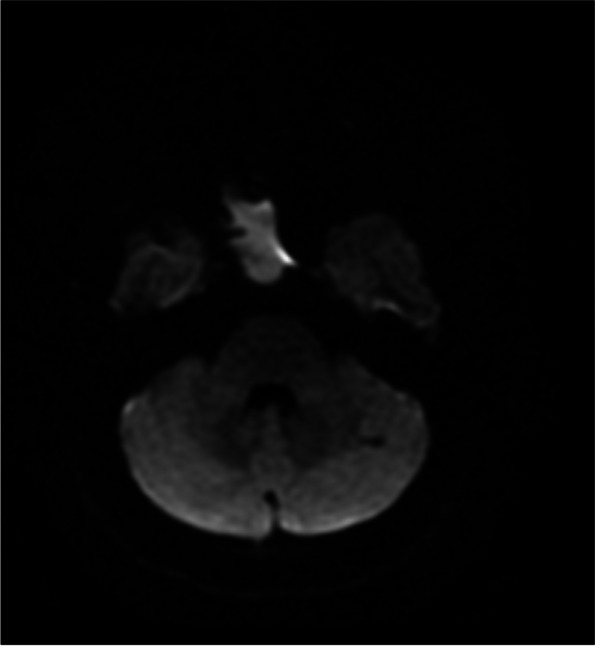
See text for description

## P077 The burden of headache disorders in Norway: a population-based cross-sectional study in adults using the HARDSHIP questionnaire (PopHEAD)

### M. B. Argren^1,2^, H. Lier^1,2^, J. A. Zwart^1,2^, E. Tronvik^1,3^, A. H. Aamodt^1,2^, B. S. Winsvold^1,2^

#### ^1^Norwegian centre for headache research, NorHEAD, Trondheim, Norway; ^2^Oslo University Hospital, Department of Neurology, Oslo, Norway; ^3^NTNU, Trondheim, Norway

##### **Correspondence:** M. B. Argren

*The Journal of Headache and Pain 2024,* **25(Suppl 1)**: P077

**Objective:** Headache disorders are a leading public health concern worldwide and contribute significantly to global disability. Our study aims to provide up-to-date epidemiological headache data for the adult population in Norway. The primary objective is to estimate the 1-year prevalence and burden of episodic tension type headache (TTH), chronic TTH, migraine and medication overuse headache in the adult Norwegian population. Secondary objectives are to describe demographic variations in headache prevalence.

**Methods:** We conducted a population-based cross-sectional study in Vestfold and Telemark County, Norway, in early 2023. A random sample of 31,500 individuals aged 18 to 70 years was invited to participate. The study applied a digital version of the Headache-Attributed Restriction, Disability, Social Handicap and Impaired Participation (HARDSHIP) questionnaire translated into Norwegian using the *lifting the burden* translation protocol for hybrid documents. It was distributed through *HelseNorge*, a digital portal for health services. The questionnaire assesses headache prevalence, pain intensity, attack frequency, comorbidities, medication use, and productivity loss. ICHD-3 diagnostic criteria were applied. A random sub-sample (N=500) were contacted by phone to validate the diagnoses.

**Results:** In total 8,217 individuals (3,331 men and 4,886 women) responded, with an average age of 47.7 years. The majority of both men (74.6%) and women (89.5%) reported headache in the past year. Migraines were reported by 42.2% overall (46.2% of men and 39.5% of women), which is substantially higher than the expected prevalence, suggesting an over-representation of individuals with migraine among participants. Data on burden will be presented.

**Conclusion:** This study provided updated data on headache burden in Norway. The data will be analyzed and presented in the upcoming EHC.

## P078 Estimated lost productivity costs amongst National Health Service (NHS) staff with migraine

### G. Shirley^1^, S. Awad^2^, X. Y. Lee^2^

#### ^1^Lundbeck Limited, Watford, United Kingdom; ^2^H. Lundbeck A/S, Valby, Denmark

##### **Correspondence:** G. Shirley

*The Journal of Headache and Pain 2024,* **25(Suppl 1)**: P078

**Objective:** Migraine is associated with significant lost productivity costs. The UK NHS is the largest European employer and is currently experiencing a chronic workforce crisis. Eptinezumab is an calcitonin gene-related peptide antagonist shown to reduce absenteeism and presenteeism in migraine patients. We aim to estimate migraine-related lost productivity costs in NHS employees, and potential productivity gains from treating them with eptinezumab.

**Methods:** Published migraine prevalence estimates and the proportion of patients who failed ≥2 or ≥3 prior migraine preventive treatments were applied to the total number of NHS employees (1.2 million) to estimate the number of employees with chronic or episodic migraine who may benefit from eptinezumab. WPAI baseline scores from the DELIVER trial were used to estimate the number of days lost to absenteeism and presenteeism (costed as 50% of full productivity) per year in patients without preventive treatment. Mean annual NHS salary was used to cost lost productivity time. In alternative scenarios which assume these patients were treated with eptinezumab, DELIVER WPAI week 24 outcomes, weighted by w12 treatment response rates, were used to estimate reductions in absenteeism, presenteeism and associated costs.

**Results:** The annual cost of absenteeism and presenteeism in NHS employees untreated with preventives and failed ≥2 preventives were estimated to be £172 million and £389 million, respectively. With eptinezumab, estimated costs were £92 million (absenteeism) and £268 million (presenteeism). For untreated patients who failed ≥3 preventives, the costs of absenteeism and presenteeism were estimated at £94 million and £216 million, respectively. With eptinezumab, estimated costs were £75 million (absenteeism) and £161 million (presenteeism).

**Conclusion:** Treating NHS migraine employees with eptinezumab could lead to annual productivity gains of ~£200 million (failed ≥2 preventives) and ~£70 million (failed ≥3 preventives).

## P079 Efficacy of OnabotulinumtoxinA among diverse racial/ethnic groups: post-hoc analysis of the phase 4 COMPEL trial

### A. Blumenfeld^1^, L. Charleston IV^2^, K. Sommer^3^, H. L. O’Brien^4^

#### ^1^The Los Angeles Headache Center, Los Angeles, CA, United States; ^2^Michigan State University College of Human Medicine, Department of Neurology and Ophthalmology, East Lansing, MI, United States; ^3^AbbVie, Irvine, CA, United States; ^4^University of Cincinnati College of Medicine, Headache Center of Hope, Cincinnati, OH, United States

##### **Correspondence:** K. Sommer

*The Journal of Headache and Pain 2024,* **25(Suppl 1)**: P079

**Objective:** This study analyzed efficacy of migraine-preventive medications among different racial/ethnic groups.

**Methods:** Single-arm, open-label COMPEL study enrolled adults with chronic migraine (CM) receiving onabotulinumtoxinA (onabotA) 155U every 12 weeks (9 treatments over 108 weeks). Non-Hispanic Whites (NHW) were comparison group. Change in number of monthly headache days (MHDs) at each visit, 6-item Headache Impact Test (HIT-6) total score, Migraine Disability Assessment (MIDAS) score, and Migraine-Specific Quality-of-Life Questionnaire (MSQ) v2.1 score vs. baseline were evaluated.

**Results:** Intent-to-treat analysis population included a total of 715 patients (*n*=581 NHW; *n*=89 Asian; *n*=41 African American; *n*=4 Pacific Islander/Alaska Native); 373 patients completed the 108-week study, (*n*=288 NHW; *n*=60 Asian; *n*=21 African American; *n*=4 Pacific Islander/Alaska Native). The Asian population had higher proportion of males, lower body mass index, and later age of migraine onset than NHW and African American populations and the African American population had earlier onset of migraine than the Asian population and higher incidence of sleep disorders and head trauma than NHW and Asian populations. After 155U of onabotA every 12 weeks, all racial/ethnic groups showed reductions in MHDs at all time points (all, *P*<0.0001; Table). All racial/ethnic groups showed similar proportions of participants who achieved ≥50% reduction in MHDs from baseline at all time points (Figure). Each racial/ethnic group showed reductions in HIT-6(*P*<0.01), MIDAS(*P*<0.05), and MSQ 2.1(*P*<0.05) Role Function Restrictive scores. Treatment with onabotA was safe and effective based on mean reductions in MHDs and proportion of ≥50% responders across various groups. Common adverse events (>2% across all groups) were neck pain, eyelid ptosis, musculoskeletal stiffness, and injection site pain.

**Conclusion:** OnabotA was safe and effective for preventive treatment of CM among diverse racial/ethnic groups.

**Fig. 1 (Abstract P079):**
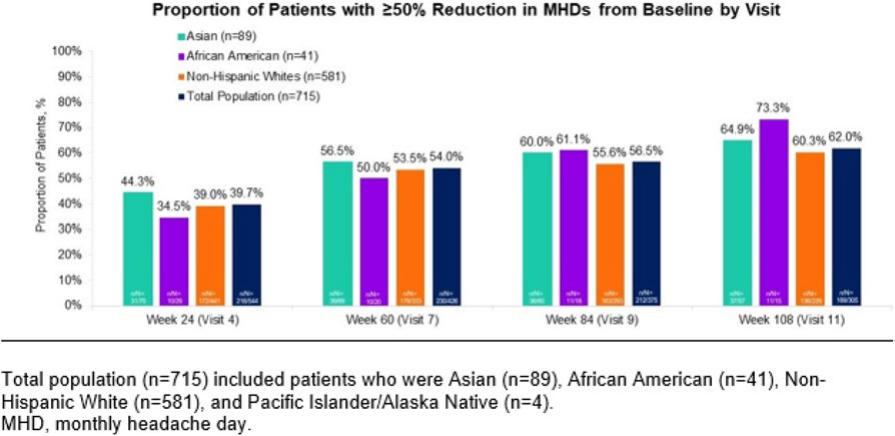
Proportion of patients with ≥50% reduction in MHDs from baseline by visit

**Table 1 (Abstract P079):**
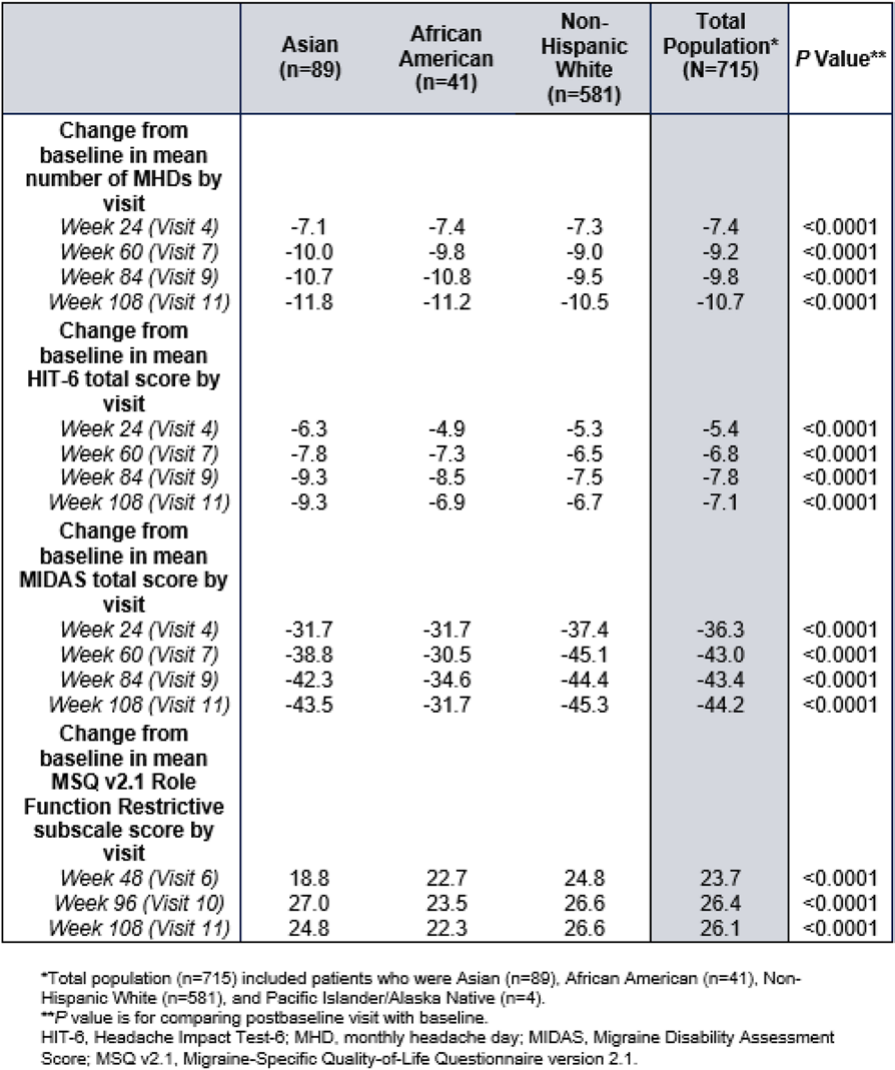
Change From Baseline in Mean Number of MHDs, HIT-6 Score, MIDAS Score, and MSQ v2.1 Role Function Restrictive Score by Visit, Analysis Population

## P080 To scan or not to scan: Are we missing tumours in headache patients?

### S. Samaha, M. Ghadiri-Sani

#### The Walton Centre NHS Foundation Trust, Neurology, Liverpool, United Kingdom

*The Journal of Headache and Pain 2024,* **25(Suppl 1)**: P080

**Objective:** To evaluate radiology referrals from regional refractory headache clinics (RRHC) at the Walton Centre NHS trust. We looked at demographics, indication, results and their impact on decision making, in accordance with local and NICE guidelines.

**Methods:** Retrospective review of 140 patients referred for neuroimaging from RRHC from May to October 2022.

**Results:** Out of 140 patients, 29 were excluded as these were not headache-related requests. The indications were benchmarked against national and local guidelines. Our compliance with national and local guidance represented 71%.

15% of the patients attending the RRHC were referred for neuroimaging, with a female predominance of 69% and a mean age around 48 (range 21-85).

52% had plain and 32% had contrast enhanced magnetic resonance imaging, out of which 30% (10% of total) were angiographic. 16% had computerised topographic imaging (CT), out of which 17% (3% of the total) were angiographic and 8% (2% of the total) venography.

There was significant abnormality in 26% with a 72% female predominance. 41% of the abnormal reports were in 41-50 age group. 41% had vascular abnormalities out of which 8% were established strokes (incidental), 50% showed neurovascular compromises, in keeping with the clinical suspicion and 42% other vascular abnormalities, not requiring intervention. 41% had features of intracranial pressure abnormalities, again, in keeping with clinical suspicion. 1 patient had high grade glioma and surgical referral was made.

48.6% had incidental findings such as small vessel disease.

**Conclusion:** Overall, we are compliant with the imaging guidelines, however, the yield of the scans remains generally low, with only one patient with an incidental high-grade glioma. Older patients with a clinical suspicion of pressure abnormalities or neurovascular compromise in specific headache disorders, had the highest and most clinically relevant findings.

## P082 Clinico-Epidemiological features of persistent idiopathic facial pain

### G. Chakhava^1,2^, I. Rukhadze^3^, O. Koniashvili^3^, M. Demuria^1,2^

#### ^1^Multiprofile Clinic Consilium Medulla, Neurology, Tbilisi, Georgia; ^2^Georgian Association of Medical Specialties, Tbilisi, Georgia; ^3^N. Kipshidze Central University Clinic of Tbilisi State Medical University, Neurology, Tbilisi, Georgia

##### **Correspondence:** G. Chakhava

*The Journal of Headache and Pain 2024,* **25(Suppl 1)**: P082

**Objective:** Contrary to trigeminal neuralgia, this condition does not involve neurovascular compression. Our study's objectives were to look into particular clinical and epidemiological aspects of PIFP as well as the condition's clinical progression.

**Methods:** We investigated retrospectively 17 patients who had been given PIFP diagnoses. The International Headache Society's International Classification of Headache Disorders was used to make the diagnosis.

**Results:** Eighty-eight percent of the patients (*n*=15) with atypical facial pain diagnoses were female, and just two patients were male. Most of the patients' discomfort (82,3%; *n*=14) was restricted to the left part of the face. Nine of the patients reported right-sided discomfort, and five reported left-sided pain. 17.6% of the remaining patients (*n*=3) exhibited bilateral pain distribution. The majority of patients (41%; *n* = 9) claimed that their pain was mostly in the maxillary, mandibular, or temporal region, while others reported frontal, orbital, parietal, occipital, gingival, or nasal distribution. 64 percent of the patients experienced mild pain, while the remaining patients claimed acute pain. The symptoms began between 8 months and 4 years ago, with a mean of 17.8 months (standard deviation: 10.4).

**Conclusion:** The symptoms of PIFP are severe, episodic, chronic, and frequently recurrent. They have a negative effect on the patient's quality of life, mood, and productivity. Since PIFP frequently exhibits treatment resistance, management can be quite difficult for healthcare professionals.

**Fig. 1 (Abstract P082):**
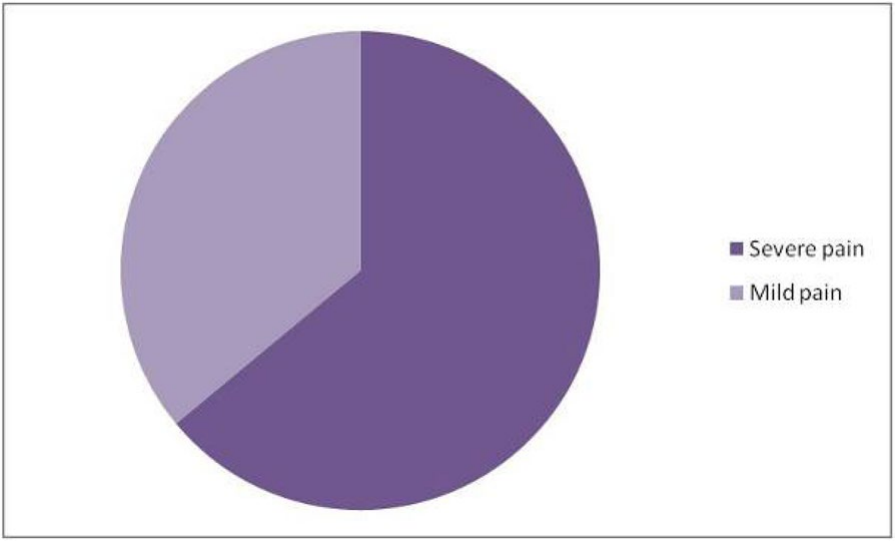
See text for description

**Fig. 2 (Abstract P082):**
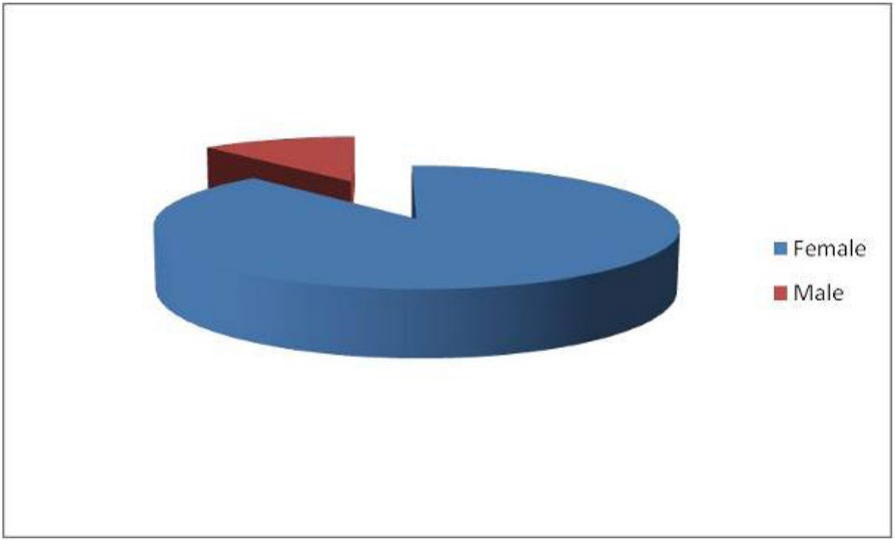
See text for description

**Fig. 3 (Abstract P082):**
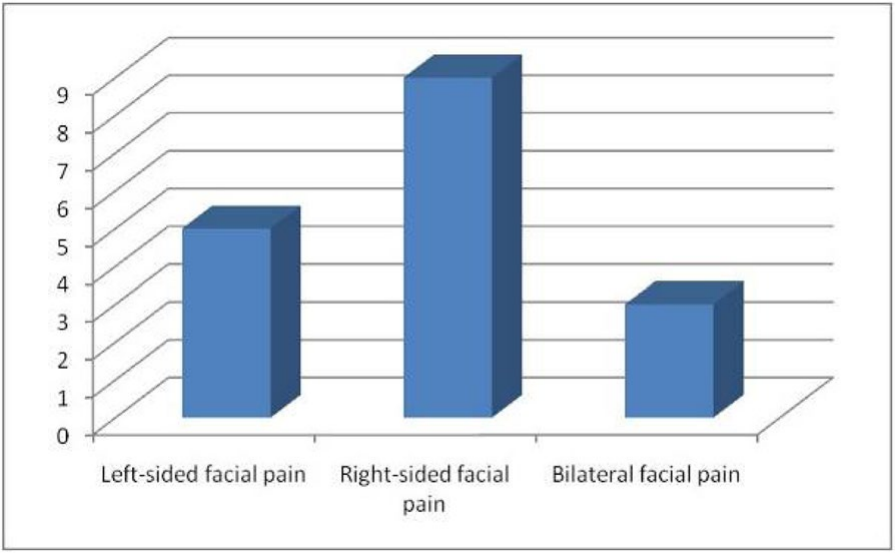
See text for description

## P083 Exploring the experience of neck pain in individuals with episodic migraine

### A. Blumenfeld^1^, M. Mordin^2^, K. Kosa^2^, C. Castro^2^, J. Stokes^3^, D. Shah^3^, D. C. Buse^4^

#### ^1^The Los Angeles Headache Center, Los Angeles, CA, United States; ^2^RTI Health Solutions, Research Triangle Park, NC, United States; ^3^AbbVie, Madison, NJ, United States; ^4^Albert Einstein College of Medicine, Bronx, MA, United States

##### **Correspondence:** D. Shah

*The Journal of Headache and Pain 2024,* **25(Suppl 1)**: P083

**Objective:** To explore the experience of neck pain (NP) among people with episodic migraine (EM) across migraine phases. NP has been documented as a migraine symptom that is associated with increased disability. However, there are limited data on the experience of NP from the perspective of individuals with migraine.

**Methods:** A targeted PubMed literature review evaluated the relationship between NP and EM. Adults with clinician-diagnosed EM (5-14 days/month) were recruited to participate in concept elicitation interviews. Trained researchers used a semi-structured interview guide based on findings from the literature review. Open-ended questions were asked to elicit spontaneous reports of the NP experience associated with migraine and its temporal occurrence. Targeted questions were used if concepts were not raised spontaneously.

**Results:** Findings from the literature review demonstrated that NP is highly prevalent before, during, and after the headache phase of migraine and was associated with increased Neck Disability Index score. Twenty participants completed the qualitative interviews; 13 (65.0%) reported NP related to migraine (pre-headache [*n*=12]; during headache [*n*=13]; post-headache [*n*=6]). Duration of NP ranged from a few hours to 1 day. NP was reported as the most bothersome symptom by 2 (10.0%) participants. Most participants described their NP as "tense," "stiff," or "tight" (*n*=8; 61.5%); others described their NP as a "dull ache" (*n*=4; 30.8%), a "kink" or "pulled muscle" (*n*=3; 23.1%), "sore" (*n*=2; 15.4%), or a "shooting pain" (*n*=1; 7.7%).

**Conclusion:** Most participants reported NP associated with migraine. NP occurred before, during, and/or after the headache phase, and was predominantly described as "tense," "stiff," or "tight." These results confirm that NP is a bothersome symptom for individuals with EM and may be an important outcome of effective treatment.

## P084 How species differences help in further understanding the mechanism of action of the gepants

### R. van Drie, D. Boucherie, T. de Vries, A. H. J. Danser, A. MaassenVanDenBrink

#### Erasmus MC University Medical Center, Division of Vascular Medicine and Pharmacology, Department of Internal Medicine, Rotterdam, Netherlands

##### **Correspondence:** R. van Drie

*The Journal of Headache and Pain 2024,* **25(Suppl 1)**: P084

**Objective:** Calcitonin gene-related peptide (CGRP) is a potent vasodilator involved in the pathophysiology of migraine. Gepants targeting the CGRP receptor have emerged as effective treatments. However, the pharmacological characteristics remain incompletely understood. In this study, we used variations in CGRP-blocking potency among gepants between species to study binding locations at the canonical CGRP receptor.

**Methods:** In porcine small coronary artery segments, concentration response curves to human α-CGRP (10^-10^ – 10^-6^ M), in the absence and presence of a gepant, were constructed using Mulvany myography. The potency of atogepant (*n*=7), olcegepant (*n*=6), rimegepant (*n*=8), telcagepant (*n*=6), ubrogepant (*n*=5) and zavegepant (*n*=8) was determined by calculating pK_b_ values. These were compared to earlier data from our lab obtained in human small coronary artery segments. Furthermore, amino acid sequence analysis was performed for the CGRP receptor components using online available sequences in the UniProt.org database.

**Results:** Potency comparison of gepants in porcine and human arteries revealed dissimilar potency differences between the species for the various gepants. While atogepant was equipotent in human and porcine tissue (pK^b^ 8.64^1^ and 8.77, respectively), zavegepant lacked CGRP-blocking activity in porcine small coronary artery segments, while it was potent in human tissue (pK_b_ 9.15^2^). For the other gepants, potency differences were 1 - 4 logarithmic units. Amino acid sequence analysis yielded a high homology between porcine and human calcitonin-like receptor (CLR, 93.1%), but only moderated homology of the receptor-amplifying protein 1 (RAMP1, 68.2%). From the published amino acids of CLR and RAMP1 interacting with gepant molecules, two amino acids are not conserved.

**Conclusion:** However, protein homology data do not explain the differences in gepant potency and consequently, our results suggest that the various gepants bind to different parts of the canonical CGRP receptor complex. Further understanding these divergent effects will enable optimalization of migraine treatment by expanding therapeutic treatment options.

^1^ Chan, et al. J Pharmacol Exp Ther. 2010

^2^ Boucherie, unpubl. data 2022

## P085 Rethinking Migraine: will shifting the emphasis towards preventative treatment of migraine, using new CGRP-targeting medicines, be of added value to society from a health economics perspective?

### M. Tinelli^1^, V. Quoidbach^2^

#### ^1^London School of Economics and Political Science, CPEC, London, United Kingdom; ^2^European Brain Council, Brussels, Belgium

*The Journal of Headache and Pain 2024,* **25(Suppl 1)**: P085

**Objective:** The calcitonin gene-related peptide [CGRP] receptor antagonists are new therapies with demonstrated efficacy both in treatment and prevention of migraine attacks. Their place among the range of treatments needs to be established. CGRP-targeting medicines include gepants (small molecular antagonists) and anti-CGRP antibodies.

Our objective is to assess the evidence of cost-effectiveness of CGRP-targeting medicines in migraine prevention (alone or in combination with other approaches) as a prerequisite for evaluating the marginal cost-effectiveness of introducing these CGRP-targeting medicines into the range of preventative measures, both pharmacological and non-pharmacological.

**Methods:** We will first conduct scoping literature reviews of (a) cost-of-illness of episodic (EM) and chronic (CM) migraine and (b) cost-effectiveness of preventative treatments for EM and CM, with particular focus on CGRP-targeting medicines. Subsequently we will conduct decision analytic modelling.

**Results:** Preliminary results from the literature reviews and our plans for the economic modelling will be presented.

**Conclusion:** In clinical trials, not all patients have benefited from the preventative effects of CGRP-targeting medicines. At the same time, these newly-developed drugs are relatively costly. In these circumstances, the public-health benefits of introducing them are uncertain. These and further studies are needed to understand whether, and by how much, CGRP-targeting medicines, when optimally introduced among other therapeutic options, will improve outcomes at what increase in costs.

## P086 A head-to-head observational cohort study on the efficacy and safety of monoclonal antibodies against calcitonin gene–related peptide for chronic and episodic migraine

### S. Braca, C. V. Russo, F. Saccà, A. Stornaiuolo, A. Miele, R. De Simone

#### Università degli Studi di Napoli "Federico II", Department of Neurological Sciences, Reproductive and Odontostomatological Sciences, University Federico II, Naples, Italy, Naples, Italy

##### **Correspondence:** S. Braca

*The Journal of Headache and Pain 2024,* **25(Suppl 1)**: P086

**Objective:** Monoclonal antibodies (mAbs) targeting the calcitonin gene–related peptide (CGRP) pathway have been tested in several clinical trials for both episodic and chronic migraine, showing high effectiveness and safety; however, there are no prospective real-world studies intending to compare their efficacy and safety. This study intends to compare galcanezumab, fremanezumab and erenumab for the treatment of chronic and episodic migraine.

**Methods:** This is a prospective observational cohort study comparing the effectiveness and safety profiles of galcanezumab, fremanezumab, and erenumab for the treatment of chronic and episodic migraine. We enrolled 140 patients at the Headache Centre of University Federico II of Naples. Framenezumab, erenumab, or galcanezumab were administered for 12 months. The mean monthly days with headache, Migraine Disability Assessment (MIDAS) score, and adverse events were evaluated at baseline and every 3 months by reviewing standardized paper patient headache diaries.

**Results:** We found a mean reduction of migraine monthly days from baseline of −12.0 (−9.8, −14.1) in the galcanezumab group, −12.3 (−10.2, −14.3) in the fremanezumab group, and −10.8 (−8.5, −13.1) in the erenumab group (for all, *p* < 0.001). We found a mean reduction of MIDAS score of −32.6 (−26.6, −38.5) in the galcanezumab group, −33.4 (−28.0, −38.9) in the fremanezumab group, and −29.2 (−23.0, −35.4) in the erenumab group (for all, *p* < 0.001). We found no significant differences between mAbs in the reduction of mean monthly days with headache and MIDAS score. We found a more rapid effect of galcanezumab and erenumab compared to fremanezumab in medication overuse headache patients after 3 months of treatment (−10.8 and −11.1 vs. −4.0 days; *p* = 0.029).

**Conclusion:** Our results confirm the therapeutic benefits of anti-CGRP mAbs. There is no evidence that suggests that one antibody may be superior to the others in terms of effectiveness, both in chronic and episodic patients.

**Table 1 (Abstract P086):**
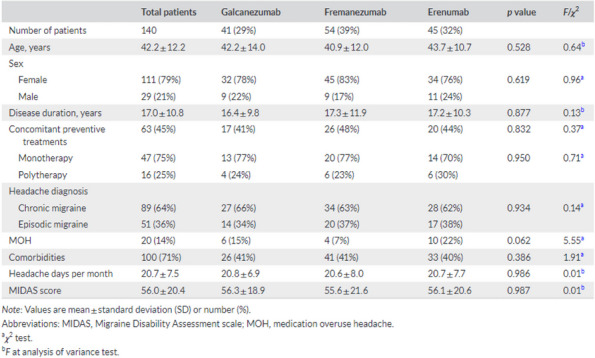
Baseline demographic and clinical characteristics

**Fig. 1 (Abstract P086):**
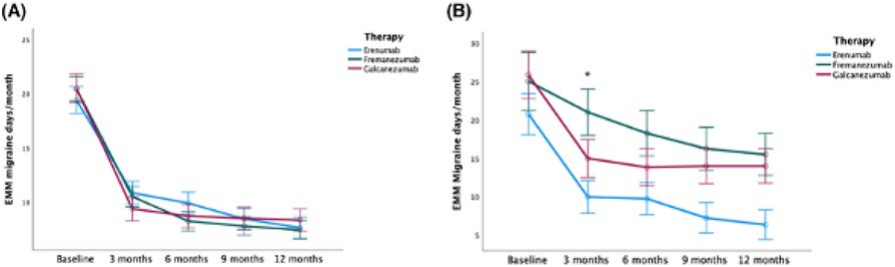
See text for description

## P087 Long-term effectiveness of monoclonal anti-CGRP antibodies in patients with migraine in real life

### C. Pérez Prol^1^, C. Espinoza Vinces^1^, R. Villino Rodríguez^1^, A. Atorrasagasti Villar^1^, M. Gimeno Rodríguez^1^, B. Benítez Martínez^2^, M. Beistegui Sarobe^1^, M. Sánchez del Río^1^, P. Irimia Sieira^1^

#### ^1^Clinica Universidad de Navarra, Department of Neurology, Pamplona, Spain; ^2^Universidad De La Sabana / Hospital Occidente de Kennedy, Neurology, Chía, Colombia

##### **Correspondence:** C. Espinoza Vinces

*The Journal of Headache and Pain 2024,* **25(Suppl 1)**: P087

**Objective:** To describe the long-term clinical response in patients with high-frequency episodic (HFEM) and chronic migraine (CM), after 24 months of treatment with anti-CGRP monoclonal antibodies (anti-CGRP mAbs).

**Methods:** Retrospective observational study of a cohort of 96 patients with migraine that received at least one dose of any anti-CGRP monoclonal antibody. Twenty-seven adults (23 women) who continued treatment for at least 2 years were analyzed. All patients had been unsuccessfully treated with ≥3 oral preventive medication classes. Of all patients, 2 presented high-frequency episodic migraine and 25 chronic migraine. Changes in monthly migraine days (MMDs), monthly headache days (MHDs), or the switch to another anti-CGRP monoclonal antibody, as well as the clinical response quantified as a reduction of at least 30% of migraine days per month were recorded. Side effects were also registered.

**Results:** Clinical improvement quantified as a reduction of at least 30% of migraine days per month was achieved in 67% (*n*=18), of which 83% (*n*=15) of patients were women. The remaining 33% (*n*=9) observed some improvement but did not achieve a sustained reduction of at least 30% of migraine days per month after two years. 16 patients (59%) maintained treatment with Erenumab at two years, 41% (*n*=11) of the patients switched to other monoclonal antibodies, and seven patients showed improvement after switching. No relevant side effects were recorded, only constipation in 10% of patients.

**Conclusion:** Our results suggest that long-term (2 years) anti-CGRP mAbs treatment provides sustained effectiveness, safety, and tolerability in real-life patients with HFEM or CM, and one quarter of patients required switching to obtain long-term benefit.

## P089 Comparing the safety, efficacy and tolerability of lasmiditan 100 mg and 200 mg in acute migraine treatment: a systematic review, meta-analysis and meta-regression of randomized controlled trials

### A. Cyntia Lima Fonseca Rodrigues^1,2^, B. Kraychete^3^, M. A. Castro Olyntho Jr.^4^, J. Miranda Figueredo^5^, V. Botelho Lorenzo^6^, L. Ferreira Pereira^7^, R. Turatti Miranda^8^, V. Cruz Majdalani^9^, S. Bernardo Lança^10^

#### ^1^Positivo University, Department of Medicine, Curitiba, Brazil; ^2^Anhembi Morumbi University, Department of Statistics, Curitiba, Brazil; ^3^Children’s Hospital Los Angeles, Los Angeles, CA, United States; ^4^D'olhos DAY Hospital, Sao Jose do Rio Preto, Brazil; ^5^Pontificia Universidade Catolica de Sao Paulo, Department of Medicine, Sorocaba, Brazil; ^6^Bahiana School of Medicine and Public Health, Department of Medicine, Salvador, Brazil; ^7^Federal University of Amazonas, Department of Clinical Surgery Otorhinolaryngology, Manaus, Brazil; ^8^Barbacena School of Medicine, Department of Medicine, Barbacena, Brazil; ^9^Hospital Aeroporto, Lauro de Freitas; ^10^Federal University of Sao Paulo, Sao Paulo

##### **Correspondence:** A. Cyntia Lima Fonseca Rodrigues

*The Journal of Headache and Pain 2024,* **25(Suppl 1)**: P089

**Objective:** This study aims to assess the safety, efficacy and tolerability of lasmiditan (LTN) 100 mg versus 200 mg for acute migraine.

**Methods:** PubMed, Embase, Web of Science and Ovid databases were searched through June 2023. Statistical analysis was performed using R version 4.3.1. Random-effects meta-analysis were estimated using the inverse variance with Mantel-Haenszel method. The outcomes assessed were headache freedom (HF) and headache relief (HR) at 2 h and treatment-emergent adverse events (TEAEs).

**Results:** We included 9 studies involving 6,919 patients. LTN 100 mg demonstrated a statistically significant increase in HF (OR 0.82, 95% CI 0.73 to 0.91, *p*<0.001, I^2^ = 0%), and a significant reduction in TEAEs (RR 0.87, 95% CI 0.82 to 0.92, *p*<0.001, I^2^ = 0%) compared with LTN 200 mg. However, it did not significantly affect HR (OR 1.01, 95% CI 0.89 to 1.15, *p*=0.845, I^2^ = 0%). The meta-regression evaluated the statistical impact of the migraine frequency/ attacks in the past 3 months in PF, which was (estimate 0.024, *p*=0.9893, 95% CI -0.3418 to 0.3465). Sensitivity analysis was performed for HF, no single study was identified as a remarkably influential study. Graphical distribution of the funnel plot shows no evidence of asymmetry in HF.

**Conclusion:** The meta-analysis found that LTN 100 mg shows a statistically significant increase in HF and a significant reduction in TEAEs when compared with a LTN 200 mg. The meta-regression demonstrated that the frequency of migraines or attacks in the past 3 months did not significantly influence HF. Given the consistency of results across studies, this analysis suggests that LTN 100 mg may be a more effective and safer dose than LTN 200 mg for managing headaches.

## P090 Subarachnoid Aneurysmal Hemorrhage & migraine: prevalence, characteristics and risk of developing acute and persistent headache

### L. Gómez-Dabó, V. J. Gallardo, D. Campos-Fernández, M. Rodrigo-Gisbert, M. Iza-Achutegui, A. Alpuente, M. Torres-Ferrús, E. Caronna, P. Pozo-Rosich

#### Vall d’Hebron Hospital & Research Institute, Universitat Autonoma de Barcelona, Neurology, Barcelona, Spain

##### **Correspondence:** L. Gómez-Dabó

*The Journal of Headache and Pain 2024,* **25(Suppl 1)**: P090

**Objective:** 1. To study the relationship between migraine and aneurysmal Subarachnoid Hemorrhage (aSAH). 2.To determine prevalence, characteristics, and prognostic factors of Acute and Persistent Headache (AH, PH) attributed to aSAH.

**Methods:** Retrospective study, including all aSAH cases attended in a tertiary hospital between Jan/2019-Sep/2021. We collected demographics, migraine history, clinical and neuroimaging data, prognostic scales (WFNS, APACHE-2, mFisher, VASOGRADE) and functional outcomes (mRS) at 3 months from medical charts. Patients who survived were phone-interviewed in May/2023 to assess AH and PH. We 1) compared aSAH patients with/without history of migraine and, 2) analyzed factors associated with AH and PH.

**Results:** 130 patients included with aSAH (mean age 60.0±13.6y.o.; 62.3% women), 36.9% (48/130) had died. 24.61% (32/130) had history of migraine. Migraine was associated with less severity of aSAH (WFNS,*p*<0.03), lower risk of mortality (APACHE-2,*p*<0.04), delayed cerebral ischemia (VASOGRADE,*p*<0.016) and less proportions of intracranial hypertension (9.4%vs42.2%;*p*<0.001). At 3 months, in the migraine group, higher proportion of patients survived (84.4%vs60.9%;*p*<0.001), with better functionality (mRS<3;71%vs42.2%;*p*<0.004). Excluding deceased patients, 83.9% (68/81) reported AH, which was associated with younger age (57.6vs64.3y.o;*p*<0.03), migraine history (33%vs7.7%;*p*<0.001), greater motor impairment (87.8%vs59.4%;*p*<0.01), higher scores in Glasgow Outcome Scale (12.5vs9.5;*p*<0.001), and lower scores in Hunt&Hess, WFNS and mFisher (*p*<0.001). At median time of 4 years of follow-up, 41.9% (34/81) presented PH, with migraine history as the only associated factor (*p*<0.001). Of them, 55.88% (19/34) reported a moderate-severe impact of PH in daily life.

**Conclusion:** aSAH affects women more than men. In aSAH, migraine prevalence is higher than in the general population and is associated with better prognosis, but it is a risk factor for presenting PH.

## P091 Factors associated with migraine interictal burden severity: results from the OVERCOME (EU) study

### S. Gonderten^1^, A. Sheikhi Mehrabadi^1^, G. Dell'Agnello^1^, S. Evers^2^, J. Pascual^3^, D. Novick^1^

#### ^1^Eli Lilly and Company, Indianapolis, IN, United States; ^2^University of Münster, Münster, Germany; ^3^University Hospital Marqués de Valdecilla, Santander, Spain

##### **Correspondence:** S. Gonderten

*The Journal of Headache and Pain 2024,* **25(Suppl 1)**: P091

**Objective:** To identify factors associated with interictal burden (IIB) severity in people with migraine.

**Methods:** The ObserVational survey of the Epidemiology, tReatment and Care Of MigrainE – Europe [OVERCOME (EU)], conducted in Germany and Spain, is part of an overarching study program that also includes the US and Japan. It was completed by 20,756 adults with migraine, defined as per the Modified International Classification of Headache Disorders-3 screening criteria or by self-report of migraine diagnosis by a physician, and that reported having a headache, that was not due to hangover or illness, or migraine in the last 12 months. Patients were grouped based on the IIB severity assessed by Migraine Interictal Burden Scale (MIBS-4) score as no/mild (0-2) vs moderate/severe (3-5+). The association between IIB severity and independent factors was investigated using univariate analysis, random forest, Lasso regression and binary logistic regression. Results are presented as odds ratios (OR) [95% CI].

**Results:** Among all respondents (*N*=20756), 57.5% (*n*= 11927) had moderate/severe IIB. Non-university education (vs university 0.878 [0.821-0.940]), no allodynia (0-2) (vs yes [3+], 0.621[0.581-0.665]), no or little migraine disability measured by MIDAS (0-5) (vs severe [21+], 0.368 (0.331-0.410), higher MSQ Role Function Restrictive (RFR) score (0.977 [0.975-0.978]), not currently taking any preventive medication (vs yes, 0.730 [0.564-0.945]), no anti-depressant, anti-seizure, and cardiovascular treatment (vs yes, 0.404 [0.321-0.510], 0.465 [0.370-0.586], 0.447 [0.354-0.566], respectively), female gender (vs male, 0.613 [0.571-0.659]) were associated with lower likelihood of being in the moderate/severe IIB.

**Conclusion:** The burden of migraine expands beyond the attacks and the IIB severity is associated with greater disability during the attacks (MIDAS), poorer QoL (MSQ) and higher likelihood of taking preventives.

## P092 The burden of migraine in the United Kingdom: a retrospective cross-sectional study

### G. O'Neil

#### Pfizer, Medical Affairs, Tadworth, United Kingdom

*The Journal of Headache and Pain 2024,* **25(Suppl 1)**: P091

**Objective:** To evaluate patient-reported outcomes relating to Healthcare Resource Utilisation (HCRU), Quality of Life (QoL), and work productivity and activity impairment (WPAI) from people with migraine compared to those without migraine in the United Kingdom (UK).

**Methods:** A retrospective cross-sectional study from the 2020 National Health and Wellness Survey (NHWS, Cerner Enviza) captured patient-reported data from migraine respondents who resided in the UK. The migraine subgroup required a physician diagnosed their migraine and ≥1 migraine headache day in the past 30 days. Matching was conducting by generating a propensity score of having migraine vs. no migraine based on 11 demographic and clinical characteristics in a 1:2 (case:control) ratio.

**Results:** A total of 2,073 respondents were eligible for inclusion in the analyses, including *n*=691 with diagnosed migraine and *n*=1,382 without migraine. Analyses revealed significant differences (all *p*<0.001) between the matched cohort with migraine versus without migraine in WPAI, QoL and HCRU. Mean productivity impairment was greater in the migraine group than the group without migraine (see Figure 1). Absenteeism impairment was nearly double in migraine cohort (16.71%, SD 27.81%) compared to the cohort without migraine (9.62%, SD 23.94%). EQ-5D index scores showed greater burden in the migraine cohort compared to the cohort without migraine (0.568 v 0.751). The mean number of general practitioner visits in the past 6 months was more than double in the migraine cohort (2.46, SD 3.66) compared to the cohort without migraine (1.20, SD 2.13). The mean number of emergency room visits in the past 6 months was also double in the migraine group (0.58, SD 1.30) compared to the matched controls (0.29, SD 0.99).

**Conclusion:** After matching for demographic and clinical variables, people with migraine in the UK had significantly higher work impairment, lower QoL, and greater HCRU compared to people without migraine.

**Fig. 1 (Abstract P092):**
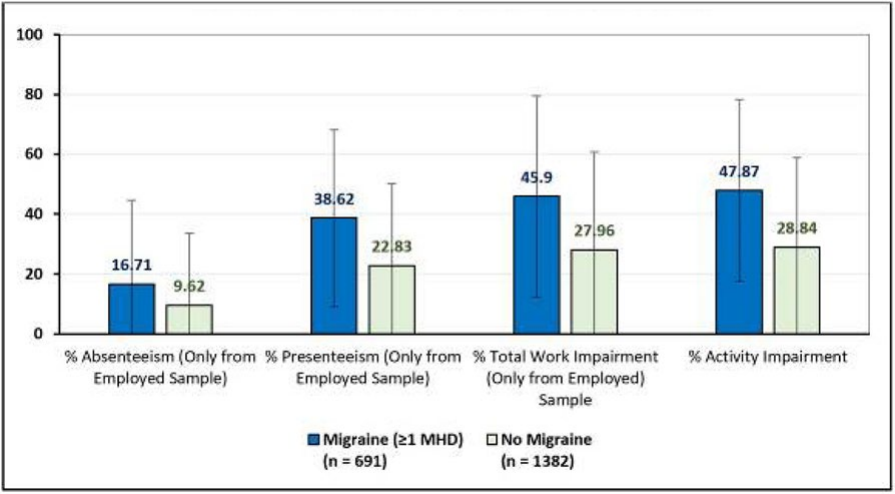
Patient-Reported Work Productivity And Activity Impairment in People with Migraine compared to match controls

## P093 Mimics and chameleons of headache attributed to Influenza infection: a prospective cohort study

### L. Santana-Lopez^1^, J. E. Lozano Alonso^2^, A. Ordax Diez^3^, I. Sanz Muñoz^4^, Y. González Osorio^1^, S. Rojo Rello^5^, J. Sánchez Martínez^6^, Á. Sierra-Mencía^1^, A. Recío García^1^, Á. L. Guerrero Peral^1^, D. García Azorín^1^

#### ^1^Hospital Clínico Universitario de Valladolid, Neurology, Valladolid, Spain; ^2^Dirección General de Salud Pública e Investigación, Desarrollo e Innovación, Valladolid, Spain; ^3^Consejería de Sanidad de Castilla y León, Valladolid, Spain; ^4^Centro Nacional de Gripe de Valladolid, Valladolid, Spain; ^5^Fundación General de la Universidad de Valladolid, Valladolid, Spain; ^6^Fundacion Instituto de Estudios de Ciencias de la Salud de Castilla y Leon, Centro Nacional de Gripe, Valladolid, Spain

##### **Correspondence:** Á. L. Guerrero Peral, D. García Azorín

*The Journal of Headache and Pain 2024,* **25(Suppl 1)**: P093

**Objective:** To evaluate the similarities between the headache phenotype of patients with acute *Influenza* infection and primary headache disorders.

**Methods:** A prospective cohort study was conducted. The full study protocol is available (NCT: 05704335). All consecutive patients with a PCR-confirmed Influenza infection who reported headache during the course of the disease were screened for eligibility. A structured questionnaire was administered by a physician, who followed-up patients until the complete restitution. Headache phenotype was assessed by using the International Classification of Headache Disorders, 3rd version (ICHD-3) criteria.

**Results:** Seventy-five patients were enrolled, aged 43 years, 56% men, 27% with prior headache history. All patients fulfilled ICHD-3 criteria for *9.2.2 headache attributed to systemic viral infection*: all patients had a microbiologically confirmed Influenza infection, and in all cases, headache was developed in temporal relation to the onset of the infection and improved or resolved in parallel with the infection. Concerning the criterion C.4: 14.7% patients reported diffuse pain and 97.3% patients had a moderate-to-severe headache.

In the case of the phenotypic criteria for migraine or tension-type headache, these were fulfilled by 42.7% and 30.7% of patients, respectively (table 1).

**Conclusion:** A substantial proportion of patients with headache attributed to Influenza infection fulfilled ICHD-3 phenotypic criteria of migraine or tension-type headache, suggesting a common pathophysiological pathway in the headache genesis.

**Table 1 (Abstract P093):**
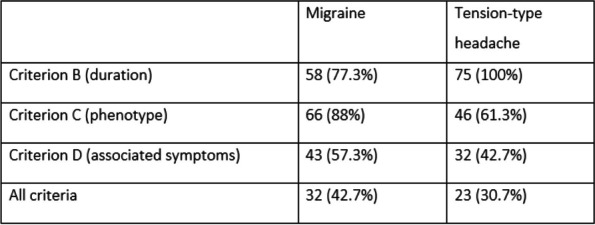
Proportion of patients who fulfilled the phenotypic criteria of migraine or tension-type headache

## P094 Vagal Cephalgia: a case report of a rare and overlooked phenomenon

### C. Zachariadi, E. Parasxou, E. Galliaki, A. Toumasis, I. Pilateris, A. Apostolakis, L. Besmerti, E. Makri, M. Terzoudi, G. Aposporos, A. Agathonikou

#### KAT General Hospital of Attica, Athens, Greece

##### **Correspondence:** C. Zachariadi

*The Journal of Headache and Pain 2024,* **25(Suppl 1)**: P094

**Objective:** The objective of this study is to present a case of a patient with vagal cephalgia as a rare manifestation of prominent infiltration of the vagal nerve in the setting of lung cancer, and discuss the clinical presentation, differential diagnosis and workup.

**Methods:** A 32-year-old male patient, smoker with no medical history, presented to our hospital complaining of right-sided facial pain throughout the past year. The pain was localized predominantly over the jaw, ear, and temporal area, and it was described as constant, severe, and dull in nature, exhibiting no autonomic features. Over this period the patient received a variety of medical regimes including carbamazepine, pregabaline, tramadol and NSAIDs in the context of possible, atypical trigeminal neuralgia, demonstrating minimum responsiveness.

**Results:** At the time of the presentation, the clinical neurological examination revealed no pathological signs or cranial nerve deficits. A brain MRI, including MRA and postgadolinium sequences, was performed, showing an asymmetrical meningeal thickness with gadolinium enhancement at the right temporal lobe (*Image 1*). Extensive laboratory tests demonstrated elevated CEA levels. A further investigation for primary malignancy was conducted, involving chest and abdominal CT scans, which revealed a large, enhancing, soft tissue tumor of the right hemithorax *(Image 2*). The patient was referred to a multimodal health center for further investigation and treatment. The neurological clinical diagnosis was vagal cephalgia, attributed to direct vagal infiltration at its thoracic route.

**Conclusion:** Vagal cephalgia is a rare headache disorder, attributed to lung neoplasm. Our case highlights the importance of a thorough diagnostic approach in patients presenting with refractory facial pain, especially when conventional drug therapies fail to provide relief. Early recognition of the syndrome and prompt diagnosis are crucial to optimize the outcome and prevent morbidity in these patients.

*Disclosure statement:* Informed consent to publish this case study and its potentially identifiable information of the patient was obtained from the individual involved. The patient gave explicit permission for the publication of this case report, including any relevant clinical details.

**Fig. 1 (Abstract P094):**
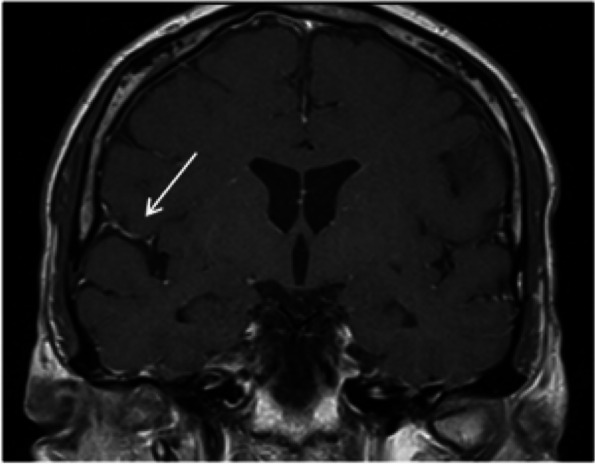
Brain MRI-T1 weighted post-gadolinium sequence showing an asymmetrical meningeal thickness with gadolinium enhancement at the right temporal lobe

**Fig. 2 (Abstract P094):**
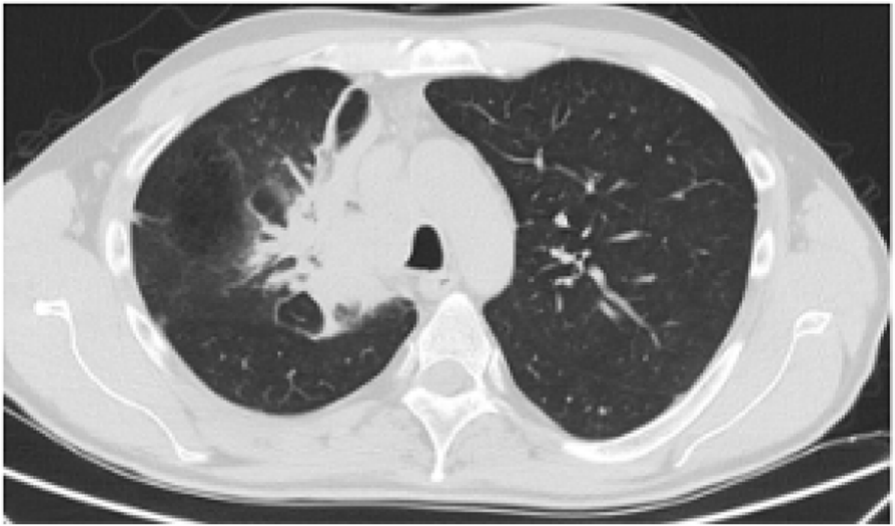
Contrast-enhanced thorax CT-scan revealing a large, enhancing, softtissue tumor of the right hemithorax

## P095 Efficacy, tolerability, and safety of onabotulinumtoxinA treatment for chronic migraine in patients with acute medication overuse: analysis of the PREEMPT and COMPEL trials

### R. Agosti^1^, A. Straube^2^, H. C. Diener^3^, M. Freeman^4^, J. Pascual Gómez^5^, M. Seminerio^6^, L. Delahaye^7^, R. J. Stark^8^

#### ^1^Kopfwehzentrum Hirslanden, Zurich, Switzerland; ^2^Ludwig Maximilian University of Munich, Munich, Germany; ^3^University of Duisburg-Essen, Essen, Germany; ^4^Headache Wellness Center, Greensboro, NC, United States; ^5^University Hospital Marqués de Valdecilla, Santander, Spain; ^6^AbbVie, North Chicago, IL, United States; ^7^AbbVie, Rungis, France; ^8^Alfred Hospital and Monash University Melbourne, Melbourne, Australia

##### **Correspondence:** R. Agosti

*The Journal of Headache and Pain 2024,* **25(Suppl 1)**: P095

**Objective:** To evaluate the efficacy, tolerability, and safety of onabotulinumtoxinA (onabotA) [BOTOX®] in patients treated for chronic migraine (CM) with or without acute medication overuse (MO).

**Methods:** Data analyzed from patients with CM treated with onabotA with or without MO across COMPEL, a phase 4, single-arm trial (NCT01516892) and PREEMPT, a phase 3, placebo (PBO)-controlled trial (NCT00156910, NCT00168428). Per ICHD, MO was defined as taking acute medication ≥2 times per week in any week (depending on the medication category) during screening. Patients received onabotA every 12wks for 108wks (COMPEL) or 56wks (PREEMPT). In PREEMPT, PBO patients received onabotA starting at week 24. Efficacy was reported as mean headache days (MHD), 6-item Headache Impact Test (6-HIT) score, and MSQ (Role Function Restrictive) score. Tolerability and safety were reported as adverse events (AEs).

**Results:** MO criteria was met by 65% (*n*=904/1384) of patients in PREEMPT (onabotulinumtoxinA: *n*=445, PBO: 459) and 64% (*n*=456/715) of patients in COMPEL (MO: *n*=456, no MO: *n*=259). In PREEMPT, onabotA reduced MHD vs PBO at 24wks in patients with MO (mean: -8.2 vs. -6.2, *P*<0.001) and without (-8.8 vs. –7.3, *P*=0.019). OnabotA reduced moderate/severe MHD with MO (*P*<0.001) and without (*P*=0.008). Severe impact via HIT-6 was reported by fewer patients with MO vs PBO at 24wks (*P*<0.001) and without MO (*P*=0.027). MSQ score was improved vs PBO at 24wks (*P*<0.001) and without (*P*<0.001). In COMPEL, the improvements were no different between patients with or without MO: MHD (mean: -10.6 vs -11.0, *P*=0.397), moderate/severe MHD (*P*=0.573) and HIT-6 score (*P*=0.644) at 108wks. Treatment related AEs were of similar frequencies in patients with MO (27%) or without (26%) and were consistent with onabotA safety profile for CM.

**Conclusion:** In this post-hoc-analysis, patients with CM and MO treated with onabotA responded in similar frequency to patients with CM without MO and displayed a similar safety profile.

## P096 Atogepant provides early improvements in daily functioning and quality of life: results from the PROGRESS chronic migraine trial

### J. Ailani^1^, P. Gandhi^2^, D. W. Dodick^3^, U. Reuter^4^, T. J. Schwedt^3^, Y. Liu^5^, B. Dabruzzo^2^, J. Stokes^2^, R. B. Lipton^6^

#### ^1^MedStar Georgetown University Hospital, Washington, DC, United States; ^2^AbbVie, Madison, NJ, United States; ^3^Mayo Clinic, Phoenix, AZ, United States; ^4^Charité – Universitätsmedizin Berlin, Berlin, Germany; ^5^AbbVie, North Chicago, IL, United States; ^6^Albert Einstein College of Medicine, Bronx, MA, United States

##### **Correspondence:** P. Gandhi

*The Journal of Headache and Pain 2024,* **25(Suppl 1)**: P096

**Objective:** Data from PROGRESS trial analyzed for impact of early onset of effect of atogepant (ato) on functioning and quality of life (QOL) in chronic migraine (CM).

**Methods:** PROGRESS was a phase 3 trial of participants with a ≥1-year history of CM, ≥15 headache days (HA days)/month in past 3 months, and ≥15 HA days over 28 days. Participants randomized to ato 30mg twice daily (BID), ato 60mg once daily (QD), or placebo for 12 weeks. Outcome measures: Performance of Daily Activities (PDA) and Physical Impairment (PI) domains of Activity Impairment in Migraine–Diary (AIM-D); 5-level European Quality of Life–5 Dimension (EQ-5D-5L) system and visual analogue scale (VAS). Changes in PDA and PI scores and proportion of participants achieving clinically meaningful within-patient change in PDA (≥12.5 points) and PI domain (≥10 points) were assessed in ato vs placebo.

**Results:** 755 participants in modified intent-to-treat population (placebo, *n*=246; ato 30mg BID, *n*=253; ato 60mg QD, *n*=256). Mean (SD) baseline for PDA (ato 60mg QD: 30.5 [18.0]; placebo 28.9 [16.7]) and PI (ato 60mg QD 27.0 [17.9]; placebo 25.1 [15.7]). Ato 60mg QD showed improvement from baseline at week 1 in PDA (least squares mean difference [LSMD]:−5.83; nominal *P*<0.0001) and reduced PI (LSMD: −4.12; nominal *P*=0.0004) scores vs placebo (Figure 1). Improved PDA and reduced PI domain scores for ato 60mg vs placebo seen at weeks 2-4. Week 1: those achieving reduction with PDA of ≥12.5 were 29.4% for placebo and 42.9% for ato 60mg QD (nominal *P*=0.008) and PI of ≥10 were 30.4% for placebo and 43.7% for ato 60mg QD (nominal *P*=0.003). Mean (SD) baseline values for EQ-5D-5L (ato 60 mg QD 0.76 [0.13]; placebo 0.77 [0.11]) and VAS scores (ato 60mg QD 65.0 [16.4]; placebo 64.4 [15.4]) similar between groups. Improvements in mean EQ-5D-5L (LSMD: 0.04; nominal *P*=0.0003) and VAS (LSMD: 4.47; nominal *P*=0.0018) scores were seen for ato 60mg vs placebo in the first 2 weeks (Figure 2).

**Conclusion:** Results show ato can improve function and QOL in those with CM.

**Fig. 1 (Abstract P096):**
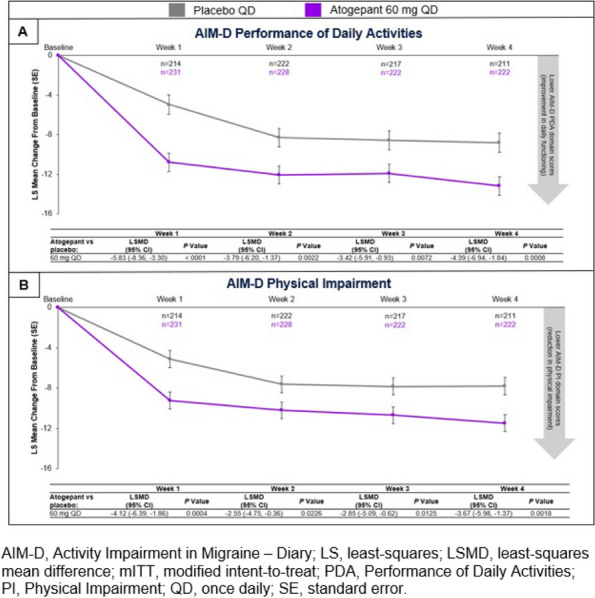
Change From Baseline in AIM-D (A) Perfoemance Of Daily Activities and (B) Physical Impairment at Weeks 1,2,3, and 4

**Fig. 2 (Abstract P096):**
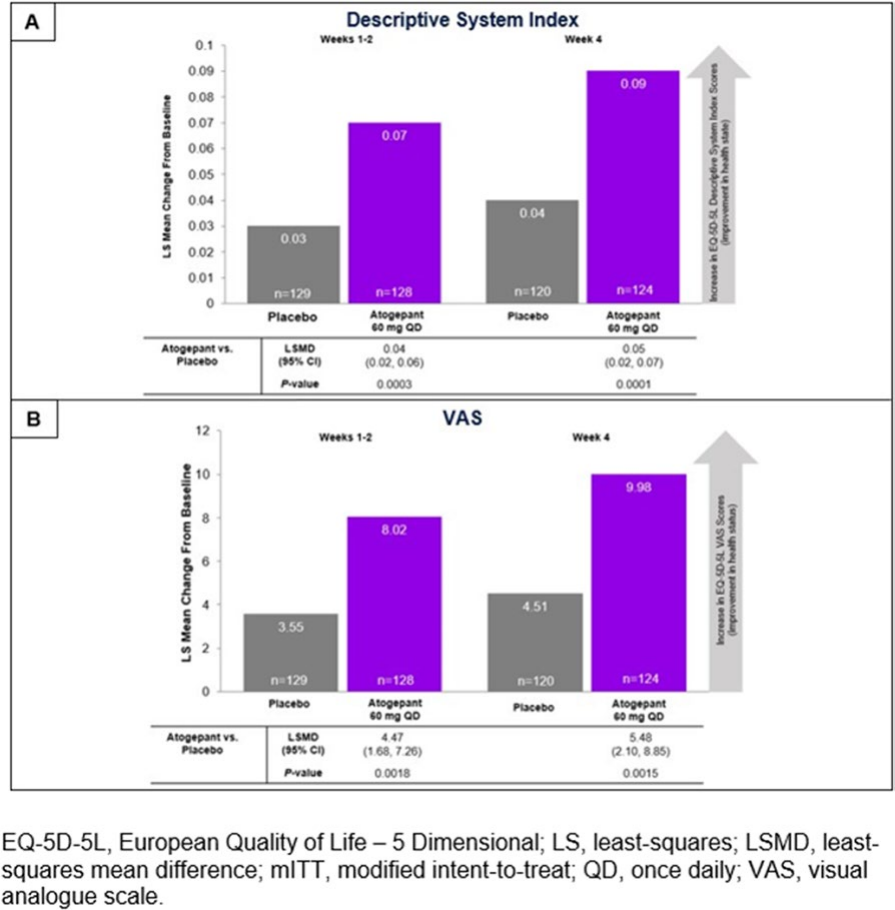
Change From Baseline in EQ-5D-5L (A) Descriptive System Index and (B) Visual Analogue Scale Scores at Weeks 1-2 and Week 4

## P097 New-onset severe headache after Covid-19 vaccine in Norway (CovaxHEAD)

### M. B. Argren^1,2^, H. Lier^1,2^, M. Boldingh^1^, K. Devik^3^, K. Gjendemsjø^4^, A. Husøy^5,2,6^, S. M. Mathisen^7^, Å. H. Morsund^8^, A. C. Poole^9^, M. E. Revheim^10^, K. Schlüter^7^, J. Sønnervik^11^, T. Skattør^12^, E. Tronvik^5,2,6^, M. Aalstad-Johansen^13^, M. Toft^1^, B. S. Winsvold^1,2^, A. H. Aamodt^1,2^

#### ^1^Oslo University Hospital, Department of Neurology, Oslo, Norway; ^2^Norwegian centre for headache research, NorHEAD, Trondheim, Norway; ^3^Namsos Hospital, Department of Neurology, Namsos, Norway; ^4^Sandvika Neuro centre, Sandvika, Norway; ^5^Norwegian University of Science and Technology, Department of Neuromedicine and Movement Science (INB), Trondheim, Norway; ^6^St. Olavs University Hospital, Department of Neurology and Clinical Neurophysiology, Trondheim, Norway; ^7^Stavanger University Hospital, Department of Neurology, Stavanger, Norway; ^8^Molde Hospital, Department of Neurology, Molde, Norway; ^9^Oslo Headache Centre, Oslo, Norway; ^10^Institute of Clinical Medicine, University of Oslo, Oslo, Norway; ^11^Hodeverket, Sandnes, Norway; ^12^Oslo University Hospital, Department of Radiology, Oslo, Norway; ^13^Innlandet Hospital Trust, Department of Neurology, Lillehammer, Norway

##### **Correspondence:** M. B. Argren

*The Journal of Headache and Pain 2024,* **25(Suppl 1)**: P097

**Objective:** Headache is the most common neurological symptom after Covid-19 vaccination, with severe cases occurring in about 1.0%. The detailed clinical phenotype of vaccination-associated headaches remains unknown. This study aims to examine the clinical characteristics and treatment effects of severe new-onset headaches after Covid-19 vaccination.

**Methods:** A national Norwegian prospective cohort study (CovaxHEAD) of consecutive patients with new-onset severe headaches after Covid-19 vaccination. Participants underwent assessments by neurologists or headache specialists, and blood samples were collected. MRI and patient-reported outcome measures were also employed. Inclusion criteria included patients with new-onset severe headache or severe worsening of pre-existing headache within 7 days after Covid-19 vaccination.

**Results:** In total 63 patient were included, with a strong female preponderance (47 (75%) women). Mean follow up time was 6.4 months. Migraine was the most common phenotype (66.7%), of which 48% had chronic migraine. Chronic tension type headache was the second most common (19%). Mean baseline HIT-6 was 65 (range 58-78); with a mean reduction to 61 (range 36-70). Baseline MRI from 53 (84%) patients showed no signs of inflammation, stroke or vascular pathology. Lumbar puncture was performed in 33% of the cases, with normal findings. During the follow up period, 77% had a breakthrough COVID-19 infection. Biomarker analyses will follow.

**Conclusion:** The most common phenotype of post-Covid-19 vaccine headache was chronic migraine with minor improvement during follow-up despite prophylactic anti-migraine treatment. Future biomarker analyses are needed to clarify mechanisms behind persistent headaches after Covid-19 vaccinations. Collaborative research efforts are essential to contribute to the development of diagnostic criteria and effective treatment strategies, and to gain experience with this newly emerged headache, understanding its implications in the context of the Covid-19 vaccination era.

## P098 Prevalence and characteristics of patients with migraine unsuitable for triptan treatment: a systematic literature review

### R. B. Lipton^1,2^, A. Gendolla^3^, A. Jenkins^4^, L. Abraham^4^, J. Telfort^4^, K. Hygge Blakeman^4^, P. Saccone^4^, I. Fotheringham^5^, I. Pustulka^5^, A. Engh^5^

#### ^1^Albert Einstein College of Medicine, New York, NY, United States; ^2^Montefiore Medical Center, New York, NY, United States; ^3^Private Practice, Essen, Germany; ^4^Pfizer R&D UK Ltd., Surrey, United Kingdom; ^5^Evidera Inc., London, United Kingdom

##### **Correspondence:** L. Abraham

*The Journal of Headache and Pain 2024,* **25(Suppl 1)**: P098

**Objective:** Triptans, although widely used in the acute treatment of migraine, are associated with clinical challenges in some patients. There is debate about the relative frequency and characteristics of this population. This search was conducted to identify and evaluate published evidence on the relative frequency and characteristics of patients with migraine who are unsuitable for triptan treatment.

**Methods:** This review considered Medline- and Embase-indexed articles and conference abstracts (2011 to Aug 2022) describing migraine unsuitable for triptans for any reason. Data describing the relative frequency and characteristics of this group were extracted. Evidence on the burden of disease in this population was also collected and is presented separately.

**Results:** Among 1460 records screened, 20 reported on the relative frequency of unsuitability for triptans (Figure 1), described as patients with contraindications or those who tried ≥1 triptan and discontinued or experienced triptan failure. It was reported that 2.4%–15% of patients with migraine had clear triptan contraindications, and that up to 20% of patients receiving triptans had contraindications. Additionally, 51%–66% of patients starting a new triptan did not refill it (Figure 2), and 43%–100% discontinued by 2 years of follow-up; <20% tried a subsequent triptan, and most received no subsequent acute migraine prescriptions (29%–91%) or switched to another class (≤59%). At 2 years of follow-up, <21% of patients persisted on the index triptan. Based on 4 survey studies, 10%–44% of patients who try triptans have insufficient response, although definitions varied.

**Conclusion:** The total population of patients unsuitable for triptans is uncertain, but the literature highlights a large group who cannot or do not persist with triptans despite ongoing migraines. Many are prescribed triptans despite having clear contraindications. Further research is needed to determine the relative frequency of unsuitability for triptan use.

**Fig. 1 (Abstract P098):**
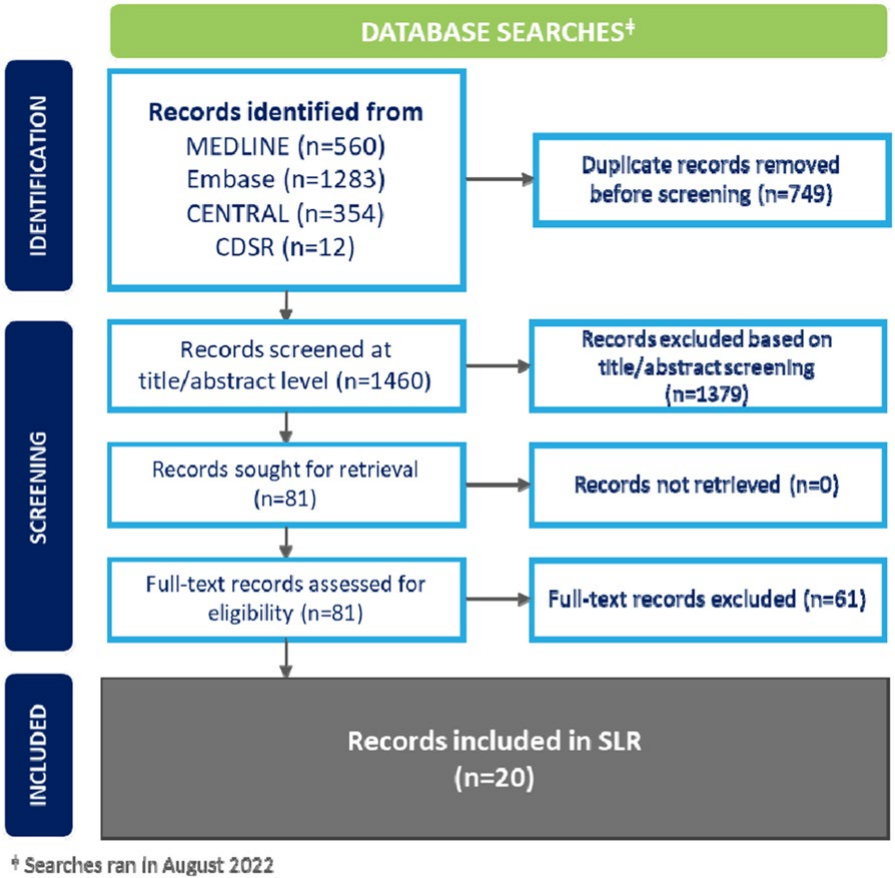
See text for description

**Fig. 2 (Abstract P098):**
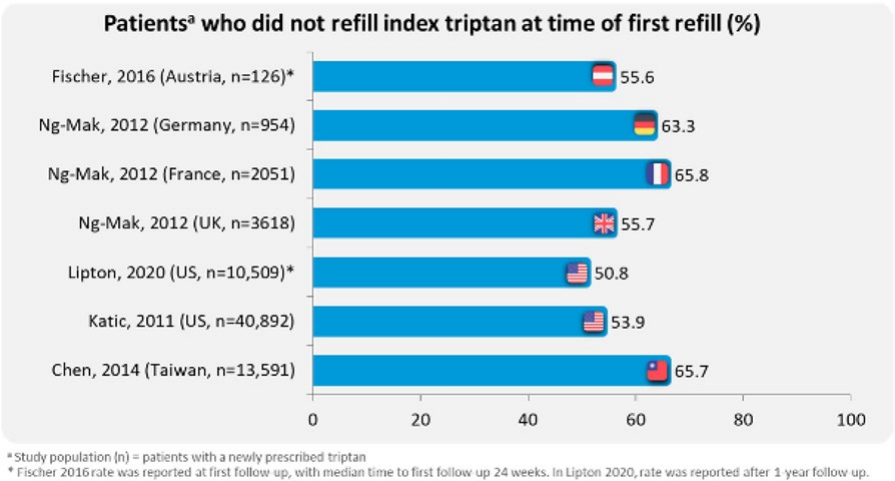
See text for description

## P099 Analysis of the demographic, treatment, and surgical profile of idiopathic intracranial hypertension with and without papilledema from a multicenter electronic medical record database

### V. Wang^1^, M. Pickles^2^, M. Peres^1,3^, H. Yuan^1^

#### ^1^Jefferson Headache Center, Neurology, Philadelphia, PA, United States; ^2^Sidney Kimmel Medical College, Thomas Jefferson University, Philadelphia, PA, United States; ^3^University of São Paulo, Institute of Psychiatry, São Paulo, Brazil

##### **Correspondence:** V. Wang

*The Journal of Headache and Pain 2024,* **25(Suppl 1)**: P099

**Objective:** To investigate the demographic, treatment, and surgical profile of idiopathic intracranial hypertension (IIH) with papilledema (IIHwP) vs without papilledema (IIHWOP).

**Methods:** Using the TriNetX database, a cohort of patients with IIH with any history of a diuretic prescription (acetazolamide, methazolamide, furosemide, topiramate or zonisamide) (IIH-D) was created to validate the diagnosis of IIH. From the IIH-D cohort, cohorts of IIHwP and IIHWOP were created. Information regarding patient demographics, medical diagnoses, surgical and procedural history, and medications was obtained.

**Results:** From a total network population of 131,850,716 patients, we identified 105,430 patients with a diagnosis of IIH (prevalence 0.1%), of which 57,246 (54.3%) were prescribed a diuretic. These patients had a mean age of 36.5±16.6, female-to-male ratio (F/M) of 4.5, 57.7% were white, 20.7% black, and 8.0% Hispanic/Latino. Medical comorbidities, procedures, and treatments included: migraine (22.3%), obesity/overweight (28.8%), visual disturbances/blindness (24.9%), visual field examination (VFE, 14.6%), ventriculoperitoneal shunt (VPS, 4.0%), and venous sinus stenting (VSS, 0.04%).

From the IIH-D population, the IIHwP vs IIHWOP patients comprised of profiles as follows: total count of 20,134 (19.1%) vs 37,112 (35.2%), mean age of 31±13.2 vs 39.6±17.5, F/M of 6.3 vs 3.8, 59.6% vs 57.3% white, 21.2% vs 20.7% black (*p*=0.14), migraine (22.8% vs 23.4%, *p*=0.13), obesity (31.9% vs 29.5%), visual disturbances/blindness (40.9% vs 18.1%), VFE (31.6% vs 7.2%), VPS (2.2% vs 5.0%), and VSS (0.2% vs 0.1%, *p*=0.01), respectively. All comparisons were statistically significant *p*<0.001 unless otherwise specified.

**Conclusion:** Based on the TriNetX database, IIHwP patients are younger, with higher rates of obesity, blindness, visual field exams, F/M ratio, and lower rates of VPS compared to IIHWOP patients.

## P100 Analysis of the epidemiologic profile and healthcare utilization impact of functional neurologic disorder in patients with migraine from a multicenter electronic medical record database

### V. Wang^1^, E. Le^2^, M. Peres^1,3^, H. Yuan^1^

#### ^1^Jefferson Headache Center, Neurology, Philadelphia, PA, United States; ^2^Sidney Kimmel Medical College, Thomas Jefferson University, Philadelphia, PA, United States; ^3^University of São Paulo, Institute of Psychiatry, São Paulo, Brazil

##### **Correspondence:** V. Wang

*The Journal of Headache and Pain 2024,* **25(Suppl 1)**: P100

**Objective:** To investigate the demographic, treatment, and healthcare utilization profile of migraine patients with and without functional neurologic disorders (FND).

**Methods:** Using the TriNetX database, cohorts of migraine patients with FND with motor weakness [HY1] [VW2] (MwF, ICD10 G43 with F44.4) and migraine patients without FND with motor weakness (MwoF) were evaluated. Information was obtained regarding patient demographics, medical comorbidities, medication use, therapy services, and outcomes [emergency department usage (EDU) and inpatient hospitalizations (IH)]. Propensity score-matched cohorts of MwF and MwoF with standardized characteristics (demographics, medical and psychiatric comorbidities, and antimigraine agents) were created to compare EDU and IH.

**Results:** From a network population of 131,850,716 patients, we identified a total of 2,666,296 (prevalence 2.0%) with a diagnosis of migraine, of which 14,108 (0.5%, prevalence 0.01%[HY1] [VW2] ) were MwF while 2,652,188 (99.5%, prevalence 2.0%) were MwoF. For MwF compared to MwoF, the mean age was 39.8±16.4 vs 38.9±17.9, female-to-male ratio was 5.4 vs 3.5, 68.8% vs 67.1% were white, and 16.2% vs 11.7% were black. Medical and psychiatric comorbidities and treatment profiles of MwF vs MwoF include transient ischemic attack (11.4% vs 3.2%), cerebral infarction (9.3% vs 1.2%), mood disorders (52.7% vs 14.4%), anxiety (71.7% vs 16.5%), and depression (16.37% vs 2.92%), sumatriptan (18.1% vs 5.7%), physical therapy (12.0% vs 2.6%), and psychotherapy (10.9% vs 1.7%). All comparisons were statistically significant *p*<0.0001. Post-propensity score-matching of MwF compared to MwoF revealed both elevated EDU: Risk 47.9% vs 41.0%, risk ratio [HY3] [EL4] (RR) 1.170, 95%CI (1.139-1.202) and elevated IH: Risk 48.6% vs 35.1%, RR 1.385, 95%CI (1.345-1.425).

**Conclusion:** MwF patients have a significantly higher proportion of black race, cerebrovascular diagnoses, psychiatric comorbidities, medication, ambulatory therapies, and use of hospital emergency and inpatient resources.

## P101 Intractable late onset headaches after pituitary macroadenoma surgery, a report of 5 cases

### M. Togha^1^, A. Nasermoghadasi^2^, S. Haghighi^1^, S. Razeghi Jahromi^3^

#### ^1^Tehran University of Medical Sciences, Headache Department, Iranian Center of Neurological Research, Neuroscience Institute, Tehran, Iran; ^2^Tehran University of Medical Sciences, Multiple Sclerosis Research Center, Neuroscience Institute, Tehran, Iran; ^3^Shahid Beheshti University of Medical Sciences, Department of Clinical Nutrition and Dietetics, Tehran, Iran

##### **Correspondence:** S. Razeghi Jahromi

*The Journal of Headache and Pain 2024,* **25(Suppl 1)**: P101

**Objective:** The Sella turcica is in the vicinity of several critical brain structures. Involvement in any of these structures can be accompanied by intense headaches. pituitary tumors, involvement of sphenoid sinus by Aspergillus sphenoid bone metastasis, and rarely Langerhans cell histiocytosis can cause severe headaches due to the irritation of pain receptors in this region. Treating diseases in the pericellular areas usually leads to headache treatment and the improvement of other signs and symptoms. Headache is a known complication of pituitary tumors. Different treatment methods such as surgery and radiosurgery are used to treat these tumors. There are some reports of patients in whom preoperative headaches still exist after surgery or de novo postoperative headaches started, but reports of intractable TAC-type headaches are scarce.

**Methods:** Here we report 5 cases of intractable headaches after transsphenoidal surgery of pituitary lesions. These patients had been referred to the Headache Clinic of Sina Hospital affiliated with the Tehran University of Medical Sciences, Tehran, Iran.

**Results:** In these patients, headaches were strictly unilateral, mostly in the periorbital area, and were accompanied by ipsilateral autonomic features. In our cases, headaches remained unabated following surgery or Gamma Knife radiosurgery, and in 4 cases, headaches developed or intensified following the procedure. As these posts, transsphenoidal surgery headaches and associated symptoms were so similar, and no CSF leak, abscess, pneumocephalus, cerebral infection or hemorrhage, meningitis, or hydrocephalus was found after surgery, we considered them as a type of secondary trigeminal autonomic cephalalgia and an important issue, which to be looked for. If the type of surgery may play a role in the development or continuation of headaches is a question that should be answered.

**Conclusion:** This case series could track the attention toward the possibility of severe headaches with cranial autonomic features after macroadenoma surgical treatment.

## P102 What is impact of biological treatment on quality of life of patients with migraine: Real-world data from patient questionnaires in Migraine Registry (ReMig) in the Czech Republic

### T. Nežádal^1^, T. Doležal^2^, D. Pejřilová^2^, B. Turková^2^, J. Marková^3^, A. Bártková^4^, L. Klečka^5^, C. Z. ReMig study group^2^

#### ^1^Military University Hospital, Prague, Czech Republic; ^2^Value Outcomes, Prague, Czech Republic; ^3^University Thomayer Hospital, Department of Neurology, Prague, Czech Republic; ^4^Palacký University Hospital, Department of Neurology, Olomouc, Czech Republic; ^5^Municipal Hospital, Department of Neurology, Ostrava, Czech Republic

##### **Correspondence:** T. Nežádal

*The Journal of Headache and Pain 2024,* **25(Suppl 1)**: P102

**Objective:** Evaluation of work productivity, quality of life, impact of migraine on life and depression from patient questionnaires in the Czech Registry of patients with Migraine on biological treatment (ReMig).

**Methods:** In addition to clinical data, patient questionnaires, specifically the WPAI, EQ-5D, HIT-6 and CUDOS, were prospectively collected within the ReMig registry. Questionnaires were completed electronically by patients at the time of the follow-up visit to the physician every 3 months.

**Results:** During the initial visit, a total of 727 questionnaires were collected and analysed, and 452 questionnaires were collected after 12 months of CGRP monoclonal antibody therapy.

For the employed patients, the mean absenteeism due to migraine was 11.6% and 2.7%, mean presenteeism was 49.6% and 15.6%, and mean overall work impairment was 52.7% and 16.5%, before and after 12 months of treatment, respectively. The mean daily activity impairment evaluated in all patients was 58.2% at treatment initiation and 19.3% after 12 months of treatment.

The mean utility assessed by the EQ-5D questionnaire reflecting patient quality of life was 0.75 and 0.88 before and after 12 months of treatment, respectively. Patient-reported current health status on the visual analogue scale was 64.6 and 78.2 before and after 12 months of biological treatment, respectively.

At the start of biological treatment, 94.6% of patients experienced significant or huge impact of migraine on their lives, after 12 months of treatment, this proportion was 48.0%.

In total, only 23.5% of patients were free of depression when initiating biological treatment, after 12 months of treatment, more than half of the patients (58.8%) were free of depressive symptoms.

**Conclusion:** Patient questionnaires contribute to clinical data by subjectively assessing patients´ quality of life. Analysis shows improvements in ratings of work productivity, quality of life, impact of migraine on life, and depression due to migraine.

## P103 Disease trajectories show increased risk of stroke in trigeminal neuralgia

### J. Worm^1^, I. F. Jørgensen^2^, Ó. B. Davídsson^1,3^, H. W. Schytz^1^, L. Bendtsen^1^, S. Brunak^2^, T. F. Hansen^1,2^, S. Maarbjerg^1^

#### ^1^Copenhagen University Hospital – Rigshospitalet, Department of Neurology, Danish Headache Center, Copenhagen, Denmark; ^2^University of Copenhagen, Novo Nordisk Foundation Center for Protein Research, Copenhagen, Denmark; ^3^Statens Serum Institut, Department of Epidemiology Research, Copenhagen, Denmark

##### **Correspondence:** J. Worm

*The Journal of Headache and Pain 2024,* **25(Suppl 1)**: P103

**Objective:** To identify temporal associated comorbidities in trigeminal neuralgia using population-based disease trajectories.

**Methods:** A total of 7.2 million unique individuals from the Danish National Patient Register (DNPR) were included from 1994 to 2018 to cover the trigeminal neuralgia population. To identify diseases more prevalent in trigeminal neuralgia, we compared participants with trigeminal neuralgia to 10,000 age- and sex-matched controls randomly selected from the DNPR. Binomial Bonferroni corrected tests were used to determine whether the diseases were diagnosed before or after the trigeminal neuralgia diagnosis. To investigate the temporal relationship further, we created a disease trajectory network which allowed us to track disease progression over time and identify significant patterns in trigeminal neuralgia. Finally, a supplementary Cox-regression analysis was conducted combining the DNPR with the Danish National Prescription Register to investigate if stroke risk was associated with carbamazepine or oxcarbazepine treatment.

**Results:** We included 7141 individuals with trigeminal neuralgia in the study. The mean age at diagnosis was 59 years and 64% were women. Among these participants, we detected 27 significantly linked comorbidities (Figure 1) with 18 specific diseases preceding a diagnosis of trigeminal neuralgia. Following a trigeminal neuralgia diagnosis, the participants had an increased risk of developing nine diseases, including ischemic stroke with a relative risk of 1.55. Furthermore, individuals with trigeminal neuralgia treated with carbamazepine or oxcarbazepine had a higher risk of stroke compared to those treated with other antiepileptic medications, with a hazard ratio of 1.78 (95% CI: 1.47-2.17).

**Conclusion:** In a Danish trigeminal neuralgia population, we discovered specific comorbidities with a significant temporal association to the trigeminal neuralgia diagnosis. Individuals with trigeminal neuralgia have a higher risk of stroke possibly augmented by using first-line medical treatment. Our results indicate a need for considering vascular risk factors in individuals with trigeminal neuralgia.

**Fig. 1 (Abstract P103):**
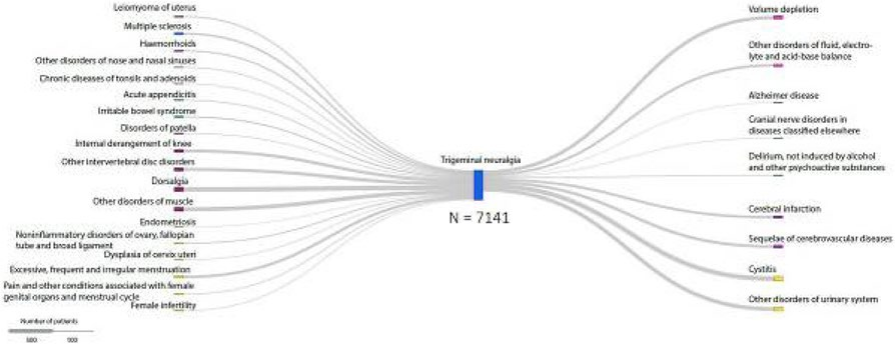
See text for description

## P104 Impact and burden of migraine and medication-overuse headache from the perspective of people with migraine: Insights from the mixed-method Migraine Community Survey

### E. Ruiz de la Torre^1^, A. Feoktistov^2^, M. Fernando Prieto Peres^3^, W. Gerhart^4^, I. Cerdá Marcos^5^, L. Pickering Boserup^5^, C. Lamb^6^, D. C. Buse^7^

#### ^1^European Migraine and Headache Alliance, Brussels, Belgium; ^2^Synergy Integrative Headache Center, Chicago, IL, United States; ^3^University of São Paulo, São Paulo, Brazil; ^4^Migraine Canada, Ontario, Canada; ^5^H. Lundbeck A/S, Copenhagen, Denmark; ^6^ApotheCom, Inizio Medical, London, United Kingdom; ^7^Albert Einstein College of Medicine, New York, NY, United States

##### **Correspondence:** E. Ruiz de la Torre

*The Journal of Headache and Pain 2024,* **25(Suppl 1)**: P104

**Objective:** To explore how headache frequency and headache pain severity relate to perceived burden of migraine. We also explore current perceptions of medication-overuse headache (MOH).

**Methods:** A cross-sectional, mixed-method, online survey was completed by people with a self-reported medical diagnosis of migraine in Europe and Canada on May 1–30, 2022. The survey was translated into 9 languages and distributed by patient advocacy groups. Headache frequency in the preceding 3 months was captured in categories (0–7, 8–14, 15–23 or ≥24 monthly headache days [MHDs]). Mean headache pain severity in the preceding 3 months and perceived current impact of migraine on aspects of daily life were ranked using 10-point scales (1=lowest; 10=highest). Overall impact results and results stratified by headache frequency (≥15 vs ≤14 MHDs) and headache pain severity (≥7 vs ≤6) are presented. Quantitative and qualitative data were analysed using descriptive statistics and framework analyses, respectively.

**Results:** Mean ranking of headache pain severity was 6.7 (*N*=1136), and 50.4% of respondents (n/*N*=572/1135) reported ≥15 MHDs in the preceding 3 months. Mean overall rankings of impact on family life, work/study, and social life were 7.1 (*N*=1139), 7.7 (*N*=1119) and 7.8 (*N*=1139), respectively (Fig. 1). Greater headache pain severity and headache frequency corresponded with greater life impact; mean differences in impact were greater when stratified by headache pain severity than headache frequency (family life, +1.2 vs +0.5; work/study, +1.0 vs +0.6; social life, +1.1 vs +0.5) (Fig. 1). In the framework analysis, MOH was most often described as headache triggered by frequent acute medication use (23.0%; n/*N*=217/927); 5.6% (n/*N*=52/927) described MOH as a secondary (new) headache type, and 2.2% (n/*N*=20/927) mentioned MOH as something to be cautious of when using acute medication.

**Conclusion:** In this sample of respondents with equal proportions of people with episodic and chronic migraine, migraine has a substantial impact on daily life, and headache pain severity has a larger perceived impact than headache frequency. Differing perceptions of MOH may represent unmet communication needs.

**Fig. 1 (Abstract P104):**
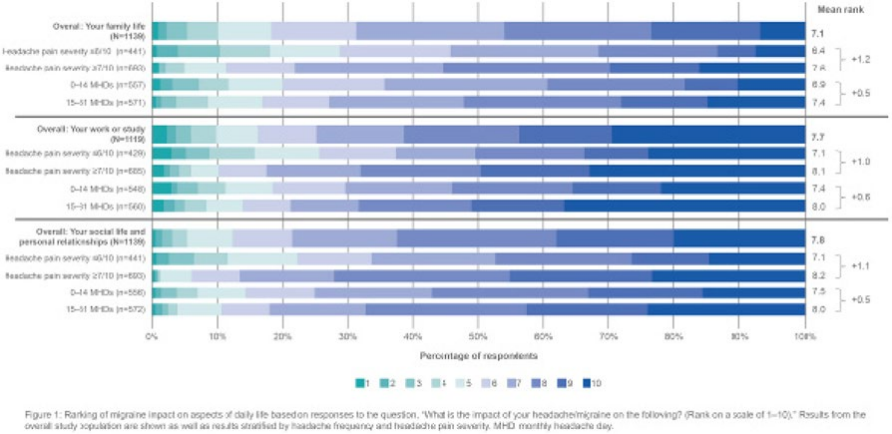
See text for description

## P105 Prevalence of headaches in dialysis patients, a cross-sectional study

### M. Togha^1^, Z. Salami^1^, D. Shahamati^2^, A. Javanmardi^1^, O. Kohandel Gargari^1^, N. EshaghHosseiny^1^

#### ^1^Tehran University of Medical Sciences, Headache Department, Iranian Center of Neurological Research, Neuroscience Institute, Tehran, Iran; ^2^Shahid Behehshti University of Medical Sciences Tehran, Faculty of Nutrition Sciences and Food Technology, National Nutrition and Food Technology Research Institute, Tehran, Iran

##### **Correspondence:** M. Togha

*The Journal of Headache and Pain 2024,* **25(Suppl 1)**: P105

**Objective:** Patients undergoing maintenance hemodialysis (MHD) for end-stage renal disease commonly experience headaches during and after dialysis sessions. Frequent headache attacks could further affect their quality of life. This study evaluated headaches in MHD patients.

**Methods:** This cross-sectional study was run through a questionnaire among ESRD patients undergoing MHD at three tertiary care medical hospitals. All patients had undergone MHD regularly for over six months. ICHD-3 classification was used to diagnose headaches in the patients. Cases with any CNS or any other organ failure were excluded, and a visual analog scale was implemented to estimate headache severity. Tests such as the T-test, Chi-square, and regression analysis were conducted to determine the association between HDH and various postulated factors.

**Results:** Forty-five cases of 111 hemodialysis patients in this study were suffering from hemodialysis headaches (HDH). The most common comorbidities observed were hypertension, diabetes, dyslipidemia, and a history of cerebrovascular attack. Sodium, potassium, calcium, phosphorus, Blood sugar, blood urea nitrogen, creatinine levels, and plasma osmolality were measured and compared before and after dialysis between those suffering from headaches and headache-free patients. There was no significant difference between the two groups. The mean Systolic blood pressure before and after dialysis among headache cases was higher than the patients who did not complain of headaches. Most patients had bilateral and not severe headaches and were usually aborted spontaneously. In about one-third of the patients, headache duration was less than 30 minutes; in one-third, it lasted one hour. Approximately one-third of patients required painkillers to alleviate the headache.

**Conclusion:** Results showed that almost 40% of these patients experience HDH. Patients with a pre-existing primary headache disorder were found to be at a higher risk of developing HDH. Frequent headaches during dialysis may negatively impact the quality of life and potentially lead to depression, emphasizing the need to find effective strategies to reduce HDH and improve the QOL of ESRD patients undergoing HDH.

## P106 Network meta-analysis of atogepant vs. CGRP mAbs on migraine-specific quality of life questionnaire v2.1 in episodic and chronic migraine

### J. Ailani^1^, L. Dupont-Benjamin^2^, C. Tassorelli^3^, S. Sacco^4^, S. J. Nahas^5^, A. Lalla^6^, I. Ubamadu^7^, G. Pietri^7^, P. Gandhi^8^, R. B. Halker Singh^9^

#### ^1^MedStar Georgetown University Hospital, Washington, DC, United States; ^2^AbbVie, Courbevoie, France; ^3^Headache Science & Neurorehabilitation Centre, C. Mondino Foundation and University of Pavia, Pavia, Italy; ^4^University of L'Aquila, L'Aquila, Italy; ^5^Thomas Jefferson University Hospital, Department of Neurology, Philadelphia, PA, United States; ^6^AbbVie, Irvine, CA, United States; ^7^AbbVie, London, United Kingdom; ^8^AbbVie, Madison, NJ, United States; ^9^Mayo Clinic, Scottsdale, AZ, United States

##### **Correspondence:** S. J. Nahas

*The Journal of Headache and Pain 2024,* **25(Suppl 1)**: P106

**Objective:** Conduct a network meta-analysis (NMA) of atogepant vs calcitonin gene–related peptide monoclonal antibodies (CGRP mAbs) on the Migraine-Specific Quality of Life questionnaire (MSQ) v2.1.

**Methods:** A clinical systematic literature review was conducted to identify data for branded preventive migraine treatments approved for episodic migraine (EM) and chronic migraine (CM) (database searches were performed May 11, 2020 and updated Sept 1, 2022). A Bayesian NMA was conducted using fixed or random effects models. The choice of model was based on best fit statistics. NMA was performed for the overall EM and CM populations. Evaluated treatments included atogepant 10mg (EM only), 30mg (EM only), and 60mg once daily; galcanezumab 120mg monthly; fremanezumab 225mg monthly and 675mg quarterly; and erenumab 70mg and 140mg monthly. The MSQ v2.1 Role Function–Restrictive (RFR), Role Function–Preventive (RFP), and Emotional Function (EF) domains from EM and CM trials were included. Results are presented as model estimates of score mean difference and corresponding 95% credible interval for atogepant vs the CGRP mAbs.

**Results:** Based on best fit statistics, a fixed effects model was used for the overall EM and CM populations. In the EM population, all atogepant doses demonstrated numerically greater improvements from baseline in MSQ RFR than all CGRP mAbs (Figure 1A). Atogepant 30mg and 60mg demonstrated numerically greater improvement in MSQ RFP than all CGRP mAbs (Figure 2A) and statistically significant greater improvement in MSQ RFR and EF domains compared with erenumab 70mg and fremanezumab 675mg (Figure 3A). In the CM population, changes in each MSQ domain (Figures 1B, 2B, 3B) were not statistically different between atogepant and CGRP mAbs.

**Conclusion:** Atogepant 30mg and 60mg demonstrated statistically significant greater improvements in MSQ RFR and EF domains compared with erenumab 70mg and fremanezumab 675mg in the EM population; other findings were comparable.

**Fig. 1 (Abstract P106):**
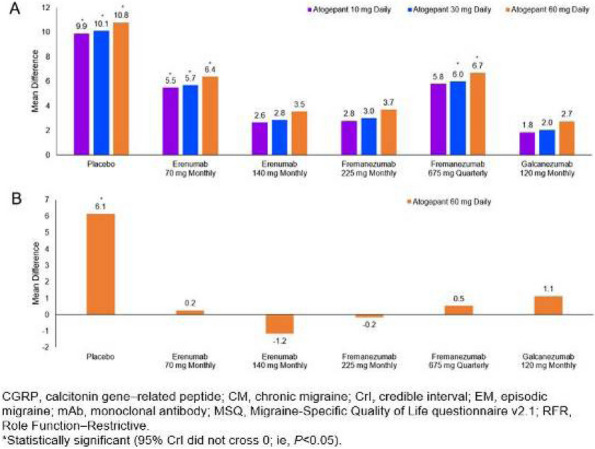
Changes From Baseline in MSQ RFR Domain at Week 12 Among the Overall (**A**) EM and (**B**) CM Populations: Network Meta-analysis of Atogepant vs CGRP mAbs

**Fig. 2 (Abstract P106):**
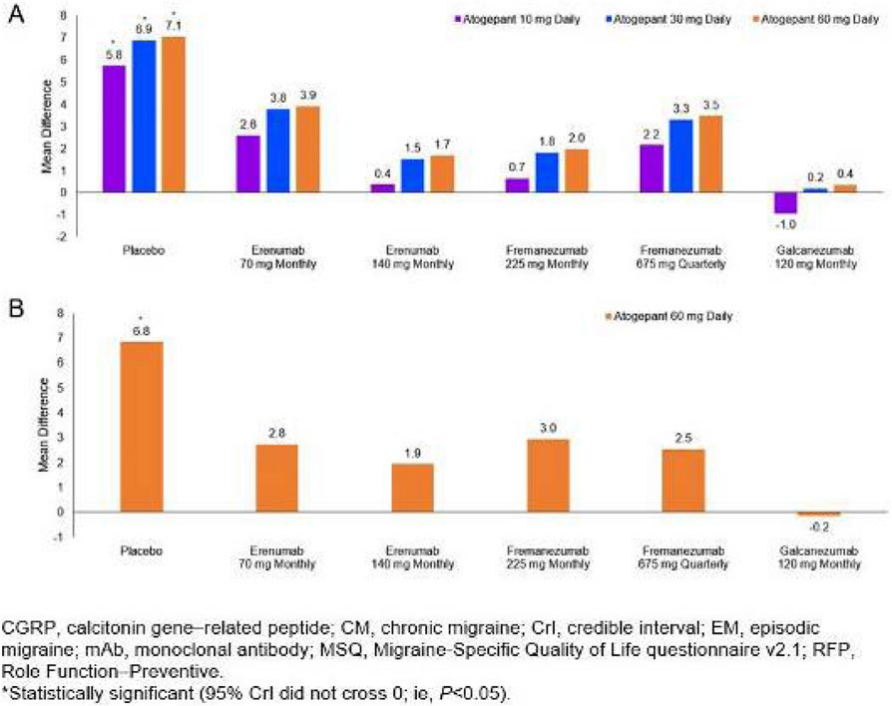
Changes From Baseline in MSQ RFP Domain at Week 12 Among the Overall (**A**) EM and (**B**) CM Populations: Network Meta-Analysis of Atogepant vs Cgrp mAbs

**Fig. 3 (Abstract P106):**
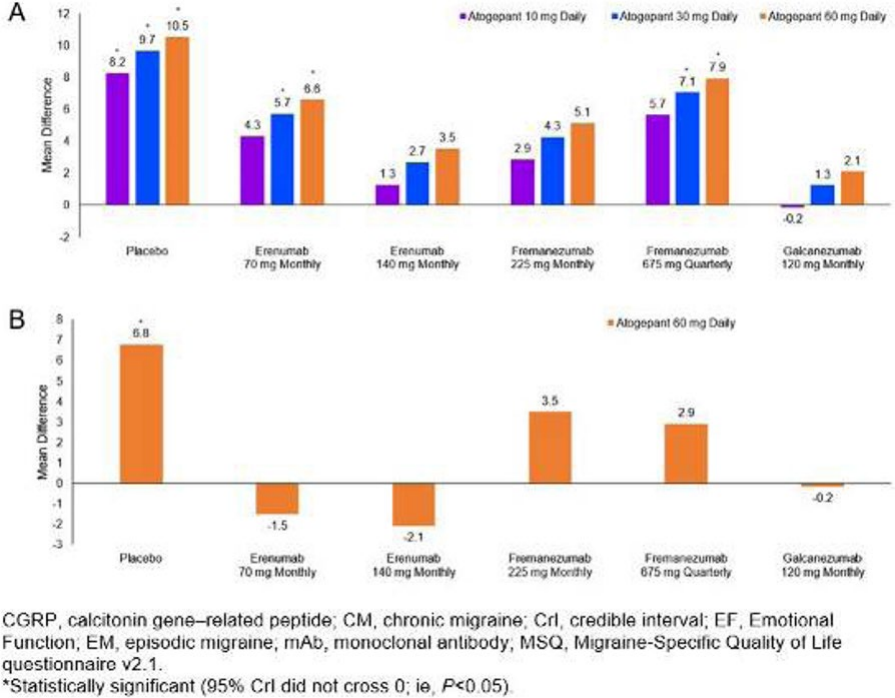
Changes From Baseline in MSQ EF Domain at Week 12 Among the Overall (**A**) EM and (**B**) CM Populations: Network Meta analysis of Atogepant vs. CGRP mAbs

## P107 An economic evaluation of eptinezumab for preventive treatment of migraine in the UK, with consideration for natural history and productivity at work

### E. Griffin^1^, G. Shirley^2^, X. Y. Lee^3^, S. Awad^3^, A. Tyagi^4^, P. J. Goadsby^5,6^

#### ^1^Edward Griffin Consulting, LTD., Abbot, London, United Kingdom; ^2^Lundbeck Limited, Watford, United Kingdom; ^3^H. Lundbeck A/S, Valby, Denmark; ^4^Queen Elizabeth University Hospital, Institute of Neurological Sciences, Glasgow, United Kingdom; ^5^NIHR-King's Clinical Research Facility, London, United Kingdom; ^6^University of California, Department of Neurology, Los Angeles, CA, United States

##### **Correspondence:** P. J. Goadsby

*The Journal of Headache and Pain 2024,* **25(Suppl 1)**: P107

**Objective:** Migraine is a highly prevalent neurological disorder with a substantial societal burden due to lost productivity. We assessed from a societal perspective the cost-effectiveness of eptinezumab for the preventive treatment of migraine.

**Methods:** An individual patient simulation of discrete competing events was developed to evaluate eptinezumab cost-effectiveness compared to best supportive care for adults in the UK with ≥4 migraine days per month and who have failed ≥3 preventive treatments: a population combining episodic and chronic migraine subgroups. Clinical efficacy, utility, and work productivity inputs were based on results from the DELIVER randomised controlled trial. Timing of natural history events and treatment holidays - informed by the literature - were simulated to unmask any natural improvement. The primary outcomes were monthly migraine days, costs, quality-adjusted life years (QALYs), and the incremental cost-effectiveness ratio (ICER). These were evaluated over a five-year time horizon from 2020. Secondary analyses explored longer horizons and alternative treatment stopping rules.

**Results:** Treatment with eptinezumab resulted in an average of 0.231 QALYs gained at a saving of £4,894 over 5 years, making eptinezumab dominant over best supportive care. I.e., better health outcomes and less costly. This result was also returned by the probabilistic analysis, and all alternative assumption scenarios under the same perspective. Univariate testing of inputs showed net monetary benefit was most sensitive to the number of days of productivity loss, and monthly salary.

**Conclusion:** This economic evaluation shows that from a UK societal perspective, eptinezumab is a cost-effective treatment in patients with ≥4 migraine days per month who have failed ≥3 other preventive treatments.

## P108 Burden of disease in patients with migraine unsuitable for triptans: a systematic literature review

### A. Gendolla^1^, R. B. Lipton^2,3^, L. Abraham^4^, A. Jenkins^4^, K. Hygge Blakeman^4^, P. Saccone^4^, J. Telfort^4^, I. Fotheringham^5^, I. Pustulka^5^, A. Engh^5^

#### ^1^Private Practice, Essen, Germany; ^2^Albert Einstein College of Medicine, New York, NY, United States; ^3^Montefiore Medical Center, New York, NY, United States; ^4^Pfizer R&D UK Ltd., Surrey, United Kingdom; ^5^Evidera Inc., London, United Kingdom

##### **Correspondence:** L. Abraham

*The Journal of Headache and Pain 2024,* **25(Suppl 1)**: P108

**Objective:** While triptans are the acute standard of care for moderate to severe migraine, they are unsuitable for many patients. This review was conducted to identify and evaluate published evidence on the burden of disease (BOD) among triptan-unsuitable patients.

**Methods:** Medline, Embase, and conference abstracts were searched (Jan 2012–Aug 2022) for evidence on patients with migraine unsuitable for triptans for any reason. Data from publications describing the clinical, humanistic, or economic BOD in this population were extracted. Evidence on the relative frequency and characteristics of this population was also collected and is presented separately.

**Results:** 1460 records were screened, resulting in 7 included publications (Figure 1). Six reported clinical burden (Figure 2), showing lack of efficacy and adverse effects to be among the reasons for triptan discontinuation. Two publications found significantly lower satisfaction in triptan-insufficient vs. sufficient responders (*p*<.001). In a third publication of patients who failed 1-2 triptans and received another triptan, 45% were dissatisfied with the final triptan. Among 6 publications on humanistic burden (Figure 2), 2 reported greater disability (MIDAS), impact of disease, and depression in patients who discontinued triptans vs. those with sustained triptan use. Two reported worse quality of life scores and utility values in triptan insufficient vs. sufficient responders. Three publications on economic burden (Figure 2) found greater migraine-related costs, work/activity impairment, and healthcare resource utilization in triptan insufficient vs. sufficient responders.

**Conclusion:** Although evidence was limited, this systematic review found a greater BOD in triptan non-responders vs. responders, and in patients who discontinued triptans vs. those who persisted. While further research is needed, current evidence suggests a high unmet need in triptan-unsuitable patients.

**Fig. 1 (Abstract P108):**
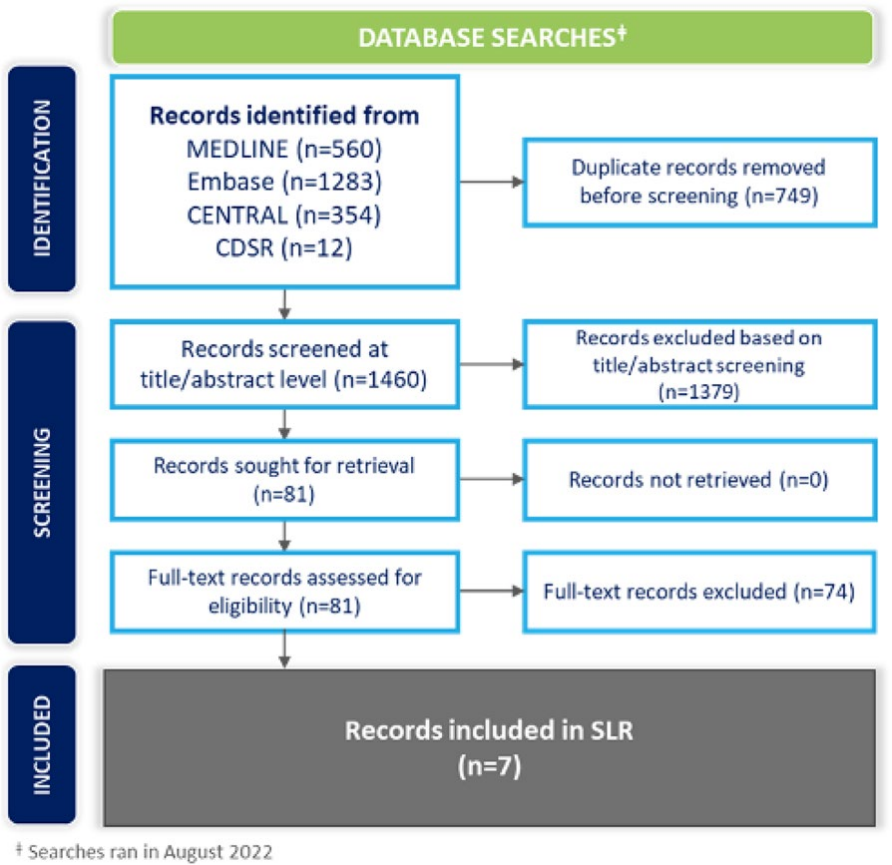
See text for description

**Fig. 2 (Abstract P108):**
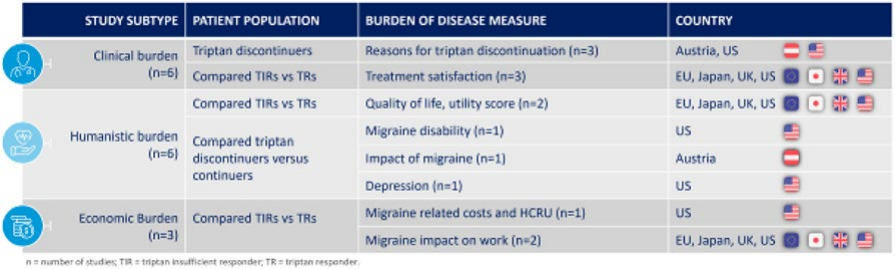
See text for description

## P109 Prevalence and functional impact of vestibular symptoms in patients with migraine: a cross-sectional study

### A. Jaimes Sanchez^1^, A. Gómez García^1^, O. Pajares^1^, P. Ibanez de la Cadiniere^1^, A. Acosta^1^, J. S. Rodriguez-Vico^1^, J. Serratosa^1^, J. Porta-Etessam^2^

#### ^1^Fundación Jiménez Díaz, Neurology, Madrid, Spain; ^2^San Carlos Clinical Hospital, Neurology, Madrid, Spain

##### **Correspondence:** A. Jaimes Sanchez

*The Journal of Headache and Pain 2024,* **25(Suppl 1)**: P109

**Objective:** To assess the prevalence and functional consequences of vestibular symptoms in individuals diagnosed with migraine.

**Methods:** This study utilized a cross-sectional design and survey to collect data from patients recruited from the headache unit and general neurology clinic.

**Results:** A total of 244 participants were included in the study, with 164 (66.9%) with migraine and 80 (32.7%) serving as controls. The participants had a mean age of 40.6 (+/-13.5) years, with 75% of them being female. Table 1 presents the baseline characteristics. Among migraine patients, a majority experienced varying degrees of anxiety (minimal 19.5%, mild 41.5%, moderate 22%, severe 17.1%) as measured by the GAD-7 scale. Persistent photophobia was reported by 30.7% of migraine patients, persistent phonophobia by 23.3%, and persistent osmophobia by 26.4%. Vestibular symptoms were more prevalent in the migraine group compared to the control group (fig 1), with 53.7% reporting dizziness vs. 15% in controls (OR 6.56, 95% CI 3.3-13; *p*<0.001), and 44.5% reporting vertigo vs. 36.3% in controls (OR 1.4, 95% CI 0.8-2.4; *p*=0.27). Among the 88 migraine patients with vestibular symptoms, a significant percentage experienced vestibular symptoms as prodromal manifestations of migraine (dizziness 17.7% and vertigo 15.9%). Additionally, the Dizziness Handicap Inventory (DHI) revealed a severe handicap at the functional level in 30.7%, at the physical level in 45.5%, and at the emotional level in 26.1%. Logistic regression analysis demonstrated a statistically significant association between dizziness, and persistent photophobia and higher values on the HIT-6 scale.

**Conclusion:** Migraine patients have a sixfold higher probability of experiencing dizziness compared to healthy controls. Factors such as persistent photophobia and higher values on the HIT-6 scale may further increase the likelihood of experiencing dizziness. According to DHI scale, these symptoms have a significant impact on quality of life.

**Table 1 (Abstract P109):**
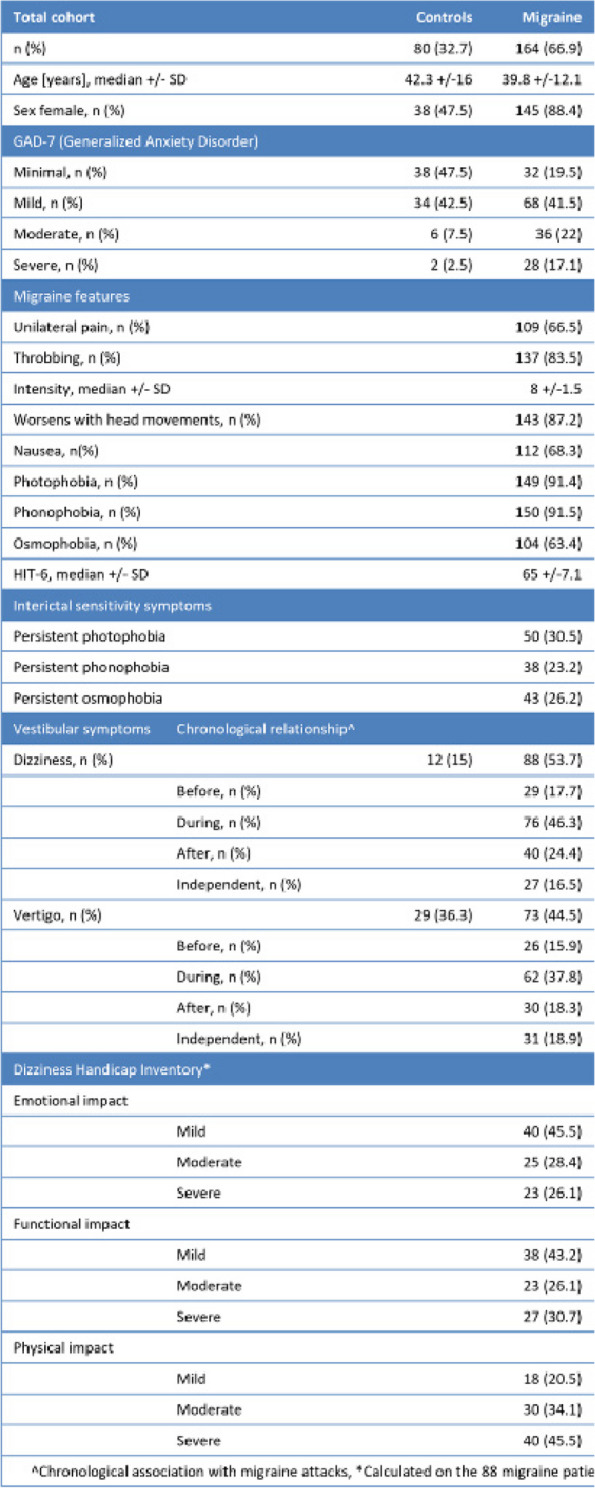
See text for description

**Fig. 1 (Abstract P109):**
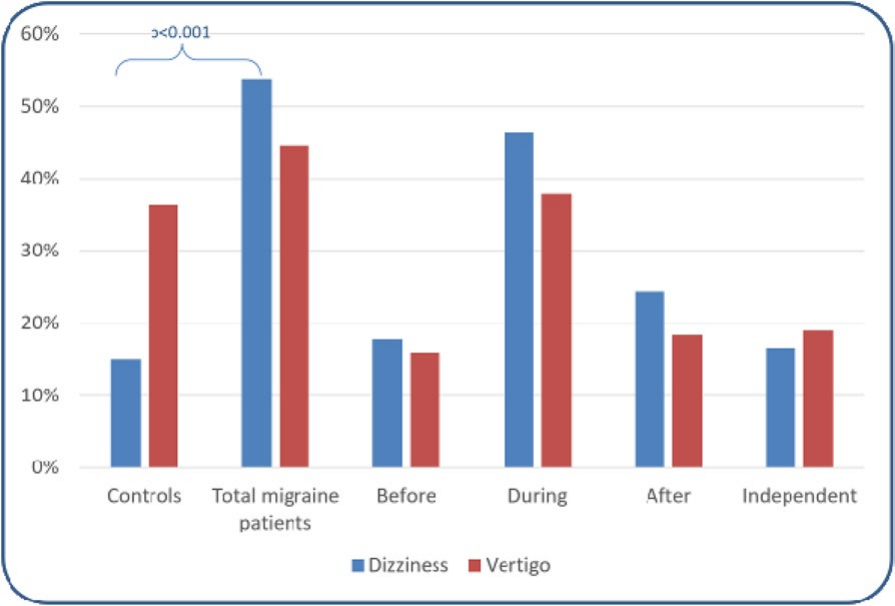
See text for description

## P110 The economic and humanistic burden of nausea and vomiting in migraine: a targeted literature review

### F. O'Sullivan^1^, L. Powell^1^, L. Harris^2^, G. L'Italien^2^, L. Abraham^3^

#### ^1^Broadstreet HEOR, Vancouver, Canada; ^2^Biohaven Pharmaceuticals Inc., New Haven, CT, United States; ^3^Pfizer R&D UK Ltd., Tadworth, United Kingdom

##### **Correspondence:** L. Abraham

*The Journal of Headache and Pain 2024,* **25(Suppl 1)**: P110

**Objective:** The objective of this study was to characterize the economic and humanistic burden of nausea and/or vomiting (NV) in migraine. Most standard of care (SOC) treatments for acute migraine attacks are oral tablet formulations, potentially posing as a barrier to those experiencing severe NV who are unable to eat or drink and could contribute to delayed treatment. It is important to understand the impact of these symptoms from both a humanistic and economic perspective in the current treatment landscape.

**Methods:** Searches were conducted in May 2022 using Medline, Embase, and supplemented with internet sources and references identified in relevant articles. Search terms included terms related to “Migraine”, “migraine disorders”, “headaches”, “nausea”, “vomiting”, “emesis” and economic and quality of life (QOL) indicators. Outcomes of interest included prevalence of NV, and measures of QOL, direct health care costs, and indirect costs, such as absenteeism and presenteeism measured among patients with migraine related NV. Studies with a non-NV migraine comparison group were of highest interest.

**Results:** 16 articles were included and examined in detail. The reported prevalence of nausea or vomiting among patients with migraine was 65-90% and 30-70%, respectively. Ability to ingest oral medication was impacted among 27-43% of those with NV. NV was associated with elevated direct healthcare costs and indirect costs. Compared to those without NV, patients with NV visited the emergency department (ED) more frequently, and had 26.3% higher mean ED costs and 27.9% higher annual absenteeism costs. Patients with NV were also more likely to experience negatively impacted sleep, missed activities, depression, and migraine-related disability, contributing to poorer QOL.

**Conclusiun:** These findings demonstrate the increased economic and QOL burden imposed by NV in migraineurs and highlight some of the challenges managing these patients with SOC oral options. Treatment with effective non-oral therapy options may reduce the significant burden associated with these symptoms.

## P111 Persistent headache after Covid-19 infection

### C. Dunne^1^, M. Boldingh^1^, T. Popperud^1^, E. A. Høgestøl^1^, B. E. Halvorsen^2,3^, M. Beyer^3,4^, H. F. Harbo^1,3^, M. B. Argren^1,2^, A. H. Aamodt^5,1^

#### ^1^Oslo University Hospital, Neurology, Oslo, Norway; ^2^Oslo University Hospital, Research Institute of Internal Medicine, Oslo, Norway; ^3^University of Oslo, Institute of Clinical Medicine, Faculty of Medicine, Oslo, Norway; ^4^Oslo University Hospital, Radiology Department, Oslo, Norway; ^5^NorHEAD, Norwegian University of Science and Technology, Neuromedicine and Movement Science, Trondheim, Norway

##### **Correspondence:** C. Dunne

*The Journal of Headache and Pain 2024,* **25(Suppl 1)**: P111

**Objective:** The present study aimed to describe headache characteristics during follow-up at six and 12 months in participants with persisting headache after Covid-19 infection.

**Methods:** The Norwegian study of nervous system manifestations and sequelae after Covid-19 (NeuroCovid) was a prospective observational multi-center study of Norwegian patients with continued neurological symptoms after Covid-19 (Figure 1). All participants were seen by experienced neurologists who assess neurological symptoms and signs in relation to COVID-19 and were also to a complementary visit by neuropsychologist and psychiatrist to assess neuropsychological signs and psychiatric diagnosis and symptoms in more details. Additionally, the participants had brain MR examinations and comprehensive biomarker assessments. In this sub study participants who reported having a headache diagnosis at the six-month visit at Oslo University Hospital were included. Detailed headache characteristics at 6 and 12-months follow-up after Covid-infection was assessed.

**Results:** Among 123 participants in the NeuroCovid study at Oslo University Hospital, 36 participants reported persistent headache at six months. There was a strong female preponderance (80 %). Median (IQR) age was 47 (29-63) years, and median (IQR) body mass index was 24 (19-35) kg/m2. The headache phenotype was migraine in more than half (53 %) and 48 % had a history of headache. There was a clear trend of decreasing headache severity, intensity, duration, NRS and frequency from six months to 12 months. In 8 %, headache had resolved after 12 months whereas 69 % still had headache with improvement. However, in 23% headache persisted with no improvement after 12 months. Biomarker assessments will be presented and compared to pateints with persistent headache after Covid vaccines (the CovaxHEAD study).

**Conclusion:** Persistent headache is common in a cohort of patients developing neurological manifestations after Covid-19 infection. Characteristics of the headaches were mostly classified as migraine-like and showed a decreasedsymptom burden over time. A history of headache may increase risk towards developing persistent headache after acute Covid-19 infection.

**Fig. 1 (Abstract P111):**
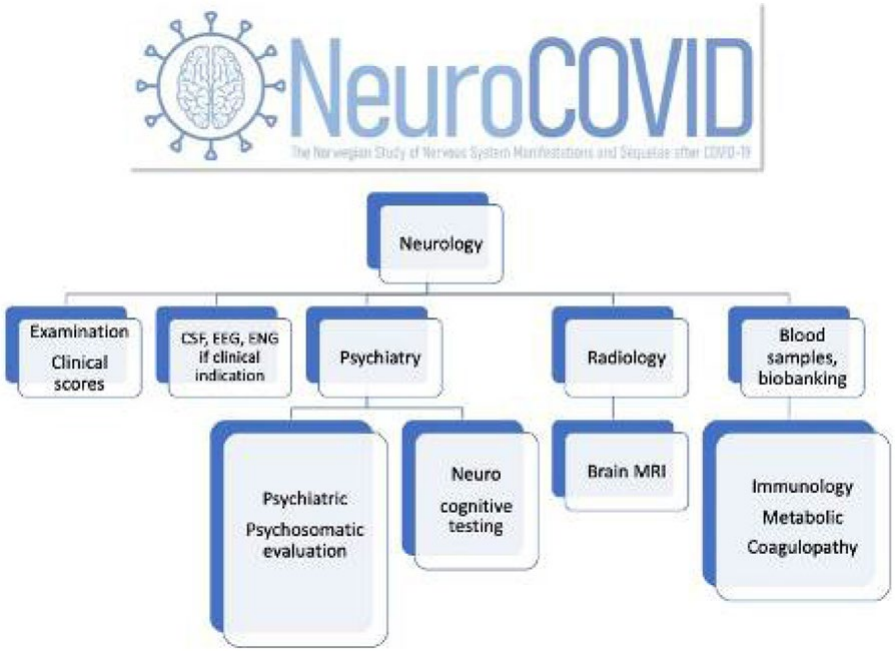
See text for description

## P112 Design of the ContemporAry ProspecTive Understanding of migraine Real-world Evidence (CAPTURE) study

### M. Lanteri-Minet^1^, C. Tassorelli^2^, M. Ashina^3,4^, P. J. Goadsby^5^, Z. Katsarava^6^, M. Matharu^7,8^, M. Peres^9^, R. J. Stark^10^, J. Ailani^11^, E. Leroux^12^, L. Delahaye^13^, H. Ha^14^, P. Pozo-Rosich^15,16^

#### ^1^CHU Nice and Côte Azur University, Pain Department and FHU InovPain, Nice, France; ^2^C. Mondino Foundation and University of Pavia, Headache Science Centre, Pavia, Italy; ^3^Copenhagen University Hospital – Rigshospitalet, Danish Headache Center and Department of Neurology, Copenhagen, Denmark; ^4^University of Copenhagen, Department of Clinical Medicine, Copenhagen, Denmark; ^5^King's College London, London, United Kingdom; ^6^Evangelical Hospital Unna, Unna, Germany; ^7^Queen Square Institute of Neurology, London, United Kingdom; ^8^Norwegian University of Science and Technology, Trondheim, Norway; ^9^University of São Paulo, São Paulo, Brazil; ^10^Alfred Hospital and Monash University Melbourne, Melbourne, Australia; ^11^MedStar Georgetown University Hospital, Washington, DC, United States; ^12^Brunswick Medical Center, Montreal, Canada; ^13^AbbVie, Rungis, France; ^14^AbbVie, Toronto, Canada; ^15^Vall d’Hebron Hospital & Research Institute, Universitat Autonoma de Barcelona, Headache Unit, Department of Neurology, Barcelona, Spain; ^16^Universitat Autonoma de Barcelona, Headache and Neurological Pain Research Group, Vall d’Hebron Institute of Research, Barcelona, Spain

##### **Correspondence:** L. Delahaye

*The Journal of Headache and Pain 2024,* **25(Suppl 1)**: P112

**Objective:** Insufficient longitudinal evidence is available describing the impact of migraine. This global study will assess how headache/migraine frequency, disability, and treatment patterns change over a 2-year period in individuals being treated for migraine.

**Methods:** ContemporAry ProspecTive Understanding of Migraine Real-world Evidence Study (CAPTURE) is a 2-year, global, observational, longitudinal, prospective study that will enroll individuals >=18 years of age being treated for migraine. Participants will be stratified into 3 baseline monthly headache day (MHD) cohorts: 4-7 days; 8-14 days; >=15 days. Eligibility criteria include men/women diagnosed with migraine for >=1 year, <=50 years of age at migraine onset, taking >=1 migraine medication, and a history of >=4 MHDs in the 3 months prior to screening, which was confirmed prospectively with headache e-diary data in the 30-day screening period. Key study design elements and endpoints are depicted in the Figure and Table.

**Results:** The target enrolled sample size is approximately 2000 (cohort 1: 30% [*n*=600]; cohorts 2-3: 35% [*n*=700 each]). Patients will be enrolled from approximately 135 sites in 15 countries. The target for first patient enrollment is early 2023 and the last patient completion is anticipated to be late 2025. The study will collect clinical outcomes, patient-reported outcomes, and changes in the number of patients among the migraine cohorts. Only the methodology of this study will be described.

**Conclusion:** CAPTURE will provide a better understanding of headache/migraine frequency, disability, and treatment patterns in individuals being treated for migraine and will be one of the first global prospective longitudinal studies of its kind.

**Fig. 1 (Abstract P112):**
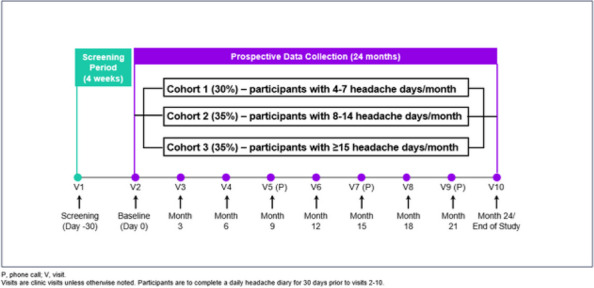
CAPTURE Study Design

**Table 1 (Abstract P112):**
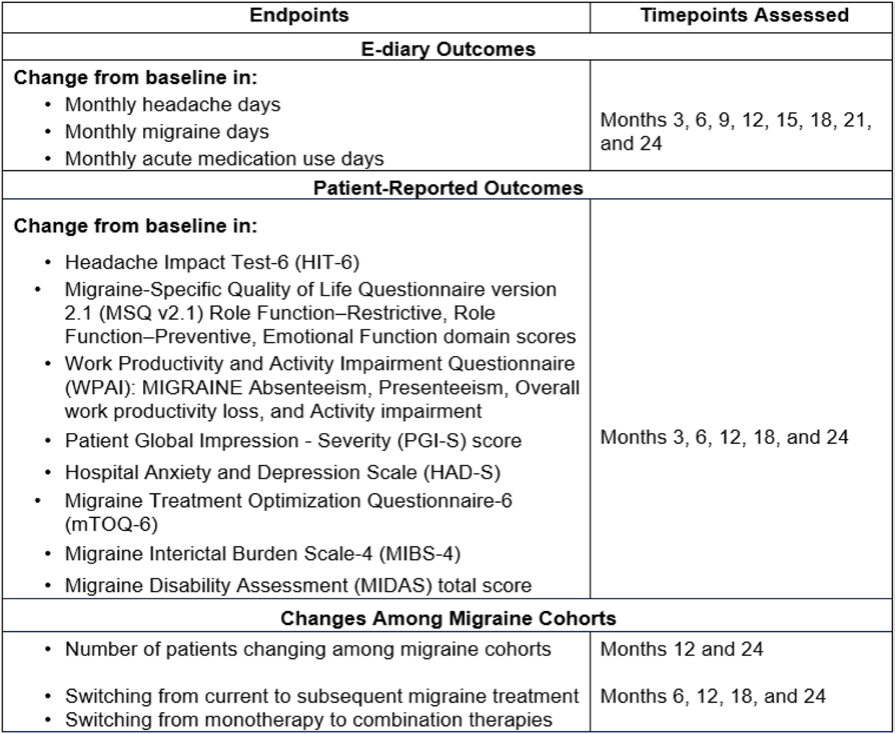
Study Endpoints

## P113 The burden of migraine: 3-month findings from the TRIUMPH (preventive TReatment of mIgraine: oUtcoMes for Patients in Real-world Healthcare Systems) study

### C. Tassorelli^1,2^, M. Matharu^3^, S. Joshi^4^, S. Ashina^5^, R. L. Robinson^6^, D. Novick^6^, C. Vallarino^6^, L. Viktrup^6^, M. Vincent^6^

#### ^1^University of Pavia, Department of Brain and Behavioral Sciences, Pavia, Italy; ^2^IRCCS Mondino Foundation, Headache Science and Neurorehabilitation Centre, Pavia, Italy; ^3^UCL Queen Square Institute of Neurology and National Hospital for Neurology and Neurosurgery, Headache and Facial Pain Group, London, United Kingdom; ^4^Community Neuroscience Services, Westborough, MA, United States; ^5^Harvard Medical School, Comprehensive Headache Center, Department of Neurology and Anesthesia, Beth Israel Deaconess Medical Center, Boston, MA, United States; ^6^Eli Lilly and Company, Indianapolis, IN, United States

##### **Correspondence:** M. Vincent

*The Journal of Headache and Pain 2024,* **25(Suppl 1)**: P113

**Objective:** To describe migraine burden in real-world clinical practice 3 months after initiating/switching to a new preventive migraine treatment such as Galcanezumab, other CGRP mAbs, traditional oral migraine preventive medications (TOMP), or botulinum toxin A/B.

**Methods:** Adult pts diagnosed with migraine switching to/initiating preventive treatment were enrolled in the study. Self-administered questionnaires such as the Migraine Disability Assessment Test (MIDAS), Migraine-Specific Quality of life questionnaire (MSQ) v2.1, Patient Global Impression of Severity (PGI-S), Work Productivity and Activity Impairment Questionnaire (WPAI) were used. In this interim, preliminary, descriptive analysis (data: 02/2020-08/2022), categorical variables were summarized using proportions and continuous variables were summarized using means with standard deviations (SD) at baseline, at 3 months, and mean change from baseline.

**Results:** The mean (SD) number of monthly migraine headache days was 13.2 (7.2) at baseline. The Galcanezumab group tended to have worse baseline scores than TOMP, better than botulinum toxin, and similar to other CGRP mAbs across all measures of migraine burden. At 3 months, the Galcanezumab group had the largest mean (SD) change in MSQ v2.1 scores (Table 1), MIDAS score (-21.2 [45.8]), WPAI domains of presenteeism (-22.4 [29.1]), work productivity (-24.2 [30.8]), and activity impairment (-23.1 [30.7]). Mean (SD) change in PGI-S scores at 3 months were similar across groups (Galcanezumab: -0.4 [1.3], other CGRP mAbs: -0.4 [1.2], TOMP: -0.5 [1.4], botulinum toxin: -0.4 [1.3], other approved treatments -0.5 [1.3]).

**Conclusion:** Although all pts had high but different levels of baseline migraine burden, those initiating Galcanezumab tended to present greater numerical improvement from baseline for the majority of scales vs other drug classes. Further substantiation of these findings depends on weighted statistical analyses of pt reported outcomes from future timepoints with a larger sample size.

Previously presented at American Headache Society - 65th Annual Scientific Meeting.

**Table 1 (Abstract P113):**
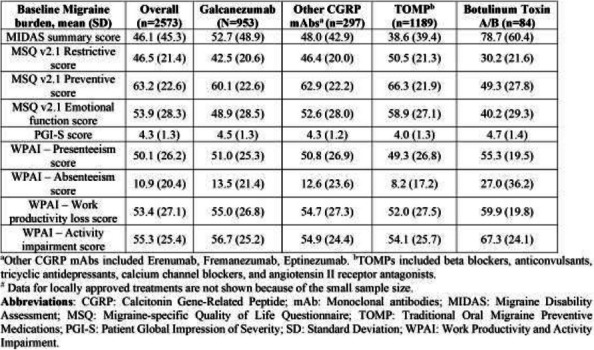
(See text for description)

## P114 Understanding the burden of persistent post-COVID headache

### S. Horváthová^1^, O. Duraníková^1^, P. Valkovič^1^, P. Sabaka^2^

#### ^1^Comenius University in Bratislava, 2nd Department of Neurology, Bratislava, Slovakia; ^2^University Hospital in Bratislava, Department of Infectology and Geographical Medicine, Bratislava, Slovakia

##### **Correspondence:** S. Horváthová

*The Journal of Headache and Pain 2024,* **25(Suppl 1)**: P114

**Objective:** Although COVID-19 primarily affects the respiratory system, it can also result in neurological symptoms. Post-COVID persisting headache refers to headaches that persist beyond the acute phase of the infection and continue for an extended duration. Our study aimed to determine number of people suffering from post-COVID headache and its impact on their life.

**Methods:** We conducted a retrospective study on patients hospitalized with COVID-19 from March 2020 to April 2021 at the University Hospital in Bratislava. Our group consisted of 634 patients, from which 339 did not fulfil inclusion criteria (age, refused participation). We collected data from 295 remaining patients by self-administered questionnaire after 12-15 months after their hospital discharge. Firstly, we assessed the presence of headaches during their COVID-19 infection. Then we focused on the characteristics, location, intensity, accompanying symptoms, response to pain relievers, and impact on their daily lives.

**Results:** Among our group, 34.6% of patients (*n*=102,56 women) experienced headaches. Headache as the most bothersome symptom was described by 31.4%. In terms of recovery, headaches disappeared along with other symptoms in 34.3% of patients, while 11.8% of responders reported resolution within two weeks and in 12.7% it took up to a month. Currently, 41.2% of patients still experience headaches. Among those with persistent headaches, 36.3% of patients found over-the-counter painkillers ineffective. The most common phenotype among this group was migraine-like headaches, accounting for 42.4%. Only 29.4 % of patients reported no limitations in their daily lives, while 25.5% experienced severe limitations and 45.1% were limited in some activities

**Conclusion:** Persistent post-COVID headache has a substantial impact on individuals' well-being, healthcare and society overall. It is essential to raise awareness, conduct research, and develop effective strategies to manage these headaches and minimize their long-term health effects.

## P115 Clinical and diagnostic characteristics of headache in patients with small vessel disease

### V. Vershuta

#### Sechenov University, Neurology, Moscow, Russian Federation

*The Journal of Headache and Pain 2024,* **25(Suppl 1)**: P115

**Objective:** Small vessel disease (SVD) is one of the most pressing problems of modern medicine. The leading complaint in SVD is headache (HA).

Aim: determination of clinical and diagnostic characteristics of HA in SVD.

**Methods:** The study included 72 patients suffering from SVD. The patients were divided into two groups: group 1 - 44 patients suffering from HA, control group -28 patients without HA. Patients underwent the questionnaire survey: MOCA test, Hospital Anxiety and Depression Scale, sleep questionnaire, MRI of the brain.

**Results:** Tension-type headache (TTH) was detected in 27 (37.5%) patients, migraine -11 (15.3%), migraine and TTH - 6(8.3%). Cognitive impairment in migraine occurred in 8(72.7%) people, in TTH in 17(62.9%), in migraine and TTH in 5(83,3%). In patients with TTH, sleep was disturbed in 18 (66.7%) cases, with migraine in 9(81.8%), with migraine and TTH in 6(100%). Anxiety in TTH patients was diagnosed in 21(77.8%) patients, migraine - 6(54.5%), depression in patients with TTH - 10(37%), with migraine - 7(63.6%). With migraine and TTH, anxiety was diagnosed in 4 (66.7%) cases, depression in 5(83.3%). According to MRI of the brain, white matter pathology corresponding to grade 3 Fazekas was diagnosed in 6(54.5%) patients with migraine, with TTH in 6(22.2%), with TTH and migraine -4(66, 7%).

**Conclusion:** According to our study, 44 (61.1%) people suffering from SVD complained of HA, while the diagnosis of HA was previously established only in 11 (15.3%). TTH was the most common among patients with SVD (37.2%). Among patients with migraine cognitive impairment was 9.8% more common than in TTH, as well as more pronounced white matter changes according to MRI of the brain: Fazekas grade 3 was 27.8% more common. Anxiety and sleep disorders prevailed among patients with TTH, depression among patients with migraine and TTH. Thus, the majority of patients with SVD suffered from primary HA, the course of which was aggravated by emotional disorders and sleep disturbance.

## P116 Migraine: A crash in slow motion – a qualitative study

### W. Laughey^1,2^, I. Lodhi^1^

#### ^1^Reckitt, Medical Sciences, Hull, United Kingdom; ^2^Hull York Medical School, Health Professions Education, York, United Kingdom

##### **Correspondence:** W. Laughey

*The Journal of Headache and Pain 2024,* **25(Suppl 1)**: P116

**Objective:** To understand the burden of migraine from the perspectives and lived experiences of people who have the disease and health care professionals (HCPs) with experience in the field. Also, to explore the role for empathy in migraine.

**Methods:** One to one semi-structured interviews were conducted with six migraine patients and three HCPs.

**Results:** Researchers generated themes, two of which centre on the burden of migraine. These were a sense of *isolation and feeling mis-understood* and the experience of a *lack of control* – whatever actions they take, migraine often requires sufferers to find refuge in sleep, rather like watching a car crash in slow motion, but being unable to influence the result. The *need for empathy* for migraine sufferers was a third theme, especially given the impact on emotions and mental health. The sense of being misunderstood had particular impact at work, with the potential to foster a lack of trust in relationships due to the under appreciation of attacks. Lack of control was evident not just during the attack but also between attacks, with patients fearing the possibility of attacks interrupting social and family engagements, also affecting relationships.

**Conclusion:** Despite the efforts of patient groups and industry campaigns, there remains an underappreciation of the impact of migraine at a human level. Most sufferers are women, and due to the "gender pain gap" pain in women is more likely to be dismissed. Sufferers may be suspected of "social cheating" – using migraine as an excuse to step away from responsibilities. In simple terms, there is an empathy gap. Empathy researchers refer to the concept of "compassionate curiosity". Future studies should consider how we foster compassionate curiosity towards people with migraine so the condition can be better understood.

## P117 Network meta-analysis comparing atogepant vs. CGRP mAbs on headache impact test-6 total scores in episodic and chronic migraine

### J. Ailani^1^, L. Dupont-Benjamin^2^, S. Ashina^3^, T. Takeshima^4^, A. Lalla^5^, I. Ubamadu^6^, G. Pietri^6^, P. Gandhi^7^, R. B. Halker Singh^8^

#### ^1^MedStar Georgetown University Hospital, Washington, DC, United States; ^2^AbbVie, Courbevoie, France; ^3^Harvard Medical School, Beth Israel Deaconess Medical Center, Department of Neurology and Department of Anesthesia, Critical Care and Pain Medicine, Boston, MA, United States; ^4^Tominaga Hospital, Department of Neurology, Headache Center, Osaka, Japan; ^5^AbbVie, Irvine, CA, United States; ^6^AbbVie, London, United Kingdom; ^7^AbbVie, Madison, NJ, United States; ^8^Mayo Clinic, Scottsdale, AZ, United States

##### **Correspondence:** S. Ashina

*The Journal of Headache and Pain 2024,* **25(Suppl 1)**: P117

**Objective:** Conduct a network meta-analysis (NMA) of atogepant vs calcitonin gene–related peptide (CGRP) monoclonal antibodies (mAbs) on Headache Impact Test-6 (HIT-6) total scores in episodic migraine (EM) and chronic migraine (CM).

**Methods:** A clinical systematic literature review was conducted to identify data for branded preventive treatments approved for EM and CM (database searches were initially performed on May 11, 2020 and later updated on Sept 1, 2022 to capture studies conducted after 2020). A Bayesian NMA was conducted using a fixed effects model or a random effects model with vague prior distribution (between-study standard deviation; ie, heterogeneity ~U[0,5]). Choice of model was based on best fit statistics. NMA was performed for the overall EM and CM populations. Based on available data, evaluated treatments included atogepant (10mg [EM only], 30mg [EM only], 60mg once daily), erenumab (70mg monthly, 140mg monthly), fremanezumab (225mg monthly, 675mg quarterly), and eptinezumab (100mg quarterly, 300mg quarterly). Improvement from baseline in HIT-6 total score was evaluated from EM and CM trials. Results are presented as model estimates of mean difference and 95% credible interval for atogepant vs the CGRP mAbs.

**Results:** Based on best fit statistics, a random effects model with vague prior distribution was used for EM and a fixed effects model was used for CM. In the EM population, all doses of atogepant showed a numerically, but not statistically significant, larger improvement in HIT-6 total score vs erenumab 70mg monthly (Figure 1). In the CM population, atogepant 60mg once daily showed a numerically larger improvement in HIT-6 total score vs all non-placebo treatments except eptinezumab 300mg quarterly (differences were not statistically significant; Figure 2).

**Conclusion:** All atogepant doses showed a numerically larger or smaller improvement in HIT-6 total scores compared with CGRP mAbs. None of the differences were statistically significant.

**Fig. 1 (Abstract P117):**
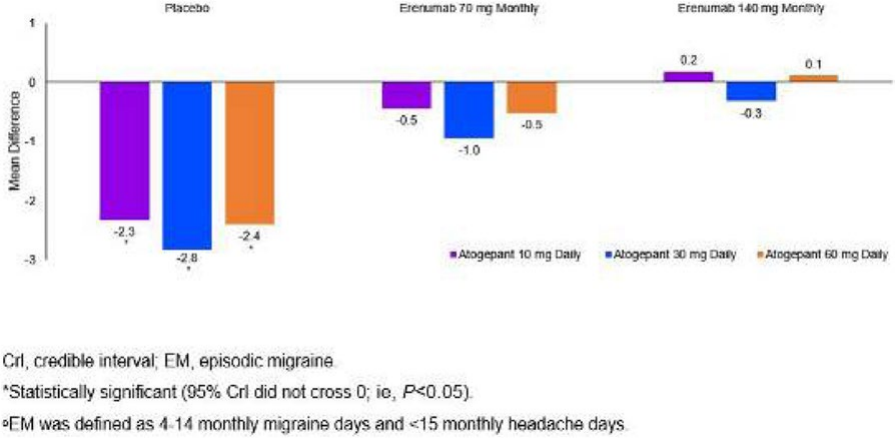
Change from Baseline in HIT-6 at Week 12 Among the Overall EM^a^ Population: Comparative Treatment Effect of Atogepant vs Erenumab

**Fig. 2 (Abstract P117):**
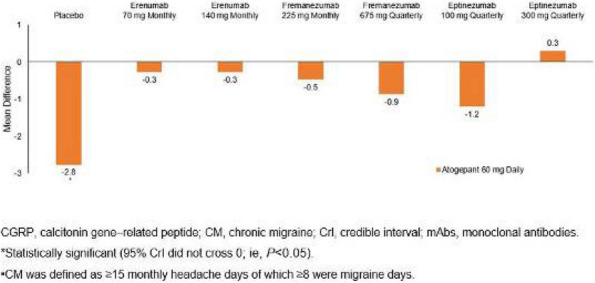
Change From Baseline in HIT-6 Total Score at Week 12 Among the Overall CM^a^ Population: Comparative Treatment Effect of Atogepant vs CGRP mAbs

## P118 Patient experiences and preferences for non-oral acute migraine treatments: a qualitative interview study

### T. Smith^1^, G. Tickler^2^, H. Skrobanski^2^, L. Harris^3^, G. L'Italien^3^, J. Coulter^4^, L. Abraham^4^, S. H. Lo^2^

#### ^1^Study Metrix LLC, St. Peters, MO, United States; ^2^Acaster Lloyd Consulting Ltd, London, United Kingdom; ^3^Biohaven Pharmaceuticals Inc., New Haven, CT, United States; ^4^Pfizer, Tadworth, United Kingdom

##### **Correspondence:** L. Abraham

*The Journal of Headache and Pain 2024,* **25(Suppl 1)**: P118

**Objective:** To understand patient views and experiences with non-oral acute migraine treatments, and patient preferences for different features of non-oral acute treatments.

**Methods:** Migraine patients who self-reported use of non-oral acute migraine treatments in the last three months were recruited in the United States and United Kingdom to participate in semi-structured interviews. Data were analysed using content and thematic analysis; data saturation was also recorded.

**Results:** In total, 20 participants were interviewed of whom a majority (80%) had received a chronic migraine diagnosis. Migraine patients commonly reported starting non-oral acute treatment because they were perceived to be faster acting and more effective than orals. Many participants also reported difficulties with oral treatment options. Participants expressed a strong preference for effective treatments, particularly those that offered fast relief and allowed them to return to their everyday life quickly. They also wanted a treatment that provided reliable relief regardless of when the medication is taken during an attack, and was not limited in the number of times it could be used per month. While participants desired a treatment with no or minimal side effects, several stated that they were willing to accept mild side effects if the medication was effective. The risk of medication overuse headache (MOH) was also a concern. Participants preferred treatment to be quick, simple, convenient, and easy to administer and therefore preferred pre-filled over non-pre-filled injectables or intranasal sprays.

**Conclusion:** Migraine patients typically start using non-oral acute treatments because they need better efficacy, faster relief, or have difficulties with oral options. Patients taking non-orals express a preference for acute migraine treatments that are reliably effective for pain relief and return to normal functioning, fast-acting, easy to use, have minimal side effects, and no risk of MOH.

## P119 Treating acute migraine attacks and preventing episodic migraine in UK primary care: insights using IQVIA OMOP data

### S. L. Collings^1^, X. Lin^2^, R. Pawinski^1^, M. Shang^2^, S. Seager^2^, E. Hamson^1^, S. Afridi^3^, A. Ahern^1^

#### ^1^Pfizer, Tadworth, United Kingdom; ^2^IQVIA, RWS, Durham, United Kingdom; ^3^Guy’s & St Thomas’ NHS Foundation Trust, London, United Kingdom

##### **Correspondence:** S. L. Collings

*The Journal of Headache and Pain 2024,* **25(Suppl 1)**: P119

**Objective:** Rapid assessment of treatment landscape and time spent on treatment for acute migraine and prevention of episodic migraines in the UK.

**Methods:** Using UK primary care data from the IQVIA UK IMRD THIN database, two cohorts of patients newly diagnosed with migraine were identified: a recent cohort (diagnosed in 01/01/2018-31/05/2021, *n*=17,738) and a larger cohort (diagnosed in 01/01/2010–30/09/2017, *n*=119,918). Migraine was defined using a clinically reviewed list of SNOMED codes. Index date was defined as date of first migraine diagnosis code. Patients were followed up from index date until last observation in the database.

The proportion of patients prescribed treatment was reported overall and at class level in the recent cohort. Length of time on continuous treatment (prescriptions received within 12 months of each other) was reported for triptans, acute treatments only and acute and preventatives in the larger cohort.

**Results:** In the recent cohort, 48.0% received triptans, 32.6% non-steroidal anti-inflammatories, and 27.2% analgesics/antipyretics. Experience with >1 type of triptan was observed in <5%. The most prescribed preventative treatments were beta-blockers (23.2%) and tricyclic antidepressants (17.3%). Anticonvulsants were prescribed to <1%.

In the larger cohort, 23,448 patients were continuously prescribed triptans, of which 55.9%, 28.3%, and 15.7% were prescribed for <2, 2-5, and ≥5 years respectively. While, those continuously prescribed only acute treatments (*n*=23,582), were 56.5%, 31.3%, and 12.2% were prescribed for <2, 2-5, and ≥5 years, respectively. Furthermore, patients continuously prescribed acute and preventative treatments (*n*=35,028), were 32.8%, 39.4%, and 27.9% were prescribed for <2, 2-5, and ≥5 years, respectively.

**Conclusion:** Triptans are the most prescribed treatment, with the majority of patients remaining on treatment for <2 years but a notable proportion for >5 years and similar proportions observed for other acute treatments.

## P120 Childhood primary stabbing headache: a double center study

### G. Monte^1^, L. Papetti^1^, F. Ursitti^1^, G. Sforza^1^, S. Tarantino^1^, M. Checchi Proietti^1^, D. D'Agnano^2^, V. Sciruicchio^2^, M. Valeriani^1^

#### ^1^Bambino Gesù Children's Hospital, Neuroscience, Rome, Italy; ^2^Children Epilepsy and EEG Center, PO, San Paolo ASL, Bari, Italy

##### **Correspondence:** G. Monte

*The Journal of Headache and Pain 2024,* **25(Suppl 1)**: P119

**Objective:** Primary stabbing headache (PSH) is an idiopathic headache disorder characterized by head pain occurring as a transient and localized single stab or a series of stabs. The aim of this study was to examine the characteristics of PSH in childhood.

**Methods:** In this retrospective study we included 60 patients seen at two headache clinics (Rome and Bari) between 2016 and 2022. A headache-focused history was obtained. All patients had normal neurological examination. PSH was defined according to ICHD-3 and we decided to use the term PSH also for probable PSH.

**Results:** Twenty-three patients were male and the median age at disease onset was 8 years (range 3-17). Stabs recurred with irregular frequency and the duration varied from few seconds to 30 minutes. Stabs were located in a variety of regions of the cranium. According to ICHD-3, thirty-one patients had a diagnosis of probable PSH due to a stabbing duration longer than few seconds (> 3 seconds). Twenty-five patients (42%) underwent neuroimaging and all were normal. Only five children reported a limitation of activities of daily living and none had a chronic pattern. Forty-seven patients (78%) had a family history of primary headache, especially migraine, and forty-three had episodic syndromes (i.e. infantile colic, benign paroxysmal vertigo, motion sickness, recurrent abdominal pain, cyclic vomiting).

**Conclusion:** Presentation of childhood PSH varies widely. As seen in previous studies, a lot of patients reported a stab duration longer than few seconds and this might suggest that the current ICHD-3 may be in need of adjustment to be applicable for children. The high frequency of associated migraine and episodic syndromes could suggest a common pathophysiological mechanism between PSH and migraine. In pediatric age, the far higher prevalence in very young children may also suggest that PSH represents a precursor of migraine. Large studies with long-term follow-up are needed to improve understanding of this condition.

## P121 Workplace disability and anti-CGRP monoclonal antibodies: a study on absenteeism

### A. Granato, L. Bartole, G. Garascia, P. Manganotti

#### Neurology Unit, Headache Centre, Department of Medicine, Surgical and Health Sciences, ASUGI, Trieste, Italy

##### **Correspondence:** A. Granato

*The Journal of Headache and Pain 2024,* **25(Suppl 1)**: P121

**Objective:** High-frequency episodic migraine and chronic migraine carry deeply impact workplace disability and in particular absenteeism and presentism. Anti-GCRP monoclonal antibodies improve frequency, intensity and the disability of migraine. Aim of the study is to evaluate workplace absenteeism and presentism before and after anti-CGRP monoclonal treatment.

**Methods:** We enrolled workers with diagnosis of high-frequency episodic migraine or chronic migraine (ICHD-3 criteria). Patients were treated with anti-GCRP monoclonal antibodies for one year according to Italian policy of drug administration (AIFA) criteria. No patient takes other prophylactic therapies. We used MIDAS to calculate the disability and the days of workplace absenteeism and presentism before (t0) and after 12 months anti-CGRP monoclonal treatment (t1).

**Results:** Sixty-two patients were enrolled (61.3% chronic migraine, 38.7% high-frequency episodic migraine; 74.2% F and 25.8% M; mean age 46±10 y.o.). Anti-GCRP monoclonal antibodies used were erenumab (36.5%), fremanezumab (47.6%) and galcanezumab (15.9%). There was a significant reduction of MIDAS at the end of treatment (t0= 95±39 vs t1= 20±18; *p*<0.001). Absenteeism and presentism reduced significantly at t1 (absenteeism: 7 [1-19] days at t0 vs 1 [0-3] days at t1 (*p*=0.002); presentism: 32 [19-47] days at t0 vs 8 [0-21] days at t1 of presentism (*p*<0.001)).

**Conclusion:** Workplace absenteeism and presentism improve with anti-CGRP monoclonal antibodies therapy.

## P122 Clinical characteristics of headache related to COVID-19 infection and vaccination in healthcare personnel: insights from a descriptive cross-sectional study

### C. Trevino-Peinado, P. Torres

#### Hospital Severo Ochoa, Leganés, Spain

##### **Correspondence:** C. Trevino-Peinado

*The Journal of Headache and Pain 2024,* **25(Suppl 1)**: P122

**Objective:** The aim of this study was to assess the clinical characteristics of headache associated with COVID-19 infection in healthcare personnel, taking into account acute SARS-CoV-2 infection, vaccination status, and treatment modalities. Additionally, we investigated the impact of different waves of the pandemic on the incidence of headache and evaluated persistent headache.

**Methods:** This descriptive cross-sectional study was conducted in a single center. A survey comprising 50 items was distributed to collect demographic information, headache characteristics, acute and preventive treatments, types of vaccines administered, and their association with headache. Frequency measures were employed for data analysis.

**Results:** A total of 392 healthcare professionals, predominantly female (71%), participated in the study. Headache associated with COVID-19 infection was primarily characterized as frontal, bilateral, pressing, and of moderate intensity. Nonsteroidal anti-inflammatory drugs (NSAIDs) were the most commonly used acute treatment (43.8%), while antidepressants were preferred for preventive treatment. Among the participants, 22 individuals experienced persistent headache, with 18 cases linked to the infection, resulting in an average HIT-6 score of 64. The Comirnaty vaccine was administered to the highest proportion of participants (64.6%), and it was also associated with the highest rates of headache occurrence (18 out of 42 patients).

**Conclusion:** Headache related to vaccination was less frequent and persistent compared to headache related to SARS-CoV-2 infection, predominantly affecting female individuals. The first and sixth waves of the pandemic were associated with the highest number of headache cases.

## P125 Global burden of headache disorders and its Trend in 38 OECD countries from 1990-2019: a benchmarking analysis for the global burden of disease study 2019

### L. Mohit^1^, P. Juhi^2^, P. Yashaswi^3^, M. Nazish^4^, D. Arushi^5^, H. Nadia^6^, D. Maulik B.^7^, P. Tirath^8^, A. Vishrant^2^, D. Hardik Dineshbhai^9^

#### ^1^Mamata Medical College, Khammam, India; ^2^G.M.E.R.S Medical College Valsad, Valsad, India; ^3^Government Medical College, Medicine, Surat, India; ^4^Universidad Iberoamericana, Dominican, Medicine, Santo Domingo, Dominican Republic; ^5^Punjab Institute of Medical Sciences, Jalandhar, India; ^6^Dhaka Medical College, Medicine, Dhaka, Bangladesh; ^7^Southwestern University, School of Medicine, Medicine, Cebu, Philippines; ^8^American University of Antigua, Saint John, Antigua And Barbuda; ^9^Gujarat Adani Institute of Medical Sciences, Research, Bhuj, India

##### **Correspondence:** L. Mohit

*The Journal of Headache and Pain 2024,* **25(Suppl 1)**: P125

**Objective:** Headache disorders were the 11th leading cause of disability adjusted life years (DALYs) in Organization for Economic Cooperation and Development (OECD) nations in 2019 accounting for 2.32% of all DALYs.

**Methods:** Using Global Burden of Disease methodology, headache disorders incidence, prevalence and DALYs were analyzed by age, sex, year across 38 OECD nations from 1990-2019 using standardized approach.

**Results:** The prevalence of headache disorders showed an overall increase from 430,969,515 [95% uncertainty interval (UI) 396,833,373-463,712,809] in 1990 to 525,367,526 (485,162,849- 563,243,023) in 2019. Over the period of 1990 to 2019, the annual percentage change (APC) in incidence counts increased by 19%, and DALYs increased by 21%. Among the OECD countries, Italy exhibited the highest APC in age-standardized incidence rate with a 3% increase, followed by Norway with a 2% increase and Switzerland with a 1% increase. In terms of age-standardized DALYs, Norway observed the highest APC with a 12% increase, followed by Belgium with a 9% increase, and Italy and the Netherlands with a 7% increase from 1990 to 2019. Regarding age groups, the highest incidence of headache disorders were observed in the 35-39 years age group in 2019, while the highest burden in terms of DALYs was seen in the 40-44 years age group. The greatest increase in the APC of incidence was observed in the 70-74 years age group by 1%, whereas the APC of DALYs showed the highest increase in the 15-19 years age group by 2% from 1990 to 2019.

**Conclusion:** Headache disorders represent a significant global burden within OECD countries, affecting individuals of all ages. It underscores the urgent need for enhanced research, public awareness, and healthcare strategies to effectively manage and reduce the burden of headache disorders. By prioritizing headache disorders as a public health concern, policymakers and healthcare providers can work together to improve the well-being of individuals affected by these conditions and mitigate their socioeconomic consequences.

**Fig. 1 (Abstract P125):**
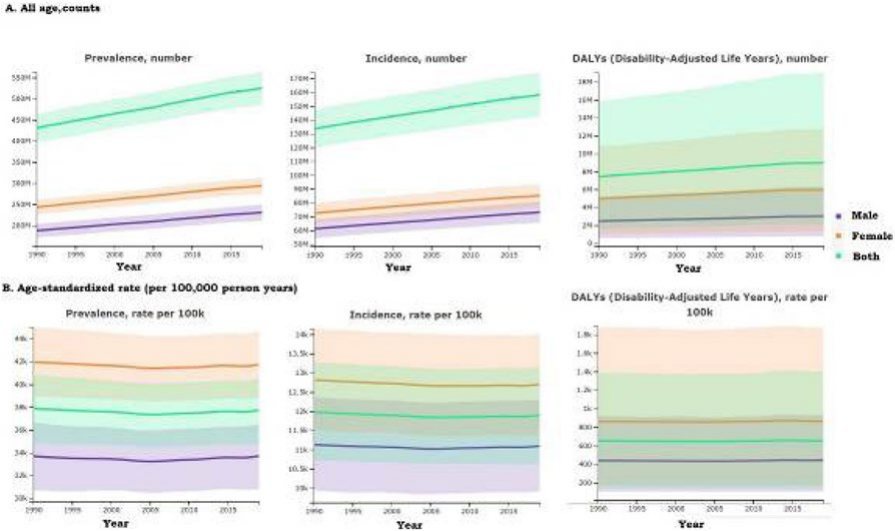
Global Trend of Headache disorders in 38 OECD countries from 1990-2019

**Fig. 2 (Abstract P125):**
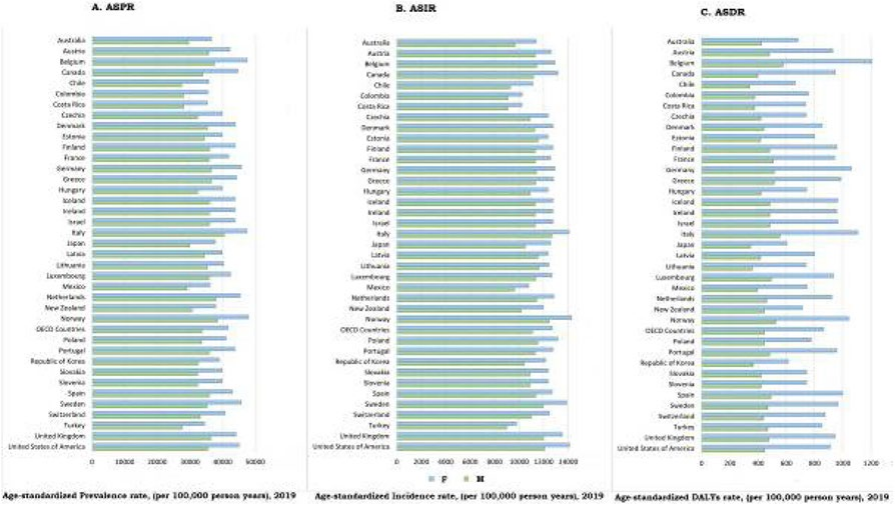
Sex-wise distribution of Headache disorders in 38 OECD Countries, 2019

**Fig. 3 (Abstract P125):**
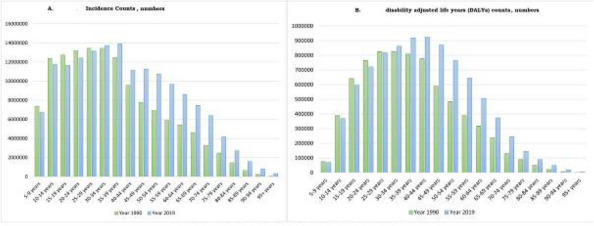
Age-wise distribution of Headache disorders in 38 OECD countries in 1990 and 2019

## P127 Cost and burden of migraine: impact in work productivity and healthcare resource utilization in Spain

### D. García Azorín^1^, C. Moya-Alarcón^2^, B. Armada^2^, M. Sánchez del Río^3^

#### ^1^Hospital Clínico Universitario de Valladolid, Headache Unit, Department of Neurology, Valladolid, Spain; ^2^Pfizer S.L.U., Alcobendas, Spain; ^3^Clínica Universidad de Navarra, Department of Neurology, Madrid, Spain

##### **Correspondence:** D. García Azorín, M. Sánchez del Río

*The Journal of Headache and Pain 2024,* **25(Suppl 1)**: P127

**Objective:** People diagnosed with migraine report greater healthcare resource utilization (HCRU) and lower work productivity than those without migraine. This study compared direct and indirect costs related to migraine between migraine and matched controls.

**Methods:** A cross-sectional study of 7,074 respondents to the 2020 National Health and Wellness Survey (NHWS) captured patient-reported data about Work Productivity and Activity Impairment (WPAI) and HCRU in Spain. Migraine cases were those who reported a physician diagnosis of migraine (*n*=1,020) and ≥1 monthly headache days (MHD) in the past 30 days (*n*= 595). Control group included respondents that reported no history of migraine (*n*=5,490), matched to cases by a propensity score based on 11 demographic and clinical characteristics (*n*=1,190). Costs were obtained from the annual household income in the Statistics National Institute and HCRU from local literature updated to €2023.

**Results:** The mean age of the study population was 41 years, 67% were females and 70% employed. Migraine patients showed statistically significant (*p*<0.001) higher HCRU and worse work productivity among employees, except for the mean number of hospitalization visits (*p*=0.359) and was correlated to the number of MHD. Migraine patients showed higher absenteeism (11.70% vs 7.11%), presenteeism (36.34% vs 21.87%) and work productivity impairment (41.37% vs 25.53%), resulting in an increased annual economic impact of 4,166€ (10,384€ vs 6,218€). Migraine patients had a 40.7% higher HCRU in the past 6 months, including visits of general practitioner, neurologist, emergency room and hospitalizations.

**Conclusion:** Current data still reveals the migraine produces a huge impact in productivity and health care utilization resulting in an additional annual cost for migraine of more than 4,000€ from the patient, payor, and societal perspectives. These results should raise awareness and promote specific health strategies to decrease the burden of migraine in Spain.

**Fig. 1 (Abstract P127):**
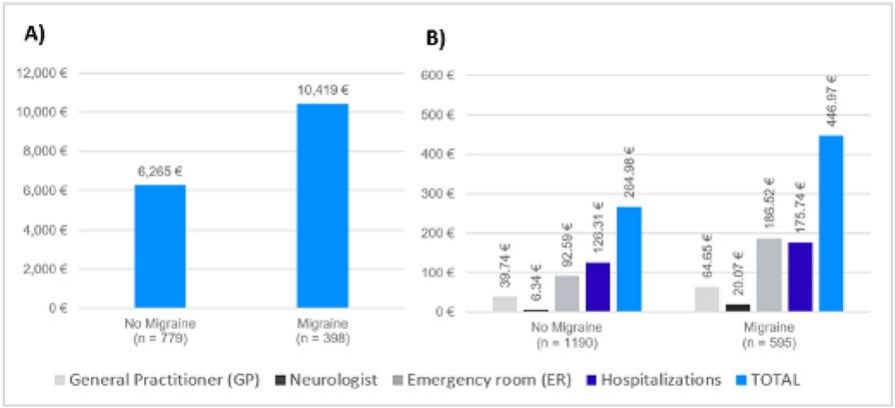
Cost comparison of annual work productivity using the Work Productivity and Activity Impairment (WPAI) in employees (A) and healthcare resource utilization in the past 6 months (B) among people with migraine matched to no migraine controls

## P128 Neuroimaging findings in persistent headache after Covid-19. Related or incidental?

### Á. Sierra-Mencía^1^, A. Peña Aísa^2^, M. Gallego Verdejo^2^, S. Parrado^2^, A. Recío García^1^, Y. González Osorio^1^, M. Sanchez Ronco^2^, B. Arenas García^2^, S. Osorio^2^, D. García Azorín^1^, M. Rodríguez^2^, Á. L. Guerrero Peral^1^

#### ^1^Hospital Clínico Universitario de Valladolid, Headache Unit, Valladolid, Spain; ^2^Hospital Clínico Universitario de Valladolid, Radiology Department, Valladolid, Spain

##### **Correspondence:** Á. Sierra-Mencía

*The Journal of Headache and Pain 2024,* **25(Suppl 1)**: P128

**Objective:** We aim to analyze the neuroimaging findings in a series of patients with persistent headache after COVID-19.

**Methods:** Patients referred throughout 2021 and 2022 to a Headache Unit of a tertiary hospital due to a persistent headache related to COVID-19, defined as present during at least 3 months after COVID-19 infection. We considered clinical and demographic characteristics and a brain MRI study was performed.

**Results:** We included 100 cases in the analysis. In 53 of them, some type of finding was appreciated. Microangiopathy, commonly mild, was the most frequent (34 patients). We also observed anatomical alterations as hippocampal malrotation (2 patients) arachnoid cyst (1 case) or developmental venous anomaly (2 cases). In 3 patients a space-occupying lesions with benign characteristics was found (pineal gland cyst in 2 patients and acoustic neuroma in 1 case). In two patients a previously known pathology was observed (1 case of multiple sclerosis and 1 case of a previous cerebral infarction).

In 2 patients there were radiological data of intracranial hypertension and in 1 a chronic hydrocephalus.

Among radiological findings that could not be ruled out as related to COVID-19, we included 1 patient with cerebral vasculitis classified as antiphospholipid syndrome, 2 with a radiologically isolated syndrome, and 4 with sinusopathy (2 maxillary, 1 frontal and 1 sphenoidal).

**Conclusion:** MRI findings in patients with persistent headache related to Covid19 are fundamentally incidental in our series.

## P130 EndoCEF: Frequency of headache and associated factors in patients with left-sided infective endocarditis

### D. García Azorín^1^, M. de Miguel^2^, J. López^2^, Á. Sierra-Mencía^1^, Á. L. Guerrero Peral^1^, J. F. Arenillas^1^, J. A. San Román^1^

#### ^1^Hospital Clínico Universitario de Valladolid, Neurology, Valladolid, Spain; ^2^Hospital Clínico Universitario de Valladolid, Cardiology, Valladolid, Spain

##### **Correspondence:** D. García Azorín

*The Journal of Headache and Pain 2024,* **25(Suppl 1)**: P130

**Objective:** To determine how frequent headache (HA) is as a symptom of left-sided infective endocarditis (LSIE) and which factors are associated to its presence.

**Methods:** Observational descriptive study with a case-control design, nested in a prospective cohort study. The study was done in three third-level university hospitals from Madrid and Valladolid, Spain. All consecutive patients with LSIE according to the Duke"s classification were included. A series of variables were compared between patients with and without HA, including demographic parameters, comorbidities, endocarditis risk factors, clinical symptoms, and clinical course.

**Results:** 1936 patients with infective endocarditis were screened, among which 1485 were LSIE and 1476 provided valid data. The prevalence of headache was 74/1476 (5.0%). The following statistically significant differences were observed: Patients with HA were younger and had a lower frequency of nosocomial origin, prior cardiopathy in the affected valve, prior history of cardiac surgery, fewer use of genitourinary or intravascular catheters, more chronic kidney disease and had a higher frequency of alcohol abuse. Regarding clinical presentation, patients with HA had lower frequency of cardiac manifestations, cardiac failure, dyspnea, and presented higher frequency of neurological manifestations, altered mental status, nausea, myalgia, meningeal syndrome, cutaneous manifestations, ischemic stroke, hemorrhagic stroke, mycotic aneurisms, and rheumatic manifestations. Concerning the clinical course, patients with HA had higher frequency of post-surgical systemic embolism, CNS embolism, fever, new-onset murmur, cutaneous manifestations, stroke, mycotic aneurisms and intracranial hemorrhage, and lower frequency of new-onset cardiac murmur and cardiac failure.

**Conclusion:** Headache was an uncommon symptom of LSIE, but its presence was associated with a different demographic profile of patients and a clinical presentation and course.

## P131 Headache in leber hereditary optic neuropathy: clinical features and influence factors

### C. Yan^1,2^, H. Zhou^3^, Y. Liu^2^, S. Wu^2^, S. Yu^1,2^

#### ^1^Nankai University, School of Medicine, Tianjin, China; ^2^The Chinese People’s Liberation Army (PLA) General Hospital, Department of Neurology, Beijing, China; ^3^The Chinese People’s Liberation Army (PLA) General Hospital, Department of Ophthalmology, Beijing, China

##### **Correspondence:** C. Yan, Y. Liu

*The Journal of Headache and Pain 2024,* **25(Suppl 1)**: P131

**Objective:** A retrospective study was conducted to analyse headache prevalence, characteristics, burdens and influencing factors in patients with Leber hereditary optic neuropathy (LHON).

**Methods:** This single-centre study recruited genetically confirmed 170 LHON cases between January 2010 and December 2021. A structured scale was applied to collect demographic information and clinical information including headache characteristics (formulated according to ICHD-3) through clinicians' telephone interviews and clinical assessments. At least two neurologists diagnosed headache types and evaluated headache burden. The headache prevalence in patients was compared with headache prevalence data from a door-to-door survey of the general population aged 18-65 years in China.

**Results:** After screening for secondary headaches caused by other factors (flu, hangover, cold, tumour or head injury), the one-year prevalence of headache in patients with LHON was 36.5% (95% CI: 29.6-43.9%), migraine-like headache (MLH) was 16.5% (95% CI: 11.7-22.8%), both significantly higher than the one-year prevalence of primary headache and migraine in the general population (*P*C mutation locus had the highest one-year prevalence of MLH (31.3%). Multiple regression analysis suggested that m.14484T>C compared to m.11778G>A (the most common mutation locus) was an independent risk factor for the occurrence of MLH (*P*=0.024). Analysis of headache burden suggested that patients with the m.14484T>C mutation had higher numerical rating scale (NRS), headache frequency, Headache Impact Test-6 (HIT-6) score, Migraine Disability Assessment (MIDAS) score than patients with m.11778G>A, m.3460G>A and other mutant loci, and the HIT-6 score was significantly higher in patients with m.14484T>C than in patients with other mutant loci (*P*=0.033).

**Conclusion:** The one-year prevalence of MLH in this cohort of LHON patients was higher than that of migraine in the general population; The m.14484T>C mutation locus may be associated with MLH onset and more severe headache burden. This study provided clinical evidence for the addition of a new diagnostic classification for secondary headache in ICHD-3.

## P132 Assessing migraine stigma in Europe: insights and implications for support

### E. Ruiz de la Torre

#### European Migraine and Headache Alliance, Brussels, Belgium

*The Journal of Headache and Pain 2024,* **25(Suppl 1)**: P132

**Objective:** To evaluate the stigma of migraine patients in Europe

**Methods:** The European Migraine & Headache Alliance (EMHA), a patient association umbrella alliance, collaborated with migraine experts to develop an anonymous and voluntary survey. Stigma Scale for Chronic Illnesses (SSCI) scales were used to assess migraine stigma and correlated with frequency, severity, and medication use. The survey was distributed through EMHA"s European network. Data was collected using Microsoft Forms and analyzed in Excel.

**Results:** 4,210 patients completed the survey from around Europe (Spain 22%, France 12%, Italy 11%, Germany 10%, Portugal 8% … ) and were predominantly women aged 25-64y. 90% were migraine sufferers, with 50% considering themselves severe and 57% having ≥ 8 migraine days/month. Disease severity exhibited a negative correlation with employment rates: 80% of part-time/unemployed responders believed their employment status was affected by their condition. Medical and workplace settings were identified as primary sources of stigma. Of responders, 74% felt medical professionals lacked an understanding of what it means to live with migraine, and 79% reported a negative impact on their careers. Respondents reported their perceived overall disease severity to be most influenced by the intensity and treatability of individual attacks. Migraine stigma was seen as more pronounced than for other neurological conditions but less than mental conditions. In terms of language, a disconnect between the scientific meaning of certain terms and how patients understand them has been observed.

**Conclusion:** Migraine stigma impacts personal and professional lives. Broader education on migraine and increased patient advocacy, both of which are underway, are key areas to address. In addition, a change in the lexicon used should also be addressed to help mitigate migraine stigma. These are valuable insights that need to be developed and hopefully continue to help people living with migraine be understood and supported.

## P133 Resistant and refractary migraine in a headache clinic population

### B. Benítez Martínez^1,2^, C. Pérez Prol^2^, C. Espinoza Vinces^2^, F. Abedrabbo^2^, P. Irimia Sieira^2^

#### ^1^Universidad de La Sabana / Hospital Occidente de Kennedy, Neurologia, Chía, Colombia; ^2^Clinica Universidad de Navarra, Pamplona, Spain

##### **Correspondence:** C. Espinoza Vinces

*The Journal of Headache and Pain 2024,* **25(Suppl 1)**: P133

**Objective:** A consensus statement of the European headache federation recently revised the definition of resistant and refractary migraine. We aimed to determine the frequency of resistant and refractary migraine in patients atended in the Headache Unit in a tertiary care center, according to recently proposed criteria.

**Methods:** The study population consisted of a consecutive sample of 340 patients (72.3 % females) with a mean age of 44.5 years (range 13-88) evaluated for the first time in our headache unit over a one-year period (between October 2021 and October 2022). We recorded information on clinical features, previous treatments and final diagnosis.

**Results:** Migraine and tension-type headache were found in 64.1% and 13.5% of patients, respectively. Resistant or refractary migraine was found in 4.4% of patients. From 16 patients with refractory migraine 40.0% (*n*=6) have medication overuse headache (MOH).

**Conclusion:** Resistant and refractory migraine are relatively common conditions between the patients atended for the first time in a headache unit. Therefore, new preventive treatments are needed.

## P134 Pilot study to analyze reasons for changing treatments in patients with chronic migraine

### M. Naprienko, A. Savicheva

#### I.M. Sechenov First Moscow State Medical University (Sechenov University), Department of Sports Medicine and Medical Rehabilitation, Moscow, Russian Federation

##### **Correspondence:** A. Savicheva

*The Journal of Headache and Pain 2024,* **25(Suppl 1)**: P134

**Objective:** Introduction: Headaches are widespread among the population and pose a serious health problem. Among these disorders, chronic migraine (CM) is responsible for the highest rates of disability. Numerous treatments and preventive measures are available for migraines that meet the criteria for chronic migraine (headaches occurring 15 or more days per month, with at least 8 days of migraine). However, not all treatments are well tolerated by patients. The purpose of this study is to analyze the reasons behind treatment changes in patients with chronic migraine.

**Methods:** The study included patients who were initially treated with botulinum toxin type A but switched to another method. To analyze the reasons for the change, a questionnaire was developed, and the following questions were implemented:

Which indicant of headache attack is the most unpleasant?

How well did you tolerate botulinum therapy?

What was the most negative factor for you during the procedure?

The survey involved 24 individuals aged 23 to 58 with a diagnosis of chronic migraine, comprising 18 women (75%) and 6 men (25%).

**Results:** The majority of participants (70%) reported that the intensity of a headache was the most distressing factor during an attack. Only 12% of respondents stated that they tolerated botulinum therapy well. Furthermore, 87% of patients reported experiencing pain during the procedure.

**Conclusion:** The decision to change the method of treatment for chronic migraine patients often stems from poor tolerance due to procedural pain. This study highlights the importance of finding treatment methods that prioritize tolerability

## P135 Global burden of migraine and its trend in G20 countries between 1990-2019: a systematic and comparative benchmarking study

### L. Mohit^1^, P. Juhi^2^, M. Nazish^3^, P. Yashaswi^4^, V. Mihir Paresh^5^, D. Maulik B.^6^, A. Vishrant^2^, D. Arushi^7^, D. Hardik Dineshbhai^8^

#### ^1^Mamata Medical College, Khammam, India; ^2^G.M.E.R.S Medical College Valsad, Valsad, India; ^3^Universidad Iberoamericana, Dominican, Medicine, Santo Domingo, Dominican Republic; ^4^Government Medical College, Medicine, Surat, India; ^5^Matias H. Aznar Memorial College of Medicine, Medicine, Cebu, Philippines; ^6^Southwestern University, School of Medicine, Medicine, Cebu, Philippines; ^7^Punjab Institute of Medical Sciences, Jalandhar, India; ^8^Gujarat Adani Institute of Medical Sciences, Research, Bhuj, India

##### **Correspondence:** L. Mohit

*The Journal of Headache and Pain 2024,* **25(Suppl 1)**: P135

**Objective:** Migraine represents a significant health burden in the Group of Twenty (G20) countries. This study provides an overview of the current understanding of the burden of Migraine and its three decades trend across the G20 countries.

**Methods:** Data from Global Burden of Disease study 2019 were employed to assess the prevalence, incidence and disability adjusted life years (DALYs) of Migraine by Age, sex, year across the G20 nations from 1990-2019 using standardized approach.

**Results:** From 1990 to 2019, the total number of prevalence cases rose from 514,074,311 (95% uncertainty interval (UI) 447,235,113-591,191,324) to 739,883,153 (647,161,170-852,249,295). Concurrently, the burden of the disease, as measured by DALYs, also increased from 19,090,019 (2,881,292-43,181,468) in 1990 to 27,517,881 (4,326,562-61,985,387) in 2019. The Age-Standardized DALYs Rate (ASDR) rose from 513/100,000 (81-1156) in 1990 to 528/100,000 (77-1197) in 2019. Analyzing the annual percentage change (APC) in Age-Standardized Incidence Rate (ASIR) from 1990 to 2019, it is evident that there was an overall increase of 4% in the G20 nations. Among these nations, China exhibited the highest APC of 7%, followed by Brazil with 6% and Italy with 4%. When considering age groups, the highest number of incidence cases was observed among individuals aged 10-14 years, followed by the 15-19 age group. In terms of DALYs, the highest burden was observed in the 30-34 age group in 2019. The APC in the total number of incidences indicated a higher increase among males compared to females (28% vs. 24%).

**Conclusion:** The burden of migraine in the G20 nations is a significant public health concern. Policymakers and healthcare providers should prioritize the development and implementation of effective strategies for migraine prevention, management, and education to alleviate the burden on individuals and societies. Furthermore, international collaboration and knowledge exchange among the G20 nations can facilitate the sharing of best practices and the development of coordinated efforts to address the global burden of migraine effectively.

**Fig. 1 (Abstract P135):**
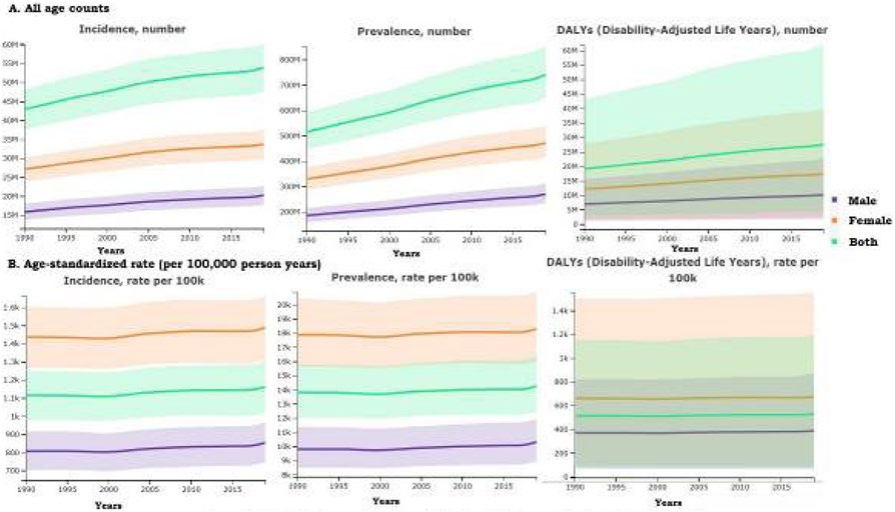
Global burden of migraine and its trend between 1990-2019, G20 Countries

**Fig. 2 (Abstract P135):**
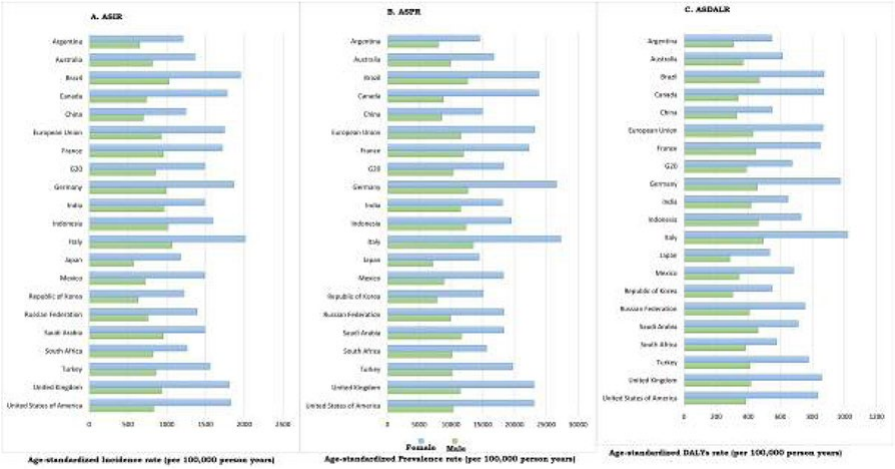
Sex-wise distribution of migraine in G20 Countries, 2019

**Fig. 3 (Abstract P135):**
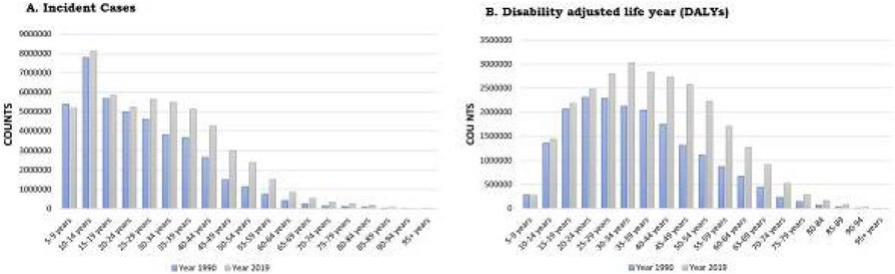
Age-wise distribution of migraine in G20 Countries in 1990 and 2019

## P138 Relationship between migraine attacks per month and the resting-state brain functional network

### V. Gutiérrez-de Pablo^1,2^, Á. L. Guerrero Peral^3^, D. García Azorín^3^, Á. Sierra-Mencía^3^, J. Gomez-Pilar^1,2^, J. Poza^1,2,4^, R. Hornero^1,2,4^, C. Gómez^1,2^

#### ^1^Biomedical Engineering Group, Valladolid, Spain; ^2^CIBER-BBN, Centro de Investigación Biomédica en Red – Bioingeniería, Biomateriales y Nanomedicina, Valladolid, Spain; ^3^Hospital Clínico Universitario de Valladolid, Headache Unit, Valladolid, Spain; ^4^IMUVA, Instituto de Investigación en Matemáticas, Valladolid, Spain

##### **Correspondence:** V. Gutiérrez-de Pablo

*The Journal of Headache and Pain 2024,* **25(Suppl 1)**: P138

**Objective:** Sociodemographic and clinical data, such as the days of headache and days of migraine per month, may be related to alterations in brain activity. The current study aims to evaluate how these variables impact the brain functional network configuration of women with episodic migraine (EM) and chronic migraine (CM).

**Methods:** We have included 78 female subjects: 22 healthy controls (HC) (age 27.0 (25.0, 31.0)), 28 EM patients (age 34.5 (26.0, 39.0)), and 28 CM patients (age 35.5 (27.0, 39.0)). Ten minutes of eyes-closed resting-state electroencephalographic (rsEEG) activity was acquired using a Brain Vision® equipment. Phase lag index (PLI) measure was computed to estimate the brain functional networks in the conventional frequency bands. Then, different graph measures were computed to describe the properties of the brain functional networks: path length (PL), clustering coefficient (ClC), closeness centrality (CC), and graph entropy (GE).

**Results:** Between-group statistically significant differences (*p* < 0.05, Kruskal-Wallis test) were found for all graph parameters in delta and theta bands. Afterwards, correlations between graph parameters and days of migraine per month and days of headache per month were computed for both EM and CM subgroups. Statistically significant Pearson's correlations between all graph parameters in theta band and days of migraine per month were found for CM patients (PL: *r* = -0.4790, *p* < 0.05; ClC: *r* = 0.4274, *p* < 0.05; CC: *r* = 0.5033, *p* < 0.05; GE: *r* = 0.4492, *p* < 0.05), which suggests that CM attacks modify the rsEEG functional network configuration in theta band.

**Conclusion:** Our analyses showed that migraine is associated with alterations in the brain functional network in delta and theta bands. Furthermore, the graph parameters obtained in theta band showed significant correlations with days of migraine per month. These findings could provide new insights into how migraine affects the brain's functional network.

## P139 The evaluation of different types of migraine with cognitive event-related potentials

### M. Waliszewska-Prosół^1^, B. Misiak^2^, M. Straburzyński^3^, S. Budrewicz^1^

#### ^1^Wrocław Medical University, Neurology, Wrocław, Poland; ^2^Wrocław Medical University, Psychiatry, Wrocław, Poland; ^3^University of Warmia and Mazury, Family Medicine and Infectious Diseases, Olsztyn, Poland

##### **Correspondence:** M. Waliszewska-Prosół

*The Journal of Headache and Pain 2024,* **25(Suppl 1)**: P139

**Objective:** Subjective cognitive decline is common in migraine patients. It can be observed not only in all phases of a migraine attack, but also in the interictal phase. We aimed to compare cognitive event-related potentials (CERP) between different types of migraine in the interictal phase.

**Methods:** 55 migraine without aura (MwoA), 40 migraine with aura (MwA), 23 migraine aura without headache (MAwH) patients and 55 control group (CG) were studied. CERP were performed in all groups, including an analysis of N200 and P300 response parameters. The Montreal Cognitive Assessment (MoCA) and the Symbol Digit Modalities Test (SDMT) were performed as a screening test for cognitive performance (including attention and executive functions) of migraine patients. All patients underwent brain magnetic resonance imaging (MRI).

**Results:** There was a significant prolongation of the latencies of N200 and P300 potentials and a significant decrease of P300 amplitude in MwaA patients than in the CG. MwA and MAwH patients showed prolonged latencies of all CERP components and significantly higher P300 wave amplitude compared to the CG. MwA patients showed significantly longer latencies of all CERP components than MwaA and MAwH patients. MAwH patients achieved the highest P300 response amplitudes compared out of all migraine patients. Neuropsychological tests and brain MRI showed no abnormalities.

**Conclusion:** Our observations confirm the existence of brain bioelectrical activity disturbances in migraine patients in the interictal phase in extensive neuronal networks involved in cognitive processes. The increased amplitude of CERP in MwA and MAwH patients may indicate increased cerebral cortex activity.

## P140 P2X7R/NLRP3 signaling pathway-mediated pyroptosis and neuroinflammation contributed to cognitive impairment in a mouse model of migraine

### Z. Xiao

#### Renmin Hospital of Wuhan University, Department of Neurology, Wuhan, China

*The Journal of Headache and Pain 2024,* **25(Suppl 1)**: P140

**Objective:** Migraine is the second most common form of headache disorder and the second leading cause of disability worldwide. Cognitive symptoms ranked second resulting in migraine-related disability, after pain. P2X7 receptor (P2X7R) was recently shown to be involved in hyperalgesia in migraine. However, the role of P2X7R in migraine-related cognitive impairment is still ill-defined. The aim of this study was to explore the molecular mechanisms underlying migraine-related cognitive impairment and the role of P2X7R in it.

**Methods:** Here we used a well-established mouse model of migraine that triggered migraine attacks by application of inflammatory soup (IS) to the dura. We evaluated the effects of repeated dural IS stimulation on NLRP3 inflammasome signaling pathway, cognition-related pathological changes (gliosis, neuronal loss and white matter damage) and cognitive behavior. We determined whether these possible pathological changes and cognitive decline were mediated by P2X7R using a specific P2X7R antagonist, Brilliant Blue G (BBG).

**Results:** Our results showed that repeated dural IS stimulation triggered upregulation of P2X7R, activation of NLRP3 inflammasome, release of proinflammatory cytokines (IL-1β and IL-18) and activation of pyroptotic cell death pathway. Gliosis (microgliosis and astrogliosis), neuronal loss and cognitive impairment also occurred in the IS-induced migraine model. No significant apoptosis or whiter matter damage was observed following IS-induced migraine attacks. These pathological changes occurred mainly in the cerebral cortex and to a less extent in the hippocampus, all of which can be prevented by pretreatment with a specific P2X7R antagonist Brilliant Blue G (BBG). Moreover, BBG can alleviate cognitive impairment following dural IS stimulation.

**Conclusion:** These results identified P2X7R as a key contributor to migraine-related cognitive impairment and may represent a potential therapeutic target for mitigating cognitive impairment in migraine.

**Fig. 1 (Abstract P140):**
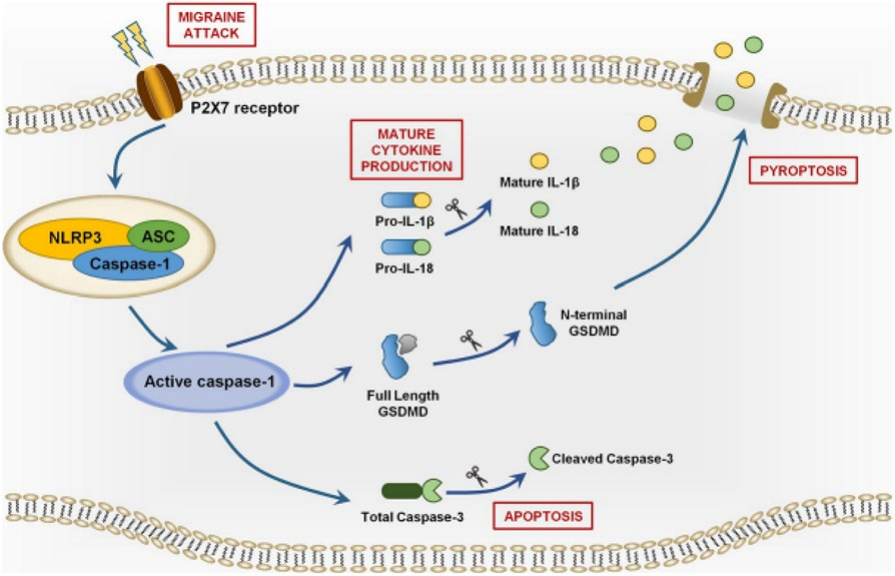
See text for description

## P142 Investigation of the association between genetic variability in oxidative stress related factors and migraine

### M. Papasavva^1,2^, M. Vikelis^3^, V. Siokas^4^, M. S. Katsarou^2^, E. Dermitzakis^5^, A. Raptis^2^, A. Kalliantasi^2^, E. Dardiotis^4^, N. Drakoulis^2^

#### ^1^European University Cyprus, Nicosia, Cyprus; ^2^National and Kapodistrian University of Athens, Pharmacy, Athens, Greece; ^3^Headache Clinic, Mediterraneo Hospital, Glyfada, Greece; ^4^University of Thessaly, Neurology, Larisa, Greece; ^5^Euromedica General Clinic, Thessaloniki, Greece

##### **Correspondence:** E. Dermitzakis

*The Journal of Headache and Pain 2024,* **25(Suppl 1)**: P142

**Objective:** Migraine is a common neurovascular disorder with significant environmental and genetic components. Emerging evidence suggests disturbed redox balance in migraine patients. Genetic variability might affect an individual"s oxidative/antioxidant capacity. Underpinning the association of single nucleotide polymorphisms (SNPs) in oxidative stress-related genes with migraine can eventually contribute to more efficacious disease management. The aim of the current study was to investigate the association of seven SNPs in genes encoding for oxidative stress-related factors, i.e., rs4880 (*SOD2*), rs1001179 (*CAT*), rs1050450 (*GPX1*), rs1695 (*GSTP1*), rs1138272 (*GSTP1*), rs1799983 (*NOS3*), and rs660339 (*UCP2*), with migraine susceptibility and diverse clinical phenotypes in a case-control population residing in Greece.

**Methods:** DNA samples were collected and extracted from 221 migraine patients and 265 control subjects with no history of any headache disorder. The genotyping for the SNPs under investigation was carried out by real-time polymerase chain reaction followed by melting curves analysis [LightCycler® 480 (Roche Ltd., Switzerland)].

**Results:** A statistically significant association (*p*<0.05) was observed (i) for the *NOS3* rs1799983 with migraine susceptibility in the male population of the study, (ii) for the *GPX1* rs1050450 with the typical duration of migraine attacks and (iii) for the *CAT* rs1001179 with the disease age of onset. In addition, evidence for an association was provided for the SNPs in the *CAT*, *GSTP1* and *UCP2* genes with sleep/weather changes, alcohol consumption and physical exercise, respectively, as migraine attacks triggers.

**Conclusion:** The current study provided evidence for a possible association of genetic polymorphisms in oxidative stress-related factors with migraine susceptibility and diverse clinical phenotypes, further reinforcing the emerging role of oxidative stress in the pathophysiology of migraine.

## P143 Primary stabbing headache vs. secondary to venous angioma: a case report

### M. D. Calabria Gallego

#### Hospital Universitario de Salamanca, Neurology, Salamanca, Spain

*The Journal of Headache and Pain 2024,* **25(Suppl 1)**: P143

**Objective:** To present a case report

**Methods:** A 35-year-old patient complained of occasional headache localized in the left frontal region, described as jabs, with an intensity of 8/10. The patient did not experience photophobia, phonophobia, nausea or vomiting and did not have a time predominance in the pain. However, his mother had a history of migraine. On physical examination, the major suboccipital and supraorbital nerves were not painful to pressure, and the fundus was normal.

Amitriptyline 10 mg was prescribed once a day at night, resulting in a notable improvement in the headache. A cranial magnetic resonance imaging (MRI) was scheduled to evaluate any possible underlying cause.

**Results:** The MRI (Figure 1) revealed findings suggestive of venous angioma in the inferior portion of the left cerebellar hemisphere with drainage veins towards the left ponto-cerebellar angle and probably towards the inferior petrosal sinus.

The patient was evaluated by neurosurgery, which considered the finding incidental and did not require surgical intervention. The patient was kept under follow-up with regular controls of his headache, and amitriptyline was maintained as treatment.

**Conclusion:** Primary stabbing headache is a rare form of headache that can be difficult to diagnose. Careful evaluation of the patient, including neurological examination and MRI, is necessary to evaluate any possible underlying cause. In this case, amitriptyline was an effective treatment for the patient's primary stabbing headache, and evaluation by neurosurgery ruled out any surgical intervention in the venous angioma.

It is important to emphasize that careful and thorough evaluation of a patient with headaches, including the use of neuroimaging when necessary, is essential to rule out possible serious diseases that could endanger the patient's life. However, once relevant findings are obtained, it is not always essential to strictly determine whether the headache is primary or secondary, especially if the detected entity does not pose a risk to the patient, such as in the case of a venous angioma without risk like the one in our case.

*Disclosure statement:* Informed consent to publish this case study and its potentially identifiable information of the patient was obtained from the individual involved. The patient gave explicit permission for the publication of this case report, including any relevant clinical details.

**Fig. 1 (Abstract P143):**
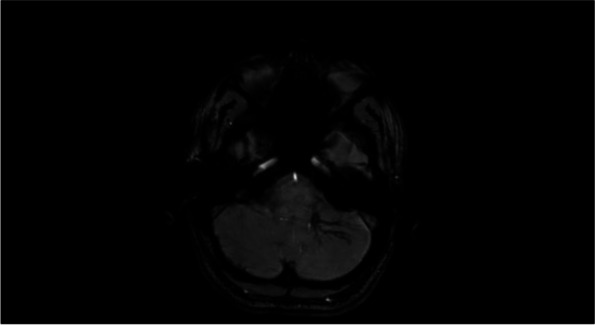
See text for description

## P145 Reduction in headache days with treatment is more important for migraine patients than if the treatment is administered subcutaneously or orally: results from a patient preference survey on calcitonin gene-related peptide (CGRP) antagonists

### C. Whichello^1^, L. Viktrup^2^, O. J. Varnado^2^, M. Quaife^1^, M. Trapali^1^, A. Tockhorn-Heidenreich^2^, R. Okonkwo^2^

#### ^1^Evidera Inc., London, United Kingdom; ^2^Eli Lilly and Company, Indianapolis, IN, United States

##### **Correspondence:** R. Okonkwo

*The Journal of Headache and Pain 2024,* **25(Suppl 1)**: P145

**Objective:** To present a discrete choice experiment (DCE) that assessed the preferences of people with episodic migraine (EM) for the attributes of self-injectable calcitonin gene-related peptide (CGRP) monoclonal antibodies (mAbs) and an oral gepant.

**Methods:** Pts aged ≥18 years with EM were recruited to an on-line DCE survey in the US and were asked to choose their preferred hypothetical treatment with 5 attributes: administration, chance of ≥50% reduction in monthly migraine headache days, time-to-onset, impact of migraine on daily activities, and reduction in the number of days with acute medication use. The relative attribute importance (RAI) scores indicating the importance of an attribute vs the remaining attributes were calculated using a mixed logit model.

**Results:** The most important attribute was a ≥50% reduction in monthly migraine headache days, RAI score 36.3% (95% CI: 32.1%; 40.5%) , followed by the impact on daily activities (RAI: 24.4%; 95% CI: 20.0%; 28.8%), treatment onset (RAI: 19.5%; 95% CI: 16.3%; 22.6%), reduction in acute medication (RAI: 15.2%; 95% CI: 11.9%; 18.5%). Treatment administration was the least important driver of patient preferences (RAI: 4.6%; 95% CI: 0.7%; 8.5%).

These results indicate that reducing the chance of having a migraine attack was 7.9 times more important than the way of administration (36.3% / 4.6% = 7.9), treatment onset (19.5%) or reduction in acute medication use (15.2%). Pts also indicated that reducing the impact of migraine on daily activities (24.4%) was more important than time to onset (19.5%) and treatment administration (4.6%). Pts who were "not at all" or "a little" reluctant to self-inject had a significantly higher RAI for treatment administration (27% vs. 5%, *p*<0.001) compared with the overall sample.

**Conclusion:** Pts with EM value achieving ≥50% reduction in monthly migraine headache days as most important, and the method of administration as the least important attribute when choosing between potential treatments.

Previously presented at American Headache Society - 65th Annual Scientific Meeting 2023.

## P146 Trends in the use of neuroimaging in non-acute pediatric headache: Does the experience of the cephalologist really matter?

### G. Sforza^1^, L. Papetti^1^, G. Deli^2^, S. Barni^3^, F. Ursitti^1^, G. Monte^1^, S. Tarantino^1^, M. Proietti Checchi^1^, M. Valeriani^1^

#### ^1^Bambino Gesù Children's Hospital, Developmental Neurology Unit, Rome, Italy; ^2^Hospital of Rome, Tor Vergata University, Child Neurology and Psychiatric Unit, Rome, Italy; ^3^Bambino Gesù Children's Hospital, University Hospital Pediatric Department, Rome, Italy

##### **Correspondence:** G. Sforza

*The Journal of Headache and Pain 2024,* **25(Suppl 1)**: P146

**Objective:** To identify trends in rates of use diagnostic neuroimaging in non-acute pediatric headache and correlate them with the experience of neurologists at the headache center.

**Methods:** Retrospective analysis of neuroimaging rates was conducted on 135 children and adolescents aged 2 to 18 years with headache on their first visit to pediatric headache center. Among the parameters of current practice were evaluated: age < 6 years, presence of neurological signs, nocturnal awakenings, occipital pain, pattern charge, sudden or abrupt onset , presence of typical or atypical aura, positional headache or precipitated by sneezing, coughing, or exercise; vertigo, failure to respond to analgesics.

Also we analyzed the rate of neuroimaging prescription in relation to the experience of neurologists.

**Results:** The neuroimaging rate is inversely proportional to the years of experience at the headache center. The most criteria for neuroimaging are in accordance with the red flags. (Data in progress).

**Conclusion:** In the evaluation of pediatric patients with non-acute headache, neuroimaging rates are related, not only to the presence of red flags, but also to the experience of the cephalologist.

## P147 Chronic whiplash-associated headache is mainly associated with neck pain intensity, neck disability and kinesiophobia: results from a network analysis

### E. Anarte-Lazo^***1***,2^, B. Liew^3^, V. Devecchi^2^, C. Bernal-Utrera^1^, C. Rodriguez-Blanco^1^, D. Falla^2^

#### ^1^University of Seville, Physiotherapy Department, Sevilla, Spain; ^2^University of Birmingham, Centre of Precision Rehabilitation for Spinal Pain, Birmingham, United Kingdom; ^3^University of Essex, School of Sport, Rehabilitation and Exercise Sciences, Essex, United Kingdom

##### **Correspondence:** E. Anarte-Lazo

*The Journal of Headache and Pain 2024,* **25(Suppl 1)**: P147

**Objective:** To evaluate which factors can influence post-traumatic headache six months after a whiplash injury

**Methods:** Patients between 18 and 65 years old suffering acute whiplash-associated disorders (WAD) grade II were recruited. A network analysis was performed using data from a prospective study. The range of motion (ROM), neck endurance, cranio-cervical flexion test, pressure pain thresholds (PPT) over the median, radial, ulnar, greater occipital nerve, and supra-orbital nerves, headache and neck pain intensity, neck disability index, and kinesiophobia were evaluated in the acute phase. Patient-reported outcomes were also evaluated six months later. Headache intensity was measured using a visual analogue scale from 0 to 100. ROM was measured with a digital goniometer, and PPT (kg/cm2) was measured with a digital algometer.

**Results:** 47 participants were initially recruited, and 45 were included in our study. The sample had a mean age of 38.94 years (SD: 10.99). Among all the variables included, the edge between neck pain and headache intensity six months after the whiplash injury had the second-highest value (0.50 [95% CI 0.34 to 0.61]). Moreover, a significant correlation was observed between neck pain and headache intensity, neck disability, and kinesiophobia at six months after the injury. Among the baseline measures, kinesiophobia and PPT were the variables with the highest correlation with headache at six months.

**Conclusion:** A network analysis showed that neck pain and headache intensity had the strongest association, and a significant interaction was observed between these two variables, as well as neck disability and kinesiophobia measured six months after the whiplash injury. These results suggest that, among the physical factors measured at baseline, only PPT over neural structures may have a correlation with the intensity of chronic whiplash-associated headache.

**Fig. 1 (Abstract P147):**
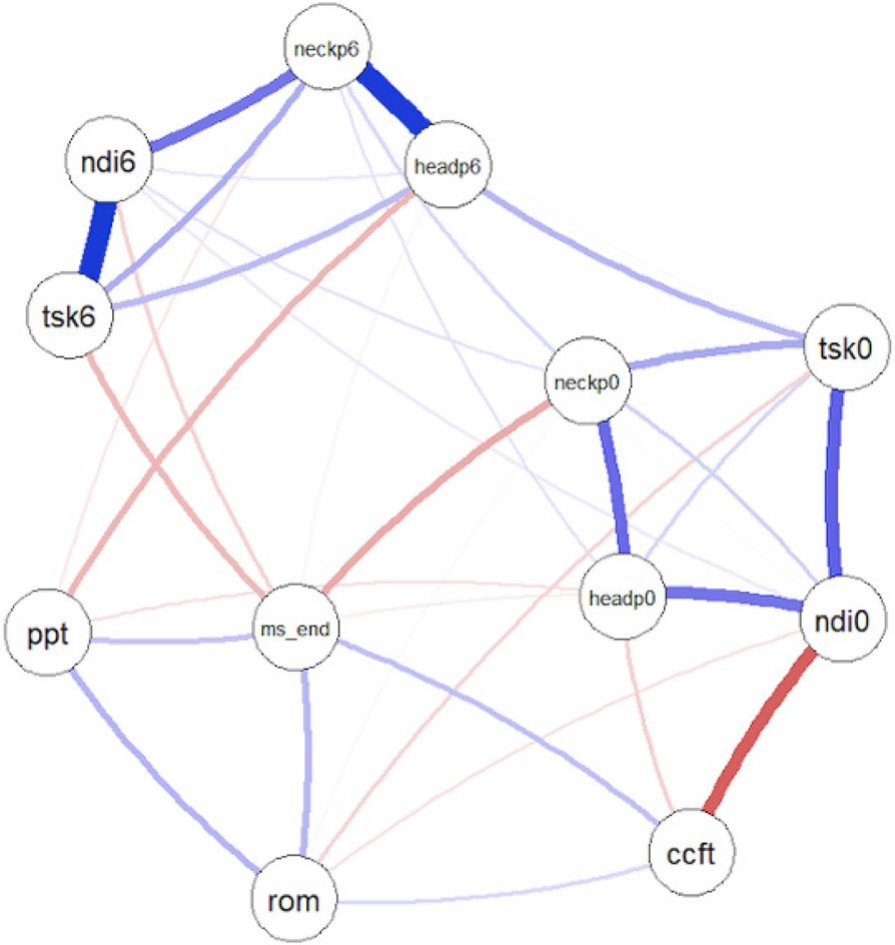
See text for description

## P148 The presence of neuropathic pain features is associated with the presence of headache in people with acute whiplash-associated disorders: a case-control study

### E. Anarte-Lazo^1,2^, M. Barbero^3^, D. Falla^2^, C. Rodriguez-Blanco^1^, C. Bernal-Utrera^1^

#### ^1^University of Seville, Physiotherapy Department, Sevilla, SpainL ^2^University of Birmingham, Centre of Precision Rehabilitation for Spinal Pain, Birmingham, United Kingdom; ^3^University of Applied Sciences and Arts of Southern Switzerland, 2rLab Rehabilitation Research Laboratory, Manno, Switzerland

##### **Correspondence:** E. Anarte-Lazo

*The Journal of Headache and Pain 2024,* **25(Suppl 1)**: P148

**Objective:** To evaluate whether the presence of neuropathic pain features is related to the presence of acute whiplash-associated headache

**Methods:** A case-control study was conducted between July 2022 and June 2023, including patients between 18 and 65 years old diagnosed with suffering from whiplash-associated disorders (WAD) grade II, according to the Quebec Task Force. We excluded patients who had a previous headache, were evaluated more than 30 days after the whiplash injury, and/or had a serious disease or congenital disturbance. A yes/no question was used to evaluate the presence of headache that appeared within seven days after the accident, and the S-LANSS questionnaire was used to assess neuropathic pain features: a score ≥ 12 indicates pain with neuropathic features. Before the evaluation, they were advised that the results from this assessment would not be considered as part of any insurance claim. Ethical approval was granted by the applicable institutional human research ethics committee from the University of Seville, Spain.

**Results:** Out of 83 participants recruited, 74 participants were included in our study, including 39 women. The mean age was 40.67 (SD: 10.81). Headache was present in 41 participants (55.41%). The mean value for the S-LANSS questionnaire was 10.01 (SD: 4.78), and it was positive in 23 participants with headache (56.10%) and 5 without headache (15.05%). A chi-squared test revealed significant differences between the groups (χ2 = 13.033; *p* < 0.001).

**Conclusion:** The presence of neuropathic pain features was higher in patients who presented with headache soon after a whiplash injury compared to those who did not develop a headache. These findings suggest that neuropathic pain features may be involved in the presence of acute whiplash-associated headache.

## P149 Refractory facial pain and the importance of the multi professional approach

### G. Nimer, J. de Souza, R. Neto

#### Hospital Universitário Antônio Pedro, Neurology/Headache, Niteroi, Brazil

##### **Correspondence:** G. Nimer

*The Journal of Headache and Pain 2024,* **25(Suppl 1)**: P149

**Objective:** To demonstrate the beneficial impact of an engaged interdisciplinary Headache team on the comprehensive approach and quality of life of a patient.

**Methods:** The patient provided authorization for the case report in accordance with ethical standards.

**Results:** In 2017, a 48-year-old male presented with pain in the nasal area, which progressed to neuropathic pain and trigemino-autonomic signs in the V2 and V3 territories on the right side of the face. Previous treatment included Gabapentin 900 mg, Duloxetine 60 mg, and Carbamazepine 1000 mg per day, with partial improvement observed with Carbamazepine. Currently, the patient is taking Pregabalin 300 mg and Amitriptyline 100 mg per day. Panoramic mouth X-rays, brain and temporomandibular joint (TMJ) MRI, and EMG showed no significant changes, except for a small retention cyst in the alveolar recess observed on a brain CT scan in 2017 and sinus MRI in 2019. After discussions with the Headache team, a neurologist performed a right sphenopalatine block, which did not provide immediate improvement. Later, an infraorbital block was suggested, with the approach (intraoral or transdermal) yet to be determined. Six months later, during a follow-up appointment, an intraoral infraorbital blockade using lidocaine was performed by a dentist, resulting in partial improvement immediately after the procedure. This case highlights the importance of a multidisciplinary team in managing complex patients. The primary objectives of multidisciplinary treatment programs are to provide patients with better information and education on managing headaches and to enhance therapy to reduce headache frequency and improve overall quality of life.

**Conclusion:** Literature demonstrates the valuable role of a multidisciplinary Headache team in the management of refractory facial pain.

*Disclosure statement:* Informed consent to publish this case study and its potentially identifiable information of the patient was obtained from the individual involved. The patient gave explicit permission for the publication of this case report, including any relevant clinical details.

## P150 Social impact of migraine and visibility in social networks

### M. Alvarez^1^, R. Alvarez^2^, E. Fernandez^3^, M. T. Temprano Fernández^1^, L. González-Fernández^1^, B. Venegas Pérez^2^, N. Riesco Pérez^2^

#### ^1^Hospital Universitario de Cabueñes, Neurology, Gijón, Spain; ^2^Hospital Central de Asturias, Neurology, Oviedo, Spain; ^3^Hospital Universitario de San Agustin , Neurology, Avilés, Spain

##### **Correspondence:** M. Alvarez

*The Journal of Headache and Pain 2024,* **25(Suppl 1)**: P150

**Objective:** To determine what perception migraine patients have about society's understanding of their disease and their visibility on social networks

**Methods:** Face-to-face surveys were carried out on patients with migraine from the Neurology clinics of the three main hospitals in Asturias (Hospital Universitario de Cabueñes, Hospital Central de Asturias, Hospital Universitario de San Agustín), between 01/10/2023 and 04/10/2023.

**Results:** 804 surveys were carried out, mostly to women (88%) with a mean age of 46 years. 72% of patients believe that migraine is not a socially recognized disease and 39% believe that it is viewed negatively. 37% have ever felt embarrassed for having a migraine and 33% have hidden it on some occasion, mainly at work (71%). These percentages were higher in patients with a greater number of headache days, those who were of working age, and in women. Most of the respondents do not know any public person with migraine, patient association or digital platform related to it. 83% use social networks —mainly Instagram and Facebook— and up to 63% do so as a means of information. 90% of patients perceive that migraine does not have visibility on social networks and 97% would like it to have more representation.

**Conclusion:** Patients with migraine feel that their disease is not understood by those around them and demand more information, disclosure and visibility on social networks.

## P151 Exploratory analysis of headache characteristics, lifestyle, and mental health in chronic headache: a mixed-methods study

### I. Potapov^1^, D. Ovchinnikov^1^, Y. Didenko^2^, A. Novikova^1^

#### ^1^Almazov National Medical Research Centre, St. Petersburg, Russian Federation; ^2^"Seven Doctors" Medical Clinic, St. Petersburg, Russian Federation

##### **Correspondence:** I. Potapov

*The Journal of Headache and Pain 2024,* **25(Suppl 1)**: P151

**Objective:** This study aimed to explore the interconnections among three major groups of factors in patients with primary chronic headache: (1) headache-related disability and characteristics of the headache, (2) lifestyle determinants, and (3) mental health comorbidities.

**Methods:** This study employed a mixed-methods approach. Qualitative data were collected through patient interviews, and quantitative data through a questionnaire. Participants were aged 18-65 with a diagnosis of chronic or high-frequency episodic migraine/tension-type headache. Interview results were independently evaluated by two researchers, with a focus on patient self-reported links between factors of interest. The research team designed a questionnaire with 134 questions. These covered clinical headache characteristics, prodromal symptoms, triggers, epiphenomena, anamnesis, lifestyle, and demographic data, supplemented by the HADS anxiety scale and Beck"s depression inventory. Quantitative data were analyzed using Python, with the Kruskal-Wallis test employed to assess statistical validity.

**Results:** The study involved 81 questionnaire respondents and 15 in-depth interviews. Patients primarily highlighted emotional disstress from ineffective prophylactic treatment and frustration from adverse effects. Most (*n*=11) linked increased headache frequency/severity to irregular/long work hours, sedentary lifestyle, and poor sleep. Quantitative analysis revealed the correlation between disability score and frequency of autonomic symptoms; higher disability scores in patients with inconsistent, rather than consistently unhealthy sleep and work habits, and in patients with remote jobs.

**Conclusion:** Chronic headache patients place significant emphasis on their lifestyle and mental health. However, certain non-apparent correlations have been identified that challenge conventional wisdom regarding sleep hygiene and other factors. Further research is warranted to determine lifestyle recommendations that could yield more beneficial prognostic outcomes

## P152 Exploring the civid experience of chronic migraine: insights from a phenomenological study

### S. Ulutas^1^, D. Uluduz^2^, A. Özge^3^

#### ^1^Bahcesehir University Medical Faculty, Neurology, İstanbul, Turkey; ^2^Cerrahpasa University Medical Faculty, Neurology, İstanbul, Turkey; ^3^Mersin University Medical Faculty, Neurology, Mersin, Turkey

##### **Correspondence:** S. Ulutas

*The Journal of Headache and Pain 2024,* **25(Suppl 1)**: P152

**Objective:** Migraine affects 1.04 billion people worldwide, comprising around 14.4% of the global population. It significantly impacts quality of life, resulting in missed work or school, social isolation, and decreased productivity.

Understanding of Chronic Migraines (CM) remains limited. To address this, a phenomenological study explored the experiences of 12 CM sufferers.

**Methods:** Phenomenological research investigates the essence of a phenomenon. Through in-depth interviews lasting 45-60 minutes in Turkish, data was collected and translated. The translation of the participants" articulation of their feelings and expressions were carefully interpreted in English to both keep the essence of their remarks and linguistically ensure the comprehension. Interviews were semi-structured, guided by questions and probing participant responses.

The transcribed interviews were analyzed using a phenomenological approach, identifying recurring themes. Participants were diverse in age, gender, socioeconomic status, and other factors.

**Results:** Findings revealed physical and emotional symptoms. Diagnosis of childhood-onset migraines occurred later, requiring multiple neurologist consultations. Patients experienced 10-15 monthly mild to moderate migraine attacks. Severe attacks led to emergency room visits. Desperation was a common description of the migraine experience. Emotional impact resulted in frustration, anxiety, and social isolation. Participants struggled with engaging in social activities, leading to loneliness. Treatment brought mixed emotions, including alienation and anxiety from medication obligations.

Coping strategies included medication, relaxation techniques, and lifestyle adjustments.

**Conclusion:** This study provides valuable insights into the complex nature of chronic migraines. Greater support and understanding are necessary, along with improved interventions. These findings can inform clinical practice and enhance the quality of life for those living with migraines.

## P153 Coping strategies to stressful events in adolescents with headache

### M. Proietti Checchi^1^, S. Tarantino^1^, L. Papetti^1^, F. Ursitti^1^, G. Monte^1^, R. Moavero^1,2^, G. Sforza^1^, M. A. N. Ferilli^1^, A. Voci^1^, M. Valeriani^1,3^

#### ^1^Developmental Neurology, Bambino Gesù Children Hospital, IRCCS, Rome, Italy; ^2^Tor Vergata University of Rome, Child Neurology and Psychiatric Unit, Rome, Italy; ^3^Aalborg University, Center for Sensory-Motor Interaction, Denmark Neurology Unit, Aalborg, Denmark

##### **Correspondence:** M. Proietti Checchi

*The Journal of Headache and Pain 2024,* **25(Suppl 1)**: P153

**Objective:** We aimed to explore: 1) coping responses to stressful events and their possible association with migraine severity (frequency of attacks and pain intensity) and the use of prophylactic treatments in adolescents with migraine; 2) the association between coping strategies, anxiety and depression levels.

**Methods:** We included 81 adolescents (m.a. 13.8±1.6 years; 18 M and 63 F). They were divided into: (1) high frequency (weekly to daily episodes) and low frequency (≤4 episodes per month); (2) mild and severe pain; (3) need for prophylactic treatment or not. To evaluate patients" anxiety, depression and coping strategies we used respectively SAFA-A, SAFA-D and CRI-Y questionnaires.

**Results:** In our sample, high frequency of attacks was associated with "Logical Analysis" (*p*=0.012) and "Positive Reappraisal" (*p*=0.002) strategies of coping. Patients with severe intensity of pain showed levels above the normal range in "Problem Solving" (*p*=0.050) and "Cognitive Avoidance" (*p*=0.034) subscales. No significant association was found between the use of a prophylactic treatment and coping responses. We found higher symptoms of "Total anxiety" (*p*=0.025), "School anxiety" (*p*=0.024) and "Feeling of hopeless" (*p*=0.029) in patients with the tendency to use a "Positive Reappraisal" strategy of coping; on the other hand, higher symptoms of depression were associated with "Cognitive Avoidance" (*p*=0.033) style.

**Conclusion:** Adolescents with migraine tend to use a coping style characterized by an approach to the problem. In particular, cognitive coping strategies may be more prevalent in high frequency patients, while behavioral coping strategies could be more commonly used in severe intensity patients.

## P154 Migraine frequency, disordered eating attitudes and psychological symptoms in adolescents: Which relationship?

### S. Tarantino^1^, M. Proietti Checchi^1^, L. Papetti^1^, F. Ursitti^1^, G. Monte^1^, G. Sforza^1^, M. Valeriani^1,2^

#### ^1^Bambino Gesù Children's Hospital, Developmental Neurology Unit, Rome, Italy; ^2^Aalborg University, Center for Sensory-Motor Interaction, Denmark Neurology Unit, Aalborg, Denmark

##### **Correspondence:** S. Tarantino

*The Journal of Headache and Pain 2024,* **25(Suppl 1)**: P154

**Objective:** Data on disordered eating attitudes in pediatric migraine are, so far, sparse. We aimed to investigate: 1) the prevalence of disordered eating behaviors and their association with the severity of migraine and body weigh; 2) the possible mediating role of anxiety and/or depression in the association between disordered eating attitude and frequency of migraine attacks in children.

**Methods:** We included 103 adolescent girls with migraine (mean age 14.2±1.6 years). The frequency of migraine was divided in: 1) high frequency (from weekly to daily episodes) and 2) low frequency (≤4 episodes per month). According to their Body Mass Index, patients were divided in "underweight" (<5th percentile), "normal weight" (from ≥5 to <85 percentile), "overweight" (from ≥85 to <95) and "obese" (≥95). Given the low number of obese patients, overweight and obese groups were considered together in the "Overweight" group. Due to their low frequencies, "underweight" patients were not included in our analysis. The Italian SAFA battery was used to investigate eating disorder risk (SAFA-P), anxiety (SAFA-A) and depression (SAFA-D).

**Results:** In our sample, 20.4% of patients had scores above the normal range in SAFA-P Total scale. We found bulimic and anorexic attitudes respectively in 17.5% and 22.3% of patients. Perfectionism was high in 46.6% of patients. We found significant higher bulimic symptoms in patients with high frequency of attacks (*p*=0.040). Overweight patients showed higher levels of disordered eating attitudes as compared with normal weight patients (SAFA-P Tot: *p*=0.011). We found a mediating role between bulimic and anorexic attitudes and high frequency for school anxiety (respectively, *p*=0.040 and *p*=0.045).

**Conclusion:** Our data suggest an association between bulimic attitudes and the frequency of migraine. We suppose that, in our sample, school anxiety may lead to disordered eating attitudes which may influence the frequency of migraine.

## P156 Do neuropsychiatric comorbidities influence the outcome of Greater Occipital Nerve Injections for headache prevention in paediatric patients? Preliminary results from a UK experience

### I. Frattale, F. Puledda, P. Prabhakar

#### Tor Vergata University of Rome, Department of Neurosciences, Rome, Italy

##### **Correspondence:** I. Frattale

*The Journal of Headache and Pain 2024,* **25(Suppl 1)**: P156

**Objective:** Primary headaches are the most common cause of pain in paediatric age. Greater occipital nerve injections (GONI) are an effective, safe and well tolerated treatment, which can be used for primary headache prevention both in adults and in children. We aim to evaluate if the presence of neuropsychiatric comorbidities influences GONI response.

**Methods:** We retrospectively analysed the clinical history and therapeutic response of all paediatric patients with a primary headache diagnosis who underwent GONIs from June to December 2022 at the Headache Centre of Great Ormond Street Hospital in London, UK. We analysed demographic characteristics, headache diagnosis, psychiatric diagnosis, considering separately mood disorders and neurodevelopment comorbidities (autism, ADHD, learning disabilities). Our primary outcome was response to treatment, considered as a >50% reduction in monthly headache days following twelve weeks from the GONI.

**Results:** 85 patients were included, 80% were female. Mean age at first injection was 14 years ± 1,6. The most frequent headache diagnosis was migraine without aura (*n*=44), followed by migraine with aura (*n*=25), NDPH (*n*=8), TTH (*n*=1) and cluster headache (*n*=1). The number of past GONIs administrations was 5 ± 2.5for each patient. Forty patients had previously received a neuropsychiatric diagnosis, *n*=25 had mood disorders and *n*=15 neurodevelopment comorbidities, of which the most frequent were learning disabilities (47%), ADHD (40%), autism (20%) and eating disorders (20%). For our primary outcome, *n*=57 (67%) patients showed >50% reduction in monthly headache days.

There was no significant difference in GONI response in patients with mood comorbidity respect to those without (χ2=1.007). We also found no difference in response in patients with neurodevelopment comorbidity (χ2=0.884).

**Conclusion:** In our sample the presence of psychiatric comorbidities did not influence the effect of GONI for primary headache prevention. GONIs are helpful in adolescents with and without neuropsychiatric comorbidities.

**Fig. 1 (Abstract P156):**
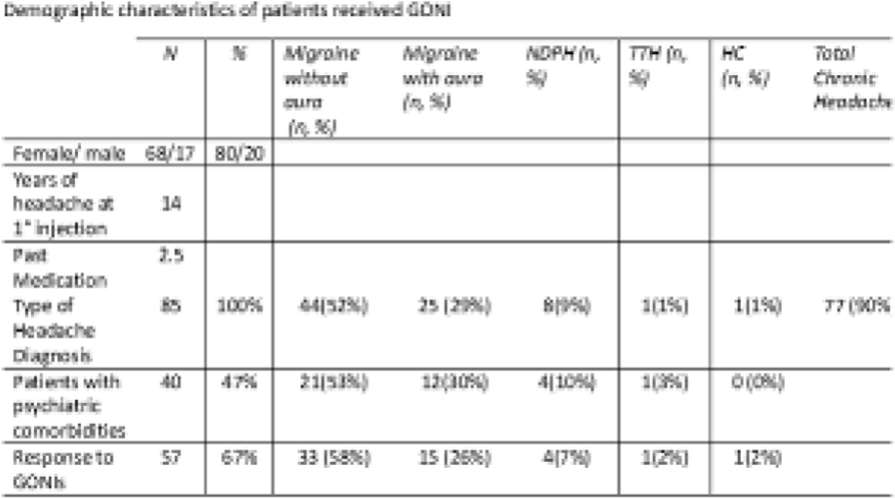
See text for description

## P157 OnabotulinumtoxinA for the prevention of chronic migraine in adolescents: the experience of an italian third level headache center

### I. Frattale, F. Ursitti, G. Sforza, G. Monte, M. A. N. Ferilli, S. Tarantino, M. Proietti Checchi, M. Valeriani, L. Papetti

#### Bambino Gesù Children's Hospital, Developmental Neurology Unit, Rome, Italy

##### **Correspondence:** I. Frattale

*The Journal of Headache and Pain 2024,* **25(Suppl 1)**: P157

**Objective:** Migraine is the main cause of headache in children with the possibility of turning into a chronic form in up to 5% of cases, causing severe disability and daily activities impairment. The use of onabotulinumtoxinA (BT-A) for the treatment of chronic migraine in adults represents one of the greatest efficacy treatments with many safety data collected. Only few data to date about the use in evolutive age. The present study aims to describe the experience with BT-A for the treatment of chronic migraine in adolescents.

**Methods:** All patients under the age of 18 treated with BT-A at the Headache Center of the Bambino Gesù Children Hospital from November 2018 to November 2022 were included. The patients underwent BT-A injections according to PREEMPT protocol.

Parameters considered were demographic characteristics, number of injections received, adverse effects and responder to treatment. We classified patients in responders if achieving a ≥50% reduction in Monthly migraine days (MMD), partial responders if achieving a 30–49% reduction in MMD and non-responders if achieving a <30% reduction from the baseline.

**Results:** The treated population consisted of 46 patients, with a mean age of 14.7 ± 1.5 years. 37 were females.

Before starting the BT-A, all subjects had previously attempted at least one prophylactic therapy and 58,7% discontinued them due to side effects. The number of BT-A administrations for each patient was 3.4 ± 3. Five patients discontinued the therapy after the first administration because of untoleration to the injections. No treated patients reported major side effects. One patient reported neck muscle weakness, 3 reported injection site redness or mild oedema. A response to treatment was observed in 35% of subjects at the first administration and 68% were responders (partially e complete) to treatment within the first three administrations of OBT-A, with a progressive improvement with the number of administrations.

**Conclusion:** this case series shows the excellent safe profile and effectiveness of BT-A in pediatric age of preventive treatment of chronic migraine.

**Fig. 1 (Abstract P157):**
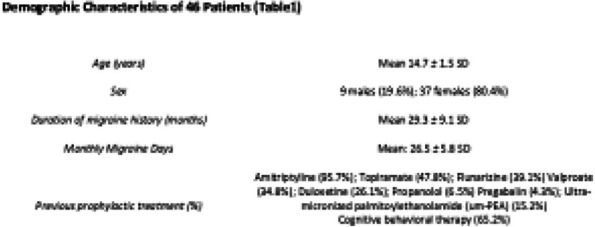
See text for description

**Fig. 2 (Abstract P157):**
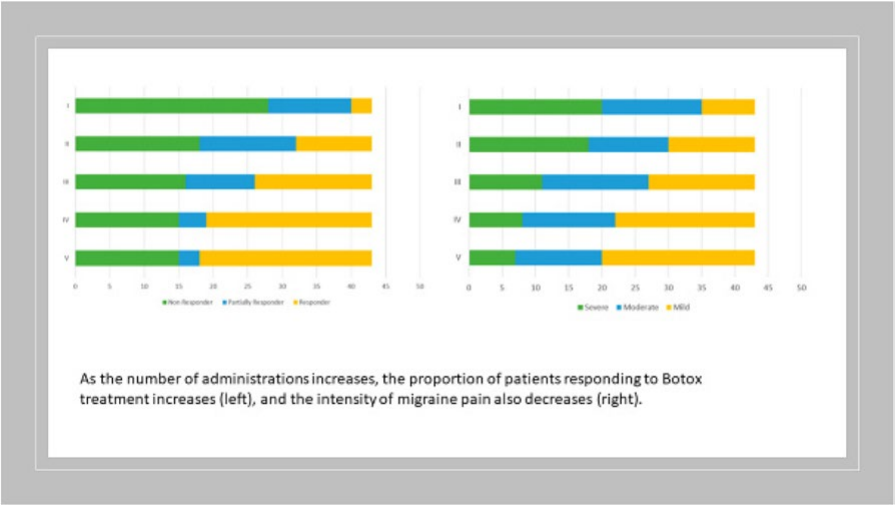
See text for description

## P158 Efficacy and safety of onabotulinumtoxinA in pediatric patients with chronic migraine

### E. Caronna, R. Mas-de-les-Valls-Cerco, V. J. Gallardo, L. Gómez-Dabó, A. Alpuente, M. Torres-Ferrús, P. Pozo-Rosich

#### Vall d’Hebron Hospital & Research Institute, Universitat Autonoma de Barcelona, Headache Clinic, Neurology Department, Barcelona, Spain

##### **Correspondence:** E. Caronna*,* L. Gómez-Dabó

*The Journal of Headache and Pain 2024,* **25(Suppl 1)**: P158

**Objective:** To analyze the efficacy and safety of onabotulinumtoxinA (BTX-A) in pediatric patients with chronic (resistant) migraine.

**Methods:** Prospective study including patients under 18 years of age with chronic migraine treated with BTX-A (PREEMPT protocol) as compassionate use. Demographic data, efficacy variables (headache days/month-HDM; migraine days/month-MMD, analgesic days/month-AMDM) and side effects were collected. A ≥50% reduction in HDM was considered response to BTX-A. Efficacy and safety were analyzed at 6 and 12 months.

**Results:** 17 patients were included, median age 15 years [14, 16]; 13 were female (76.5%). Most frequent comorbidities: anxiety (2/17) and depression (3/17). The median age at chronification was 13 [10, 14]. Basal frequency: 24.2±5.1 HDM, 17.9±9.2 MMD and 14.0±8.7 AMDM. At 6 months (n=17), 11 patients (64.71%) were responders, with a mean reduction in HDM of -11.2±11.2 (p=0.011). At 12 months (n=12), 8 patients (66.7%) were responders, with a mean reduction in HDM of -15.0±7.4 (p=0.015). No adverse effects were reported. 3 patients discontinued treatment before 12 months due to clinical improvement.

**Conclusion:** BTX-A is effective, well tolerated, and safe in adolescents with chronic migraine resistant to oral preventatives. Our data support the use of BTX-A as a therapeutic tool in the pediatric population.

## P159 Sleep and pain perception in children and adolescents with primary headaches and the effect of olfactory training on pain perception

### B. Höfer^1^, J. Knipping^1^, M. Richter^1^, M. Pieniak^2^, A. Hübler^1^, R. Sabatowski^1^, A. Hähner^2^, V. Schriever^3^, G. Gossrau^1^

#### ^1^Carl Gustav Carus University Hospital, TU Dresden, Headache Clinic, University Pain Center, Dresden, Germany; ^2^University Hospital Carl Gustav Carus, TU, Interdisciplinary Center for Smell and Taste of the University Hospital Carl Gustav Carus, Dresden, Germany; ^3^Charité – Universitätsmedizin Berlin, Department of Pediatric Neurology, Berlin, Germany

##### **Correspondence:** B. Höfer

*The Journal of Headache and Pain 2024,* **25(Suppl 1)**: P159

**Objective:** We comparatively investigate pain perception and sleep and the effect of a three-month placebo-controlled olfactory training on pain perception in children and adolescents with primary headaches and healthy individuals.

**Methods:** We examined 173 children and adolescents (103 with primary headaches, 70 healthy individuals). We collected data on headache diagnosis (ICHD III), clinical parameters, Depression Inventory Questionnaire for Children and Adolescents (DIKJ), State and Trait Anxiety (Staik S/T), Pittsburgh Sleep Quality Index (PSQI), Pediatric Migraine Disability Assessment Score (PedMIDAS)). Furthermore, we performed psychophysical testing of sensory and pain thresholds using Quantitative Sensory Testing (QST.)

Additional 89 children and adolescents with primary headaches performed a three-month placebo-controlled olfactory training (OT). We compared pain thresholds and sleep quality before and after the olfactory training.

**Results:** Children and adolescents with primary headaches (PH) have significantly worse sleep quality than healthy controls (C) (Mean PSQI: PH 5.99±0.334, C 3.64±0.261; *p*<.001). Furthermore, patients showed significantly higher pain sensitivity and lower mechanical pain thresholds (*p*=0.028).

Children and adolescents with primary headaches had significantly more comorbidities than healthy individuals (*p*<.001), other pain disorders in 11.49% and psychiatric comorbidities in 14.49% of children and adolescents with primary headaches.

After OT children and adolescents showed increased pain thresholds (*p*<.001) and lower pain sensitivity (*p*<.001) than patients with placebo training. The headache diagnosis had no influence on the effect of the OT regarding pain threshold (*p*=0.092) and pain sensitivity (*p*=0.563).

**Conclusion:** Patients with primary headaches showed increased pain sensitivity and decreased sleep quality compared to healthy controls. OT can decrease pain sensitivity in Children and adolescents with primary headaches.

## P160 Ketogenic diet in migraine: an experience of pediatric headache center

### G. Sforza, R. Moavero, L. Papetti, F. Ursitti, G. Monte, S. Tarantino, M. Proietti Checchi, M. Valeriani

#### Bambino Gesù Children's Hospital, Developmental Neurology Unit, Rome, Italy

##### **Correspondence:** G. Sforza

*The Journal of Headache and Pain 2024,* **25(Suppl 1)**: P160

**Objective:** To evaluate the efficacy and tolerability of the ketogenic diet in pediatric patients with chronic migraine. The ketogenic diet (KD) is a safe and well tolerated therapeutic tool for various metabolic and neurological disorders, used successfully for more than a century for epilepsy but its potential therapeutic efficacy on migraine has been little explored, especially in pediatric age. Different experimental models show that KD is able to reduce the propagation of cortical diffusion depression and to decrease cerebral excitability favoring GABAergic transmission. Furthermore, KD and ketone bodies can inhibit neuroinflammation, oxidative stress and free radical formation, which are processes involved in the pathophysiology of migraine

**Methods:** We conducted a retrospective observational study on 5 chronic migraine patients who received a KD; mean age was 15 years, all female, mean baseline BMI was 25.8; baseline frequency of headache was 24.4 episodes/months. Were evaluated at the baseline and then after 1, 3 and 6 months both from a neurological and a nutritional point of view, including BMI.

**Results:** Only 2 patients completed the 6-month follow-up (FU); 2 patients discontinued the ketogenic diet respectively after 3 weeks and after one month due to metabolic acidosis and side effects. One patient discontinued after 3 months due to poor efficacy. In the two patients who completed FU, there was a reduction in headache frequency greater than 50% in one patient and less than 50% in the other, respectively. Only in one of the two patients there was also a reduction in BMI (from 24 to 20.1). In both patients there was a significant difficulty in adhering to the dietary pattern and they stopped DK at the end of FU.

**Conclusion:** KD as a preventive treatment for migraine seems to be effective, also in pediatric age; however, in children there is greater difficulty in adherence and tolerability to the dietary regimen, which is difficult to apply as a first-line prophylaxis.

## P162 Hemifacial spasm as initial presentation of idiopathic intracranial hypertension

### C. Pires^1^, J. Ferreira^2^, D. Rodrigues^2^, L. Neto^2^, C. Guarda^1^

#### ^1^Centro Hospitalar Barreiro Montijo, Neurology, Barreiro, Portugal; ^2^Hospital Cuf Descobertas, Lisbon, Portugal

##### **Correspondence:** C. Pires

*The Journal of Headache and Pain 2024,* **25(Suppl 1)**: P162

**Objective:** To present a case of Idiopathic intracranial hypertension (IIH), initially manifested by hemifacial spasm (HFS), occurring several years before the onset of headache. IIH typically presents with headache, tinnitus, ocular findings, and less frequently, sixth cranial nerve palsy. Other false localising signs, such as seventh cranial nerve dysfunction, have rarely been reported, but are usually associated with headache.

**Methods:** Case report and literature review.

**Results:** A 41-year-old woman, with a 5-year history of right-sided facial twitching and tinnitus, presented with daily persistent headache. The headache was pulsatile, located in the right temporal area, and had worsened over the past three months. She experienced associated symptoms of nausea, photophobia and transient visual obscurations. The headache and the facial twitching would worsen when lying down. No diplopia or dysautonomic features were identified. Her body mass index was 35 kg/m2. On examination bilateral papilloedema and right HFS were observed. Optic coherence tomography revealed increased thickness of both optic nerves. Imaging showed an empty sela and narrowing of both the lateral and sigmoid sinuses. Lumbar puncture (LP) opening pressure (OP) was measured over 50cmH_2_O. Following the LP, there was an immediate improvement of the right HFS.

**Conclusion:** To the best of our knowledge this is the first reported case of IHH presenting as HFS several years before the onset of headache. This case emphasizes the importance of considering IIH as a possible diagnosis in the presence of atypical clinical features, such as hemifacial spasm associated with persistent tinnitus and suggestive neuroimaging or ophthalmologic IIH features, even in the absence of headache. Prompt diagnosis and treatment are crucial. The elevated OP and immediate resolution of the HFS after the LP further confirmed the aetiology of the clinical presentation. In this case the diagnosis was delayed by 5 years.

*Disclosure statement*: Informed consent to publish this case study and its potentially identifiable information of the patient was obtained from the individual involved. The patient gave explicit permission for the publication of this case report, including any relevant clinical details.

## P164 Treatment with anti-CGRP mAbs in patients with idiopathic intracranial hypertension: a pilot study

### N. Krajnc^1,2^, S. Macher^1,2^, W. Marik^2,3^, M. Michl^4^, K. Novak^2,5^, C. Wöber^1,2^, B. Pemp^4^, G. Bsteh^1,2^

#### ^1^Medical University of Vienna, Department of Neurology, Vienna, Austria; ^2^Medical University of Vienna, Comprehensive Center for Clinical Neurosciences & Mental Health, Vienna, Austria; ^3^Medical University of Vienna, Division of Neuroradiology and Musculoskeletal Radiology, Vienna, Austria; ^4^Medical University of Vienna, Department of Ophthalmology, Vienna, Austria; ^5^Medical University of Vienna, Department of Neurosurgery, Vienna, Austria

##### **Correspondence:** N. Krajnc

*The Journal of Headache and Pain 2024,* **25(Suppl 1)**: P164

**Objective:** Idiopathic intracranial hypertension (IIH) is a disease mostly occurring in young obese women, often presenting with chronic migraine-like headache. Monoclonal antibodies (mAbs) against calcitonin gene-related peptide (CGRP) or its receptor are a novel effective preventive treatment in migraine patients.

**Methods:** In this pilot single-centre study, pwIIH with resolved papilledema, yet persisting migraine-like headache, were offered to receive anti-CGRP mAbs. The primary endpoint was mean change in number of headache days/month after three (M3) and six months (M6). Secondary endpoints were defined as follows: (1) reduction of acute headache medication use, (2) improvement of headache days (≥50%) or headache freedom, (3) adverse events (AE).

**Results:** Eight pwIIH (mean age 30.5 years [SD 10.3], 100% female, median disease duration 1.2 years [IQR 0.4–6.8])were included (erenumab: 6, fremanezumab: 2). Mean number of headache days/month at baseline was 15.3 (5.3). The latter was reduced by 8.4 (5.7) and 9.5 (5.3) days at M3 and M6, respectively (*p*<0.001). Mean number of days/month with acute headache medication was reduced at M3 (–3.8 [2.9], *p*=0.004) and M6 (–4.5 [3.3], *p*<0.001). Improvement in headache days was seen at M3 (4 [50.0%]) and M6 (5 [62.5%]), whereas headache freedom was achieved in one patient at M3 but none at M6. No serious AE were observed. One patient experienced transient injection-site reaction, and no infections or elevation of liver enzymes were noted. There was no discontinuation of treatment.

**Conclusion:** Anti-CGRP mAbs may be a safe, efficient and well-tolerated treatment option in pwIIH with migraine-like headache persisting after papilledema resolution.

**Fig. 1 (Abstract P164):**
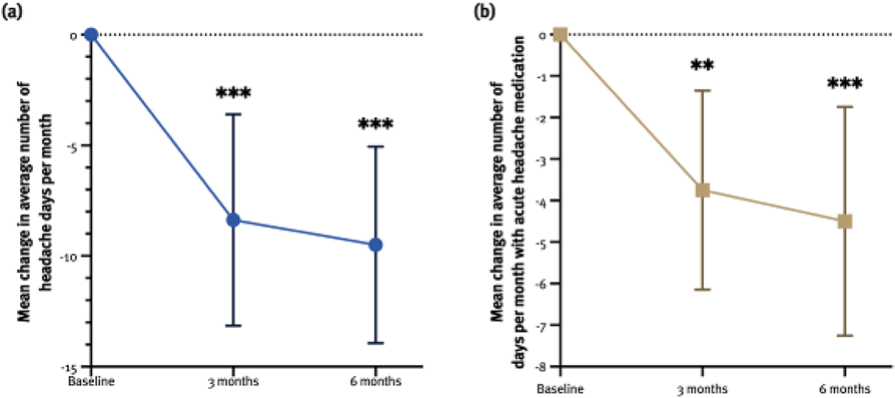
See text for description

## P165 Treatment with GLP-1 receptor agonists is associated with significant weight loss and favorable headache outcomes in idiopathic intracranial hypertension

### N. Krajnc^1,2^, B. Itariu^3^, S. Macher^1,2^, W. Marik^2,4^, J. Harreiter^3^, M. Michl^5^, K. Novak^2,6^, C. Wöber^1,2^, B. Pemp^5^, G. Bsteh^1,2^

#### ^1^Medical University of Vienna, Department of Neurology, Vienna, Austria; ^2^Medical University of Vienna, Comprehensive Center for Clinical Neurosciences & Mental Health, Vienna, Austria; ^3^Medical University of Vienna, Division of Endocrinology, Department of Internal Medicine, Vienna, Austria; ^4^Medical University of Vienna, Division of Neuroradiology and Musculoskeletal Radiology, Vienna, Austria; ^5^Medical University of Vienna, Department of Ophthalmology, Vienna, Austria; ^6^Medical University of Vienna, Department of Neurosurgery, Vienna, Austria

##### **Correspondence:** N. Krajnc

*The Journal of Headache and Pain 2024,* **25(Suppl 1)**: P165

**Link to published article:**

https://thejournalofheadacheandpain.biomedcentral.com/articles/10.1186/s10194-023-01631-z

**Fig. 1 (Abstract P165):**
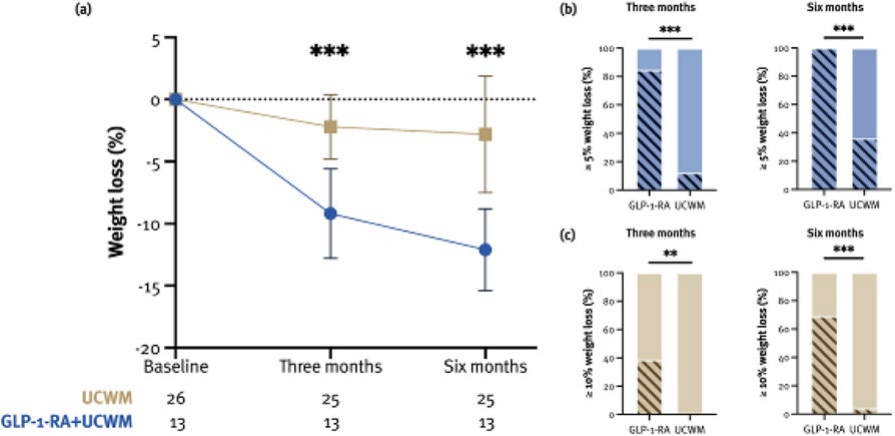
See text for description

**Fig. 2 (Abstract P165):**
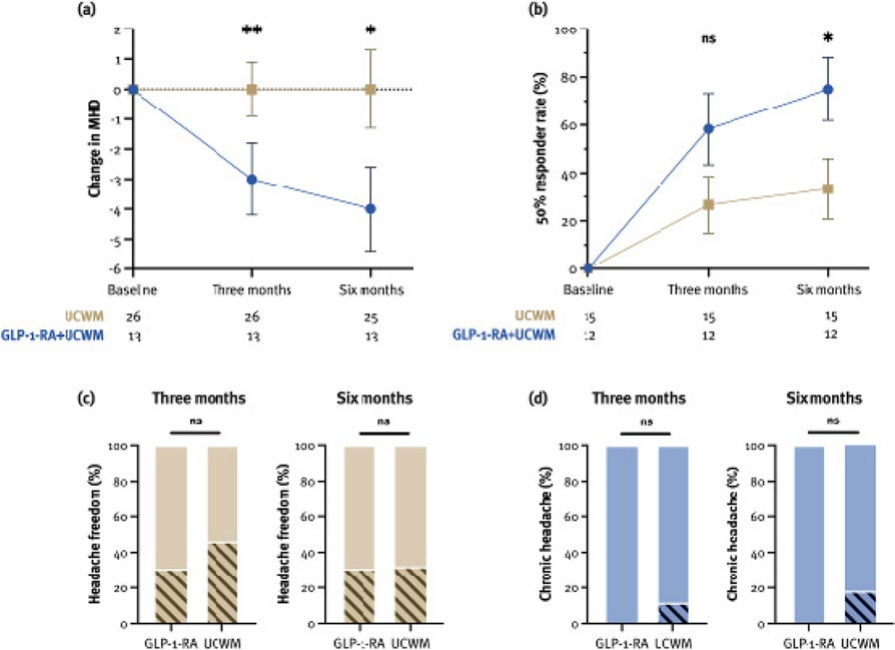
See text for description

**Fig. 3 (Abstract P165):**
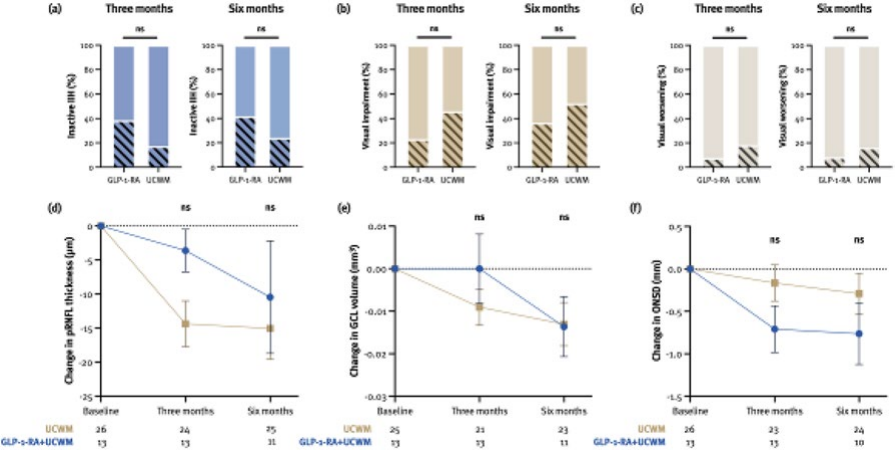
See text for description

## P166 The prevalence of migraine headache in patients with MS: a case series

### Z. Ebadi^1^, M. Togha^1^, A. Nasermoghadasi^1^, N. Rezaeimanesh^1^, S. Razeghi Jahromi^2^

#### ^1^Tehran University of Medical Sciences, Tehran, Iran; ^2^Shahid Beheshti University of Medical Sciences, Department of Clinical Nutrition and Dietetics, Tehran, Iran

##### **Correspondence:** S. Razeghi Jahromi

*The Journal of Headache and Pain 2024,* **25(Suppl 1)**: P166

**Objective:** The purpose of this study is to investigate the prevalence and characteristics of migraine-type headaches in patients with multiple sclerosis (PwMS).

**Methods:** A questionnaire was developed following the criteria of the international society of Headaches and was provided to patients with MS online. The study was conducted in the Sina Hospital affiliated with Tehran University of Medical Sciences, Tehran. Iran.

**Results:** Approximately 360 pwMS filled out the questionnaire. About 90% of these patients were women. The average duration of the disease was about 10 years. About 70% of the patients were relapsing-remitting MS, 10% primary progressive MS, and 15 % secondary progressive MS. The most common disease-modifying drugs were platform drugs. According to the international headache society criteria, approximately 43% of patients reported migraine headaches. More than 40% of patients reported their headaches as very severe pain. The most commonly used migraine prophylactic medications were nortriptyline, propranolol, and gabapentin. Nearly 50% of all patients reported headaches accompanied by MS relapses. Most of them pointed out their headaches in the preceding days before relapses. Of those who received corticosteroids, the majority reported improvement in headaches.

**Conclusion:** Migraine is one of the common headaches in patients with MS, and the characteristics of this headache in MS patients are similar to the healthy population. Some MS patients reported aggravation of their headaches before or during an attack, and the majority of them improved with steroids. But many headache sufferers have their attacks for a prolonged time and considerably affecting their quality of life. So it is important to consider migraine as a common and potentially disabling problem in MS patients. Also, as a headache might be a predictor of an MS attack, it could track more attention of the physicians to control it even before the start of a profound neurological deficit.

## P167 Prevalence of trigeminal neuralgia in patients with MS

### Z. Ebadi^1^, M. Togha^1^, A. Nasermoghadasi^1^, N. Rezaeimanesh^1^, S. Razeghi Jahromi^2^

#### ^1^Tehran University of Medical Sciences, Tehran, Iran; ^2^Shahid Beheshti University of Medical Sciences, Department of Clinical Nutrition and Dietetics, Tehran, Iran

##### **Correspondence:** S. Razeghi Jahromi

*The Journal of Headache and Pain 2024,* **25(Suppl 1)**: P167

**Objective:** MS is one of the main causes of disability in young people. One of the most important complaints in MS patients is pain. This pain occurs in a variety of ways. Trigeminal neuralgia is characterized by recurrent unilateral sharp pain attacks, often triggered by innocuous stimuli, which occur in the distribution areas of branches of the trigeminal nerve. It seems that there is an association between the occurrence of TN with MS. The prevalence of TN in the general population is less than 0.1%versus 1.1 and 6.3% in different studies of MS patients. This research aims to investigate the prevalence and characteristics of TN in patients with multiple sclerosis (PwMS) in the MS clinic, in Tehran.

**Methods:** A questionnaire was developed following the criteria of the international society of Headaches and was provided to patients with MS online. The study was conducted in the Sina Hospital of the Tehran University of Medical Sciences.

**Results:** About 350 patients with MS completed the questionnaire. The majority of these patients were women. The mean disease duration was about 9 years. Approximately 70% of the patients were diagnosed with relapsing-remitting MS, 10 % with primary progressive MS, and 14 % with secondary progressive MS. More than 40% of patients received platform drugs. According to the international headache society criteria, about 10% reported TN. More than 50%of patients mentioned improvement in their pain by conventional treatment of idiopathic trigeminal neuralgia. In contrast to classic and idiopathic TN, about 70% of MS patients with TN experience pain in the V1 branch of the trigeminal nerve. The pain has combined with jaw weakness in about 30% of patients. In about 20% of patients, trigeminal neuralgia leads to MS diagnosis. In nearly 30% of patients, the other side of the face also was involved. Nearly 10% of PwMS experienced more than one episode of TN attacks.

**Conclusion:** In our study, the prevalence of TN was about 10% which is significantly higher than 0.1% in the general population. The ophthalmic (V1) division of the trigeminal nerve was the most common part of pain. In the majority of the study patients, the onset of MS preceded the onset of pain.

## P168 Spontaneous intracranial hypotension experience in a headache center in Argentina

### M. V. Nagel^1^, M. Aguilar^2^, H. Lambre^2^, A. Perez^3^, L. Bonamico^1^, Y. Bravo^1^, G. Portuondo^1^, S. Crema^1^, M. T. Goicochea^1^

#### ^1^FLENI, Neurology, Buenos Aires, Argentina; ^2^FLENI, Radiology Department, Buenos Aires, Argentina; ^3^FLENI, Anaesthesiology, Buenos Aires, Argentina

##### **Correspondence:** L. Bonamico

*The Journal of Headache and Pain 2024,* **25(Suppl 1)**: P168

**Objective:** To analyze the clinical characteristics of our patients with spontaneous intracranial hypotension (SIH) and evaluate the results of our workup algorithm.

**Methods:** Prospective study. Patients over 18 years old with SIH who did not responded to bedrest, evaluated in our center from July 2022 to May 2023, were included. To try to localize the CSF leak we performed a complete spine MRI. We selected targeted blood patch (BP) in patients with a leak visible in the MRI, otherwise we used blind BP. In patients with persistent headache at 2 weeks visit, a CT myelography (CTm) was performed. If the leak was found we did a targeted BP patch, if not, we repeated the blind BP. We analyzed sex, age, diagnostic delay, headache phenotype, related symptoms, image studies results, type of treatment, response at 1 and 6 months (after the last treatment).

**Results:** We included 11 patients (55% women). 45 years old in average. Mean diagnostic delay was of 40 days. All the patients suffered orthostatic headache. 10 patient had related symptoms: nausea and vomit 73%, tinnitus 45%, dizziness 36%. Brain MRI signs of hypotension was present in 10 patients (91%). Spine MRI showed epidural collection in 6 patients, but none with visible leak. Blind BP was performed in every patient. In 7 patients (64%) no other treatment was needed, being all of them headache free at 1 month visit. Regarding the 6 months visit, 4 patients were pain free, 1 did not reached it yet and 2 were lost. 4 patients did not responded to the first BP and underwent a CTm. In 3 of them the leak was found and a targeted BP was performed. At 1 month 2 patients were pain free and one is still pending. The only patient with no visible leak in the CTm underwent a second blind BP and was asymptomatic at 1 month (6 month visit was not reached yet).

**Conclusion:** Orthostatic headache was the most common symptom in SIH patients. Blind BP was effective to treat most patients. In our group, CTm was useful to localize the leak, while spine MRI was not.

## P170 Application of ultrasound measurement of the optic nerve sheath diameter as a diagnostic and follow-up test in idiopathic intracranial hypertension: a case report

### L. Moreno-Navarro^1,2^, M. Farrerons-Llopart^1^, N. López-Hernández^1^, I. Beltrán Blasco^1^, A. Fríes-Ramos^1^, C. Aledo-Sala^1^, M. D. Warnken-Miralles^1^, E. Ginés-Murcia^1^, L. Ruiz-Escribano-Menchén^1^

#### ^1^Hospital General Universitario Dr. Balmis, Neurology, Alicante, Spain; ^2^Instituto de Investigación Sanitaria Biomédica de Alicante (ISABIAL), Group 1: Neuroscience Research, Alicante, Spain

##### **Correspondence:** L. Moreno-Navarro

*The Journal of Headache and Pain 2024,* **25(Suppl 1)**: P170

**Objective:** This case report highlights the utility of orbital ultrasonography as a non-invasive diagnostic tool in suspected cases of headache attributed to idiopathic intracranial hypertension (IIH).

**Methods:** A 40-year-old obese male with no prior medical history was admitted to the Neurology ward presenting with a 10-day course of moderate-intensity holocranial and oppressive headache that was unresponsive to analgesics. The headache worsened while lying down and was accompanied by nausea but no vomiting. The patient denied diplopia or visual deficits. Neurological examination and fundoscopic evaluation revealed no abnormalities.

**Results:** Based on clinical suspicion of headache due to intracranial hypertension, a brain magnetic resonance imaging was conducted, revealing posterior sclera flattening and vertical tortuosity of both optic nerves, suggestive of IIH. A lumbar puncture (LP) was performed, obtaining a cerebrospinal fluid (CSF) with an opening pressure at the upper limit of normal (19 cm H2O in lateral decubitus position). Cytobiochemical and microbiological analysis of CSF were normal. In order to avoid a repeat LP, an orbital ultrasonography was performed, revealing a mild papilledema and bilateral optic nerve thickening. Measurement of the optic nerve sheath diameter (ONSD) at 3 mm behind the optic disc showed high values of 9.6 mm in the right eye and 9.3 mm in the left eye, strongly indicative of intracranial hypertension. Following treatment with acetazolamide, the patient showed progressive improvement of his symptoms, with an early restoration of the ONSD value.

**Conclusion:** It has been determined that threshold value above which the ONSD indicates intracranial hypertension is 5.7 mm (sensitivity and specificity close to 100%). It can be detected earlier than papilledema during fundoscopic exam. Thus, ultrasound measurement of the ONSD is considered a potential tool for early diagnosis and monitoring of IIH.

*Disclosure statement:* Informed consent to publish this case study and its potentially identifiable information of the patient was obtained from the individual involved. The patient gave explicit permission for the publication of this case report, including any relevant clinical details.

**Fig. 1 (Abstract P170):**
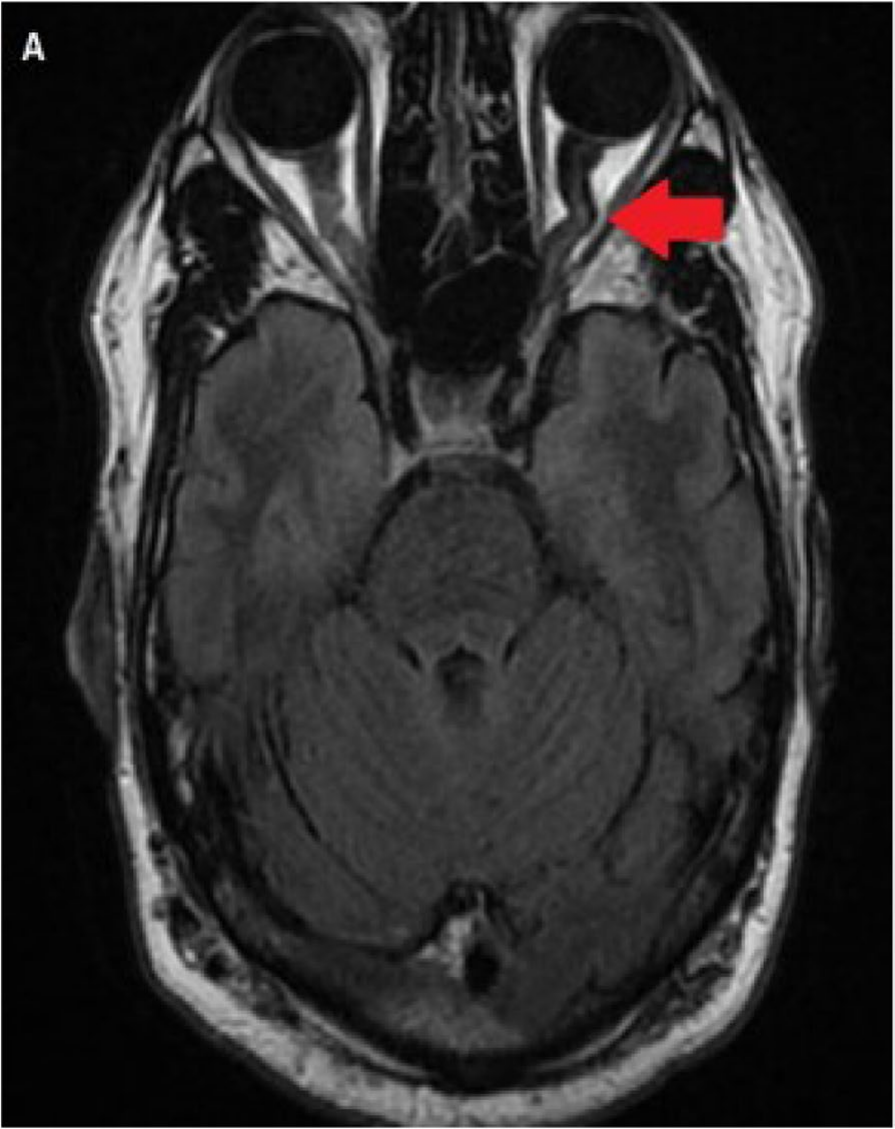
See text for description

**Fig. 2 (Abstract P170):**
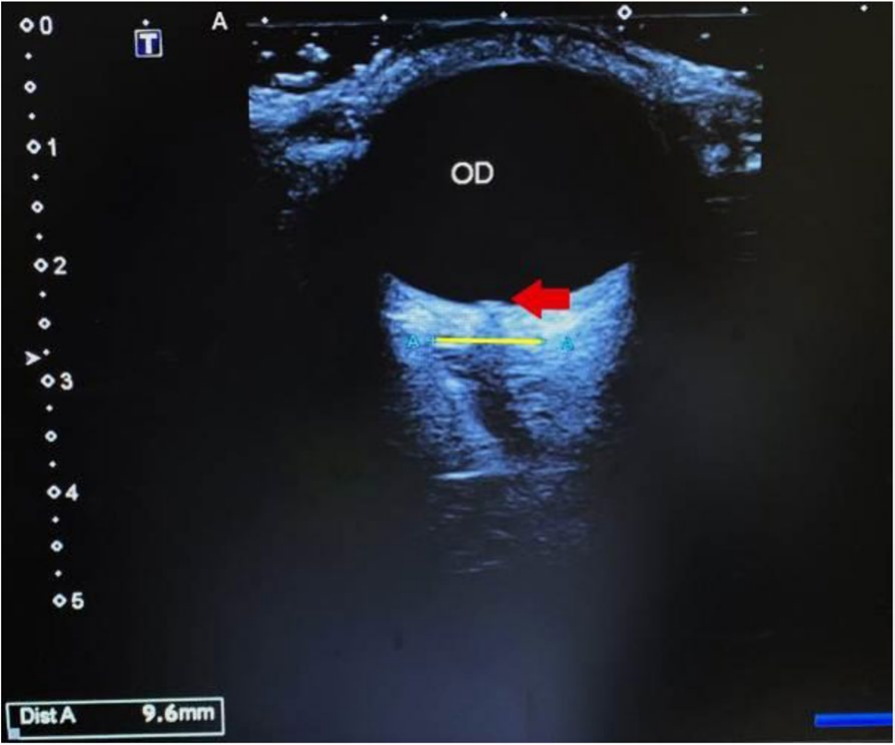
See text for description

**Fig. 3 (Abstract P170):**
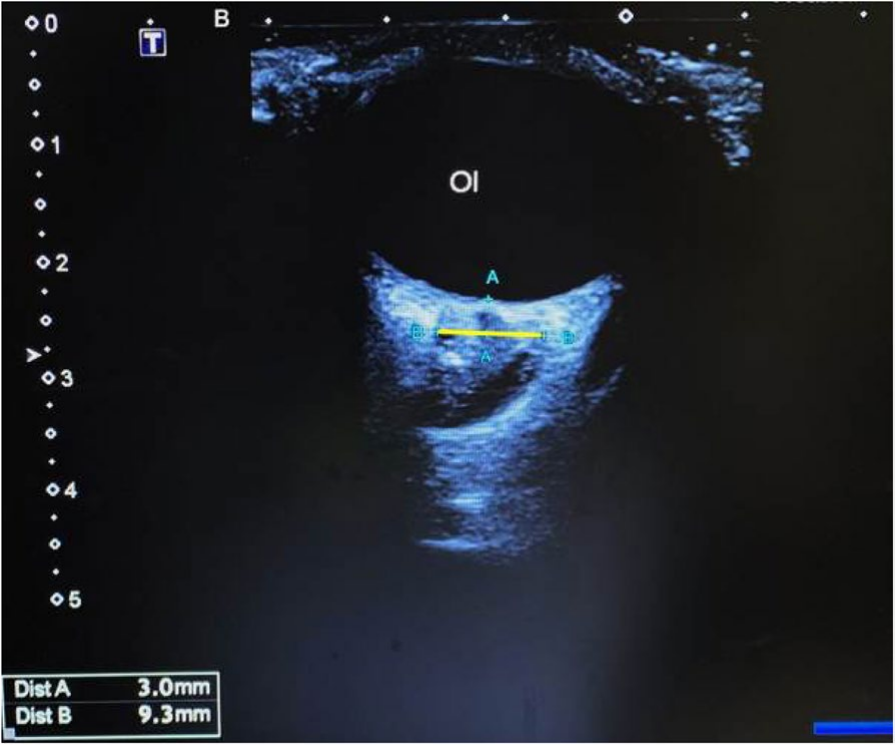
See text for description

## P171 Spectrum of new-onset acute or subacute headache not explained by brain CT

### L. Joppekova^1,2^, I. Srotova^1,2^, I. Niedermayerova^1^, T. Kubecova^1^, J. Bednarik^1,2^, E. Vlckova^1,2^

#### ^1^University Hospital Brno, Department of Neurology, Brno, Czech Republic; ^2^Masaryk University, Faculty of Medicine, Brno, Czech Republic

##### **Correspondence:** L. Joppekova

*The Journal of Headache and Pain 2024,* **25(Suppl 1)**: P171

**Objective:** The aim of this study was to determine the spectrum of headache causes in patients with new-onset headache that was not explained by the initial CT scan. We also wanted to identify the clinical variables associated with a higher likelihood of secondary headache in this group and to determine the importance of cerebrospinal fluid (CSF) examination in these patients.

**Methods:** In this retrospective study, the medical records of 218 patients (140 women, median age 44 years, range 15 - 87) admitted to the large university hospital between 2016 and 2023 with new-onset headache lasting up to 2 weeks and a negative initial CT scan were reviewed. All the patients underwent CSF examination and (in unexplained cases) other imaging (mostly MR and MR-venography).

**Results:** Secondary headache (SH) was found in 86 patients (39.4%), while 132 patients (60.6%) had primary headache (mostly migraine with or without aura). The CSF was abnormal in 36 patients (16.5% of the total group): meningitis or meningoencephalitis was found in 26 cases, subarachnoid haemorrhage (SAH) was confirmed in 10 patients (of which 2 were aneurysmal). The spectrum of other SH causes included intracranial venous thrombosis (14 patients), cerebral infarction (11), Tolosa-Hunt syndrome (8), and a few others. On a group level, patients with SH were older and more likely to have abnormal findings on clinical neurological examination. SH was also more likely in patients with longer duration of symptoms: it was confirmed in about 2/3 of patients with symptoms lasting more than a week, but only in 1/3 of those with symptoms lasting less than 24 hours.

**Conclusion:** The study confirms the importance of CSF examination in new-onset short-term headache with negative CT scan. In addition to neuroinfections, it allows the detection of SAH, which is confirmed in about 5% of such patients. Secondary headache is more likely in older patients with objective neurological abnormalities and longer duration of symptoms.

## P173 Stenting in idiopathic intracranial hypertension (IIH): from pathophysiology to treatment

### I. M. Lucas Requena, F. J. Alberola Amores

#### Hospital General Universitario de Elche, Neurología, Elche, Spain

##### **Correspondence:** I. M. Lucas Requena

*The Journal of Headache and Pain 2024,* **25(Suppl 1)**: P173

**Objective:** IIH is a disease characterized by increased intracranial pressure typically in young and obese population, whose pathogenesis remains unknown. Controversy still exists as to its pathophysiology, with three possible intracranial mechanisms described: alterations in CSF dynamics, increased venous sinus pressure, and hormonal and metabolic mechanism. We present the case of a patient diagnosed with ICH refractory to medical treatment who was treated with venous stenting.

**Methods:** A 25-year-old male with a history of obesity was referred from Ophthalmology with clinical symptoms of loss of visual acuity of 3 months of evolution and bilateral papilledema in the ocular fundus. He had oppressive holocranial headache with alarm symptoms. A cranial scan was performed, which was normal, and later a lumbar puncture, with outflow pressure > 55 cmH20, leaving an outflow pressure of 15cmH20, improving clinically. The study was completed with blood test, cerebral MRI with arterial/venous vascular study, in which alteration in the transverse venous sinus was observed.

**Results:** Medical treatment was optimized, requiring a progressive increase up to maximum doses of acetazolamide and furosemide, requiring a new lumbar puncture for evacuation. The study was completed with cerebral angiography, which confirmed a stenosis of the transverse sinus with a gradient of more than 8 mmHg, so a stent was placed, with complete recovery of the caliber. The patient is currently asymptomatic with withdrawal of medication.

**Conclusion:** Venous sinus stenting is a growing treatment option in selected patients who are refractory to medical treatment that shows a lower rate of complications and recurrences compared to other invasive treatments.

*Disclosure statement:* Informed consent to publish this case study and its potentially identifiable information of the patient was obtained from the individual involved. The patient gave explicit permission for the publication of this case report, including any relevant clinical details.

## P174 Treatment response of venous sinus stenting in patients with refractory idiopathic intracranial hypertension and correlation of venous manometry findings with magnetic resonance venography and high-resolution T1-contrast measurements

### A. Nisar^1^, E. Qiu^1^, I. Sharan^2^, J. Chen^2^, N. Arunkumar^2^, P. Mondel^3^, K. Talekar^3^, N. Jain^3^, S. Thumar^3^, F. Alfaer^3^, K. E. Naamani^4^, J. Evans^4^, S. Faro^3^, M. Marmura^1^, N. Spare^1^, S. Parikh^1^, H. Yuan^1^, M. R. Gooch^4^

#### ^1^Thomas Jefferson University Hospital, Jefferson Headache Center, Department of Neurology, Philadelphia, PA, United States; ^2^Thomas Jefferson University Hospital, Sidney Kimmel Medical College, Philadelphia, PA, United States; ^3^Thomas Jefferson University Hospital, Department of Radiology, Philadelphia, PA, United States; ^4^Thomas Jefferson University Hospital, Department of Neurosurgery, Philadelphia, PA, United States

##### **Correspondence:** H. Yuan

*The Journal of Headache and Pain 2024,* **25(Suppl 1)**: P174

**Objective:** What is the treatment response of transverse sinus stenting (TSS) in patients with refractory idiopathic intracranial hypertension (rIIH), and how venous manometry correlates with magnetic resonance venography (MRV) and high-resolution T1-contrast (HRTC) measurements?

**Methods:** A retrospective study evaluated patients who received venous manometry for rIIH between 1/2022-12/2022 at Thomas Jefferson University. Subjects with prior brain/spine surgery or those with secondary causes of elevated intracranial hypertension were excluded. TSS responses were assessed at 3 and 6 months post-TSS. Pre-TSS venous assessments, using MRV and HRTC, were correlated with venous manometry findings (trans-stenotic pressure gradient [TSPG] ≥8 mmHg).

**Results:** Twenty subjects who underwent venous manometry were identified; eighteen (female 17, age 39.6±10.8, BMI 35.7±8.6; Caucasian 9, African American 8) received TSS. Post-TSS 3-month responses (TSPG <8 [*n*=6] vs. ≥8 [*n*=12]) were reportedly better/no change/worse/no data for headache (4/0/1/1 vs. 8/1/0/3), vision (3/1/0/2 vs. 4/3/0/5), papilledema (1/0/0/5 vs. 2/0/0/10), and pulsatile tinnitus (3/0/0/3 vs. 4/1/1/6); 6-month responses for headache (1/1/1/3 vs. 6/0/1/5), vision (1/1/0/4 vs. 3/1/0/8), papilledema (1/1/0/4 vs. 5/1/0/6), and pulsatile tinnitus (3/0/0/3 vs. 3/0/1/8). For pre-TSS assessment, unilateral stenosis (≥67%) with contralateral hypoplasia correlated with TSPG-confirmed stenosis (TSPG ≥8) in 5/6 (83.3%) vs. 2/2 (100%) subjects using MRV vs. HRTC, respectively, whereas unilateral stenosis alone correlated in 1/3 (33.3%) vs. 0/1 (0%), bilateral stenosis in 4/8 (50.0%) vs. 3/6 (50.0%), and no stenosis in 0/2 (0%) vs. 0/1 (0%) subjects.

**Conclusion:** TSS may improve rIIH symptoms, including headache, vision, papilledema, and tinnitus. Unilateral stenosis with contralateral hypoplasia on MRV/HRTC seemed to correlate with TSPG-confirmed stenosis more than bilateral stenosis or unilateral stenosis alone.

**Table 1 (Abstract P174):**
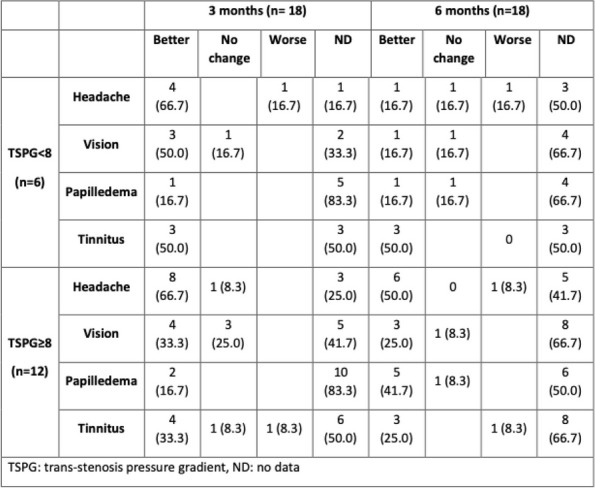
Transverse sinus stenting response at 3 and 6 month follow-up

## P175 Sustained effectiveness of Bariatric Surgery beyond 5 years in Idiopathic Intracranial Hypertension: the IIH Weight Trial

### M. Thaller^1,2^, M. Sassani^1^, H. Lyons^1^, A. Yiangou^1^, J. Mitchell^1,2^, S. Mollan^1,3^, A. Sinclair^1,2^

#### ^1^University of Birmingham Institute of Metabolism and Systems Research, Translational Brain Science, Birmingham, United Kingdom; ^2^Queen Elizabeth Hospital Birmingham, Neurology, Birmingham, United Kingdom; ^3^Queen Elizabeth Hospital Birmingham, Birmingham Neuro-Ophthalmology, Birmingham, United Kingdom

##### **Correspondence:** M. Thaller

*The Journal of Headache and Pain 2024,* **25(Suppl 1)**: P175

**Objective:** Idiopathic intracranial hypertension, a systemic metabolic disease, causes headaches and visual loss. Sustained weight loss is the only disease modifying therapy. The objective was to evaluate the effectiveness of bariatric surgery as compared to a dietary intervention beyond 5 years in people with active IIH.

**Methods:** This 5-year randomized clinical trial (Idiopathic Intracranial Hypertension Weight Trial) enrolled women with active IIH and a body mass index ≥35 kg/m2 between March 1, 2014, and May 25, 2017, for either bariatric surgery or community weight management. Outcome measures included ICP measured by lumbar puncture and anthropometric measures after 60 months.

**Results:** Twenty-eight participants were followed up for the 60-month visit. Eleven had received bariatric surgery within the first 12 months of randomisation. A further 4 were initially randomised to community weight management but had bariatric surgery after 24 months. Participants in the surgical arm lost significantly more weight compared to baseline with mean (SD) reduction of 18.0kg (13.4) versus an increase in weight of 4.9kg (18.1) in the community weight management arm, p=0.0049. A greater reduction in intracranial pressure compared to baseline was also demonstrated at the 60-month visit in the surgical arm with a reduction of 12.5 cmCSF (8.5) vs 3.8 cmCSF (8.0) in the community weight management arm, p=0.064. The reductions in the weight and intracranial pressure in the surgical arm were sustained since the 12- and 24-months visits.

**Conclusion:** There was maintenance of effect for both weight loss and intracranial pressure reduction beyond 5 years post bariatric surgery. The persistent improvement demonstrates sustained disease remission.

## P176 Headache as an aura in patients with focal and generalized seizures

### D. W. Kim, D. W. Kwack

#### Konkuk University School of Medicine, Seoul, South Korea

##### **Correspondence:** D. W. Kim

*The Journal of Headache and Pain 2024,* **25(Suppl 1)**: P176

**Objective:** Although aura in epilepsy is usually considered as a phenomenon in patients with focal seizures, headache can occur as an aura or solitary epileptic symptom in patients with generalized seizures. There is only limited information on the headache as an aura in patients with focal and generalized seizures

**Methods:** We performed a 14-year retrospective study of patients with focal and generalized seizures and analyzed the proportion and characteristics of patients with auras including headache.

**Results:** In our Cohort, 1,632 patients were classified as having focal seizures, while 162 patients were classified as having generalized seizures. We excluded 311 patients with focal seizures and 60 patients with generalized seizure from analysis due to insufficient follow-up or lack of detailed clinical information. Among the 1321 patients with focal seizures, headache was described as an aura in 103 patients (7.8%) while headache was described as an aura in 26 patients with generalized seizures (25.2%) While there was no difference between patients with or without headache in focal seizures, the age of onset of seizures was significantly lower in patients with headache as an aura than in patients without headache in generalized seizures (14.8 ± 3.8 vs 24.7 ± 16.2, *p* = 0.003).

**Conclusion:** Our study showed that headache as an aura was more common in patients with generalized seizures and patients with a younger age of onset of seizures are more likely to experience headache as an aura in these patients.

## P177 Efficacy of ubrogepant for the treatment of migraine symptoms during the prodrome (premonitory phase): results from the PRODROME trial

### P. J. Goadsby^1,2^, J. Ailani^3^, D. W. Dodick^4^, A. J. Starling^4^, C. Liu^5^, S. Y. Yu^5^, E. Brand-Schieber^5^, M. Finnegan^5^, J. M. Trugman^5^

#### ^1^King's College London, London, United Kingdom; ^2^University of California, Los Angeles, CA, United States; ^3^MedStar Georgetown University Hospital, Washington, DC, United States; ^4^Mayo Clinic, Phoenix, AZ, United States; ^5^AbbVie, Madison, NJ, United States

##### **Correspondence:** P. J. Goadsby, J. M. Trugman

*The Journal of Headache and Pain 2024,* **25(Suppl 1)**: P177

**Objective:** To evaluate the efficacy of ubrogepant 100 mg to treat migraine symptoms during the prodrome (premonitory phase). Ubrogepant is a calcitonin gene-related peptide (CGRP) receptor antagonist approved for the acute treatment of migraine. The primary objective of the PRODROME trial was to evaluate the efficacy of ubrogepant to prevent or attenuate headache following administration during the prodrome.

**Methods:** PRODROME was a multicenter, randomized, double-blind, placebo-controlled, crossover trial that enrolled adults who experienced 28 migraine attacks with moderate-to-severe headache per month. Eligible participants treated 2 "qualifying prodrome events," defined as a migraine attack with prodromal symptoms in which the participant was confident a headache would follow within 1-6 hours. Participants used an e-diary to record the presence and severity of symptoms at the time of each qualifying prodrome event. We report the frequency and severity of common prodromal symptoms and the presence/absence of these symptoms over 48 hours post-dose.

**Results:** During the double-blind treatment period, the most common prodromal symptoms reported prior to study drug administration were (ubrogepant-treated and placebo-treated events, respectively) sensitivity to light (60.9% and 60.8%), fatigue (50.7% and 50.3%), neck pain (40.2% and 40.1%), sensitivity to sound (35.9% and 36.1%), and dizziness (29.0% and 31.0%). Between 30.8% and 57.2% of these symptoms were moderate or severe in intensity. The proportion of events with absence of sensitivity to light after ubrogepant 100 mg was numerically greater than after placebo, starting 2 hours post-dose and extending through 48 hours (nominal *P*≤0.0109 for hours 2-8). Time to absence of each individual prodromal symptom was shorter after treatment with ubrogepant than after placebo.

**Conclusion:** Treatment with ubrogepant 100 mg during the prodrome improved the common migraine prodromal symptoms compared with placebo.

## P178 Non-Exophytic carcinoma of the tongue: a rare etiology of continuous hemicranial pain

### J. Munoz-Cerón^1^, S. Castro^1,2^

#### ^1^Hospital Universitario Mayor Méderi – CIMED, Neurology, Bogotá, Colombia; ^2^Hospital Universitario Mayor Méderi – CIMED, Neurology, Bogotá, Colombia

##### **Correspondence:** J. Munoz-Cerón

*The Journal of Headache and Pain 2024,* **25(Suppl 1)**: P178

**Objective:** To describe non-exophytic carcinoma of the tongue as a etiology of hemicranial pain and to contribute to

**Methods:** Case report

**Results:** Most of the tumor lesions in the tongue are evident on physical examination, in cases in which this does not occur, it is possible that invasion of the adjacent neural structures could bring about headache as initial manifestation. We present the case of a 57-year-old man with complaining of persistent hemicranial left pain in whom, after extensive work up and multiple therapeutical interventions, including indomethacine, a non-exophytic carcinoma of the tongue with infiltration to the ipsilateral glossopharyngeal nerve was documented as the etiology of the headache. After surgical treatment, chemotherapy, radiotherapy and follow up for 2 years, the patient has remained asymptomatic.

**Conclusion:** This case report suggests considering the tongue as a structure of potential origin for secondary etiologies of persistent hemicranial headache and reports a clinical variant of *Painful glossopharyngeal neuropathy attributed to a known cause (ICHD - 13.2.2.1)*

*Disclosure statement:* Informed consent to publish this case study and its potentially identifiable information of the patient was obtained from the individual involved. The patient gave explicit permission for the publication of this case report, including any relevant clinical details.

**Fig. 1 (Abstract P178):**
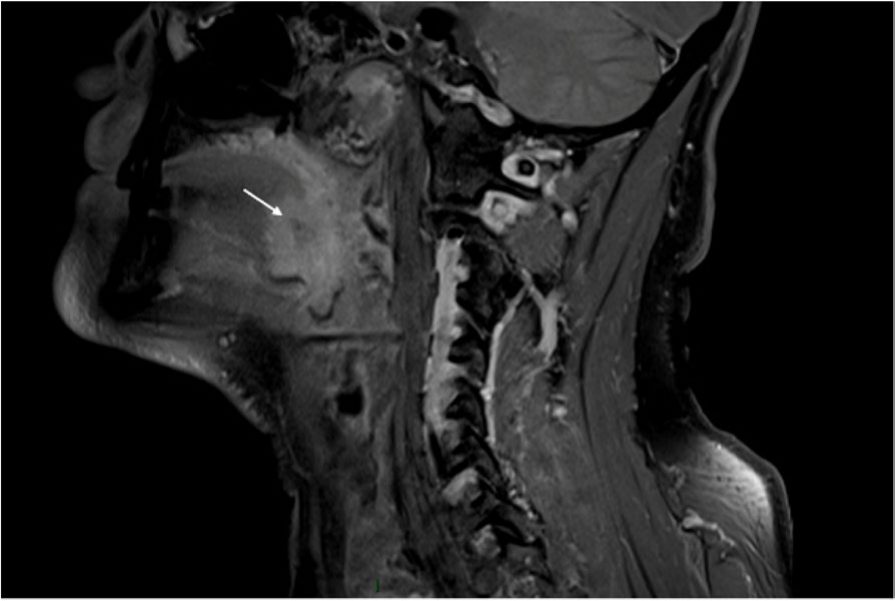
Sagittal T1+C fat sat. llidefine and heterogeneously enhancing mass in the posterior left tongue, infiltrates mylohyoid and genioglossus muscles (white arrow)

**Fig. 2 (Abstract P178):**
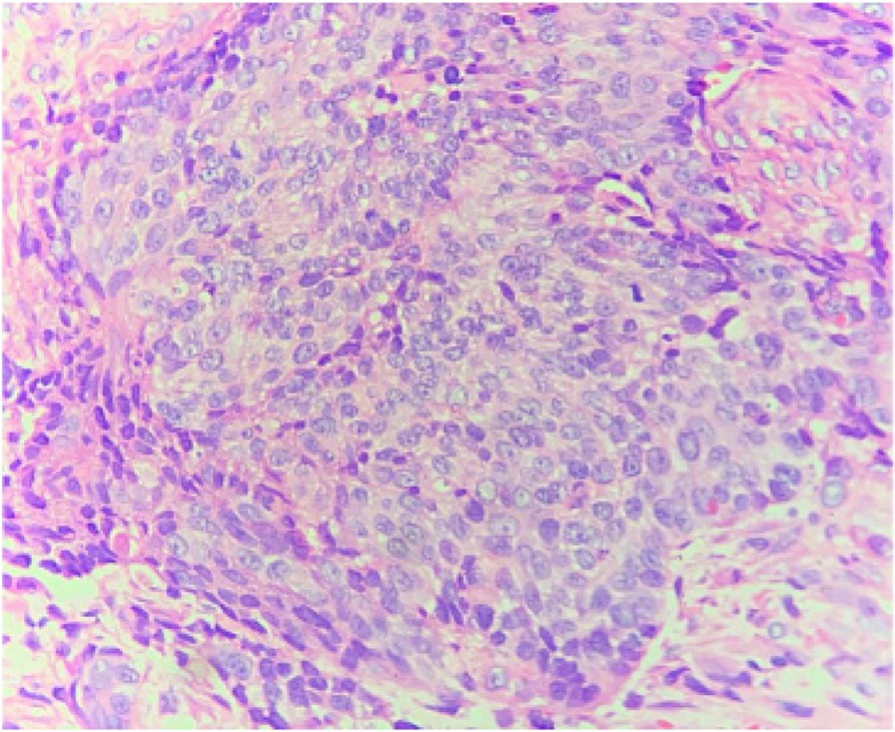
[40X, hematoxylin and eosin (HE)]. Tongue mucosa with Squamos cell carcinoma moderately differentiated consisitingn of large cells, non-keratinizing with lymphatic and perinueral invasion. Grade 2, P16 positive 65%

## P179 Visual snow syndrome in patients with migraine from a tertiary headache center: prevalence, phenomenology and impact on quality of life

### P. Triller^1^, K. S. Lange^1^, P. Kull^1^, J. Mecklenburg^1^, L. H. Overeem^1^, M. Fitzek^1^, A. Siebert^1^, M. Steinicke^1^, J. P. Dreier^1^, D. Kondziella^2,3^, U. Reuter^1,4^, B. Raffaelli^1,5^, L. Neeb^1,6^

#### ^1^Charité – Universitätsmedizin Berlin, Department of Neurology, Berlin, Germany; ^2^Rigshospitalet Glostrup, University of Copenhagen, Department of Neurology, Copenhagen, Denmark; ^3^University of Copenhagen, Department of Clinical Medicine, Copenhagen, Denmark; ^4^Universitätsmedizin Greifswald, Greifswald, Germany; ^5^Berlin Institute of Health at Charité (BIH), Clinician Scientist Program, Berlin, Germany; ^6^Helios Global Health, Berlin, Germany

##### **Correspondence:** P. Triller

*The Journal of Headache and Pain 2024,* **25(Suppl 1)**: P179

**Objective:** Visual snow syndrome (VSS) is a recently classified neurological disorder. Its main symptom is a pan-field visual disturbance described as tiny flickering dots, resembling the static noise of an untuned television. Additional symptoms include palinopsia, entoptic phenomena, photophobia, and nyctalopia. Based on an epidemiological association with migraine aura and hypothesized shared pathomechanisms, the International Classification of Headache Disorders included VSS as a complication of migraine. This study aims to investigate the prevalence and characteristics of VSS among migraine patients of a tertiary headache center.

**Methods:** This analysis is part of a cross-sectional cohort study conducted at a specialized headache center. Migraine patients were asked to complete various questionnaires covering demographic information, headache characteristics, VSS features and the Depression, Anxiety and Stress Scale 21 (DASS-21).

**Results:** Out of 808 migraine patients (mean age 44.4 ± 13.3 years, 87.0% female, 43.7% with migraine with aura), 25 (3.1%, 95% confidence interval (CI), 1.9 – 4.3) met the diagnostic criteria for VSS. The prevalence of migraine aura did not differ significantly between patients with and without VSS (48.0% vs. 43.6%, p=0.813), nor did the type and duration of the aura. Higher DASS-21 scores were associated with VSS (Anxiety *p*<0.01, Depression *p*<0.05, Stress *p*<0.05).

**Conclusion:** In this large study of migraine patients, the prevalence of VSS was unaffected by the presence, type and duration of migraine aura and comparable to the prevalence of population-based studies in the UK. The lack of association between migraine aura and VSS suggests that VSS is an independent medical condition rather than a manifestation of migraine aura.

**Fig. 1 (Abstract P179):**
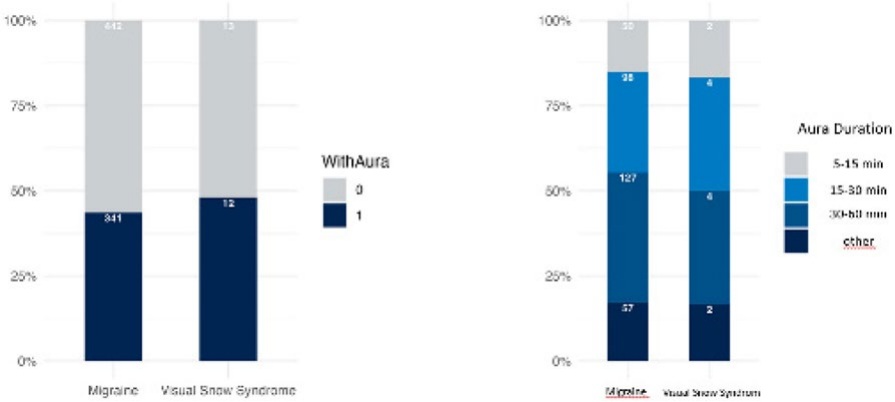
See text for description

**Fig. 2 (Abstract P179):**
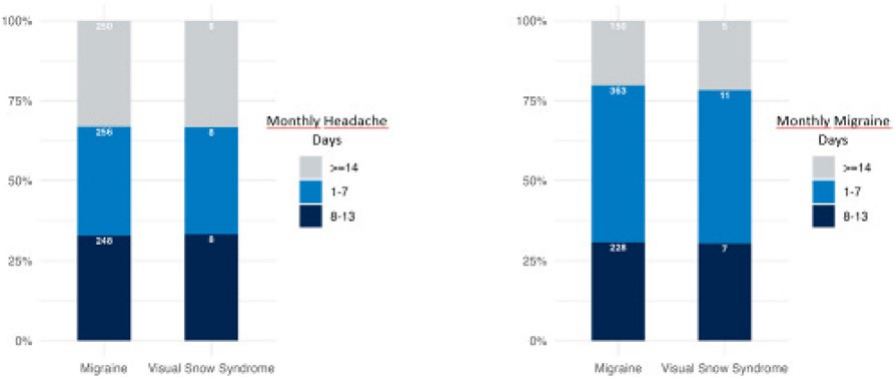
See text for description

**Fig. 3 (Abstract P179):**
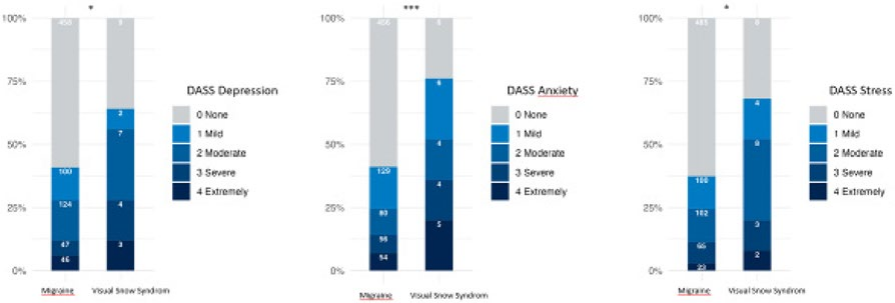
See text for description

## P180 Alice in Wonderland Syndrome (AIWS) and migraine: prevalence and characteristics of AIWS in adults with migraine

### M. Fitzek^1^, J. Mecklenburg^1^, P. Kull^1^, K. S. Lange^1^, L. H. Overeem^1^, A. Siebert^1^, P. Triller^1^, L. Neeb^1,2^, J. P. Dreier^1^, D. Kondziella^1,3^, U. Reuter^1,4^, B. Raffaelli^1,5^

#### ^1^Charité – Universitätsmedizin Berlin, Department of Neurology, Berlin, Germany; ^2^Helios Global Health, Berlin, Germany; ^3^Rigshospitalet Glostrup, University of Copenhagen, Department of Neurology, Copenhagen, Denmark; ^4^Universitätsmedizin Greifswald, Greifswald, Germany; ^5^Berlin Institute of Health at Charité (BIH), Clinician Scientist Program, Berlin, Germany

##### **Correspondence:** M. Fitzek

*The Journal of Headache and Pain 2024,* **25(Suppl 1)**: P180

**Objective:** Alice in Wonderland Syndrome (AIWS) is a rare sensory disorder, characterized by an imbalance between self-perception and surroundings resulting in distorted somatosensory and/or visual perception of the body image or the environment. It also involves distorted time perception and symptoms of derealization/depersonalization. AIWS is frequently reported among individuals with migraine, prompting discussions about its potential recognition as a form of migraine aura. Prevalence, underlying causes and clinical characteristics however are poorly understood. This study aims to investigate the prevalence and features of AIWS in patients with migraine.

**Methods:** The analysis was conducted at a tertiary headache center as part of a prospective cross-sectional cohort study. Migraine patients completed questionnaires that collected information on demographics, headache and AIWS features, and various visual phenomena (e.g. fragmented vision, scotoma, tunnel vision).

**Results:** Out of 808 migraine patients (mean age 44.4 ± 13.3 years, 87% women), a total of 133 (16.5%) reported having experienced AIWS core symptoms at some point in their lives, lasting half an hour on average. The most frequent symptoms were micro- and/or teleopsia (72.9%), followed by macro- and/or microsomatognosia (49.6%) and macro- and/or pelopsia (38.2%). About two-thirds (65.1%) experienced headaches before, after, or during the occurrence of AIWS symptoms. More than half (53.3%) reported their initial AIWS episode happening at an age of 18 years or younger. Patients with AIWS were more likely to be diagnosed with migraine with aura compared to those without AIWS (52% vs. 42%, p = 0.04), and reported a higher occurrence of 16 out of 22 visual phenomena investigated.

**Conclusion:** In this study of migraine patients, AIWS was found to be a common lifetime phenomenon. The association between AIWS and migraine with aura, as well as the similar time course, might suggest shared underlying mechanisms.

## P181 Prospective evaluation of aura during anti-CGRP monoclonal antibodies after 52 weeks of treatment: a case series

### G. Vigani^1^, M. Romozzi^2^, A. Burgalassi^1^, G. Tabasso^1^, F. De Cesaris^1^, C. Vollono^2^, P. Calabresi^2^, A. Chiarugi^1^, P. Geppetti^1^, L. F. Iannone^1^

#### ^1^University of Florence, Health Sciences, Florence, Italy; ^2^University Cattolica del Sacro Cuore, Rome, Italy

##### **Correspondence:** G. Vigani

*The Journal of Headache and Pain 2024,* **25(Suppl 1)**: P181

**Objective:** To assess changes of aura episodes and their relation with the onset of headache during prolonged long anti-CGRP mAb treatment.

**Methods:** We evaluated the entire cohort of patients treated with anti-CGRP mAbs in a tertiary Headache Center. All patients who experienced at least one episode of aura per month, as reported in their medical history and during the baseline period were included. The presence of attacks with aura was carefully monitored at baseline and in the last 3 months of treatment.

**Results:** We analyzed data from 13 patients who provided complete information on aura. Among them, 9 were females (69.2%), 12 had chronic migraine (92.3%), and 12 had MO (92.3%) at baseline. Twelve patients received a diagnosis of both migraine without (MwoA) and with aura (MwA) for 12 patients, and one patient only MwA. The mean duration from the onset of first aura episode was 17.8±7.9 years, with an mean duration of aura episodes of 34.2±15.7 minutes. Nine patients (69.2%) reported visual aura, while 4 patients (30.8%) experienced both visual and sensory aura. At baseline, the average number of MHDs was 22.3±7.5, with 9.08±9.1 with aura. After 12 months of treatment, the number of d MHDs with aura in the last 3 months of treatment was stably reduced to 2.6±2. All patients, except one, reported a consistent reduction in aura episodes throughout therapy. Three patients reported episodes of aura without subsequent headache, a phenomenon that was not present prior to anti-CGRP treatment.

**Conclusion:** Anti-CGRP mAbs reduced the frequency, intensity, and duration of aura consistently with the reduction of MHDs. Only one patient reported episodes of aura without subsequent headache.

## P182 Factors contributing to the pain lateralization in migraine

### H. C. Lee^1^, S. Cho^2^, B. K. Kim^1^

#### ^1^Nowon Eulji Medical Center, Eulji University School of Medicine, Department of Neurology, Seoul, South Korea; ^2^Uijeongbu Eulji Medical Center, Eulji University School of Medicine, Department of Neurology, Uijeongbu, South Korea

##### **Correspondence:** H. C. Lee

*The Journal of Headache and Pain 2024,* **25(Suppl 1)**: P182

**Objective:** Recent studies have suggested right-side dominance in migraine pain and possible linking handedness and psychiatric comorbidity to pain lateralization. The objective of our study was to investigate the frequency of unilateral headaches and identify the factors contributing to pain lateralization.

**Methods:** In this prospective study, we examined the relationship between pain lateralization and demographic data, handedness, and comorbid psychiatric illness in patients with migraine. Handedness was determined using the Edinburgh handedness inventory.

**Results:** Among the 430 patients included in the study, 84.9% were female. The age range of the participants was 19-81 years, with 88.4% being younger than 60 years old. The duration of the disease ranged from 1 to 55 years, with a mean of 22.7 years. The majority of the patients (89.8%) were right-handed. Comorbidity with depression and anxiety was observed in 58.3% and 47.2% of the patients, respectively. Out of the total participants, 49.8% (214 patients) reported experiencing unilateral pain. Among these cases, 42.5% had right-sided pain, 41.6% had left-sided pain, and 15.9% reported unilateral pain without any specific side predominance. The absence of depression was associated with a unilateral headache (p=0.03). There were no statistically significant differences observed in terms of pain lateralization when considering sex, age, handedness, presence of chronic migraine, and psychiatric comorbidities.

**Conclusion:** Our study suggests that there is no dominant side for migraine pain, challenging the notion of right-side predominance. Additionally, our findings indicate that handedness and psychiatric comorbidity do not seem to be significantly associated with pain lateralization.

## P183 Clinical characteristics of migraine with and without aura: a comparative study

### M. Togha^1^, O. Kohandel Gargari^1,2^, S. Nematgorgani^3^, R. Rasekh Magham^3^, Z. S. Ahmadi^3^

#### ^1^Iranian Center of Neurological Research, Neuroscience Institute, Headache Department, Tehran, Iran; ^2^Tehran University of Medical Sciences, Headache Research center, Tehran, Iran; ^3^Shahid Beheshti University of Medical Sciences, Department of Clinical Nutrition and Dietetics, Tehran, Iran

##### **Correspondence:** O. Kohandel Gargari

*The Journal of Headache and Pain 2024,* **25(Suppl 1)**: P183

**Objective:** The objective of this study was to compare the demographic features and clinical characteristics of headaches in patients diagnosed with migraine with aura and migraine without aura. Additionally, the association between a history of minor head trauma with aura and headache frequency was explored.

**Methods:** Patients were enrolled based on their diagnosis of migraine, following the criteria outlined in the International Classification of Headache Disorders (ICHD-3). Detailed headache characteristics were assessed using a one-month headache survey. Medical history, including the presence of comorbidities such as GI upsets, hypertension, diabetes mellitus, and hypothyroidism, and a history of head trauma was documented. Laboratory tests were conducted to screen for hypothyroidism and diabetes mellitus. Patients were categorized into two groups based on the presence of aura. To compare the variables the chi-square test, Fisher exact test, and independent T-test through the SPSS software version 26.0 were used. A significance level of 0.05 was used to determine statistical significance.

**Results:** Finally, a total of 391 patients were selected, including 338 cases of migraine without aura and 53 cases of migraine with aura. Most patients were female in both groups (about 80% in each group). The mean age was 35 ± 11 years in both groups. There was no significant difference in age or gender between both groups. Patients experienced about 12 ± 10 headache days a month without significant intergroup differences. Constipation (OR=0.3, *P*<0.05) and dyspepsia (OR=0.4, *P*<0.05) were significantly more frequent among patients with migraine without aura. Other comorbidities including hypertension, hypothyroidism, diabetes mellitus, and sleep problems were not significantly different between groups. We found no significant association between a history of minor head trauma and differences in the presence of aura or headache frequency.

**Conclusion:** In conclusion, our study revealed only a significantly higher occurrence of GI problems including constipation and dyspepsia among patients diagnosed with migraine without aura, while no other significant differences were found between the two migraine types.

## P184 Migraine in childhood: gender differences

### F. Ursitti^1^, L. Papetti^1^, G. Sforza^1^, M. A. N. Ferilli^1^, G. Monte^1^, A. Voci^1^, R. Moavero^1,2^, M. Valeriani^1,3^

#### ^1^Bambino Gesù Children's Hospital, Developmental Neurology Unit, Rome, Italy; ^2^Tor Vergata University of Rome, Child Neurology Unit, Systems Medicine Department, Rome, Italy; ^3^Aalborg University, Center for Sensory-Motor Interaction, Denmark Neurology Unit, Aalborg, Denmark

##### **Correspondence:** F. Ursitti

*The Journal of Headache and Pain 2024,* **25(Suppl 1)**: P184

**Objective:** Migraine involves up to 20% of children and adolescents. Although gender differences in migraine epidemiology and clinical characteristics have been largely investigated in adulthood, this issue is less known in children. We aim at providing an overview of gender differences in pediatric migraine.

**Methods:** The most recent literature was reviewed taking into account epidemiological, pathophysiological, and clinical differences between boys and girls with migraine.

**Results:** All the reviewed literature suggests that gender differences in migraine are not only characteristic of adulthood or post-pubertal adolescents, but they involve younger children. Epidemiological differences between genders depend typically on age, with an inversion of the male/female ratio during and after puberty. Indeed, while before puberty migraine prevalence is slightly higher in boys than in girls, the disease becomes more frequent in post-pubertal females. Also clinical characteristics of the migraine attack can be different between males and females. Hormonal differences can account for many gender differences, especially after puberty, but other factors may be important to explain the different development trajectories of migraine in girls and boys. Promising results are those issued from genetic studies, which are trying to interpret some gender differences in terms of different genotypes. Also neuroimaging investigation is discovering gender differences in the brain cortex structure.

**Conclusiun:** Different aspects of childhood migraine may vary depending on gender and age, especially with regard to pubertal development. Future research should investigate: 1) genetic-clinical correlations in males and females; 2) correlation between migraine features and hormonal levels (estrogens and testosterone); 3) possible gender differences in the response to treatments; 4) possible gender differences in the migraine equivalents, which represent the early symptoms of the childhood migraine.

## P185 Effect of smoking on the development of migraine in women: a nationwide cohort study

### S. A. Kim^1^, M. J. Lee^1^, K. Han^2^

#### ^1^Seoul National University Hospital, Neurology, Seoul, South Korea; ^2^Soongsil University, Department of Statistics and Actuarial Science, Seoul, South Korea

##### **Correspondence:** S. A. Kim

*The Journal of Headache and Pain 2024,* **25(Suppl 1)**: P185

**Objective:** In this study, we aimed to investigate the effect of smoking on the incidence of migraine in women and effect modification of menopause.

**Methods:** Using a nationally representative National Health Insurance Service data, women aged ≥40 years who participated national breast cancer screening in 2009 were followed up until the end of 2018. Participants were classified based on their smoking status: non-smoker, ex-smoker, and current smoker. Information regarding the duration and amount of smoking were also collected. A Cox proportional hazard regression model was used to assess the independent effect of smoking on the risk of incident migraine. The model was adjusted for age, household income, body mass index, hypertension, diabetes mellitus, chronic kidney disease, dyslipidemia, alcohol assumption, regular physical activity, age at menarche, parity, breast feeding, and oral contraceptives and stratified by menopause (premenopause vs. postmenopause). The interaction between smoking and menopause was also investigated.

**Results:** A total of 1,827,129 women were included in the analysis. In both premenopausal and postmenopausal women, current smoking increased the risk of incident migraine compared to never smoking. However, the effect was greater in premenopausal women (adjusted HR 1.140 [95% CI 1.108–1.172]) than in postmenopausal women (adjusted HR 1.045 [95% CI 1.018–1.073]) (p for interaction <0.0001). The risk increased with increased amount of smoking in both premenopausal and postmenopausal, with greater association in premenopausal women (p for interaction <0.0001). Past smoking increased the risk of incident migraine only in premenopausal women (adjusted HR 1.055 [95% CI 1.011–1.100]).

**Conclusion:** Smoking increases the development of migraine attack in women, and its effect is influenced by menopausal factors. Interaction between smoking and estrogen may increase the vulnerability of migraine brain.

**Fig. 1 (Abstract P185):**
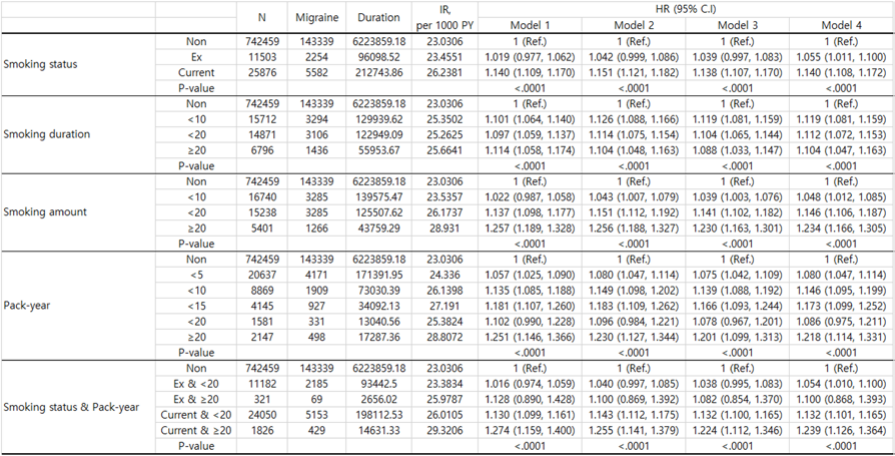
See text for description

**Fig. 2 (Abstract P185):**
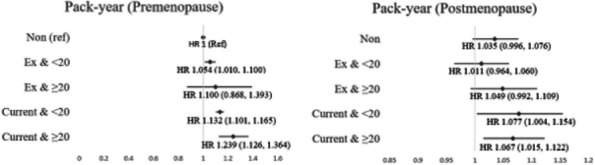
See text for description

**Fig. 3 (Abstract P185):**
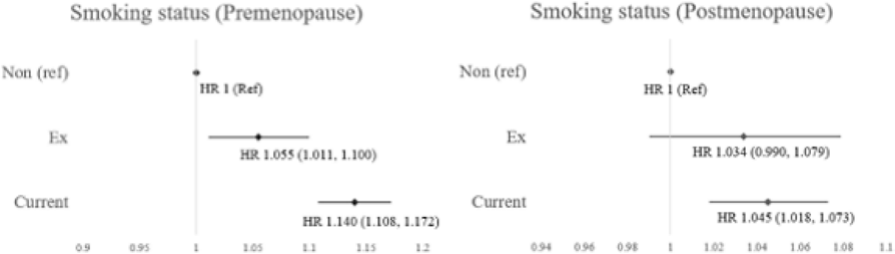
See text for description

## P186 Sex differences of aggravating factors of migraine: a clinic-based multicenter study

### N. Imai^1^, T. Takizawa^2^, N. Watanabe^2^, Y. Matsumori^3^

#### ^1^Japanese Red Cross Shizuoka Hospital, Department of Neurology and Headache Center, Shizuoka, Japan; ^2^Keio University School of Medicine, Department of Neurology, Tokyo, Japan; ^3^Sendai Headache and Neurology Clinic, Sendai, Japan

##### **Correspondence:** N. Imai

*The Journal of Headache and Pain 2024,* **25(Suppl 1)**: P186

**Objective:** Migraine is aggravated by several factors. Menstruation is an important aggravating factor in women, and there are several other aggravating factors common to men and women. We aimed to investigate gender differences in aggravating factors and to categorise aggravating factors using factor analysis.

**Methods:** A total of 1868 migraineurs (72.2% women; mean age at first consultation: 33.4 ± 13.6 years) who visited 3 headache education centres accredited by the Japanese Headache Society between March 2021 and March 2022. Aggravating factors, including bathing or warming up, head shaking, body movement, drinking alcohol, smoking, lack of sleep, sleeping too much, worsening weather, bright light, noise, smell, cold, eating cold food, eating spicy food, crowding, holidays, lack of sleep, and overwork, were collected using the questionnaire. We used factor analysis to categorise the aggravating factors.

**Results:** The most common aggravating factor was worsening weather, which accounted for 49.0%, followed by shaking the head at 45.8%, moving the body at 36.0%, lack of sleep at 33.7%, and overwork at 33.4%. Women had significantly higher rates of worsening weather, moving the body, lack of sleep, overwork, bright light, noise, crowding, sleeping too much, smell, bathing or warming up, and holidays, and significantly lower rates of smoking and eating spicy food (Table 1). Factor analysis estimated 5 common factors: Factor 1, noise, bright light, smell, crowd and worsening weather; Factor 2, moving the body, shaking the head and bathing or warming up; Factor 3, drinking alcohol, sleeping too much, lack of sleep, overwork, smoking and holidays; Factor 4, cold and eating cold food; Factor 5, eating spicy food than men. (Table 2).

**Conclusion:** Our study showed that the most common aggravating factor was worsening weather and that women had more aggravating factors than men. Worsening weather fell into the same category as noise, bright light, smell, which seemed to be hypersensitivity symptoms.

**Table 1 (Abstract P186):**
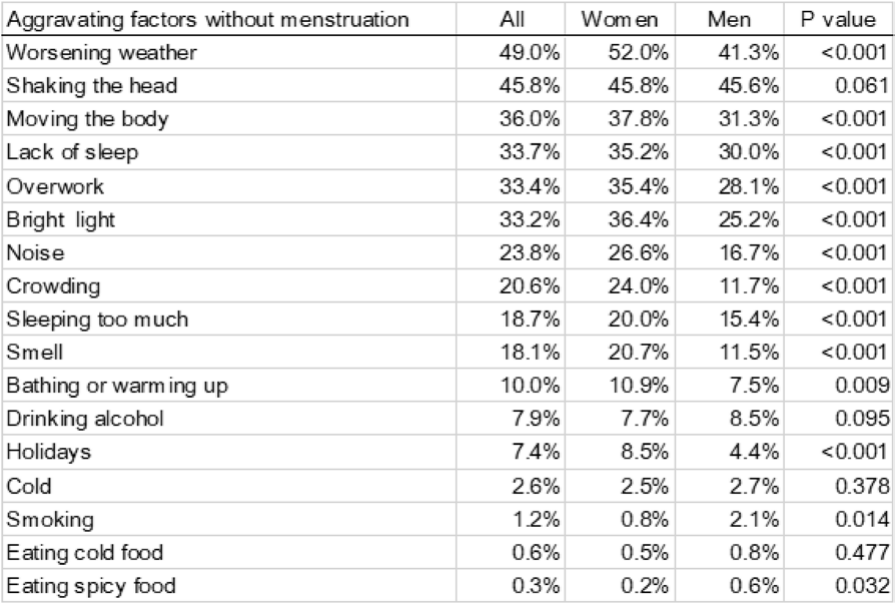
See text for description

**Table 2 (Abstract P186):**
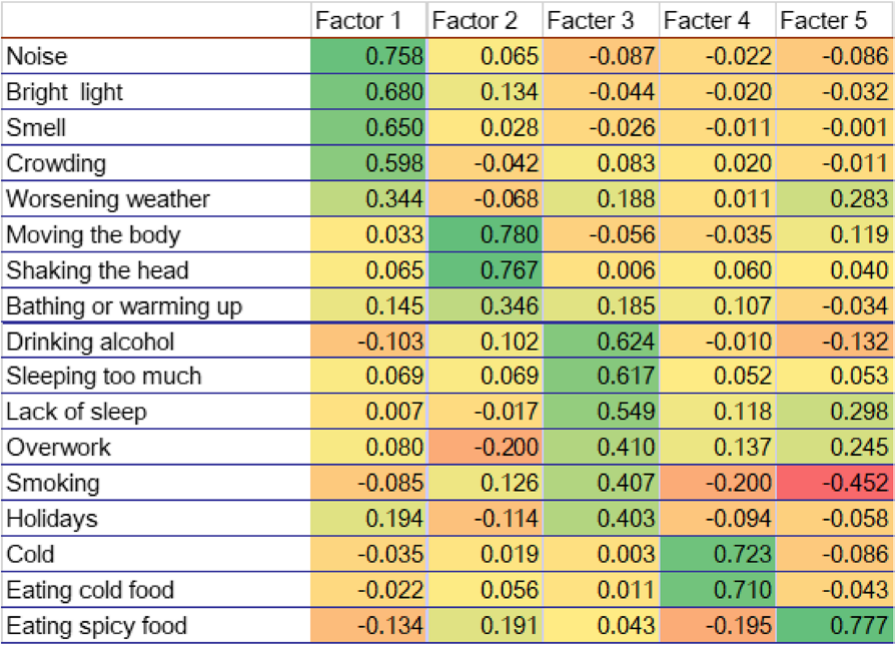
See text for description

## P187 Migraine is associated with female sex and painful temporomandibular disorders among adolescents: an epidemiological study

### D. A. Godoi Gonçalves, L. B.Campi, P. Jordani, G. V.V.Braido, G. Fernandes

#### Sao Paulo State University (Unesp), School of Dentistry, Araraquara, Dental Materials and Prosthodontics, Araraquara, Brazil

##### **Correspondence:** D. A. Godoi Gonçalves

*The Journal of Headache and Pain 2024,* **25(Suppl 1)**: P187

**Objective:** Among primary headaches (PH), migraine is the type more frequently associated with temporomandibular disorders (TMD), establishing a comorbid relationship. In adults, both conditions are more prevalent among females than males. Herein we investigated the association of painful TMD with PH. We also investigated the participation of gender, and other variables in this association.

**Methods:** We assessed the presence of painful TMD (Research Diagnostic Criteria for TMD) and PH (International Classification for Headache Disorders – 2nd Edition) in a non-representative sample of adolescents (12-14 years old). We also assessed the TMD pain intensity, sedentarism, symptoms of depression, sleep quality, and the stage of pubertal development. To characterize the sample, descriptive statistics were performed. The sample was stratified by gender for the study of the associations with TMD and PH. For comparison of proportions, the Chi-Squared test was performed. Linear regression models were used to compare adolescents with painful TMD, migraine, PM, and TTH, and to test the influence of other variables in these associations.

**Results:** The sample consisted of 690 individuals, mean age of 12.7 years, of whom 56.4% were girls. Of the total, 16.2% presented painful TMD and 37.4% presented PH. Migraine was the most frequent type identified in 40.7% of the volunteers. We found a significant association between female sex and migraine (*p*<0.0001) but not with painful TMD (p=0.065). The association between painful TMD and migraine was significant (*p*<0.001) and was not altered by sex. The linear regression analyses showed that the TMD pain intensity (*p*<0.001) and economic classification (*p*=0.007) contributed to the association between painful TMD and migraine.

**Conclusion:** Migraine was the most frequent type of PH among adolescents and was significantly associated with female sex and painful TMD. Adolescents with both painful TMD and migraine showed higher TMD pain intensity.

## P188 The epidemiology and unmet need of diagnosed migraine respondents using the national health and wellness survey in five european countries

### J. Brown^1^, S. Drakeley^2^, A. Mercadante^2^, A. Jenkins^1^, K. Hygge Blakeman^1^, A. Gendolla^3^

#### ^1^Pfizer, Inc., New York, NY, United States; ^2^Cerner Enviza, Kansas City, MO, United States; ^3^Praxis Gendolla, Essen, Germany

##### **Correspondence:** J. Brown

*The Journal of Headache and Pain 2024,* **25(Suppl 1)**: P188

**Objective:** This study examined the prevalence and burden of migraine in Europe by collecting data from the National Health and Wellness Survey in five European countries: France, Germany, UK, Italy, and Spain.

**Methods:** A retrospective cross-sectional study using the 2020 EU National Health and Wellness Survey (NHWS, Cerner Enviza) captured patient-reported data from respondents with migraine. A self-reported physician diagnosis of migraine was required for the respondents to be included in the migraine cohort. Demographic, health, and clinical characteristics were summarized by weighted descriptive statistics.

**Results:** There was an estimated 30.5 million adults with diagnosed migraine and a weighted prevalence of 11.5% in the 5 countries. Migraine prevalence peaked in respondents between ages 18 and 49 years, with 62.5% of individuals diagnosed with migraine within this age group and a mean age of 44.4 years. Among all those with diagnosed migraine, 66.4% were female, and 31.8% of females reported their migraines were associated with their menstrual cycle. Individuals in the migraine cohort reported a mean of 3.1 ± 4.8 migraine days and 6.2 ± 6.2 headache days in the past 30 days. Migraine disability measured via MIDAS showed 32% of people with migraine reported mild or moderate disability, and 24% reported severe disability. Regarding medication use, 29% reported using over-the-counter (OTC) medication only, 27% reported using prescription (Rx) only, and 24% used both Rx and OTC. The majority (73%) reported using medications typically indicated for acute migraine treatment. Based on current utilization rates of acute migraine treatments, 13.8% of all people with migraine were at risk for medication overuse.

**Conclusion:** Migraines impact nearly 12% of the adult population in Europe, with disproportionate effects on women and those in their 30s and 40s. Among all people with migraine, 80% are currently treated with either Rx or OTC medications with a high rate of medication overuse. Novel treatment options may help alleviate the high overall burden and unmet need in people with migraine.

## P189 Comparison of healthcare resource utilization, productivity, and quality of life in people with migraine versus without migraine among national health and wellness survey respondents in Europe

### J. Brown^1^, S. Drakeley^2^, A. Mercadante^2^, N. Sternbach^2^, K. Hygge Blakeman^1^, A. Jenkins^1^, A. Gendolla^3^

#### ^1^Pfizer, Inc., New York, NY, United States; ^2^Cerner Enviza, Kansas City, MO, United States; ^3^Praxis Gendolla, Essen, Germany

##### **Correspondence:** J. Brown

*The Journal of Headache and Pain 2024,* **25(Suppl 1)**: P189

**Objective:** This study evaluated patient-reported outcomes from people with migraine compared to those without migraine to examine Healthcare Resource Utilization (HCRU), Quality of Life (QoL), and work productivity and activity impairment (WPAI) in respondents in five European countries (5EU).

**Methods:** A retrospective cross-sectional study of 62,319 individuals from the 2020 5EU National Health and Wellness Survey (NHWS, Cerner Enviza) captured patient-reported data from migraine respondents who resided in five European countries (Germany, France, UK, Spain, Italy). Matching was conducted by generating a propensity score of having migraine vs. no migraine based on 11 demographic and clinical characteristics in a 1:2 (case:control) ratio. Demographic, health, and clinical characteristics were summarized by descriptive statistics (means and standard deviations for continuous measures and counts and percentages for categorical variables).

**Results:** A total of 51,768 respondents were eligible for analyses, including n=3,985 with diagnosed migraine and n=47,783 with no migraine. Analyses revealed significant differences (all *p*<0.001) between the matched cohort with migraine versus those with no migraine in WPAI, QoL, and HCRU. The average number of ER visits in the past six months for diagnosed migraine respondents (0.64 SD 1.88) was twice that of the no-migraine group (0.33, SD 1.15). The mean number of hospitalization visits in the past six months was also significantly different in both groups 0.32 (SD 1.90) and 0.22 (SD 1.44) for migraine and no migraine groups. Among the migraine group, 12% had a neurologist visit in the past 6 months compared to only 4% of the no migraine group.

**Conclusion:** After matching for demographic and clinical variables, diagnosed migraine individuals in Europe had significantly higher work impairment, lower QoL (both mental and physical), and greater HCRU compared to people without migraine. Addressing the burden of migraine and current unmet need may have tremendous impacts on patient"s QoL, productivity, and HCRU as well as direct and indirect costs from the patient, payor, and societal perspectives.

## P190 Migraine and thyroid disease – the relationship between Hashimoto's thyroidits, migraine severity and female sex

### M. Nowaczewska^1^, M. Straburzyński^2^, G. Meder^3^, M. Waliszewska-Prosół^4^

#### ^1^Collegium Medicum of Nicolaus Copernicus University, Bydgoszcz, Poland; ^2^University of Warmia and Mazury, Department of Family Medicine and Infectious Diseases, Olsztyn, Poland; ^3^Jan Biziel University Hospital No. 2, Department of Interventional Radiology, Bydgoszcz, Poland; ^4^Wrocław Medical University, Department of Neurology, Wrocław, Poland

##### **Correspondence:** M. Nowaczewska

*The Journal of Headache and Pain 2024,* **25(Suppl 1)**: P190

**Objective:** Migraine has been linked with several comorbidities including thyroid diseases, especially hypothyroidisms. Although Hashimoto's thyroiditis (HT) is nowadays the leading cause of hypothyroidism with high and still growing prevalence in general population, there are lack of data regarding migraine and HT connection. The aim of this study was to answer the questions: What is the prevalence of HT in migraine sufferers? Does the presence of HT influence migraine severity?

**Methods:** This retrospective observational cohort study involved consecutive migraine patients consulted at our Headache Centre with diagnosis of migraine. Electronic charts of migraineurs were collected, including data on migraine type, duration of disease, presence of cranial autonomic symptoms (CAS), monthly migraine days (MMD), medication overuse headache (MOH), and the presence of comorbidities including HT.

**Results:** We found 928 eligible migraine patients, 88.7% were women. The mean age was 36,09 years. 592 (63.8%) were diagnosed with episodic migraine, 336 (36,2%) with chronic migraine (CM), 156 (16,8%) had migraine with aura. MOH was additionally diagnosed in 258 (27,8%) patients. The duration of disease was 15,99 years. 106 (11.4%) was diagnosed with HT, 148 (15.9%) with hypothyroidisms, while 84 (9,05%) had both. Migraine patients with HT were significantly older (*p*<0.001), were more frequently women (*p*=0.0017), had longer duration of disease (*p*<0.001), had CAS more frequently (<0.001), develop chronic migraine (*p*=0.0169) and depression more frequently (*p*=0.0047) and had more MMD (*p*=0.0195) as compared with individuals without HT. According to our multivariate logistic model, the presence chronic migraine was positively associated with Hashimoto thyroiditis (OR 1.76, *p*=0.045). All patients with CM and HT were woman, as compared with CM without HT group ( 100% vs 89.2%, *p*>0.0075).

**Conclusion:** Thyroid diseases, including HT is very prevalent in migraine patients; more prevalent than in the general population. This is the first study considering migraine and HT to be comorbid and suggesting that HT may influence the course of migraine causing its chronification, but only in woman.

## P192 Epidemiology, disability and treatment satisfaction of physician-diagnosed migraine respondents using the National health and wellness survey in Spain

### D. García Azorín^1^, C. Moya-Alarcón^2^, B. Armada^2^, M. Sánchez del Río^3^

#### ^1^Hospital Clínico Universitario de Valladolid, Headache Unit, Department of Neurology, Valladolid, Spain; ^2^Pfizer S.L.U., Alcobendas, Spain; ^3^Clínica Universidad de Navarra, Department of Neurology, Madrid, Spain

##### **Correspondence:** García Azorín, M. Sánchez del Río

*The Journal of Headache and Pain 2024,* **25(Suppl 1)**: P192

**Objective:** The current study aimed to assess data on the prevalence and burden of migraine in Spain by analyzing data from the 2020 National Health and Wellness Survey (NHWS).

**Methods:** A cross-sectional study using NHWS 2020 (Cerner, Enviza) captured data reported by migraine patients from a representative sample of the Spanish adult population. Demographic, health and clinical characteristics were summarized by weighted descriptive statistics. Migraine burden measures included monthly migraine days (MMD) and monthly headache days (MHD) in the preceding 30 days, disability (assessed with the MIDAS scale), current treatments for migraine, treatment satisfaction and medication overuse risk.

**Results:** A total of 7,074 respondents completed the NHWS survey. Among which, 1,584 (22.4%) self-reported having migraine and 1,020 (65.4%) of those reported having a physician diagnosis and were included in the analysis. This represented a weighted prevalence of 14.0% in Spain (Figure 1A). Migraine patients had a mean age of 43.5 years, 63.7% were female and reported mean MMD 2.9±4.3 and MHD 6.3±5.9, with 75.7% of patients reporting less than 4 MMD (Figure 1B). MIDAS scores corresponded to severe disability in 23.2% and mild-to-moderate severity in 39.1% patients. Regarding treatment, 96.2% reported using prescribed medication, 91.7% acute and 8.3% preventive treatment.

The percentage of patients who reported not being satisfied with the treatment corresponded to 28.4% with acute drugs (*N*=1,267) and 30.7% with preventive drugs (*N*=81), being triptans (*N*=219) the treatment with lower satisfaction rate (33.6%) (Figure 2). Based on utilization rates of acute treatments, 16.9% of patients were at risk of medication overuse.

**Conclusion:** One in seven Spanish citizens suffers from migraine, most of them with low-frequency episodic migraine. However, 62% patients showed significant disability and almost a third of patients were dissatisfied with their current treatment.

**Fig. 1 (Abstract P192):**
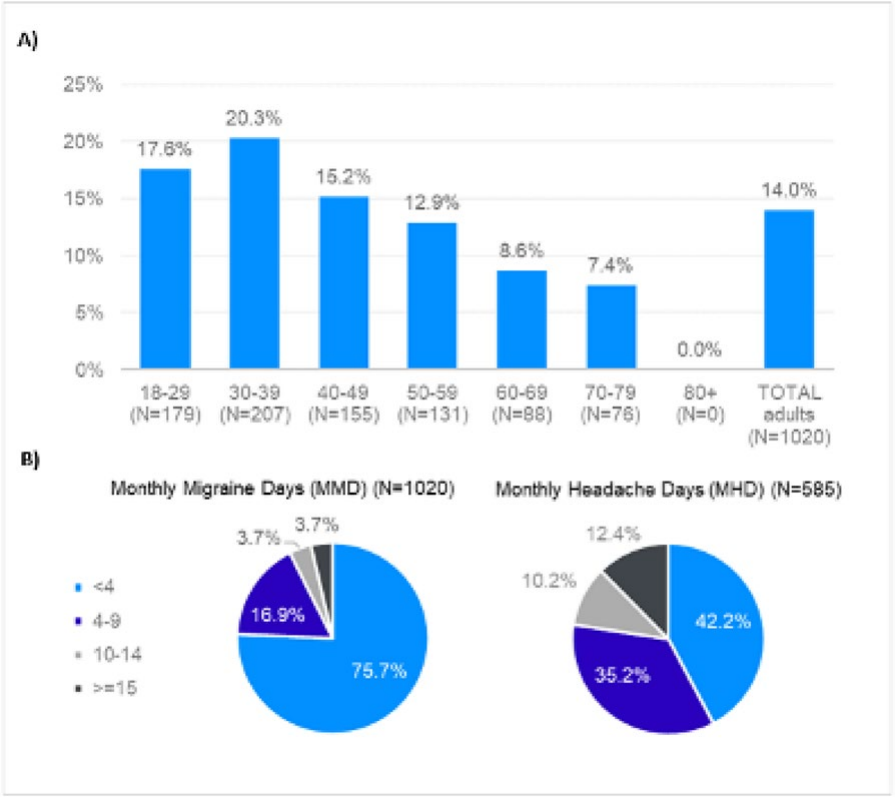
Spanish migraine prevalence (A) and monthly migraine or headachen days (B) among adults self-reported physician diagnosed by age group in the 2020 NHWS

**Fig. 2 (Abstract P192):**
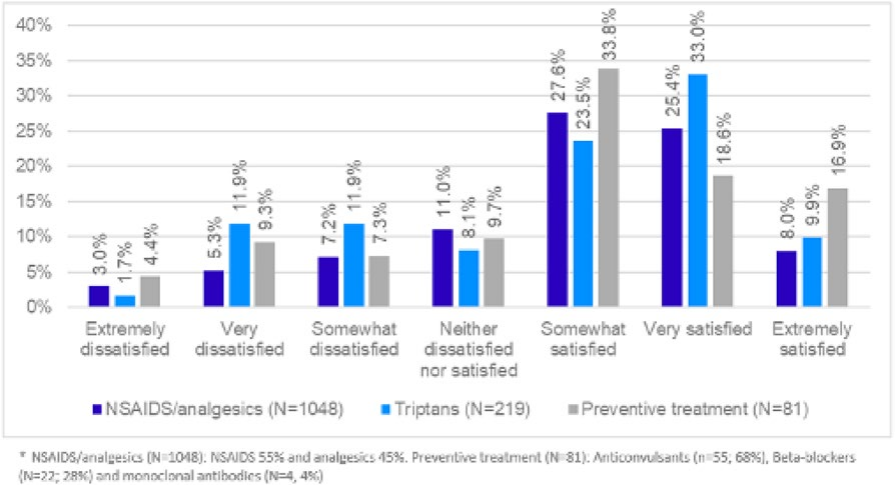
Treatment satisfaction among adults self reported physician diagnosed in the 2020 NHWS

## P193 Brain perfusion magnetic resonance during persistent sensorimotor aura in a case of familial hemiplegic migraine due to PRRT2 gene deletion: the first case report

### G. Garascia^1^, A. Granato^1^, L. Bartole^1^, M. Ukmar^2^, F. Degrassi^2^, P. Manganotti^1^

#### ^1^Cattinara University Hospital, ASUGI, University of Trieste, Neurology Unit, Headache Centre, Department of Medical, Surgical and Health Sciences, Trieste, Italy; ^2^Cattinara University Hospital, ASUGI, University of Trieste, Radiology Unit, Department of Medical, Surgical and Health Sciences, Trieste, Italy

##### **Correspondence:** G. Garascia

*The Journal of Headache and Pain 2024,* **25(Suppl 1)**: P193

**Objective:** Migraine aura (MA) can mimic a stroke. This is particularly true for Familial Hemiplegic Migraine (FHM), in which MA stroke-like symptoms can last up to weeks. Perfusion-weighted brain imaging technique can help to discriminate MA from stroke. Nevertheless, few studies have been conducted with perfusion Magnetic Resonance Imaging (pMRI) in FHM. Aim of this study is to analyse a pMRI performed during a unique case of persistent FHM sensorimotor aura due to PRRT2 gene deletion.

**Methods:** Two different qualitative and quantitative brain MRI assessments were done in a 27-year-old affected by FHM. The first one was conducted during MA symptoms (T0), consisting in right limbs weakness and hypoesthesia lasting for several weeks. The second one was performed after some months of an antiepileptic treatment effective on MA symptoms (T1). The imaging was acquired and evaluated by an expert neuroradiologist.

**Results:** Both at T0 and at T1, standard MRI sequences were previously scanned and did not show particular relieves. Then, perfusion sequences with Mean Transit Times (MTT), Time To Peak (TTP), Cerebral Blood Volume (CBV) and Flow (CBF) maps were acquired. Therefore, two different Regions Of Interests (ROI) in brain contralateral basal ganglia areas were posed. By a quantitative analysis, a left basal ganglia hypoperfusion was detected at T0, on the side corresponding to MA symptoms. At T1, during the antiepileptic treatment, a reduction of the aforementioned hypoperfusion was observed. A simply qualitative analysis of both the exams did not permit to detect significant asymmetries of brain perfusion, instead.

**Conclusion:** To date, this is the first study reporting the results of a pMRI performed during symptoms of a unique sensorimotor persistent MA in a case of FHM due to PRRT2 gene deletion, showing a brain hypoperfusion on the side corresponding to MA symptoms, reduced by a clinically impactful antiepileptic treatment.

*Disclosure statement*: Informed consent to publish this case study and its potentially identifiable information of the patient was obtained from the individual involved. The patient gave explicit permission for the publication of this case report, including any relevant clinical details.

## P194 Allodynia in migraineurs with idiopathic epilepsy is associated with headache and seizure triggers

### E. Ekizoğlu^1^, B. Baykan^1^, A. Ç. Atalar^2^, B. G. Türk^3^, D. Kurt Gök^4^, P. Topaloglu^5^, A. Özge^6^, S. Ayta^7^, F. F. Erdoğan^4^, S. N. Yeni^3^, B. Taşdelen^8^, I. Study Group^9^, S. K. Velioglu^10^

#### ^1^Istanbul University, Istanbul Faculty of Medicine, Neurology, İstanbul, Turkey; ^2^Health Sciences University, Kanuni Sultan Süleyman Education and Research Hospital, Neurology, İstanbul, Turkey; ^3^Istanbul University-Cerrahpasa, Faculty of Medicine, Neurology and Clinical Neurophysiology, İstanbul, Turkey; ^4^Erciyes University, Faculty of Medicine, Neurology and Clinical Neurophysiology, Kayseri, Turkey; ^5^Istanbul University, Istanbul Faculty of Medicine, Neurology and Child Neurology, İstanbul, Turkey; ^6^Mersin University Medical Faculty, Neurology, Algology and Clinical Neurophysiology, Mersin, Turkey; ^7^Spastic Children’s Foundation of Turkey, İstanbul, Turkey; ^8^Mersin University Medical Faculty, Biostatistics and Medical Informatics, Mersin, Turkey; ^9^Multicenter Study Group, Turkey, Turkey; ^10^Karadeniz Technical University, Faculty of Medicine, Neurology and Clinical Neurophysiology, Trabzon, Turkey

##### **Correspondence:** E. Ekizoğlu

*The Journal of Headache and Pain 2024,* **25(Suppl 1)**: P194

**Objective:** The aim of this multi-center study was to investigate whether allodynia is associated with headache triggers or seizure triggers, in patients with comorbid idiopathic/genetic epilepsies (I/GE) and migraine.

**Methods:** The data were collected from a cross-sectional large study, using two structured questionnaires for headache and epilepsy features, fulfilled by neurologists. Allodynia Symptom Checklist (ASC-12) was used to detect allodynia. We compared demographic and clinical differences between two groups of adult patients with and without allodynia statistically, and used ROC curves to determine a threshold of the total number of headache and seizure triggers associated with the presence of allodynia.

**Results:** Among 298 (229 females, 76.8%) patients with I/GE and migraine with a mean age of 28.2± 7.9 years, 53 (17.8%) patients reported allodynia. Allodynia was rated as mild in 26, moderate in 16 and severe in 11 patients according to results of ASC-12. Patients with allodynia had higher number of headache triggers and seizure triggers in comparison to those without allodynia (*p*<0.000 for both) in association with the severity of allodynia. Moreover, ROC curves showed that allodynia was associated with having ≥3 seizure triggers (AUC:0.679; 95%CI: 0.602-0.755; *p*<0.000), and ≥9 headache triggers (AUC:0.763; 95%CI: 0.698-0.828; *p*<0.000).

**Conclusion:** Our findings showed that allodynia reflecting central sensitization is associated with a higher number of triggers for both headache and seizure, suggesting a more prominent hyperexcitability in migraineurs with I/GEs. Allodynia should be evaluated in patients with comorbid I/GEs and migraine in the routine clinical practice to achieve a better management of these diseases.

**Fig. 1 (Abstract P194):**
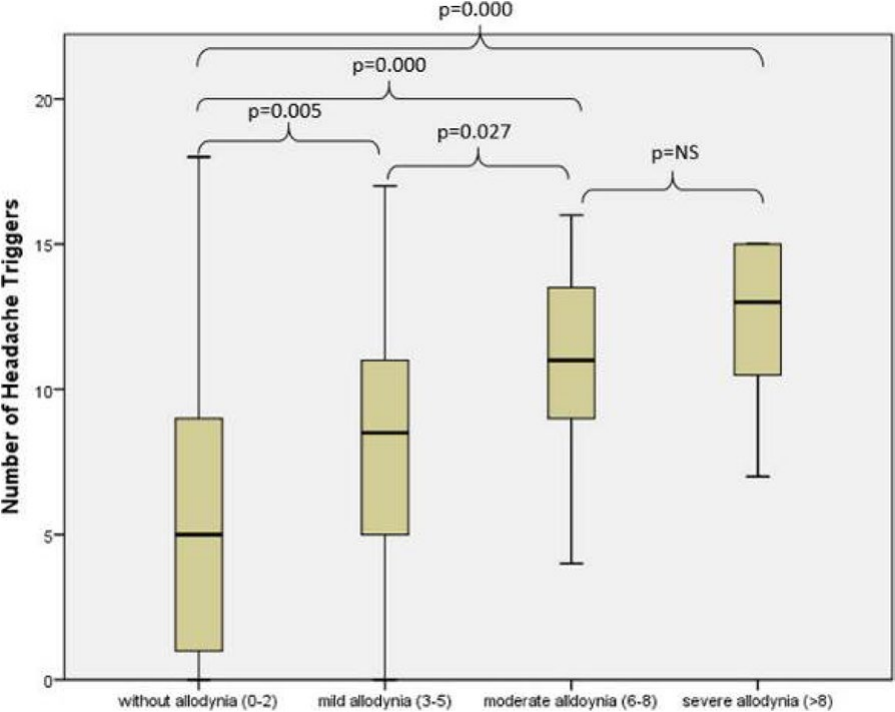
See text for description

**Fig. 2 (Abstract P194):**
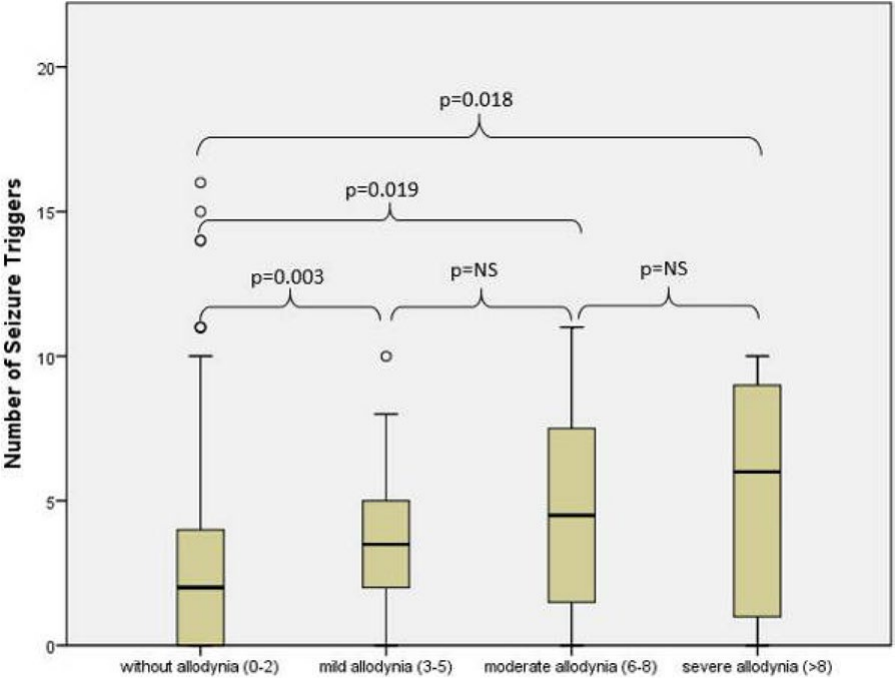
See text for description

**Fig. 3 (Abstract P194):**
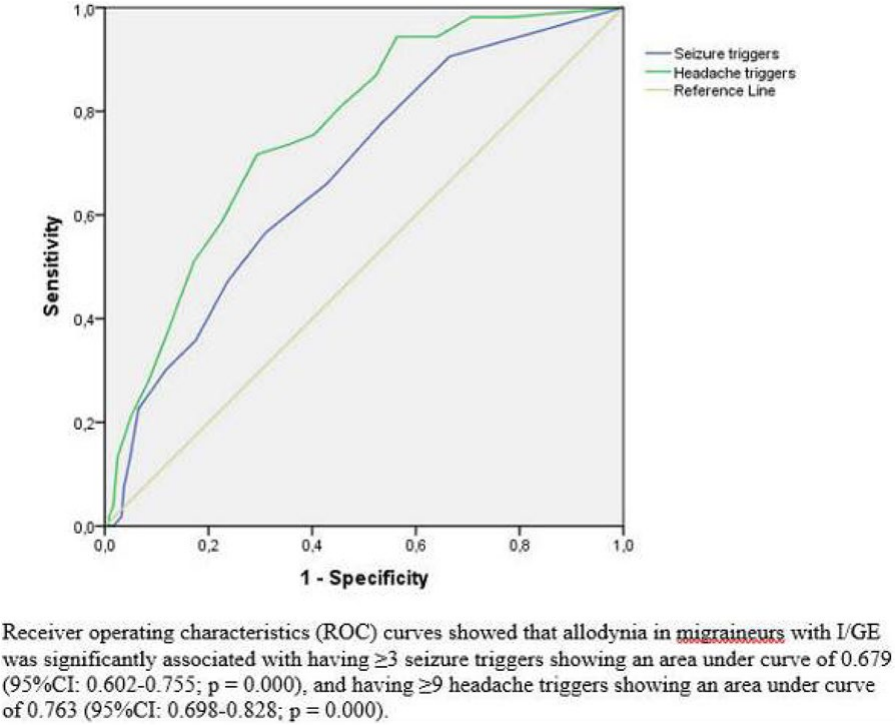
See text for description

## P195 Persistent familial hemiplegic migraine sensorimotor aura due to PRRT2 gene deletion: the first case report

### G. Garascia, A. Granato, L. Bartole, P. Manganotti

#### Cattinara University Hospital, ASUGI, University of Trieste, Neurology Unit, Headache Centre, Department of Medical, Surgical and Health Sciences, Trieste, Italy

##### **Correspondence:** G. Garascia

*The Journal of Headache and Pain 2024,* **25(Suppl 1)**: P195

**Objective:** Familial Hemiplegic Migraine (FHM) is a rare genetic illness in which at least one first- or second-degree relative experiences stroke-like symptoms, typically long-lasting, along with migraine pain. Mutations of three genes, CACNA1A, ATP1A2 and SCN1A, explain the vast majority of FHM cases. A loss of function of the PRRT2 gene, which is involved in neurotransmission, has recently been identified as another major responsible for FHM. The purpose of this study is to describe the first example of FHM with sensorimotor persistent aura related to PRRT2 gene deletion.

**Methods:** We examined the clinical history and neurological symptoms of 27 years old male, migraineur since he was 4 years old.

**Results:** The patient's sister was given a hemiplegic migraine clinical diagnosis at our Headache Centre after being hospitalised for stroke-like symptoms. His headache first manifested as a typical migraine without aura. Since he was 15, there has been a correlation between the pain and a right hemibody strength deficiency that lasts for up to 12 hours after the discomfort first begins. Since he turned 21, he has also had a right hemibody hypoesthesia. Then, weakness and sensitivity deficiencies have always been associated and have lasted for up to 12 hours on average. Speech and visual problems seldom were added. Attacks might occur up to three days each month. When the patient was 27 years old, weakness and hypoesthesia in the right limbs appeared three months earlier were neurologically objectified in our Centre. Brain MRI, brain CT and EEG went negative. The patient was given lamotrigine 50 mg daily starting to improve, but after two months the medication was terminated due to suicide thoughts. After two months of switching from lamotrigine to valproic acid 300 mg daily, aura symptoms disappeared. Later, a heterozygous deletion of the whole PRRT2 gene resulted from MLPA analysis both of the patient and of his sibling.

**Conclusion:** We discussed the first example of FHM with sensorimotor persistent aura linked to PRRT2 gene deletion, responsive to antiepileptic drugs.

*Disclosure statement*: Informed consent to publish this case study and its potentially identifiable information of the patients was obtained from the individuals involved. The patients gave explicit permission for the publication of this case report, including any relevant clinical details.

## P196 Plasma level of calcitonin gene-related peptide (CGRP) in the diagnosis of episodic migraine with comorbid conditions

### O. Dubenko, A. Chernenko

#### Kharkiv national medical university, Neurology and Child Neurology, Kharkiv, Ukraine

##### **Correspondence:** O. Dubenko

*The Journal of Headache and Pain 2024,* **25(Suppl 1)**: P196

**Objective:** Comorbid and co-occurring diseases are risk factors for the progression of EM to chronic migraine. Biomarkers for migraine could help with diagnosis and treatment selection. **Aim -** to examine the role of the CGRP plasma level in the diagnosis of EM in combination with comorbid conditions in the form of cervicalgia and psychoemotional disorders.

**Methods:** The study included 112 patients (84 women, 28 men; mean age 18-58 years), EM with typical aura – 17, without aura – 60, who divided into 3 groups: I – EM with cervicalgia (n = 42), II – EM only (n = 35), III – cervicalgia only (n = 35). The MIDAS, HIT-6, Neck Disability Index, State-Trait Anxiety Inventory, Beck"s Depression Inventory were assessment. The control group had 30 healthy persons to compare the level of CGRP. The serum level of CGRP was determine by the sandwich ELISA principle.

**Results:** Plasma level of CGRP was higher in groups I and II compared with group III (p = 0.012543), where it did not differ from the control (51.48 ± 5.08 pg/ml). The highest level of CGRP was observed in group I (242.98 ± 5.08 pg/ml) in comparison with group II (145.82 ± 15.38 pg/ml, P = 0.000341). Co-occurring neck-pain in patients with EM was associated with mood and anxiety disorders. Migraine severity according to the MIDAS was most significantly influenced by plasma level of CGRP, severity symptoms of headache by the HIT-6, level of State-anxiety and Trait-anxiety, number of days with headache during the last 3 months. The ROC-analysis demonstrated the relationship between the prediction of the degree of impairment of daily activities according to the MIDAS and the plasma level of CGRP (p=0.011). The sensitivity and specificity of the method were 81% and 60%.

**Conclusion:** The serum level of CGRP is a reliable diagnostic and differential diagnostic laboratory biomarker for EM. Additional painful syndrome such as cervicalgia influences CGRP level and daily activity, mood and anxiety disorders in EM patients.

## P197 The significance of the circadian time of administration on the effectiveness and tolerability of OnabotulinumtoxinA for chronic migraine prophylaxis

### E. Dermitzakis^1^, M. Vikelis^2^, A. Argyriou^3^

#### ^1^Euromedica General Clinic, Thessaloniki, Greece; ^2^Mediterraneo Hospital, Headache Clinic, Athens, Greece; ^3^“Agios Andreas” State General Hospital of Patras, Neurology Department, Patras, Greece

##### **Correspondence:** E. Dermitzakis

*The Journal of Headache and Pain 2024,* **25(Suppl 1)**: P197

**Objective:** To provide insights on the role of the circadian time of administration in influencing the efficacy and tolerability/safety profile of ΟnabotulinumtoxinA (BoNTA) for patients with chronic migraine (CM).

**Methods:** We retrospectively reviewed the medical files of BoNTA-naïve patients with CM who completed three consecutive cycles of treatment, according to the standard PREEMPT paradigm. Participants were classified to those scheduled to be treated in the morning hours from 8:00 to 12:00 (AM) or afternoon hours from 13:00 to 18:00 (PM). We then assessed and compared between groups the changes from baseline (T0—trimester before BoNTA"s first administration) to the period after its third administration (T3) in the following efficacy outcomes: (i) mean number of headache days/month, (ii) mean number of days/month with peak headache intensity of >4/10, (iii) mean number of days/month with consumption of any abortive treatment. Safety–tolerability was also compared between groups.

**Results:** A total of 50 AM and 50 PM-treated patients were evaluated. The within-group analysis in both groups showed a significant decrease in all efficacy variables between T0 and T3. However, the between-group comparisons of all BoNTA-related efficacy outcomes at T3 vs. T0 documented comparable improvements between AM vs. PM-treated patients. Safety/tolerability was also similar between groups

**Conclusion:** We were not able to identify significant differences between patients treated in the AM vs. PM, so as to demonstrate that the circadian time of administration should be considered before initiating BoNTA in CM patients.

## P198 Long-term evolution of white matter structural changes in migraine patients

### C. Martín-Martín^1^, Á. Planchuelo-Gómez^2^, Á. L. Guerrero Peral^3,4^, D. García Azorín^3,4^, R. de Luis-García^1^, S. Aja-Fernández^1^

#### ^1^Image Processing Lab – University of Valladolid, Valladolid, Spain; ^2^Cardiff University Brain Research Imaging Centre, Cardiff, United Kingdom; ^3^Hospital Clínico Universitario de Valladolid, Valladolid, Spain; ^4^Universidad de Valladolid, Department of Medicine, Valladolid, Spain

##### **Correspondence:** Á. Planchuelo-Gómez

*The Journal of Headache and Pain 2024,* **25(Suppl 1)**: P198

**Objective:** White matter (WM) changes have been identified in chronic migraine patients (CM) compared to episodic migraine (EM) using diffusion tensor imaging (DTI). There are few previous descriptions regarding migraine evolution over time. Our goal is to evaluate whether patients with CM who improve to EM, after preventive treatment, have a comparable WM structure to those patients with persistent EM.

**Methods:** Single-shell diffusion Magnetic Resonance Imaging (MRI) data was acquired in two different times – time 0 (t_0_) and time 1 (t_1_) – distributed as Figure 1, being mean time between acquisitions of 61.6 ± 9.5 months. Three DTI-based measures were calculated using FSL: Fractional Anisotropy (FA), Axial Diffusivity (AD) and Mean Diffusivity (MD). The average value for each region from the JHU WM atlas was calculated using the 2 and 98% percentiles. *Post-hoc* two-by-two comparisons were considered taking *p* < 0.05 for statistical significance. Three different comparisons were studied (Figure 2A): 1) Patient´s division at t_0_ evaluated at t_0_ (CM_0_ vs. EM_0_), 2) CM_0_ patients´ division evaluated at t_1_, those CM_0_ patients who persist as CM (CM_1_) vs. those improving to EM in t_1_ (EM_1_), and 3) EM_1_ patients´ division evaluated at t_1_, those CM_0_ patients who improve to EM_1_ vs. those persisting as EM_1_ in t_1_.

**Results:** CM_0_ patients showed higher FA and lower MD and AD than EM_0_ (Figure 2B) in regions such as corpus callosum or internal capsule (Figure 3). No statistically significant differences were revealed between the persisting (CM_0_ to CM_1_) and the improving (CM_0_ to EM_1_) CM baseline patients at t_1_ (CM_1_ vs. EM_1_). Patients with EM at t_1_ from different baseline groups (CM_0_ to EM_1_ compared with EM_0_ to EM_1_) showed similar but less differences compared to the differences between CM_0_ and EM_0_ patients.

**Conclusion:** EM patients improving from CM present WM structural properties more similar to those observed in baseline CM than to EM despite the clinical improvement. Patients with CM at baseline preserve the WM differences compared to patients with EM at baseline throughout time regardless the clinical evolution from CM.

**Fig. 1 (Abstract P198):**
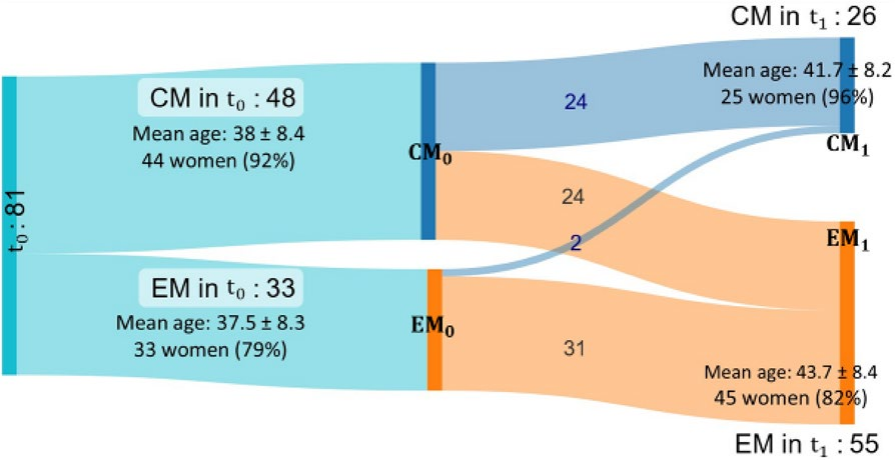
See text for description

**Fig. 2 (Abstract P198):**
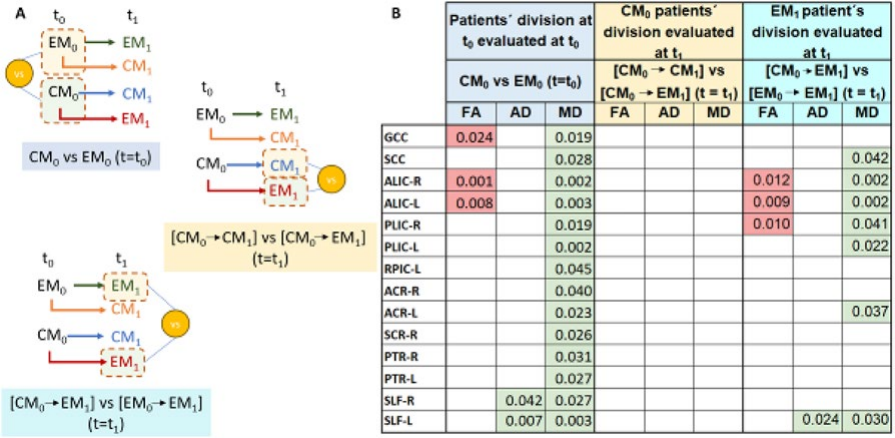
See text for description

**Fig. 3 (Abstract P198):**
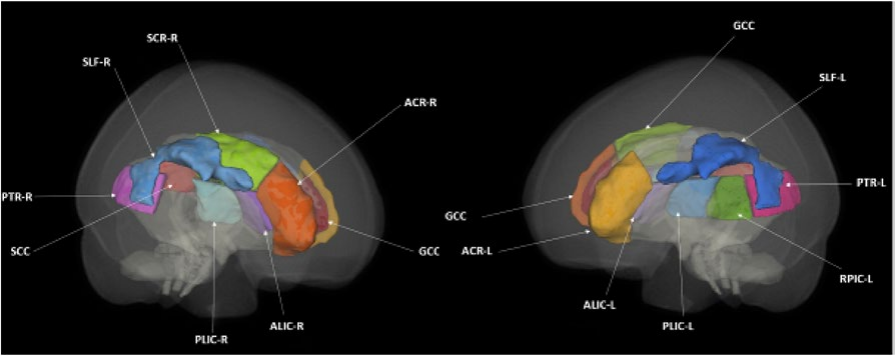
See text for description

## P199 The relationship between serum vitamin D levels and the severity of headache in adult with migraine

### H. Younis, T. Omar, A. Daabis

#### King Fahad Military Hospital, Neuroscience, Jeddah, Saudi Arabia

##### **Correspondence:** H. Younis

*The Journal of Headache and Pain 2024,* **25(Suppl 1)**: P199

**Objective:** Migraine is one of primary episodic headache disorder associated with neurological, gastrointestinal, and autonomic changes. The aim of this study is to compare vitamin D levels with the severity of headache in patients with migraine.

**Methods:** A total of 980 patients diagnosed with migraine were evaluated. We measured vitamin D levels and 25-hydroxy vitamin D3 Serum vitamin D was defined as 50- 125 nmol/l. The severity of the headache was assessed according to number of Migraine days per month.

**Results:** The mean serum 25-hydroxy vitamin D3 levels of migraine patients were 16.6±5.9 ng/ml. As the level of vitamin D decreased, so the severity of the headache increased with increased number of headache days.

**Conclusion:** The severity of headache is associated with reduced serum vitamin D levels in adult with migraine.

**Fig. 1 (Abstract P199):**
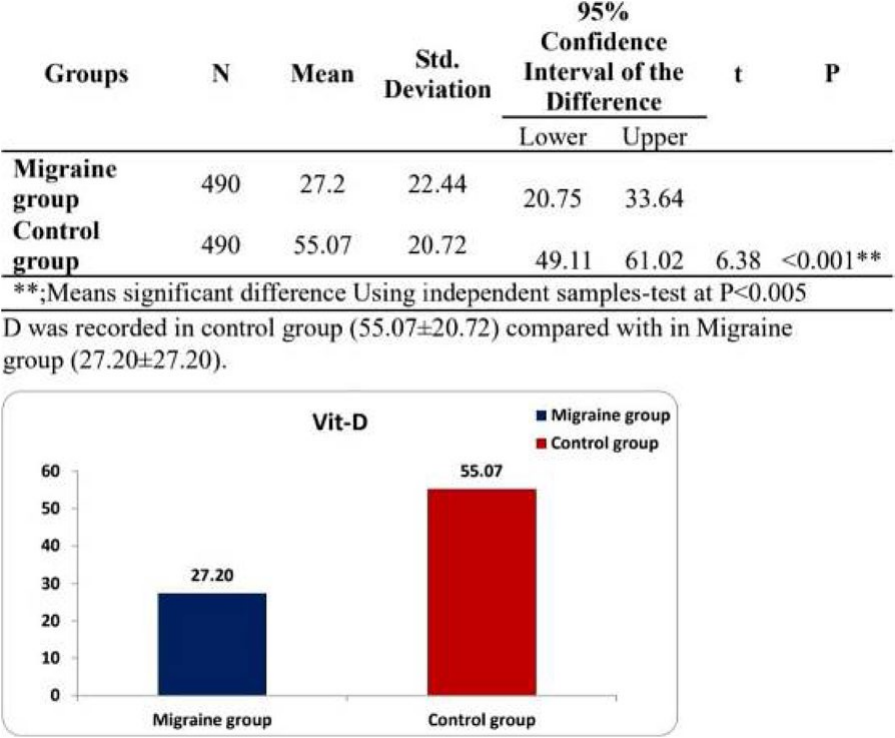
Comparison between Migraine and control groups for Vit. D

**Table 1 (Abstract P199):**
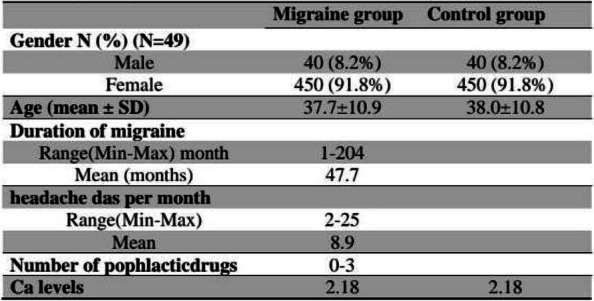
Descriptive data for Migraine and control groups

**Fig. 2 (Abstract P199):**
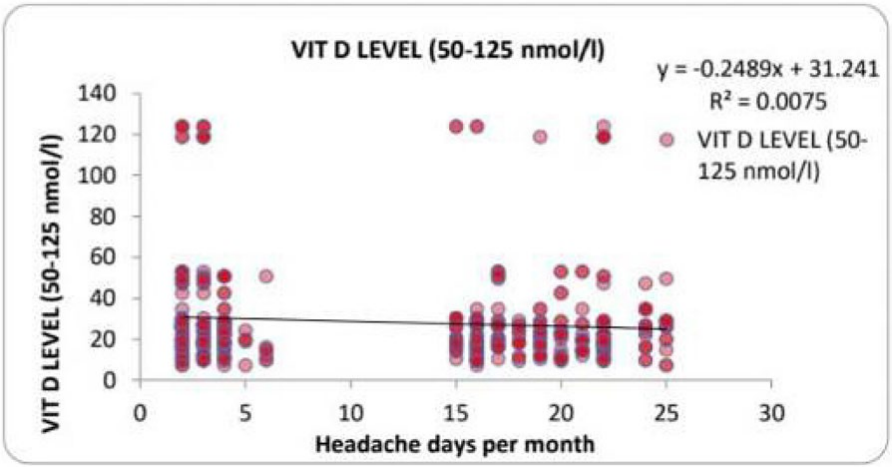
See text for description

## P200 Differential expression of components of the CGRP-receptor family in human coronary and human middle meningeal arteries

### T. de Vries^1^, D. Schutter^1^, A. van den Bogaerdt^2^, R. Dammers^3^, A. J. P. Vincent^3^, A. H. J. Danser^1^, A. MaassenVanDenBrink^1^

#### ^1^Erasmus MC University Medical Center, Departrment of Internal Medicine, Division of Pharmacology and Vascular Medicine, Rotterdam, Netherlands; ^2^ETB-BISLIFE, Heart Valve Department, Beverwijk, Netherlands; ^3^Erasmus MC University Medical Center, Department of Neurosurgery, Rotterdam, Netherlands

##### **Correspondence:** T. de Vries

*The Journal of Headache and Pain 2024,* **25(Suppl 1)**: P200

**Objective:** Some of the gepants induce different responses in human coronary arteries (HCA) and human middle meningeal arteries (HMMA), suggesting the presence of different receptor populations in the two vascular beds. Here, we aim to elucidate which receptors are involved in the relaxation to calcitonin gene-related peptide (CGRP), adrenomedullin (AM) and adrenomedullin 2 (AM2).

**Methods:** RNA was isolated from homogenized human arteries (23 HCAs; 12F, 11M, age 50±3 years and 26 HMMAs; 14F, 12M, age 51±3 years) and qPCR was performed for different receptor subunits. Additionally, concentration-response curves to CGRP, AM or AM2 were constructed in a myograph system, in the presence or absence of the antagonists AM22-52 and/or olcegepant.

**Results:** Calcitonin-like receptor (CLR) was expressed equally in both vascular beds, while calcitonin receptor (CTR) and receptor activity-modifying protein 3 (RAMP3) expression was low and not measurable in all samples. In HCA, the expression of RAMP1 was higher than RAMP2. In HMMA, a mixed population exists with some patients with higher RAMP1 expression and some with higher RAMP2 expression. Moreover, receptor component protein (RCP) expression was higher in HMMA than in HCA. Functional experiments showed that olcegepant inhibits relaxation to all three agonists in both vascular beds. Moreover, AM and AM2 could exert effects via a receptor that can be blocked by AM22-52 in HMMA of some patients, while this was not the case in HCA.

**Conclusion:** Relaxation of HCA is mainly mediated via the canonical CGRP receptor (CLR-RAMP1), while relaxation of HMMA can be mediated via both the canonical CGRP receptor and the AM receptor (CLR-RAMP2). Future research should investigate whether RAMP2 predominance over RAMP1 in the meningeal vasculature results in altered migraine susceptibility or in a different response to anti-migraine medication in these patients. Moreover, the exact role of RCP in CGRP receptor signaling should be elucidated in future research.

**Fig. 1 (Abstract P200):**
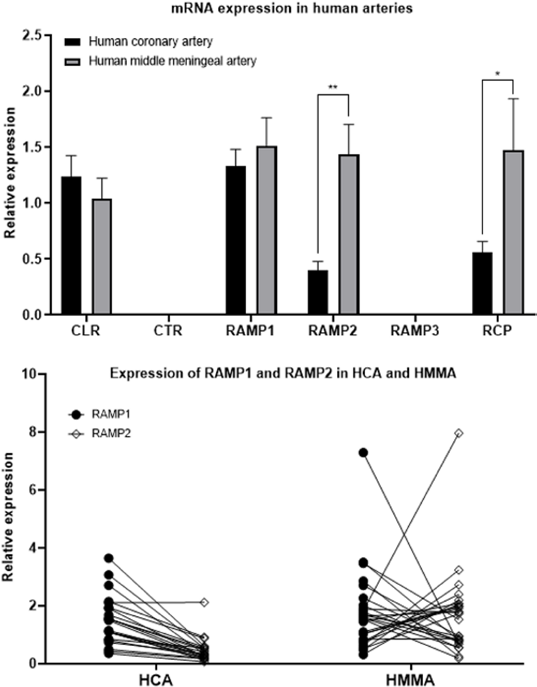
mRNA expression of receptor subunits in the human coronary artery (HCA) and human middle meningeal artery (HMMA)

## P201 Measuring circulating plasma CGRP, PACAP and VIP levels in control and patients with migraine

### A. A. Asuni, J. Søderberg, O. Tzara

#### H. Lundbeck A/S, Copenhagen, Denmark

##### **Correspondence:** A. A. Asuni

*The Journal of Headache and Pain 2024,* **25(Suppl 1)**: P201

**Objective:** Migraine is a disabling disease that continues to pose a significant societal burden. Patients have treatment options, but it remains challenging to identify patients in need as there are no validated biomarkers. Thus, efforts are warranted to find biomarkers to identify individuals with Migraine or explain Migraine chronification. We aimed to investigate the levels of circulating plasma CGRP, PACAP and VIP neuropeptides and circulating immune markers in peripheral blood samples as biomarkers of Migraine.

**Methods:** Plasma samples from patients with Migraine and Controls (n=20) from commercial sources were initially profiled. Subsequently, we profiled plasma samples from patients with Migraine (during and after a headache attack, n=12) and Controls (n=12). Plasma CGRP and PACAP were quantified with a CGRP or PACAP MSD S-PLEX assay, whereas VIP was quantified in a VIP Gyrolab assay. Broad immune profiling was performed with *Olink*® *Target 48* Cytokine panel.

**Results:** In the first cohort, circulating CGRP and PACAP levels were not significantly different in Controls compared to patients with Migraine, whereas VIP levels were elevated in patients compared to Controls. However, in the second cohort all patients with Migraine showed changes in one or more of the three neuropeptides during their attack periods compared to post attack. Circulating plasma CGRP, PACAP and VIP levels in Control subjects were consistent with previously measured levels. Compared to Control plasma samples, Migraine plasma samples showed increases in the levels of several circulating inflammation-related proteins.

**Conclusion:** Plasma CGRP, PACAP and VIP levels are elevated in people with Migraine during an attack, and the increased plasma neuropeptides levels during an attack may help the differentiation of people with and without Migraine. The difference in peripheral immune profile between patients and Controls, further supports a potential role for inflammation in the exacerbation of Migraine pathophysiology.

## P202 Miniscope-based blood vessel imaging enables a novel assay for migraine therapeutic concept testing

### P. Botta^1^, K. Zitelli^2^, Z. Balewski^2^, D. Ollerenshaw^2^, J. Nassi^2^, A. A. Asuni^1^, B. J. Hall^1^

#### ^1^H. Lundbeck A/S, Copenhagen, Denmark; ^2^Inscopix, Inc., Mountain View, CA, United States

##### **Correspondence:** A. A. Asuni

*The Journal of Headache and Pain 2024, ***25(Suppl 1)**: P202

**Objective:** Neurovascular dynamics play an important role in brain health and CNS disorders like migraine, but precisely whether cerebrovascular reactivity in migraine is the disease's cause or consequence remains an active area of research that warrants further attention. Of great interest is the middle meningeal artery (MMA); a dural blood vessel that is implicated in migraine pathology in patients - dilation of this vessel is often associated with the onset of a migraine episode

**Methods:** To enable optical access to MMA and its secondary and tertiary branches, we have been using one-photon miniaturized microscopes ("miniscopes"), which enables high spatio-temporal resolution of blood vessels permitting longitudinal measurements of vessel diameter and/or red blood cell velocity in freely moving mice. Together with our collaborators at Inscopix, we have applied the capabilities of this platform to develop an *in vivo* assay for screening therapeutic compounds in mechanistic migraine-like hypersensitivity models in mice.

**Results:** We observed that Levcromakalim, a drug known to consistently elicit migraine-like attacks in patients, resulted in significant dilation of the MMA compared to vehicle control injections, consistent with published data. Caffeine, administered as a negative control to induce a vasoconstriction response, also resulted in significant MMA dilation after a brief period of vasoconstriction, suggesting a more complex effect of caffeine than previously reported.

**Conclusion:** These data and establishment of the methods lay the groundwork for a miniscope-based assay for efficacy assessment of novel migraine therapeutic with a neurovascular component. We will continue to use these methods to evaluate other potential mechanisms and therapeutic targets implicated in migraine pathophysiology, such as PACAP, CGRP, nitic oxide (NO) and Monoacylglycerol lipase (MAGL) signalling, as well as to assess standard-of-care migraine treatments and novel drug strategies.

## P203 CGRP and calcitonin receptor expression in the amygdala of rats and mice

### L. Forrester^1^, M. Garelja^1^, A. Dawson^1^, T. Rees^2^, D. Hay^1^

#### ^1^University of Otago, Dunedin, New Zealand; ^2^University of Auckland, Auckland, Australia

##### **Correspondence:** D. Hay

*The Journal of Headache and Pain 2024,* **25(Suppl 1)**: P203

**Objective:** Calcitonin gene-related peptide (CGRP) is a key player in the pathophysiology of migraine. CGRP can act through the amylin 1 (AMY1) receptor, comprising the calcitonin receptor (CTR) and receptor activity-modifying protein 1 (RAMP1). To aid understanding of the potential role of this receptor in mediating CGRP signaling, we investigated the distribution of CTR, relative to CGRP, in the amygdala, a component of migraine circuitry in the brain.

**Methods:** Brains were collected from adult male and female Sprague-Dawley rats and C57BL/6J mice. Sections were collected through the amygdala (subregions: lateral, LA; basolateral, BLA; central, CeA; medial, MeA). Fluorescent immunohistochemistry was performed on sections using validated antibodies against CGRP and CTR. Fluorescence was visualised using confocal microscopy on an Opera PHENIX High Content Imager.

**Results:** CGRP and/or CTR like-immunoreactivity (IR) were detected in each subregion of the amygdala in fibers (CGRP and CTR) and/or cell bodies (CTR). Minimal CGRP or CTR like-IR were detected in the LA and BLA. Within the CeA, CGRP like-IR was especially intense in the capsular CeA (CeC). CTR like-IR was more abundant in regions adjacent to the CeC, including the lateral CeA (CeL) and medial CeA (CeM). Strong CTR like-IR was detected in the MeA, where only minimal CGRP like-IR was detected.

**Conclusion:** The proximity of CGRP and CTR in subregions of the amygdala suggest that CTR could be responsible for some CGRP signalling in these regions, potentially as part of the AMY1 receptor.

## P205 Phenotyping migraine patients according to clinical and psychophysical characteristics: a cluster analysis approach (Part 1)

### S. Di Antonio^1,2^, L. Arendt-Nielsen^2^, C. Finocchi^3^, P. Torelli^4^, M. Castaldo^2^

#### ^1^University of Genoa, Department of Neuroscience, Rehabilitation, Ophthalmology, Genetics and Maternal Child Health, Italy; ^2^Aalborg University, Department of Health Science and Technology, Center for Pain and Neuroplasticity (CNAP), SMI, School of Medicine, Aalborg University, Denmark, Aalborg, Denmark; ^3^ASL 2 Savonese, Ospedale San Paolo, Savona, Italy; ^4^University of Parma, Headache Centre, Department of Medicine and Surgery, Parma, Italy

##### **Correspondence:** S. Di Antonio

*The Journal of Headache and Pain 2024,* **25(Suppl 1)**: P205

**Objective:** This study aims to profile migraine patients according to clinical and psychophysical characteristics.

**Methods:** In this observational study, two cohorts of migraine patients (episodic/chronic) were included. Cohort-1: ictal/perictal phase; Cohort-2: interictal phase. The following variables were assessed: headache frequency; disability; cervical active range of motion (AROM); pressure-pain threshold (PPT) over: temporalis, two cervical areas(C1/C4 vertebral segments), and two distal pain-free areas (hand/leg). Cluster analysis was performed using the K-means algorithm. Differences across clusters were investigated.

**Results:** Cohort-1: 100 patients were included, and two clusters were identified. Cluster-1.1(19%), Cluster-1.2(81%). Cluster-1.1 had a higher percentage of men and lower disability compared to Cluster-1.2(all, *p*<0.037). Cluster-1.2 had reduced AROM in flexion, extension, and left/right lateral flexion, and lower PPT value in all areas compared to Cluster-1.1(all, *p*<0.037).

Cohort-2: 98 patients were included, and three clusters were identified. Cluster-2.1(18%), Cluster-2.2(45%), and Cluster-2.3(37%). Cluster-2.1 had a higher percentage of men compared to clusters-2.2 and 2.3(p=0.009). Cluster-2.3 had higher headache frequency, and disability compared to Cluster-2.2(*p*<0.006), and higher disability compared to Cluster-2.1(p=0.010). Cluster-2.3 had reduced AROM in all directions compared to Clusters-2.1 and 2.2(*p*<0.029). Clusters-2.2 and 2.3 have lower PPT values in all areas compared to Cluster-1.1(*p*<0.001).

**Conclusion:** In the ictal/perictal phase, two clusters were identified according to clinical and psychophysical characteristics, with one group showing no psychophysical impairment and one with increased pain-sensitivity and cervical musculoskeletal-dysfunctions.

In the interictal phase, three clusters could be identified, with one group showing no psychophysical impairment, one increased pain-sensitivity, and one increased pain sensitivity and cervical musculoskeletal-dysfunctions.

**Fig. 1 (Abstract P205):**
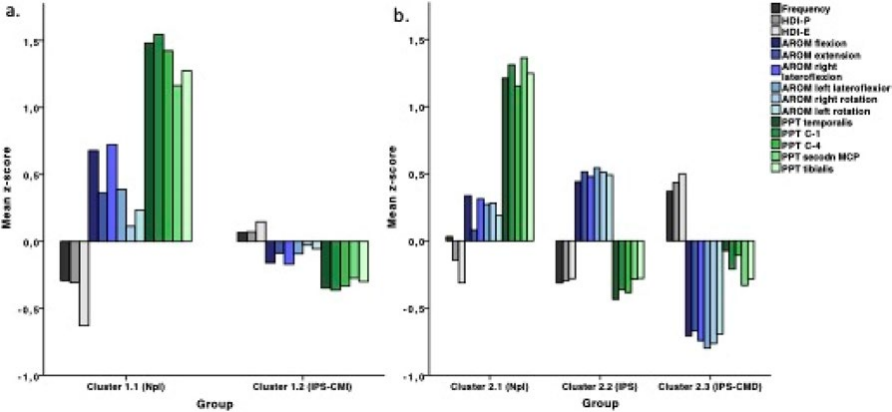
See text for description

**Table 1 (Abstract P205):**
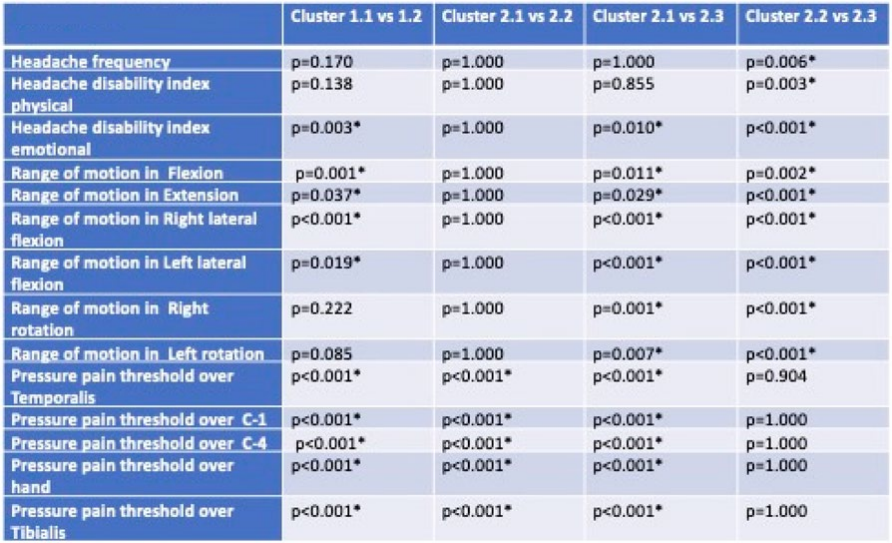
Difference between groups

## P206 Medium-term gray matter structural longitudinal changes in patients with persistent headache after COVID-19

### Á. Planchuelo-Gómez^1,2^, E. J. Gutiérrez-Ocaña^3^, Á. Sierra-Mencía^3^, Y. González Osorio^3^, Á. L. Guerrero Peral^3,4^, M. Rodríguez^5^, R. Moro^5^, S. Aja-Fernández^2^, R. de Luis-García^2^, D. García Azorín^3,4^

#### ^1^Cardiff University Brain Research Imaging Centre, CUBRIC, Cardiff, United Kingdom; ^2^Universidad de Valladolid, Imaging Processing Laboratory, Valladolid, Spain; ^3^Hospital Clínico Universitario de Valladolid, Headache Unit, Department of Neurology, Valladolid, Spain; ^4^Universidad de Valladolid, Department of Medicine, Valladolid, Spain; ^5^Hospital Clínico Universitario de Valladolid, Department of Radiology, Valladolid, Spain

##### **Correspondence:** Á. Planchuelo-Gómez

*The Journal of Headache and Pain 2024,* **25(Suppl 1)**: P206

**Objective:** To determine whether patients who suffered from persistent headache after the acute phase of coronavirus disease 2019 (COVID-19) presented gray matter longitudinal changes at least one year after the diagnosis.

**Methods:** High-resolution 3D brain T1-weighted Magnetic Resonance Imaging (MRI) data were acquired twice in 30 patients who suffered from persistent headache after COVID-19. Gray matter structure was evaluated with the cortical curvature (CC), cortical thickness (CT), surface area (SA) and gray matter volume (GMV), extracted with FreeSurfer (version 6.0), in the cerebellum, and 68 cortical and 14 subcortical regions. Generalized Linear Mixed Models were used to carry out a longitudinal analysis. All models were corrected by age, and intracranial volume in the GMV assessments, establishing *p* < 0.05 as threshold for statistical significance, correcting the results by multiple comparisons using the Benjamini-Hochberg procedure.

**Results:** The mean time between both MRI acquisitions was 19.4 ± 3.9 months. The patients" age at baseline was 42.8 ± 10.4 years, there were 22 women (73.3%), and in 20 patients (66.7%) the number of monthly headache days was reduced at least 50%. All patients presented daily headache at baseline. A CC longitudinal decrease between -0.04% and -0.10% per month compared to baseline was observed in the right orbitofrontal and pericalcarine cortex, lingual, superior frontal and cingulate gyri, and precuneus (Figure 1). Longitudinal CT (Figure 2) and GMV (Figure 3) decrease between -0.08% and -0.10% per month compared to baseline were found in the left rostral middle-frontal and superior frontal gyri, finding specific CT decrease in the left cingulate gyrus (-0.08% per month), and GMV decrease in the superior parietal lobule (-0.09% per month) and frontal pole (-0.11% per month). Longitudinal CT increase of 0.12% per month compared to baseline was found in the left entorhinal cortex (Figure 2). Neither SA nor changes related to the clinical improvement were found.

**Conclusion:** Patients with persistent headache after COVID-19 present gray matter longitudinal changes, defined mainly by CC, CT and GMV decrease, unrelated to the clinical evolution.

**Fig. 1 (Abstract P206):**
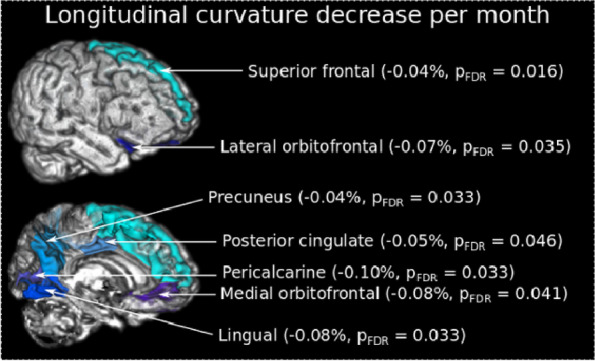
See text for description

**Fig. 2 (Abstract P206):**
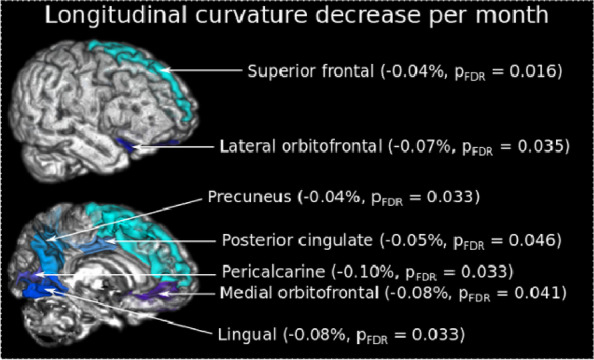
See text for description

**Fig. 3 (Abstract P206):**
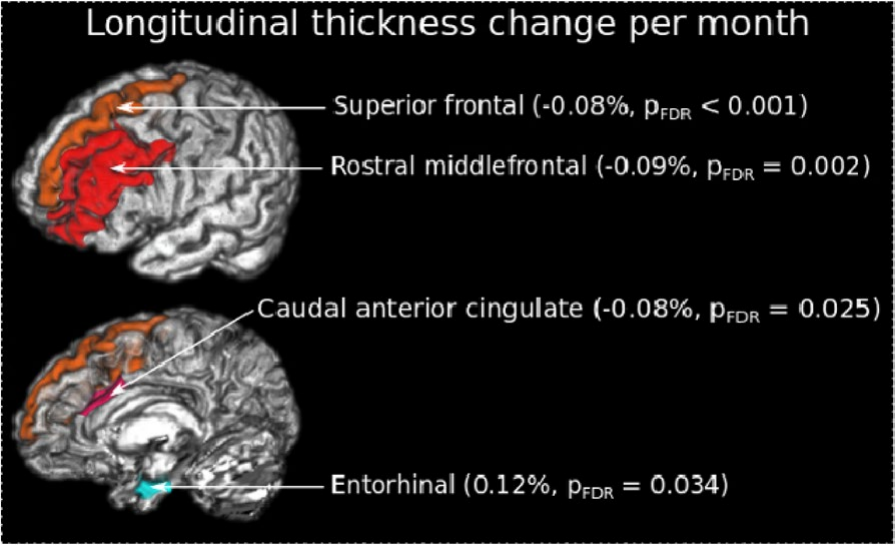
See text for description

## P207 Phenotyping migraine patients according to clinical and psychophysical characteristics: clinical validity of distinct migraine clusters (Part 2)

### S. Di Antonio^1,2^, L. Arendt-Nielsen^2^, P. Torelli^3^, C. Finocchi^4^, M. Castaldo^2^

#### ^1^University of Genoa, Department of Neuroscience, Rehabilitation, Ophthalmology, Genetics and Maternal Child Health, Italy; ^2^Aalborg University, Department of Health Science and Technology, Center for Pain and Neuroplasticity (CNAP), SMI, School of Medicine, Aalborg University, Denmark, Aalborg, Denmark; ^3^University of Parma, Headache Centre, Department of Medicine and Surgery, Parma, Italy; ^4^ASL 2 Savonese, Ospedale San Paolo, Savona, Italy

##### **Correspondence:** S. Di Antonio

*The Journal of Headache and Pain 2024,* **25(Suppl 1)**: P207

**Objective:** Investigate if different clinical and psychophysical bedside tools can differentiate distinct migraine phenotypes.

**Methods:** Two independent samples in which patients were subgrouped into distinct clusters using standardized bedside assessment tools were included: Cohort-1: Ictal/perictal migraine patients were subgrouped, based on previous studies, into: Cluster-1.1 No Psychophysical Impairments (NPI), Cluster-1.2: Increased Pain-Sensitivity and Cervical Musculoskeletal Dysfunction (IPS-CMD); Cohort-2: Interictal migraine patients were subgrouped into: Cluster-2.1 NPI, Cluster-2.2: IPS, and Cluster-2.3: IPS-CMD. Clinical characteristics (multiple questionnaires), quantitative sensory testing (QST), and cervical musculoskeletal impairments were assessed and compared across headache clusters and 56 healthy controls.

**Results:** Cohort-1: Cluster-1.2(IPS-CMD) had worse clinical characteristics assessed through multiple questionnaires (all, *p*<0.048) vs. Cluster-1.1(NPI). Cluster-1.2(IPS-CMD) had reduced cervical active and passive range of motion, reduced functionality of deep cervical flexors, and reduced values in all QST vs. controls (all, *p*<0.023); and reduced active mobility in flexion, left/right lateral flexion, and reduced values in QST(*p*<0.001) vs. Cluster-1.1(NPI) (all, *p*<0045).

Cohort 2: Cluster-2.3(IPS-CMD) had worse clinical characteristics assessed through multiple questionnaires vs. Cluster-2.2(IPS)(all. *p*<0.027) and vs Cluster-2.1(NPI) (all, p<0.010). Cluster-2.3(IPS-CMD) had reduced cervical active and passive range of motion, and reduced functionality of deep cervical flexors, vs. Controls, Custer-2.1(NPI), and Cluster 2.2(IPS) (all,* p*<0.034). Cluster-2.2(IPS) and 2.3(IPS-CMD) had reduced QST values vs. controls and Cluster-2.1(all, *p*<0.039).

**Conclusion:** Clinical and psychophysical bedside tools can separate migraine clusters with different clinical characteristics, somatosensory functions, and cervical musculoskeletal impairments, confirming the existence of distinct migraine phenotypes.

**Table 1 (Abstract P207):**
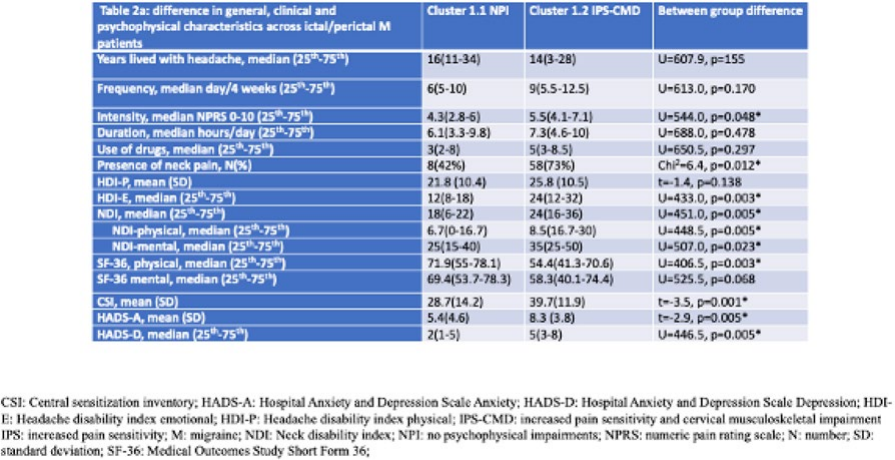
See text for description

**Table 2 (Abstract P207):**
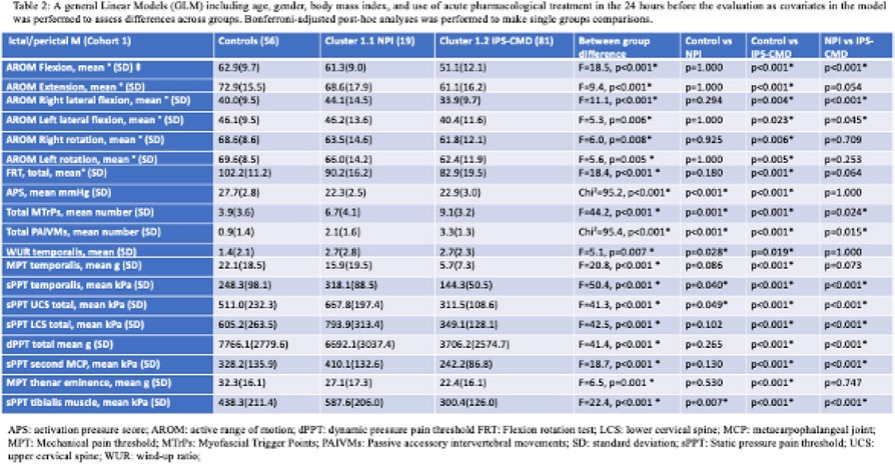
See text for description

## P208 Differences in clinical characteristics, sensitization, musculoskeletal impairments, and psychological burden between migraine patients with and without neck pain

### M. Castaldo^1^, L. Arendt-Nielsen^1^, P. Torelli^2^, C. Finocchi^3^, S. Di Antonio^4,1^

#### ^1^Aalborg University, Department of Health Science and Technology, Center for Pain and Neuroplasticity (CNAP), SMI, School of Medicine, Aalborg University, Denmark, Aalborg, Denmark; ^2^University of Parma, Headache Centre, Department of Medicine and Surgery, Parma, Italy; ^3^ASL 2 Savonese, Ospedale San Paolo, Savona, Italy; ^4^University of Genoa, Department of Neuroscience, Rehabilitation, Ophthalmology, Genetics and Maternal Child Health, Italy

##### **Correspondence:** M. Castaldo

*The Journal of Headache and Pain 2024,* **25(Suppl 1)**: P208

**Objective:** This study aims to assess differences in multiple aspects across healthy controls and migraine patients with (MNP) and without (MwoNP) neck pain.

**Methods:** This study assessed in both populations: headache frequency; headache disability index (HDI); central sensitization inventory (CSI); Hospital Anxiety (HADS-A) and Depression (HADS-D) scale; active range of motion (AROM); flexion rotation test (FRT); activation pressure score (APS); number of active/latent myofascial trigger points (MTrPs) in head/neck muscles; number of positive cervical vertebral segments (C1/C2) who reproduce migraine pain; wind-up ratio (WUR); mechanical pain threshold (MPT) and static pressure pain threshold (sPPT) over the trigeminal area; sPPT and dynamic PPT (dPPT) over the cervical area; sPPTs and MPT over the hand.

**Results:** Compared to controls, MNP had: worse CSI, HADS-A, and HADS-D (all, *p*<0.002); reduced AROM (flexion, extension, left lateral-flexion, and right-rotation), FRT, APS, and a higher number of MTrPs and positive cervical vertebral segments (all, *p*<0.020); reduced trigeminal MPT and sPPT, cervical sPPT and dPPT, hand MPT and sPPT (all, *p*<0.006).

Compared to controls, MwoNP had: worse CSI, and HADS-A (all, *p*<0.002); reduced AROM (flexion, and left lateral-flexion), FRT, APS, and a higher number of MTrPs and positive cervical vertebral segments (all, *p*<0.017); reduced trigeminal MPT and cervical dPPT (all, *p*<0.007).

Compared to MwoNP, MNP had higher headache frequency, worse HDI and CSI (all, *p*<0.006); reduced AROM (flexion, and right rotation) (all, *p*<0.037); reduced cervical dPPT (all, *p*<0.002).

**Conclusion:** MNP had worse headache characteristics, more cervical musculoskeletal impairments, enhanced signs and symptoms related to sensitization, and worse psychological burden compared to MwoNP

**Table 1 (Abstract P208):**
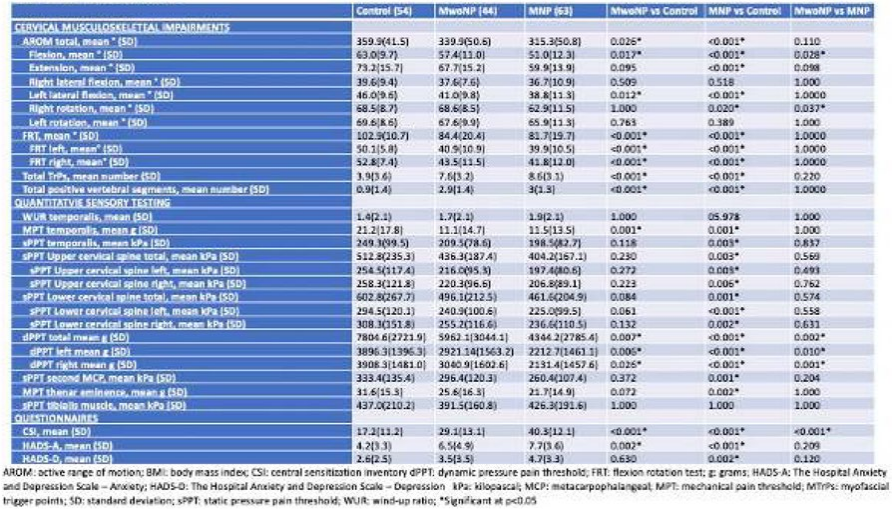
Differences across groups

**Fig. 1 (Abstract P208):**
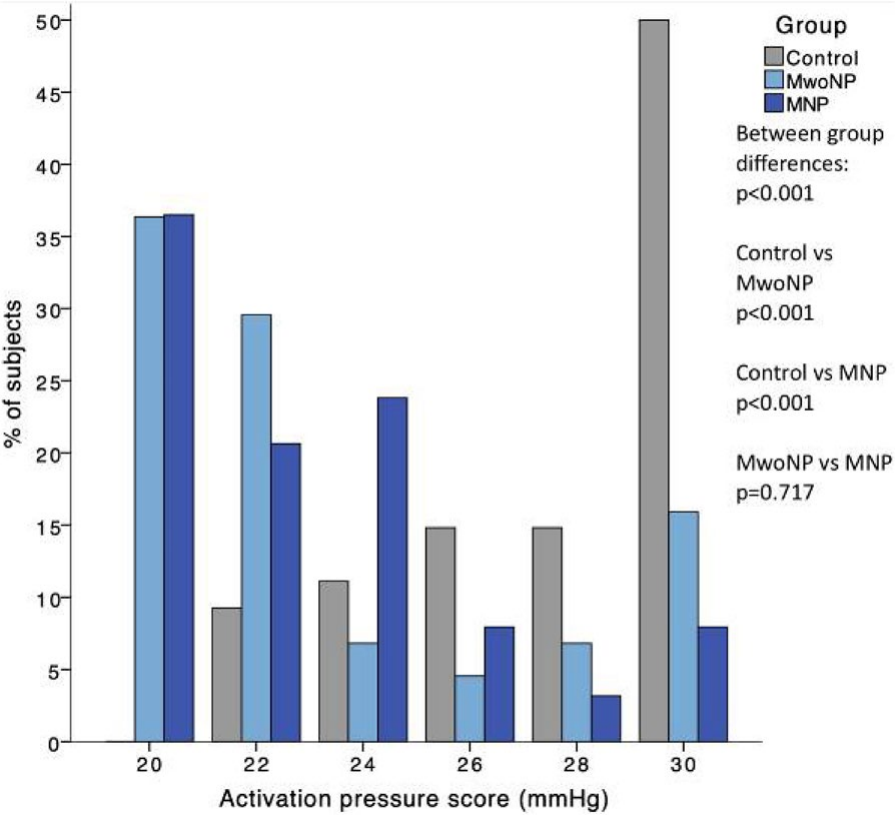
See text for description

## P209 Altered noradrenergic-enriched connectivity in migraine with and without aura

### N. Karsan^1,2,3^, O. Dipasquale^3^, F. Puledda^1,2,3^, R. P. Bose^1,2,3^, M. Mehta^3^, S. Williams^3^, P. J. Goadsby^1,2,4^

#### ^1^King's College London, Headache Group, London, United Kingdom; ^2^NIHR-King's Clinical Research Facility, King's College Hospital, London, United Kingdom; ^3^King's College London, Centre for Neuroimaging Sciences, London, United Kingdom; ^4^University of California, Department of Neurology, Los Angeles, CA, United States

##### **Correspondence:** N. Karsan

*The Journal of Headache and Pain 2024,* **25(Suppl 1)**: P209

**Objective:** Migraine is a complex brain disorder, with pathophysiological evidence for monoaminergic dysfunction. Specific brain circuitry changes within different neurotransmitter networks have not yet been explored. We aimed to use receptor target maps with resting state functional MRI data to identify which neurotransmitters may be modulating brain circuitry changes in migraine, and to compare these to healthy controls.

**Methods:** Using Receptor-Enriched Analysis of Functional Connectivity by Targets (REACT), we estimated and compared the molecular-enriched functional networks of healthy controls (HCs, *n*=21) and patients with migraine (*n*=25), both with (MwA *n*=15) and without aura (MwoA, *n*=10). For REACT, we employed receptor density templates of the transporters of dopamine (DAT), noradrenaline (NAT) and serotonin (SERT), as well as the GABA-A, mGlu5, NMDA and CB1 receptors, and estimated the subject-specific voxel-wise functional connectivity (FC) maps related to these molecular systems in a mixed model analysis, using voxel-wise independent samples *t*-tests (HC vs migraine and MwA vs MwoA) in each system. We also ran a supplementary analysis focused on SERT, exploring FC related to SERT, 5-HT_1B_ and 5-HT_2A_.

**Results:** Significant differences in the NAT-, SERT- (specifically 5HT_2A_) and NMDA-enriched functional networks were identified between migraine and HC, with only the NAT- network surviving multiple comparison correction (*P*=0.0008). Within this network, increased functional connectivity in migraine compared HCs was found in a cluster including the right frontal pole, middle and inferior frontal gyri and the precentral gyrus. Analyses of the MwA and MwoA subgroups showed significant decreases in the mGlu5, CB1 and 5HT2A functional networks in MwA, not withstanding multiple comparison correction.

**Conclusion:** Noradrenaline is involved in alterations in functional connectivity in areas of the prefrontal cortex in migraine, providing a potential neural substrate for cognitive and behavioural change.

## P210 Could a surgery cure migraine? A case report showing the link between migraine and endometriosis

### C. Fernandes^1^, J. M. Alves^1^, M. Coelho^1^, H. Gens^1^, I. Luzeiro^1,2^

#### ^1^Hospitalar and University Center of Coimbra, Neurology, Coimbra, Portugal; ^2^Coimbra Health School, Coimbra, Portugal

##### **Correspondence:** C. Fernandes

*The Journal of Headache and Pain 2024,* **25(Suppl 1)**: P210

**Objective:** Migraine and endometriosis are conditions that affect women of childbearing age, with an estimated prevalence of 18% and 10%, respectively. Our aim was to review the literature on the association of migraine and endometriosis and to describe a case report.

**Methods:** We described a clinical case of a patient diagnosed with migraine and endometriosis and performed a nonsystematic literature search in the PubMed and EMBASE databases. The search strategy included the following MeSH terms: "migraine", "headache" and "endometriosis".

**Results:** A 29-year-old woman was referred to the neurology department because of abdominal pain that occurred simultaneously with migraine attacks. Endometriosis was diagnosed and treatment was started with combined hormone therapy and oral migraine prophylaxis. At age 36, abdominal pain recurred, and the number of migraine days increased. Hormone therapy was switched to GnRH analogs, and onabotulinumtoxinA was started. Two years later, her condition worsened and we suggested anti-CGRP monoclonal antibody treatment. Three months later, we postponed treatment because the patient reported improvement in headache after total hysterectomy and bilateral adnexectomy for refractory endometriosis. Twelve months later, the patient was free of migraine attacks and abdominal pain, with no need for preventive treatment. There is evidence that patients with endometriosis are more likely to suffer from headaches, studies show a prevalence of 30%. Although the reasons for this association are not yet known, it is clear that both conditions are influenced by ovarian hormones and a correlation between the severity of these entities has been suggested. In our clinical case, we speculated on the possibility of secondary headache because patient’s symptoms had improved after surgical treatment for endometriosis.

**Conclusion:** There is some evidence that hormonal influences and chronic inflammatory processes in ectopic endometrial tissue are associated with migraine attacks. The clinical case highlights the therapeutic challenges and allows speculation about the suggestive effect of invasive treatments or the possible role of a secondary headache with migraine-like features in endometriosis.

*Disclosure statement*: Informed consent to publish this case study and its potentially identifiable information of the patients was obtained from the individuals involved. The patients gave explicit permission for the publication of this case report, including any relevant clinical details.

## P211 Psychological profiles and clinical characteristics of migraine patients with early life traumas

### S. Bottiroli^1,2^, M. Allena^1^, R. De Icco^1,3^, G. Sances^1^, G. Vaghi^1,3^, E. Guaschino^1,3^, N. Ghiotto^1^, C. Tassorelli^1,3^

#### ^1^IRCCS Mondino Foundation, Pavia, Italy; ^2^Giustino Fortunato University, Benevento, Italy; ^3^University of Pavia, Pavia, Italy

##### **Correspondence:** S. Bottiroli

*The Journal of Headache and Pain 2024,* **25(Suppl 1)**: P211

**Objective:** To evaluate the impact of childhood traumas in a large sample of subjects with migraine in terms of psychological profiles and clinical characteristics.

**Methods:** A sample of patients with chronic migraine with medication overuse (CM+MO) (n = 200; age: 47.6±10.9) or episodic migraine (EM) (n = 198; age: 39.1±11.1) was enrolled and evaluated for migraine characteristics. Patients received a psychological assessment including self-report questionnaires and, for a subgroup, a clinical interview based on DSM-V criteria for psychopathology and personality disorders.

**Results:** Thirty-five percent of participants reported childhood traumas (CT), with a higher prevalence in the CM+MO (41%) than in the EM (28%) group (p=.006). CT individuals had significantly more days of migraine attacks per month (17.8±11.3 vs 14.1±10.7, p = .002), more days with medication intake (16.7±12.4 vs 13.5±10.1, p = .007) and more doses per month (26.5±26.2 vs 20.4±29.0, p = .04) when compared with patients without CT (wCT). The CT group was also characterized by a significantly higher anxious (8.0±4.0 vs 5.9±3.8, p = .001) and depressive (7.4±4.8 vs 5.2±4.0, p = .001) symptoms, alexithymic levels (47.3±12.7 vs 44.0±12.9, p = 0.04), and a higher prevalence of severe (66% vs 34%, p = .001) and very severe (66% vs 34%, p = .001) current stressors than the wCT group. Moreover, the CT group had a higher prevalence of patients with personality disorders (62% vs 40%, p = .001) and psychopathologies (94% vs 75%, p = .001) than the wCT group.

**Conclusion:** Childhood trauma can have a critical impact on the clinical and psychological characteristics of migraineurs. Patients with childhood trauma are characterized by a more complicated form of migraine associated with psychopathology and personality disorders. These findings have important practical implications and suggest that clinicians should treat these patients also from a psychological perspective because of the high risk of poor prognosis.

## P212 Behavioural characterisation of a rodent model of migraine using preventive and acute treatments

### C. Tsantoulas, D. Cohn, C. Taylor, G. Higgins, M. Duxon

#### Transpharmation, Sandwich, United Kingdom

##### **Correspondence:** C. Tsantoulas

*The Journal of Headache and Pain 2024,* **25(Suppl 1)**: P211

**Objective:** To pharmacologically validate a clinically relevant rodent model of migraine against standard of care analgesic treatments.

**Methods:** Adult male Sprague-Dawley rats (n=10/group) were treated with glyceryl trinitrate (GTN, 10mg/kg, i.p.) either acutely (single administration) or chronically (4 x bidaily injections). Migraine-like pain in response to mechanical stimulation of the plantar or periorbital region was assessed at baseline and following GTN induction using von Frey filaments. The analgesic efficacy of sumatriptan (1mg/kg, s.c.) and topiramate (30mg/kg, i.p.) was assessed.

**Results:** A single GTN administration induced mechanical allodynia at both plantar (from 14.0 ± 1.1g at baseline to 1.7 ± 0.3g at 90min; *p*<0.05) and periorbital (from 17.4 ± 1.7g at baseline to 1.7 ± 0.3g at 90min; *p*<0.05) regions and this effect lasted for at least 5hrs. Acute mechanical sensitivity could be reversed by sumatriptan (BL, 15.1 ± 1.5g; 30min post-dose, 9.1 ± 1.5g; 2hr post-dose, 11.3 ± 2.7g; *p*<0.05). In the chronic model, repeated GTN dosing induced prolonged sensitivity (plantar; from 14.0 ± 1.1 on day 0, to 1.6 ± 0.2g on day 9; periorbital; from 17.4 ± 1.6g on day 0 to 1.2 ± 0.4g on day 9; *p*<0.05). Daily administration of topiramate prevented development of mechanical allodynia at all time points (day 9; plantar, 11.3 ± 2.2g; periorbital, 6.2 ± 0.9g; *p*<0.05 vs vehicle). In contrast, sumatriptan was not effective in preventing chronic pain development (day 0, 12.8 ± 2.5g vs day 7, 2.9 ± 0.8g).

**Conclusion:** Pre-clinical models of migraine in rodents show face validity when tested against golden standard preventive (i.e. topiramate) or rescue (i.e. sumatriptan) treatments. These models can be readily combined with additional endpoints such as non-evoked pain, locomotion and blood flowmetry to facilitate drug discovery.

**Fig. 1 (Abstract P212):**
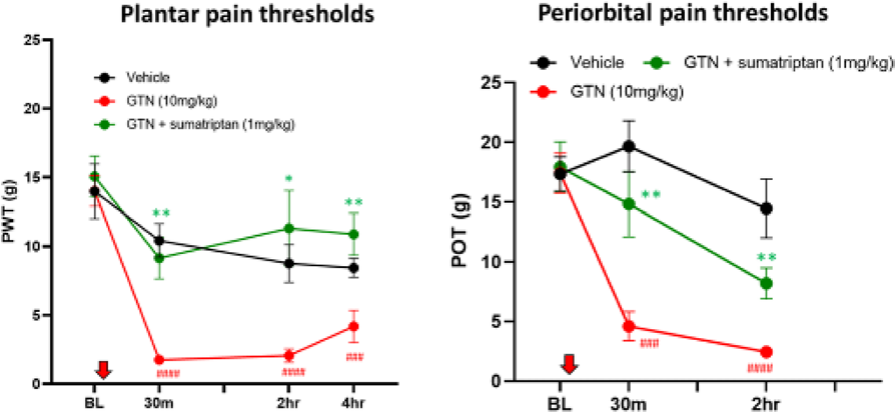
Sumatripan (1mg/kg, s.c.) is analgesic against pain triggered by acute GTN (single injection 10mg/kg i.p.)

**Fig. 2 (Abstract P212):**
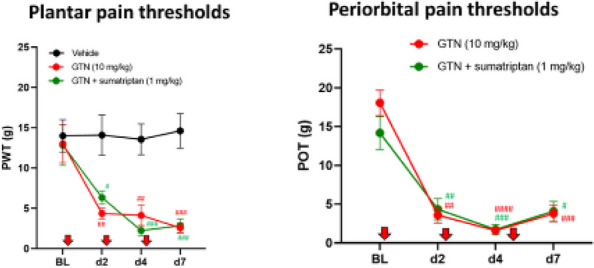
Repeated topiramate treatment (30mg/kg., i.p. daily up to d9) precludes development of migraine pain in the chronic GTN model (4×10mg/kg, bi-daily i.p. injections)

**Fig. 3 (Abstract P212):**
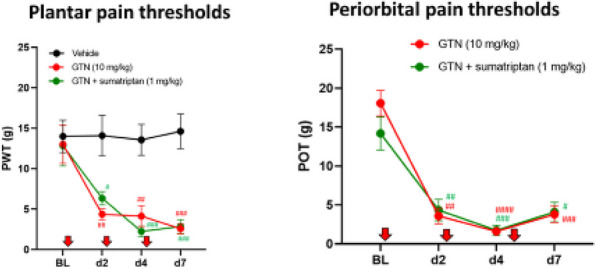
Repeated sumatripan treatments (1mg/kg., s.c. daily up to d7) is not preventive of migraine pain in the chronic GTN model (3×10mg/kg, bi-daily i.p. injections)

## P213 Glial cells and their function in migraine chronification

### Z. Salami^1,2^, M. Togha^2^, S. Razeghi Jahromi^3^

#### ^1^Kharazmi University, Tehran, Iran, Department of Genetics, Faculty of Biology, Tehran, Iran; ^2^Tehran University of Medical Sciences, Headache Department, Iranian Center of Neurological Research, Tehran, Iran; ^3^Shahid Beheshti University of Medical Sciences, Department of Clinical Nutrition and Dietetics, Tehran, Iran

##### **Correspondence:** S. Razeghi Jahromi

*The Journal of Headache and Pain 2024,* **25(Suppl 1)**: P213

**Objective:** chronic migraine usually progresses from episodic migraine. each year 2.5% of episodic migraine patients convert into chronic migraine. with chronification, structural, physiological, and biochemical changes in the brain lead to increased headache frequency and poor response to treatment.

**Methods:** Here, review the previous research results focus on migraine chronification by the interaction between microglial and neurons.

**Results:** Studies have shown that Brain-derived neurotrophic factor (BDNF) is expressed in the trigeminovascular system and is involved in the pathophysiology of migraine. Increased expression of P2X4Rs in TNC is associated with changes in neurochemical symptoms related to migraine in TNC, such as c-Fos and calcitonin gene-related peptide signaling, while TNP-ATP treatment prevents p38-BDNF signaling as well as trigeminal allodynia. P2X4Rs are sufficient to mediate the release and synthesis of BDNF and aberrant activation of microglia and P2X4R expression may be a critical component regulating migraine chronicity. BDNF is a pain modulator because it acts on fast excitatory and inhibitory signals; its action on excitatory signals is potentially mediated by glutamate receptors, especially NMDA receptors. BDNF binding to the Trk receptor, especially binding to the tyrosine kinase B subtype (TrkB), plays a vital role in glial cell proliferation. Findings show that BDNF-TrkB signaling can regulate the expression and release of CGRP from sensory neurons. BDNF binding induces dimerization and autophosphorylation of TrkB and activates its tyrosine kinase activity to signal through three common pathways for Trk family receptors: the PI3K-Akt, MAPK/ERK, and PLCγ pathways. All three are involved in local TrkB modulation of synapse development and modulation events. An increase in the phospho cascades that phosphorylate NMDA increases the probability that this receptor is open.

**Conclusion:** Microglia signaling and microglia-neuron crosstalk involves in central sensitization as an essential regulator in migraine chronification: BDNF downstream and upstream pathways are crucial signaling that mediates this microglia-neuron crosstalk—emphasizing the need to clarify its molecular mechanism.

## P214 Brain connectivity changes induced by monoclonal antibodies targeting the CGRP pathway in migraine patients: a prospective HD-EEG Study

### F. Cammarota^1^, R. De Icco^1^, M. Corrado^1^, G. Vaghi^1^, F. Bighiani^1^, V. Grillo^1^, A. Putortì^1^, D. Martinelli^1^, M. Semprini^2^, M. Allena^1^, G. Sances^1^, C. Tassorelli^1^

#### ^1^IRCCS Mondino Foundation, Department of Brain and Behavioral Sciences, University of Pavia, Pavia, Italy, Pavia, Italy; ^2^Rehab Technologies, Istituto Italiano di Tecnologia, Genoa, Italy

##### **Correspondence:** F. Cammarota

*The Journal of Headache and Pain 2024,* **25(Suppl 1)**: P213

**Objective:** This open-label study aims to investigate the cortical brain connectivity of migraine patients (MIG) and the changes during a one-year treatment with monoclonal antibodies targeting the calcitonin-gene related peptide pathway (CGRP-mAbs) using high-density electroencephalography (HD-EEG).

**Methods:** Forty-five migraine patients (age 44.3±12.3, 39 females, 29 with chronic migraine) who completed the first six months of CGRP-mAbs treatment were included. Five HD-EEG recordings were conducted: baseline (T0) and at three-month intervals (T3, T6, T9, T12). Resting state networks (RSNs) examined included DMN, VN, DAN, VAN, LN, and SMN. Seed-based connectivity analysis was performed for gamma, beta, alpha, theta, and delta bands. Comparison was made with 30 healthy controls (HC, age 37.5±14.0, 14 females).

**Results:** MIG patients exhibited significantly increased delta-band activity and connectivity in the delta range between RSNs compared to HC (*p*<0.005 for all RSNs), which persisted throughout CGRP-mAbs therapy (*p*<0.005 for all RSNs). At T6, the VAN showed decreased alpha activity (*p*=0.01), and there was reduced beta-band connectivity between VAN and VN (*p*<0.05). Treatment responders, based on MIDAS score, displayed enhanced connectivity between DMN and DAN in theta-delta bands (*p*<0.05).

**Conclusion:** MIG patients consistently exhibited increased delta-band activity, possibly representing an electrophysiological signature of the disease. CGRP-mAbs therapy modulated the VAN network and may reduce peripheral trigeminovascular system sensitization. Specific connectivity patterns observed in treatment responders offer insights into individual differences in treatment response. This open-label study with a small sample size calls for further research.

## P215 Characteristics and biomarkers of headache as leading symptom of COVID-19 infection

### O. Duraníková^1,2^, S. Horváthová^1,2^, P. Sabaka^1,2,3^, P. Valkovič^1,2,3^

#### ^1^Comenius University in Bratislava, 2nd Department of Neurology, University Hospital in Bratislava, Bratislava, Slovakia; ^2^Comenius University in Bratislava, 2nd Department of Neurology, Bratislava, Slovakia; ^3^Comenius University in Bratislava, Department of Infectology and Geographical Medicine, Bratislava, Slovakia

##### **Correspondence:** O. Duraníková

*The Journal of Headache and Pain 2024,* **25(Suppl 1)**: P215

**Objective:** Although respiratory symptoms usually prevail the clinical manifestation of COVID-19, headache is one of the most common neurological symptoms with various characteristics. The aim of our study was to assess the prevalence and characteristics of headache as the most dominant symptom during COVID infection and search for possible associated biomarkers.

**Methods:** We conducted retrospective analysis of 295 (126 women) hospitalized patients with confirmed COVID-19 infection in Slovakia 12-15 months after their hospital dismissal. Retrospectively patients were contacted by headache specialists via videocall with self-administered questionnaire. In positive headache patients we searched for participants with headache as leading and most bothersome symptom. Subsequently we evaluated dominant headache"s phenotype and its possible biomarkers.

**Results:** 34.6% (n=102) of patients experienced headache as an accompanying symptom, from which 31.4%(n=32) considered headache as the leading and most bothersome symptom. Headache was mostly unilateral with pulsating quality in 31.3% (n=10) respectively, associated with photophobia in 34.4% (n=11), nausea in 56.3% (n=18) and vomitus in 18.3%(n=6). In 50% (n=16) headache was resistant to analgesics. Patients with headache as a leading symptom had statistically significant higher serum median levels of IL-6 compared with group with headache in which headache was not a dominant symptom (66.5 vs. 44.8, p=0.04). Serum levels of CRP, PCT, vitamin D, ferritin and fibrinogen were higher in group with headache-dominant group, but no statistical significance was present.

**Conclusion:** In our study we confirmed headache as a disabling and most bothersome symptom in 1/3 of patients with predominance of migraine-like characteristics. Headache as a leading symptom was associated with statistically higher levels of IL-6 in comparison with non-dominant headache group, which may reflect more severe cytokine storm during infection.

## P216 Expression of miR-155 in migraine: association with migraine phenotypes and disease severity

### F. Bighiani^1,2^, R. De Icco^1,2^, M. Corrado^1,2^, F. Cammarota^1,2^, M. Allena^1^, V. Grillo^1,2^, R. Greco^1^, C. Demartini^1,2^, A. Zanaboni^1^, G. Sances^1^, E. Guaschino^1^, N. Ghiotto^1^, C. Tassorelli^1,2^

#### ^1^IRCCS Mondino Foundation, Headache Science and Neurorehabilitation Centre, Pavia, Italy; ^2^University of Pavia, Brain and Behavioral Sciences, Pavia, Italy

##### **Correspondence:** F. Bighiani

*The Journal of Headache and Pain 2024,* **25(Suppl 1)**: P216

**Objective:** At present the headache scientific community is intensely investigating the molecular signatures of migraine. microRNAs are small endogenous noncoding RNAs which operate as post-transcriptional regulator of gene expression. Several recent lines of preclinical evidence highlighted the role of miR-155 in inflammation and pain generation and maintenance. In the present study we aim to study the role of miR-155 in migraine, with a particular interest in its association with migraine phenotype and disease severity.

**Methods:** This is a cross-sectional and controlled study involving three study groups: healthy controls (HCs), episodic migraine (EM) and chronic migraine with medication overuse headache (CM-MOH). We assessed the expression of miR-155 (Relative Quantification - RQ) in peripheral blood monocytes. All determinations were performed in the inter-ictal migraine phase.

**Results:** Demographic features were comparable among the three study groups. Anxiety was more represented in CM-MOH when compared to EM (p=0.046). Currently, we analysed miR-155 expression in 23 HCs (0.5±0.16 RQ), 52 EM (1.73±2.09 RQ), and 31 CM-MOH (2.65±2.39 RQ) subjects. Migraine patients showed higher miR-155 expression when compared to HCs (p=0.001). In addition, miR-155 expression was higher in CM-MOH patients when compared to EM group (p=0.002). This finding was confirmed in a logistic regression (EM vs CM-MOH; *p =* 0.019), after controlling for age, sex, ongoing preventive treatment, and psychological comorbidities.

**Conclusion:** Our findings suggest that miR-155 is elevated in migraine patients, and associated with disease phenotype. The study of microRNAs may represent a useful tool to characterized different phenotypes across the migraine spectrum. Hopefully, microRNAs may represent novel molecular targets for drug development ("antagomir").

## P217 Hypothalamus involvement in the migraine cycle: a task-free fMRI analysis during nitroglycerin-induced attacks

### D. Martinelli^1^, M. M. Pocora^1,2^, R. De Icco^1,2^, M. Allena^1^, G. Sances^1^, G. Castellazzi^1^, C. Tassorelli^1,2^

#### ^1^IRCCS Fondazione Mondino, Headache Science and Rehabilitation Center, Pavia, Italy; ^2^Unirsità di Pavia, Brain and Behavioral Science Department, Pavia, Italy

##### **Correspondence:** D. Martinelli

*The Journal of Headache and Pain 2024,* **25(Suppl 1)**: P217

**Objective:** The hypothalamus plays a crucial role in migraine pathophysiology, contributing to pain perception modulation, circadian rhythm disturbances, autonomic dysfunction, and neuroendocrine alterations. In this resting-state functional magnetic resonance imaging (rs-fMRI) study, we assessed the relationship between the hypothalamus and the cortex during various phases of a nitroglycerin-induced migraine attack (NTG).

**Methods:** Ten episodic migraine patients (EM) and 10 healthy controls (HC) underwent 3T MRI scans during subsequent phases of the NTG migraine attack (baseline, prodrome, full-blown attack, recovery). A seed-based correlation analysis assessed the relationship between the hypothalamus and the cortex during the different phases of the attack in EM and at pre-specified time points in HC. A comparison between each scan and the baseline (pain-free) condition was performed, also testing whether there was a correlation between mean functional connectivity (FC) and anamnestic features.

**Results:** At baseline, compared to HC, EM presented a reduced FC between the hypothalamus and supramarginal gyrus, middle frontal gyrus and angular gyrus, and left crus II; FC was increased with the posterior cingulate cortex. No significant FC changes were detected during the prodromal phase when compared to the pain-free condition at baseline. In the full-blown phase instead, EM expressed increased FC between the hypothalamus and right frontal lobe (orbitofrontal and middle frontal gyrus, frontal pole), pons and crus II. These alterations persisted during the painful and the recovery phases. The FC alteration observed during the full-blown phase correlated with the presence of nausea in EM at the time of the scan.

**Conclusion:** These findings help to highlight the disease-specific and phase-specific contribution of the hypothalamus in migraine attacks. fMRI studies have already shown increased hypothalamic activation during migraine attacks, suggesting its involvement in generating pain. This study focuses on its impact on the autonomous system, its role in pain maintenance, and its termination.

## P218 Characteristics and comorbidities of patients with migraine without aura: insights from a cross-sectional study

### M. Togha^1^, O. Kohandel Gargari^2^, S. Nematgorgani^3^, R. Rasekh Magham^3^, Z. S. Ahmadi^3^

#### ^1^Iranian Center of Neurological Research, Neuroscience Institute, Headache Department, Tehran, Iran; ^2^Tehran University of Medical Sciences, Headache Research center, Tehran, Iran; ^3^Shahid Beheshti University of Medical Sciences, Department of Clinical Nutrition and Dietetics, Tehran, Iran

##### **Correspondence:** O. Kohandel Gargari

*The Journal of Headache and Pain 2024,* **25(Suppl 1)**: P216

**Objective:** This cross-sectional study aims to investigate characteristics and comorbidities in patients with migraine without aura and explore the association between comorbidities and headache frequency.

**Methods:** Migraine without aura patients (diagnosed according to the International Classification of Headache Disorders-3) from a Tehran outpatient clinic were included. Data on demographics, medical history, and detailed headache characteristics were collected, including a one-month headache survey reporting episode quality and number. Headache frequency was categorized as low (<4 days), medium (5-8 days), high (9-14 days), and chronic (≥15 days). Chi-square and ANOVA tests assessed associations. *P*<0.05 indicated statistical significance. Data were analyzed using SPSS software

**Results:** The study included 967 migraine without aura patients. Females comprised 80% of the sample. The mean age was 37.38 ± 12.02 years, with an average of 13.65 ± 10.53 headache days per month. Frequency categories were: low (25.2%), medium (18.8%), high (13.6%), and chronic (38.4%). Constipation (29.5%) and sleep disturbance (20.3%) were the most common comorbidities. Other comorbidities observed were dyspepsia, hypertension, dyslipidemia, hypothyroidism, and diabetes mellitus. The chi-square test revealed a significant association between sleep disturbances and more frequent headache episodes (*P*<0.05), while other comorbidities did not show significance (*P*>0.05). Headache frequency did not significantly associate with age or gender (*P*>0.05).

**Conclusion:** this study highlights the high prevalence of comorbidities, such as constipation and sleep disturbance, in patients with migraine without aura. Addressing these comorbidities is crucial for effective management. The significant association between sleep disturbances and more frequent headache episodes underscores the importance of addressing sleep problems in the management of migraine without aura. Further research is needed to investigate the mechanisms underlying these comorbidities and their impact on the course and treatment of this condition.

## P221 Causal relationship between COVID-19 and migraine: a mendelian randomization study

### Z. Xiong, D. Qiu, W. Wang, Y. Mei, Z. Yuan, P. Zhang, Y. Wang

#### Capital Medical University, Headache Center, Department of Neurology, Beijing Tiantan Hospital, Beijing, China

##### **Correspondence:** Z. Xiong

*The Journal of Headache and Pain 2024,* **25(Suppl 1)**: P221

**Objective:** While observational studies have found a correlation between COVID-19 and migraine, the causal relationship remains unclear. Our study aims to fill this gap by utilizing the two-sample Mendelian randomization (MR) method to assess whether COVID-19 has a causal effect on migraine.

**Methods:** We identified COVID-19 genetic instrumental variables (IVs) using a genome-wide association study (GWAS) consisting of 32,494 cases and 1,316,207 controls with European ancestry. These IVs were then used to evaluate the previously reported GWAS for migraine, which included 179,648 individuals also of European ancestry. To assess pleiotropy and heterogeneity in the MR analysis, we employed several statistical methods including MR-Egger_intercept, MR-PRESSO, MR-Egger, and inverse variance weighted (IVW) in Cochran's Q-test. Additionally, we used MR-egger, weighted median, IVW, simple mode and weighted mode for MR analysis. The effect of single nucleotide polymorphisms (SNPs) on SNP bias was also tested.

**Results:** There was no significant pleiotropy or heterogeneity among all four selected COVID-19 genetic instruments in the migraine GWAS. Our analysis in individuals of European ancestry has revealed a positive correlation between genetically increased COVID-19 and an elevated risk of migraine with aura. Specifically, we observed an increasing trend in risk of migraine as COVID-19 genetically increased (β = 0.298, 95% confidence interval [CI]: 1.015-1.790, p = 0.039), and the data were robust without obvious bias.

**Conclusion:** Our findings suggested a putative causal link between genetically increased COVID-19 and an increased risk of migraine in European ancestries.

**Fig. 1 (Abstract P221):**
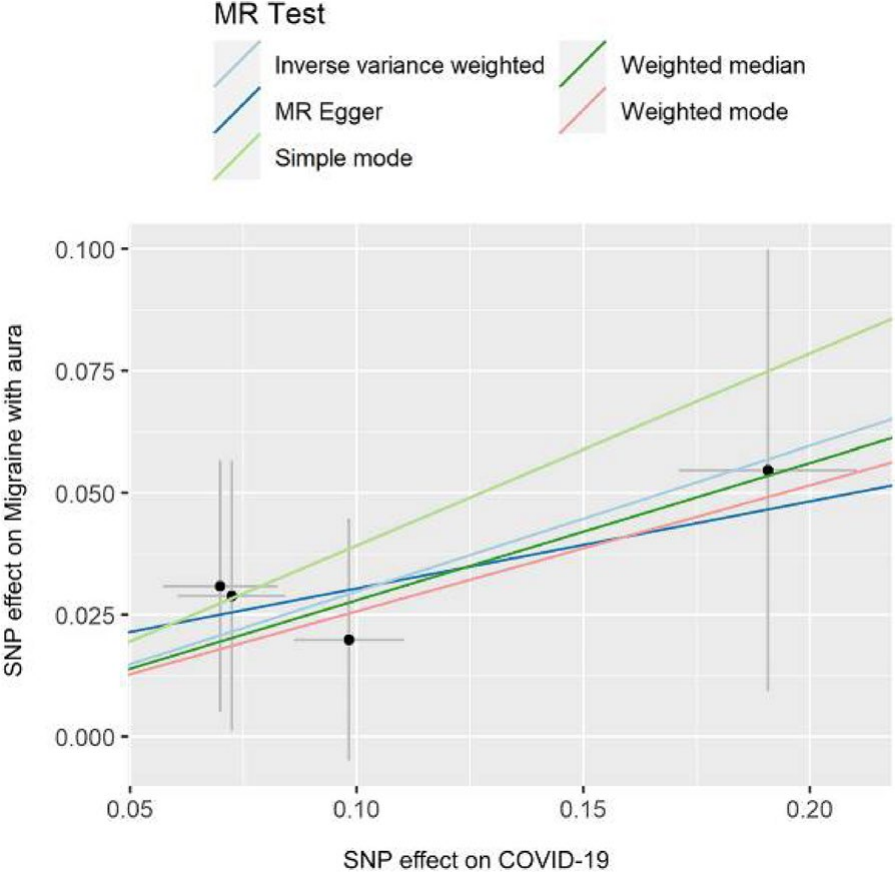
See text for description

**Fig. 2 (Abstract P221):**
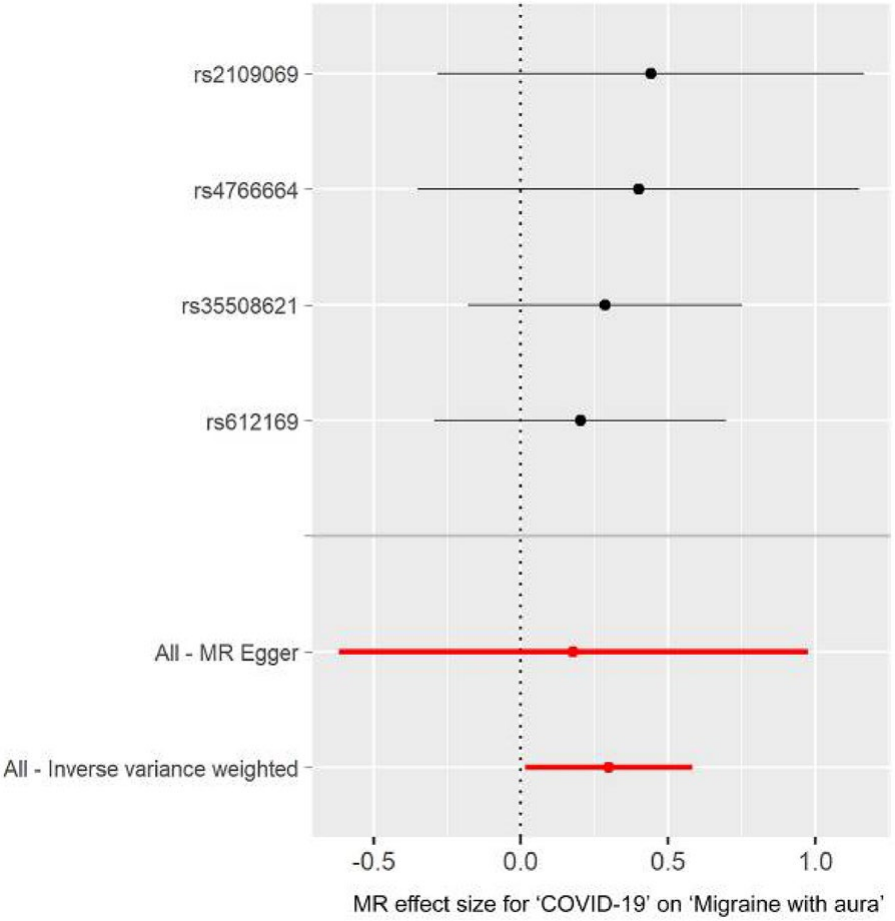
See text for description

**Fig. 3 (Abstract P221):**
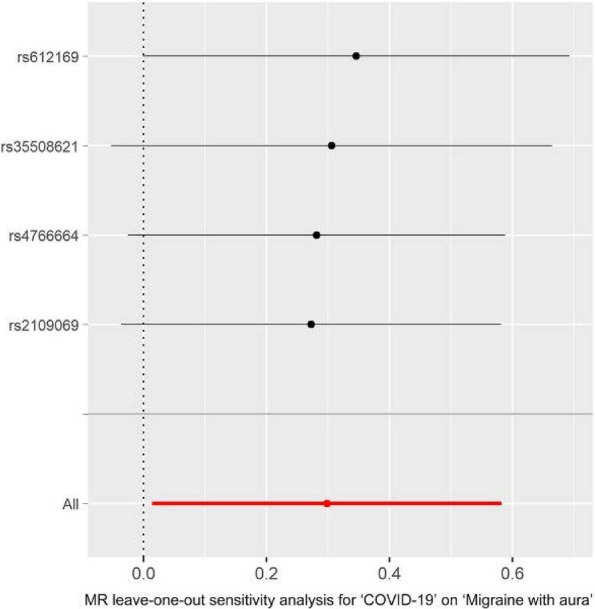
See text for description

## P223 Mapping the aberrant brain functional connectivity in new daily persistent headache: a resting-state functional magnetic resonance imaging study

### W. Wang^1^, Z. Yuan^1^, H. Tang^1^, Y. Mei^1^, D. Qiu^1^, P. Zhang^1^, Y. Zhang^1^, X. Yu^1^, B. Sui^2^, Y. Wang^1^

#### ^1^Beijing Tiantan Hospital, Capital Medical University, Headache Center, Department of Neurology, Beijing, China; ^2^China National Clinical Research Center for Neurological Diseases, Tiantan Neuroimaging Center of Excellence, Beijing, China

##### **Correspondence:** W. Wang

*The Journal of Headache and Pain 2024,* **25(Suppl 1)**: P223

**Objective:** The pathogenesis of new daily persistent headache (NDPH) is not fully understood. We aim to map aberrant functional connectivity (FC) in patients with NDPH using resting-state functional magnetic resonance imaging (MRI).

**Methods:** Brain structural and functional MRI data were acquired from 29 patients with NDPH and 37 well-matched healthy controls (HCs) in this cross-sectional study. Region of interest (ROI) based analysis was used to compare FC between patients and HCs, with 116 brain regions in the automated anatomical labeling (AAL) atlas were defned as seeds. The correlations between aberrant FC and patients" clinical characteristics, and neuropsychological evaluation were also investigated.

**Results:** Compared with HCs, patients with NDPH showed increased FC in the left inferior occipital gyrus, right thalamus and decreased FC in right lingual gyrus, left superior occipital gyrus, right middle occipital gyrus, left inferior occipital gyrus, right inferior occipital gyrus, right fusiform gyrus, left postcentral gyrus, right postcentral gyrus, right thalamus and right superior temporal gyrus. There were no correlation between FC of these brain regions and clinical characteristics, neuropsychological evaluation after Bonferroni correction (*p*>0.05/266).

**Conclusion:** Patients with NDPH showed aberrant FC in multiple brain regions involved in perception and regulation of emotion and pain.

**Fig. 1 (Abstract P223):**
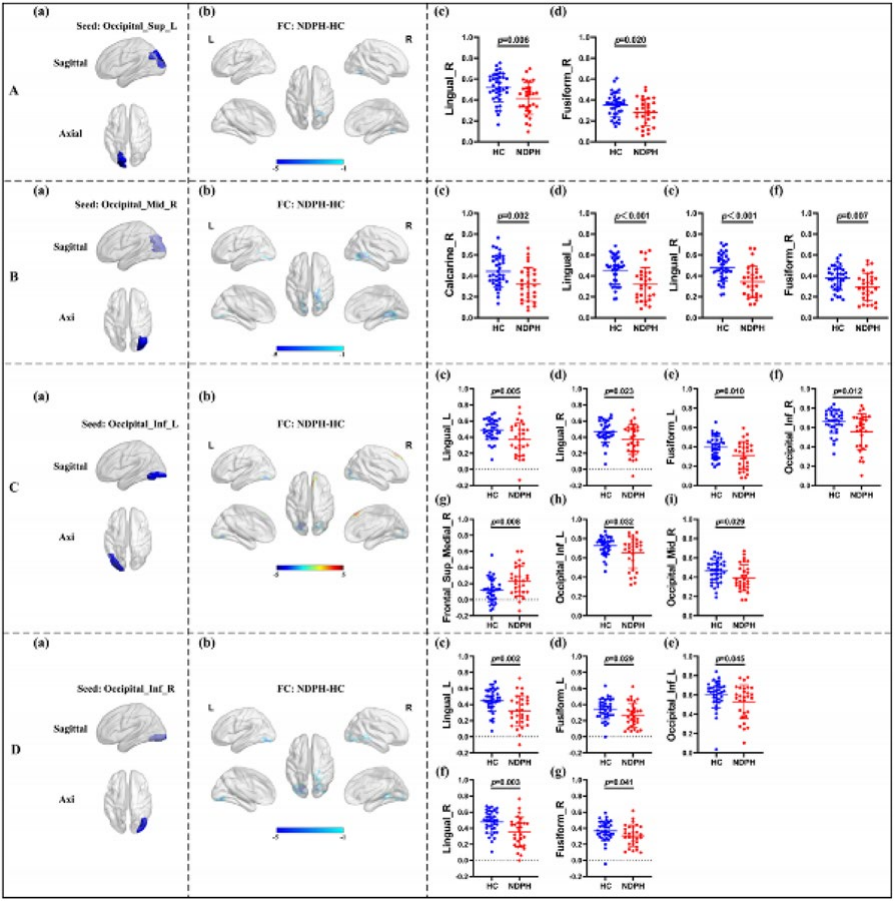
See text for description

**Fig. 2 (Abstract P223):**
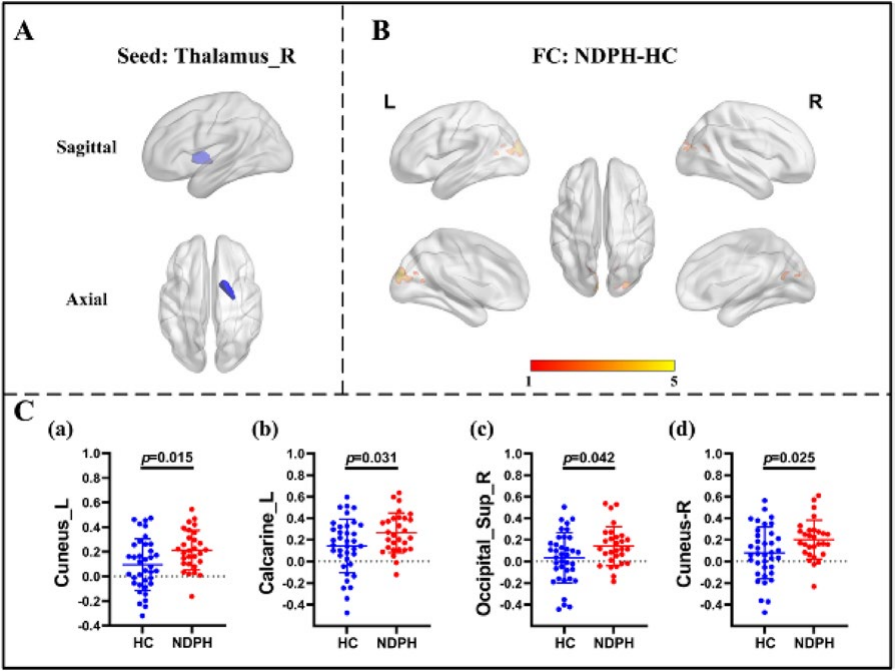
See text for description

**Fig. 3 (Abstract P223):**
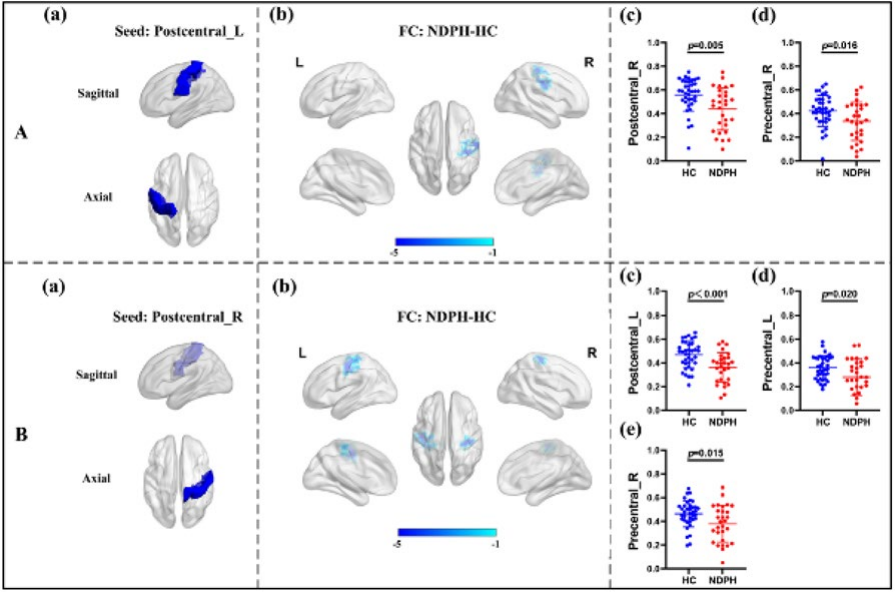
See text for description

## P224 Normal glymphatic system function in patients with new daily persistent headache using diffusion tensor image analysis along the perivascular space

### W. Wang^1^, X. Zhang^2^, X. Bai^2^, Z. Yuan^1^, P. Zhang^1^, Z. Li^2^, H. Tang^1^, Y. Zhang^1^, X. Yu^1^, B. Sui^2^, Y. Wang^1^

#### ^1^Beijing Tiantan Hospital, Capital Medical University, Headache Center, Department of Neurology, Beijing, China; ^2^China National Clinical Research Center for Neurological Diseases, Tiantan Neuroimaging Center of Excellence, Beijing, China

##### **Correspondence:** W. Wang

*The Journal of Headache and Pain 2024,* **25(Suppl 1)**: P224

**Objective:** NDPH, a rare and treatment-refractory primary headache disorder, is poorly understood. There is limited evidence to suggest that headaches are associated with glymphatic dysfunction. Thus far, no studies have evaluated glymphatic function in patients with NDPH. This study aims to investigate the glymphatic function in patients with new daily persistent headache (NDPH) using the diffusion tensor image analysis along the perivascular space (DTI-ALPS) method.

**Methods:** In this cross-sectional study conducted in the Headache Center of Beijing Tiantan Hospital, patients with NDPH and healthy controls were enrolled. All participants underwent brain magnetic resonance imaging examinations. Clinical characteristics and neuropsychological evaluation were examined in patients with NDPH. ALPS indexes for both hemispheres were measured to determine the glymphatic system function in patients with NDPH and healthy controls.

**Results:** In total, 27 patients with NDPH (14 males, 13 females; age [mean ± standard deviation (SD)]: 36.6 ± 20.6) and 33 healthy controls (15 males, 18 females; age [mean ± SD]: 36.0 ± 10.8) were included in the analysis. No significant differences between groups were observed in the left ALPS index (1.583 ± 0.182 vs. 1.586 ± 0.175, mean difference = 0.003, 95% confidence interval [CI] of difference = −0.089 to 0.096, *p*= 0.942), or right ALPS index (1.578 ± 0.230 vs. 1.559 ± 0.206, mean difference = −0.027, 95% CI of difference = −0.132 to 0.094, *p*= 0.738). Additionally, ALPS indexes were not correlated with clinical characteristics or neuropsychiatric scores.

**Conclusion:** No glymphatic dysfunction was detected in patients with NDPH by means of the ALPS method. Additional studies with larger samples are needed to confirm these preliminary findings and improve the understanding of glymphatic function in NDPH.

**Fig. 1 (Abstract P224):**
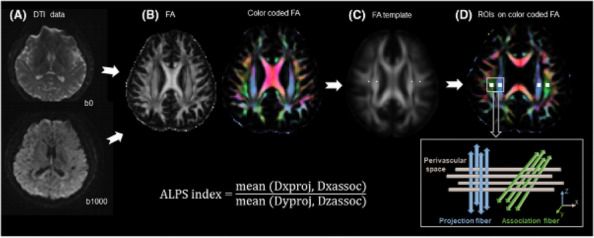
See text for description

**Fig. 2 (Abstract P224):**
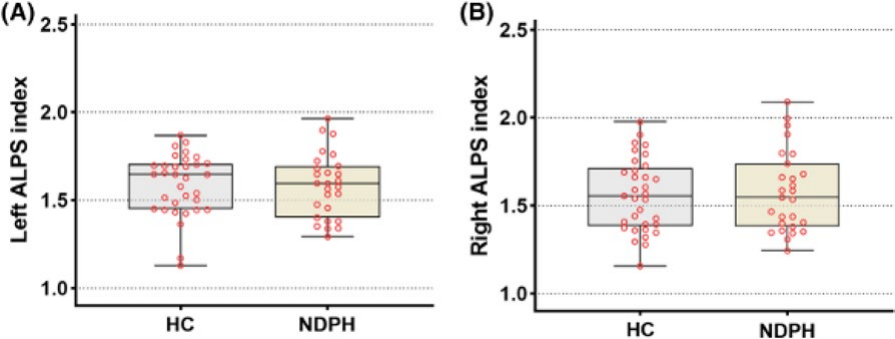
See text for description

## P225 Cerebral perfusion variance in new daily persistent headache and chronic migraine: an arterial spin-labeled MR imaging study

### W. Wang^1^, X. Bai^2^, Z. Li^2^, X. Zhang^2^, Z. Yuan^1^, H. Tang^1^, X. Yu^1^, P. Zhang^1^, Y. Zhang^1^, B. Sui^2^, Y. Wang^1^

#### ^1^Beijing Tiantan Hospital, Capital Medical University, Headache Center, Department of Neurology, Beijing, China; ^2^China National Clinical Research Center for Neurological Diseases, Tiantan Neuroimaging Center of Excellence, Beijing, China

##### **Correspondence:** W. Wang

*The Journal of Headache and Pain 2024,* **25(Suppl 1)**: P225

**Link to published article:**

https://thejournalofheadacheandpain.biomedcentral.com/articles/10.1186/s10194-022-01532-7

**Fig. 1 (Abstract P225):**
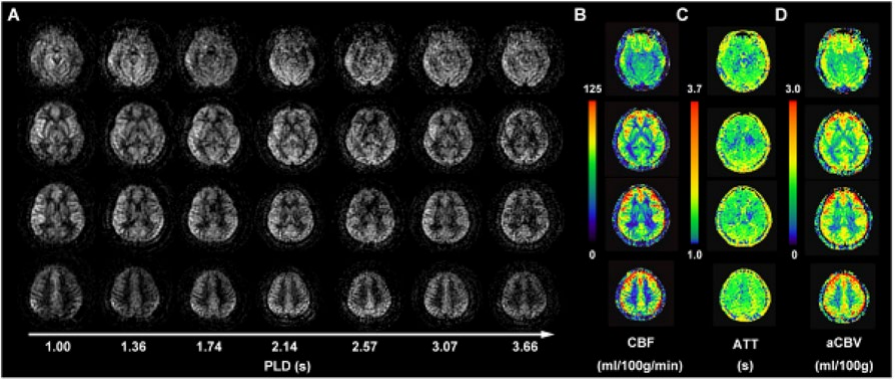
Digram of cerebral perfusion by multi-delay ASL MR imaging. (A) The raw maps of ASL imaging, including different post label delays (PLDs) ranging from 1.00 to 3.66 seconds (s). (B) The arrival-time-corrected cerebral blood flow (CBF) colormaps. (C) The arterial arival time (ATT) colormaps. (D) The arterial cerebral blood colume (aCBV) colormaps

**Fig. 2 (Abstract P225):**
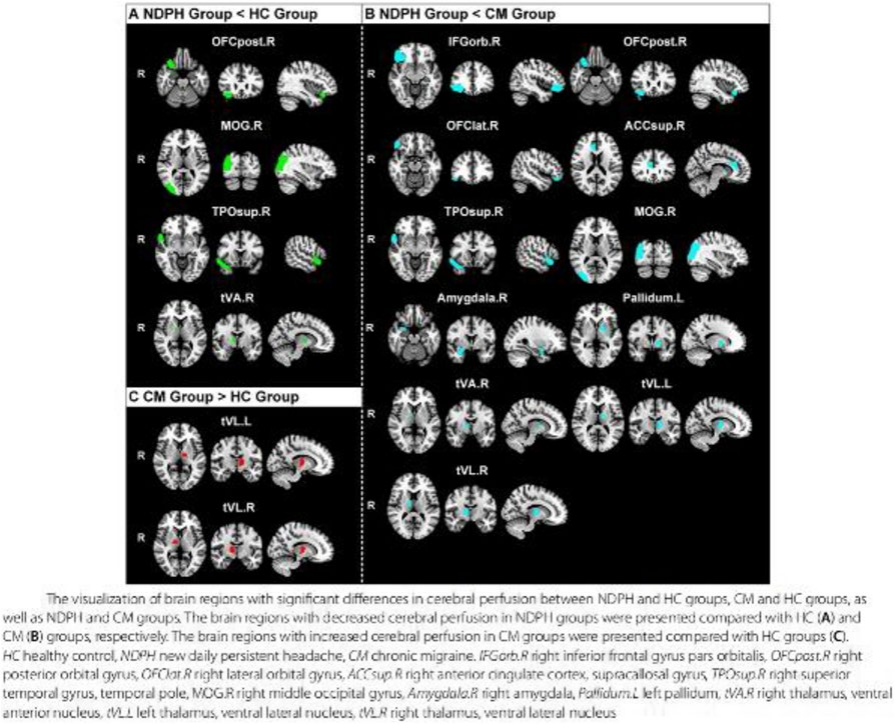
See text for description

**Fig. 3 (Abstract P225):**
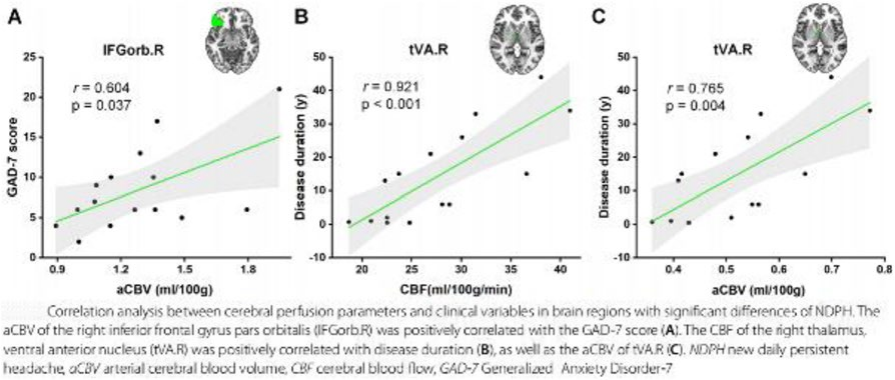
See text for description

## P226 Mechanisms of PACAP38 in genetically modified mice: Relevance of VPAC1, VPAC2 and PAC1 receptors

### S. Guo^1,2^, A. Hay-Schmidt^1^, M. Ashina^2^, A. A. Asuni^3^, J. Olesen^2^, S. L. Christensen^2^

#### ^1^Panum Institute, Faculty of Health, University of Copenhagen, Department of Odontology, Copenhagen, Denmark; ^2^Danish Headache Center, Copenhagen University Hospital – Rigshospitalet Glostrup, Department of Neurology, Copenhagen, Denmark; ^3^H. Lundbeck A/S, Department of Neuroscience, Copenhagen, Denmark

##### **Correspondence:** S. Guo

*The Journal of Headache and Pain 2024,* **25(Suppl 1)**: P226

**Objective:** To increase our understanding of the mechanisms of how pituitary adenylate cyclase-activating peptide-38 (PACAP38) causes migraine-like hypersensitivity via its receptors VPAC1, VPAC2 and PAC1 in a rodent model. A recent phase II trial using PACAP-inhibiting antibodies showed positive results in migraine prevention, whereas antibodies inhibiting the PAC1-R had previously failed. Better understanding of the ligand/receptor interaction may identify more specific drug targets with better efficacy and fewer side effects.

**Methods:** We used an *in vivo* mouse model of provoked migraine-like hypersensitivity based on multiple PACAP38 injections and subsequent measurement of cutaneous tactile sensitivity response with von Frey filaments. PACAP38 response was tested in wildtype (WT) and three genetically modified strains of knockout (KO) mice lacking the respective three PACAP-receptors (*n*total = 138). We also conducted *ex vivo* experiments in the same mice using myograph to test for vasoactivity and performed qPCR to confirm the lack of mRNA expression of the receptor genes.

**Results:** PACAP38 significantly induced hypersensitivity in both WT and KO mice (*P*<0.009), but we found no significant difference in PACAP38 response between WT and the three strains of KO mice (*P*>0.05). However, exploratory analyses using area under the curve showed a partial diminished PACAP38 response in VPAC1 and VPAC2 KO mice compared to WT (*P*<0.05), but not for PAC1 KO mice (*P*>0.05). Myograph experiments supported this finding and showed partial or compensated vasoactivity in VPAC1 and VPAC2 KO mice.

**Conclusion:** This is the first study testing the effect of all three PACAP-receptors on the same in vivo mouse model. The absence of VPAC1-R, VPAC2-R or PAC1-R in KO mice did not show a statistically significant reduction in PACAP38-response compared to WT, but we found a trend of partial diminished response for VPAC1-R and VPAC2-R. The findings suggest that the VPAC-receptors may not be solely responsible for the induction of migraine-like pain but may instead act in conjunction. It is also possible that other receptors or compensatory mechanisms play a role in the genetically modifed mice.

**Fig. 1 (Abstract P226):**
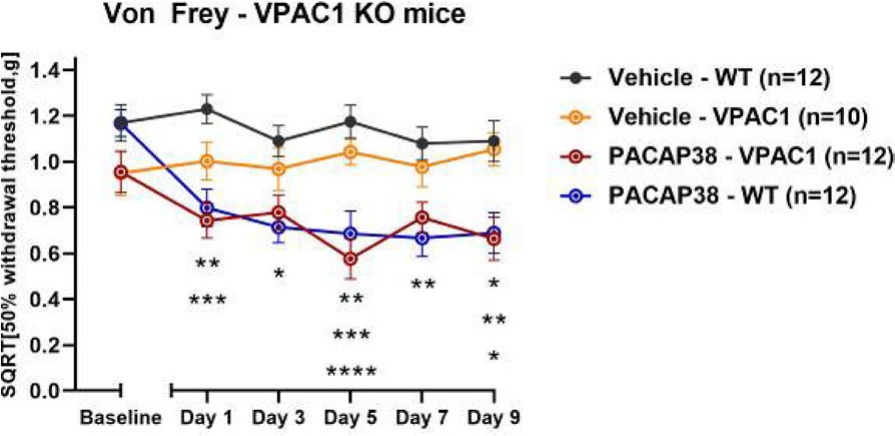
See text for description

**Fig. 2 (Abstract P226):**
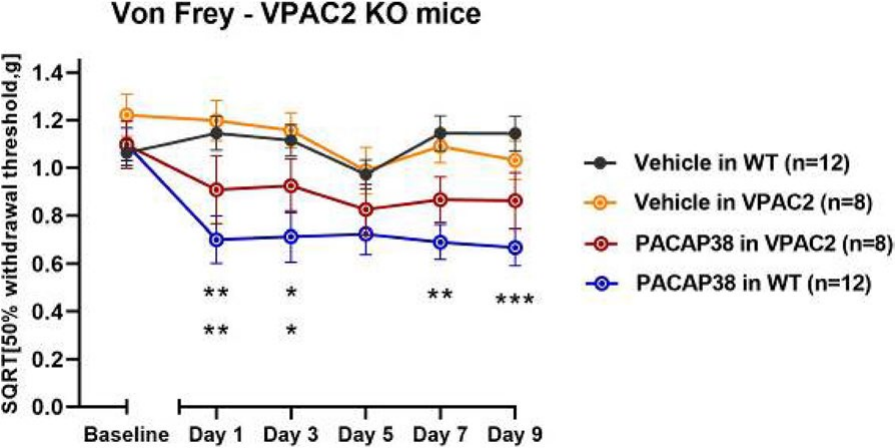
See text for description

**Fig. 3 (Abstract P226):**
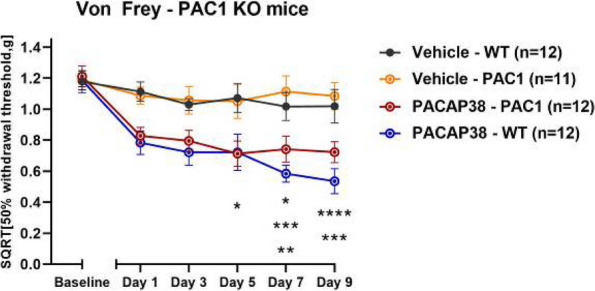
See text for description

## P227 Alpha and beta-CGRP levels in inflammatory bowel diseases and its association with migraine: the importance of discriminate isoforms

### G. Gárate^1,2,3^, M. Pascual^2,3,4^, M. Rivero^2,3,4^, M. J. García^2,3,4^, J. Crespo^2,3,4^, V. González-Quintanilla^1,2,3^, J. Madera Fernandez^1,2,3^, S. Pérez-Pereda^1,2,3^, J. Pascual^1,2,3^

#### ^1^University Hospital Marqués de Valdecilla, Neurology, Santander, Spain; ^2^Universidad de Cantabria, Santander, Spain; ^3^IDIVAL, Santander, Spain; ^4^University Hospital Marqués de Valdecilla, Gastroenterology, Santander, Spain

##### **Correspondence:** G. Gárate

*The Journal of Headache and Pain 2024,* **25(Suppl 1)**: P227

**Objective:** Different studies have stablished an epidemiological association between inflammatory bowel disease (IBD) and migraine. CGRP is, due to its effects and spatial location, a candidate to play a role in both entities.

**Methods:** 85 patients recently diagnosed with IBD (either ulcerative colitis, undifferentiated colitis or Crohn"s disease) and 78 healthy controls without IBD and migraine history, paired by sex and age, were enrolled.

**Results:** Alpha-CGRP levels were increased in IBD (57.0±29.7 pg/mL) compared to HC (45.2±27.5 pg/mL, *p*<0.01) independently of the presence of headache/migraine. When patients were divided into presence of migraine history (*n*=14, 70.6±25.9 pg/mL) and its absence (54.3±29.8 pg/mL) both sub-groups remained significant compared with HC (migraine: *p*<0.01, no-migraine: *p*<0.05) and significant differences arose between sub-groups (*p*<0.05). Beta-CGRP was decreased in IBD (3.1±2.0 pg/mL) compared to HC (4.5±2.6 pg/mL, *p*<0.001) independently of the diagnosis and consistently through all three of the afflictions. Beta-CGRP levels did not change when patients were classified by headache nor migraine history.

**Conclusion:** IBD patients show altered levels of both CGRP isoforms. The increase found in alpha-CGRP clearly represents a junction point between migraine and IBD because it was not found specific for migraine sufferers, although the highest values come from migraineurs. The decrease in beta-CGRP supports for a protective role of the peptide, whose reduced levels could be involved in the altered homeostasis of the intestinal mucosa found in IBD patients. Our results show that, when analysing CGRP levels it is crucial to differentiate between isoforms because depending on what is being measured results can lead to opposite conclusions. Supported by a grant from Fondos Feder (ISCIII PI20/01358).

## P228 Analysing parameters potentially altering serum alpha and beta-CGRP measurements

### G. Gárate^1,2,3^, V. González-Quintanilla^1,2,3^, M. Pascual^2,3,4^, J. Madera Fernandez^1,2,3^, S. Pérez-Pereda^1,2,3^, A. Rufino^1,3^, J. Pascual^1,2,3^

#### ^1^University Hospital Marqués de Valdecilla, Neurology, Santander, Spain; ^2^Universidad de Cantabria, Santander, Spain; ^3^IDIVAL, Santander, Spain; ^4^University Hospital Marqués de Valdecilla, Gastroenterology, Santander, Spain

##### **Correspondence:** G. Gárate

*The Journal of Headache and Pain 2024,* **25(Suppl 1)**: P228

**Objective:** To analyse how different parameters may have an impact on the CGRP levels.

**Methods:** Participants consisted in a subset of healthy volunteers and patients with chronic migraine, inflammatory bowel disease (IBD) or COVID-19 who participated in previous studies in which we determined by ELISA alpha (ABBEXA, UK) and beta (CUSABIO, China) CGRP concentrations.

**Results:** Short-term stability analysis in volunteers revealed that an immediate refrigeration (4oC) of the samples after clotting and prior to its deep congelation (-80oC) preserved the content of the peptides unaltered for up to 24 hours (*n*=6; alpha t0: 29.2±20.6pg/mL, t24: 30.2±19.6 pg/mL *p*=0.69, beta t0: 4.6±1.6 pg/mL, t24: 4.4±1.8 pg/mL, *p*=0.99). The practise of exercise (running 30 minutes) right before blood sampling did not alter CGRP concentration (*n*=6; alpha exercise: 31.0±19.0 pg/mL *p*=0.4; beta exercise: 4.8±1.7 pg/mL, *p*=0.3). Frozen sample storage of more than 6 months was associated with decreased peptide levels when compared to same samples with earlier determinations (*n*=11, *p*<0.01 both). Alpha-CGRP levels did not correlate with age (*n*=400, *p*=0.3) but showed higher concentrations in men when grouped by sex (*p*<0.01). Beta-CGRP levels correlated positively with age (*n*=337, *p*<0.01) but did not show difference between sex (*p*=0.7).

**Conclusion:** CGRP content remains unaltered for at least the first 24 hours when kept refrigerated before getting frozen and is not influenced by immediate exercise. Supporting previous results with plasma, the stability of CGRP in deep frozen samples is limited in periods over 6 months. The effects on the CGRP levels caused by age and sex depends on the isoform and should be considered while designing study groups. Supported by a grant from Fondos Feder (ISCIII PI20/1358)

## P229 Modeling Familial hemiplegic migraine type 3 with patient-specific iPSC-derived ventral forebrain organoid and cellular models

### T. Wang^1,2^, W. Tang^1^, Z. Dong^1^, S. Yu^1^

#### ^1^Chinese PLA General Hospital, Neurology, Beijing, China; ^2^Medical School of Chinese PLA, Beijing, China

##### **Correspondence:** T. Wang

*The Journal of Headache and Pain 2024,* **25(Suppl 1)**: P229

**Objective:** Familial hemiplegic migraine type 3 (FHM3) is a severe form of migraine with aura caused by mutations in the SCN1A gene encoding the voltage-gated sodium channel Nav1.1. Effects of FHM3 mutations on Nav1.1 channels functioning in heterologous expression systems is variable and data from mouse cannot translate to human. Therefore, patient-specific models are needed for investigating the pathology.

**Methods:** We generated induced pluripotent stem cells (iPSC) from one FHM3 patient. FHM3 iPSC and a human control iPSC was differentiated to forebrain GABAergic interneurons (GINs) and ventral forebrain organoid. Functionality of differentiated GINs and ventral forebrain organoid was examined by whole-cell patch-clamp recordings and multielectrode array (MEA) recordings. Bulk RNA sequencing analysis was performed on FHM3 and control lines after 65 days of differentiation to detect the changes in transcriptome profile.

**Results:** The patient had typical clinical manifestations of hemiplegic migraine and carried a novel mutation, F1774C in SCN1A. FHM3 and human control iPSCs differentiate into GINs and ventral forebrain organoid. Electrophysiologic recordings showed significantly lower sodium current density and decreased neural firing in FHM3 GINs compared with control. Consistent with electrophysiologic analysis, MEA recordings showed lower weighted mean firing rate in FHM3 GINs and ventral forebrain organoid. Transcriptome analysis revealed specific dysregulations of genes for binding, catalytic activity, transporter activity and signal transducer activity in FHM3 GINs versus control.

**Conclusion:** We have for the first time investigated FHM3 pathophysiological mechanisms using patient-specific iPSC-derived ventral forebrain organoid and cellular models. Our results indicate a functional decline in FHM3 GINs and patient-specific neurons are useful for modelling FHM3.

## P230 Observational prospective study on the presence of typical migraine features in nummular headache patients: The Numamig study (NCT04299958)

### D. García Azorín^1^, M. Cadenas Astorga^1^, Y. González Osorio^1^, A. Recío García^1^, Á. Sierra-Mencía^1^, P. J. Goadsby^2^, Á. L. Guerrero Peral^1^

#### ^1^Hospital Clínico Universitario de Valladolid, Headache Unit, Valladolid, Spain; ^2^King's College London, London, United Kingdom

##### **Correspondence:** D. García Azorín

*The Journal of Headache and Pain 2024,* **25(Suppl 1)**: P230

**Objective:** Patients with nummular headache (NH) may present a unique headache disorder or as a phenotypic variant of another headache disorder, such as migraine.

**Methods:** To evaluate the frequency and type of typical migraine features in patients with NH, a prospective cohort study was conducted (NCT04299958). Patients were evaluated by a headache expert and were trained in the completion of a headache diary for 14 consecutive days, systematically assessing the presence of 35 additional symptoms before, during or after the NH episodes.

The inclusion criteria were: 1) diagnosis of NH according to the International Classification of Headache Disorders, 3rd version (ICHD-3); 2) ≥ 3 months of NH evolution; 3) Age ≥18 years; 4) ability to register headache characteristics in a diary; 5) informed consent signature. Patients were excluded if they had 1) other primary or secondary headache, except for infrequent tension-type headache or medication overuse headache; 2) major psychiatric disorders; 3) alcoholism or drug abuse.

**Results:** 19/51 patients were eligible, 15 (79%) female, aged 62 [inter-quartile range (IQR): 44-79] years. NH duration at enrolment was 24 [IQR: 7-36] months. Patients registered 291 NH episodes, with a median duration of 10 [IQR: 6.6-15] hours. Patients reported bilateral pain in 2 (1%) episodes, pulsating quality in 57 (20%), moderate-to-severe intensity in 235 (81%) and worsening by routine physical activity in 75 (26%) episodes. Regarding associated symptoms, patients described nausea and/or vomiting in 29 (10%), photophobia and phonophobia in 64 (22%) episodes. The proportion of patients who fulfilled migraine phenotypic criteria was: criterion C in 233 (80%) episodes, criterion D in 79 (27%), and all phenotypic migraine criteria in 71 (24%) episodes.

**Conclusion:** Typical phenotypic features of migraine were observed in half of patients with NH with no prior history of migraine, which suggest a common pathophysiology.

## P231 Familial hemiplegic migraine caused by simultaneous mutations in ATP1A2 and SCN1A, a new subtype?

### T. Wang^1,2^, W. Tang^1^, Z. Dong^1^, S. Yu^1^

#### ^1^Chinese PLA General Hospital, Neurology, Beijing, China; ^2^Medical School of Chinese PLA, Beijing, China

##### **Correspondence:** T. Wang

*The Journal of Headache and Pain 2024,* **25(Suppl 1)**: P231

**Objective:** Familial hemiplegic migraine (FHM) is an autosomal dominant disorder and characterized by transient hemiplegia during the aura phase. Three major genes have been identified to be associated with FHM, including voltage-dependent calcium channel, alpha 1A subunit (CACNA1A) for FHM1, ATPase Na+/K+ pump, alpha 2 subunit (ATP1A2) for FHM2, and voltage-gated sodium channel type 1 alpha subunit (SCN1A) for FHM3.

**Methods:** We identified a Chinese FHM family and whole-exome sequencing revealed the existence of two novel mutations, namely R279Q in ATP1A2 and F1774C in SCN1A. To ascertain whether one of these mutations is causative gene or if both are pathogenic, we conducted following methods, mutation prediction, two-electrode voltage-clamp recordings conducted in microinjected xenopus oocytes, generation of patient-derived iPSC models, differentiation of iPSCs into GABAergic interneurons (GINs), and whole-cell patch clamping.

**Results:** Six patients in four generations within the family affected by FHM exhibited the characteristic clinical manifestations associated with this condition. Patients harboring two mutations experienced more severe headaches, with one individual also experiencing concurrent headaches and epilepsy. Six out of seven computational algorithms indicated that the R279Q mutation was benign, but oocytes study revealed a reduction in ouabain-sensitive pump currents and lower affinity for potassium. F1774C mutation obtained scores over the pathogenic threshold in all seven prediction systems. We generated iPSCs from two FHM family members, and then differentiated them into GINs. Electrophysiologic recordings showed notable reduction in sodium current density and decreased neural firing in FHM GINs.

**Conclusion:** This study presents a novel finding of a family exhibiting the simultaneous presence of two pathogenic mutations within the same disease, thereby enhancing its pathogenicity. This unique occurrence establishes the first FHM-Mixed pedigree documented to date.

## P232 Photophobia in headache disorders: characteristics and potential mechanisms

### Z. Xiao

#### Renmin Hospital of Wuhan University, Department of Neurology, Wuhan, China

*The Journal of Headache and Pain 2024,* **25(Suppl 1)**: P232

**Objective:** Photophobia is present in multiple types of headache disorders. The coexistence of photophobia and headache suggested the potential reciprocal interactions between visual and pain pathways. The aim of this review is to summarize photophobic characteristics of different headache disorders and to propose a common mechanism explaining photophobia in all three categories of headache disorders.

**Methods:** We systematically searched all works from MEDLINE, EMBASE and Cochrane databases from 2002 to the present. Searches were performed using a combination of MeSH (medical subject headings) terms and key terms.

**Results:** In this review, we summarized the photophobic characteristics in different types of headache disorders in the context of the three diagnostic categories of headache disorders: (1) primary headaches: migraine, tension-type headache, and trigeminal autonomic cephalalgias; (2) secondary headaches: headaches attributed to traumatic brain injury, meningitis, non-traumatic subarachnoid hemorrhage and disorder of the eyes; (3) painful cranial neuropathies: trigeminal neuralgia and painful optic neuritis. We then discussed potential mechanisms for the coexistence of photophobia and headache.

**Conclusion:** In conclusion, the characteristics of photophobia are different among these headache disorders. The coexistence of photophobia and headache is associated with the interactions between visual and pain pathway at retina, midbrain, thalamus, hypothalamus and visual cortex. The communication between these pathways may depend on calcitonin gene-related peptide (CGRP) and pituitary cyclase-activating polypeptide (PACAP) transmission. Moreover, cortical spreading depression (CSD), an upstream trigger of headache, also plays an important role in photophobia by increased nociceptive input to the thalamus.

## P233 Proteomics profiling reveals mitochondrial damage in the thalamus in a mouse model of chronic migraine

### W. Xie^1^, R. Li^2^, C. Li^1^, Z. Ma^1^, Y. Zhou^3^, S. Yu^1^

#### ^1^The First Medical Center of Chinese PLA General Hospital, Department of Neurology, Beijing, China; ^2^The First Medical Center of Chinese PLA General Hospital, Department of Laboratory Medicine, Beijing, China; ^3^Northwest University, College of Life Science, Xi’an, China

##### **Correspondence:** W. Xie

*The Journal of Headache and Pain 2024,* **25(Suppl 1)**: P233

**Objective:** Migraine is a complex brain disorder, which is regarded as a possible clinical manifestation of brain energy dysfunction. We performed quantitative proteomics to analyze the protein characteristics of specific regions in the trigeminovascular system, focusing on the changes in proteins related to mitochondrial function.

**Methods:** The mouse model of chronic migraine was established by repeated nitroglycerin (NTG) stimulation. Differentially expressed proteins (DEPs) in some subcortical brain regions of the trigeminovascular system were screened based on liquid chromatography-tandem mass spectrometry (LC-MS/MS). Compare the commonality and specificity of key signal pathways in different brain regions. Mitochondrial function was measured by Elisa, and mitochondrial morphology was observed by transmission electron microscope.

**Results:** The mouse central sensitization model of chronic migraine was successfully established by repeated NTG stimulation, which was characterized by periorbital and hind paw allodynia, and photophobia. The results of quantitative proteomics showed that 529,109,163,152 and 419 DEPs were detected between the NTG and control group in the thalamus, hypothalamus, periaqueductal grey (PAG), trigeminal ganglion (TG) and trigeminocervical complex (TCC), respectively. No common key signal pathways were found across brain regions. The most significant change in the brain region-specific pathway was the thalamic mitochondrial dysfunction. Compared with the control group, the concentration of ATP and the activity of respiratory chain Complex I decreased (*P* < 0.05), and the number of abnormal mitochondria per unit area increased (*P* < 0.01) in the thalamus in the NTG group.

**Conclusion:** Our findings highlight the involvement of mitochondrial damage in the thalamus in central sensitization of chronic migraine, which provides evidence of possible metabolic mechanisms in migraine pathophysiology.

**Fig. 1 (Abstract P233):**
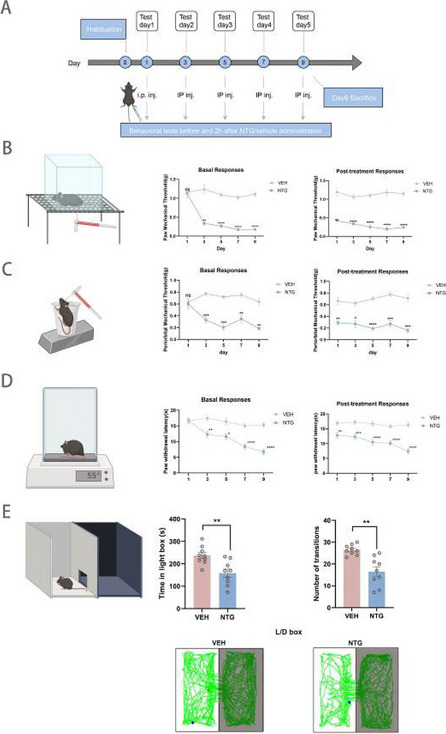
See text for description

**Fig. 2 (Abstract P233):**
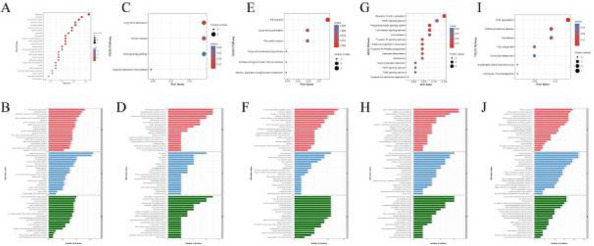
See text for description

**Fig. 3 (Abstract P233):**
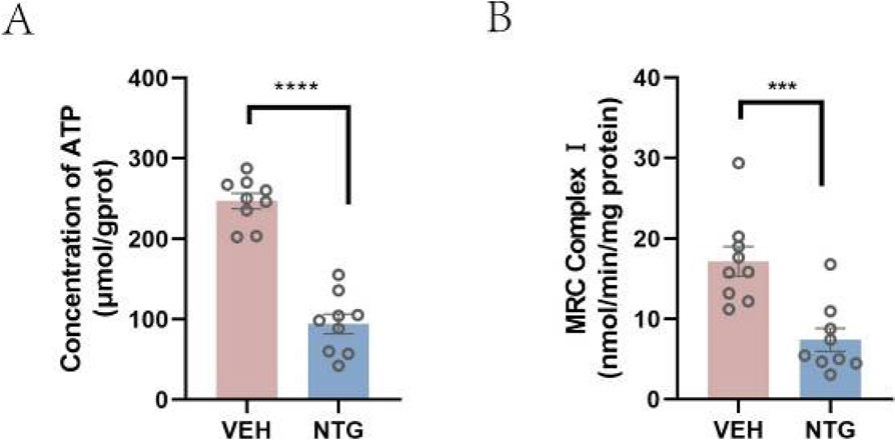
See text for description

## P234 The thalamic ventral posterior nuclear complex involvement in the migraine cycle: a task-free fMRI analysis during nitroglycerin-induced migraine like attacks

### M. M. Pocora^1,2^, D. Martinelli^2^, R. De Icco^1,2^, M. Allena^2^, A. Bacila^2^, G. Sances^2^, A. Pichiecchio^1,2^, G. Castellazzi^2^, C. Tassorelli^1,2^

#### ^1^University of Pavia, Department of Brain and Behavioral Sciences, Pavia, Italy; ^2^IRCCS C. Mondino Foundation, Headache Science and Rehabilitation Center, Pavia, Italy

##### **Correspondence:** M. M. Pocora

*The Journal of Headache and Pain 2024,* **25(Suppl 1)**: P234

**Objective:** In this resting-state functional magnetic resonance imaging (rs-fMRI) study, we assessed the interplay between the ventroposterior thalamic nuclei complex (ventral posterolateral, VPL and ventral posteromedial, VPM) and the rest of the brain during various phases of a nitroglycerin-induced migraine like attack (NTG).

**Methods:** Ten episodic migraine patients (EM) and ten healthy controls (HC) underwent 3T MRI scans during subsequent phases of the NTG migraine attack (baseline, prodrome, full-blown attack, recovery). A seed-based correlation analysis assessed the relationship between the cortex and VPL/VPM during the different phases of the attack in EM and at pre-specified time points in HC. A comparison between each scan and the pain-free condition (baseline) was performed, also testing whether there was a correlation between mean functional connectivity (FC) and anamnestic features, namely with allodynia.

**Results:** At baseline, compared to HC, EM presented a reduced FC between the left VPL/VPM nuclei and homolateral superior and middle temporal gyrus, superior frontal gyrus and precuneus bilaterally. When considering phase-specific alterations, compared to baseline, EM presented a global loss of FC throughout the migraine cycle, involving superior frontal gyrus and cingulate gyrus during the prodromal and full-blown phases, as well as an increased FC concerning the dorsal midbrain, which persisted during recovery. The FC alterations observed during the full-blown phase failed to correlate with the presence of allodynia, demonstrating a correlation, instead, with the presence of yawning during the prodromal phase.

**Conclusion:** Migraine is a disorder of sensory processing which presents a significant involvement of the VPM/VPL nuclei complex, amplifying the perception of pain signals and contributing to the associated sensory symptoms. Indeed, in this study alterations in FC were observed in the main somatosensory areas as well as in areas appointed for emotional elaboration of pain. Interestingly, phase-specific alterations were observed with a global loss of FC in EM when compared to baseline, throughout the migraine cycle, possibly reflecting the thalamo-cortical dysrhythmia phenomena.

**Fig. 1 (Abstract P234):**
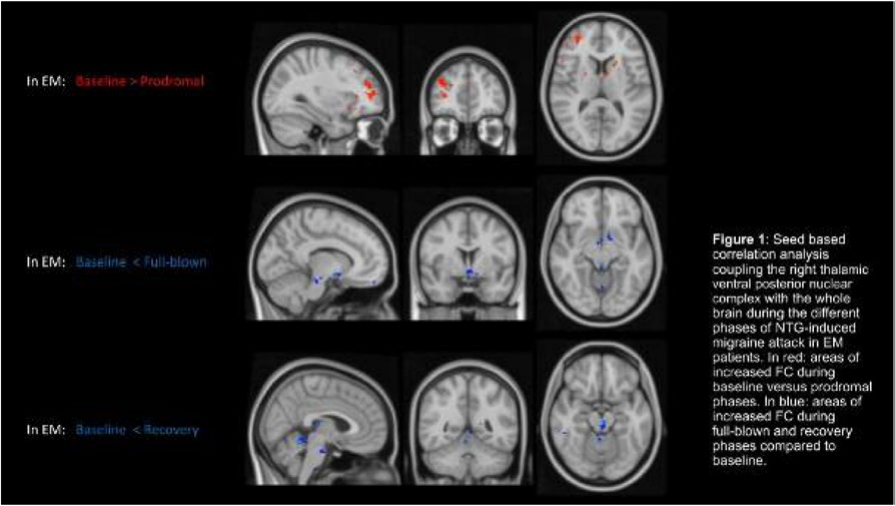
See text for description

## P236 The gut microbial features in migraine patients

### W. Tang^1^, Q. Zhang^2^, S. Yu^1^

#### ^1^Chinese PLA General Hospital, Neurology Department, Beijing, China; ^2^Chinese Academy of Sciences, Institute of Microbiology, Beijing, China

##### **Correspondence:** W. Tang

*The Journal of Headache and Pain 2024,* **25(Suppl 1)**: P236

**Objective:** To find out the real features of gut microbiota in migraine patients.

**Methods:** During the enrollment stage, to exclude the potential factors that influence the gut microbiota composition, we screened 81 migraine patients from 1264 suspicious patients and 87 healthy controls (HC). The propensity score matching method was used to adjust confounding factors of lifestyle, diet, and demography. Then, 44 migraine patients and 44 HCs were grouped as the training cohort, while the remaining subjects were grouped as the test cohort. We used 16S-rRNA sequencing and Tax4fun to detect the composition and predict the potential function of gut microbiota, respectively. Random forest was used to generate migraine classifier and to assess the importance order of microbial features. Spearman correlation analysis was used to evaluate critical relationships between clinical and gut microbial features.

**Results:** We found that two distinct microbial communities play an essential role in the gut microbiota of migraine, one with two upregulated families, Prevotellaceae and Veillonellaceae, and the other with two downregulated families, Lachnospiraceae and Ruminococcaceae; over 90% differential expressed taxa were from these four families. Thirty two differential expressed taxa on the top of the importance list composed a random forest classifier with 82.5% AUC in the training cohort and 91% AUC in all the enrolled participants. The most influential taxa OTU726 from Butyricimonas, was negatively correlated with headache frequency by the Spearman correlation analysis (*r* = 0.15, *p* < 0.05). The Tax4Fun analysis showed that starch metabolism and polysaccharide transporters in both the ABC and phosphotransferase systems were suppressed, while energy metabolism including oxidative phosphorylation and citrate cycle were upregulated.

**Conclusion:** We first identified a combinatorial gut microbial model capable of discriminating migraine from HCs. The dysbiosis of gut microbiota may affect the enery balance in the migraine hosts.

**Fig. 1 (Abstract P236):**
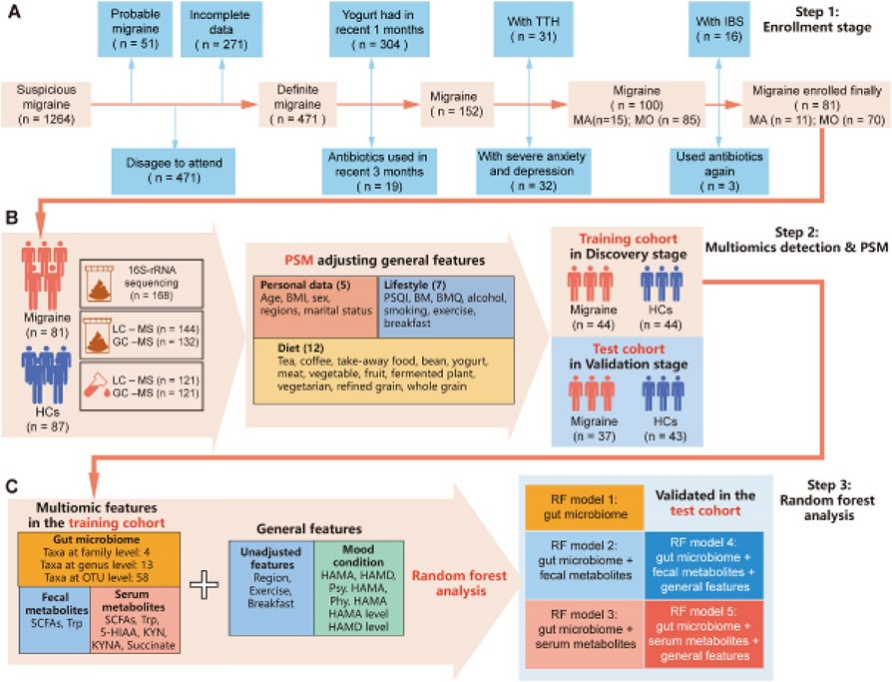
See text for description

**Fig. 2 (Abstract P236):**
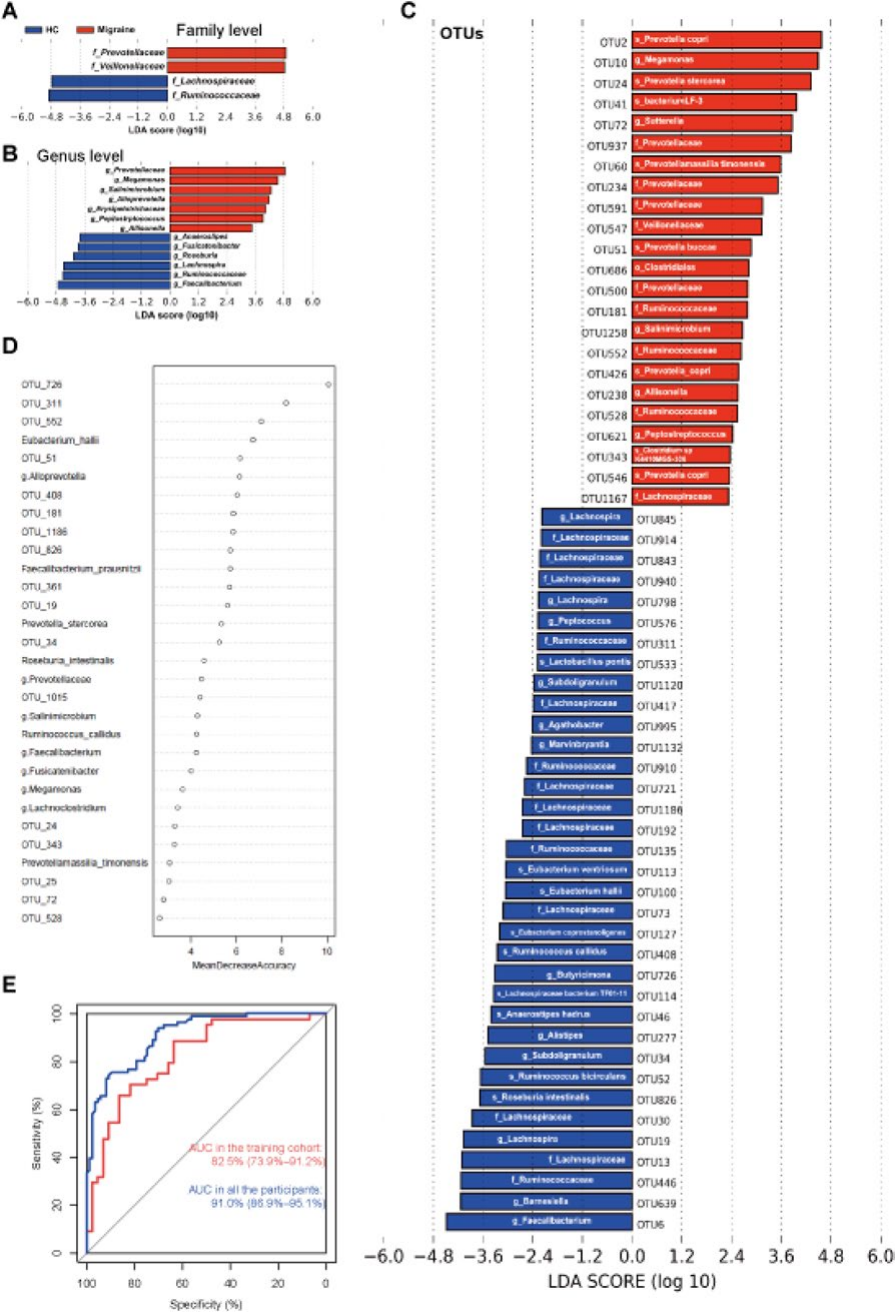
See text for description

**Fig. 3 (Abstract P236):**
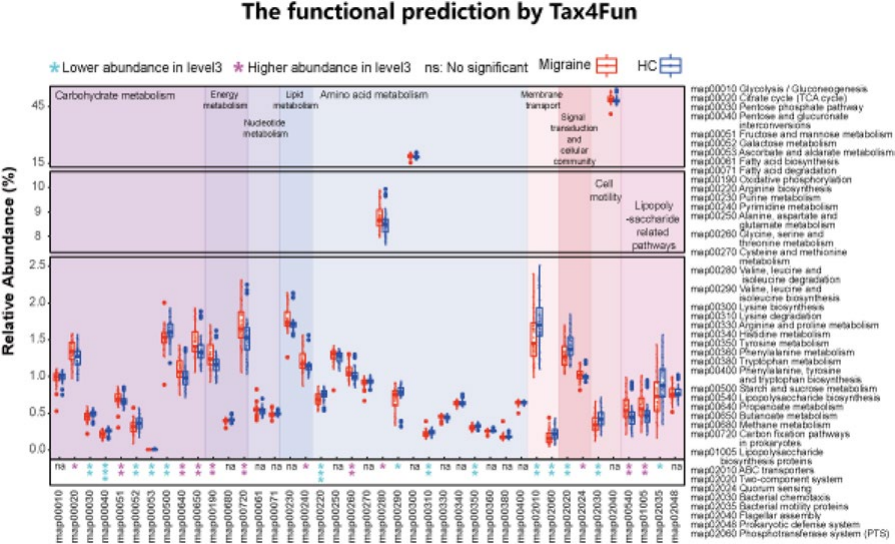
See text for description

## P237 Introduction of a standardised interview to identify post-traumatic headache

### H. Lyons^1,2^, M. Sassani^1,2^, J. Mitchell^1,2,3^, M. Thaller^1,2^, A. Yiangou^1,2^, A. Sinclair^1,2^

#### ^1^University of Birmingham, Institute of Metabolism and Systems Research, Birmingham, United Kingdom; ^2^University of Birmingham, Birmingham, United Kingdom; ^3^Defense Medical Rehabilitation Centre, Academic Department of Military Rehabilitation, Stanford Hall, United Kingdom

##### **Correspondence:** H. Lyons

*The Journal of Headache and Pain 2024,* **25(Suppl 1)**: P237

**Objective:** Mild traumatic brain injury (TBI) can cause a range of sequalae, most commonly post-traumatic headache (PTH). The incidence of PTH varies post mild TBI, with up to 80% affected by three months and 60% by one year. PTH is usually heterogenous in nature but most commonly presents as migraine or tension-type-like. Our aim is to introduce a standardised interview to identify PTH. Our primary outcome will be to identify PTH, and secondary outcomes to identify the phenotype of PTH.

**Methods:** There is a planned recruitment of 150 patients from a UK tertiary centre hospital. Inclusion criteria includes a diagnosis of mild TBI or concussion by headache specialist or sporting physician; brain imaging within normal limits; head injury within a year; aged 17 years and older. Exclusion criteria includes those with serious underlying pathology; secondary causes of headache (excluding PTH), non-English speakers, moderate or severe TBI or an abnormal brain scan. The non-headache specialist will phone the patient to run through the structured headache interview. Following this, the headache specialist will conduct a telephone clinical consultation as "gold-standard". Both interviewers will define PTH as definite (≤7 days), probable (8 – 30 days) and unlikely (>30 days).

**Results:** A total of 119 people have been screened from concussion clinics at present, with 55 included for interview and 33 completed paired interviews. The mean age is 25, with 79% male, and sport being the most common modality of injury at 94%. We are not sufficiently powered at present to conduct the inter-agreement rate, but using PABAK there is an agreement rate for PTH diagnosis of 0.87 (very good), and sensitivity agreement between migraine and tension-type headache is 0.76 (good).

**Conclusion:** We will be aiming to recruit more participants to standardize an interview to diagnose PTH. The development and validation of the classification interview for PTH has yielded promising results. The level of agreement was very good for PTH, and good for its sub-phenotypes. There is currently no standardised interview within our institution, that aids health care professionals with identifying post-traumatic headache and its sub-phenotypes.

## P238 A multi-centre, real-world study of migraine-like headache in mitochondrial diseases

### C. Yan^1,2^, S. Zhang^2^, H. Zhou^3^, Y. Liu^2^, S. Wu^2^, S. Yu^1,2^

#### ^1^Nankai University, School of Medicine, Tianjin, China; ^2^The Chinese People’s Liberation Army (PLA) General Hospital, Department of Neurology, Beijing, China; ^3^The Chinese People’s Liberation Army (PLA) General Hospital, Department of Ophthalmology, Beijing, China

##### **Correspondence:** C. Yan

*The Journal of Headache and Pain 2024,* **25(Suppl 1)**: P238

**Objective:** A retrospective study was conducted to analyse migraine-like headache (MLH) prevalence, characteristics and influencing factors in patients of multiple mitochondrial diseases (MDs).

**Methods:** This study recruited genetically confirmed 48 MDs patients from 7 centres between January 2020 and March 2023, including 29 mitochondrial myopathy, encephalopathy, lactic acidosis and stroke-like episodes (MELAS), 7 chronic progressive external ophthalmoplegia (CPEO) and CPEO+, 4 mitochondrial encephalomyopathy, 3 Leigh syndrome (LS), 2 mitochondrial encephalopathy, 2 mitochondrial myopathy and 1 myoclonic epilepsy with ragged red fibres (MERRF) cases. A structured scale was applied to collect demographic information and clinical information including headache characteristics (formulated according to ICHD-3) through clinicians' on-site or telephone interviews and clinical assessment records. At least two neurologists diagnosed and evaluated headache types. MLH prevalence was analysed by comparing with migraine prevalence data from a door-to-door survey of the general population aged 18-65 years in China.

**Results:** The one-year prevalence of MLH was 47.9% (95% CI: 34.5%-61.7%) in MDs patients, which was significantly higher than the one-year prevalence of migraine in the general population (*P*<0.001). Univariate regression analyses suggested that m.3243A>G locus mutation (*P*=0.043), epilepsy (*P*=0.004), acute phase of neurological impairment with fever (*P*=0.019), resting state blood lactate concentration (*P*=0.016), and cranial MRI showing occipital lobe involvement (*P*=0.011) were items associated with the occurrence of MLH, multiple regression analysis suggested that epilepsy and high rest blood lactate concentrations were independent risk factors for the occurrence of MLH in this cohort of MDs patients (*P*=0.038 and =0.027, respectively).

**Conclusion:** In this cohort of MD patients, the one-year prevalence of MLH was significantly higher than that of migraine in the general population; Epilepsy and high rest blood lactate concentrations were independent risk factors for the occurrence of MLH; Migraine may be directly related to abnormalities in CNS mitochondrial function and energy metabolism.

## P239 Pain thresholds in patients with migraine assessed by quantitative sensory testing

### K. A. Ebner, F. Burguet, N. Cerdá-Fuertes, T. Sprenger, A. Gantenbein, J. Kuhle, A. Papadopoulou

#### University Hospital of Basel, Neurology, Basel, Switzerland

##### **Correspondence:** K. A. Ebner

*The Journal of Headache and Pain 2024,* **25(Suppl 1)**: P239

**Objective: Background** Cutaneous allodynia and hyperalgesia are described in migraine, indicating central sensitization. However, quantitative sensory findings vary widely among studies, stimuli and locations. Moreover, it is unclear if these symptoms might be predictors of treatment response.

**Objective** In our prospective study, we aim at characterizing the somatosensory profile in migraine and its potential role in predicting response to calcitonin gene-related peptide (CGRP)-antibodies.

**Methods:** From our ongoing study, we report baseline data from quantitative sensory testing (QST). Migraine patients (according to ICHD-3) with ≥8 monthly migraine days were recruited in Basel. QST was performed according to a standardized protocol on the hand and face at baseline (before treatment initiation; one side per patient). Pain thresholds for: i) cold, ii) heat, iii) mechanical (pinprick-) and iv) pressure stimuli (PPT), as well as v) mechanical pain sensitivity and vi) dynamic mechanical allodynia (DMA) were assessed.

**Results:** 18 patients underwent QST at baseline (14/18 women, 44.2±14.3 y, 10/18 with chronic migraine, 4/18 with aura). All patients had at least one abnormal QST-finding, 14 (78%) on both face and hand, 2 (11%) only on the face and 2 only on the hand. Decreased PPT was the most common abnormal finding (13/18=72% face, 9/18= 50% hand), followed by DMA to light touch (10/18=56% face, 11/18=61% hand). Thermal pain- and mechanical thresholds were normal in most patients (4/18 and 5/18 with abnormalities, repsectively). So far we collected 6-month data of 3/18 patients, thus analysis for prediction of response is pending.

**Conclusion:** QST abnormalities –particularly pressure hyperalgesia and allodynia to light touch- are very common in patients with frequent migraine, not only in the trigeminal area, but also in the hand. Preliminary data on their predictive value in treatment response to CGRP-antibodies will be also available within the next months.

## P129 Worst Headache Neurocriptococcosis in an immunocompetent patient: a case report

### E. Melhado, L. C. Guerra, N. Della Matta, L. Buzzo do Amaral, C. Alexandra Ferro, L. Estrela Thomé, M. Freitas Martins, N. Prando, A. Haddad de Souza

#### UNIFIPA, Neurology, Catanduva, Brazil

##### **Correspondence:** E. Melhado

*The Journal of Headache and Pain 2024,* **25(Suppl 1)**: P239

**Objective:** To report a case of neurocriptococcosis in an immunocompetent patient.

**Methods:** This is a descriptive study, through the analysis of the medical record.

**Results:** Female, 23 years old, healthy, with intense headache for 15 days, pulsatile and clenching. Initial neurological examination was uneventful. A CT scan of the skull was performed, no changes, CSF was collected which showed nucleated cells 204, presence of yeast, glucose 46, proteins 28, chloride 112, VDRL: non-reactive and culture for Cryptococcus gattii, positive China ink. Brain MRI showed hypersignal on T2/FLAIR images affecting the cortical sulci, encephalic fissures, cerebellar foliae and on the pial surface of the brainstem in association with diffuse leptomeningeal. Volumetric reduction of the supratentorial ventricular system, findings of leptomeningitis of fungal nature, related to cryptococcosis. Treatment was started with amphotericin B and fluconazole. She still presented with refractory headache, requiring serial CSF punctures for relief of intracranial hypertension. She evolved with instability and underwent a right ventriculoperitoneal shunt. Postoperatively, she presented left hemiparesis. Skull CT showed hematoma and emphysema of adjacent extracranial soft tissues; right frontoparietal brain lesion and edema of adjacent parenchyma. She evolved with clinical instability and died after 61 days of hospitalization.

**Conclusion:** This case showed a pattern of CSF hypotension due to compression of the frontal horns of the lateral ventricles by the granulomas. Several repeated punctures were performed and a ventriculoperitoneal shunt was required, but the patient had fatal complications.

*Disclosure statement*: Informed consent to publish this case study and its potentially identifiable information of the patient was obtained from the individual involved. The patient gave explicit permission for the publication of this case report, including any relevant clinical details.

## P240 Hemorrhagic reversible cerebral vasoconstriction syndrome in korean woman: a case report

### K. T. Kim, B. H. Cho

#### Korea University Anam Hospital, Department of Neurology, Seoul, South Korea

##### **Correspondence:** K. T. Kim

*The Journal of Headache and Pain 2024,* **25(Suppl 1)**: P240

**Objective:** Reversible cerebral vasoconstriction syndrome (RCVS) is a group of conditions typically heralded by thunderclap headache associated with reversible segmental multifocal cerebral artery vasoconstriction. We report a Korean woman patient of RCVS with hemorrhagic complication that showed stepwise aggravation.

**Methods:** A 53-year-old woman visited emergency room presenting with thunderclap headache. Initial brain computed tomography (CT) revealed subarachnoid hemorrhage (SAH) in the left frontal sulci. Brain CT angiography showed focal mild liminal narrowing at M2 segment of left middle cerebral artery. As patient’s headache relieved after conservative treatment, she was discharged from the emergency room. Three days later, she revisited emergency room with aggravated headache and vomiting.

**Results:** CT showed newly developed right frontal intracerebral hemorrhage (ICH) combined with subdural hemorrhage (SDH). Brain CT angiography (CTA) imaging showed multifocal stenosis in bilateral middle cerebral arteries. On admission, she was alert, but mild frontal dysfunction was noted. Diagnostic tests including vasculitis lab, RNF 213 gene mutation were negative. To prevent further expansion, we controlled blood pressure in tight below 140/90mmHg. Continuous nimodipine was applied to prevent the aggravation of vasospasm. Digital subtraction angiography revealed multi-segmental narrowing in bilateral middle cerebral arteries and anterior cerebral arteries. Nimodipine infusion at the terminal internal carotid artery partially enlarged the vessel diameter. She was diagnosed as hemorrhagic reversible cerebral vasoconstriction syndrome (RCVS). With successful conservative management, lobar intracranial hemorrhage decreased in time and no new intracranial hemorrhage developed.

**Conclusion:** We report a case of hemorrhagic RCVS in Korean woman. Although the pathophysiology of this syndrome is not well established, clinicians should consider vasoconstriction as the cause of intracranial hemorrhage, not only as the result.

*Disclosure statement:* Informed consent to publish this case study and its potentially identifiable information of the patient was obtained from the individual involved. The patient gave explicit permission for the publication of this case report, including any relevant clinical details.

## P242 Migraine and risk of atrial fibrillation. A 9-year follow-up based on the Trøndelag health study

### S. Giri^1,2^, E. Tronvik^1,2,3^, H. Dalen^4^, H. Ellekjær^1^, J. P. Loennechen^4^, A. Olsen^2,5^, K. Hagen^1,2,6^

#### ^1^Norwegian University of Science and Technology, Department of Neuromedicine and Movement Science (INB), Trondheim, Norway; ^2^NorHEAD-Norwegian centre for Headache Research, Trondheim, Norway; ^3^Norwegian Advisory Unit on Headache , St. Olavs Hospital, Department of Neurology and Clinical Neurophysiology, Trondheim, Norway; ^4^Norwegian University of Science and Technology, Department of Circulation and Medical Imaging, Trondheim, Norway; ^5^Norwegian University of Science and Technology, Department of Psychology, Trondheim, Norway; ^6^Clinical Research Unit Central Norway, St. Olavs University Hospital, Trondheim, Norway

##### **Correspondence:** S. Giri

*The Journal of Headache and Pain 2024,* **25(Suppl 1)**: P242

**Objective:** To investigate association between primary headache disorders and the risk of atrial fibrillation in a population-based cohort study.

**Methods:** In a population-based 9-year follow-up design, we evaluated questionnaire-based headache diagnoses, including migraine and tension-type headache (TTH), collected in the Trøndelag Health Study (HUNT3) between 2006-2008, and the subsequent risk of AF in the period until December 2015. The population at risk consisted of 39,340 individuals ≥20 years of age without AF, who answered the headache questionnaire during HUNT3. Prospective associations were evaluated by multivariable Cox proportional hazard models with 95% confidence intervals (CIs).

**Results:** Among the 39,340 participants, a total of 1524 (3.8%) developed AF during the 9-year follow up, whereof 91% of these were ≥55 years. In the multivariate analyses, adjusting for known confounders, we did not observe any association between migraine or TTH and risk of AF. The adjusted hazard ratios (HRs) were 0.84 (95% CI, 0.64-1.11) for migraine, 1.16 (95% CI, 0.86-1.27) for TTH, and 1.04 (95% CI, 0.86-1.27) for unclassified headache. However, in sensitivity analyses using age ≥55 years as cutoff, a lower risk of AF was found for migraine (HR 0.53, 95% CI, 0.39-0.73). No significant difference was observed among men and women.

**Conclusion:** In this large population-based study, no increased risk of AF was observed among individuals with migraine or TTH at baseline. In fact, among individuals aged ≥55 years, migraine was associated with a lower risk for AF.

## P243 Is there a link between migraine and stroke? A population-based register-linked cohort study

### S. Giri^1,2^, E. Tronvik^1,2,3^, H. Dalen^4^, H. Ellekjær^1^, J. P. Loennechen^4^, A. Olsen^2,5^, K. Hagen^1,2,6^

#### ^1^Norwegian University of Science and Technology, Department of Neuromedicine and Movement Science (INB), Trondheim, Norway; ^2^NorHEAD-Norwegian centre for Headache Research, Trondheim, Norway; ^3^Norwegian Advisory Unit on Headache , St. Olavs Hospital, Department of Neurology and Clinical Neurophysiology, Trondheim, Norway; ^4^Norwegian University of Science and Technology, Department of Circulation and Medical Imaging, Trondheim, Norway; ^5^Norwegian University of Science and Technology, Department of Psychology, Trondheim, Norway; ^6^Clinical Research Unit Central Norway, St. Olavs University Hospital, Trondheim, Norway

##### **Correspondence:** S. Giri

*The Journal of Headache and Pain 2024,* **25(Suppl 1)**: P243

**Objective:** To evaluate whether primary headache disorders, including subtypes of migraine, increase the risk of stroke.

**Methods:** This population-based 15-year follow-up study used baseline headache data from the third Nord-Trondelag Health Survey (HUNT3) performed between 2006 and 2008. The headache HUNT3 data were linked to the Norwegian National Stroke Register that includes stroke diagnoses recorded from 2012 until December 2021. The association between stroke and headache status was investigated in 37,364 individuals, with ≥20 years of age without stroke diagnosis, who answered headache questionnaire during HUNT3. Prospective associations were evaluated using multivariable Cox proportional hazard models with 95% confidence intervals (CIs). Separate sub-group analyses by age and sex were performed.

**Results:** Among 37,364 included participants, 1,095 (2.9%) developed stroke, whereof 13.4% were younger than 55 years. In the multi-adjusted model, reporting migraine with aura (MA) at baseline was associated with increased risk of stroke at follow-up (HR 1.55, 95% CI 1.16-2.08) compared with those reporting no headache. The risk of stroke was much higher among individuals with MA who were <55 years old (HR 1.98, 95% CI 1.20-3.27) and were women (HR 1.64, 95% CI 1.12-2.41).

**Conclusion:** Individuals with MA were more likely to have new onset of stroke compared to persons without headache. The relationship with MA was even stronger in women, and for young individuals aged

## P244 The prevalence of contraindications to triptans and cardiovascular risk in adults with migraine in the UK: a retrospective cross-sectional study

### G. O'Neil

#### Pfizer, Medical Affairs, Tadworth, United Kingdom

*The Journal of Headache and Pain 2024,* **25(Suppl 1)**: P244

**Objective:** To examine the prevalence of patient-reported triptan contraindications and cardiovascular risk factors in people with migraine in the United Kingdom (UK).

**Methods:** A retrospective cross-sectional study from the 2020 National Health and Wellness Survey (NHWS, Cerner Enviza) captured patient-reported data from adults ≥18 years old with migraine in the UK. The migraine subgroup eligibility criteria required a physician diagnosed their migraine. Triptan contraindications in the survey included the self-reported presence of angina, arrhythmias, atrial fibrillation, heart failure, myocardial infarction, left ventricular hypertrophy, transient ischemic attacks, stroke, and peripheral vascular disease. Cardiovascular risk factors included hypertension, dyslipidaemia, current smoking, type 2 diabetes, and obesity (BMI ≥30), with ≥2 risk factors deemed "high-risk." NHWS weights were used in all analyses to make the survey sample generalisable to the adult population in the UK based on age and sex.

**Results:** Among 5.4 million adults with migraine represented by the weighted survey (*n*=1611), 12.96% had a contraindication to triptans and 24.65% had ≥2 cardiovascular risk factors. Combined, 32.21% of people with migraine had a contraindication to triptans or ≥2 cardiovascular risk factors. The most common contraindication to triptans was unstable angina/chest pain (see Figure 1), whilst the most common cardiovascular risk factor among people with migraine in the UK was obesity (see Figure 2).

**Conclusion:** The prevalence of triptan contraindications and the presence of ≥2 cardiovascular risk factors were common, with 1 out of every 3 migraine patients in the UK meeting these criteria.

**Fig. 1 (Abstract P244):**
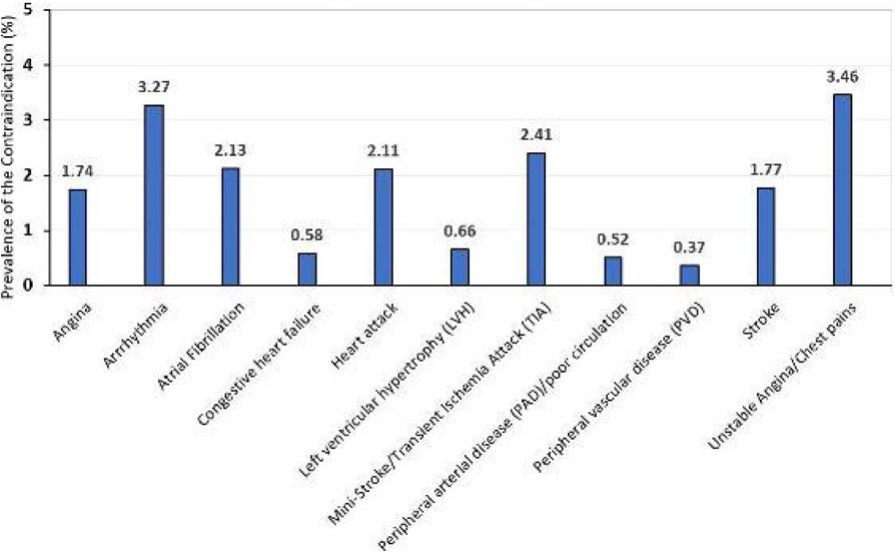
Patient-Reported Cardiac Contraindicatons to Triptans (%) Among People with Migrane in the United Kingdom

**Fig. 2 (Abstract P244):**
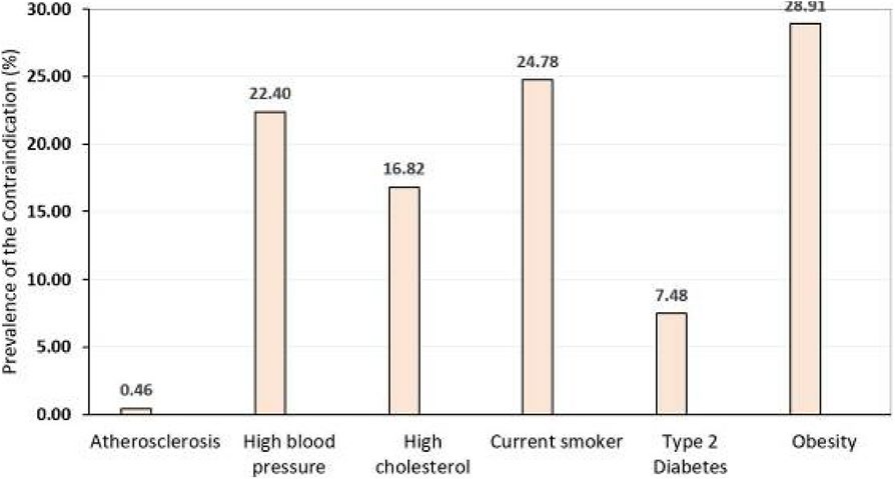
Patient-Reported Cardiovascular Risks Factors (%) Among People with Migrane in the United Kingdom

## P245 Sex-specific association between migraine and lipid levels: results from the LifeLines cohort study

### D. Boucherie, L. Al-Hassany, A. H. J. Danser, A. MaassenVanDenBrink

#### Erasmus MC University Medical Center, Internal Medicine, division of Vascular Medicine and Pharmacology, Rotterdam, Netherlands

##### **Correspondence:** D. Boucherie

*The Journal of Headache and Pain 2024,* **25(Suppl 1)**: P245

**Objective:** Migraine is associated with major cardiovascular disease. Yet, the association between cardiovascular risk factors, in particular lipid levels, and migraine is disputed. This heterogeneity might be attributed to the modifying impact of sex on the association between lipid levels and migraine. While most studies adjust for sex, we hypothesized that sex rather serves as an effect modifier in the association between lipid levels and migraine. Therefore, we studied the association between migraine and lipid profiles in males and females separately.

**Methods:** The association between total cholesterol (TC), triglycerides (TG), low-density lipoprotein cholesterol (LDL-C), high-density lipoprotein cholesterol (HDL-C) (all in mmol/L) and migraine was studied in the Dutch population-based Lifelines cohort, consisting of 142,769 adults with a known, self-reported (history of) migraine and complete lipid profile at baseline. Logistic regression analyses with adjustments for age, body mass index, educational level, current smoking, dietary intake, and physical activity were performed for each lipid profile. All analyses were stratified by sex.

**Results:** In total, 26,307 (18.4%) current or past migraine patients (median age 44 years [IQR 36–51]) were analyzed. Compared with males without migraine history, males with current or past migraine had modestly increased odds ratios (ORs) [95% CI] for elevated lipid levels of 1.05 [1.02–1.08] for TC, 1.05 [1.02–1.08] for TG, 1.07 [1.03–1.11] for LDL–C, and an OR of 0.68 [0.62–0.76] for HDL-C. Compared with females without migraine history, females with current or past migraine had modestly increased ORs [95% CI] for elevated lipid levels of 1.03 [1.01–1.05] for TC, 1.08 [1.05–1.12] for TG, 1.04 [1.02–1.06] for LDL–C, and an OR of 0.88 [0.83–0.92] for HDL-C.

**Conclusion:** In a generally healthy population and without distinguishing between migraine patients with and without aura, we observed a slightly unfavorable lipid profile in migraine patients without evident sex differences. However, we noted a more pronounced association with decreased levels of HDL-C, especially among males.

## P246 Migraine in patients with preeclampsia: prevalence and correlation with angiogenic factors

### L. Melgarejo Martinez^**1**^, L. Gómez-Dabó^1^, J. Rosell-Mirmi^1^, A. Alpuente^1^, M. Torres-Ferrús^1^, M. Mendoza Cobaleda^2^, E. Caronna^1^, P. Pozo-Rosich^1^

#### ^1^Vall d’Hebron Hospital & Research Institute, Universitat Autonoma de Barcelona, Neurology, Barcelona, Spain; ^2^Vall d’Hebron Hospital & Research Institute, Universitat Autonoma de Barcelona, Gynecology and Obstetrics, Barcelona, Spain

##### **Correspondence:** L. Melgarejo Martinez, L. Gómez-Dabó, E. Caronna

*The Journal of Headache and Pain 2024,* **25(Suppl 1)**: P246

**Objective:** To analyze the prevalence of migraine in a cohort of patients with preeclampsia (PE) and to determine its relationship with angiogenic factors.

**Methods:** This is a retrospective study in a cohort of patients with PE who were attended in a tertiary Spanish hospital from February/2016 to July/2021. Demographic, clinical, laboratory data including angiogenic factors (sFlt-1, PlGF and sFlt-1/PlGF index) and the APGAR score were collected from medical charts. Through a phone interview, two neurologists confirmed previous history of migraine. We compared patients with and without previous history of migraine.

**Results:** We included 223 patients. 39.6% (83/223) with migraine [EC1] (74/83 with episodic and 9/83 with chronic migraine) and a mean age of 33.5 ±6.3 years. When comparing patients with and without previous history of migraine: there were no significant differences in baseline characteristics [EC2] , symptoms of PE or blood pressure values of PE diagnosis. 38.1% (85/223) had headache as the principal symptom of PE. Regarding analytical parameters, the 24-hour proteinuria values, and the index sFlt-1/PlGF were discreetly higher in patients with migraine but did not reach statistical significance. We did not find greater fetal complications in the group of patients with migraine.

**Conclusion:** The prevalence of migraine is higher in patients with PE than in the general population. Even though the vascular system plays an important role in migraine pathophysiology, alterations in angiogenic factors, as seen in PE, are not different if they have migraine or not.

## P247 Measuring the prevalence of cardiovascular risk and contraindications to triptan therapy among adults with migraine from five European countries in the national health and wellness survey

### A. Gendolla^1^, J. Brown^2^, A. Jenkins^2^, K. Hygge Blakeman^2^, L. Abraham^2^, N. Sternbach^3^, A. Mercadante^3^, S. Drakeley^3^, R. B. Lipton^4^

#### ^1^Praxis Gendolla, Essen, Germany; ^2^Pfizer, Inc., New York, NY, United States; ^3^Cerner Enviza, Kansas City, MO, United States; ^4^Montefiore Medical Center and Albert Einstein College of Medicine, Bronx, MA, United States

##### **Correspondence:** J. Brown

*The Journal of Headache and Pain 2024,* **25(Suppl 1)**: P247

**Objective:** To examine the prevalence of triptan contraindications and cardiovascular risk factors in five European countries (France, Germany, UK, Italy, and Spain).

**Methods:** This was a retrospective, cross-sectional study of individuals from the 2020 EU National Health and Wellness Survey (NHWS, Cerner Enviza). Triptan contraindications (either explicitly mentioned in labels or directly related) in the survey included angina, arrhythmias, atrial fibrillation, heart failure, myocardial infarction, left ventricular hypertrophy, transient ischemic attacks, stroke, and peripheral vascular disease. Cardiovascular risk factors included hypertension, dyslipidemia, current smoking, type 2 diabetes, and obesity (BMI ≥30), with ≥2 risk factors deemed "high-risk." The weighted prevalence of triptan contraindications, cardiovascular risk factors, migraine headache days, migraine burden using the Migraine Disability Assessment Test (MIDAS), and current migraine treatments were summarized.

**Results:** Among 30.5 million adults in Europe with physician-diagnosed migraine represented by the weighted survey, 17.1% had a triptan contraindication and 25% had ≥2 cardiovascular risk factors. Combined, 35% of the migraine cohort had a triptan contraindication or ≥2 cardiovascular risk factors (Figure). Migraine burden was higher in those with cardiovascular conditions, with 29% vs. 21% reporting severe and 18% vs. 15% reporting moderate disability using MIDAS (*p*<0.05). Current triptan treatment was reported by 28% of those who reported at least one contraindication to triptan therapy and 25% of those with ≥2 cardiovascular risk factors compared to 34% of those without any of these conditions.

**Conclusion:** One out of every three migraine patients in five European countries had a triptan contraindication or ≥2 cardiovascular risk factors.

## P248 Hypertensive posterior reversible leukoencephalopathy presenting as migraine like headache

### S. Zamanian

#### Social Security organization mashhad, Mashhad, Iran

*The Journal of Headache and Pain 2024,* **25(Suppl 1)**: P248

**Objective:** Posterior reversible encephalopathy syn-drome with spinal cord involvement (PRES-SCI) is a rare entity with only about 15 cases being reported .The Aim is to present a case of 7-year-old girl who presented with complaints of migraine like headache and was later found to be hypertensive with features of PRES-SCI.

**Methods:** A 7-year-old girl presented with recurrent episodic, bilateral frontotemporal throbbing headache since 3 months. Headaches occurred every 3-4 days, lasting for 2-3 hours with nausea, vomiting, vertiginous sensations and phonophobia and used to subside after a bout of vomiting or sleep. There was no significant past history. Her initial evaluation by a GP revealed no abnormality including fundus examination. However, no record of her BP measurement was available. She was referred to our centre as her headaches became continuous for the Dast 7 days. On examination, patient was conscious but jittery. She was well oriented but her sustained attention was impaired. Her pulse was 106/min and BP 240/130 mm. Eye examination showed bilateral grade-4 hypertensive retinopathy with bilateral exudative retinal detachment. Neurological examination revealed bilateral hyper-reflexia, dysdiadochokinesia and impaired tandem gait. Planters were flexors. A diagnosis of malignant hypertension with hypertensive encephalopathy was entertained which was treated immediately with tablet Amlodipine followed by addition of Clonidine. MRI brain showed patchy areas of signal alteration iso-intense to hypo-intense signals involving cortical, sub-cortical white matter.

**Results:** Over next 6 weeks, her BP normalized on treatment and repeat MRI brain and spine became absolutelynormal thereby confirming the diagnosis of PRES-SCI. Her headaches improved dramatically.

**Conclusion:** BP measurement should be an integral part of headache evaluation even in young children. PRES-SCI although rare can present with migraine like headaches.

*Disclosure statement*: Informed consent to publish this case study and its potentially identifiable information of the patient was obtained from the individual's guardian. The patient's guardian gave explicit permission for the publication of this case report, including any relevant clinical details.

## P249 Migraine-like headache in subjects with isolated Lambl"s excrescences: a case series and literature review

### W. Xie^1^, R. Li^2^, C. Li^1^, R. Liu^1^, S. Yu^1^

#### ^1^The First Medical Center of Chinese PLA General Hospital, Department of Neurology, Beijing, China; ^2^The First Medical Center of Chinese PLA General Hospital, Department of Laboratory Medicine, Beijing, China

##### **Correspondence:** W. Xie

*The Journal of Headache and Pain 2024,* **25(Suppl 1)**: P249

**Objective:** Lambl"s excrescences are mobile, thin, fibrinous connective tissue strands typically found on left-sided cardiac values. Migraine is positively associated with structural cardiac anomalies. However, it remains unclear whether Lambl"s excrescences are associated with migraine.

**Methods:** Retrospective review of 182 inpatients with Lambl"s excrescences confirmed by transesophageal echocardiogram in Chinese PLA General Hospital since January 2010. Among them, those with isolated Lambl"s excrescences presented with migraine-like headache were included. We collected information on the demographics and clinical profiles of all participants, and performed follow-up visits.

**Results:** A total of 8 patients presented with migraine-like headache among 15 patients with isolated Lambl"s excrescences. They included 2 men and 6 women, with an average age of 44.63±12.24 years. Among these patients, 3 had visual aura, and 6 manifested infarct-like lesions on magnetic resonance imaging, of which 2 developed lesions after first visit. During follow-up, 4 patients suffering from intervention for Lambl"s excrescences dramatically reduced headache recurrence compared to the other 4 patients only receiving migraine preventive medications.

**Conclusion:** This study supports the hypothesis that microemboli from isolated Lambl"s excrescences could cause migraine-like headache. And intervention for Lambl"s excrescences may be crucial for preventing headache recurrence.

**Fig. 1 (Abstract P249):**
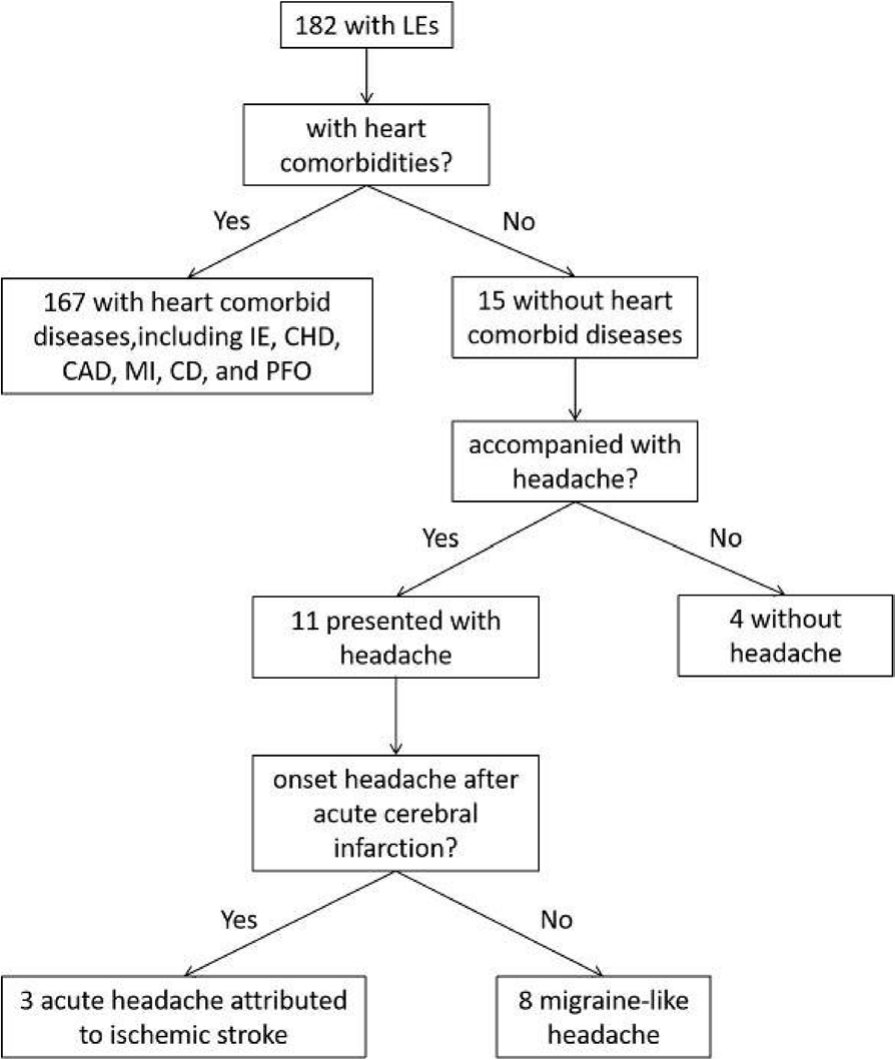
See text for description

**Fig. 2 (Abstract P249):**
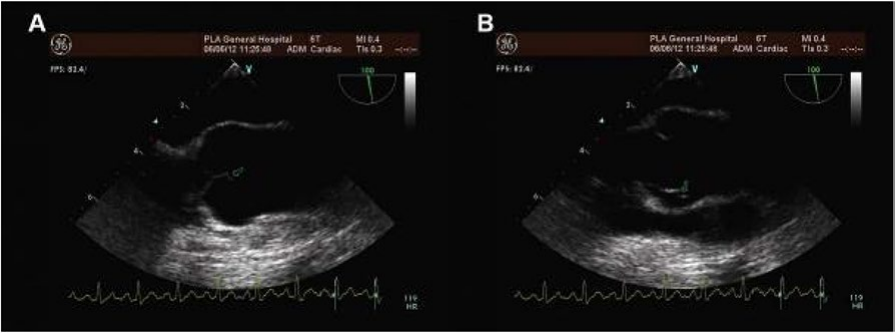
See text for description

**Fig. 3 (Abstract P249):**
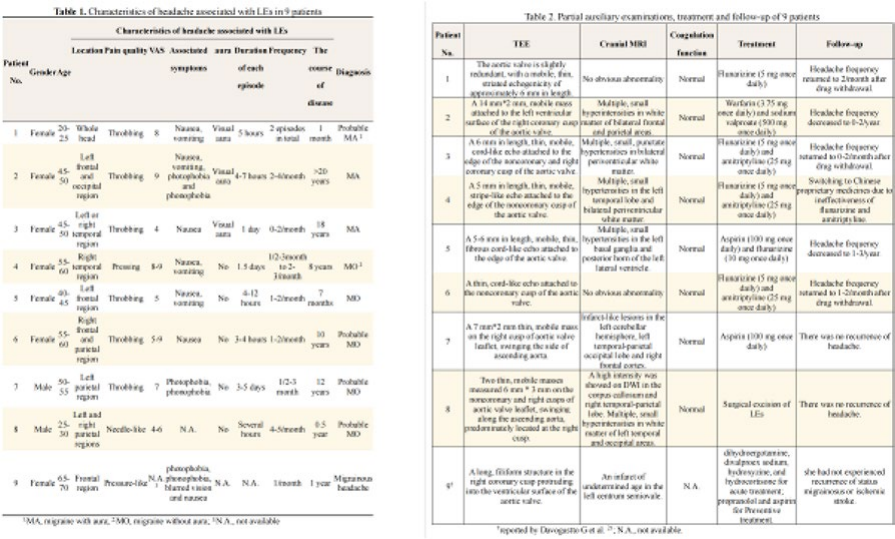
See text for description

## P250 Migrainous infarction in a young woman: a case report

### E. R. Lebedeva^1^, N. M. Gurary^2^, J. Olesen^3^

#### ^1^The Ural State Medical University, International Headache Center "Europe-Asia", Yekaterinburg, Russian Federation; ^2^Medical Union “New Hospital”, Yekaterinburg, Russian Federation; ^3^Danish Headache Center, Copenhagen University Hospital – Rigshospitalet Glostrup, Copenhagen, Denmark

##### **Correspondence:** E. R. Lebedeva

*The Journal of Headache and Pain 2024,* **25(Suppl 1)**: P250

**Objective:** Migrainous infarction (MI) is a rare disorder that reportedly accounts for 0.5–1.5% of all ischemic strokes. It is more frequent in females, especially before age 50. Precise diagnostic criteria for this diagnosis are available in the International Classification of Headache Disorders (ICHD) since 1988, but many cases are not fulfilling these criteria.

**Methods:** We report a very special case of MI in 18 y.o. woman and analyze vascular risk factors.

**Results:** An 18-year-old woman (a student) was admitted to the International Headache Centre "Europe-Asia" in Yekaterinburg on April 4th, 2018. She had a recurrent episode of migraine with a similar aura with numbness of the right arm and speech disturbances which had unusually long duration (>120 minutes). On admission, she complained of slowness of speech and problems with the choice of words. MRI showed acute lacunar infarcts in the left parietal subcortical area. Ischemic infarcts were localized in a relevant area on the left side and the aura symptoms were right-sided. The patient, therefore, fulfilled the ICHD-3 diagnostic criteria for "Migrainous infarction". 24 microembolus signals in the middle cerebral artery were registered on transcranial Doppler cerebral embolus detection. We found no peripheral or central source of embolism and cardiac and extracardiac shunts and arterial disease were absent. MR-angiography of cerebral vessels and cervical MRI with fat suppression were unremarkable. Lipids, glucose, and coagulation were normal. No mutations in the NOTCH 3 gene and no antiphospholipid antibodies were detected. Vasculitis workup did not find any abnormalities. She did not have any recurrent episodes of MI or stroke during 5-years of follow-up.

**Conclusion:** It is crucially important to detect changes in clinical characteristics of headaches because they can serve as red flags warning about a causative disorder. They were indications for MRI and led to the diagnosis of migrainous infarction in the described case.

*Disclosure statement*: Informed consent to publish this case study and its potentially identifiable information of the patient was obtained from the individual involved. The patient gave explicit permission for the publication of this case report, including any relevant clinical details.

**Fig. 1 (Abstract P250):**
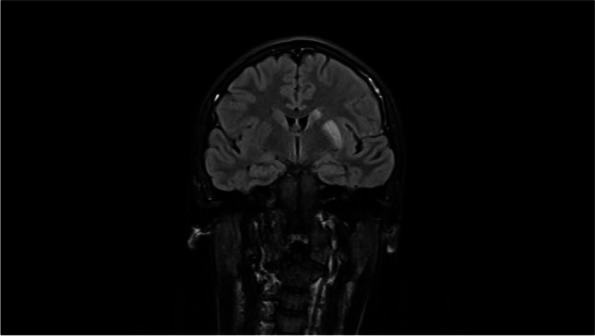
See text for description

## P155 Gut microbiota profiling of pediatric patients with migraine

### L. Papetti^1^, F. Del Chierico^2^, I. Frattale^3^, M. Scanu^2^, F. Toto^2^, S. Levi Mortera^2^, F. Ursitti^1^, G. Sforza^1^, G. Monte^1^, M. Valeriani^1^, L. Putignani^2^

#### ^1^IRCCS Bambino Gesù, Developmental Neurology Unit, Rome, Italy; ^2^IRCCS Bambino Gesù, Unit of Human Microbiome, Rome, Italy; ^3^Tor Vergata University of Rome, Child Neurology and Psychiatric Unit, Rome, Italy

##### **Correspondence:** L. Papetti

*The Journal of Headache and Pain 2024,* **25(Suppl 1)**: P155

**Objective:** We want to verify if gut microbiota (GM) in children with migraine shows differences in the profiling respect to healthy controls (HCs) and if different migraine phenotype (aura or not; presence of nausea/vomiting or photo/phonophobia during the attacks; duration of disease and frequency of monthly days with headache) are associated with differences in GM.

**Methods:** Patients aged between 6 and 18 years were recruited. The GM profiling was obtained by the 16S rRNA region sequencing from faecal samples of migraine patients (*n* = 98) and of healthy subjects (*n* = 100, HCs). Alpha and beta diversity analyses and multivariate (unsupervised Principal Component Analysis [PCA] and the supervised Partial Least Square Discriminant Analysis [PLS-DA]) and univariate (Linear Discriminant analysis [LDA] effect size [LEfSe]) tests were applied to compare the gut microbiota profiles between migraine and HC groups by R v4.0.2.

**Results:** α-diversity was not significantly different between MS and HC (*p*> 0.05). The analysis of β-diversity revealed a dissimilarity statistically significant among two groups (*p*<0.01), suggesting a different GM profile of MS compared with HCs (fig.1). Multivariate analysis evidenced the presence of GM fingerprints specific of MS and HC (fig.2A) and two differential profiles of GM for MS and HC cohorts (fig 2B). Compared to HCs, Bacteroides, Faecalibacterium, Butyricicoccuus, Lactobacillus and Enterobacteriaceae were assigned as biomarker of the patient"s microbiota, while Bifidobacterium, Akkermansia, Collinsella, Eggerthella, Clostridium, Erysipelotrichaceae, Mogibacteriaceae and Coriobacteriaceae to GM of HCs. We found no significant differences in the subgroup analyses of MS (fig.2C).

**Conclusion:** Our study shows that pediatric migraine patients have a very different GM composition compared to HCs. These differences do not appear to be related to disease characteristics such as duration, presence of aura, or neurovegetative or sensory symptoms.

**Fig. 1 (Abstract P155):**
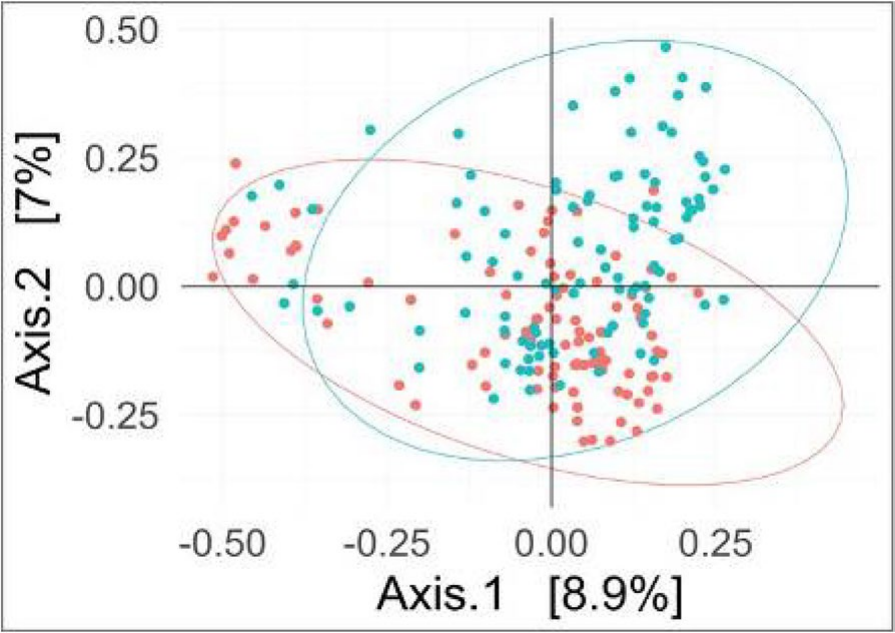
See text for description

**Fig. 2 (Abstract P155):**
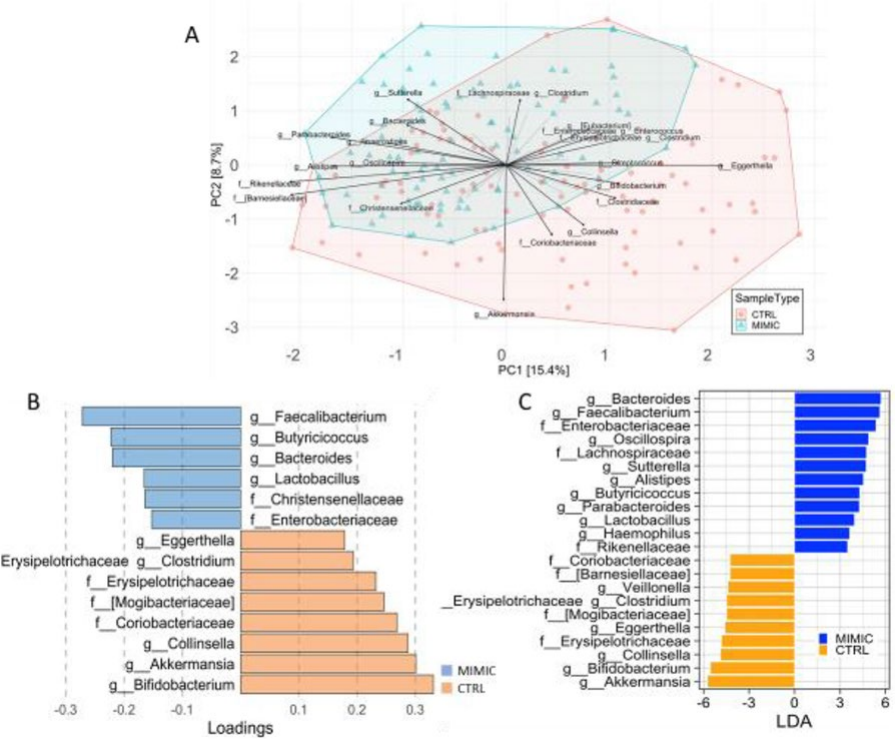
See text for description

## P251 Retrospective evaluation of fremanezumab failures in migraine patients at the Walton Centre NHS Trust

### U. K. Akberali, M. Ghadiri-Sani, S. Broadhurst

#### The Walton Centre NHS Foundation Trust, Neurology, Liverpool, United Kingdom

##### **Correspondence:** M. Ghadiri-Sani

*The Journal of Headache and Pain 2024,* **25(Suppl 1)**: P251

**Objective:** Evaluation of patients who did not respond to Fremanezumab.

**Methods:** Retrospective review of 186 patients who did not respond to Fremanezumab between November 2020 to May 2023. Parameters that were assessed include age, gender, secondary headaches, comorbidities, previous preventatives, number of crystal-clear days, side-effects (SEs), and follow-on treatments. Currently, we have over 1300 patients on Fremanezumab.

**Results:** At the time of this study, we had 1100 patients on Fremanezumab, 186 (17%) of whom stopped the treatment. 3 patients were excluded due to lack of data. Analysis of 183 patients (149 F/34 M; mean age 46) showed 5 (3%) with episodic and 178 (97%) with chronic migraine.

Only 27% of patients had crystal-clear days prior to initiating Fremanezumab. On average 6 previous preventatives were used (range 3-21), including, Botox (46%), Erenumab (4%), Galcanazemab (0.5%), and cranial nerve blocks (CNBs) (51%). 50 (27%) patients received a combination of Botox and CNBs.

72 (32%) patients had anxiety and depression, 24 (13%) restless leg syndrome, and 15 (8%) fibromyalgia. 18% had no comorbidities.

15% of patients had hemicrania continua, 9% cluster headaches but 70% had no secondary headaches.

Only 22 (12%) patients discontinued treatment due to SEs (constipation 4, site reaction 8).

Among this cohort, 102 switched to another CGRP MAB, (91 Erenumab and 11 Galcanazemab). 43 (23%) patients went onto Botox, others to alternative treatments.

**Conclusion:** Based on our experience, Fremanezumab is a highly effective and well-tolerated treatment for patients with migraine.

Our overall non-responder rate was 17%, 12% of whom stopped due to SEs (overall stop rate due to SEs was: 2%). Majority stopped due to inefficacy (88%). Factors contributing to this could include lack of crystal-clear days (73%), comorbid depression (39%), non-responders to other effective treatments, and older age.

## P252 Audit of referrals for occipital nerve blocks

### R. McGinty, M. Wazil, M. Ghadiri-Sani

#### The Walton Centre NHS Foundation Trust, Neurology, Liverpool, United Kingdom

##### **Correspondence:** R. McGinty

*The Journal of Headache and Pain 2024,* **25(Suppl 1)**: P252

**Objective:** To evaluate adherence to local guidelines for cranial nerve blocks (CNB).

**Methods:** Prospective review of CNB referrals (August – October 2022) and electronic notes; variables assessed include headache diagnosis, lifestyle measures and safety screening.

**Results:** 19 consultant neurologists referred 59 patients - 44 (74.6%) female, mean age 50. Mean number of referrals per consultant was 3 (range 1 – 10). Headache specialists accounted for 9 (15.3%) referrals. Mean wait from referral to CNB was 45 days (range 7-203 days). Headache subtypes are listed in the accompanying table. 31 referrals (52.5%) were for treatment-naïve patients. Mean number of preventatives was 3.7 (range 1 – 8). 6 (10.2%) patients never tried preventatives; 3 failed botulinum toxin and 2 fremanezumab. 19 (3%) had medication overuse headache but only 5 (8.5%) were highlighted on referral forms. 41 (69.5%) CNB referral forms were completed in full and 43 (72.9%) satisfied referral criteria. Safety-related omissions included a previous adverse drug reaction to lidocaine and a patient on apixaban.

**Conclusion:** Most referrals were complete and met Trust criteria. Headache specialists generated a minority of the referrals. Analgesic overuse was underreported. Introducing an electronic referral with mandatory fields and links to guidelines could improve compliance.

**Table 1 (Abstract P252):**
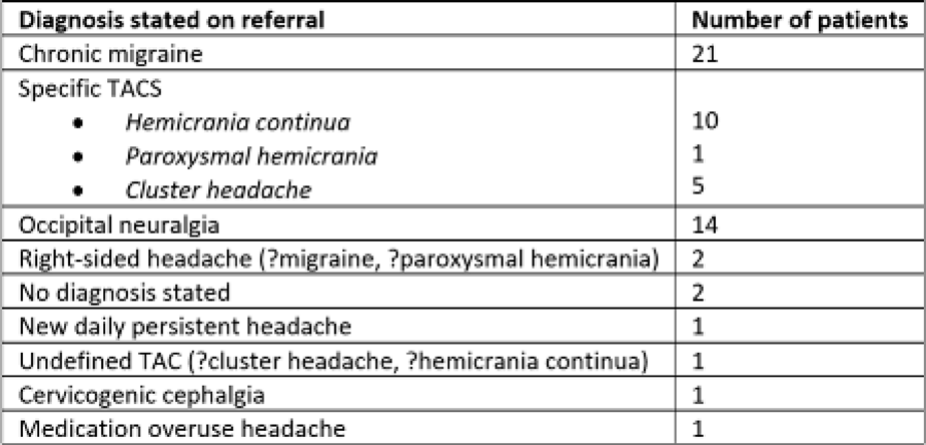
Headache subtypes

## P253 Is CGRP-induced headache sensitive predictor for anti-CGRP therapy?

### M. Zaletel, B. Žvan, G. Požlep

#### University Clinical Centre of Ljubljana, Ljubljana, Slovenia

##### **Correspondence:** M. Zaletel

*The Journal of Headache and Pain 2024,* **25(Suppl 1)**: P253

**Objective:** αCGRP induces hemodynamic changes in cerebral circulation and CGRP-induces headache (CGRP-IH), which is classified into immediate and delayed CGRP-IH (iCGRP-IH and dCGRP-IH). Study showed cerebral hemodynamic responses to CGRP. It is unclear whether iCGRP-IH and dCGRP-IH are associated with CGRP induced hemodynamic changes. Therefore, we studied the effects of αCGRP on cerebral hemodymics, iCGRP-IH, dCGRP-IH using transcranial Doppler (TCD).

**Methods:** Twenty healthy subjects participated in our study. Polymodal recording of mean arterial velocity in MCA (vm MCA) and PCA (vm PCA), end-tidal carbon dioxide partial pressure (Et-CO2), mean arterial pressure (MAP), and heart rate (HR) was monitored using TCD. During the experiment, we administered intravenous infusion of CGRP at a rate of 1.5 mcg/min. The vm MCA, Et-CO2, HR, and MAP were determined at time points T 0, T 1, T 2, and T 3. We calculated the responses at different time points. We monitored intensity of iCGRP-IH and dCGRP-IH using visual analog scale (VAS).

**Results:** We found positive association between Δvm MCA and iCGRP-IH (*p*=0.003, OR=1.18) as well as Δvm PCA and iCGRP-IH (*p*=0.002, OR=1.16). We did not significant association between Δvm MCA, Δvm PCA and dCGRP-IH (*p*=0.170, OR=1.05 and *p*=0.228, OR=1.08 respectively. Significant relationship was found between Δvm MCA and VAS of iCGRP-IH (*p*=0.012) as well as between Δvm PCA and iCGRP-IH (*p*=0.045). but not between Δvm MCA and VAS of dCGRP-IH (*p*=0.326) as well as between Δvm PCA and iCGRP-IH (*p*=0.279).

**Conclusion:** We have concluded that hemodynamics after CGRP provocation is associated with iCGRP-IH but not to dCGRP-IH. iCGRP-IH but not dCGRP could be sensitive predictor for anti-CGRP therapy in migraine.

## P254 Real-world evidence on fremanezumab for migraine treatment in Japan: a retrospective analysis

### T. Takizawa^1^, S. Ohtani^1,2^, N. Watanabe^1^, K. Ihara^1^, N. Takahashi^1^, N. Miyazaki^3^, K. Ishizuchi^1^, R. Takemura^3^, S. Hori^2^, J. Nakahara^1^

#### ^1^Keio University School of Medicine, Department of Neurology, Tokyo, Japan; ^2^Keio University Faculty of Pharmacy, Division of Drug Informatics, Tokyo, Japan; ^3^Keio University Hospital, Biostatistics Unit, Clinical and Translational Research Center, Tokyo, Japan

##### **Correspondence:** T. Takizawa

*The Journal of Headache and Pain 2024,* **25(Suppl 1)**: P254

**Objective:** We presented real-world data on galcanezumab in Japan at EHC 2022. Fremanezumab, along with erenumab, is one of the second anti-calcitonin gene-related peptide (CGRP) monoclonal antibodies that became available in Japan. Our aim in the present study was to evaluate the efficacy and safety of fremanezumab in patients in a real-world setting in Japan. To the best of our knowledge, no real-world studies focusing solely on fremanezumab have been published in any international journal from Asia.

**Methods:** We retrospectively examined patients with migraine who received four doses of fremanezumab as their first CGRP monoclonal antibodies (mAbs) between December 2021 and August 2022 at Keio University Hospital. We assessed changes in monthly migraine days, responder rate, and migraine-associated symptoms. We also investigated injection site reactions and adverse events.

**Results:** Twenty-nine patients (79.3% female, aged 47.2 ± 12.4 years) were analyzed. Compared with those at baseline, monthly migraine days had decreased by 5.9 days (95% confidential interval, 3.1-8.7; *p*<0.001) at 4 months. The 50% responder rate was 55.2% at 4 months. A total of 57.9%, 47.8%, and 65.0% of patients showed improvement in the severity of photophobia, phonophobia, and nausea/vomiting, respectively. Injection site reaction was the most common adverse event (55.2%).

**Conclusion:** This study revealed that fremanezumab is effective and safe for the prevention of migraine in Japanese patients. Fremanezumab also improved migraine-associated symptoms in approximately half of the patients.

## P255 Galcanezumab for prevention of episodic and chronic migraine in real life in Colombia and Mexico: a multicenter prospective cohort study

### J. Munoz-Cerón^1,2^, K. Velez^3^, I. Rodríguez^3^, N. Hernández^4^, C. Guerra^4,5^, S. Bohorquez^6^, J. D. Jiménez^7^, C. Moreno^8,2^, M. Ramos^9^, P. Cavanzo^9^, Y. Rojas^10^, R. Bernal^10^, R. López^11^, L. Gallo^1^

#### ^1^Hospital Universitario Mayor Méderi – CIMED, Neurology, Bogotá, Colombia; ^2^Clínica Universitaria Colombia – Keralty, Bogotá, Colombia; ^3^Asociación Mexicana de Cefaleas y MIgraña AC, Ciudad de México, Mexico; ^4^SURA, Medellín, Colombia; ^5^Clínica SOMA, Medellín, Colombia; ^6^Hospital Kénnedy, Bogotá, Colombia; ^7^ACN, Pereira, Colombia; ^8^ACN, Bogotá, Colombia; ^9^ACN, Neurology, Bogotá, Colombia; ^10^ACN, Manizales, Colombia; ^11^ACN, Medellín, Colombia

##### **Correspondence:** J. Munoz-Cerón

*The Journal of Headache and Pain 2024,* **25(Suppl 1)**: P255

**Objective:** To describe the efficacy and safety of galcanezumab in real life setting in Colombia and Mexico

**Methods:** Real life, independent, prospective, multicentric, cohort study. Patients aged ≥18 years with diagnosis of migraine according to ICHD 3 criteria who were selected to be treated with galcanezumab were included. The analysis included reduction in monthly headache days (MHD) and monthly migraine days (MMD) as co-primary outcomes, secondary outcomes encompassed change in HIT6, GAD7, PHQ9 scores, analgesic intake days, global self-perception and incidence of side effects focused on constipation. Observations at base line, months 3 and 6 were carried out.

**Results:** 98 patients were included, men age 43,4 (SD12.8), rank 18-75 years, female 83,9%. At base line patients reported and average 22.6 and 6.3 MHD for chronic and episodic migraine patients respectively. The study was completed by 72% of the patients. At 6th month we observed a significant reduction in MHD − 12.3 days and − 2.5 days in chronic and episodic groups respectively *p* < 0.05 for both cases. HIT 6, GAD 7 and PHQ 9 scores improved significantly comparing baseline to month 6, (64.1 to 52.3), (6.84 to 3,67) and (8.99 to 4,56) respectively. Analgesic intake changed from 14.4 to 7.6 (*p*<0.01). The data regarding constipation showed that 8% of the patients presented it as emergent side-effect, of those whith history of this symptom 33.3% did not worse after starting treatment, 10.3% got worse, 6.9% improved; 41.4% of the subjects never described this complain. ≥50% response was achieved by 57% and 52% of the subjects with episodic and chronic migraine respectively. The exploratory analysis identified HIT-6 higher than 60 and analgesic intake more than 10 days/month as predictive factors of therapeutical failure.

**Conclusion:** Galcanezumab is efective and safe in patients sufering episodic and chronic migraine at the real sife setting in patients from Colombia and Mexico.

**Fig. 1 (Abstract P255):**
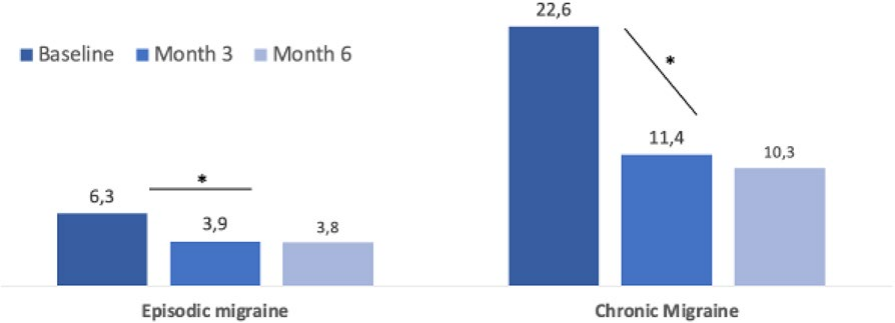
MHD variation from base line to month 3 and 6. *p<0.05

**Fig. 2 (Abstract P255):**
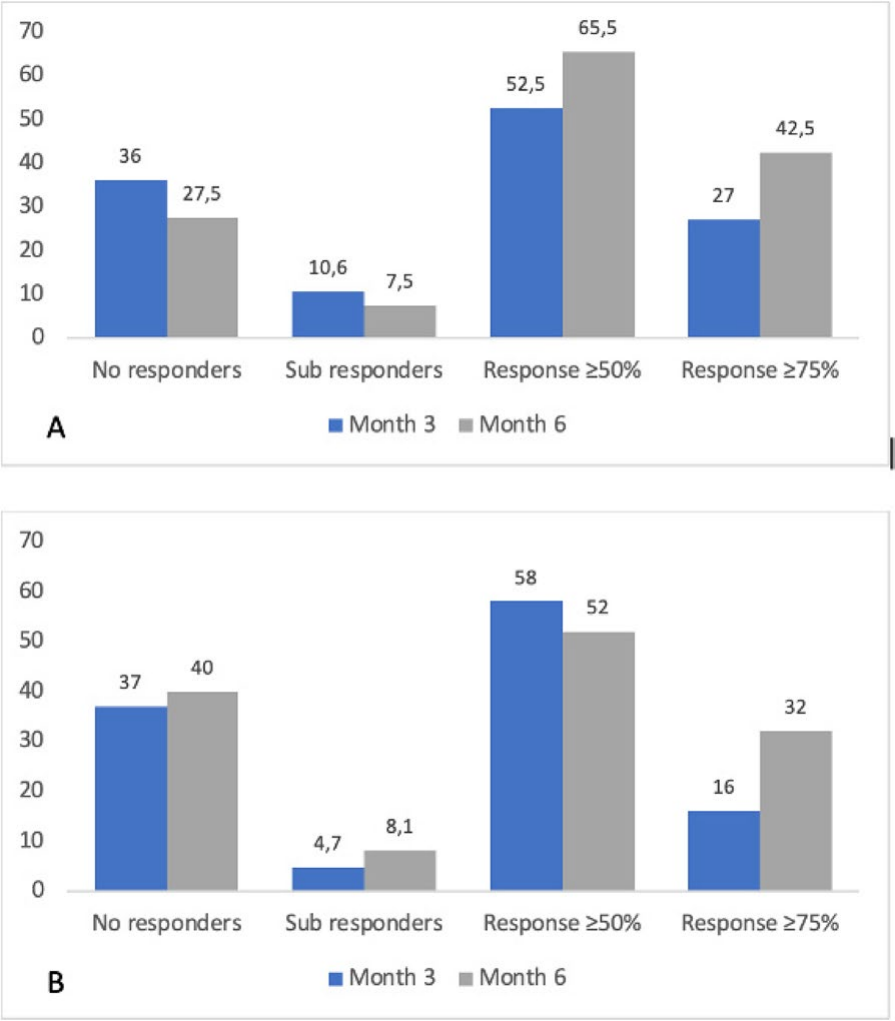
Mean percent of patients with no response, Sub-response ≥50%, and ≥75%, **A**. Chronic migraine, **B**. Episodic migraine

**Fig. 3 (Abstract P255):**
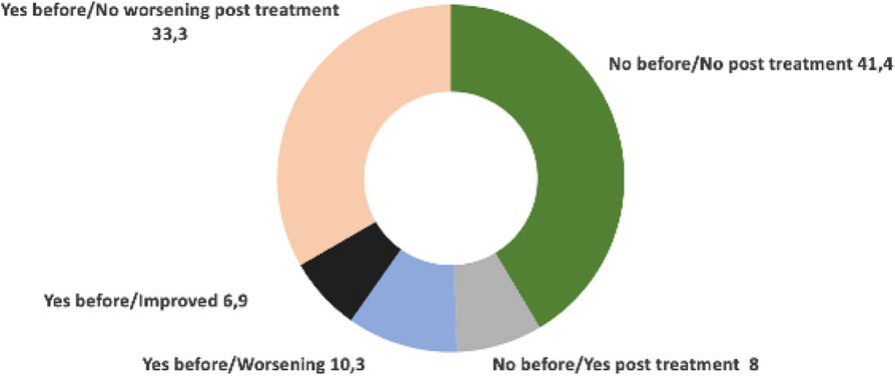
Constipation specific data at month 3 comparing pre to post treatment-percentages

## P256 Maximizing migraine relief: unveiling the therapeutic potential of switching between anti-CGRP monoclonal antibodies

### A. Jaimes Sanchez, A. Gómez García, O. Pajares, P. Ibanez de la Cadiniere, J. S. Rodriguez-Vico

#### Fundación Jiménez Díaz, Neurology, Madrid, Spain

##### **Correspondence:** A. Jaimes Sanchez

*The Journal of Headache and Pain 2024,* **25(Suppl 1)**: P256

**Objective:** To evaluate the efficacy of switching between anti-CGRP monoclonal antibodies (mAb) in the treatment of patients with migraine.

**Methods:** A review of clinical records from a tertiary hospital headache unit was conducted. Migraine patients who switched between mAb and received ≥3 doses of the second treatment were included. The primary objective was to assess efficacy (≥50% reduction in monthly headache/migraine days) at 3rd and 6th months. Secondary variables were median headache/migraine frequency reduction, and a multivariate study for prognostic factors.

**Results:** A total of 937 records were evaluated, 174 patients switched, 130 met inclusion criteria. Most participants had chronic migraine (90%). Table 1 shows demographic/clinical features. The first mAb used was erenumab (76.9%), galcanezumab (5.4%), or fremanezumab (17.7%). Patients had received a median of 10 doses. Reasons for switching included ineffectiveness (53.8%), administrative reasons (23.1%), adverse effects (15.4%), loss of efficacy (12.3%) and ineffectiveness upon reinitiation (1.5%). For the second treatment, erenumab was used in 3.1% of cases, galcanezumab in 64.6%, and fremanezumab in 32.3%. In 80% of cases, a change in target (ligand/receptor) was made. The median time to switch was 4 months. The switch led to a >50% response in 26% of patients at 3rd month and 31.8% at 6th month (Fig 1). Furthermore, the monthly headache days decreased from 23.3(+/-8.2) to 19.7(+/-9.7) days at the 3rd month, and to 17.7(+/-9.9) at the 6th month. Regarding migraines, the median decreased from 14.7(+/-8.7) to 9(+/-9.1) days in the first quarter and remained at 9(+/-9.3) in the second quarter (*p*<0.001). Multivariate analysis identified lower baseline headache frequency, shorter chronicity, and switching to another target as predictors of favorable response.

**Conclusion:** Switching anti-CGRP mAb reduces headache/migraine frequency, especially in patients with lower burden, shorter chronicity, and change of target (ligand/receptor).

**Table 1 (Abstract P256):**
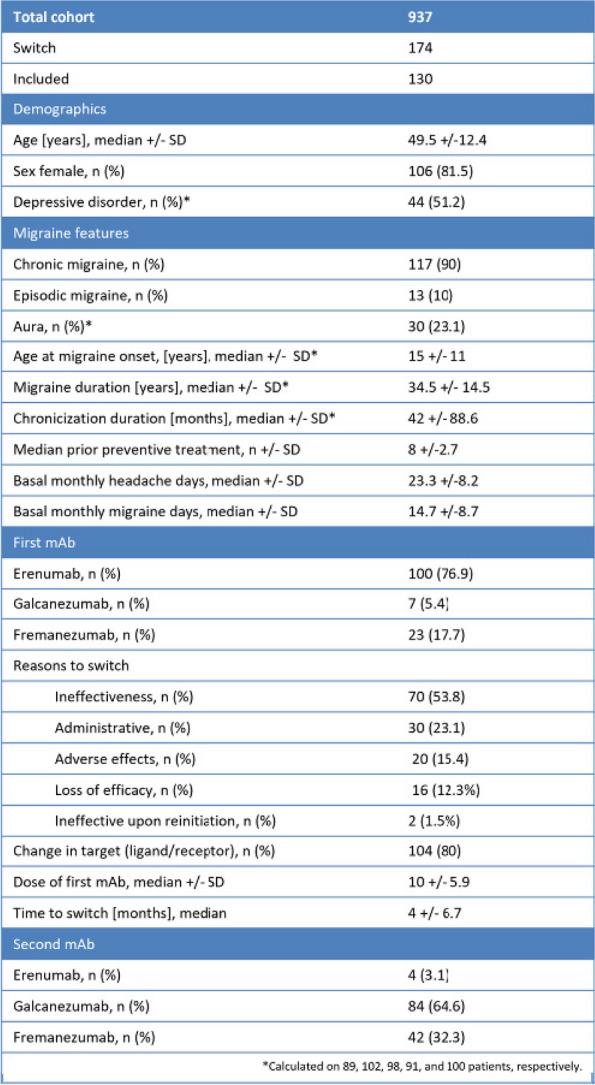
See text for description

**Fig. 1 (Abstract P256):**
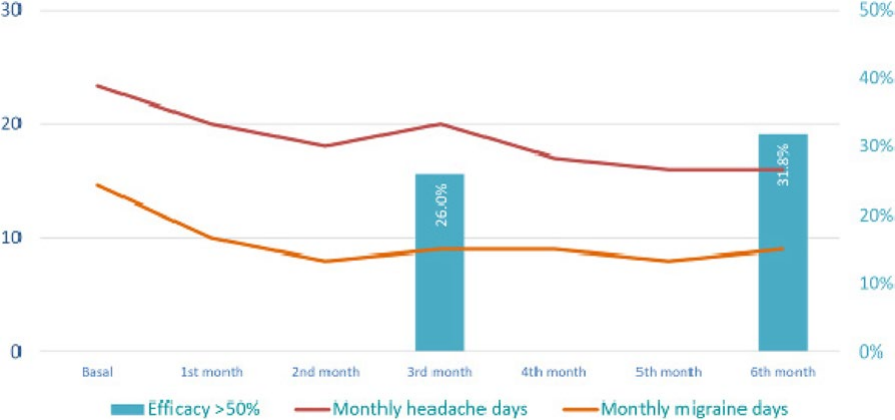
Percentage of patients with a >50% response in headaches or migraines at the third and sixth month and decrease in the number of the monthly headache and migraine days

## P257 Effectiveness of switching CGRP monoclonal antibodies in non-responder patients in the UAE: a retrospective study

### R. Suliman, V. Santos, I. Al Qaissi, B. Aldaher, A. Al Fardan, H. Al Barrawi, Y. Bader, J. L. Supena^1^, K. Alejandro, T. Alsaadi

#### American Center for Psychiatry and Neurology, Neurology, Abu Dhabi, United Arab Emirates

##### **Correspondence:** T. Alsaadi

*The Journal of Headache and Pain 2024,* **25(Suppl 1)**: P257

**Objective:** CGRP mAbs have shown promising effectiveness in migraine management compared to other preventative treatment options. Currently there are several studies related to the efficacy and tolerability of CGRP mAbs as a preventative treatment for migraine. However, many questions remain unanswered when it comes to switching between antibody classes as a treatment option in migraine patients. The present study seeks to explore and assess the treatment response to CGRP mAb in patients who have previously failed other CGRP mAbs. This study represents the first real-world analysis and will provide valuable insights into the use of CGRP mAbs in clinical practice.

**Methods:** This was a retrospective, real-world, exploratory study. Patients who were treated with two GCRP mAbs were retrospectively analyzed. Data was collected from one site, 53 patients with migraine switched between 3 CGRP mAb classes due to lack of efficacy of the original prescribed CGRP mAb. Efficacy of switching between classes of CGRP mAb"s was evaluated through documented MMD"s in patient diaries and clinical records. Non-parametric analysis was used to compare efficacy of the first 6 months of each prescribed medication.

**Results:** The analysis of efficacy demonstrated that some improvements were seen in both class switch cohorts (CGRP/R to CGRP/L and CGRP/L to CGRP/R). However, the most noticeable improvement in efficacy of the prescription switch was found in patients who switched between different medications of the CGRP/L class. Both CM and EM patients showed improved MMD"s, however CM patients demonstrated a higher responsiveness of efficacy in lateral switching, The safety of switching between CGRP classes was well noted as many AEs presented pre- class switch did not lead to the discontinuation of treatment.

**Conclusion:** The findings of this study suggest that switching between different classes of CGRP mAbs is a potentially safe and clinically viable practice that may have applications for those experiencing AEs on their current CGRP mAb. This is especially true for patients initially on ligand targeted CGRP mAb who experience AEs or lack of meaningful efficacy, as the ligand-ligand cohort demonstrates the best outcome.

## P258 Non-responders to the first anti-CGRP mAb: Is there benefit in switching antibody class or adding botulinum toxin?

### S. Malheiro, J. Fernandes, I. Laranjinha, C. Andrade

#### Centro Hospitalar Universitário de Santo António, Neurology, Porto, Portugal

##### **Correspondence:** S. Malheiro

*The Journal of Headache and Pain 2024,* **25(Suppl 1)**: P258

**Objective:** Monoclonal antibodies against calcitonin gene-related peptide or its receptor (anti-CGRP mAbs) are new mechanism-based prophylactic drugs in refractory migraine. However, there are still few data regarding possible therapeutic strategies to be adopted in case of non-responders to a first antibody. The aim of our study was to evaluate the benefit of switching antibody class or adding botulinum toxin in our cohort of non-responders to a first antibody.

**Methods:** A prospective database of patients with refractory migraine treated with anti-CGRP mAbs at our hospital center was analyzed at four moments: at baseline; after treatment with the first antibody; after treatment with a second antibody; and, in a selected group of patients, after add-on therapy with botulinum toxin.

**Results:** There were 18 patients, all female, with a median age of 45,50 (41,00-49,75), with a median number of headache days per month of 21,50 (13,75-30,00) and of migraine days of 13,50 (10,75-17,00) before treatment with the first antibody. At the time of withdrawal of the first antibody, the median number of headache days was 17,00 (14,00-30,00) (*p*=0,551), and migraine days 13,50 (10,75- 18,00) (*p*=0,938). Assuming non-response to the first antibody, a switch was made to another class of antibody, with a change of median number of headache days to 18,00 (11,25-28,00) (*p*=0,450) and migraine days to 15,00 (10,00-20,25) (*p*=0,167). In 7 patients, concomitant treatment with botulinum toxin was associated; after at least 3 months of association, and comparing with after failure of the first antibody, the median of headache days changed to 19,00 (5,00-26,00) (*p*=0,128), and migraine days to 11,00 (5,00-13,00) (*p*=0,167).

**Conclusion:** In our cohort, switching between anti-CGRP mAbs or associating botulinum toxin did not reach a statistical significant benefit in the number of pain days. Given the small sample size and short time of follow-up of some of the patients there is a possibility of a more significant and late response.

## P259 Psychological intervention in refractory chronic migraine patients

### M. Zambrano Camiña, C. Nieves Castellanos, M. A. Olivier, L. Ferré González, S. Díaz Insa

#### Hospital Universitari i Politècnic La Fe, Headache Unit. Neurology, Valencia, Spain

##### **Correspondence:** S. Díaz Insa

*The Journal of Headache and Pain 2024,* **25(Suppl 1)**: P259

**Objective:** Refractory Chronic Migraine (RCM) patients are the migraine population with the poorest quality of life and highest impact in their daily living, affecting work, family and social aspects. We wanted to know if a psycological groupal intervention could benefit those severely affected patients.

**Methods:** We describe basal and after intervention outcome in RCM patients. All of them had failed to all available migraine preventive treatments (including all 3 antiCGRP mAbs available). We present sociodemographic condition, MIDAS (discapacity), HIT-6 (impact), MsQol (quality of life), PCS (catastrophising), SWLS (life satisfaction), PANAS (afectivity), EEP-10 (psycological stress), BRCS (resiliency), HADS (anxiety and depression), C-SSRS (suicidal risk) and CRES-4 (treatment satisfaction). Psycological intervention consisted in 10 groupal sessions during 2 months.

**Results:** 23 of 27 patients completed all prior and after intervention measures. Patients were randomized half of them in intervention cohort and the rest as control group. We found an improvement in almost all measures (slight in most of them) after intervention compared to basal situation and compared to control group. In the intervention group, resiliency improved from 10.64 to 13.18; MIDAS decreased from 80.45 to 67.09 and the most significant improvement was in MsQol,from 14.94 to 28.56 points. The improvement was subjectively greater referred by patients. Most of them wanted to continue with this kind of intervention and expressed that sharing their condition with similarly affected pairs helped to manage this usually alone-fighting illness.

**Conclusion:** Psycological intervention can help RCM patients feel better and improve skills to cope with this terrific condition. Sharing with other patients was useful for most of the patients.

## P260 Predictors of response to anti-CGRP monoclonal antibodies: a scoping review and meta-analysis of real-world experience

### J. B. Hong^1^, K. S. Lange^1^, L. H. Overeem^1,2^, P. Triller^1^, B. Raffaelli^1,3^, U. Reuter^1,4^

#### ^1^Charité – Universitätsmedizin Berlin, Department of Neurology, Berlin, Germany; ^2^Humboldt Graduate School, International Graduate Program in Medical Neurosciences, Berlin, Germany; ^3^Berlin Institute of Health at Charité (BIH), Clinician Scientist Program, Berlin, Germany; ^4^Universitätsmedizin Greifswald, Greifswald, Germany

##### **Correspondence:** J. B. Hong

*The Journal of Headache and Pain 2024,* **25(Suppl 1)**: P260

**Objective:** Monoclonal antibodies (mAbs) against calcitonin gene-related peptide (CGRP) and its receptor (CGRP-R) are increasingly being used as preventive treatments for migraine. Their efficacy and safety were established through numerous randomized placebo-controlled trials and real-world studies, yet a significant proportion of patients do not respond to this treatment. We aimed to review and analyze the current literature on real-world predictors of response to CGRP-targeted therapies for migraine prevention.

**Methods:** We searched Embase and MEDLINE databases for real-world studies reporting on predictors of response to CGRP and/or CGRP-R mAbs, defined as a 30% or 50% reduction in monthly headache or migraine days at varying durations of follow-up. Quantitative synthesis was performed when more than one effect size was available for a predictor.

**Results:** We identified 38 real-world studies that investigated the association between various predictors and response rates. A good response to triptans and unilateral pain were predictors of a good response to CGRP(-R) mAbs (pooled OR for being a responder: 2.66, 95% CI: 1.73-4.09 and 2.69, 95% CI: 1.01-7.20 respectively). Conversely, obesity, the presence of daily headaches, a higher number of non-successful previous prophylactic medications, and comorbid depression were predictive of a poor response to CGRP(-R) mAbs (pooled OR for being a responder: 0.38, 95% CI: 0.15-1.00; 0.24, 95% CI: 0.14-0.43; 0.83, 95% CI: 0.73-0.93 and 0.51, 95% CI: 0.35-0.73 respectively).

**Conclusion:** Certain characteristics of migraine headaches, migraine history, and the presence of comorbid conditions such as depression or obesity can help predict the response to treatment with CGRP(-R) mAbs. Future studies should confirm these results and solidify our understanding of mechanisms behind treatment failure. Knowledge about robust predictors of response can help generate more tailored treatment strategies in patients with migraine.

## P261 Non responders to anti-CGRP/R monoclonal antibodies: unmet needs and challenges in the management of drug-resistant migraine

### L. F. Iannone, A. Burgalassi, G. Tabasso, F. De Cesaris, G. Vigani, G. Mannaioni, A. Chiarugi, P. Geppetti

#### University of Florence, Health Sciences, Florence, Italy

##### **Correspondence:** L. F. Iannone

*The Journal of Headache and Pain 2024,* **25(Suppl 1)**: P261

**Objective:** To describe the outcome of patients who withdraw anti-calcitonin gene-related peptide monoclonal antibodies (anti-CGRP mAbs) and the causes for withdrawal and to characterize patients who do not respond to treatment.

**Methods:** We conducted a prospective analysis on outpatients who started erenumab, galcanezumab, or fremanezumab until February 2023, and assessed the follow-up of patients who withdrew from treatment and restarted or not treatment with a new anti-CGRP mAb. Reasons for anti-CGRP mAb withdrawal and the follow up thereafter were recorded. The overall population (*i.e.,* withdrawn for any reason) and a subgroup that discontinued solely for ineffectiveness were considered.

**Results:** A total of 472 patients were treated with anti-CGRP mAbs, and 136 (28.8%) discontinued the treatment. Among them, 46.3% received erenumab, 14.7% fremanezumab, and 39% galcanezumab. Almost all patients have chronic migraine (91.9%) and 81.6% medication overuse. Most patients withdrew treatment due to ineffectiveness (*n*=96, 70.6%), followed by lost to follow up during treatment (18, 13.1%) or adverse events (13, 9.6%). Three (2.2%) patients withdrew treatment for unspecified personal choice or pregnancy. One patient discontinued for no compliance (0.7%), and 3 (2.2%) for physician decision. Overall, 106 (77.9%) patients discontinued treatment during the first 12-month follow-up. At the first follow-up after withdrawn, 66 (48.5%) patients started a new pharmacological treatment (i.e., switching anti-CGRP mAbs, OnabotulinumtoxinA, anticonvulsants, others), 54 (39.7%) were lost to follow-up and 16 (11.8%) decided to not start other treatments.

**Conclusion:** Managing patients who do not respond to anti-CGRP treatment remains a challenge, that requires tailored management strategies to optimize response a a timely identification of non-responders.

## P262 Tablet preventive therapy for CGRP monoclonal antibody non-responders

### N. Vashchenko^1^, A. Uzhakhov^2^, D. Korobkova^1^, J. Azimova^1^, K. Skorobogatykh^1^

#### ^1^University Headache Clinic, Moscow, Russian Federation; ^2^Darmed University Clinic, Astana, Kazakhstan

##### **Correspondence:** N. Vashchenko

*The Journal of Headache and Pain 2024,* **25(Suppl 1)**: P262

**Objective:** CGRP monoclonal antibody therapy has emerged as a highly effective treatment for migraine and is considered first-line therapy in some countries. However, a subset of patients do not respond favourably to this therapy. The aim of our study was to investigate the potential benefit of subsequent preventive treatment with tablets in these non-responders.

**Methods:** We conducted a retrospective analysis of patients in a headache clinic who had an inadequate response to CGRP monoclonal antibody therapy (specifically, erenumab 70 mg or fremanezumab 225 mg monthly or both). Patients who switched to prophylactic tablet therapy, such as candesartan, venlafaxine, amitriptyline, topiramate or beta-blockers, were included in the study. Patients who opted for botulinum toxin prophylaxis were not included.

**Results:** A total of 128 patients who did not respond to CGRP monoclonal antibody prophylaxis were included in the study. Of these, 72 patients (27 with episodic migraine, 45 with chronic migraine) tried tablet prophylaxis, which they had not previously used. Remarkably, 37 (51%) of these patients showed a positive response to tablet therapy after three months. The specific tablets used were amitriptyline - 11 (30%) patients, topiramate - 9 (24%) patients, venlafaxine - 7 (19%) patients, metoprolol - 6 (16%) patients and candesartan - 4 (11%) patients.

**Conclusion:** While CGRP monoclonal antibodies are considered highly effective drugs for migraine prophylaxis, it is noteworthy that a subset of patients may benefit from more cost-effective tablet therapies, even in cases of poor response to CGRP monoclonal antibodies.

## P263 Risk and reward seeking in cluster headache

### W. Naber, R. Brandt, M. Ferrari, R. Fronczek

#### Leiden University Medical Center, Neurology, Leiden, Netherlands

##### **Correspondence:** W. Naber

*The Journal of Headache and Pain 2024,* **25(Suppl 1)**: P263

**Objective:** Anecdotally, patients with cluster headache (CH) have an increased tendency towards risk-reward seeking. We assessed risk-reward seeking behavior in people with CH, people with migraine, and headache-free controls (HC) using the Zuckerman Sensation Seeking Scale (ZSSS) and the Balloon Analogue Risk Task (BART).

**Methods:** In this single-center, cross-sectional, explorative study, all patients with episodic or chronic CH (eCH;cCH), or migraine from our Neurology outpatient clinic were screened for eligibility between 2019-2020. HC were recruited through advertisements. All groups were matched for age and sex. Participants completed the ZSSS and two BART rounds. To stimulate risk-taking behavior, the second BART round "suddenly" included an actual reward. Results of the BART are expressed in terms of the number of balloon "pumps", as a measure of risk taking behavior. Between-groups outcomes were analyzed using a multivariate regression analysis, within-groups outcomes with a paired T-Test.

**Results:** 140 participants (35 eCH,35 cCH, 35 migraine & 35 HC) were included. Participants with eCH scored higher compared to all groups on the total ZSSS (eCH adjusted mean: 21.8 SD±9.5; cCH:+8.2 (95%CI: 3.9;12.4); Migraine +6.9 (95% CI: 2.9; –11.0);HC:+6.6 (95% CI:2.8; 10.4)), the "experience seeking" subscale and the "disinhibition" subscale. During the second BART, participants with cCH had a lower number of corrected mean pumps (Δ -10.6,95% CI(-20.9; -0.3)) and popped balloons (Δ-2.3, 95%CI(-4.5; -0.1)) compared to eCH, and lower number of mean pumps (Δ-5.9, 95%CI (-11.7;-0.05)) compared to HC, indicating a decrease in risk-reward behavior.

**Conclusion:** While the ZSSS points towards an increase in risk-reward seeking behavior in eCH compared to people with migraine and HC, the BART paradoxically shows a decrease in this behavior in cCH. We hypothesize that there is an inherent increase in risk-reward seeking behavior in CH, which is dampened due to the impact of the chronic form of the disease

**Table 1 (Abstract P263):**
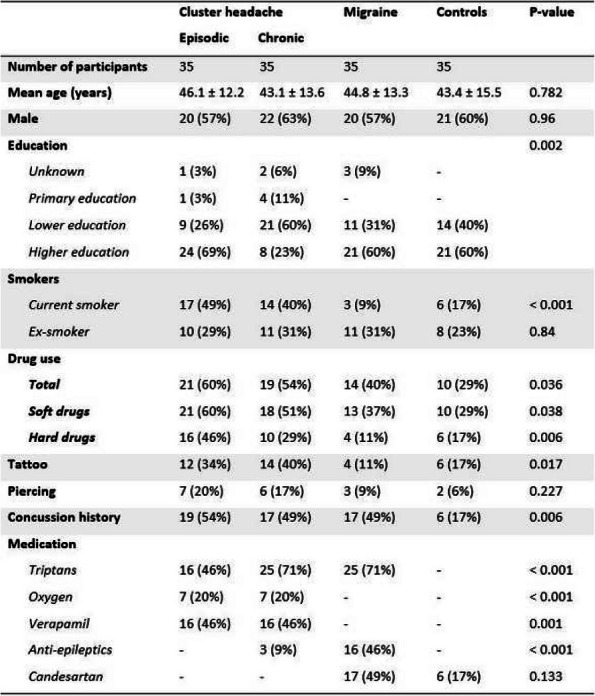
Baseline characteristics. All data is depicted as mean ±SD or N (%)

**Fig. 1 (Abstract P263):**
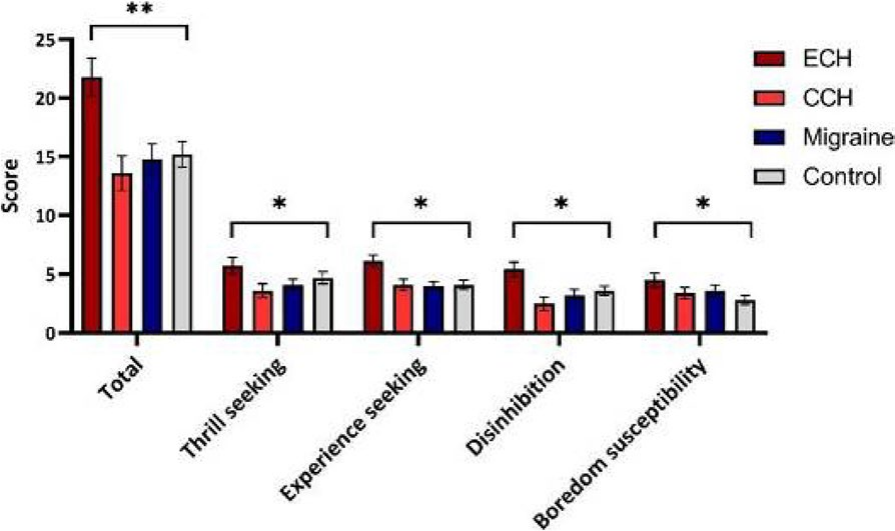
Zuckerman sensation seeking scale. Adjusted total scores and sub scores (**=*p*<0.05; *=*p*<0.05)

**Table 2 (Abstract P263):**
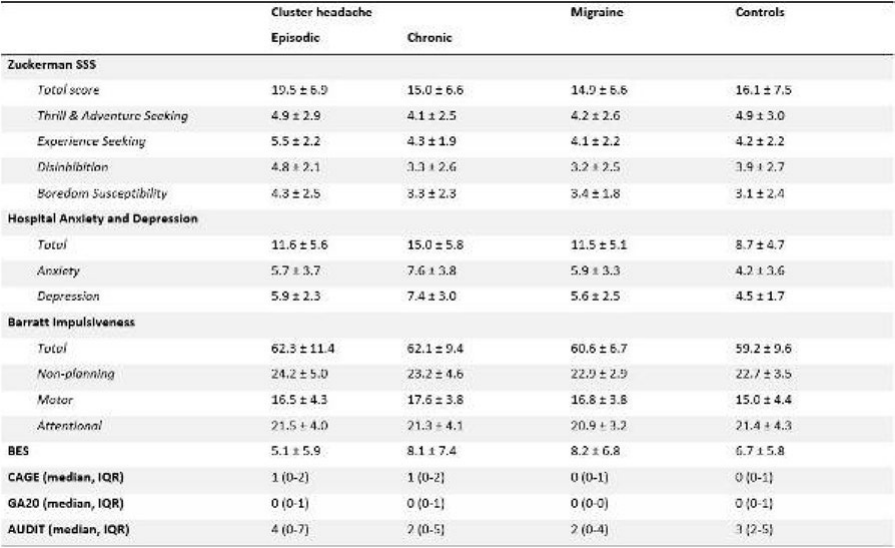
Psychometric questionnaired (raw means ± SD or meian (IQR) when applicable)

## P264 Validation of the english Cluster Headache Impact Questionnaire (CHIQ)

### K. Kamm, A. Straube, R. Ruscheweyh, M. Burish

#### LMU Hospital, LMU Munich, Department of Neurology, Munich, Germany

##### **Correspondence:** K. Kamm

*The Journal of Headache and Pain 2024,* **25(Suppl 1)**: P264

**Objective:** Cluster headache (CH) is a severe, highly disabling primary headache disorder. To assess disability in CH patients the CH-specific, short (8 items) Cluster Headache Impact Questionnaire (CHIQ) was first developed and validated in German. Here, we present the validation of the English CHIQ version.

**Methods:** The English translation of the CHIQ was already provided with the initial publication. Here, the CHIQ and additional questionnaires were answered online by CH patients visiting UT Health Houston headache outpatient center and via an U.S. patient group. Reliability and validity were evaluated.

**Results:** Analysis was based on 271 CH patients. Reliability and validity were determined in active episodic (*n* = 65) and chronic (*n* = 69) CH patients (67.2% male, 52.8 ± 13.1 years). The CHIQ showed good internal consistency (Cronbach"s α = 0.79) and factor analysis identified a single factor. Test-retest reliability was adequate (ICC 0.94, *n* = 40). Convergent validity was shown by significant correlations with the Headache Impact Test™ (HIT-6™; *r* = 0.75, p < 0.01), subscales of the Hospital Anxiety and Depression Scale (HADS; depression: *r* = 0.63, *p* < 0.01; anxiety: *r* = 0.64, *p* < 0.01), Perceived Stress Scale (PSS-10; *r *= 0.65, *p* < 0.01) and with CH attack frequency (*r* = 0.31; *p* < 0.01).

Highest CHIQ total scores were found in cCH patients (25.2 ± 8.2, *n* = 69), followed by active eCH patients (21.4 ± 8.9, *n* = 65). As shown before, also eCH patients in remission reported disability by CH (14.1 ± 12.8, *n* = 126). Group differences were significant (H [2] = 39,011, *p* < 0.001).

**Conclusion:** The CHIQ is a short, CH-specific questionnaire for the assessment of the impact of CH. The questionnaire is reliable, valid, and easy to administer which makes it a useful tool for clinical use and research.

**Fig. 1 (Abstract P264):**
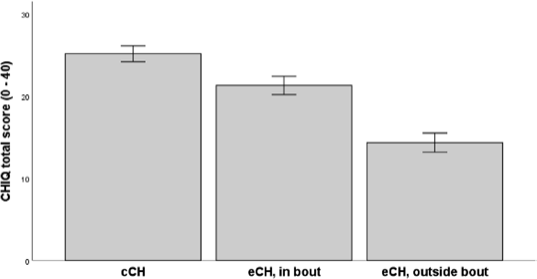
See text for description

**Fig. 2 (Abstract P264):**
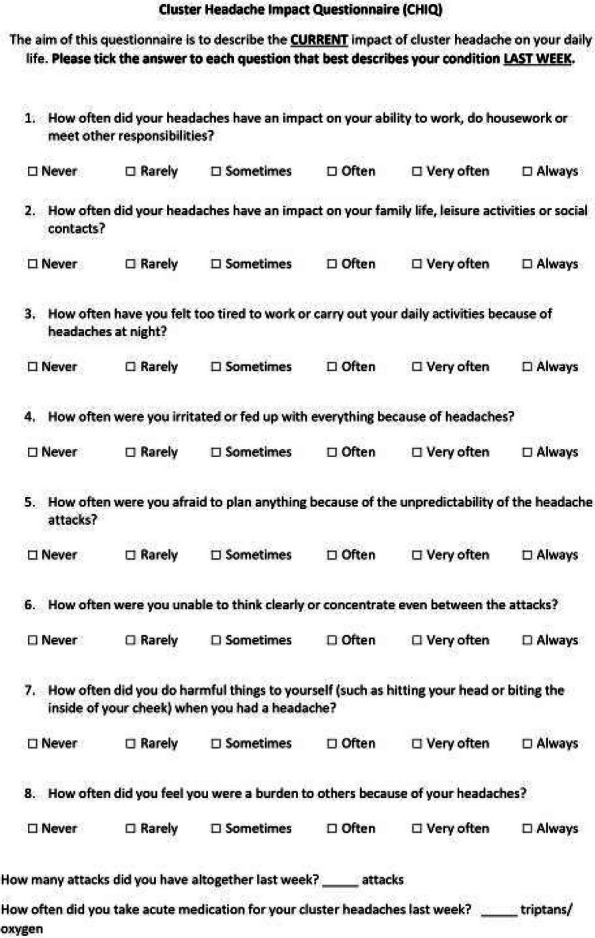
See text for description

## P265 The enigmatic triangle of cluster headache attacks, sleep and the biological clock: rationale and protocol of the CIESTA study

### P. J. van Tilborg^1^, E. A. Tolner^2^, O. C. Meijer^3^, M. R. Boon^3^, T. J. Upton^4^, S. L. Lightman^4^, W. Karlen^5^, G. M. Terwindt^1^, G. J. Lammers^1^, R. Fronczek^1^

#### ^1^Leiden University Medical Center, Neurology, Leiden, Netherlands; ^2^Leiden University Medical Center, Neurology and Human Genetics, Leiden, Netherlands; ^3^Leiden University Medical Center, Endocrinology, Leiden, Netherlands; ^4^University of Bristol, Henry Wellcome Laboratories for Integrative Neuroscience and Endocrinology, Translational Health Sciences, Faculty of Health Sciences, Bristol, United Kingdom; ^5^Ulm University, Institute of Biomedical Engineering, Ulm, Germany

##### **Correspondence:** P. J. van Tilborg

*The Journal of Headache and Pain 2024,* **25(Suppl 1)**: P265

**Objective:** The etiology of cluster headache (CH) is unclear. While research links attacks, sleep and the circadian rhythm, results are inconsistent and mostly based upon single-night measurements in hospital setting with limited data. We aim to determine whether CH attacks occur in relation to specific characteristics of sleep and the circadian rhythm.

**Methods:** Patients with chronic (*n*=16) or episodic (*n*=8) CH according to the ICHD-3 criteria with ≥6 nocturnal CH attacks per week will be included. A headache and sleep diary will be used to determine patients' sleep schedule and the occurrence of nocturnal CH attacks during a 1-week baseline period and a second week of neurophysiological measurements. To approximate regular home-sleep, all measurements will be conducted with wearables in a remote setting. Polysomnography will be performed with 'SleepLoop', a head worn biopotential (i.e. EEG, EOG, EMG) monitoring device. Circadian rhythm will be recorded with 'U-RHTYHM'. It enables microdialysis samples to be collected automatically at 20-minute intervals over 72 hours, resulting in high-frequency subcutaneous cortisol and melatonin levels. Additionally, actigraphy, skin and core body temperature (CBT), and heart rate variability (HRV) will be measured with skin sensors. Timing of attacks will be recorded in the diary and with the sleep headband. For episodic CH, all measurements are repeated during remission.

**Results:** Primary outcome is time from sleep stage transition to attack onset (sleep), and time from melatonin zenith and cortisol nadir respectively to attack onset (circadian rhythm). Secondary outcomes will include sleep stage and mean cortisol- and melatonin levels at attack onset, differences in CBT pattern and the sympathicovagal balance (HRV) at attack onset. Additionally, differences in sleep macrostructure, as well as melatonin and cortisol rhythms, during versus outside a cluster episode will be assessed.

**Conclusion:** This study will create a unique ambulatory neurophysiological dataset: CH attacks in relation to sleep and clock time-series data. Unravelling the role of the hypothalamus in CH will be of great value for evolving etiological hypotheses and can create targets for new treatments.

## P266 Migraine-cluster contiuum – Does it exist?

### J. Afra, E. Balogh

#### National Institute of Mental Health, Neurology and Neurosurgery, out-patient, Budapest, Hungary

##### **Correspondence:** J. Afra

*The Journal of Headache and Pain 2024,* **25(Suppl 1)**: P266

**Objective:** To present case histories of patients with headache characteristics of migraine and cluster headache

**Methods:** Data analysis of 5 patients

**Results:**

**Conclusion:** Our female patients present stages of a possible migraine-cluster continuum.

*Disclosure statement*: Informed consent to publish this case study and its potentially identifiable information of the patient was obtained from the individual or the individual's guardian. The patient or their guardian gave explicit permission for the publication of this case report, including any relevant clinical details.

**Table 1 (Abstract P266):** See text for description

	**K.A.**	**K.N.**	**S.A.**	**B.Cs.T.**	**G.J.**
**age at onset**	15	26	18	28	12
**frequency**	1-2 mths /y, after pregnancy 3-6 mths /year, attacks every 2-3 days, later daily	2-3 mths/y, always in summer, daily attacks in the first period, every other day later	3-4 mths/y always in spring, daily attacks	2-3 weeks/y, 1-2 attacks/day	1,5-2 mths/2-3 y, attacks every 1-2 days
**attack duration**	1-2h before and 7-8h after pregnancy	1-2h in the first period 12-24h later	3-5h	3h	2h
**localisation**	always R temporal	always R temporal	always behind the R eye	always R temporal,	always behind the L eye
**type of pain**	constant	pulsating	constant	constant	sharp
**intensity**	severe	severe	severe	severe	severe
**accompanying symptoms**	nausea/vomiting photo/osmophobia lacrimation, conjunctival injection, running nose on the R no aggravation by physical activity but bed rest	nausea/vomiting, photo/phonophobia lacrimation, nasal congestion on the R aggravation by physical activity, bed rest	nausea photo/phonohobia running nose on both sides bed rest	nausea photo/phonophobia ptosi, nasal congestion on the R aggravation by physical activity, bed rest, sleep helps	nausea/vomiting, strong osmo- mild photo/phonophobia diarrhea conjunctival injection, lacrimation, running nose on the L bed rest
**provocing factors**	none	weather change	none	none	none – but no attacks during and in between pregnancies
**familariry**	none	migraineous sister	none	none	none

## P267 Greater occipital nerve injection plus verapamil versus verapamil alone as first-line prophylactic treatment in episodic cluster headache: the multicentre, randomised, double-blind, placebo-controlled CHIANTI trial

### R. Brandt^1^, E. Couturier^2^, H. Carpay^3^, O. Gerlach^4^, E. van Zwet^5^, M. Niesters^6^, J. Haan^7^, W. Mulleners^8^, M. Ferrari^1^, R. Fronczek^1^

#### ^1^Leiden University Medical Center, Neurology, Leiden, Netherlands; ^2^Boerhaave Clinics, Neurology, Amsterdam, Netherlands; ^3^Migraine Clinic, Amsterdam, Netherlands; ^4^Zuyderland Hospital, Neurology, Geleen, Netherlands; ^5^Leiden University Medical Center, Biomedical Data Sciences, Leiden, Netherlands; ^6^Leiden University Medical Center, Anaesthesiology, Leiden, Netherlands; ^7^Alrijne Hospital, Neurology, Leiderdorp, Netherlands; ^8^Canisiu-Wilhelmina Hospital, Neurology, Nijmegen, Netherlands

##### **Correspondence:** R. Brandt

*The Journal of Headache and Pain 2024,* **25(Suppl 1)**: P267

**Objective:** To investigate whether a GON-injection with 80mg methylprednisolone at the start of a cluster headache episode just before the start of standard therapy with verapamil, provides a faster reduction in attack frequency at a lower required dosage of verapamil with fewer side effects compared to standard therapy with verapamil alone.

**Methods:** Participants were recruited from six academic and non-academic headache clinics in the Netherlands within 4 weeks after the start of their cluster episode, before the start of any preventive medication. A GON-injection (verum or placebo) was administered directly after randomisation, after which normal treatment with verapamil was started with a standardised dose escalation scheme (Figure 1). Attack frequency and intensity, side effects and use of medication were monitored with a daily electronic attack journal. Follow-up duration was 12 weeks.

**Results:** In total, 70 participants were included (36 verum, 34 placebo). The mean daily dose of verapamil in weeks 1-4 in the verum group (227 mg ± 126 mg) was lower than in the placebo group (287 mg ± 107 mg; Difference = 60mg, 95% CI: -4 to -116) (Figure 2, Figure 3). A lower median number of weekly attacks was observed during week 1 in the verum group compared to the placebo group (7 [2 – 11.75] vs 10 [6 – 17.5]; 95% CI = -1.0 to -8.0). The mean attack intensity in the verum group was lower than in the placebo group in week 1 (5.7 ± 1.9 vs 6.6 ± 1.8; CI 0.0 to 1.8) and over the entire 12-week study period (5.0 ± 1.8 vs 5.9 ± 1.9; 95% CI 0.01 to 1.8). Injection site pain did not differ between groups (*p*=0.19). Days with side effects were reported less frequently in the verum group (455/2520, 18%) than in the placebo group (605/2850, 21%; *p*<0.01). No treatment-related serious adverse events occurred.

**Conclusion:** A GON-injection with 80mg methylprednisolone at the start of a cluster headache episode in combination with standard therapy with verapamil is well tolerated and safe, provides a rapid improvement in attack frequency and intensity, and reduces the required dose of verapamil and the risk of side effects.

**Fig. 1 (Abstract P267):**
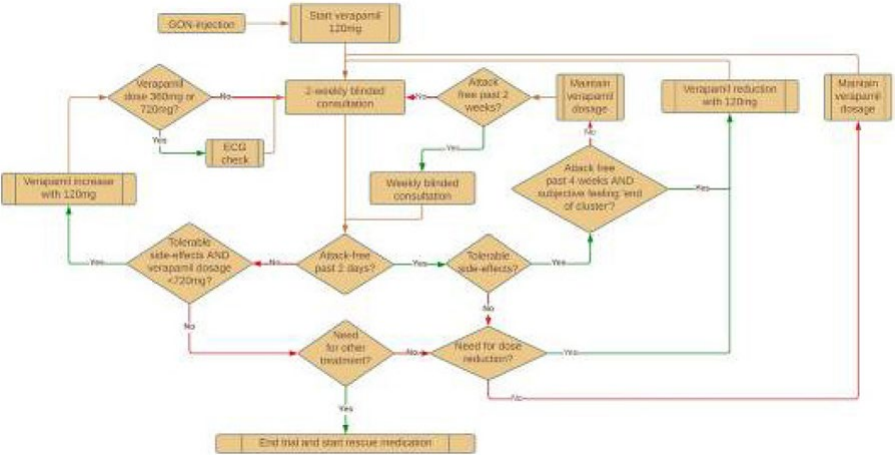
See text for description

**Fig. 2 (Abstract P267):**
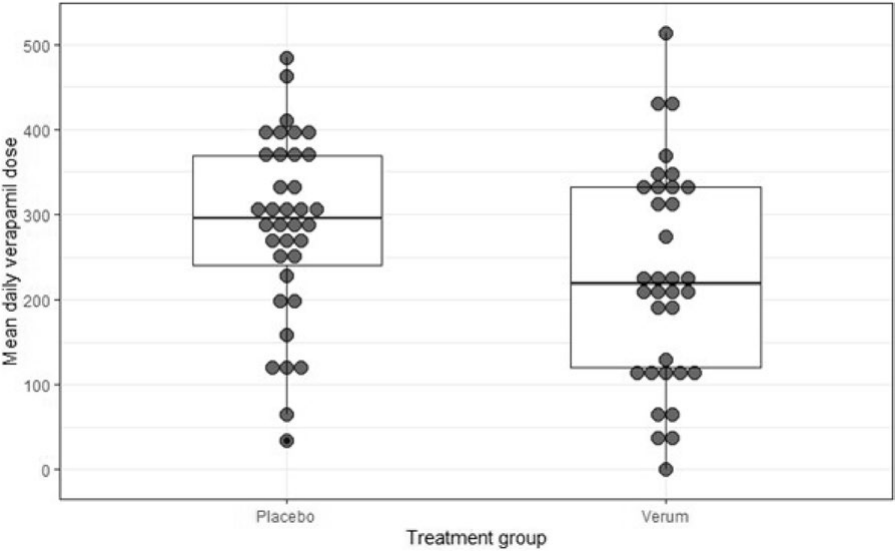
See text for description

**Fig. 3 (Abstract P267):**
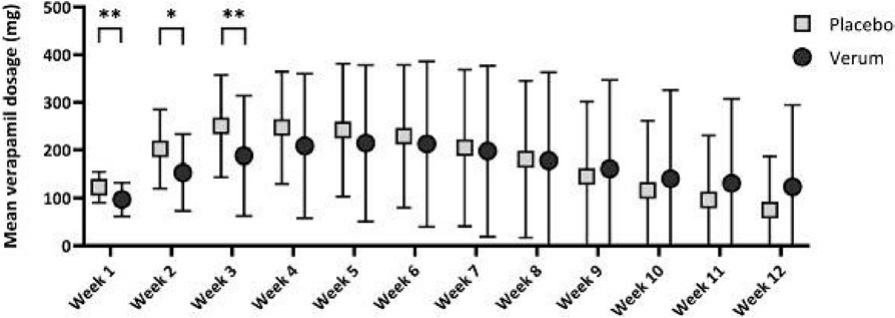
See text for description

## P268 Androgen profile in cluster headache. A prospective case-controlled study

### A. S. Petersen^1^, D. M. Kristensen^2,3,4^, T. F. Hansen^1,5^, N. Lund^1^, M. Barloese^6,7^, M. L. K. Søborg^1^, A. Snoer^1,8^, T. H. Johannsen^2,9^, H. Frederiksen^2,9^, A. Juul^2,7,9^, R. H. Jensen^1,7^

#### ^1^Danish Headache Center, Copenhagen University Hospital – Rigshospitalet Glostrup, Department of Neurology, Glostrup, Denmark; ^2^Copenhagen University Hospital – Rigshospitalet, Department of Growth and Reproduction, Copenhagen, Denmark; ^3^University of Rennes, Inserm, EHESP, Irset (Institut de Recherche en Santé, Environnement et Travail)-UMR_S 1085, Rennes, France; Roskilde University, Department of Science and Environment, Roskilde, Denmark; ^5^Copenhagen University, Novo Nordisk Foundation Center for Protein Research, Copenhagen, Denmark; ^6^University of Copenhagen, Department of Clinical Physiology and Nuclear Medicine,, Hvidovre, Denmark; ^7^University of Copenhagen, Department of Clinical medicine, Copenhagen, Denmark; ^8^H. Lundbeck A/S, Copenhagen, Denmark; ^9^Rigshospitalet Glostrup, University of Copenhagen, International Center for Research and Research Training in Endocrine Disruption of Male Reproduction and Child Health (EDMaRC), Copenhagen, Denmark

##### **Correspondence:** A. S. Petersen

*The Journal of Headache and Pain 2024,* **25(Suppl 1)**: P268

**Objective: **Is the testosterone concentration lower in patients with cluster headache compared to healthy controls and does remission restore testosterone concentrations to normal?

**Methods:** We did a prospective, observational case-controlled study in adults with cluster headache as defined by the ICHD-3. Participants with episodic cluster headache were sampled twice (during a bout and in remission), and participants with chronic cluster headache and healthy controls were sampled once. Sera were analyzed for total testosterone, luteinizing hormone (LH), and sex hormone-binding globulin. The concentration of free testosterone (fT) was calculated according to Vermeulen. Furthermore, we used genetic analysis to assess whether there are shared genetic risk variants for cluster headache and testosterone levels.

**Results:** In total, 60 males with episodic cluster headache, 60 males with chronic cluster headache and 60 healthy age-matched males were included. There was a reduction in the fT/LH ratio in patients with cluster headache independent of disease state as compared to controls (*P*<0.04). In the paired analyses of episodic cluster headache, we found no difference between bout and remission. In a linear regression model adjusted for age, number of hours slept, and use of acute medication, the mean fT/LH ratio was reduced by 35% (95%CI: 21-47%, *P*=2.0^-05^) in patients with chronic cluster headache and 24% (95%CI: 9-37%, *P*=0.004) in patients with episodic cluster headache in remission compared to controls. Additionally, a common single nucleotide polymorphism, rs112572874, was associated with both cluster headache (*P*=3.3^-5^) and fT concentration (*P*=6.0^-9^).

**Conclusion:** Our results demonstrate that the male endocrine system is altered in patients with cluster headache to a state of compensated hypogonadism. The reduction is not restored in remission. Further, our data demonstrates that compensated hypogonadism is not an epiphenomenon of reduced sleep, or the use of acute medication.

## P269 Association between calcitonin gene-related peptide and disease states in cluster headache. A prospective and controlled study

### A. S. Petersen^1^, N. Lund^1^, K. Messlinger^2^, S. L. Christensen^1^, M. Barloese^3,4^, N. R. Jørgensen^4,5^, L. J. Kogelman^1^, R. H. Jensen^1,4^

#### ^1^Danish Headache Center, Copenhagen University Hospital – Rigshospitalet Glostrup, Department of Neurology, Glostrup, Denmark; ^2^Friedrich-Alexander-Universität Erlangen-Nürnberg, Institute of Physiology and Pathophysiology, Erlangen, Germany; ^3^University of Copenhagen, Department of Clinical Physiology and Nuclear Medicine, Hvidovre, Denmark; ^4^University of Copenhagen, Department of Clinical medicine, Copenhagen, Denmark; ^5^Rigshospitalet Glostrup, University of Copenhagen, Department of Clinical Biochemistry, Copenhagen, Denmark

##### **Correspondence:** A. S. Petersen

*The Journal of Headache and Pain 2024,* **25(Suppl 1)**: P269

**Objective:** Does plasma levels of calcitonin gene-related peptide differ among episodic cluster headache in bout, episodic cluster headache in remission, chronic cluster headache, and age- and sex matched controls?

**Methods:** This study used data from The Danish Cluster Headache Biobank, a prospective observational case-control study. Plasma was collected from 201 participants diagnosed with cluster headache according to the International Classification of Headache disorders (3rd edition) and from 100 age- and sex matched controls. Participants with episodic cluster headache were sampled twice (bout and remission). CGRP concentrations were measured with a fully validated radioimmunoassay for human CGRP*.*

**Results:** For participants with episodic cluster headache, plasma levels of CGRP were higher in bout than in remission (mean difference: 17.8 pmol/L, 95%CI: 6.6-28.0, *p*=0.002). CGRP levels in bout were not different from participants with chronic cluster headache (*p*=0.24). Overall, levels of CGRP were significantly lower in participants with cluster headache compared to controls (*p*<0.05). Plasma CGRP did not associate with attack frequency or use of acute medication within the 24 hours preceding the sampling (*P*>0.60).

**Conclusion:** We have established that plasma CGRP is lower in the remission phase but does not differ among participants with ongoing cluster headache attacks, i.e., chronic cluster headache and episodic cluster headache in bout. These results support the hypothesis that the CGRP system is disrupted in persons with cluster headache even in remission as compared to controls.

**Fig. 1 (Abstract P269):**
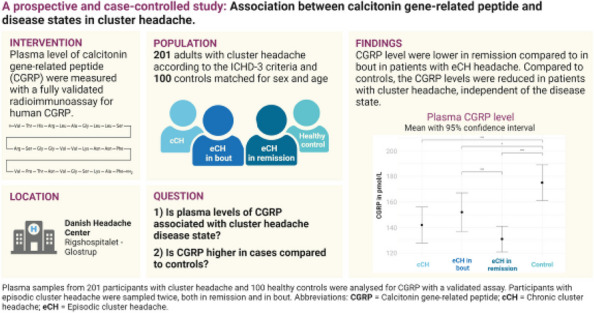
See text for description

## P270 Role of the resting state networks in episodic cluster headache: cerebral connectivity analysis with Hd-Eeg

### M. Corrado^1,2^, R. De Icco^1,2^, F. Bighiani^1,2^, G. Vaghi^1,2^, M. Semprini^3^, G. Sances^1,2^, A. Putortì^1,2^, V. Grillo^1,2^, F. Cammarota^1,2^, M. Allena^1,2^, C. Tassorelli^1,2^

#### ^1^Mondino Foundation, Pavia, Italy; ^2^University of Pavia, Department of Brain and Behavioral Sciences, Pavia, Italy; ^3^Italian Institute of Technology, Genova, Italy

##### **Correspondence:** M. Corrado

*The Journal of Headache and Pain 2024,* **25(Suppl 1)**: P270

**Objective:** The pathophysiological mechanisms underlying episodic cluster headache (eCH), and shift between active and remission phases, are still not fully understood. We aimed to define specific internodal connectivity patterns of the default mode network (DMN) in eCH patients, through advanced brain connectivity analyses with high-density EEG (HD-EEG).

**Methods:** Twenty-four patients with eCH and 19 healthy controls (HCs) were enrolled. Patients with eCH were evaluated during both the active (T0) and the remission (T1) phases of disease. Of these 24 patients, 8 were registered only at T0, 10 only at T1, while 6 completed both registrations. The DMN areas considered for the analysis were: the right and left angular gyrus (RANG and LANG), the medial pre-frontal cortex (MPC) and the posterior cingulate cortex (PCC).

**Results:** The study of internodal brain connectivity in patients showed lower connectivity at T1 (remission) when compared to T0 between PCC and MPC (T0=0.078±0.009 vs. T1=0.049±0.006, *p*=0.022) and between PCC and RANG (T0=0.076 ± 0.008 vs. T1=0.052±0.005, *p*=0.024). Furthermore, connectivity at T1 was lower when compared to HCs, specifically between PCC and MPC areas (CHe-T1=0.049±0.005 vs. HS=0.067±0.005, *p*=0.028).

**Conclusion:** eCH patients evaluated during a remission phase of disease showed lower brain connectivity between specific areas of the DMN when compared with either eCH patients tested during an active phase and HCs. This finding may represent a biological marker of disease, while the fluctuation in PCC connectivity may reflect pathophysiological mechanisms involved in the shift from one phase of disease to the other.

## P271 Beyond coincidence: clinical course and biology of coinciding cluster headache and SUNHA

### J. Jansen^1^, J. Scheurink^2^, T. Balvers^3^, J. Haan^4,5^, R. Fronczek^4^, W. Mulleners^1^

#### ^1^Canisiu-Wilhelmina Hospital, Neurology, Nijmegen, Netherlands; ^2^Radboud University Medical Center, Nijmegen, Netherlands; ^3^Stichting Epilepsie Instellingen Nederland (SEIN), Heemstede, Netherlands; ^4^Leiden University Medical Center, Neurology, Leiden, Netherlands; ^5^Alrijne Hospital, Leiderdorp, Netherlands

##### **Correspondence:** J. Jansen

*The Journal of Headache and Pain 2024,* **25(Suppl 1)**: P271

**Objective:** Limited research has been conducted on the combination of cluster headache and short-lasting unilateral neuralgiform headache attacks (SUNHA). This case-series describes our established cases.

**Methods:** A retrospective case-series of 10 patients with cluster headache and SUNHA.

**Results:** Cluster headache and SUNHA can occur concurrently or consecutively, with overlapping localization of pain and autonomic symptoms observed in 9 of our 10 cases. Magnetic resonance imaging showed an ipsilateral trigeminal neurovascular conflict in 62% of cases. Among them, 80% underwent microvascular decompression with varying efficacy on both cluster headache and SUNHA.

**Conclusion:** Co-occurring cluster headache and SUNHA suggests a pathophysiological relationship between the two, which may be explained by a bidirectional interaction between posterior hypothalamic function and trigeminal root damage. The role of a trigeminal neurovascular conflict seems likely in this context. Thus, in medically intractable cases, dedicated magnetic resonance imaging is suggested. Microvascular decompression should be considered if a neurovascular conflict is found.

## P272 Awareness of cluster headache among students of hail university

### W. Alesefir

#### University of Hail, Hail, Saudi Arabia

##### **Correspondence:** W. Alesefir

*The Journal of Headache and Pain 2024,* **25(Suppl 1)**: P272

**Objective:** Cluster headache (CH) needs to be better studied in our region, given the absence of previous literature in Saudi Arabia regarding awareness of this rare disease. Furthermore, CH is one of the leading causes of life disability. Therefore, we looked to increase CH awareness among students at Hail University

**Methods:** We used a cross-sectional study prospectively studying the awareness of CH among Hail University students by an electrical form questionnaire; our study aimed to improve the understanding of CH among Hail University students and secondly to measure the prevalence of CH in our university.

**Results:** The study survey was completed by 400 students in total. 32.8 percent of the candidate has heard about CH. Only 86 (21.5%) of the subjects experienced CH. Compared to 17.7% of male students, 25.7% of female students reported having an attack at least. A precise 158 (39.5%) students attended medical colleges, while 242 (60.5%) attended non-medical institutions

**Conclusion:** We found a low level of awareness among Hail University students. We ultimately concluded that to improve Hail University students" knowledge of the disease more effectively, we needed to raise their degree of awareness. Although it"s one of the

region"s first studies, more studies must follow to confirm our results

**Fig. 1 (Abstract P272):**
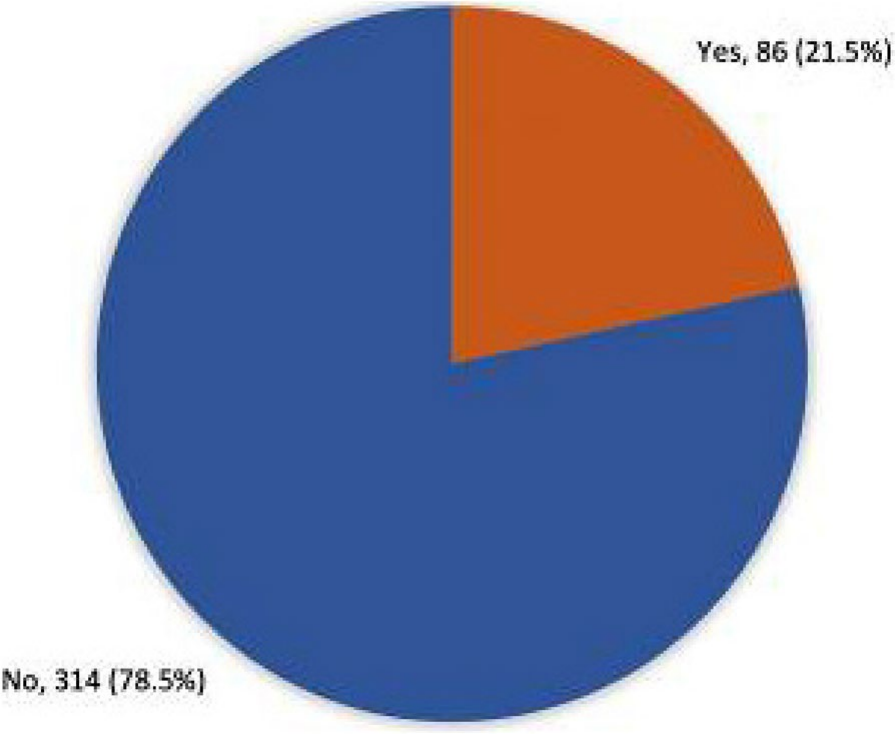
Prevalence of cluster headache attacks among university students, Hail University, Saudi Arabia

**Fig. 2 (Abstract P272):**
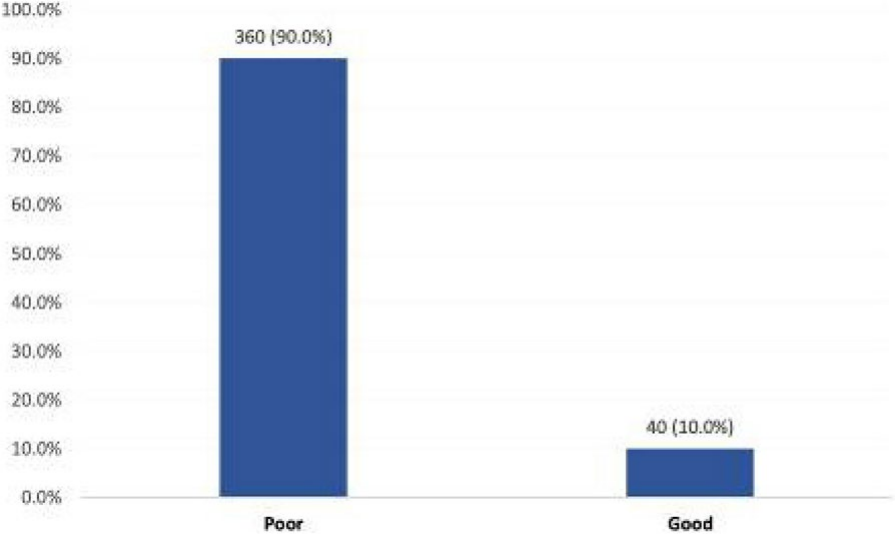
Overall awareness level regarding cluster headache among university students, Hail University. Exact 40 (10%) had a general awareness level regarding cluster headaches, while 360 (90%) had a poor rank

## P273 Switching preventive migraine treatments during the COVID-19 pandemic: a 4-year observational analysis

### L. Idrovo^1^, L. Fernandes^1^, K. Suwanlaong^2^, P. J. Goadsby^2^

#### ^1^Leeds Teaching Hospitals NHS, Neurology- Headache Service, Leeds, United Kingdom; ^2^NIHR-King's Clinical Research Facility, London, United Kingdom

##### **Correspondence:** L. Idrovo

*The Journal of Headache and Pain 2024,* **25(Suppl 1)**: P273

**Objective:** During COVID-19 access to BTX-A became restricted. CM patients were switched to anti-calcitonin gene-related peptide (CGRP) monoclonal antibody (MAB) therapies although they had been responding to BTX-A. Our objective was to analyse the outcome of switching to anti-CGRP therapies and the effect of COVID-19 pandemic on our cohort

**Methods:** We audited outcomes from consecutive chronic migraine patients referred from Jan2019 until Jan2023 to two headache services in the UK. We analysed demographics, tolerability, response rate of BTX-A, number of patients switched from BTX-A to anti-CGRP MABs before, during and post pandemic and reasons behind the decision to switch. We also analysed tolerability and response rates of patients on anti-CGRP therapy. We analysed the clinical characteristics of patients who switched back from anti-CGRP therapies to BTX-A

**Results:** 496 new patients with CM, 421 (84%) were female, mean age was 45 (± 13, SD) years. 341 (69%) patients received BTX-A. In 2019, 137 had ≥ 2 BTX-A cycles by PREEMPT. Sixty-three (46%) had a ≥30% reduction of monthly headache days (MHD) and 51 (37%) stopped BTX-A: lack of efficacy 47 (92%), side effect 2 (4%), episodic migraine 2 (4%). There were 39 (29%) switched to anti-CGRP therapies from which 35 (89%) had at least a 30% reduction of HD and 4 (10%) stopped; side effects in 1 and no efficacy in 3. During pandemic,132 had BTX-A, 57 (43)% had a ≥30% reduction of MHD and 81 stopped BTX-A: 72 (88%) due to lack of efficacy/personal preference, 2 (2%) side effects, and 7 (5%) due to clinic delays/pandemic. Sixty-six (50%) were switched to anti-CGRP therapies of which 45 (68%) had a ≥ 30% reduction of HD and 21 (32%) stopped anti-CGRP: 16 lack of efficacy and 5 side effects. In 2022, 72 had ≥ 2 BTX-A cycles. Fifty-three (73%) had a ≥30% reduction of MHD and 15 (21%) stopped BTX-A due to lack of efficacy. Fifteen were switched to anti-CGRP therapies of which 14 (93%) had a ≥30% reduction of MHD and 1 (7%) stopped

**Conclusion:** About a third of our cohort referred to BTX-A clinic switched to Anti-CGRP therapy. Lack of efficacy was the main reason for switching as both treatments were well tolerated. A small proportion switched back to BTX-A

## P274 Anti-CGRP in the treatment of Hereditary transthyretin amyloidosis – related migraine

### J. Fernandes^1^, S. Ferreira^2^, C. Alves^1^, A. P. Sousa^3^, I. Laranjinha^1^, C. Andrade^1^

#### ^1^Centro Hospitalar Universitário de Santo António, Neurology Department, Porto, Portugal; ^2^Centro Hospitalar Universitário de Santo António, Liver Transplant Unit, Porto, Portugal; ^3^Centro Hospitalar Universitário de Santo António, Neurophysiology Department, Porto, Portugal

##### **Correspondence:** J. Fernandes

*The Journal of Headache and Pain 2024,* **25(Suppl 1)**: P274

**Objective:** Anti-CGRP antibodies are considered a safe and well tolerated treatment for migraine prophylaxis, however caution is needed in patients with vascular comorbidities. In hereditary transthyretin amyloidosis (ATTRv amyloidosis) it has been described an amyloid deposition in leptomeningeal vessels resulting in cerebral amyloid angiopathy, albeit the prevalence of vascular lesions is still unknown.

**Methods:** We present 2 cases of refractory migraine in patients with ATTRv amyloidosis where fremanezumab was implemented. Both cases report to women with ATTRv amyloidosis with Val30met diagnosed more than 20 years ago that underwent liver transplantation, and chronic migraine without aura since early adulthood.

**Results:** The first case, a 46-year-old, presented with 20 migraine days/month, and scored 76 on the HIT-6 scale, despite trial with 7 different prophylactic regimens previously, including onabotulinumtoxin A. After 12 months of treatment with Fremanezumab 225mg/month there was a reduction to 13 migraine days/month, scoring 64 on the HIT-6 scale and no reported side effects. The second case, a 50-year-old, had 16 migraine days/month, scoring 72 on the HIT-6 scale in spite of 3 different prophylactic drugs and onabotulinumtoxinA in the past. After 3 months of treatment with fremanezumab 225mg/month, there was a reduction to 7 migraine days/month, scoring 52 on the HIT-6 scale, again with no reported side effects.

**Conclusion:** Treatment with fremanezumab in patients with ATTRv amyloidosis was effective and safe in our experience. To the best of our knowledge this is the first report of anti-CGRP in ATTRv amyloidosis related chronic migraine.

*Disclosure statement*: Informed consent to publish this case study and its potentially identifiable information of the patients was obtained from the individuals involved. The patients gave explicit permission for the publication of this case report, including any relevant clinical details.

## P275 Rimegepant as off-Label treatment for cluster headache attacks: case series

### V. Grozeva

#### Private Headache Practice, Sofia, Bulgaria

*The Journal of Headache and Pain 2024,* **25(Suppl 1)**: P275

**Objective:** Rimegepant is a calcitonin gene-related peptide receptor (CGRP) antagonist that has shown efficacy and safety in both acute and preventive treatment of migraine. CGRP can trigger migraine, and blockade of the canonical CGRP receptor is effective in the treatment of migraine. Based on good translational studies showing CGRP is elevated in acute cluster headache and CGRP can trigger attacks in patients in bout, rimegepant was tested off-label for acute treatment of episodic cluster headache.

**Methods:** To examine the effect of rimegepant 75 mg as an acute treatment of cluster headache attacks through a case series of four patients.

**Results:** Four male patients with episodic cluster headache reported varying decrease in their headache attack intensity, and a change in attack duration after using rimegepant. These patients, who were not taking any preventive drugs, like verapamil, agreed to try rimegepant 75 mg as an off-label acute treatment for 4 consecutive attacks during their cluster headache bout. One of them had a complete relief of his current attack, and he remained attack-free for 1.5 days after intake of rimegepant. The rest three reported pain intensity decrease, but not a complete relief after taking 75 mg of rimegepant. The attacks were milder and "bearable", but prolonged (longer than 3 hours). There were no adverse events noted, and the attacks returned to their natural characteristics after treatment discontinuation.

**Conclusion:** Tried as an acute treatment, rimegepant 75 mg showed encouraging results in terms of cluster headache pain intensity, and attack suppression. There was good tolerability and no safety concerns. Larger patient cohort and probably a higher rimegepant dose is needed to prove efficacy in episodic cluster headache.

## P276 Side shift in cluster headache: a prospective single center study

### M. Youn^1^, H. Jang^2^, M. J. Lee^1,3^

#### ^1^Seoul National University Hospital, Neurology, Seoul, South Korea; ^2^Samsung Medical Center, Sungkyunkwan University School of Medicine, Neurology, Seoul, South Korea; ^3^Seoul National University Hospital, Seoul, South Korea

##### **Correspondence:** M. Youn

*The Journal of Headache and Pain 2024,* **25(Suppl 1)**: P276

**Objective:** Cluster headache (CH) is characterized by strict unilaterality of attacks. Side shift of attacks has been reported in several cohorts and recently suggested as a potential predictor of conversion between episodic and chronic CH. However, the prevalence and detailed pattern of side shift are available only in limited literature. In addition, no study has investigated clinical characteristics and treatment responses regarding side shifts. We aimed to assess the prevalence and pattern of side shifts and clinical characteristics and treatment response associated with side shifts in CH patients.

**Methods:** We prospectively recruited and followed up CH patients in a single university hospital. Patients who experienced two or more lifetime CH bouts were interviewed regarding their history and patterns of side shifts using a structured questionnaire. The demographics and disease characteristics collected at baseline and treatment response followed up after 2 – 4 weeks were compared between patients with vs. without side shifts. Treatment response of acute and preventive medications was defined as ≥ 50% reduction in pain intensity and ≥ 50% reduction of headache frequency, respectively.

**Results:** Out of 124 patients, 26 (21.0%) experienced side shifts, of which 16 (12.9%) experienced shifts between bouts, 13 (10.5%) within a bout, and 4 (3.2%) within an attack. Among patients who experienced between-bout shifts, the pain persisted on one side once a shift has occurred in 6 (37.5%) and the side of pain shifted between bouts in 10 (62.5%) patients. The demographics and characteristics of CH did not differ according to the history of side shift. Treatment response from acute and preventive medications were comparable between patients with and without history of side shifts.

**Conclusion:** About 1 out of 5 patients experienced side shifts during disease courses, which occurred between bouts, during a bout, and even during an attack. No difference between patients with vs. without history of side shifts was noted in demographics, clinical characteristics, and treatment responses, suggesting that side-shifted CH is not a distinct entity or migraine variant but within a spectrum of CH.

## P277 Premonitory symptoms in cluster headache: a comparison between a clinical collection and the broader patient population

### H. Gosalia^1^, M. Khalil^2,3^, D. Y. Wei^4^, P. J. Goadsby^1,5^

#### ^1^NIHR-King's Clinical Research Facility, London, United Kingdom; ^2^Hamad Medical Corporation, Doha, Qatar; ^3^Spire Hull and East Riding Hospital, Hull, United Kingdom; ^4^King's College London, Department of Neurology, London, United Kingdom; ^5^University of California, Los Angeles, CA, United States

##### **Correspondence:** H. Gosalia

*The Journal of Headache and Pain 2024,* **25(Suppl 1)**: P277

**Objective:** To compare premonitory symptom findings from a cluster headache questionnaire-based study of patients from a broad National Health Service (NHS)-based sample and data collected at a tertiary headache centre at King's College Hospital.

**Methods:** An audit was conducted at King's College Hospital. We reviewed patients' headache symptomology, particularly the premonitory symptoms. In addition, a questionnaire-based study was conducted amongst NHS home-oxygen users across 14 Health Boards in Scotland, UK. The diagnosis of cluster headache was established using the criteria of the International Classification of Headache Disorders (ICHD-3). Questions around cluster headache symptoms, treatment and premonitory symptoms were administered.

**Results:** The audit identified 36 patients investigating nine premonitory-symptom questions and the questionnaire-based study consisted of 95 patients, at interim analysis, with fourteen premonitory-symptom questions. In the audit, 33% of the cohort had episodic cluster headache (ECH), while 67% had chronic cluster headache (CCH). In the questionnaire study 53% had ECH and 47% had CCH. For the questionnaire study, the presence of premonitory symptoms was reported by 80% of the cohort, the most prominent: cravings (52%), irritability (47%) and neck stiffness (46%). The presence of premonitory symptoms was reported by 92% of the patients in the audit. The most common premonitory symptoms in the audit being mood changes (67%), neck stiffness (56%) and concentration changes (56%). The average number of premonitory symptoms was 4 (IQR: 1-6) and 3 (IQR: 1-4) in the questionnaire study and audit, respectively. A history or diagnosis of migraine was reported by 37% of patients in the questionnaire study and 58% in the audit cohort.

**Conclusion:** Premonitory symptoms are present in cluster headache patients in both populations. The symptoms may prove to be useful clinical indicators of the disorder presence and indicate the early stage of attacks. A common prominent premonitory symptom amongst both cohorts was neck stiffness, which provides a useful insight into the clinical presentation of cluster headache.

## P278 Cluster headache. Are there differences by gender?

### M. A. Olivier, C. Nieves Castellanos, L. Ferré González, S. Díaz Insa

#### Hospital Universitari I Politécnic La Fe, Neurology, Valencia, Spain

##### **Correspondence:** S. Díaz Insa

*The Journal of Headache and Pain 2024,* **25(Suppl 1)**: P278

**Objective:** Cluster headache (CH) is a trigeminal-autonomic cephalalgia; it more common in men, beginning at 20-40 years. Infrequent; incidence: 53 /100,000 inhabitants/year. 10-15% is chronic.

Objective: Analyze a cohort of patients diagnosed with CH and identify clinical differences and response to treatment according to gender.

**Methods:** Retrospective, descriptive study of a cohort of 72 patients diagnosed with CH. We analyzed: age at onset and at diagnosis, clinical symptoms and evolution, weekly attacks, treatments received and response to them.

**Results:** 72 patients: 13 women ( 18.1%) and 59 men (81.9%).

Mean age (MA) onset: W 32.5. M 35.4.

MA at diagnosis: W 40.2. M 39.5.

Laterality: W: right (69.2%). M: right (47.5%) and 8.5% alternating.

W: 61.5% episodic; 38.5% chronic.

M: 81.4% episodic; 18.6% chronic.

Rhinorrhea: W: 84.6%; M: 66.1%.

Epiphora: W: 76.9%; M:83%.

Ptosis: W: 76.9%; M:50.8%.

Nasal packing: W: 84.6%; M: 64.4%.

Eye redness: W: 69.2%; M: 74.6%.

Facial sweating: W:23.1%; M:32.2%.

Irritability: W: 69.2%; M:83%.

Average number of weekly attacks: W:27.75; M:20.3.

Smoking: W: 61.5%; M:84.7%

Alcoholic habit: W: 7.7%; M: 50.85%

Effectiveness:

Verapamil: W:18.2%. M:50%.

Topiramate: W: 0%. M: 20.8%.

Blocks: W:20%. M: 57.4%.

Lithium: W: 0%. M: 12.5%.

Botox: W: 14.3%. M: 53.3%.

Monoclonal: W: 16.7%. M:42.8%.

Corticosteroids: W: 42.8%. M: 44.4%.

Oxygen: W:50%. M: 76.2%

**Conclusion:** In our cohort, we found that women had an earlier onset compared to men but were diagnosed later. They also exhibited a higher degree of right-side pain, increased chronicity, and less irritability during attacks (a characteristic symptom of CH). Additionally, women had fewer toxic habits. They experienced a higher number of attacks per week and a diminished response to treatments. It is crucial to consider CH in women, even if their symptoms are not as typical, because their quality of life is adversely affected due to disease progression and the limited effectiveness of standard treatments.

## P279 A demographic comparison between the North and South of the UK, in a cluster headache population: What can we learn?

### H. Gosalia^1^, N. Karsan^1^, P. J. Goadsby^1,2^

#### ^1^NIHR-King's Clinical Research Facility, London, United Kingdom; ^2^University of California, Los Angeles, CA, United States

##### **Correspondence:** H. Gosalia

*The Journal of Headache and Pain 2024,* **25(Suppl 1)**: P279

**Objective:** To report demographic and clinical findings from a cluster headache questionnaire-based study of a National Health Service (NHS)-based sample, from the North and South of the UK: Scotland and South-Central England, respectively.

**Methods:** A questionnaire-based study was conducted and distributed amongst NHS home-oxygen users across 14 Health Boards in Scotland, UK, covering 5,463,300 patients, with a sample size of 95 patients. At the time of analysis, 7 Clinical Commissioning Groups were involved in England, covering 4,581,933 with a sample size of 83. The diagnosis of cluster headache was established using the criteria of the International Classification of Headache Disorders (ICHD-3).

**Results:** From the England database of 489 patients, 176 patients consented to receiving questionnaires, of which, 83 responded. On the Scotland database of 514 patients, 198 patients consented to receiving questionnaires of which 95 returned a completed response. Episodic cluster headache (ECH) was 53% and chronic cluster headache (CCH) was 47% in Scotland. For England, ECH was 66% and CCH was 34%. The median time to diagnosis was 2 (IQR: 10-1) months for Scotland. For England 3 (IQR: 8-1) months. The top three most reported cranial autonomic symptoms were congestion (85%), aural discomfort (70%) and miosis (63%) for Scotland, and lacrimation (90%), ptosis (67%) and rhinorrhoea (69%) for England. The median number of cranial autonomic symptoms reported were 6 (IQR: 6-3) for Scotland and 5 (IQR: 6-4) for England. The most effective acute and preventive treatment was oxygen and verapamil in both cohorts. Of ECH patients, 53% reported rebound headache with mean time 33 (SEM: 11) minutes and of CCH patients, 54% with mean time 17 (SEM: 4) minutes after oxygen use in Scotland. For England, ECH rebound headache was reported by 64% with mean time 48 (SEM: 11) minutes and for CCH patients 64% with mean time 29 (SEM: 8) minutes.

**Conclusion:** Treatment responses for both acute and preventives are common to both regions, suggesting that clinical treatment and management is uniform across the country. Rebound headache with oxygen is relatively common in this cohort and needs further study.

## P280 Exercise as a non-pharmacological treatment option for patients with cluster headache

### M. K. Kang, Y. Hong, S. J. Cho

#### Dongtan Sacred Heart Hospital, Hallym University College of Medicine, Neurology, Hwaseong, South Korea

##### **Correspondence:** M. K. Kang

*The Journal of Headache and Pain 2024,* **25(Suppl 1)**: P280

**Objective:** Exercise has been proposed as treatment option for other chronic pain and primary headaches. This study aimed to evaluate effects of exercise in cluster headache.

**Methods:** This study included patients, who had been diagnosed with cluster headache according to the International Classification of Headache Disorders (ICHD) at a single center in Korea from September 2016 to April 2023. At the time of enrollment or follow-up period, participants were asked whether they exercised when cluster headache occur. Headache improvement after exercise was evaluated. This study also examined the types and intensity of exercise that were effective in reducing cluster headache symptoms. Different exercise modalities, such as aerobic exercise and strength training, were included in the exercise program. The intensity of exercise was classified based on the Borg Rating of Perceived Exertion (RPE).

**Results:** Among 167 patients, 54 patients did exercise during cluster period. After exercise 23 patients (42.6%) showed improvement of headache. Among them14 patients (25.9%) showed more than 50% improvement of headache, and 3 patients (13%) showed disappearance of pain. The effective exercise type for relief headache were running (39.1%), squat (30.4%), and stair climbing (21.7%), followed by walking and push-up at 17.4% each. Exercise intensity effective in reducing headache was severe intensity in 12 patients(52.2%) and moderate intensity in 10 patients (43.5%). 11 patients tried exercise only, of them 3 patients (27.3%) showed improvement of headache and 2 patients (18.2%) showed more than 50% redcution of headache severity. 34 patients tried exercise and triptans, of them 17 patients (50.0%) improved headaches and 10 patients (29.4%) showed more than 50% improvement in headaches with exercise (*p* < 0.001).

**Conclusion:** This study provides evidence that exercise can be an effective treatment option for cluster headache. And exercise, especially moderate to severe intensity can be considered as a promising treatment option for cluster headache patients.

## P282 40 years of experience in cluster headache: description of a Spanish series

### J. Madera Fernandez^1,2,3^, S. Pérez-Pereda^1,2,3^, V. González-Quintanilla^1,2,3^, G. Gárate^1,2,3^, A. González-Suárez^1,2,3^, N. Cavada Bustamante^1,2,3^, A. Fernández Rufino^1,2,3^, J. Pascual Gómez^1,2,3^

#### ^1^University Hospital Marqués de Valdecilla, Neurology, Santander, Spain; ^2^IDIVAL, Santander, Spain; ^3^Universidad de Cantabria, Santander, Spain

##### **Correspondence:** J. Madera Fernandez

*The Journal of Headache and Pain 2024,* **25(Suppl 1)**: P282

**Objective:** There are few published series of cluster headache (CH). Our aim was to longitudinally describe the epidemiological and clinical features of cluster headache (CH) patients in our region (Cantabria, Spain) since 1980.

**Methods:** Descriptive analysis of data from a CH registry created in 1980 in a tertiary hospital in Spain. Baseline clinical and demographic characteristics were calculated for the CH general population and specifically for episodic cluster headache (eCH) and chronic cluster headache (cCH) patients. A comparison of the different characteristics was also made between individuals with eCH and cCH. The analysis was performed with the statistical software SPSS. Statistical significance was set at *p*<0.05.

**Results:** 153 patients were diagnosed as CH (89,5% male). Mean age at the onset of symptoms was 32.8 years (mean deviation (MD) 11,3) and at diagnosis was 39.5 years (MD 11,4). There was a diagnostic delay of approximately 6 years (*p*= 0.00). 128 had eCH and 25 cCH. The proportion of men in eCH and cCH subgroup was 92.2 and 76%, respectively (*p*=0.027). Patients with cCH had a delay in the onset of symptoms compared to eCH of 5.74 years (*p*=0.023). In patients with eCH, the median duration of the cluster was 60 days with a median frequency every 2 years. At the time of diagnosis, 66% were smokers and 15% drinkers.

**Conclusion:** Considering the population of our Health Area, the theoretical prevalence of CH in our region is 0.05%. eCH was the most frequent phenotype. There is a delay in diagnosis of 6 years, which indicates an insufficient recognition of this entity. In our series, male predominance was higher than that described in the most recent literature, diminishing in cCH phenotype. A late onset and being female was associated with the development of a cCH.

**Table 1 (Abstract P282):**
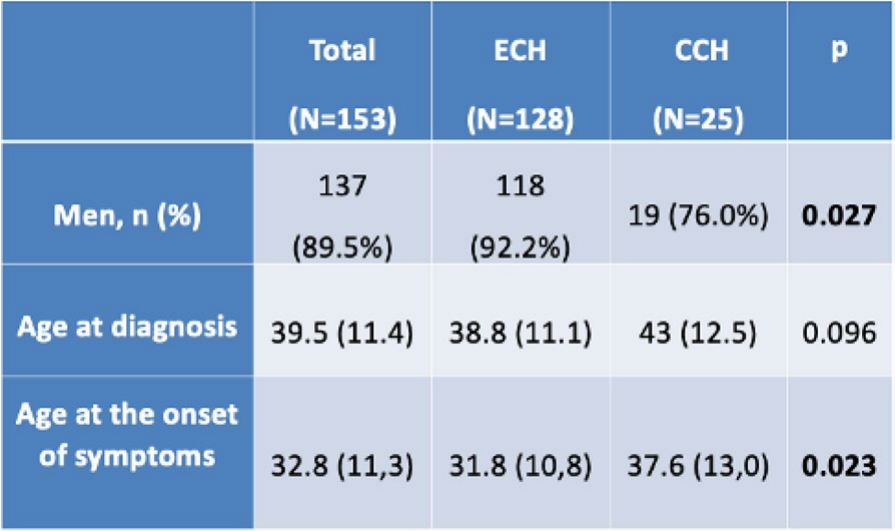
Main demographic characteristics of our CH series

**Fig. 1 (Abstract P282):**
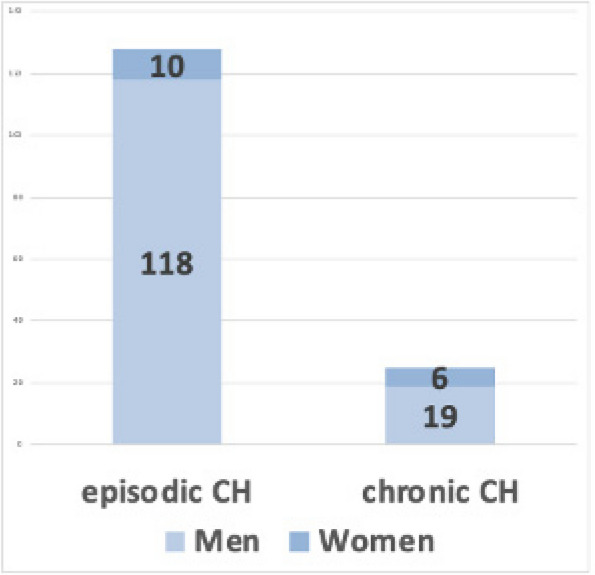
Distribution by diagnosis and sex of patients with CH

## P283 Diagnostic delay of trigeminal-autonomic cephalalgias in Armenia

### H. Vekilyan, H. Manvelyan

#### YSMU, Neurology, Yerevan, Armenia

##### **Correspondence:** H. Vekilyan

*The Journal of Headache and Pain 2024,* **25(Suppl 1)**: P283

**Objective:** Trigeminal- autonomic cephalalgias (TACs) are one of the rare types of primary headaches in which unilateral, mainly one-side locked headache accompanied by parasympathetic symptoms on the side of the pain. According to ICHD 3rd edition in this group are included following headaches: cluster headache (CH), paroxysmal hemicrania (PH), Short Lasting Unilateral Neuralgiform Headache Attacks (SUNCT/SUNA), hemicrania continua (HC). They mainly differ each from other by duration of pain attacks. Unfortunately, in many countries and also in Armenia these headaches are misdiagnosed, which cause serious delay and time lost for correct diagnosis. Main aim of our study was to estimate what is the time window between the onset of the headache and time when patient receive correct diagnosis.

**Methods:** We included 168 patients (116 women/ 52 men) addressed at Neurology Department of YSMU, from which 17 (9 male/ 6 female) was diagnosed TAC. Age of participants was 18-60 years. All patients were estimated by headache specialist and diagnosed according to ICHD -3 diagnostic criteria.

**Results:** Data analysis revealed that from 17 patients with TACs - 9 men diagnosed CH, 1 man probably CH, 4 women paroxysmal hemicrania, 1 men SUNCT, 2 women hemicrania continua correspondingly. CH, SUNCT previously was diagnosed as trigeminal neuralgia and in 9 cases of CH diagnosis was delayed for 12-20 years, in 2 cases for 2 years (main reason of last 2 cases that they addressed to headache specialist during their second cluster bout). PH and HC previously diagnosed as migraine and diagnostic delay was 6-10 years for PH and 5-7 years for HC correspondingly.

**Conclusion:** Summarizing our data, we concluded that main part of addressed patients were severely disabled because of their headache for many years and in Armenia we still have serious underestimation of TACs and we really need to make efforts for improve de situation mainly by spreading the knowledges among specialist and information among people.

## P284 Deleterious effect on morbimortality associated with the diagnosis of cluster headache: retrospective review of a Spanish series

### J. Madera Fernandez^1,2,3^, V. González-Quintanilla^1,2,3^, S. Pérez-Pereda^1,2,3^, A. González-Suárez^1,2,3^, G. Gárate^1,2,3^, N. Cavada Bustamante^1,2,3^, A. Fernández Rufino^1,2,3^, J. Pascual Gómez^1,2,3^

#### ^1^University Hospital Marqués de Valdecilla, Neurology, Santander, Spain; ^2^IDIVAL, Santander, Spain; ^3^Universidad de Cantabria, Santander, Spain

##### **Correspondence:** J. Madera Fernandez

*The Journal of Headache and Pain 2024,* **25(Suppl 1)**: P284

**Objective:** Cluster headache is one of the most disabling headaches that exist and is also the most frequent of the trigemino-autonomic cephalalgias. Poor lifestyle habits are often a comorbidity associated with this pathology. However, probably due to its low incidence, there are no studies on morbimortality in this pathology. Our objective was to study the years of potential life lost (YPLL) associated with this entity

**Methods:** A calculation of the YPLL among individuals with cluster headache in our series was performed by subtracting their age at death from the estimated life expectancy in our region for their sex and year of death. A descriptive analysis was made of the causes of death among individuals who died earlier than estimated for their sex and year of death. Finally, we compared the percentage of deaths due to cancer, cardiovascular disease and other causes among individuals with cluster headache who died earlier than expected and the general population of our region, as well as the percentage of smokers in both subgroups. The analysis was performed using SPSS software and statistical significance was considered if *p*<0.05.

**Results:** There were 25 deaths among the 162 patients registered. Twenty-one of them (84%) lived fewer years than the mean expected for their sex and year of death, with a mean of 13.7 years of potential life lost (SD 9.3). The most frequent cause of death was cancer, and it was significantly more frequent among individuals with cluster headache than in the general population of our region (68% vs. 28.5% *p*<0.001). The percentage of male smokers was significantly higher among individuals with cluster headache than in the general population of Cantabria (*p*=0.0095).

**Conclusion:** Deceased patients with cluster headache in our series had an average of almost 14 YPLL, usually due to cancer. Tobacco could play an essential causal role, so it is essential to establish measures aimed at controlling unhealthy lifestyle habits in this population.

**Table 1 (Abstract P284):**
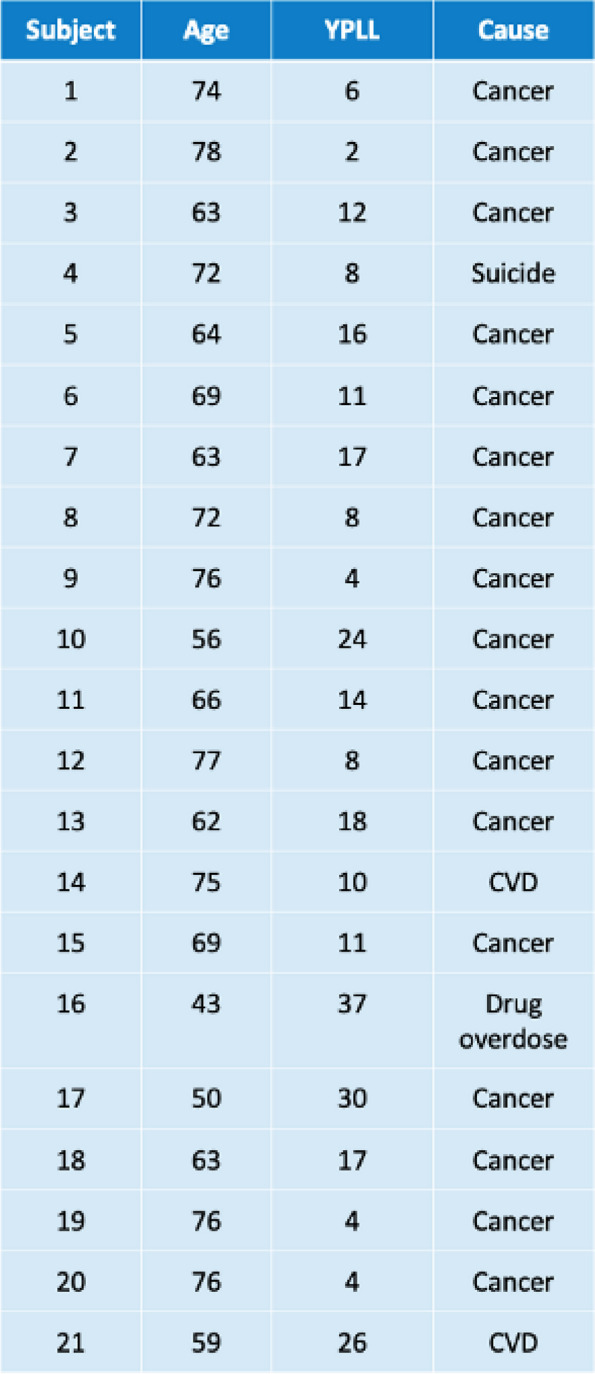
Death causes among CH patients who died before than expected

**Fig. 1 (Abstract P284):**
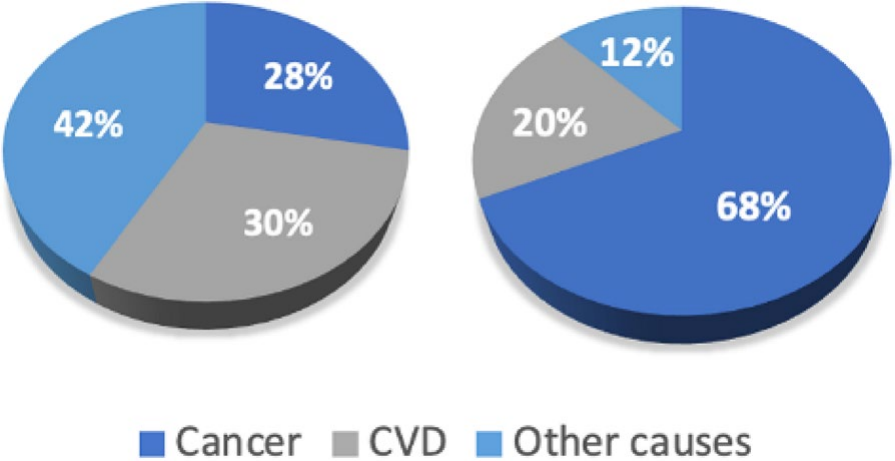
Main causes of death in Cantabria (Spain) Main causes of death in our registry

## P286 The efficacy of physical therapy and rehabilitation approaches in chronic migraine

### D. Onan^1^, E. Ekizoğlu^2^, H. Arıkan^3^, B. Taşdelen^4^, A. Özge^4^, P. Martelletti^5^

#### ^1^Hacettepe University, Ankara, Turkey; ^2^Istanbul University, İstanbul, Turkey; ^3^Gaziosmanpasa University, Tokat, Turkey; ^4^Mersin University Medical Faculty, Mersin, Turkey; ^5^Sapienza University, Rome, Italy

##### **Correspondence:** D. Onan

*The Journal of Headache and Pain 2024,* **25(Suppl 1)**: P286

**Objective:** Our aim was to investigate the efficacy of physical therapy and rehabilitation (PTR) approaches in the management of chronic migraine (CM). The research question is, what is the effect of physical therapy and rehabilitation approaches for CM on headache symptoms?

**Methods:** We conducted data synthesis of the headache-related outcomes assessed in randomized controlled trials (RCTs) performed in adults with CM and quantitative analysis of the eligible data. The outcomes of interest were intensity, frequency, and duration of headache, disability, and Quality of Life (QoL). We assessed also the methodological quality of the RCTs using the Physiotherapy Evidence Database (PEDro) scale.

**Results:** Seven RCTs were reviewed, and five of them were eligible for quantitative analysis. These trials employed various intervention arms, which included different modalities of physical therapy as follows: aerobic exercise, osteopathic manual therapy, occipital transcutaneous electrical stimulation, acupressure, hydrotherapy, instrument-assisted soft tissue mobilization, facial proprioceptive neuromuscular facilitation, and connective tissue massage. The methodological qualities of the trials were generally good to excellent. Overall, the physical therapy and rehabilitation arms showed more favorable outcomes compared to the control or medical therapy alone arms. The quantitative analysis showed the combined therapy with physical therapy was more effective to improve the intensity of headache and to reduce headache days per month (p=0.047) in comparison to standard management of migraine alone.

**Conclusion:** The PTR approaches show promise in improving headache, disability, and QoL related to CM. Meta-analysis of the eligible data supported favorable outcomes for both intensity and headache days per month. Further research is needed to better understand the efficacy and to define the optimal duration of physical therapy and rehabilitation approaches in CM.

**Fig. 1 (Abstract P286):**
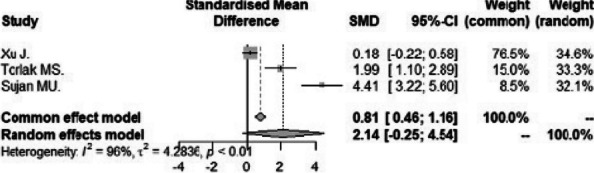
See text for description

**Fig. 2 (Abstract P286):**
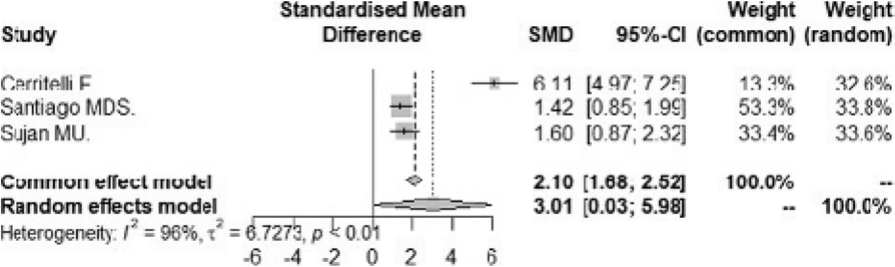
See text for description

## P287 Studying physical and psychological barriers and limitations to exercise in chronic migraine patients

### M. Randall^1^, S. Waters^2^, A. Ballario^2^

#### ^1^Leeds Teaching Hospitals NHS, Department of Neurology, Leeds, United Kingdom; ^2^Leeds Teaching Hospitals NHS, Leeds, United Kingdom

##### **Correspondence:** M. Randall, S. Waters, A. Ballario

*The Journal of Headache and Pain 2024,* **25(Suppl 1)**: P287

**Objective:** The aim of this study was to determine physical and psychological barriers responsible for low exercise uptake in migraine patients. We aimed to evaluate if migraine patients attending outpatient clinics are routinely enquired and followed-up on their physical activity habits, in line with the recommended holistic management of chronic migraine sufferers. In addition, we explored the extent of education provided on the relationship between migraine and physical activity, as a potential prophylactic intervention

**Methods:** The data consisted of 50 sets of clinical notes from chronic migraine patients currently on the most expensive pharmacological migraine treatment: CGRP drugs. Participants selected were current patients at the Neurology department at Leeds General Infirmary hospital. The second set of data included 14 survey responses distributed to consultants and specialty trainees running clinics for chronic migraine participants. The notes were audited against the National Institute for Health and Care Excellence (NICE) guideline standards for migraine management.

**Results:** NICE guidelines are not currently met. Although 86% of doctors surveyed believed that discussion around lifestyle has a role in the management of patients with migraine, only 6% routinely discuss physical activity at consultations. Clinicians reported limited time, unfamiliarity with nonpharmacological management approaches, and fear of offending patients as the main barriers.

**Conclusion:** This study suggests neurologists" routine practices in migraine management at the point of care are sub optimal. Interventions aimed at improving the quality of clinician"s discussions around physical activity with patients are necessary, in order to overcome barriers and diminish the burden of migraine

## P289 Consensus recommendations on the role of nurses in the care of headache patients: a european e-Delphi study

### A. V. Rasmussen^1^, R. H. Jensen^2^, L. Eklund Karlsson^3^, L. S. Mose^4^

#### ^1^Zealand University Hospital, Department of Neurology, Roskilde, Denmark; ^2^Copenhagen University Hospital – Rigshospitalet, Danish Headache Center and Department of Neurology, Copenhagen, Denmark; ^3^Unit for Health Promotion Research, University of Southern Denmark, Denmark, Esbjerg, Denmark; ^4^Esbjerg Hospital, University Hospital of Southern Denmark, Department of Neurology, Esbjerg, Denmark

##### **Correspondence:** L. S. Mose

*The Journal of Headache and Pain 2024,* **25(Suppl 1)**: P289

**Objective:** At the European headache centers nurses contribute largely to the migraine treatment. A unified definition of nursing tasks and conduction of tasks is lacking. The objectives are: 1. To obtain opinions from headache experts on tasks associated with nurses" care in migraine treatment. 2. To analyze for group consensus and develop evident European nursing recommendations.

**Methods:** This e-Delphi study contains three rounds of e-questionnaires. Nurses (*n*=18) and neurologists (*n*=10) working in specialized headache centers in Finland, Denmark, Norway, Sweden, United Kingdom, Netherlands, Germany, Ireland, Estonia, and Switzerland participated anonymously in the expert panel. Open-ended questions captured the essentials of nurse tasks as understood by the expert panel members. Predefined statements, synthesized from a systematic examination of the existing literature, were applied for the experts to rate the importance of nurses" tasks. Consensus was measured using descriptive statistics. Measurement of agreement among participants were analyzed using inferential statistics; Kendall"s coefficient and stability between the rounds; Wilcoxon rank-sum test. Statements receiving consensus (≥65% consistency in the answers) after the third round, are included in the final recommendations.

**Results:** Twenty-one responded (75%). Data analyzes were commenced and forty-five tasks received consensus to constitute the recommendations. The included tasks were divided into four themes: *Role in the clinical setting, Follow-up on treatment, The organizing frame,* and *Education/support* (See Table 1).

**Conclusion:** Unified European recommendations provide a basis for tasks and management for nurses working with migraine patients. For the benefit of patients and nurses, implementing the recommendations in a structured way will increase quality of nursing care and enable further research into the nurse's role in the headache field.

**Table 1 (Abstract P289):**
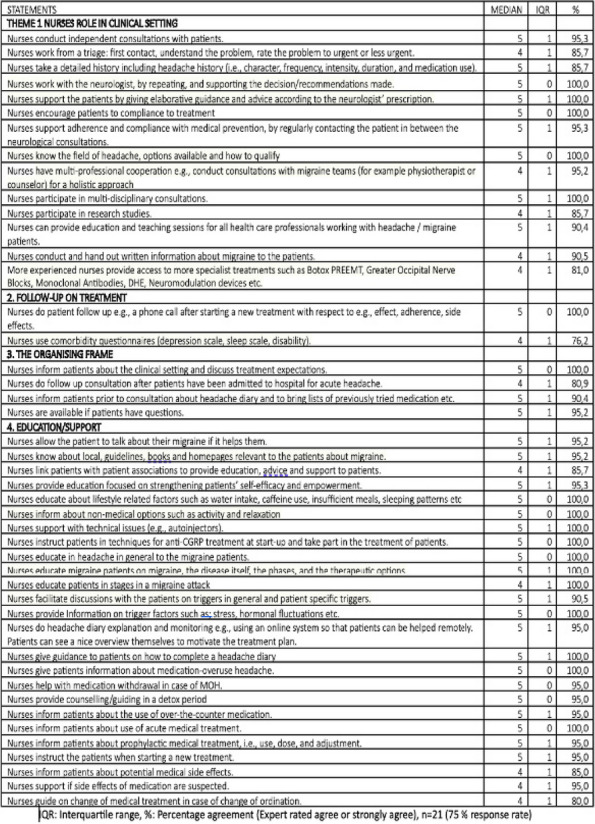
Statements with ≥ 65% agreement in the expert panel after third round of questionnaires

## P291 Headache in relation to uncorrected refractive errors, screen use, physical activity, and musculoskeletal pain in Norwegian adolescents

### H. M. S. Thorud, I. H. H. Minsås, T. Langaas, G. H. Vikesdal, C. O. Bjørset, S. J. Gilson, L. A. Hagen, T. R. Johansen, H. R. Pedersen, E. Svarverud, R. C. Baraas

#### University of South-Eastern Norway, Department of Optometry, Radiography and Lighting Design, Kongsberg, Norway

##### **Correspondence:** H. M. S. Thorud

*The Journal of Headache and Pain 2024,* **25(Suppl 1)**: P291

**Objective:** Headache and musculoskeletal pain are leading causes of sickness absence, and largely affect quality of life. An increased prevalence is seen in children concurrently with a more sedentary lifestyle and increased time on digital screens. The load on the visual system and head-stabilizing musculature in the upper body increases with extensive near visual tasks. Uncorrected vision problems may cause headache and musculoskeletal pain, and up to 40 % of school children have undetected vision problems. This study aimed to investigate the relationship between headache, uncorrected refractive errors, need for eye examinations, musculoskeletal pain, physical activity, and time spent on digital screens.

**Methods:** In this cross-sectional study 143 fifth and tenth grade children participated. Vision data was collected as part of a school vision testing program in Kongsberg, Norway. A questionnaire was used to collect data regarding headache, musculoskeletal pain, physical activity, and digital screen use.

**Results:** Headache frequency was significantly associated with uncorrected vision and the need for eye examinations, and there were significant associations between headache type and uncorrected refractive errors. Further, headache frequency was positively correlated with female sex, increased age, smartphone time, and musculoskeletal pain, and headache intensity was positively correlated with smartphone and laptop time. The presence of uncorrected refractive errors, increased shoulder pain frequency, and increased physical activity was negatively correlated with headache intensity.

**Conclusion:** This study describes associations between headache, musculoskeletal pain, uncorrected refractive errors, screen time, and physical activity, and highlights the importance of eye examinations in children with headache.

## P292 Effectiveness of transcranial direct current stimulation and monoclonal antibodies acting on the CGRP as a combined treatment for migraine (TACTIC) – preliminary results

### C. Rosignoli^1^, R. Ornello^1^, A. D'Atri^1^, F. De Santis^1^, V. Caponnetto^1^, F. Salfi^1^, D. Corigliano^1,2^, R. De Icco^3,4^, V. Grillo^3,4^, M. Corrado^3,4^, F. Bighiani^3,4^, G. Vaghi^3,4^, G. Sances^4^, M. Ferrara^1^, C. Tassorelli^3,4^, S. Sacco^1^

#### ^1^University of L'Aquila, Department of Biotechnological and Applied Clinical Sciences, L'Aquila, Italy; ^2^University of Rome, Sapienza, Rome, Italy; ^3^University of Pavia, Department of Brain and Behavioral Sciences, Pavia, Italy; ^4^IRCCS Mondino Foundation, Headache Science and Neurorehabilitation Centre, Pavia, Italy

##### **Correspondence:** C. Rosignoli

*The Journal of Headache and Pain 2024,* **25(Suppl 1)**: P292

**Objective:** To assess whether Transcranial Direct Current Stimulation (tDCS), as an add-on treatment to Monoclonal Antibodies Acting on the Calcitonin Gene Related Peptide (CGRP-MAbs), improves migraine prevention. We also measured electroencephalographic (EEG) power changes to estimate the biological effects of this combined therapeutic approach.

**Methods:** TACTIC (NCT05161871) 1 is an ongoing randomized, double-blind, multicenter, sham-controlled trial including patients with migraine treated with CGRP-MAbs for ≥90 days and still reporting ≥8 monthly migraine days. We performed a 5-day tDCS protocol with bilateral electrodes positioned on occipital and primary motor areas (sham/active sessions lasting 20 minutes) and we followed up patients for 28 days. We also recorded 64-channel EEG at day 1 (pre-stimulation) and day 5 (end of stimulation). We analyzed change in monthly migraine days, clinical scales, and EEG changes in spectral power in the delta (2-4Hz), theta (5-7Hz), alpha (8-12Hz) and beta bands (13-30Hz).

**Results:** We included 13 patients (mean age=46.2 ±12.5, 92.0% female), 7 in active session and 6 in sham. tDCS led to a decrease in monthly migraine days that was more pronounced – although not significant – in the active group (mean difference 3.57, standard error (SE)=1.91, *p*=0.089). An improvement in the HADS-D scale for depression was found in the active group (mean difference 2.85, SE=0.85 *p*=0.007) compared with sham (mean difference 1.3, SE=0.92, *p*=0.17). tDCS induced a significant reduction in the alpha band power and a decrease in delta power over the motor areas only in the active stimulation group (both *p*<0.05); no difference was found in the sham group.

**Conclusion:** We described a clinical improvement, although not significant from the combination of tDCS with CGRP-MAbs. The strong peripheral action of CGRP-MAbs might hinder subtle clinical effects of tDCS. These results must be taken with caution due to the small sample size recruited to date or to the short duration of the stimulation period. According to our preliminary results, tDCS changed basal cortical activity on stimulated areas without any significant improvement in headache in patients treated with CGRP-MAbs.

## P293 Combined supra-orbital and occipital nerve stimulation: Early results with a novel craniofacial implantable neurostimulator for the treatment of refractory chronic migraine

### M. Goethals^1^, B. Billet^2^, K. Hanssens^2^, R. De Vos^3^, B. Tsang^4^, L. Simmonds^5^, M. Matharu^5^

#### ^1^AZ Delta Campus Torhout, Neurology, Torhout, Belgium; ^2^AZ Delta, Anaesthesia Pain Centre, Roeselare, Belgium; ^3^AZ Delta, Intensive Care Pain Centre, Roeselare, Belgium; ^4^Beach Brain, Neurology, Birtinya, Australia; ^5^The National Hospital for Neurology and Neurosurgery, Headache and Facial Pain Group, London, United Kingdom

##### **Correspondence:** L. Simmonds

*The Journal of Headache and Pain 2024,* **25(Suppl 1)**: P293

**Objective:** Chronic migraine (CM) is a debilitating neurological disorder characterised by recurrent, disabling headaches. The pathophysiology of CM is complex and involves multiple factors, including central sensitisation, cortical spreading depression, neurogenic inflammation, and dysfunctional pain modulation mechanisms. Despite the availability of numerous treatment options for CM, there remains a substantial unmet need for effective therapies, underscoring the importance of progressive therapeutic approaches such as combined supra-orbital and occipital nerve stimulation.

**Methods:** A novel implantable neurostimulator system has been developed for use in the craniofacial region. The system comprises two implants, each with an ultra-thin lead connected to an integrated, battery-free stimulator. One implant covers the left and right supra-orbital nerves, while the other covers the left and right occipital nerves. The system is implanted during a short procedure that is minimally invasive and non-traumatic, using specialised surgical instruments designed for this device implantation.

**Results:** We present the early surgical and 3 months clinical outcomes of an ongoing early feasibility study evaluating the safety and efficacy of this novel craniofacial implantable neurostimulator to treat refractory CM using combined supra-orbital and occipital nerve stimulation. The study eligibility criteria required the failure of three or more preventive therapies, including CGRP mabs or Onabotulinumtoxin A.

**Conclusion:** In conclusion, the complex pathophysiology of migraine suggests the importance of targeting multiple pain pathways simultaneously. If proven effective, this innovative neurostimulator will provide a new therapeutic option for preventing CM attacks, offering relief and improving the quality of life of individuals with refractory CM.

## P294 Combined supra-orbital and occipital nerve stimulation: early results with a novel craniofacial implantable neurostimulator for the treatment of refractory chronic cluster headache

### P. Frank^1^, B. Tsang^2^, M. Goethals^3^, L. Simmonds^4^, M. Matharu^4^

#### ^1^Resolve Pain, Anaesthesia Pain Centre, Buderim, Australia; ^2^Beach Brain, Neurology, Birtinya, Australia; ^3^AZ Delta, Neurology, Torhout, Belgium; ^4^The National Hospital for Neurology and Neurosurgery, Headache and Facial Pain Group, London, United Kingdom

##### **Correspondence:** L. Simmonds

*The Journal of Headache and Pain 2024,* **25(Suppl 1)**: P294

**Objective:** Refractory chronic cluster headache (CCH) is a debilitating condition characterised by severe, unilateral headache pain accompanied by autonomic symptoms. Despite available treatments, some patients continue to experience frequent and intense attacks, significantly impacting their quality of life. These individuals often face limited therapeutic options and require alternative interventions.

**Methods:** The development of a novel craniofacial implantable neurostimulator represents a potential breakthrough in managing refractory CCH. The system comprises two implants, each with an ultra-thin lead connected to an integrated, battery-free stimulator. One implant covers the left and right supra-orbital nerves, while the other covers the left and right occipital nerves. The system is implanted during a short outpatient procedure that is minimally invasive and non-traumatic, using specialised surgical instruments designed for this device implantation.

**Results:** We present the early surgical and 3 months clinical outcomes of an ongoing first-in-human study evaluating the safety and efficacy, initial programming parameters, differences in quality of life scores, and the initial user experience with the therapy.

**Conclusion:** In conclusion, the multifactorial pathophysiology of CCH highlights the significance of simultaneously targeting multiple pain pathways. If demonstrated to be effective, this innovative neurostimulator will introduce a promising therapeutic solution for the prevention and acute treatment of cluster headache attacks, delivering substantial pain relief and enhancing the quality of life of individuals with refractory CCH.

## P295 Classification of patients with migraine regarding the pain curve of a migraine attack and demographic characteristics

### A. González Martínez^1^, Á. Martínez-Petit^2^, J. Gálvez-Goicuría^3,2^, J. Pagán^4,2^, S. Quintas Gutierrez^1,3^, A. Vieira^1,3^, J. L. Ayala^4,5^, A. Sanz^6^, M. Sobrado^1^, J. Vivancos^1^, A. Gago-Viega^1^

#### ^1^Hospital Universitario de la Princesa, Neurology, Madrid, Spain; ^2^Electronic Engineering Department, Madrid, Spain; ^3^Brainguard SL, Pozuelo de Alarcón, 28223, Madrid, Spain, Spain; ^4^CCS: Center for Computational Simulation, Madrid, Spain; ^5^Computer Architecture and Automation Department, Madrid, Spain; ^6^Data Analysis Unit, Hospital Universitario de la Princesa, Madrid, Spain

##### **Correspondence:** A. González Martínez

*The Journal of Headache and Pain 2024*, **25(Suppl 1):**P295

**Objective:** Previous studies have identified the clinical characteristics of a migraine attack; moreover, previous researched performed in our group has classified the migraine attacks in 4 types according to the main characteristics of the pain curve: 1 (high intensity), 2 (acute onset), 3 (prolonged and intense) y 4 (low intensity). However, weather there are different types of patients considering both the clinical characteristics and the pain curve has not been explored yet.

**Methods:** In this study we evaluated the presence of patient types regarding their pain curve type. Patients included in the study were patients with episodic migraine (EM) according to the current criteria (ICHD-III) who participated in a prospective real time study using a smartphone application. To generate the model K-means and a supervised validation technique using a logistic model tree was used.

**Results:** A total 51 patients (mean age 39 years, 90.2% women) and 344 migraine crisis were analyzed. Patients were classified in five groups: A (high intensity), B (high intensity, sudden onset, prolonged and intense), C (acute onset), D (prolonged and intense) y E (low intensity). Group A had low-education, lower cognitive reserve, and migraine with aura. Group B, C and E had higher cognitive reserve. Group E were patients with migraine onset under 10 years old There were no other features clearly present regarding the patient type in terms of triggers or concomitant symptoms.

**Conclusion:** The evolution of the pain crisis can be successfully categorized in 4 types of curves and 5 types of migraine patients, which highlights a new possible way of migraine classification. The relationships found need further attention.

## P296 External validation of the predictive model of response to anti-CGRP monoclonal antibodies in patients with migraine

### A. González Martínez^1^, C. Sánchez-Rodríguez^1^, J. Pagán^2,3^, I. Fernández Lázaro^1^, J. S. Rodrígue-Vico^4^, A. Jaimes Sanchez^4^, A. Gómez García^4^, J. Casas Limón^5^, J. Díaz de Terán^6^, M. Sastre Real^6^, J. A. Membrilla López^6^, G. Latorre^7^, C. Calle de Miguel^7^, S. Gil Luque^8^, C. Trevino-Peinado^9^, S. Quintas Gutierrez^1,9^, P. Heredia^1,9^, D. García Azorín^10^, A. Echavarría Íñiguez^10^, Á. L. Guerrero Peral^10^, Á. Sierra-Mencía^10^, N. González García^11^, J. Porta-Etessam^11^, A. Gago-Viega^1^

#### ^1^Hospital Universitario de la Princesa, Neurology, Madrid, Spain; ^2^CCS: Center for Computational Simulation, Madrid, Spain; ^3^Electronic Engineering Department, Madrid, Spain; ^4^Headache Unit, Neurology Department, Madrid, Spain; ^5^Headache Unit, Neurology Department, Alcorcón, Spain; ^6^Headache Unit, Neurology Department, Madrid, Spain; ^7^Headache Unit, Neurology Department, Hospital Universitario de Fuenlabrada,, Fuenlabrada, Spain; ^8^Headache Unit, Neurology Department, Burgos, Spain; ^9^Headache Unit, Neurology Department, Madrid, Spain; ^10^Headache Unit, Neurology Department, Hospital Clínico Universitario de Valladolid, Valladolid,, Valladolid, Spain; ^11^Headache Unit, Neurology Department, Madrid, Spain

##### **Correspondence:** A. González Martínez

*The Journal of Headache and Pain 2024*, **25(Suppl 1):**P296

**Objective:** Predicting the response to recent anti-CGRP therapies is a topic of interest in the field of migraine. Previous studies carried out in our group have developed a predictive tool for response to anti-CGRP using an approach based on machine-learning techniques. The aim of this study was to validate this tool and its usefulness in patients with chronic (CM) and episodic (ME) migraine.

**Methods:** Multicenter, retrospective cohort study, with patients with migraine from 9 Headache Units, different from the model generation cohort. Sensitivity(S), specificity (E) and global positive (PPV) and negative (NPV) predictive values and for the different groups were obtained.

**Results:** 127 patients with migraine were included, 104 (81.88%) with CM, 108 (85.03%) women and mean age 53.73 (SD 13.84) years. In the evaluation of the algorithm of global response greater than 50% at 6 months, the global S was 78.04% and the global E was 80%. The area under the curve (AUC) was 0.790, CI[0.726-0.849) and weighted F1 of 79% in the validation cohort, with an AUC of 0.819 CI[0.762-0.884] and weighted F1 of 81.88% in MC and AUC of 0.592 CI[0.322-0.842] with a weighted F1 of 68.97% in ME.

**Conclusion:** Our study confirms the external validity of the predictive model of response to anti-CGRP in a different cohort from the generation of the algorithm. The S and E of the predictive model were higher in the group of patients with CM. Future models could improve the predictive capacity of this tool in patients with ME.

## P297 Multicenter investigation of AI Systems for headache diagnosis: advancing clinical approaches

### S. Ulutas^1^, B. Caliskan^2^, S. Mumcu^2^, H. Genç^3^, D. Uluduz^4^, A. Özge^5^, H. B. Yilmaz^2^, O. T. B. Hamit Genc Betul Baykan Hayrunnisa B^6^

#### ^1^Bahcesehir University Medical Faculty, Neurology, İstanbul, Turkey; ^2^Bogazici University, Dept. of Computer Engineering, İstanbul, Turkey; ^3^Gaziantep Dr. Ersin Arslan Training and Research Hospital, Neurology, Gaziantep, Turkey; ^4^Cerrahpasa University Medical Faculty, Neurology, İstanbul, Turkey; ^5^Mersin University Medical Faculty, Neurology, Mersin, Turkey; ^6^University of Health Sciences, Neurology, Van, Turkey

##### **Correspondence:** S. Ulutas

*The Journal of Headache and Pain 2024*, **25(Suppl 1):**P297

**Objective:** Headache is a common and disruptive symptom necessitating accurate diagnosis for effective treatment decisions. Artificial intelligence (AI) systems hold promise in this area, utilizing machine learning algorithms. This study aims to compare two different AI learning methods by exploring the application of AI systems in headache diagnosis.

**Methods:** Dataset from 69 neurologists utilized to estimate the characteristics of primary headaches, focusing on diagnosis. In order to assess the model's performance, K-fold cross-validation was employed, dividing the data into train and test datasets. As for the methods preferred, we applied the isolation forest method for outlier elimination since data may include various types of errors. Additionally, we focused on balancing the class cardinalities by data augmentation to eliminate the bias during learning. We applied decision trees and fully connected artificial neural networks (AAN) methods. For the performance evaluation, we considered confusion matrix, accuracy, precision, and recall metrics.

**Results:** The AAN method achieved an Accuracy_All 56.1% and Accuracy_Binary 81.5% and decision tree method achieved an Accuracy_All %62 and Accuracy_Binary 85% in diagnosing primary headache subtypes which can be improved by hyperparameter tuning. Precision and recall scores indicated the models" reliable performance in correctly identifying headache classes.

**Conclusion:** This study highlights the potential of AI systems in enhancing headache diagnosis. It is the first time different learning methods of AI have been used to diagnose headache subtypes in a diverse randomized dataset. The most common problem was the small quantity of rare headaches compared to a huge number of more common headaches. The high accuracy rates and robust performance metrics validate the effectiveness of AI models in identifying different primary headache subtypes. To improve diagnostic accuracy in future studies, increasing the sample size for each headache group may be beneficial.

**Fig. 1 (Abstract P297):**
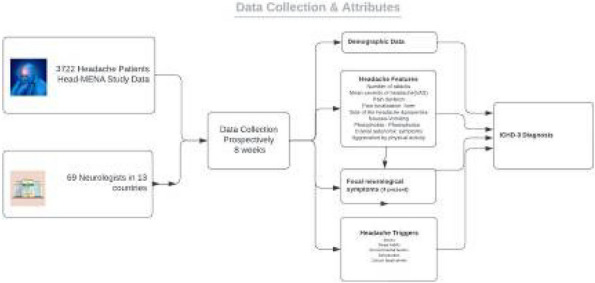
See text for description

**Table 1 (Abstract P297):**
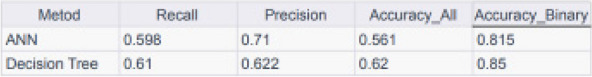
See text for description

**Fig. 2 (Abstract P297):**
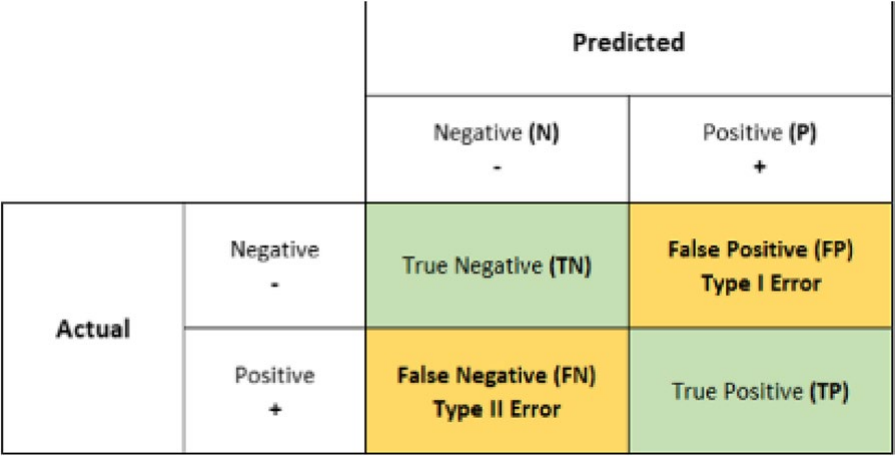
See text for description

## P298 Occipital nerve stimulation as an alternative treatment for hemicrania continua: a systematic review

### F. Wolff Fernandes^1^, G. Duarte^2^

#### ^1^Hannover Medical School, Department of Neurosurgery, Hannover, Germany; ^2^University Hospital of Zurich, Department of Neurology, Zurich, Switzerland

##### **Correspondence:** F. Wolff Fernandes

*The Journal of Headache and Pain 2024*, **25(Suppl 1):**P298

**Objective:** Hemicrania continua (HC) is a disabling primary headache defined as a unilateral continuous headache with ipsilateral cranial autonomic features. Despite its known sensitivity to indomethacin, some patients have contraindications or are unable to tolerate this treatment. Occipital nerve stimulation (ONS) is an effective and safe treatment option for medically intractable headache disorders. The aim of this study was to examine the efficacy and safety described in the existing literature concerning ONS in HC.

**Methods:** A systematic literature review of articles describing the use of ONS in HC was conducted by two independent authors. Each article was evaluated for study type, number of treated patients, type of headache, mean intensity of pain and frequency of headaches at baseline and at follow-up, and adverse effects.

**Results:** Out of the 80 studies for inclusion, 7 relevant full-text articles met the eligibility criteria. We identified 4 prospective cohort studies, 2 retrospective cohort studies, and one case report. A total of 29 patients were identified, with a mean age of 50,6 ± 3,3 years at the time of surgery. Eight patients (27,6%) suffered from an additional type of headache. The visual analogue scale improved by 2,9 out of 10,0 points (a mean reduction of 45,3%) during an average follow-up of 29,2 months. The frequency of headaches decreased from an average of 29,3 to 19,9 headaches per month (a mean reduction of 33,4%). A 50% or higher response rate was reported in 16 out of 27 patients (59,3%). Reported adverse effects included 4 cases of overstimulation, and three patients required at least one lead revision.

**Conclusion:** These findings provide evidence supporting ONS as an effective alternative to decrease the intensity and frequency of headaches in patients with HC who do not tolerate indomethacin. Future work should prioritize controlled trials and a standard description of outcomes in patients with HC.

## P301 Post-traumatic stress differ between those who present with headache and those who do not soon after a whiplash injury: a case-control study

### E. Anarte-Lazo^1,2^, M. Barbero^3^, D. Falla^2^, C. Bernal-Utrera^1^, C. Rodriguez-Blanco^1^

#### ^1^University of Seville, Physiotherapy Department, Sevilla, Spain; ^2^University of Birmingham, Centre of Precision Rehabilitation for Spinal Pain, Birmingham, United Kingdom; ^3^University of Applied Sciences and Arts of Southern Switzerland, 2rLab Rehabilitation Research Laboratory, Manno, Switzerland

##### **Correspondence:** E. Anarte-Lazo

*The Journal of Headache and Pain 2024*, **25(Suppl 1):**P301

**Objective:** To evaluate whether patients who develop a headache shortly after a whiplash injury show higher values of post-traumatic stress disorder when compared to those without headache.

**Methods:** A case-control study was conducted between March 2022 and June 2023, including patients between 18 and 65 years old diagnosed with suffering from whiplash-associated disorders (WAD) grade II, according to the Quebec Task Force. We excluded patients who had a previous headache, were evaluated more than 30 days after the whiplash injury, and/or had a serious disease or congenital disturbance. Post-traumatic stress was evaluated through the Spanish version of the Impact of Event Scale-Revised (IES-R). Before the evaluation, they were advised that the results from this assessment would not be considered as part of any insurance claim.

**Results:** 61 patients were recruited, and 7 were excluded. Thus, 54 patients were included in our study, 34 of whom were women. Among them, 30 presented with a headache (55.5%). The mean age was 35.48 (SD: 12.46). The mean value for the headache group was 38.80 (SD: 17.87). A significant difference was observed between the groups (*p* < 0.001; Mean Difference = -19.51 [95% CI -28.23 to -10.78]). No differences were found when the groups were grouped by sex (*p* = 0.367).

**Conclusion:** Higher values of post-traumatic stress were observed in participants with acute whiplash-associated headache when compared to those who did not develop a headache. These findings may be useful for clinicians who should consider this factor for a comprehensive management of this population.

## P302 Safety and efficacy of greater occipital nerve block in migraine: a systematic review, meta-analysis and meta-regression of randomized controlled trials

### A. Cyntia Lima Fonseca Rodrigues^1,2^, B. Kraychete^3^, G. M. Ali Malik^4^, D. Shivakumar^5^, D. Wankhade^6^, S. G.C.^7^

#### ^1^Positivo University, Department of Medicine, Curitiba, Brazil; ^2^Anhembi Morumbi University, Department of Statistics, Curitiba, Brazil; ^3^Children’s Hospital Los Angeles, Los Angeles, CA, United States; ^4^Dow International Medical College, Karachi, Pakistan; ^5^Kamineni Academy of Medical Sciences and Research Centre, Hyderabad, India; ^6^Terna Medical College, Navi Mumbai, India; ^7^Enam Medical College and Hospital, Dhaka, Bangladesh

##### **Correspondence:** A. Cyntia Lima Fonseca Rodrigues

*The Journal of Headache and Pain 2024*, **25(Suppl 1):**P302

**Objective:** This study aims to evaluate the safety and efficacy of greater occipital nerve block (GONB) in patients with migraine.

**Methods:** PubMed, Embase, Web of Science and Ovid databases were searched through June 2023. Statistical analysis was performed using R version 4.3.1. Random-effects meta-analysis were estimated using the inverse variance with Mantel-Haenszel method and DerSimonian-Laird estimator. The outcomes assessed were: headache severity (HS), headache frequency (HF) and adverse events (AEs).

**Results:** We included 12 RCTs involving 540 patients. GONB resulted in a significant reduction in both HS (MD -1.42, 95% CI -1.95 to -0.89, *p*<0.001, I^2^ = 60%) and HF (MD -3.68, 95% CI -6.74 to -0.662, *p*=0.019, I^2^ = 89%). GONB did not significantly affect the risk of adverse events (RR 0.97, 95% CI 0.68 to 1.38, *p*=0.876, I^2^ = 0%). The meta-regression evaluated the statistical impact of the mean age in HS, which was (estimate 0.0515, *p*=0.8131, 95% CI -0.37 to 0.47). Sensitivity analysis was performed for all outcomes to strengthen the reliability of the analysis, no single study was identified as a remarkably influential study. Graphical distribution of the funnel plot shows no evidence of asymmetry in the assessed outcomes.

**Conclusion:** The meta-analysis found that GONB may be an effective treatment option for reducing HS and HF without increasing AEs, although further high-quality studies are recommended to confirm these findings and explore the source of outcome heterogeneity. The meta-regression found that mean age did not significantly affect HS.

**Fig. 1 (Abstract P302):**
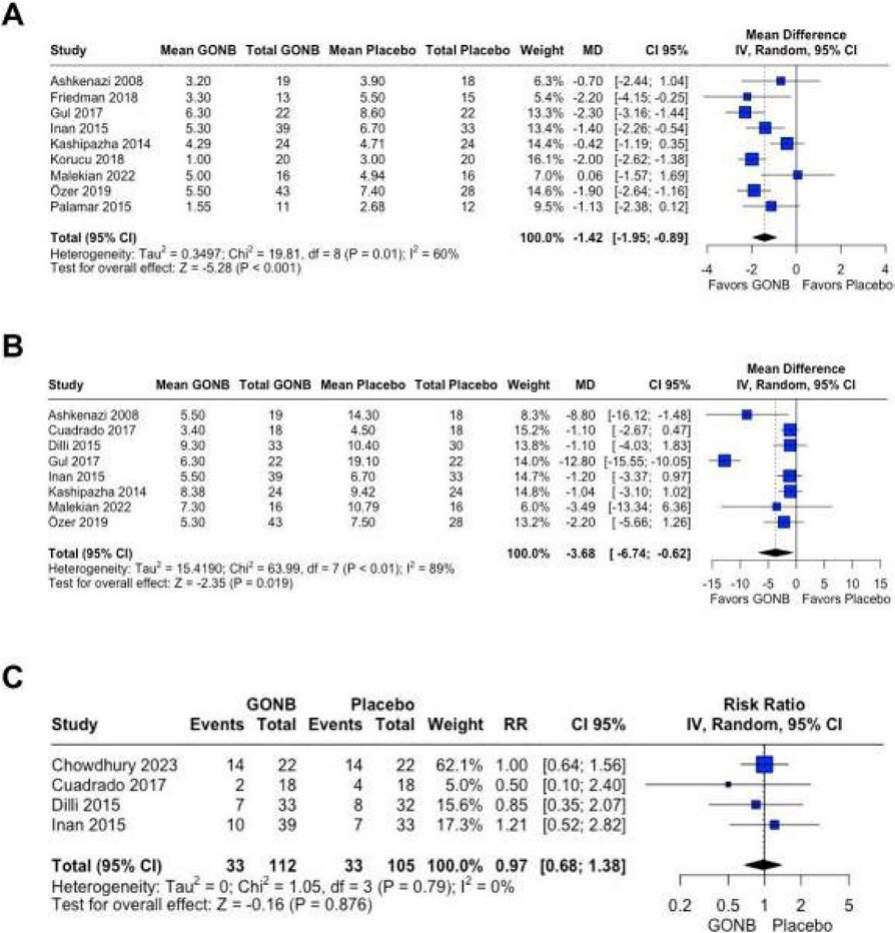
Forest plot outlining random effects meta-analysis for the outcomes **a** Headache Severity, **b** Headache Frequency, **c** Adverse Events

**Fig. 2 (Abstract P302):**
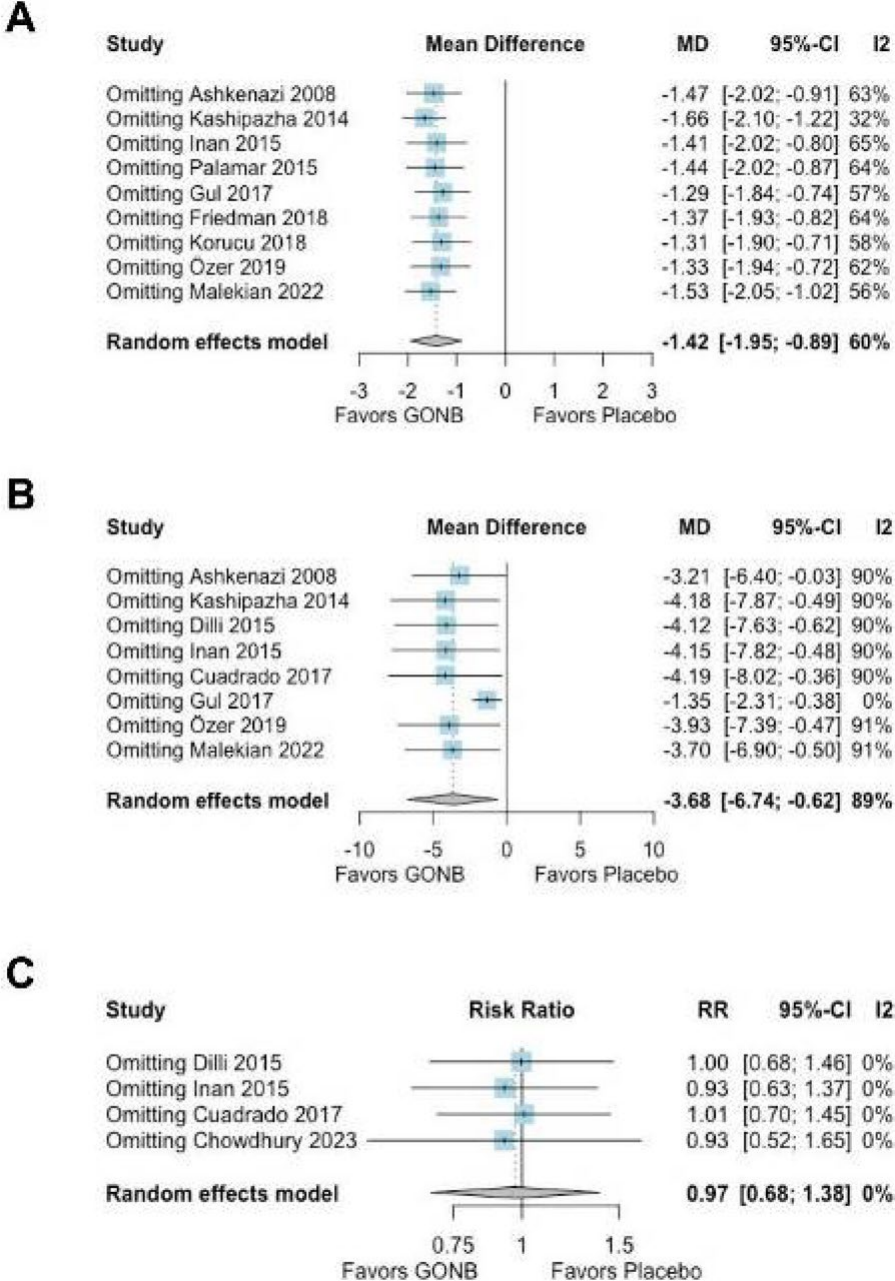
Sensitivity Analysis (Leave-one-out Method) for the outcomes **a** Headache Severity, **b** Headache Frequency, **c** Adverse Events

**Fig. 3 (Abstract P302):**
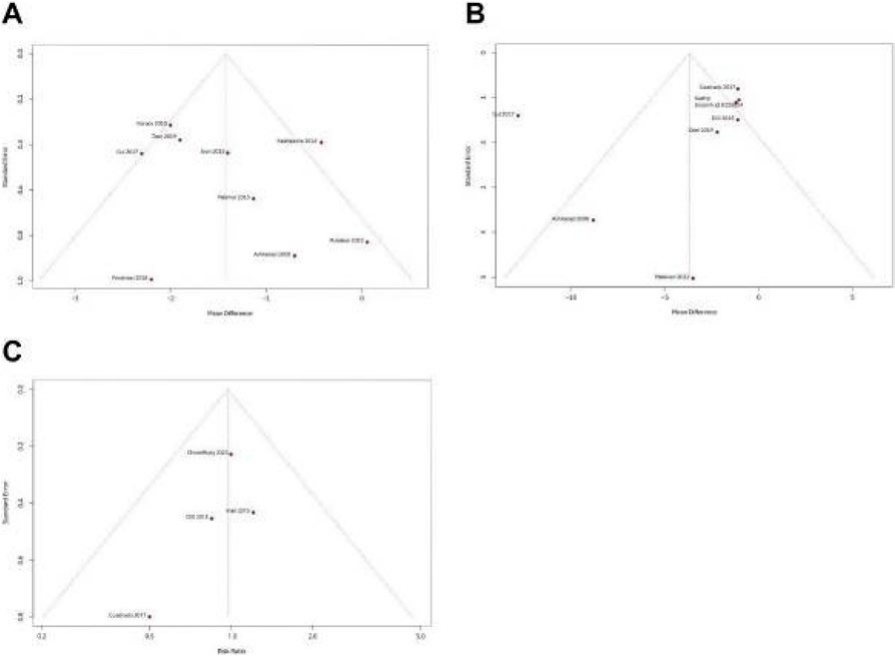
Sensitivity Analysis (Leave-one-out Method) for the outcomes **a** Headache Severity, **b** Headache Frequency, **c** Adverse Events

**Fig. 4 (Abstract P302):**
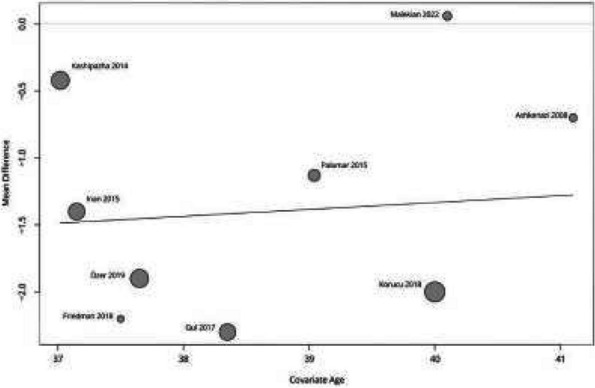
Bubble pot of meta-regression analysis of covariate age for headache severity

## P303 An interesting case of secondary headache

### A. Dubey^1^, S. Dubey^2^

#### ^1^GMC & Hamidia Hospital, Medicine, Bhopal, India; ^2^AIIMS, New Delhi, Neurology, Bhopal, India

##### **Correspondence:** A. Dubey

*The Journal of Headache and Pain 2024*, **25(Suppl 1):**P303

**Objective:** Headache is one of the most disabling conditions faced in daily routine. There are many causes of secondary headache documented in literature. We describe an interesting case here.

**Methods:** A 55 year old obese male presented to outpatient department with complaints of morning headaches since past 5 months. There was no papilloedema and no neuro deficits. He underwent brain imaging which was normal. Considering a diagnosis of IIH without papilloedema, he was started on acetazolamide with no benefit in next 2 months.

**Results:** Considering no response to therapy, the case was reviewed. Patient gave history of snoring for last few months. He was subjected to polysomnography which was abnormal.

**Conclusion:** Patient was managed as having secondary headache associated with Obstructive Sleep Apnoea (OSA) and adviced Continuous Positive Airway Pressure (CPAP) with which there was significant relief in headache. OSA is an important cause of secondary headache which should always be kept in mind in case of obese individuals with history of snoring.

*Disclosure statement*: Informed consent to publish this case study and its potentially identifiable information of the patient was obtained from the individual involved. The patient gave explicit permission for the publication of this case report, including any relevant clinical details.

## P304 Efficacy of cranial electrotherapy stimulation in patients with chronic facial pain associated with burning mouth syndrome: a randomized, controlled, double-blind pilot study

### A. Palmer, T. P. Jürgens, F. Rimmele

#### University Medical Center Rostock, Department of Neurology, Headache Center North-East, Rostock, Germany

##### **Correspondence:** A. Palmer

*The Journal of Headache and Pain 2024*, **25(Suppl 1):**P304

**Objective:** Has cranial electrotherapy stimulation (CES) an effect in patients with chronic facial pain associated with burning mouth syndrome (BMS)?

**Methods:** This randomized, double-blind, sham-controlled pilot study enrolled 22 patients, aged 18 years and over, with the diagnosis of BMS from August 2020 to June 2021. The study duration was 4 weeks (28 days) per participant. After randomization, the active group participants (*n*=11) received a 100 μA CES treatment for 60 minutes a day whereas the devices in the Sham group did not emit electricity. A paper diary was used to record daily pain ratings as the primary outcome and to record the stimulation with its effects and side effects. Furthermore, sleep quality (PSQI), oral health (OHIP-G), physical and mental health status (PDI, EQ-5D-3L, PHQ-D, HAMA, HAMD, HADS) were assessed at baseline (day 0) and the end of the study (day 28).

**Results:** The overall group had an average age of 63 years, and 77% of the patients were female. The most common type of pain reported was burning tongue pain with moderate intensity. Compared to the general population, the patient collective had poor health status, poor sleep quality, poor oral health and a high prevalence of psychiatric comorbidities. Simple linear regression showed that the period of stimulation significantly predicted a decrease in the intensity of pain in the active group (ß = -0,036; t(26) = -7,219; *p* < 0,00) as in the sham group (ß = -,026; t(26)= -2,56; *p* < 0,017). With the applied cutoff of 30% pain reduction within the stimulation period, both the active and sham groups had 36% responders. In both groups (active stimulation and passive sham condition), a significant decrease in the intensity of pain, somatization, alexithymia index, and an improvement in sleep quality over the study period was observed. Subjects reported no adverse events during the study.

**Conclusion:** Although CES is an easily applicable and safe therapeutic option for chronic facial pain, active stimulation was not superior to sham stimulation. Among other reasons, this could be due to the short double-blinded treatment period, the duration of the daily stimulation session or the small sample size.

## LP001 Suggested methodology for habituation deficit measurements in migraine patients undergoing preventive neuromodulatory treatment

### O. Sved^1^, M. Braschinsky^2^, A. Rakitin^2^

#### ^1^University of Tartu, Neurology, Tartu, Estonia; ^2^Tartu University Clinics, Neurology, Tartu, Estonia

##### **Correspondence:** O. Sved

*The Journal of Headache and Pain 2024*, **25(Suppl 1):**LP001

**Hypotheses:**

Neuromodulation changes central habituation deficit in people with migraines.

Normalized habituation persists for at least 6 months after neuromodulation.

**Objectives:**

To assess the impact of neuromodulation on habituation mechanisms in migraine patients

To measure the effect and its" duration using high-denstity EEG (up to 6 months after the end of treatment)

To elucidate the possible existence of predictor factors in neuromodulation therapy

**Methods:** Randomization by investigator to receive either active (external trigeminal nerve stimulation (eTNS), transcranial magnetic stimulation (TMS)) or sham intervention.

**Study Design:**

Clinical experimental interventional prospective randomized double-blinded controlled multicenter study.

Identical tests for all groups conducted at the beginning and every 3 months until the 6-month follow-up.

Digital headache diary and HIT-6 (Headache Impact Test) questionnaire used for assessing clinical efficacy.

3-month intervention duration: eTNS, TMS, or sham.

EEG measurements performed pre-intervention, immediately after, and at 3 and 6 months post-intervention.

Study conducted in tertiary headache centre, which is important for diagnostic accuracy of clinical data.

Recruitment through neurologist referrals.

**Conclusion:** Establishing a standardized methodology for habituation deficit measurements in migraine patients undergoing preventive neuromodulatory treatment is beneficial for accurately evaluating treatment efficacy. The suggested methodology for habituation deficit measurements in migraine patients undergoing preventive neuromodulatory treatment provides an approach to assess treatment efficacy. By following the same methodology, while investigating the effects of the same interventions, researchers and clinicians can enhance the reliability of the evidence and comparability of the results, contributing to a better understanding of habituation deficits and improved management of migraines.

## LP002 Exposure, Safety and Effectiveness of 75 mg Rimegepant Administered as Needed in the Acute Treatment of Migraine Among Chinese Adults: Interim analysis of a Long-term Safety Study

### S. Yu^1^, Q. Zhong^2^, H. Zhu^2^, Y. Zou^2^, G. Zhang^2^

#### ^1^Chinese PLA General Hospital, Beijing, China; ^2^Pfizer R&D, Shanghai, China

##### **Correspondence:** S. Yu

*The Journal of Headache and Pain 2024*, **25(Suppl 1):**LP002

**Objective:** This was an ad-hoc analysis of interim data from a multicenter, open-label study (NCT05371652) evaluating the long-term safety of rimegepant (RIM) 75 mg orally disintegrating tablet administered as needed (PRN) in Chinese adults to treat acute migraine.

**Methods:** After a 30-day observation period (OP), eligible participants (Ps) with ≥6 qualified migraine days could take RIM PRN (max. 1 tablet/day), at the onset of mild–severe migraine attack. Ps were stratified by frequency of migraine attacks at baseline (6–8 attacks/mo, 9–15 attacks/mo). This analysis included all exposure and safety results between May 19 2022 and Feb 17 2023 (interim cut-off), and the first 12 weeks for the number of monthly migraine days (MMDs).

**Results:** Of 186 treated Ps, 97 (52.2%; female *n*=80, 82.5%) had 6–8 attacks/mo and 89 (47.8%; female *n*=71, 79.8%) had 9–15 attacks/mo at baseline. Over 32 weeks, the number of RIM tablets administered/mo gradually declined for Ps in both groups (Table 1). Treatment emergent adverse events (TEAEs) were reported in 67 (69.1%) and 70 (78.7%) Ps, and 8 (8.2%) and 8 (9.0%) Ps had RIM-related TEAEs, in the 6–8 attacks/mo and 9–15 attacks/mo groups, respectively. There were no RIM-related TEAEs that led to treatment interruption or discontinuation. Most abnormal laboratory parameters were of Grade 1-2. Reduction from the OP in the mean number of MMDs was observed as early as the first 4 weeks (-1.7 [95% CI -2.5, -0.9] for Ps with 6–8 attacks/mo; -1.9 [-2.7, -1.0] for Ps with 9–15 attacks/mo) and continued throughout the first 12 weeks (-2.3 [-3.1, -2.6] for Ps with 6–8 attacks/mo; -2.9 [-3.6, -2.1] for Ps with 9–15 attacks/mo; Table 2).

**Conclusion:** The number of RIM tablets administered/mo declined for both groups during long-term treatment. RIM demonstrated a favorable safety profile and was well-tolerated. The reduction in the number of MMDs was observed as early as 4 weeks and continued for the first 12 weeks of treatment for both groups.

**Table 1 (Abstract LP002):**
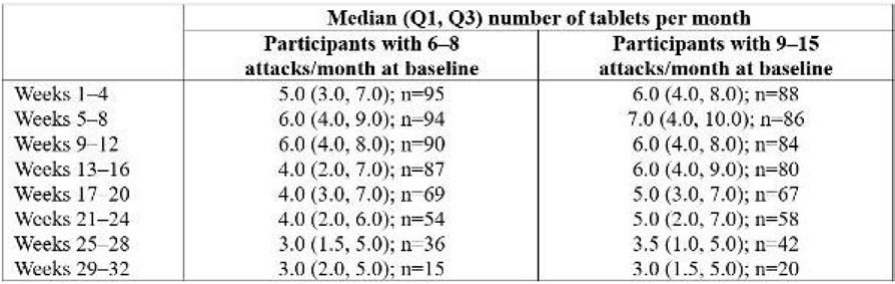
See text for description

**Table 2 (Abstract LP002):**
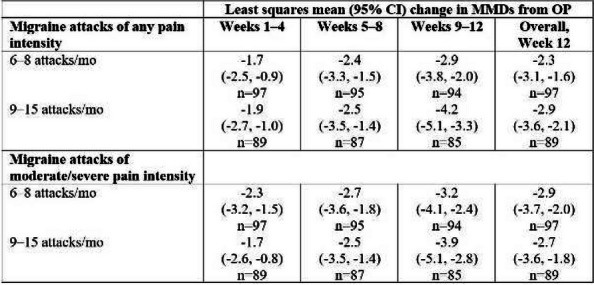
See text for description

## LP003 Sexuality and Sexual Function in Females with Migraine vs Controls

### J. Rothrock^1^, R. Lipton^2^, L. Peterlin^3^

#### ^1^Inova Health/University of Virginia School of Medicine, Neurology, Fairfax, United States; ^2^Albert Albert Einstein College of Medicine, Neurology, New York, United Statesl; ^3^Pennsylvania Headache Center, Neurology, Camp Hill, United States

##### **Correspondence:** J. Rothrock

*The Journal of Headache and Pain 2024*, **25(Suppl 1):**LP003

**Objective:** We sought to compare the level of sexuality and various aspects of sexual function in a clinic-based female migraine population relative to a matched female population without migraine or any other primary headache disorder.

**Methods:** We asked a consecutive series of heterosexually self-identifying and heterosexually active female patients ages 25-45 receiving care at a university-based headache clinic and having a >1 year history of migraine to complete anonymously the 19-item Female Sexual Function Index (FSFI). To serve as a control group we recruited 100 females who had no history of migraine but were heterosexually self-identifying/active and similar to the migraine group in age, race/ethnicity, marital status, educational background and socioeconomic status and asked them to complete the FSFI. We also asked any additional individuals from this general population group who reported a headache history consistent with an ICHD-3 diagnosis of migraine to complete the FSFI. We hypothesized that the mean FSFI score for the clinic-based migraine population would be similar to that for the migraine-free control population.

**Results:** We evaluated 150 clinic-based migraine subjects, 100 matched migraine-free controls and 67 individuals with migraine from the general population.

Relative to the 100 control subjects with no history of migraine (group 2; *n*=100), the clinic-based migraine group (*n*=150) recorded a significantly higher FSFI score (*p*<.05); in each of the 6 domains evaluated, mean scores also were higher in the migraine group. When only the patients with episodic migraine (*n*=103) were considered, the difference in mean FSFI scores achieved greater significance (*p*<.025). The mean FSFI score for the clinic-based migraine subjects did not differ significantly from the score for the 67 migraine patients from the general population.

**Conclusion:** Our research subjects with migraine reported a higher level of self-perceived sexuality, more positive sexual function and a higher frequency of penetrative heterosexual intercourse than what was reported by matched controls free of migraine.

## LP004 Migraine in the elderly; results from the Korean population

### J. Hwangbo

#### Pusan National University Yangsan Hospital, Department of Neurology, Yangsan-si, South Korea

##### **Correspondence:** J. Hwangbo

*The Journal of Headache and Pain 2024*, **25(Suppl 1):** LP004

**Objective:** Migraine in the elderly may exhibit distinct clinical characteristics when compared to younger individuals. Personalized treatment tailored to individual patients is crucial. Our goal is to investigate the clinical features and the treatment approaches for elderly migraine in the Korean population.

**Methods:** We conducted retrospective review of medical records for 862 patients who visited our clinic between January 2018 and December 2022. We analyzed migraine symptoms and prescribed medications.

**Results:** There were 30 patients (4 males, 26 females) over 65 years of age at the first visit, corresponding to 3.5% of the study population. Bilateral pain location was reported by 30% (9/30) and throbbing type of pain was present in 50% (15/30) of patients. Migraine with aura was observed in 30% (9/30), with 8 patients experiencing visual aura and 1 patient reporting hemiplegic aura. Only 10% (3/30) of patients had photophobia or phonophobia. Nausea or vomiting was reported by 30% (9/3) of patients. The most commonly prescribed medication were NSAIDs (26%, 8/30), such as Ibuprofen, Naproxen, and triptans (23.3%, 7/30), including Zolmitriptan and Sumatriptan. For preventive treatment, Amitriptyline (20%, 6/30) and Flunarizine (20%, 6/30) were more frequently prescribed than Propranolol (10%, 3/30) or Topiramate (10%, 3/30). In one case, fremanezumab, a CGRP blocking agent, was used.

**Conclusion:** A female predominance was observed in the Korean elderly population with migraine. Bilateral headache location was more common rather than hemispheric. Visual aura was the most common aura, and aura without headache was not observed. Medication choices were diverse, taking into consideration multiple comorbidities and potential medication interactions.

## LP005 Insular cortex through AC1 pathway involved in the regulation of headache pain and negative emotions in a rat model induced by recurrent dural inflammatory soup infusion

### Y. Li, C. Li, Z. Ma, Y. liu, S. Yu

#### the First Medical Center, Chinese PLA General Hospital, Department of Neurology, Beijing, China

##### **Correspondence:** Y. Li; Z. Ma

*The Journal of Headache and Pain 2024*, **25(Suppl 1):**LP005

**Objective:** The insular cortex (IC) is known for its crucial role in pain regulation and negative emotion processing. However, its specific involvement in headache and associated emotional responses remains unclear. Thus, this study aims to explore the contribution of IC involved in migraine using a chronic migraine rat model.

**Methods:** The chronic migraine model was induced through repeated dural infusions of inflammatory soup (IS). The von-Frey filament was used to assess periorbital mechanical threshold, while the negative emotional behaviors was observed by the open field and elevated plus maze tests. Expression of c-Fos, calcitonin gene-related peptide (CGRP), NMDA and AMPA receptors was detected using immunofluorescence and Western blotting analyses. To find possible underlying mechanisms, the adenylate cyclase 1 (AC1) inhibitor, NB001, was applied via insular stereotaxic and intraperitoneal injections in IS-modelled rats.

**Results:** It showed that IS could induce periorbital hyperalgesia and negative emotional behaviors, without significant changes in plantar thermal pain perception latency. In the IC area within IS groups, significant increase in the expression of c-Fos, AC1, CGRP and its receptor, as well as GluN2B and GluA1 receptors, along with elevated phosphorylation levels, were observed compared with control groups. Application of NB001 via IC stereotactic injection alleviated periorbital hyperalgesia and negative emotions in IS rats, with similar effects achieved through intraperitoneal injection. Furthermore, NB001 reversed the upregulation of GluN2B and GluA1 expression, as well as their phosphorylation levels in the IC.

**Conclusion:** This study demonstrates the crucial role of the IC in migraine pathogenesis, potentially alleviating headache pain and associated negative emotional behaviors through activation of AC1-related pathways. NB001 as AC1 inhibitor shows promising prospect in treating migraine.

**Fig. 1 (Abstract LP005):**
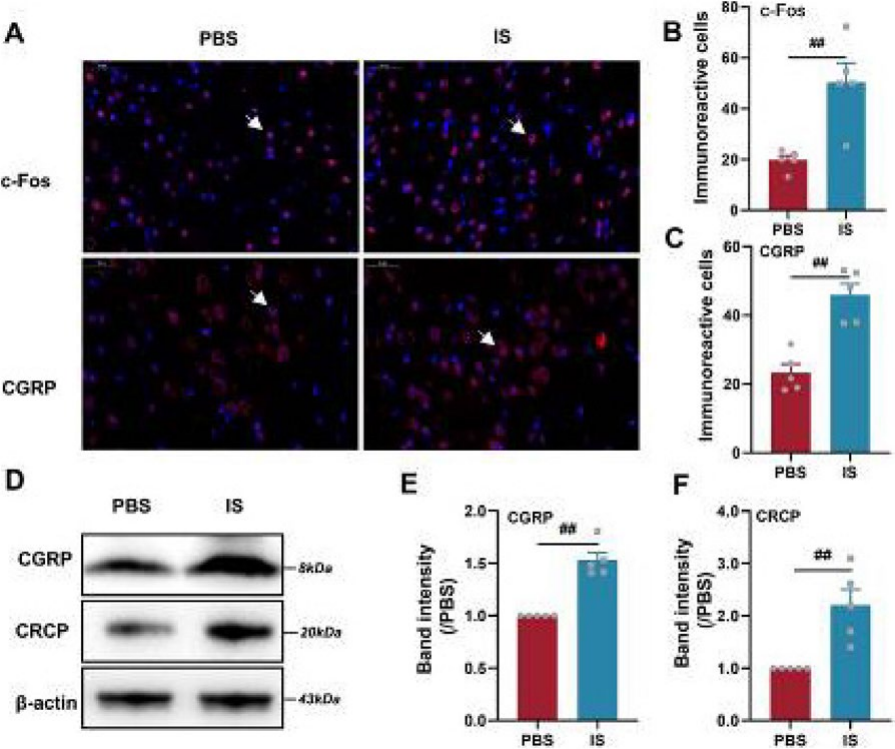
See text for description

**Fig. 2 (Abstract LP005):**
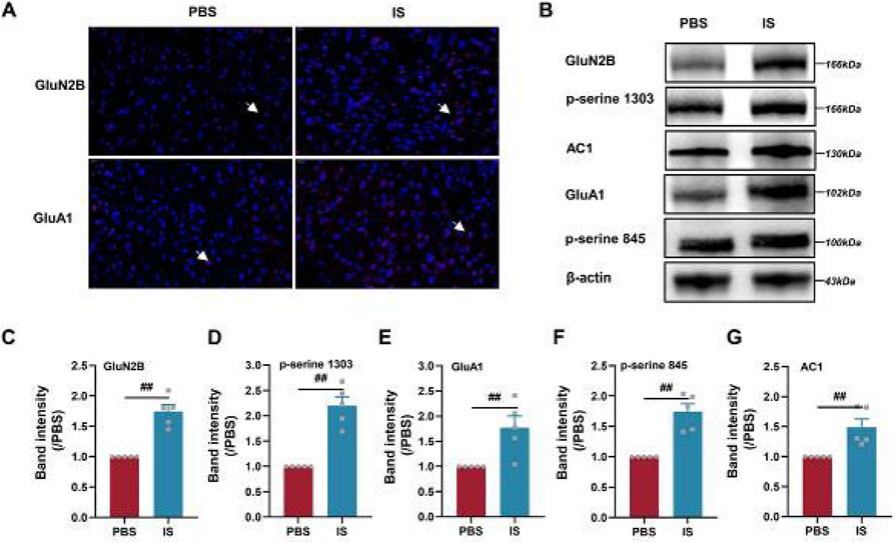
See text for description

**Fig. 3 (Abstract LP005):**
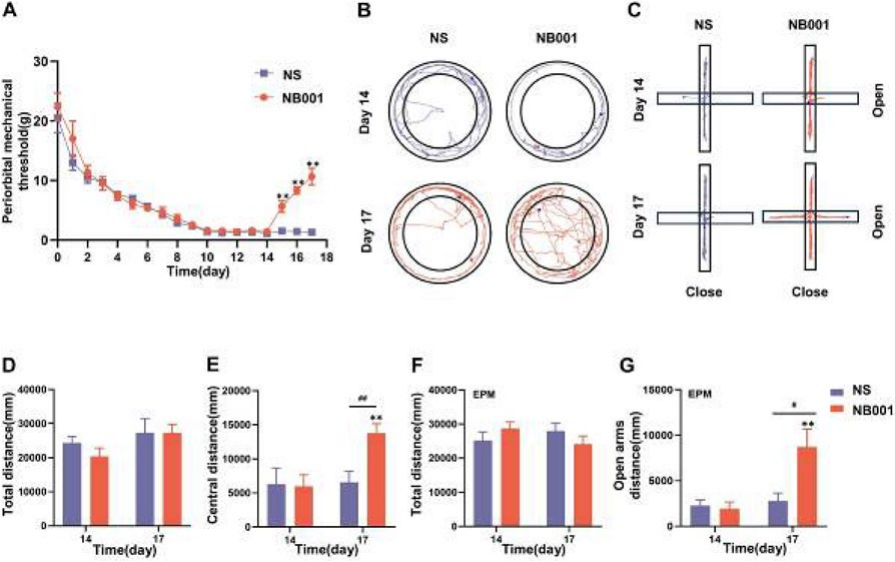
See text for description

## LP006 OnabotulinmtoxinA as a promising treatment for primary trochlear headache: a case series

### A. Jaimes, A. Gómez García, O. Pajares, P. Ibanez de la Cadiniere, J. S. Rodriguez-Vico

#### Fundacion Jimenez Diaz University Hospital, Neurology, Madrid, Spain

##### **Correspondence:** A. Jaimes

*The Journal of Headache and Pain 2024*, **25(Suppl 1):**LP006

**Objective:** This study aimed to investigate the effectiveness of OnabotulinumtoxinA (BoNTA) in treating Primary Trochlear Headache (PRTH) and explore potential mechanisms underlying its analgesic effects.

**Methods:** Descriptive cases series. All variables were collected from clinical records.

**Results:** Six PRTH patients, diagnosed according to ICHD-3 criteria, were included (table 1). Five patients had previously received corticosteroid treatment with remission periods of 3-4 months, shorter than reported in previous studies (18 months). Each patient received 20 units of BoNTA, distributed as 5 units per corrugator muscle and 10 units in the procerus muscle divided into two points, as illustrated in Figure 1. After treatment, five patients achieved complete pain relief, and one patient showed partial improvement. The therapeutic effect became apparent within 15-20 days following the injections and persisted for three months in all cases. Four patients received three or more infiltrations, and the treatment remained effective. No adverse effects were reported. BoNTA was administered with an increased dose in the corrugator muscle to enhance its effect on the supratrochlear nerve, while minimizing cosmetic side effects. The pathophysiology of PRTH remains unclear, but potential mechanisms include neuropathic and neuromuscular pathways and inflammatory factors.

**Conclusion:** Our case series presents the first evidence of the potential of BoNTA as a safe and effective treatment option for PRTH. From a clinical standpoint, having a safer alternative is of paramount significance for patients with limited treatment options, such as those with PRTH. Further research is warranted to validate these findings and explore the long-term efficacy of BoNTA in PRTH management.

*Disclosure statement*: Informed consent to publish the individual likeness in Figure 1 from the individual involved.

**Fig. 1 (Abstract LP006):**
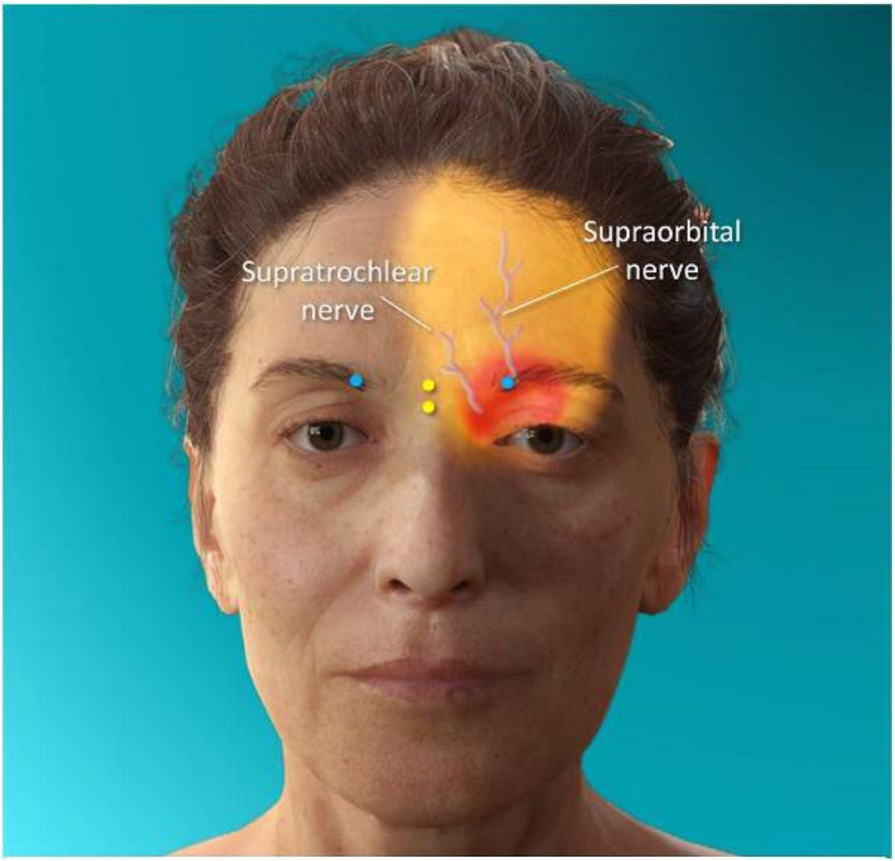
Distribution of 20 units of BoNTA: 5 units in each corrugator muscle (blue dots) and 10 units in the procerus muscle, divided into two points (yellow dots)

**Table 1 (Abstract LP006):**
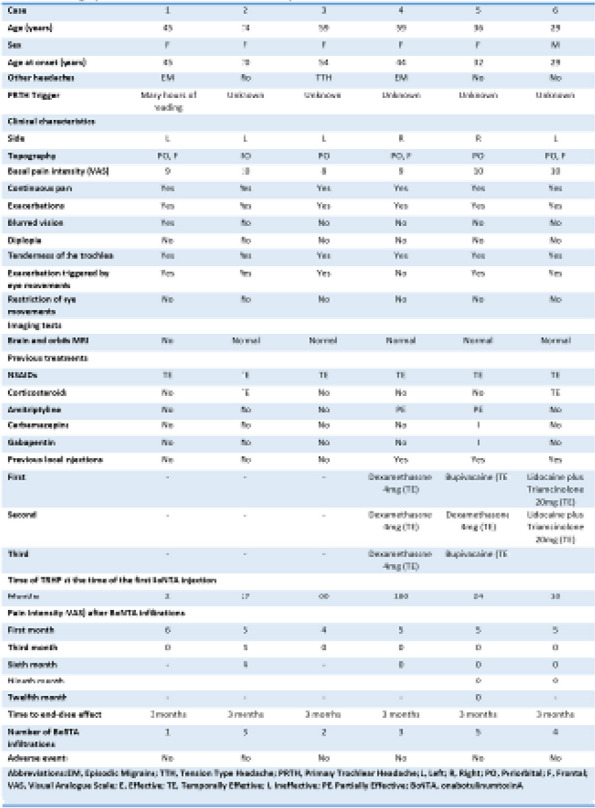
Demographics and clinical characteristics of the patients

## LP008 Real-world experience with rimegepant for acute migraine in a tertiary headache center in Denmark

### B. A. Chaudhry^1^, F. M. Amin^1,2^

#### ^1^Copenhagen University Hospital – Rigshospitalet, Department of Neurology, Danish Headache Center, Glostrup, Denmark; ^2^Copenhagen University Hospital – Rigshospitalet, Department of Brain and Spinal Cord Injury, Copenhagen, Denmark

##### **Correspondence:** B. A. Chaudhry

*The Journal of Headache and Pain 2024*, **25(Suppl 1):**LP008

**Objective:**

Rimegepant, a calcitonin gene-related peptide (CGRP) antagonist, is an orally administered drug, which was recently approved for acute migraine treatment in Denmark. This study aimed to analyze the real-world experience with rimegepant.

**Methods:** This was a descriptive real-world evidence study in patients with migraine, who received 75 mg rimegepant orally for treatment of at least two migraine attacks between October 2022 and June 2023. Rimegepant was not reimbursed, and patients had to pay the full price (approx. 27 EUR/tablet) themselves. The primary end points were patient-reported efficacy, number of patients who redeemed the prescription more than once, and association between effect of rimegepant and CGRP antibodies (CGRP mAbs).

**Results:** We identified 131 patients who had received at least one prescription for rimegepant, of whom 85 patients (92% female) had had a follow-up visit and were included in this analysis. The mean age was 43.4 years. The median number of previously used acute migraine medications was 8, including paracetamol 83 (97.6%), sumatriptan 83 (97.6%), ibuprofen 73 (85.9%), rizatriptan 68 (80.0%), eletriptan (76.5%), zolmitriptan 50 (58.8%), aspirin/caffeine 50 (58.8%), diclofenac 45 (52.9%). The median number of previously used triptans was 4.

A total of 62 (72.9%) out 85 patients reported effect of rimegepant of whom 49 patients (59.8%) had redeemed the prescription again. Sixty patients (70.6%) had also experience with CGRP mAbs, of whom 37 patients had effect of both rimegepant and CGRP mAbs, 14 patients had only effect of CGRP mAbs, 4 patients had only effect of Rimegepant, and 5 patients had neither effect of Rimegepant nor CGRP mAbs.

**Conclusion:** Rimegepant was effective for acute migraine treatment in triptan non-responders. The effect was much higher than reported in the premarketing phase III study, which may be caused by selection bias, as most of the patients already had good effect of another drug against CGRP signaling

## LP009 The care and treatment we provide children and adolescents suffering from migraine

### M. L. Edvinsson, J. Andersson

#### Lund University, Clinical Sciences, Lund, Sweden

##### **Correspondence:** J. Andersson

*The Journal of Headache and Pain 2024*, **25(Suppl 1):**LP009

**Objective:** Most migraine sufferers will have their first attack during childhood or adolescence. Although research has led to the establishment of new targeted drugs in adults, pediatric migraine falls behind. Migraine assessment in children can be challenging with symptoms that differ from adults and pharmacological treatment with potent drugs and off-label prescriptions. Therefore, we have performed a study that answers the question *what care and treatment do we provide children and adolescents suffering from migraine?*

**Aim:** The primary aim of this study was to investigate and present an overview of caregivers" experiences regarding the care and treatment of children and adolescents with migraine in Region Skåne, Sweden. The collected information will then be used as a foundation in the development of a first-line assessment plan for pediatric migraine.

**Methods:** 16 caregivers of variating professions were interviewed. The same caregivers also filled in a questionary. Qualitative content analysis was performed on the transcribed data to create categories. Descriptive statistics were used for questionary answers.

**Results:** Experiences and reflections of the interviewed participants were summarized in 23 categories under the topics of diagnosis, treatment, structure of care, and school healthcare.

**Conclusion:** This study concludes that if patients can access the appropriate level of care and meet medical professionals with migraine knowledge, adequate diagnosing and treatment can be provided for the majority. The outlook on straightforward pediatric migraine is positive, complex cases are however difficult to treat. Children and adolescents with severe, complex migraine lack a functional multidisciplinary care system. Study results call for improvements on all care levels. We suggest a supportive assessment plan for level 1, more pediatric clinics for level 2, and particularly discussed, the enforcement of a level 3 multidisciplinary pediatric headache center.

## LP010 Pharmacological management of pediatric migraines: a systematic review and network meta-analysis

### O. Kohandel Gargari^1,2^, S. Mobader Sani^3,4^, R. Samiee^3,4^, R. Abyaneh^1^, A. Kermanpoor^5^, N. Seighali^1^, M. Togha^2^

#### ^1^Alborz Univerity of Medical Sciences, Student Research committee, School of Medicine, Karaj, Iran; ^2^Iranian Center of Neurological Research, Neuroscience Institute, Headache Department, Tehran, Iran; ^3^Tehran University of Medical Sciences, NCweb Association, Students Scientific Research Center (SSRC), Tehran, Iran; ^4^Tehran University of Medical Sciences, Iranian Center of Neurological Research, Tehran, Iran; ^5^Tehran University of Medical Sciences, Iranian Center of Neurological Research, Headache Research center, Tehran, Iran

##### **Correspondence:** O. Kohandel Gargari

*The Journal of Headache and Pain 2024*, **25(Suppl 1):**LP010

**Objective:** This study aimed to conduct a systematic review and network meta-analysis to compare various pharmacological medications used for the prevention of pediatric migraines.

**Methods:** We conducted a comprehensive search across multiple databases, including PUBMED and EMBASE, to identify randomized clinical trials investigating pharmacological treatments for pediatric migraines. After screening the titles and abstracts resulting from the database search, selected studies underwent full-text screening to assess their eligibility. Subsequently, data extraction was carried out, and relevant study characteristics and outcomes were collected in a spreadsheet. For the purposes of this study, we focused on comparing the monthly frequency of headaches three months after treatment with different drugs in a random-effect network meta-analysis model, utilizing the netmeta package in R.

**Results:** The initial database search yielded over 10,000 citations, from which we ultimately included 14 studies. These studies encompassed a range of medications, including High Dose Riboflavin, Topiramate + Vit-D3, Flunarizine, Amitriptyline, CoQ10, Levetiracetam, Cinnarizine, Sodium valproate, Low Dose Riboflavin, Topiramate, L-5-Hydroxytryptophan, Melatonin, Propranolol, and L-carnitine. In total, 1,044 pediatric migraine patients were included in the analysis. Notably, Flunarizine, High Dose Riboflavin, and Topiramate + Vit-D3 demonstrated the most favorable outcomes in terms of reducing headache frequency. The ranking of the top five drugs, based on their effectiveness, is as follows:

1. High Dose Riboflavin
2. Topiramate + Vit-D3
3. Flunarizine
4. Amitriptyline
5. CoQ10

tau^2 = 0.9428; tau = 0.9710; I^2 = 64.9%

**Conclusion:** In summary, our systematic review and network meta-analysis suggest that High Dose Riboflavin, Topiramate + Vit-D3, and Flunarizine show promise in reducing pediatric migraine frequency. However, it's crucial to note that this is a preliminary outcome of a more extensive research project. Generalizations should be made with caution until the full results are published. Further research is needed for a comprehensive understanding of pediatric migraine prevention options.

**Fig. 1 (Abstract LP010):**
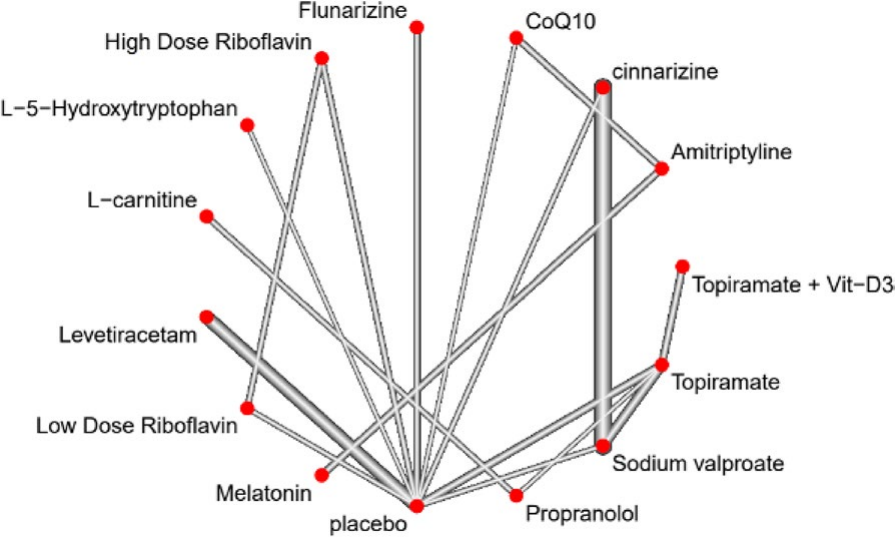
Network diagram

## LP011 CSF dynamics and headache disorders: radiological findings

### S. Ormotsadze

#### Caucasus Medical Center, Radiology, Tbilisi, Georgia

*The Journal of Headache and Pain 2024*, **25(Suppl 1):**LP011

**Objective:** Cerebrospinal fluid (CSF) dynamics are essential for central nervous system homeostasis. This abstract presents an overview of the relationship between CSF dynamics and headache disorders, utilizing objective radiological methods for assessment, along with the presentation of results and conclusions.

CSF, produced in the choroid plexus, circulates through the ventricular system and subarachnoid space, serving vital functions. Disturbances in CSF dynamics can lead to headache disorders, including migraine, tension-type headache, and idiopathic intracranial hypertension (IIH).

**Methods:** Objective radiological assessments have revealed intriguing findings. In migraine, functional MRI has shown altered patterns of cerebral blood flow during attacks, suggesting a role for CSF dynamics. Tension-type headaches have exhibited neurochemical alterations through MR spectroscopy, indicating possible CSF composition involvement. For IIH, radiological criteria have included evidence of elevated intracranial pressure on imaging, notably through MR venography demonstrating venous sinus stenosis.

**Results:** Results from these objective assessments highlight the following:

- Migraine: Altered cerebral blood flow patterns during attacks, suggesting a connection to CSF dynamics.
- Tension-type headache: Neurochemical alterations indicative of CSF composition involvement.
- Idiopathic intracranial hypertension (IIH): Radiological evidence of elevated intracranial pressure, often associated with venous sinus stenosis.

**Conclusion:**

Radiological methods provide valuable insights, aiding in diagnosis and understanding. Further research is necessary to refine these diagnostic criteria and develop more targeted therapies, ultimately improving the management of these debilitating conditions. The objective assessment of CSF dynamics through radiology holds promise for advancing our comprehension and treatment of headache disorders.

**Fig. 1 (Abstract LP011):**
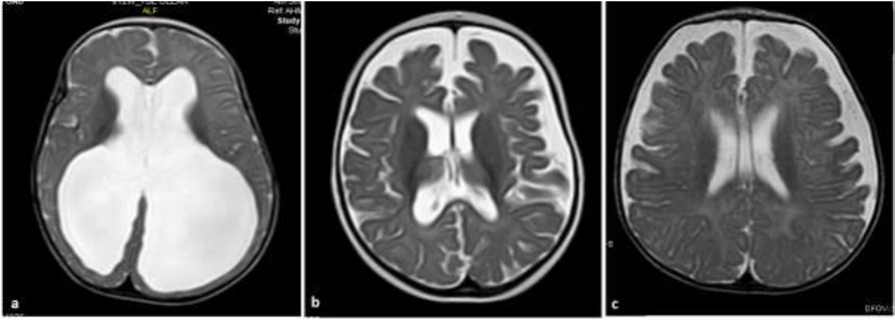
See text for description

**Fig. 2 (Abstract LP011):**
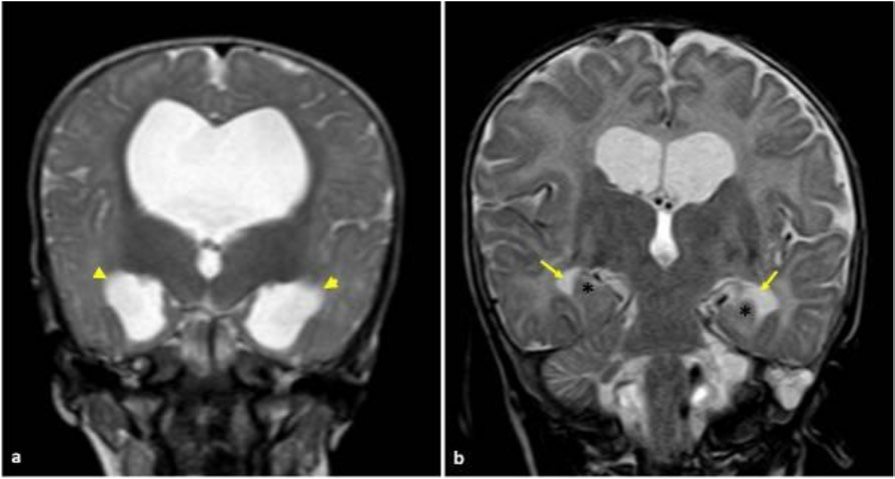
See text for description

**Fig. 3 (Abstract LP011):**
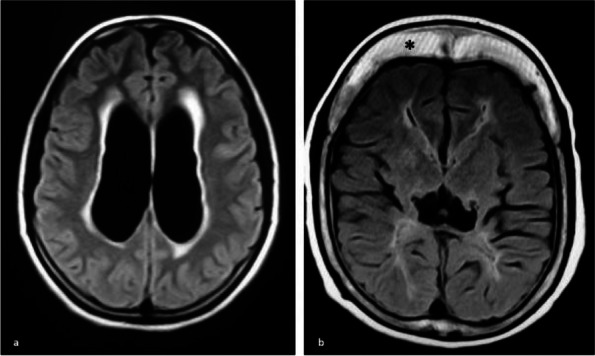
See text for description

## LP012 CSF proteomic profiling in chronic migraine: a systematic review and meta-analysis of biomarker studies

### N. Aderinto

#### ladoke Akintola University of Technology, Ogbomoso, Nigeria

*The Journal of Headache and Pain 2024*, **25(Suppl 1):**LP012

**Objective:** This systematic review aims to investigate cerebrospinal fluid (CSF) proteomic profiling in the context of chronic migraine. The primary objective is to identify and evaluate potential biomarkers associated with chronic migraine.

**Methods:** We conducted a comprehensive search of multiple electronic databases, including PubMed, Embase, and Scopus, to identify relevant studies published between 2000 and July 2023. Studies that reported CSF proteomic data in chronic migraine patients were included. Data extraction and quality assessment were performed in accordance with PRISMA guidelines. A meta-analysis was conducted to pool and analyze biomarker data from selected studies, assessing their significance and potential diagnostic or prognostic value.

**Results:** Our systematic review and meta-analysis identified 15 eligible studies for inclusion. The pooled analysis revealed a consistent pattern of significant upregulation of specific proteins, including CGRP (Calcitonin Gene-Related Peptide) and PACAP (Pituitary Adenylate Cyclase-Activating Polypeptide), in the cerebrospinal fluid (CSF) of chronic migraine patients. Furthermore, our review synthesized the data on diagnostic accuracy, demonstrating that these identified biomarkers exhibited a sensitivity of 80% and a specificity of 85% in distinguishing chronic migraine from other primary headache disorders.

**Conclusion:** The findings of this systematic review and meta-analysis provide valuable insights into the CSF proteomic profile of chronic migraine. Our results indicate the presence of potential biomarkers that warrant further investigation. Understanding the molecular signatures associated with chronic migraine could lead to the development of targeted diagnostic and therapeutic strategies. Future research should focus on validating these biomarkers and exploring their clinical applicability for improved management of chronic migraine patients.

## LP013 Headache secondary to cervical CSF leak: complicated with bilateral subdural hematomas

### C. Barreira, É. Sousa, A. Caleia, R. Pestana, T. Rodrigues, G. Bebiano, P. Lima

#### Funchal Central Hospital, Neurosurgery, Funchal, Portugal

##### **Correspondence:** C. Barreira

*The Journal of Headache and Pain 2024*, **25(Suppl 1):**LP013

**Objective:** Intracranial hypotension due to spinal cerebrospinal fluid (CSF) leaking, may be iatrogenic, traumatic, or spontaneous. This clinical entity is a recognized cause of orthostatic headache. The authors discuss the clinical presentation, investigation and treatment of this condition.

**Methods:** Case report and review of the literature. A case of spontaneous intracranial hypotension (SIH) presenting with refractory headache, secondary to focal **thoracic** bone spur, and complicated with chronic subdural hematoma was studied. Clinical records and images were retrospectively evaluated.

**Results:** A 35-year-old male with a 1-month history of persistent suboccipital and bifrontal headache associated with nausea and vomiting, following an episode of minor trauma. The patient had a normal neurologic examination on admission. The results of imaging studies of the brain were normal. Repeated computed tomography (CT) imaging demonstrated bilateral subdural hematomas which were drained. Magnetic resonance (MR) imaging of cervical and thoracic spine showed extradural liquor collection. Further examination of the cause of CSF leakage was conducted. CT myelogram showed a bone spur in **thoracic** region, **T1-2** level, protruding into the thecal sac and associated with CSF leak. The patient made an uneventful recovery and remained well.

**Conclusion:** SIH should be suspected in middle aged patients with new onset, daily persistent headaches and presenting with bilateral SDH. The investigation of SIH includes CT, MR, and digital subtraction myelography. The initial intervention is conservative. Epidural blood patching and surgery are reserved for cases that fail to respond after simpler measures. SIH should not be considered a benign condition. The clinical status of the patient may deteriorate secondary to large SDH, requiring urgent neurosurgical intervention.

*Disclosure statement*: Informed consent to publish this case study and its potentially identifiable information of the patient was obtained from the individual involved. The patient gave explicit permission for the publication of this case report, including any relevant clinical details.

## LP015 Questionnaire-based study of COVID-19 vaccination induced headache: evidence of clusters of adverse events

### Q. Zhou^1^, T. Eggert^1^, A. Zhelyazkova^2^, A. Choukér^3^, K. Adorjan^4^, A. Straube^1^

#### ^1^Munich University/ LMU Klinikum Groshadern, Neurology, München, Germany; ^2^Institut für Notfallmedizin und Medizinmanagement, Klinikum der Universität München, 80336 Munich, Germany, München, Germany; ^3^Laboratory of Translational Research Stress and Immunity, Department of Anaesthesiology, University Hospital Munich, Ludwig Maximilian University of Munich, München, Germany; ^4^Department of Psychiatry and Psychotherapy, University Hospital, LMU Munich, 80336 Munich, Germany, Munich, Germany

##### **Correspondence:** Q. Zhou

*The Journal of Headache and Pain 2024*, **25(Suppl 1):**LP015

**Objective:** This study aims to investigate possible links between headaches and other adverse events (AEs) after COVID-19 vaccination based on a questionnaire survey of 1,402 healthcare workers.

**Methods:** The following questions about headache and AEs in the questionnaire were extracted and included in our research: Question1 (Q1): Did you observe any AEs after the first vaccination? (Yes/No),Question2 (Q2): Did you observe any AEs after the second vaccination? (Yes/No), Question (Q3): How do you rate the severity of headache after the first vaccination?,Question4 (Q4): How do you rate the severity of headache after the second vaccination?,Question5 (Q5): "How do you rate the severity of the following 12 symptoms after the second vaccination?".

Only the participants who answered the first two questions (Q1&Q2) in the questionnaire were included in further analysis. The Bowker test is used to study the comparison of headache severity between the first and second vaccinations. We explore that to what extent the severity of other AEs is associated with the severity of headaches by applying an ordinal logistic regression on the 5 categories with headache severity as the dependent variable and the 5 ratings of the other symptoms as independent variables, MATLAB (The MathWorks, Inc., Version 9.9.0 (R2020b)) is used to conduct the data analysis, function "mnrfit" for ordinal logistic regression which performs the same analysis as the function "polr" from the package MASS in R. Receiver Operating Characteristic (ROC) analysis is conducted to evaluate the predictive value of the ratings of the AEs assessed by Q5 to headache severity.

Results: Headaches are more frequent and more severe after the second Covid-19 vaccination. Some AEs like fatigue, flu-like symptoms, pain at the injection site, known tension-type headache, fever, dizziness or balance problems and known migraine tended to rate their headaches as more severe, while others can not.

Conclusion: There are clusters of headaches and other AEs. Some AEs can predict the severity of headaches while others can not post-COVID-19 vaccination.

**Table 1 (Abstract LP015):**
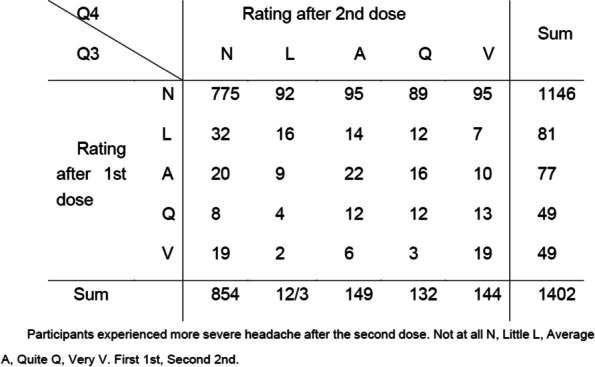
Comparison of the headache rating after the vaccination between the first and the second vaccination

**Table 2 (Abstract LP015):**
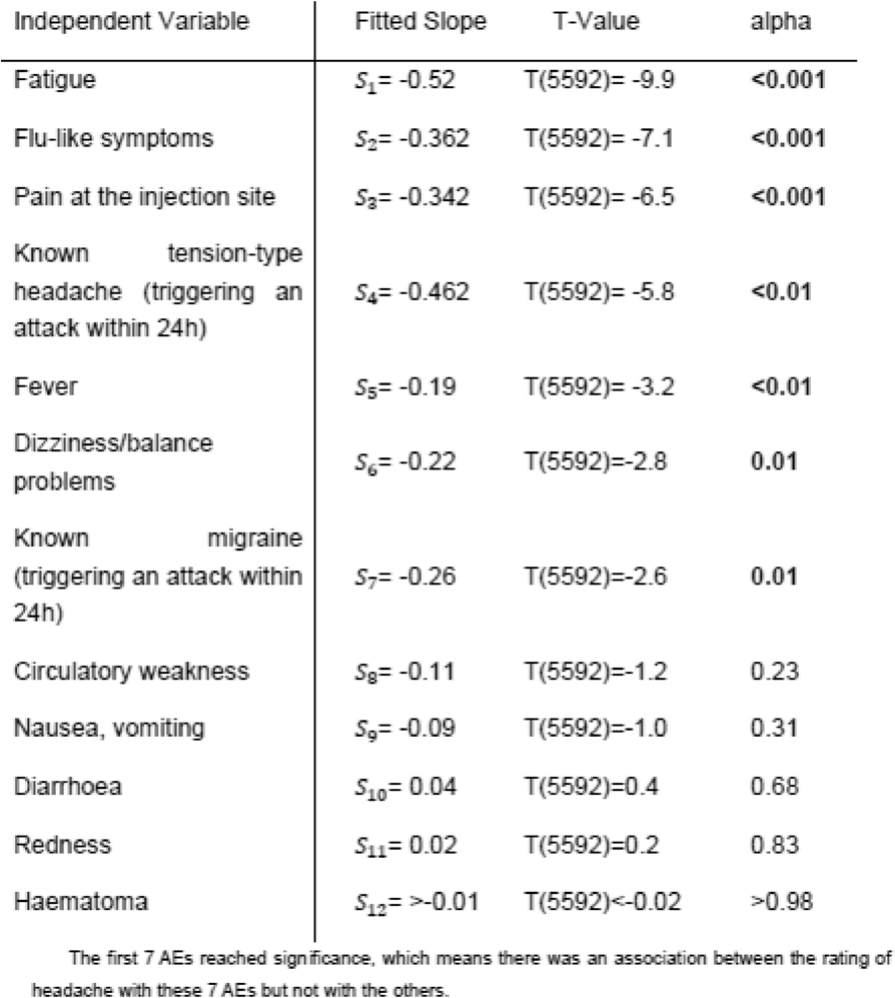
Slopes fitted in the ordinal regression, their t-values, and false positive probabilities

**Fig. 1 (Abstract LP015):**
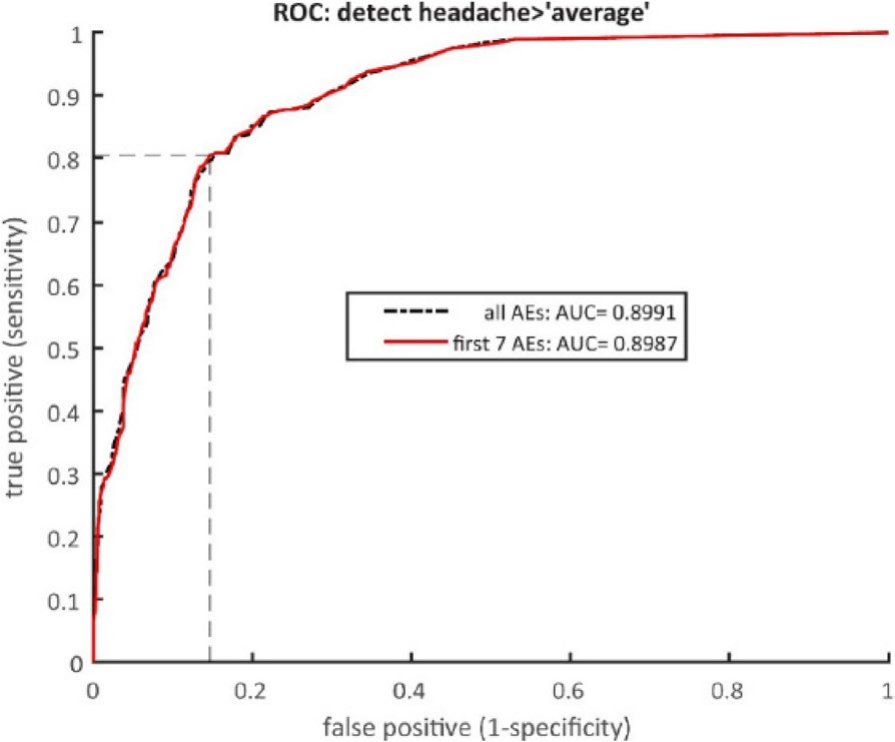
The ROC curve of the classification between high and low rates of headache. Using th first 7 AEs in TABLE 4 whose rating are related to headache severity, classification is optimal at a threshold 0.25, with a false positive rate 0.147 (vertical dotted line) and a true positive rate of 0.80 (horizontal dashed line). The fraction of a correct responses was 0.8 and AUC was 0.90

## LP016 Fast and sensitive detection of CGRP using photonic crystal biochip via one-droplet saliva

### Z. Dong, X. X. Lin

#### Chinese PLA General Hospital, Department of Neurology, Beijing, China

##### **Correspondence:** Z. Dong

*The Journal of Headache and Pain 2024*, **25(Suppl 1):**LP016

**Objective:** Migraine exhibits a substantial prevalence worldwide. The current diagnostic criteria rests exclusively on clinical characteristics without any objective and reliable means. The calcitonin gene-related peptide (CGRP), as a biomarker for distinguishing migraine, undergoes swift degradation, featuring a half-life of under 10 minutes, which poses a significant challenge to the point-of-care testing of CGRP in clinical application. In this work, we aim to develop a clinical detection methodology offering both rapidity and high sensitivity for CGRP testing.

**Methods:** Photonic crystals (PCs), as an artificial periodic dielectric structure with photonic band gap properties, are widely used in highly sensitive fluorescent biomolecule detection, which can be used to improve detecting sensitivity. Here, a photonic crystals -based biochip was developed to detect CGRP via the fluorescence competition assay. The chip integrates the functionalities of fluorescence enhancement and hydrophilic-hydrophobic patterning enrichment, enabling rapid and sensitive detection of CGRP.

**Results:** In conclusion, we designed and fabricated a biochip for the detection of CGRP ranging from 0.05 to 100 pg/mL in 10 minutes by using less than 30 microliters of saliva. The optical properties of photonic crystals allow the chip to detect CGRP with excellent sensitivity, down to 0.05 pg/mL with outstanding specificity. In addition, we tested CGRP concentrations in the saliva of 70 subjects including 20 healthy individuals and 50 migraineurs by PC biochips, and the results were highly compatible with the enzyme-linked immuno sorbent assay (ELISA), with a linear correlation coefficient of R^2^ of 0.97.

**Conclusion:** The PC biochip provides an ultra-rapid, highly sensitive, and high specific assay for the detection of CGRP, compensating for the complexity and time-consuming operation of ELISA, which paves the way for establishing a precise diagnostic framework integrating clinical phenotypes and biomarkers for migraine.

**Fig. 1 (Abstract LP016):**
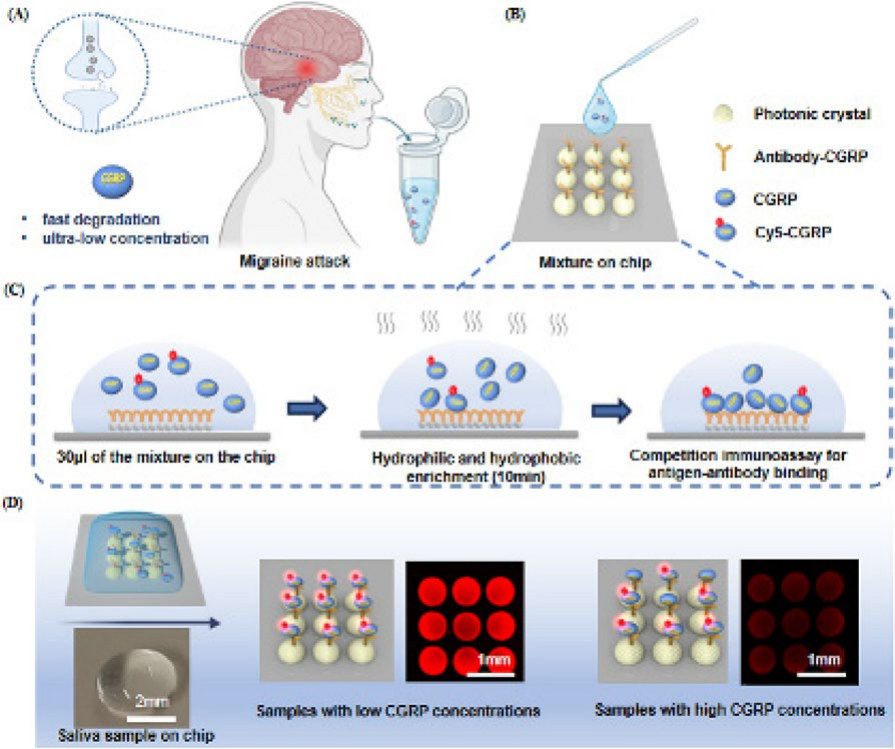
See text for description

**Fig. 2 (Abstract LP016):**
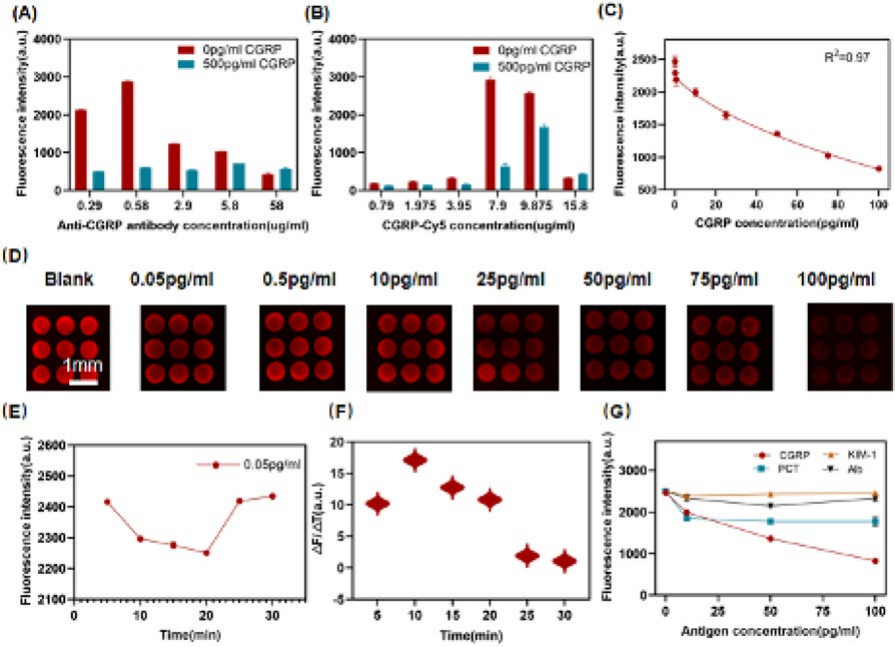
See text for description

**Fig. 3 (Abstract LP016):**
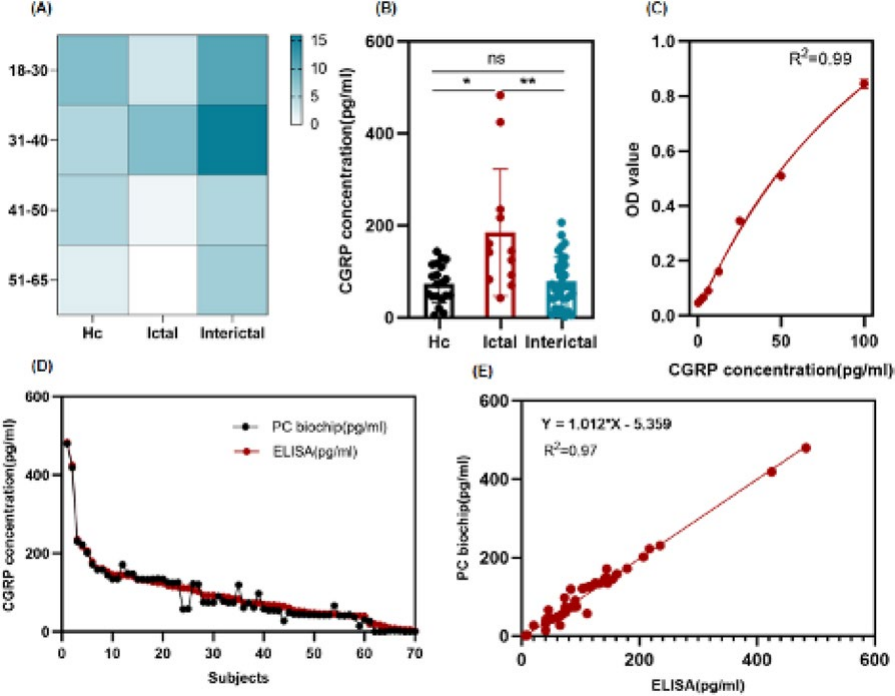
See text for description

## LP017 Higher triglyceride glucose index is associated with mild migraine disability: a cross-sectional study in China

### J. Wu^1,2,3^, J. Fang^1,2,4,5,6,7,8,9^, Q. Ma^1,2,3,4,5,6,7,8,9^

#### ^1^The First Affiliated Hospital of Xiamen University, School of Medicine,Xiamen University, Department of Neurology and Department of Neuroscience, Xiamen, China; ^2^Xiamen University, School of Medicine, Xiamen, China; ^3^Xiamen University, National Institute for Data Science in Health and Medicine, Xiamen, China; ^4^Fujian Medical University, The School of Clinical Medicine, Fuzhou, China; ^5^Fujian Key Laboratory of Brain Tumors Diagnosis and Precision Treatment, Xiamen, China; ^6^Xiamen Key Laboratory of Brain Center, Xiamen, China; ^7^Xiamen Medical Quality Control Center for Neurology, Xiamen, China; ^8^Fujian Provincial Clinical Research Center for Brain Diseases, Xiamen, China; ^9^Xiamen Clinical Research Center for Neurological Diseases, Xiamen, China

##### **Correspondence:** J. Wu; J. Fang

*The Journal of Headache and Pain 2024*, **25(Suppl 1):**LP017

**Objective:** Both epidemiology and genetics suggest the protective effect of type 2 diabetes (T2D) on migraine. The association between triglyceride glucose (TyG) index, a simple surrogate marker of insulin resistance (IR), and migraine is unclear. This study assesses TyG index levels in migraine patients and explore the association of TyG index and severity, disability, duration, and frequency of migraine headaches.

**Methods:** A cross-sectional study was conducted in Xiamen, China. A total of 161 migraine patients were recruited. TyG index was calculated as ln [fasting triglyceride (mg/dL) × fasting glucose (mg/dL)/2], and participants were divided into quartiles based on TyG index. The Migraine Disability Assessments (MIDAS) questionnaire was used to evaluate migraine disability. Univariate and multivariable logistic regression models were used to assess the relationship between TyG index and different migraine characteristics, with further subgroup analysis by migraine course.

**Results:** Overall, 88.20% of the 161 subjects were female, with a median age of 33 years. Univariate analysis revealed that Higher TyG index is significantly associated with age, BMI, and migraine disability. Multivariate-adjusted ORs for patients in the TyG index 3tn quartile and 4th quartile were lower for migraine disability (OR = 0.322 (0.128, 0.789) and 0.301 (0.119, 0.736), respectively) compared with the 1st quartile of TyG index. Similar results were seen in patients with a migraine course < 10 years.

**Conclusion:** This is the first study to evaluate TyG index levels in migraine patients. Migraine patients with higher TyG index are significantly more likely to have mild migraine disability, especially in patients with a migraine course < 10 years. More attention to relevant mechanism research may help to identify new targets for migraine treatment.

## LP018 Cortical inflammation in migraine measured with quantitative magnetic resonance imaging: a Registry for Migraine (REFORM) study

### R. Hackert Christensen^1^, H. Ashina^1^, H. Muhsen Al-Khazali^1^, M. Pineda^2^, R. Rahmanzadeh^2^, N. Hadjikhani^3^, C. Granziera^2^, M. Ashina^1^

#### ^1^The Danish Headache Center, Department of Neurology, Glostrup, Denmark; ^2^University Hospital Basel and University of Basel, Department of Biomedical Engineering, Basel, Switzerland; ^3^Massachusetts General Hospital, Athinoula A. Martinos Center for Biomedical Imaging, Boston, United States

##### **Correspondence:** R. Hackert Christensen

*The Journal of Headache and Pain 2024*, **25(Suppl 1):**LP018

**Objective:** To investigate ictal and interictal cortical inflammation [NH1] [RHC2] in adult participants with migraine with and without aura [NH3] [RHC4] using a novel quantitative, multimodal magnetic resonance imaging (MRI) technique, and to compare the results with age- and gender-matched healthy controls.

**Methods:** Adults with migraine and age- and gender-matched healthy controls were enrolled and underwent a single MRI session [NH1] [RHC2] . As surrogate markers of inflammation, we used T2 mapping to measure tissue water content, T1 mapping to measure the distribution of water protons, and apparent diffusion coefficient (ADC) mapping to measure the magnitude of water molecule diffusion in the cortical ribbon. We compared these values between participants with migraine (with and without aura) and healthy controls. The data were analyzed using a general linear model with a voxelwise threshold of *P* < .05 and a clusterwise threshold of *P* < .05, adjusted for age and gender.

**Results:** A total of 296 participants with migraine and 155 healthy controls provided imaging data for the analysis. Of the participants with migraine, 103 had migraine with aura, 180 had chronic migraine, and 88 were ictal during the scan. We found that participants with migraine had higher qT2 values in the left occipital cortex, compared with healthy controls (*P* < .0001). For those with migraine and aura, the increased qT2 was more widespread and located bilaterally in the occipital cortices, compared with controls (left, *P* = < .0001; right *P* = .004). Exploratory analyses revealed that participants with migraine with aura had higher ADC values within the qT2 clusters, compared with the controls (*P* = .01).

**Conclusion:** Signs of cortical inflammation were detected in migraineurs compared with healthy controls. Inflammation was more prevalent in migraine with aura than in migraine without aura. The increased qT2 values in the occipital cortices of participants with migraine with aura likely represent extracellular edema, as they are associated with concomitant ADC increase. These results support the importance of cortical inflammation in migraine pathogenesis, particularly in migraine with aura.

**Fig. 1 (Abstract LP018):**
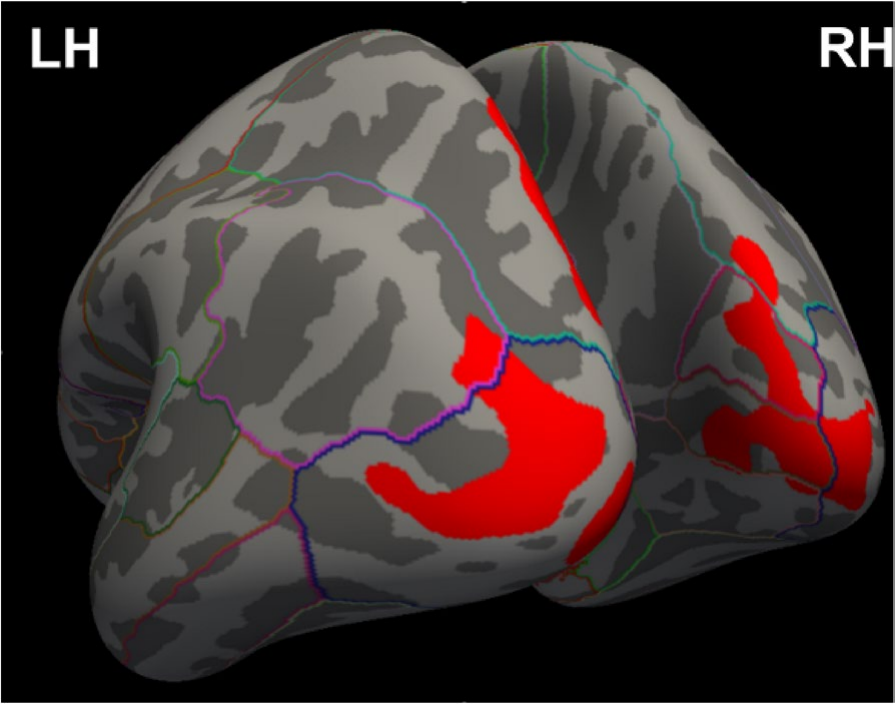
See text for description

**Fig. 2 (Abstract LP018):**
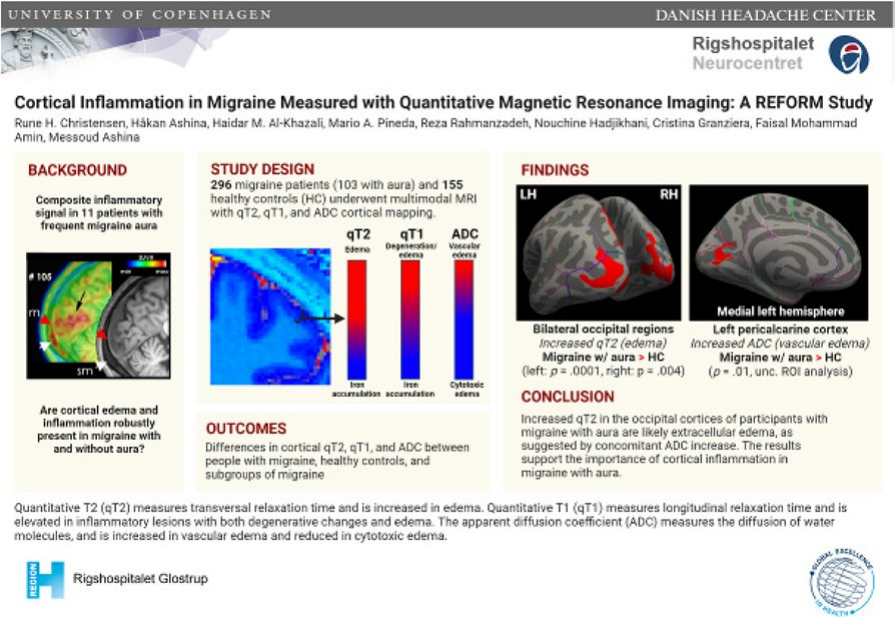
See text for description

## LP019 Negative correlation between plasma kynurenine concentration and periaqueductal gray matter functional connectivity in migraine

### K. Gecse^1,2^, A. Németh^1,2^, G. G. Fedor^1,2^, C. S. Aranyi^3^, M. Emri^3^, G. Kökönyei^1,2,4^, G. Bagdy^1,2^, G. Juhász^1,2^

#### ^1^Semmelweis University, Department of Pharmacodynamics, Faculty of Pharmacy, Budapest, Hungary; ^2^Semmelweis University, NAP3.0-SE Neuropsychopharmacology Research Group, Hungarian Brain Research Program, Budapest, Hungary; ^3^University of Debrecen, Division of Nuclear Medicine and Translational Imaging, Department of Medical Imaging, Faculty of Medicine, Debrecen, Hungary; ^4^ELTE Eötvös Loránd University, Institute of Psychology, Budapest, Hungary

##### **Correspondence:** K. Gecse

*The Journal of Headache and Pain 2024*, **25(Suppl 1):**LP019

**Objective:** Previous studies demonstrated that altered kynurenine pathway may play a role in migraine patomechanism. Its metabolites could modulate the periaqueductal gray matter (PAG) projections which is important in pain perception and modulatory system. However, this is the first study investigating a relationship between plasma L-kynurenine concentration and PAG functional connectivity in migraine patients.

**Methods:** Functional connectivity (FC) analysis with PAG as seed region was conducted involving 26 episodic migraine without aura patients and 33 headache-free, healthy controls. Blood samples were collected to measure plasma L-kynurenine concentration before fMRI session. Participants were headache and medication free during the experiment. To investigate the differences between the relationship of kynurenine concentration and PAG-FC in migraineurs compared to healthy controls, kynurenine concentration was used as covariate in interaction with PAG-FC in two sample t-tests using the Statistical Parametric Mapping (SPM12) toolbox in MATLAB environment.

**Results:** Significant difference was found between migraine and healthy controls in the relationship of kynurenine concentration and PAG-FC with inferior occipital gyrus (pFWE=0.019). Post-hoc analysis revealed a negative correlation between kynurenine concentration and connectivity of PAG with inferior occipital gyrus in migraine patients (pFWE<0.001, k=237, Peak-T value=-4.718). However, there was no significant correlation in healthy controls.

**Conclusion:** Our results suggest that the kynurenine pathway may play an important role in migraine related hyperexcitability. Thus, the decreased functional connectivity between PAG and occipital gyrus in association with increased plasma kynurenine concentration might be involved in hypersensitive sensory and pain processing circuits of migraine patients.

**Funding:** ÚNKP-23-4-I-SE-31, 2017-1.2.1-NKP-2017-00002, NAP2022-I-4/2022; TKP2021-EGA-25; OTKA (K143391); ERA PerMed (2019-2.1.7-ERA-NET-2020-00005)

## LP020 Serum neurofilament light chain levels in migraine patients: a monocentric case–control study in China

### J. Fang^1,2,3,4,5,6,7,8^, J. Wu^1,8,9^, T. Zhang^10^, X. Yuan^11^, J. Zhao^2^, L. Zheng^1,2,3,4,5,6,7^, G. Hong^12^, L. Yu^13^, Q. Lin^1,2,3,4,5,6,7^, X. An^1,2,3,4,5,6,7^, C. Jing^1,2,3,4,5,6,7^, Q. Zhang^1,2,3,4,5,6,7^, C. Wang^1,2,3,4,5,6,7^, Z. Wang^2,3,4,5,6,7,8,9,14^, Q. Ma^1,2,3,4,5,6,7,8,9^

#### ^1^The First Affiliated Hospital of Xiamen University, School of Medicine,Xiamen University, Department of Neurology and Department of Neuroscience, Xiamen, China; ^2^Fujian Medical University, The School of Clinical Medicine, Fuzhou, China; ^3^Fujian Key Laboratory of Brain Tumors Diagnosis and Precision Treatment, Xiamen, China; ^4^Xiamen Key Laboratory of Brain Center, Xiamen, China; ^5^Xiamen Medical Quality Control Center for Neurology, Xiamen, China; ^6^Fujian Provincial Clinical Research Center for Brain Diseases, Xiamen, China; ^7^Xiamen Clinical Research Center for Neurological Diseases, Xiamen, China; ^8^Xiamen University, School of Medicine, Xiamen, China; ^9^Xiamen University, National Institute for Data Science in Health and Medicine, Xiamen, China; ^10^The Fifth Hospital of Xiamen, Department of Neurology, Xiamen, China; ^11^Department of Gynecology, Xiamen Maternal and Child Health Care Hospital, Xiamen, China; ^12^Zhangzhou Hospital of Fujian Province, Cerebrovascular Interventional Department, Zhangzhou, China; ^13^Changxing People’s Hospital, Department of Neurology, Huzhou, China; ^14^The First Affiliated Hospital of Xiamen University, School ofMedicine, Xiamen University, Department of Neurosurgery and Department of Neuroscience, Xiamen, China

##### **Correspondence:** J. Fang; J. Wu

*The Journal of Headache and Pain 2024*, **25(Suppl 1):**LP020

**Objective:** Serum neurofilament light chain (sNfL) can reflect nerve damage. Whether migraine can cause neurological damage remain unclear. This study assesses sNfL levels in migraine patients and explores whether there is nerve damage in migraine.

**Methods:** A case–control study was conducted in Xiamen, China. A total of 138 migraine patients and 70 healthy controls were recruited. sNfL (pg/mL) was measured on the single-molecule array platform. Univariate, Pearson correlation and linear regression analysis were usedto assess the relationship between migraine and sNfL levels, with further subgroup analysis by migraine characteristics.

**Results:** Overall, 85.10% of the 208 subjects were female, with a median age of 36 years. sNfL levels were higher in the migraine group than in the control group (4.85 (3.49, 6.62) vs. 4.11 (3.22, 5.59)), but the difference was not significant (*P*=0.133). The two groups showed an almost consistent trend in which sNfL levels increased significantly with age. Subgroup analysis showed a significant increase in sNfL levels in patients with a migraine course ≥ 10 years (β=0.693 (0.168, 1.220), *P*=0.010). Regression analysis results show that age and migraine course are independent risk factors for elevated sNfL levels, and there is an interaction between the two factors. Patients aged < 45 years and with a migraine course ≥ 10 years have significantly increased sNfL levels.

**Conclusion:** This is the first study to evaluate sNfL levels in migraine patients. The sNfL levels significantly increased in patients with a migraine course ≥ 10 years. More attention to nerve damage in young patients with a long course of migraine is required.

**Fig. 1 (Abstract LP020):**
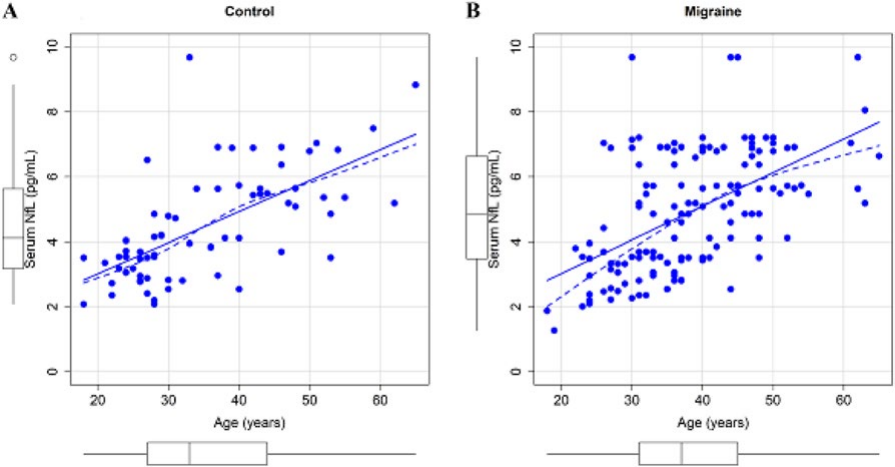
See text for description

**Fig. 2 (Abstract LP020):**
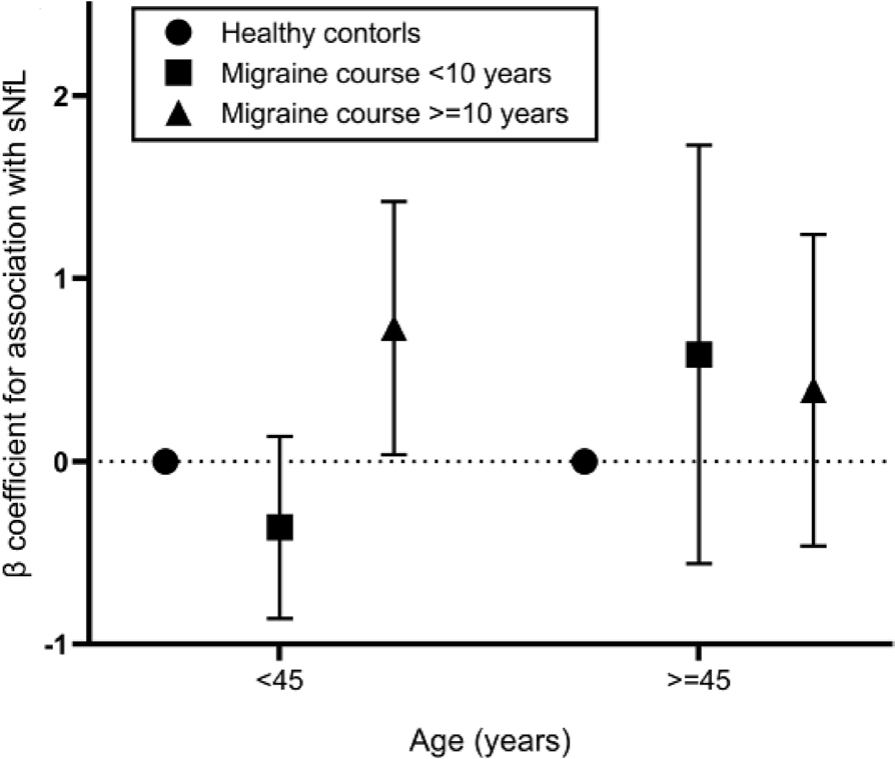
See text for description

## LP021 Transcriptomic signatures of stress in migraineurs: a GWAS-connected approach

### S. Kumar^1,2^, K. Gecse^1,2^, D. Baksa^1,2^, G. Bagdy^1,2^, G. Juhasz^1,2^, P. Petschner^1,3,4^

#### ^1^Semmelweis University, Department of Pharmacodynamics, Budapest, Hungary; ^2^Semmelweis University, NAP-3.0-SE Neuropsychopharmacology Research Group, Hungarian Brain Research Program, Budapest, Hungary; ^3^Kyoto University, Bioinformatics Center, Institute for Chemical Research, Uji, Kyoto, Japan; ^4^Kyoto University, Research Unit for Realization of Sustainable Society, Gokasho, 611-0011, Uji, Kyoto, Japan

##### **Correspondence:** S. Kumar

*The Journal of Headache and Pain 2024*, **25(Suppl 1):**LP021

**Objective:** This study aimed to investigate transcriptomic changes in migraine without aura (MO). Perception in migraine patients differs from that of healthy individuals (HC) and is potentially influenced by stress. To test such differences, we conducted RNA sequencing on a well-defined MO population following i.v. placebo infusion, analyzed differences in pathways, the corresponding leading edges (LEGs), and their association with recent GWAS data.

**Methods:** We studied 22 episodic MO patients (17 females) in the interictal phase, and 30 HC (16 females), aged 20-37, who weren't on regular medication except contraceptives or occasional headache remedies. RNA sequencing was performed on whole blood samples, with each individual having baseline and post-placebo measurements. Comparisons were made between MO patients and controls after correction for their pre-placebo baselines. We used fGSEA with Canonical Pathways and Gene Ontology gene sets to identify pathways. LEGs of significant pathways (FDR < 0.05) were checked against the recent migraine GWAS literature.

**Results:** Positively enriched pathways after placebo challenge included Chromatin Organization, Positive Regulation of Histone Modification, Regulation of Histone Methylation, and Regulation of Histone Modification when comparing MO patients with HCs. The PHF20 gene emerged as an important LEG behind these pathways and was also found in one GWAS.

**Conclusion:** This study revealed an enrichment of epigenetic pathways in MO patients compared to HCs with the potential role of PHF20 driving these enrichments. PHF20's association with stress-induced autophagy was identified in a study using mouse embryonic fibroblasts under glucose amino acid starvation conditions. Taken together, administering placebo infusion to MO patients seemed to induce a stress response, highlighting a potential mechanism behind the heightened susceptibility of MO patients to stress.

## LP022 Altered cortical morphometry in migraine: a Registry for Migraine (REFORM) MRI study

### R. Hackert Christensen^1^, H. Ashina^1^, H. Muhsen Al-Khazali^1^, Y. Zhang^1^, D. Tolnai^2^, A. Cagol^3^, N. Hadjikhani^4^, C. Granziera^3^, F. M. Amin^1^, M. Ashina^1^

#### ^1^The Danish Headache Center, Department of Neurology, Glostrup, Denmark; ^2^Rigshopitalet Glostrup, Department of Radiology, Glostrup, Denmark; ^3^University Hospital Basel and University of Basel, Department of Biomedical Engineering, Basel, Switzerland; ^4^Massachusetts General Hospital, Athinoula A. Martinos Center for Biomedical Imaging, Boston, United States

##### **Correspondence:** R. Hackert Christensen

*The Journal of Headache and Pain 2024*, **25(Suppl 1):**LP022

**Objective:** Structural imaging can offer insights into the cortical morphometry of migraine, which might reflect adaptations to recurring nociceptive messaging. This study expands upon prior findings by comparing cortical morphometry between a large sample of people with migraine and healthy controls, as well as across migraine subtypes.

**Methods:** Adult participants with migraine and age- and gender-matched healthy controls attended a single MRI session with magnetization-prepared rapid acquisition gradient echo (MPRAGE) and fluid-attenuated inversion recovery (FLAIR). Cortical surface area, thickness, and volume were compared between participants with migraine (including subgroups) and healthy controls across the whole cortex. The analysis used a cluster-determining thresholds of *P* < .0001 and clusterwise thresholds of *P* < .05, adjusted for age and gender.

**Results:** A total of 296 participants with migraine *(mean age 41.6 years ± 12.4 SD, 261 females)* and 155 healthy controls *(mean age 41.1 years ± 11.7 SD, 133 females)* were included. The participants with migraine had reduced cortical surface area in the left insula, compared with controls (*P* < .0001). Furthermore, participants with chronic migraine (*n* = 180) exhibited reduced surface area in the left insula (*P* < .0001) and increased surface area in the right caudal anterior cingulate cortex (*P* < .0001), compared with controls.

**Conclusion:** The identified cortical changes in migraine were limited to specific pain processing regions, including the insula and caudal anterior cingulate gyrus, and were most notable in participants with chronic migraine. These findings suggest persistent cortical changes associated with migraine.

**Fig. 1 (Abstract LP022):**
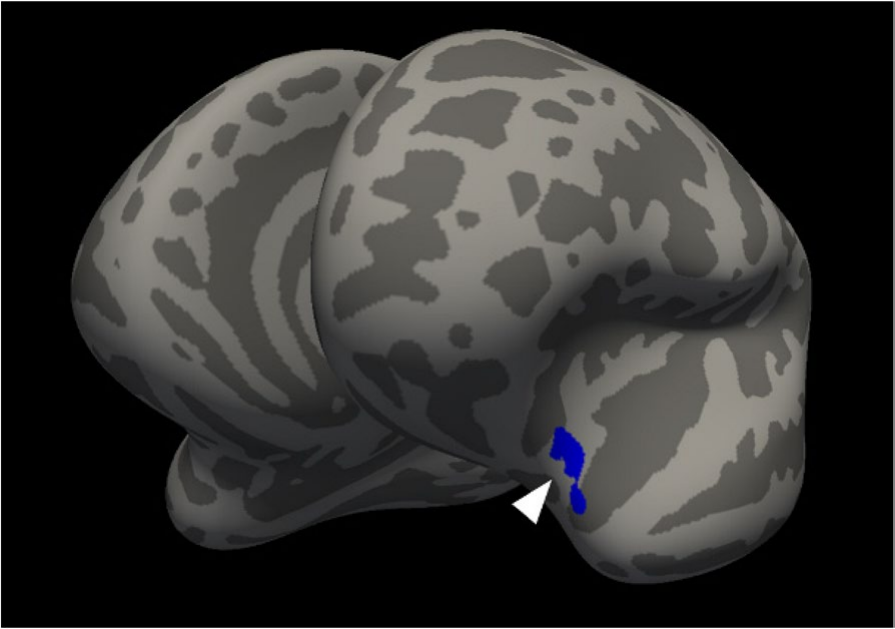
See text for description

**Fig. 2 (Abstract LP022):**
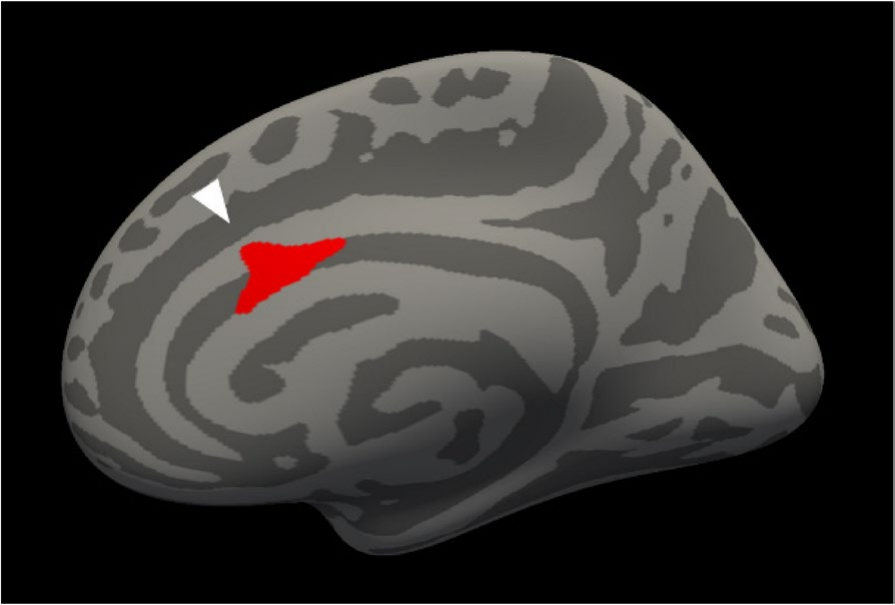
See text for description

**Fig. 3 (Abstract LP022):**
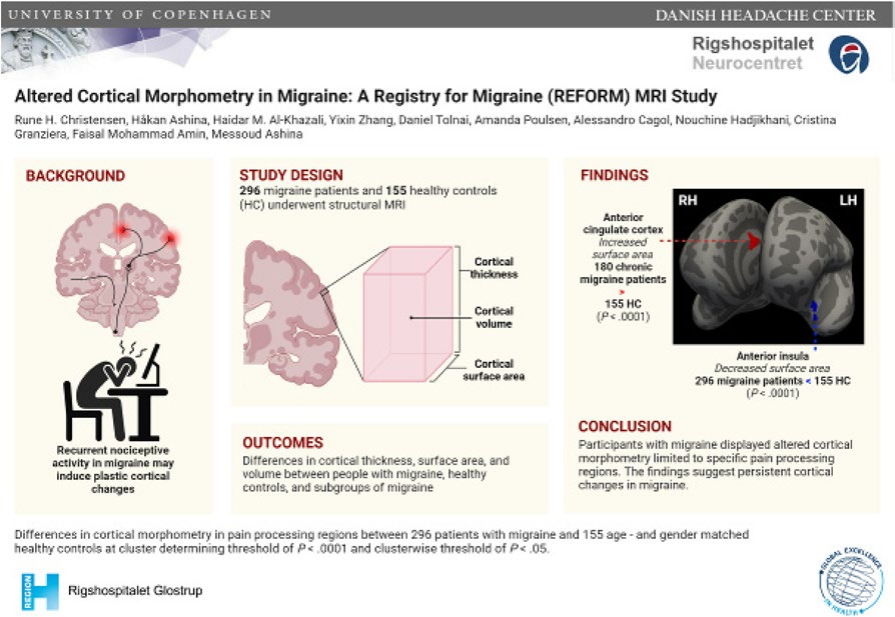
See text for description

## LP023 Does physical activity and inflammation mediate the job stress-headache relationship? A sequential mediation analysis in the ELSA-Brasil study

### A. Oliveira^1^, H. Schytz^1,2^, M. Peres^3^, J. Mercante^4,3^, A. Brunoni^3^, Y. P. Wang^3^, M. D. C. Molina^5^, L. Koji Uchiyama ^1^, P. Lotufo^1^, R. Jensen^2^, I. Benseñor^1^, R. Griep^6^, A. Goulart^1^

#### ^1^Universidade de São Paulo, Center for Clinical and Epidemiological Research, Sao Paulo, Brazil; ^2^University of Copenhagen, Danish Headache Center, Department of Neurology, Glostrup, Denmark; ^3^Universidade de São Paulo, Instituto de Psiquiatria, Sao Paulo, Brazil; ^4^Universidade de São Paulo, Center For Clinical and Epidemiological Research, Casa Branca, Brazil; ^5^Universidade Federal de Ouro Preto, Ouro Preto, Brazil; ^6^Instituto Oswaldo Cruz, Fundação Oswaldo Cruz, Laboratório de Educação em Ambiente e Saúde, Rio de Janeiro, Brazil

##### **Correspondence:** A. Oliveira

*The Journal of Headache and Pain 2024*, **25(Suppl 1):**LP023

**Objective:** To test whether physical activity and high-sensitivity C-reactive protein (hs-CRP) would mediate the associations of job stress with headache disorders.

**Methods:** We cross-sectionally evaluated the baseline data from the Brazilian Longitudinal Study of Adult Health (ELSA-Brasil) study regarding to job stress (higher demand and lower control and support subscales), migraine and tension-type headache (ICHD-2 criteria), self-reported leisure-time physical activity, and serum hs-CRP levels. Conditional process analyses with a sequential mediation approach were employed to compute path coefficients and 95% confidence intervals (IC) around the indirect effects of physical activity and hs-CRP levels in the job stress-headache relationship. Separate models were adjusted for sex, age, depression and anxiety, socioeconomic factors, BMI, and smoking status.

**Results:** In this sample (*n* = 7,644), the 1-year prevalence of migraine and tension-type headache were 13.1% and 49.4%, respectively. Higher job stress in all subscales was associated with migraine. Control exhibited a positive and inverse association with physical activity (B = 5.99, 95% CI: 4.54, 7.44) and migraine (B = -0.039, 95% CI: -0.074 to -0.010), respectively. Physical activity was inversely associated with hs-CRP [B. = -0.008 (95%IC: -0.0010, -0.005)] and migraine [B = -0.006 (95%IC: -0.0011, -0.002)]. The association between control and migraine was mediated by physical activity [effect = -0.039 (95%IC: -0.074, -0.010)] regardless of sex, age, anxiety, and depression but not after the addition of socioeconomic factors, BMI, and smoking status. [WYP1] [AO2] Higher control was positively associated with TTH (B = -0.037, 95% CI: (0.0218, 0.0530)[AO3] . Neither the job stress-migraine link nor the job stress-tension-type headache relationship exhibited any other mediating effects.

**Conclusion:** Findings from baseline data of the ELSA-Brasil have indicated that physical activity but not hs-CRP, mediated the relationship between domain-specific job stress and migraine, but not tension-type headache.

## LP024 Connective tissue, autonomic and autoimmune differences between Vestibular Migraine and Chronic Migraine

### M. D. Villar Martinez, D. Cheung, D. Moreno-Ajona, K. Nagaraj, P. Goadsby

#### King’s College London, London, United Kingdom

##### **Correspondence:** M. D. Villar Martinez

*The Journal of Headache and Pain 2024*, **25(Suppl 1):**LP024

**Objective:** Vestibular migraine (VM) presents more allodynia, although their response to CGRP antibodies is similar to that of migraine patients without vestibular symptoms. We aimed to understand whether VM patients present with similar comorbidities to chronic migraine (CM) patients in which the vestibular component is not the most prominent symptom

**Methods:** Cross-sectional study as a service evaluation of the headache and neuro-otology and general neurology clinics at King"s College Hospital and Charing Cross Hospital in London from February 2020 to July 2023. We reviewed the past medical history from the referral and first medical interview of consecutive cases that had been diagnosed with either CM or VM. We compared the presence of Postural Orthostatic Tachycardia syndrome (POTS), Ehler-Danlos syndrome (EDS) or hypermobility, and autoimmune conditions. Groups were compared using χ^2^ at *P* < 0.05 with Bonferroni correction for multiple comparisons, SPSS 28.

**Results:** Of 525 cases, 185 had a diagnosis of vestibular migraine and 340 chronic migraine. Mean age and percentage of males were not significantly different between groups. The presence of POTS (5.4% vs 2.4%, respectively, χ^2^=3.47, *P*=0.05), inflammatory conditions (35.7% vs 24.7%, χ^2^=7.06, *P*<0.01) and hypermobility (4.4% vs 3.8%, χ^2^=0.07, NS) was higher in VM than CM patients.

**Conclusion:** Our results show a higher proportion of POTS among patients with VM, in comparison with CM and the 0.1-1% prevalence reported in the literature. Cranial autonomic dysfunction in patients with migraine has been thoroughly described before and dysautonomia symptoms have been correlated with chronification of headache in patients with POTS. VM may present an even more dysfunctional autonomic system, which could, similarly, affect different organs. Autoimmune conditions, such as rheumatoid arthritis or psoriasis, have demonstrated a potential comorbidity and may have a genetic association with migraine. More studies are needed to help elucidating the mechanisms

**Fig. 1 (Abstract LP024):**
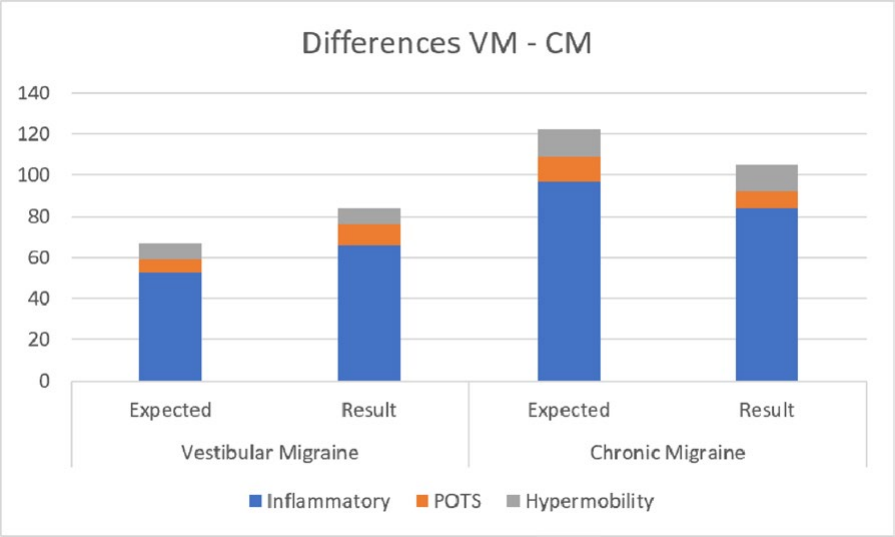
Differences VM-CM

## LP025 The link between higher cortical dysfunctions during migraine aura and volume of the hippocampus

### I. Petrušić^1^, A. Radojičić^2^, M. Daković^1^

#### ^1^University of Belgrade, Faculty for Physical Chemistry, Belgrade, Serbia; ^2^University of Belgrade, Faculty of Medicine, Belgrade, Serbia

##### **Correspondence:** I. Petrušić

*The Journal of Headache and Pain 2024*, **25(Suppl 1):**LP025

**Objective:** The role of the hippocampus is insufficiently explored in migraine with aura (MwA). Moreover, the heterogeneity of MwA patients prevents studies to investigate particular roles of specific brain regions in MwA pathophysiology. This study aimed to compare subgroups of MwA patients and healthy controls (HCs) regarding the volume of the hippocampus.

**Methods:** MwA patients were assigned to the MwA-HCD group if they reported higher cortical dysfunctions (HCDs) during the aura or to the MwA-non-HCD group if they did not experience HCDs. Both subgroups were compared in between and to HCs to evaluate any specific changes in the hippocampus. Neuroimaging data derived from FreeSurfer-based segmentation of hippocampal subfields acquired from a 3 T magnetic resonance machine was used for comparisons.

**Results:** A total of 46 MwA patients (28 MwA-HCD group and 18 MwA-non-HCD group) and 31 HCs were studied. There were no significant differences in age and sex between groups (*p*=0.490 and *p*=0.934, respectively). The MwA-HCD group had significantly smaller volumes of the left (3328 vs. 3677 mm3, *p*<0.001) and right (3395 vs. 3748 mm3, *p*<0.001) hippocampus compared to the MwA-non-HCD group. Also, the MwA-HCD group had significantly smaller volumes of the left (3328 vs. 3541 mm3, *p*=0.002) and right (3395 vs. 3592 mm3, *p*=0.015) hippocampus compared to HCs. There was no significant difference between the MwA-non-HCD group and HCs relative to the hippocampal volumes.

**Conclusion:** Smaller volumes of the left and right hippocampus might play an important role in the pathophysiology of HCDs during MwA attacks. Further functional neuroimaging studies are needed to explain the results of this study.

## LP026 Hypersensitivity to PACAP-38 in post-traumatic headache: a randomized clinical trial

### H. Al-Khazali

#### Rigshospitalet, Danish Headache Center, Glostrup, Denmark

*The Journal of Headache and Pain 2024*, **25(Suppl 1):**LP026

**Objective:** To ascertain whether intravenous infusion of pituitary adenylate cyclase-activating polypeptide-38 (PACAP-38) induces migraine-like headache in people with persistent post-traumatic headache (PTH) attributed to mild traumatic brain injury.

**Methods:** A randomized, double-blind, placebo-controlled, 2-way crossover trial was conducted at a single center. Participants were randomly assigned to receive a 20-minute continuous intravenous infusion of either PACAP-38 (10 pmol/kg/min) or placebo (isotonic saline) on two separate experimental days, with a 1-week wash-out period in between. The primary outcome was the difference in incidence of migraine-like headache between PACAP-38 and placebo during a 12-hour observational period post-infusion. The secondary outcome was the difference in the area under the curve (AUC) for baseline-corrected median headache intensity scores during the same 12-hour observational period.

**Results:** Of 49 individuals assessed for eligibility, 21 were enrolled and completed the trial. The participants had a mean age of 35.2 years, and 16 (76%) were women. Most of them (19 [90%] of 21) had a migraine-like phenotype. During the 12-hour observational period, 20 (95%) of 21 participants developed migraine-like headache after intravenous infusion of PACAP-38, compared with 2 (10%) participants after placebo (*P* < 0.001). Furthermore, the baseline-corrected AUC values for median headache intensity scores during the 12-hour observational period was higher after PACAP-38 than placebo (*P* < 0.001).

**Conclusion:** These compelling results demonstrate that PACAP-38 is potent inducer of migraine-like headache in people with persistent PTH. Thus, targeting PACAP-38 signalling might be a promising avenue for the treatment of PTH.

**Fig. 1 (Abstract LP026):**
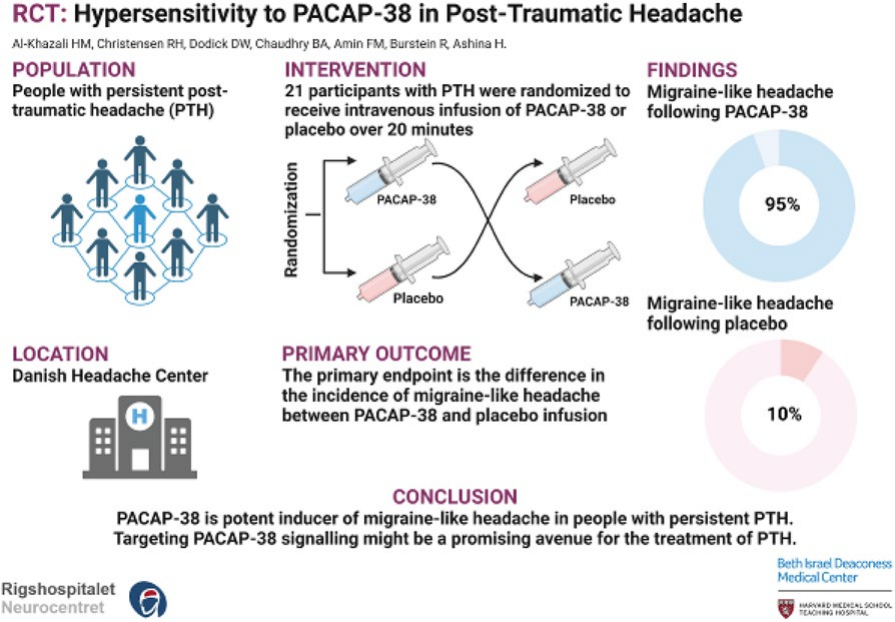
See text for description

## LP028 A pilot study of stereoelectroencephalography electrodes in a patient with refractory chronic headache: personalized targets for precise deep brain stimulation

### Z. Dong^1^, H. Zhao^1^, S. Zhang^1^, Y. Wang^2^, C. H. Yeh ^2^, S. Yu^1^

#### ^1^Chinese PLA General Hospital, Department of Neurology, Beijing, China; ^2^School of Information and Electronics, Beijing Institute of Technology, Beijing, China

##### **Correspondence:** Z. Dong

*The Journal of Headache and Pain 2024*, **25(Suppl 1):**LP028

**Objective:** The integration of stereoelectroencephalography with therapeutic DBS holds immense promise as a viable approach for precise treatment of refractory disorders, yet it has not been explored in the domain of headache or pain management.

**Methods:** We implanted 14 stereoelectroencephalography electrodes in a patient with refractory migraine for clinical monitoring and electrophysiological recording. During monitoring, we collected the VAS score in 5-min increments, and recorded electrophysiological data in real-time. Data were classified into two types of symptoms (high and low symptoms) for determining the spectral power features of specific brain regions reflecting pain fluctuations, which we called Biomarker, using statistical analyses and cross-validated machine-learning models. During stimulation, we tested the clinical effect through a systematic bipolar stimulation survey, and collected real-time electrophysiological data. Based on the identification of brain areas with clinical improvement, the optimal target for stimulate was determined by validating the clinical response against the biomarker, and phase-amplitude coupling finally.

**Results:** For biomarker, RNAc-HFO was the most considerably correlated with VAS score (rho = 0.5292, *p* < 0.0001), and differed significantly between mild and severe pain levels (*p* = 0.0003), also with the greatest weighting in the characteristic ranking. The machine-learning model showed an accuracy and AUC remaining at 0.75 and 0.77, respectively, for RAC- HFO. For target, LdACC was identified as the most effective stimulation target, based on the VAS score reported over the stimulation period. VAS score (*p* = 0.006), RNAc- HFO (*p* = 0.0029) were significantly improved after stimulation compared to pre-stimulation in LdACC. The significant modulatory effect of RNAc- HFO by the low-frequency phase of LdACC also confirmed the modulatory effect of LdACC and RNAc during headache fluctuation.

**Conclusion:** In this pilot study, the concept of the herein-proposed data-driven approach to optimizing precise and personalized treatment strategies for DBS may create a new frontier in the field of refractory headache and even pain disorders.

**Fig. 1 (Abstract LP028):**
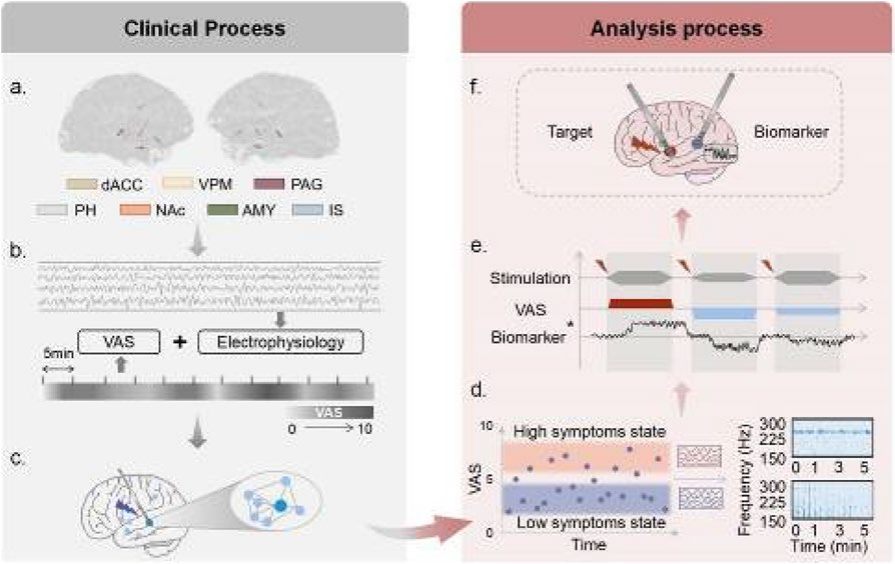
See text for description

**Fig. 2 (Abstract LP028):**
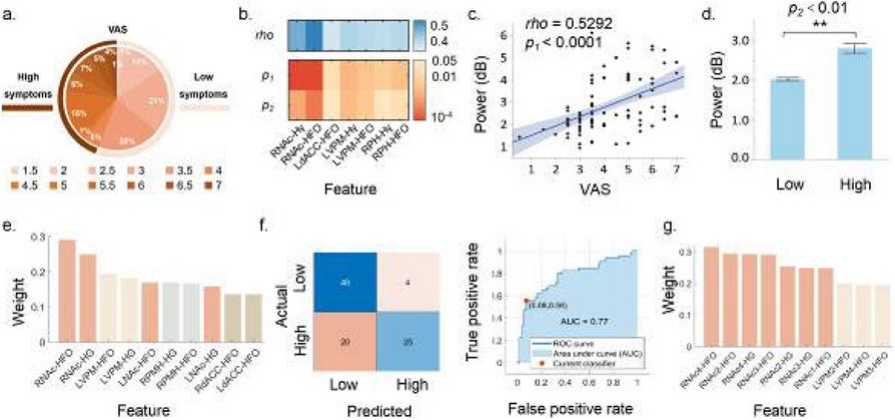
See text for description

**Fig. 3 (Abstract LP028):**
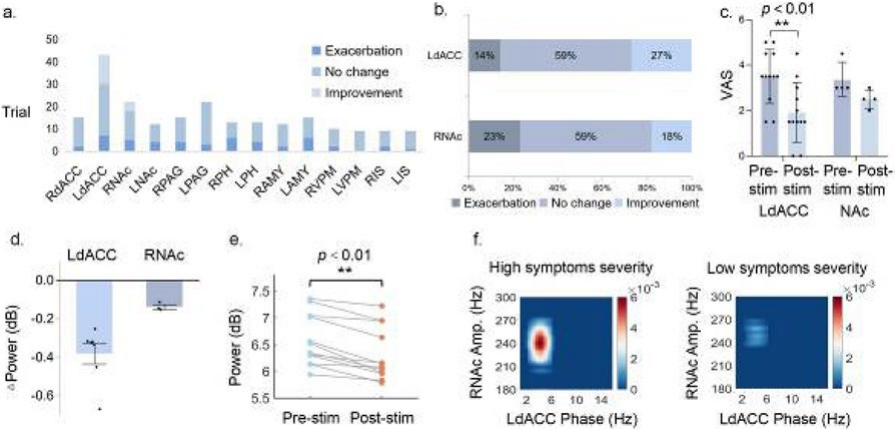
See text for description

## LP029 Visual and cognitive dysfunctions in migraine with aura: ERSP naming task study

### V. Jovanović^1^, I. Petrušić^2^

#### ^1^Faculty of Philosophy, Belgrade, Serbia; ^2^Faculty for Physical Chemistry University of Belgrade, Belgrade, Serbia

##### **Correspondence:** I. Petrušić

*The Journal of Headache and Pain 2024*, **25(Suppl 1):**LP029

**Objective:** Accurately diagnosing migraine and identifying specific biomarkers that can reliably indicate the disorder, regardless of the ongoing phase, remains a persistent challenge. As a distinctive symptom characterizing migraine with aura (MwA), various impairments within the visual system have been identified, including difficulties in object recognition and naming.

**Methods:** In our study, we utilized an overt naming task involving both easy and challenging visual stimuli to explore how processing differs between individuals with MwA during their interictal phase and healthy controls (HC). A total of 30 participants took part in this study, including 15 MwA patients and 15 HC. We employed event-related spectral perturbations (ERSP) analysis to examine specific regions of interest and uncover the complexities of dynamics across various frequency ranges.

**Results:** Compared to the HC group, individuals with MwA exhibited diminished ERSP values in theta/alpha band oscillations (4-12Hz) within occipital regions, along with reduced alpha-band activation (8-12Hz) in the left temporal cortex within the initial 100 milliseconds. A weaker mid-beta-band (15-20Hz) desynchronization was observed after 200 milliseconds in the left parietal lobe, persisting until the response phase. These findings were consistent across both stimulus conditions (easy and difficult-to-name).

**Conclusion:** Our study aligns with prior research that indicates alpha and beta activity disruptions in migraines, even within the interictal phase. Furthermore, it provides additional insights into the somatosensory cortex's activity during object identification and naming processes.

## LP030 Comorbid depression, cognitive impairment, and migraine in Azerbaijani population: a comprehensive study

### I. Azizova^1,2^, R. Hasanov^3^

#### ^1^Neurology Clinic "New Medical Technologies", Baku, Azerbaijan; ^2^Medipoint, Baku, Azerbaijan; ^3^Azerbaijan State Advanced Training Institute for Doctors named after A. Aliyev, Department of Neurology and Clinical Neurophysiology, Baku, Azerbaijan

##### **Correspondence:** I. Azizova

*The Journal of Headache and Pain 2024*, **25(Suppl 1):**LP030

**Objective:** This study aimed to explore the comorbidity of depression and cognitive impairment in Azerbaijani individuals diagnosed with migraines. Specifically, the objectives were to assess the prevalence and severity of depression in this population, evaluate their cognitive functioning with a focus on memory-related complaints, investigate the potential relationship between depression, cognitive impairment, and migraines, and consider the implications for enhancing migraine management and overall well-being.

**Methods:** We enrolled 346 participants clinically diagnosed with migraines, including 324 who reported memory-related complaints. Depression levels were evaluated using the Hamilton Depression Rating Scale (HAM-D), categorizing participants into mild, moderate, and severe depression categories. Cognitive assessment was conducted using the Montreal Cognitive Assessment (MOCA). Data were analyzed to determine depression prevalence and the extent of cognitive impairment in those with memory-related complaints.

**Results:** The findings revealed significant depression comorbidity within the Azerbaijani migraine population, with 223 individuals experiencing mild depression, 82 having moderate depression, and 19 enduring severe depression. Notably, among the 324 participants reporting memory issues, 87.96% exhibited mild cognitive impairment, while 12.04% had moderate cognitive dysfunction.

**Conclusion:** This research underscores the intricate interplay between depression, cognitive impairment, and migraines in Azerbaijani individuals. It challenges the conventional notion of direct cognitive deficits in migraine patients, suggesting a strong link between cognitive dysfunction and the presence and severity of depressive symptoms. The study underscores the importance of adopting a comprehensive approach to migraine management, recognizing the multifaceted nature of this condition.

## LP031 Impact of comorbid primary headache on cognitive function of patients with multiple sclerosis

### M. Andriievska

#### Vinnytsia National Pirogov memorial Medical University, Department of Nervous Disorders, Vinnitsya, Ukraine

*The Journal of Headache and Pain 2024*, **25(Suppl 1):**LP031

**Objective:** Primary headaches, especially migraine, are highly linked to multiple sclerosis (MS). We aimed to compare groups of MS patients with and without comorbid primary headache (CPH) in cognitive function evaluation.

**Methods:** The Symbol Digit Modalities Test (SDMT), oral type, was used to assess cognitive function among patients with MS and CPH. Statistical analysis was performed in Microsoft Excel (mean with standard deviation SD, t-test, confidence interval). P-value ≤0.05 was considered significant.

**Results:** A total of 130 patients with MS (63,9% women) were included, 73 with CPH (56,2%). A median age of headache onset - of 18 [12-57] years (y). Age of MS onset in a group with CPH - of 27,8 ± 7,96 y, without CPH - 27,3 ± 6,7. Duration of headache - 4,9 ± 6,44 y. The next types of CPH were presented among patients with MS: 54% of migraine (*n*=40), 41% of tension type headache (*n*=30), 5% vegetative cephalgias (*n*=3). The mean SDMT score among all MS patients was 48.72 ± 13.71. Among men with MS - 46.47 ± 14.33, among women - 50.0 ± 13.26, the difference between the indicators is not clinically significant. Patients with comorbid migraine [52,4±12,39] performed the test better than patients with comorbid GBN [43,6±12,9], *p*=0,06 . CPH reduced the SDMT score among patients with MS [-0.198; -0.189]. The longer the duration of CPH, the lower was the SDMT index [ -0.472; -0.467]. It demonstrates the negative impact of CPH on the cognitive ability of patients with MS. A relationship was found between the age of onset of GB and the SDMT score: with a later onset of CPH, the indicators of the SDMT score were worse [-0.272; -0.265]. It is likely that cognitive function worsened with later onset of CPH because compensatory mechanisms to prevent the progression of neurodegeneration in MS become weaker with age.

**Conclusion:** CPH had a negative impact on cognitive function in patients with MS. Later onset of CPH showed worse SDMT score. Patients with comorbid migraine performed SDMT evaluation better than patients with comorbid tension type headache.

## LP032 Clinical predictors of therapeutic failure of occipital nerve stimulation in refractory chronic cluster headache

### J. A. Membrilla^1^, M. L. Cuadrado^2^, N. Gonzalez-Garcia^2^, J. Porta-Etessam^2^, A. Sanchez-Soblechero^3^, A. Lozano-Ros^3^, A. Gonzalez-Martinez^4^, A. B. Gago-Veiga^4^, S. Quintas^4^, J. S. Rodriguez-Vico^5^, A. Jaimes^5^, L. Llorente-Ayuso^6^, J. Roa^5^, C. Estebas^7^, J. Diaz-de-Teran^7^

#### ^1^Hospital Universitari Francesc de Borja, Gandia, Spain; ^2^Hospital Universitario Clínico San Carlos, Madrid, Spain; ^3^Hospital Universitario Gregorio Marañón, Madrid, Spain; ^4^Hospital Universitario La Princesa, Madrid, Spain; ^5^Hospital Universitario Fundación Jiménez Díaz, Madrid, Spain; ^6^Hospital Universitario Infanta Leonor, Madrid, Spain; ^7^Hospital Universitario La Paz, Madrid, Spain

##### **Correspondence:** J. A. Membrilla

*The Journal of Headache and Pain 2024*, **25(Suppl 1):**LP032

**Objective:** Occipital nerve stimulation (ONS) is a treatment with evidence in refractory chronic cluster headache (CCH). However, the variable response rate and cost make it necessary to investigate predictors of response.

**Methods:** This is a cross-sectional study conducted through the review of medical records of CCH patients from six hospitals in Madrid. Epidemiological and clinical variables were compared between patients with ONS failure and the rest. ONS failure was defined as the need for device withdrawal or switch off because of lack of response or adverse events.

**Results:** From a series of 88 CCH, 26 (29.6%) were treated with ONS, of which 13/26 (50.0%) failed. In all of them the cause was non-response. The ONS failure group had earlier headache debut (mean 27.7 years SD 6.9 vs 36.7 years SD 11.8, *p*=0.026) and a higher rate of active smoking (100% vs 42.9%, *p*=0.006), as well as the presence of seasonal exacerbations (58.3% vs 7.7%, *p*=0.007) and nocturnal exacerbations (91.7% vs 53.9%, *p*=0.035). There were no differences between groups in diagnostic delay, years of evolution prior to surgery, psychiatric pathology or comorbidity with other headaches or other chronic pain syndromes. There were also no differences in previous response to anesthetic blocks or other treatments.

**Conclusion:** Some clinical features such as an early debut, smoking, and seasonal or circadian fluctuations could be related to failure of ONS in refractory CCH.

## LP033 The lifelines diet score and migraine headaches: results from a case-control study

### M. Noormohammadi^1^, S. Razeghi Jahromi^2^, M. Togha^3^, S. Ariyanfar^4^

#### ^1^Iran University of Medical Sciences, Department of Nutrition, School of Public Health, Tehran, Iran; ^2^Shahid Beheshti University of Medical Sciences, Department of Clinical Nutrition and Dietetics, Faculty of Nutrition and Food Technology, Tehran, Iran; ^3^Tehran University of Medical Sciences, Headache Department, Iranian Centre of Neurological Research, Neuroscience Institute, Tehran, Iran; ^4^Virginia Tech, Department of Human Nutrition, Foods, and Exercise, College of Agriculture and Life Science, Blacksburg, VA 24060, United States

##### **Correspondence:** S. Razeghi Jahromi

*The Journal of Headache and Pain 2024*, **25(Suppl 1):**LP033

**Objective:** Migraine headaches are a common health problem responsible for disability that affects about one in eight people worldwide. Migraine and its symptoms may be improved by different dietary approaches. The Lifelines Diet Score (LLDS) is a method for assessing the relative quality of the diet, based on the Dutch diet guidelines for 2015. The current study aimed to investigate the association between LLDS and odds of migraine headaches.

**Methods:** This case-control study involved newly diagnosed adults with migraine headaches, according to the International Classification of Headache Disorders 3rd edition (ICHD-III criteria), and healthy controls. A validated 168-item semi-quantitative food frequency questionnaire (FFQ) was used to collect the dietary intakes of the participants. LLDS was calculated based on the method of Vinke et al.

**Results:** According to the crude model, the highest quartile of LLDS was associated to a 68% reduced odds of migraine headaches (odds ratios (OR): 0.32, 95% confidence intervals (CI): 0.21, 0.48, Ptrend < 0.001). After adjusting for total calories intake, age, sex and body mass index, multivariable logistic regression analysis revealed that, compared with the lowest quartile, the highest quartile of LLDS had a 71% decreased odds of migraine headaches (adjusted odds ratios (aOR): 0.29, 95%CI: 0.18, 0.46, Ptrend < 0.001).

**Conclusion:** The highest LLDS, which consists of nine food groups that are beneficial for health, such as vegetables, fruits, whole grain products, legumes and nuts, fish, oils and soft margarines, unsweetened dairy, coffee, and tea, and emphasizes on reducing consumption of three food groups that are detrimental for health, such as red and processed meat, butter and hard margarines, and sugar-sweetened beverages, may have a positive impact on migraine headache prevention.

## LP034 Progesterone distribution in the trigeminal system and its role to modulate sensory neurotransmission: influence of sex

### A. Maddahi, L. Edvinsson

#### Lunds University, Clinical science, Lund, Sweden

##### **Correspondence:** A. Maddahi

*The Journal of Headache and Pain 2024*, **25(Suppl 1):**LP034

**Objective:** Women are disproportionately affected by migraine. This discrepancy has been proposed to be influenced by differences in sex hormone levels. One such hormone is progesterone. The calcitonin gene-related peptide (CGRP) system is an important factor in migraine pathophysiology and could be influenced by circulating hormones. The purpose of this study was to investigate the distribution of progesterone and its receptor (PR) in the trigeminovascular system, and to examine the role of progesterone in sensory neurotransmission.

**Methods:** Expression of progesterone and PR proteins, and mRNA levels from TG and hypothalamus were analyzed by immunohistochemistry and RT-qPCR. CGRP release from TG and dura mater were measured using ELISA. The vasomotor effect of progesterone on male and female basilar artery was investigated with myography.

**Results:** In TG, progesterone was located predominantly in cell membrane and in Aδ-fibers, and for PR-A in neuronal cytoplasm and nucleus, and in satellite glial cells. The number of progesterone immunoreactive cells in the TG was higher in female compared to male. The PR mRNA was expressed in both hypothalamus and TG ; however, the PR expression level was significantly higher in the hypothalamus. Progesterone did not induce a significant change neither in basal level nor upon stimulated release of CGRP from dura mater or TG in male or female rats when compared to the vehicle control. However, pre-treated with 10 μM progesterone enhanced capsaicin induced CGRP release observed in the dura mater of male rats. In male basilar arteries, progesterone significantly amplified the dilation in response to capsaicin.

**Conclusion:** In conclusion, these results highlight the potential for progesterone to modulate sensory neurotransmission and vascular responses in a complex manner, with effects varying by tissue type, sex, and the nature of the stimulus. Further investigations are needed to elucidate these findings.

## LP035 No wearing-off effect of erenumab or fremanezumab for chronic migraine prevention: a single-center, real world, observational study

### A. M. Florescu, L. V. Lannov, S. Younis, C. K. Cullum, B. A. Chaudhry, T. P. Do, F. M. Amin

#### Danish Headache Center, Neurology, 2600, Denmark

##### **Correspondence:** S. Younis

*The Journal of Headache and Pain 2024*, **25(Suppl 1):**LP035

**Objective:** The implementation of antibody treatment has increased. This raises the question of whether the effect of monthly administration lasts the entire month or gradually wears off until next dosing requiring more frequent administration.

The present study investigates the wearing-off effect in adults with chronic migraine treated with erenumab or fremanezumab.

**Methods:** Pre-collected headache diaries from chronic migraine patients treated with 140 mg erenumab or 225 mg fremanezumab were used. Wearing-off effect was investigated in two consecutive treatment months to ensure that the effect was not due to random causes. Consistent wearing-off was defined as increase of ≥2 weekly migraine days in the last week compared to the second week over two consecutive 4-week treatment periods. Primary endpoint was wearing-off in total population. Secondary endpoints were (1) difference in wearing-off in patients treated with erenumab vs fremanezumab; (2) wearing-off in patients with ≥30% reduction in monthly migraine days vs baseline.

**Results:** 100 patients (erenumab 59, fremanezumab 41) were included, hereof 62 (46 erenumab, 28 fremanezumab) had consistent ≥30% treatment response. There was no consistent wearing-off over 2 consecutive months from week 2 to week 4 (3.04%, *p*=0.558). There was no wearing-off within erenumab (*p*=0.194) or fremanezumab (*p*=0.581) groups. Among ≥30% treatment responders, there was no consistent wearing-off over 2 consecutive months (2.6%, *p*=0.573).

**Conclusion:** No wearing-off in treatment responders was in alignment with premarketing data from placebo-controlled phase III studies. The results suggest that patients should be informed upfront that no wearing-off effect is expected, as anxiety for attacks at end of month may induce migraine attacks.

## LP036 Prophylactic polytherapy for chronic migraine: a prospective observational evaluating the potential efficacy resulting from the addition of atogepant to OnabotulinumtoxinA

### J. Rothrock, A. Koutsandreas, L. Armstead

#### Inova Health/University of Virginia School of Medicine, Neurology, Fairfax, United States

##### **Correspondence:** J. Rothrock

*The Journal of Headache and Pain 2024*, **25(Suppl 1):**LP036

**Objective:** To determine whether the addition of atogepant 60 mg daily to ongoing treatment with BotoxA in partial positive responders to injection therapy is safe, well-tolerated and potentially synergistic in terms of treatment efficacy

**Methods:** To CM patients who had been receiving serial BotoxA injection therapy for at > 1 year and had experienced a partial positive response to that treatment we offered the option of adding atogepant 60 mg daily.

We assessed mean monthly migraine days and mean "migraine burden index (MBI)" (a calculation based on headache frequency and severity) for the 3 months preceding initiation of treatment with atogepant. For all patients who remained on treatment with BotoxA and atogepant we reassessed the same variables 12 weeks following initiation of treatment with atogepant.

A patient was considered to be a positive responder to polytherapy with BotoxA and atogepant if he/she completed the 12 week treatment period and reported a 50% or greater reduction in mean monthly migraine days, MBI or both relative to their pre-atogepant status (primary endpoint).

**Results:** 314 patients with CM met our criterion for "partial positive responder", and 234 (74.5%) elected to continue BotoxA and add atogepant. No participating subject had taken any other prophylactic medication for at least 3 months prior to study entry.

211 (90%) completed the 12 week treatment period. 4 patients (2%) discontinued atogepant consequent to adverse events, none of which were clinically serious

Of the 211 patients who completed the treatment period, 132 (63%) achieved the primary treatment endpoint of a 50% or greater reduction in or MBI experienced over the 12 week treatment period relative to their pre-atogepant status. As for secondary variables, mean monthly headache days declined by 6.6 days, mean functionally incapacitating headache days by 2.7 days, mean monthly days of symptomatic medication use by 5.4 days, MIDAS score by 21.6 and MBI score by 16.6.

**Conclusion:** In this open-label observational trial, adding atogepant 60 mg to the prophylactic regimen of CM patients receiving BotoxA was potentially synergistic in almost two-thirds of patients who completed the treatment phase.

## LP037 A chronic cluster headache registry in Madrid: series of 88 cases

### J. A. Membrilla^1^, M. L. Cuadrado^2^, N. Gonzalez-Garcia^2^, J. Porta-Etessam^2^, A. Sanchez-Soblechero^3^, A. Lozano-Ros^3^, A. Gonzalez-Martinez^4^, A. B. Gago-Veiga^4^, S. Quintas^4^, J. S. Rodriguez-Vico^5^, A. Jaimes^5^, L. Llorente-Ayuso^6^, J. Roa^5^, C. Estebas^7^, J. Diaz-de-Teran^7^

#### ^1^Hospital Universitari Francesc de Borja, Gandia, Spain; ^2^Hospital Universitario Clínico San Carlos, Madrid, Spain; ^3^Hospital Universitario Gregorio Marañón, Madrid, Spain; ^4^Hospital Universitario La Princesa, Madrid, Spain; ^5^Hospital Universitario Fundación Jiménez Díaz, Madrid, Spain; ^6^Hospital Universitario Infanta Leonor, Madrid, Spain; ^7^Hospital Universitario La Paz, Madrid, Spain

##### **Correspondence:** J. A. Membrilla

*The Journal of Headache and Pain 2024*, **25(Suppl 1):**LP037

**Objective:** Chronic cluster headache (CCH) is relatively uncommon, for that reason large series are scarce. Our aim is to describe the clinical characteristics of patients with CCH.

**Methods:** This is a cross-sectional study performed through the review of clinical records of patients with CCH from six hospitals in Madrid. Epidemiological, clinical and treatment-related variables and their outcomes were described.

**Results:** Eighty-eight patients with CRC were included. European Headache Federation refractory CCH criteria were met in 60/88 (68.2%). Mean age at debut was 33.6 (SD 12.9), with a mean diagnostic delay of 4.2 years (SD 6.3). Verapamil, lithium and topiramate were used in 87/88 (98.9%), 37/88 (42.1%) and 74/88 (84.1%) and discontinued in 36/87 (41.4%), 37/52 (71.2%) and 47/74 (63.5%), respectively. OnabotulinumtoxinA and galcanezumab were initiated in 68/88 (77.3%) and 5/88 (5.7%), discontinued in 36/68 (52.9%) and 3/5 (60.0%). Occipital nerve stimulators (ONS) were implanted in 26/88 (29.6%), 13/26 (50.0%) were withdrawn or turned off. Most treatment discontinuations were for ineffectiveness. At the time of collection, 53/88 (60.2%) had poor clinical status (defined as at least three attacks per week with impact on quality of life). OnabotulinumtoxinA and ONS were the treatments most associated with good clinical status in refractory CCH.

**Conclusion:** CCH is a poor prognostic disease, with refractoriness criteria being met in more than half. OnabotulinumtoxinA and ONS could be the best treatments to offer in these cases.

## LP038 Clinical characteristics related to refractoriness in chronic cluster headache

### J. A. Membrilla^1^, M. L. Cuadrado^2^, N. Gonzalez-Garcia^2^, J. Porta-Etessam^2^, A. Sanchez-Soblechero^3^, A. Lozano-Ros^3^, A. Gonzalez-Martinez^4^, A. B. Gago-Veiga^4^, S. Quintas^4^, J. S. Rodriguez-Vico^5^, A. Jaimes^5^, L. Llorente-Ayuso^6^, J. Roa^5^, C. Estebas^7^, J. Diaz-de-Teran^7^

#### ^1^Hospital Universitari Francesc de Borja, Gandia, Spain; ^2^Hospital Universitario Clínico San Carlos, Madrid, Spain; ^3^Hospital Universitario Gregorio Marañón, Madrid, Spain; ^4^Hospital Universitario La Princesa, Madrid, Spain; ^5^Hospital Universitario Fundación Jiménez Díaz, Madrid, Spain; ^6^Hospital Universitario Infanta Leonor, Madrid, Spain; ^7^Hospital Universitario La Paz, Madrid, Spain

##### **Correspondence:** J. A. Membrilla

*The Journal of Headache and Pain 2024*, **25(Suppl 1):**LP038

**Objective:** The diagnostic criteria for refractory chronic cluster headache (CCH) have been defined by the European Headache Federation. It is not known whether clinical features may vary between refractory and non-refractory CCH.

**Methods:** This is a cross-sectional study performed through the review of clinical records of patients with CRC from six hospitals in Madrid. Epidemiological and clinical variables of patients with refractory and non-refractory CCH were compared.

**Results:** From a series of 88 CCH, 60 (68.2%) met criteria for refractory. There were no differences with respect to non-refractory CCH in terms of sex, psychiatric history, comorbidity with another headache or other chronic pain syndrome. The age of debut was similar in both groups (33.6 in refractory and 33.5 in non-refractory, *p*=0.245), but the diagnostic delay was greater in refractory (4.6 vs 3.2 years, *p*=0.017), also the use of opioids was greater (38.6% vs 14.3%, *p*=0.022). Refractory patients more frequently presented absence of remission periods with respect to non-refractory patients (72.9% vs 42.9%, *p*=0.007), who more frequently presented remission periods of less than 3 months. The presence of seasonal exacerbations was also less frequent in refractories (32.1% vs 55.6%, *p*=0.041).

**Conclusion:** Patients with refractory CCH have a longer diagnostic delay, higher opioid use and more frequently a course without periods of remission or seasonal fluctuations. The identification of these characteristics may be of interest when trying to predict the evolution of CCH.

## LP039 Randomized, open-label, clinical trial for evaluating the use of extended-release paracetamol as a prophylactic for fasting headache during the first week of Ramadan

### O. Almohammed^1^, N. Albishi^1^, A. Alwhaibi^1^, F. Alasmari^2^, F. Almutairi^2^, M. Assiri^2^, A. Albilali^3^, S. Alsanea^2^

#### ^1^King Saud University, College of Pharmacy - Clinical Pharmacy, Riyadh, Saudi Arabia; ^2^King Saud University, College of Pharmacy - Pharmacology, Riyadh, Saudi Arabia; ^3^King Saud University, College of Medicine - Neurology, Riyadh, Saudi Arabia

##### **Correspondence:** O. Almohammed

*The Journal of Headache and Pain 2024*, **25(Suppl 1):**LP039

**Objective:** Fasting headache mainly occurs during the first few days of the month of Ramadan and the treatment is challenging due to fasting. This study aims to evaluate the protective effect of extended-release paracetamol against fasting headache.

**Methods:** In our randomized, open-label, clinical trial we investigated the efficacy of using extended-release paracetamol with a daily dose of 1330 mg to prevent or delay headache. Adults aged 18 years and older were eligible to participate in the study by fasting around 13.5 hours during the first week of Ramadan (2023 AD). Participants in treatment and control arms were followed to investigate the incidence, severity, and timing of headache through self-reporting using a standardized headache diary scale. The study was registered with the Saudi Food and Drug Authority (SFDA) as phase 3 clinical trial (SCTR No. 22122102).

**Results:** We enrolled and randomized 238 participants, of which 181 followed the protocol for at least the first day and were included in the analysis; 85 in the paracetamol treatment group and 96 in the control group. The overall number of headache episodes was 33.2% on day 1 and had decreased to 11.7% on day 7. Initially, there was no significant difference in the incidence of headache between the treatment and control groups. On day three, however, the treatment group had a significantly fewer headache episodes over the control group (5.3% vs. 16.3%; *p*-value=0.026). Overall, the severity of headache was mainly mild to moderate, and the timing of headache was similar in both groups. No side effects have been reported during the study period.

**Conclusion:** There was no significant difference in the incidence of headache between the treatment and control groups. Additional studies are required to overcome the fasting headache during the first week of Ramadan.

**Fig. 1 (Abstract LP039):**
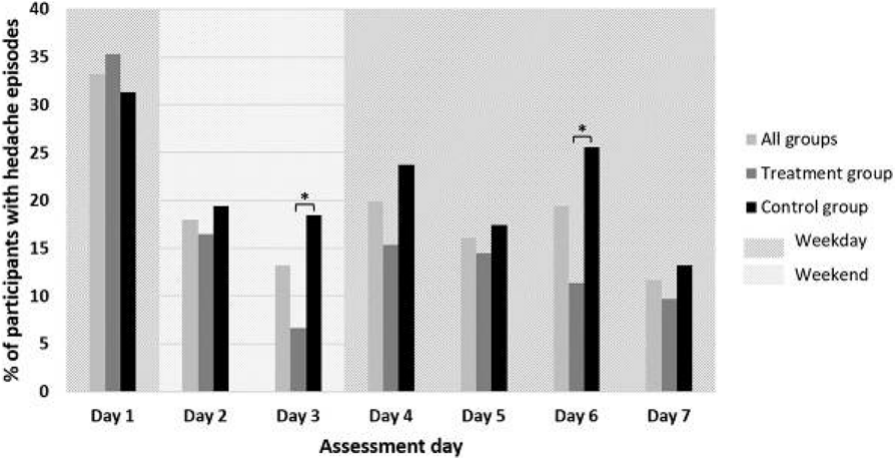
See text for description

## LP040 The combined impact of migraine and pregnancy-induced hypertension on long-term risk of premature myocardial infarction and stroke

### C. Hvitfeldt Fuglsang^1,2^, L. Pedersen^1,2^, M. Schmidt^1,2,3^, J. Vandenbroucke^2,4,5^, H. E. Bøtker^3^, H. Toft Sørensen^1,2^

#### ^1^Aarhus University Hospital, Department of Clinical Epidemiology, Aarhus N, Denmark; ^2^Aarhus University, Department of Clinical Medicine, Aarhus, Denmark; ^3^Aarhus University Hospital, Department of Cardiology, Aarhus, Denmark; ^4^Leiden University, Medical Center, Leiden, Netherlands; ^5^University of London, London School of Hygiene and Tropical Medicine, London, United Kingdom

##### **Correspondence:** C. Hvitfeldt Fuglsang

*The Journal of Headache and Pain 2024*, **25(Suppl 1):**LP040

**Objective:** The aim of this study was to examine the combined impact of migraine and PIH on risk of premature (age ≤60 years) major adverse cardiovascular and cerebrovascular events (MACCE), a composite endpoint consisting of fatal and non-fatal myocardial infarction and stroke.

**Methods:** We conducted a population-based cohort study in Denmark (1996–2018) among women who had delivered at least one child. This population was divided into four cohorts: women with neither migraine nor PIH, women with migraine, women with PIH, and women with both migraine and PIH. As a measure of absolute risk, we computed the 20-year cumulative incidence of premature MACCE, treating death by other causes than myocardial infarction and stroke as a competing risk. Cox regression was used to compute 20-year adjusted hazard ratios (HRs) of premature MACCE. Women with neither migraine nor PIH served as the comparison cohort.

**Results:** The 20-year absolute risk of premature MACCE was 1.3% (95% CI: 1.2%; 1.3%) for women without migraine and without PIH (*n*=1,288,541); 2.2% (95% CI: 2.0%; 2.4%) for women with migraine (*n*=54,827); 2.8% (95% CI: 2.6%; 3.1%) for women with PIH (*n*=49,008); and 3.1% (95% CI: 2.1%; 4.4%) for women with both migraine and PIH (*n*=3140). The adjusted HR of premature MACCE was 1.66 (95% confidence interval [CI]: 1.50; 1.84) for women with migraine; 2.76 (95% CI: 2.52; 3.03) for women with PIH; and 2.41 (95% CI: 1.61; 3.61) for women with both migraine and PIH.

**Conclusion:** Migraine and PIH separately increased the risk of premature MACCE, while the risk of premature MACCE among women who had both migraine and PIH was similar to that among women with PIH only.

## LP041 Cerebral venous sinus thrombosis complicated with raised intracranial pressure

### S. Patel, C. Southall, P. Suganthan

#### Armadale Health Service, General Medicine, Perth, Australia

##### **Correspondence:** S. Patel; C. Southall

*The Journal of Headache and Pain 2024*, **25(Suppl 1):**LP014

**Objective:** We present the case of a patient presenting with an acute cerebral venous sinus thrombosis (CVT), progressively complicated by raised intracranial pressure (ICP) despite commencement on treatment.

**Methods:** An obese woman in her 40s presented with frontal headache associated with vomiting and photophobia. She has been on the oral contraceptive pill long-term. Her first physical examination, including fundoscopy, was unremarkable. Computerised Tomography brain scan with venogram revealed a left transverse sinus thrombosis. Her autoimmune and thrombotic screen were negative. Following commencement on therapeutic low-molecular weight heparin, the patient reported persistent severe headache with photophobia.

**Results:** Magnetic Resonance venogram demonstrated a more extensive thrombus with features suggestive of raised intracranial pressure. Repeat fundoscopy revealed bilateral papilledema. Her symptoms gradually improved on acetazolamide and she was discharged on the oral anticoagulant rivaroxaban.

**Conclusion:** This case shows the importance of considering CVT as a differential in patients presenting with non-specific headaches, especially in patients with multiple risk factors. Obesity and oral contraceptive pills synergistically increase the risk of CVT; therefore, clinicians should have a lower threshold and consider a diagnosis of CVT earlier in this cohort. We have also showed that patients with confirmed CVT who do not improve symptomatically post-commencement of treatment, MRI/MR venogram is useful in finding secondary complications and assessing extent of parenchymal damage.

*Disclosure statement*: Informed consent to publish this case study and its potentially identifiable information of the patient was obtained from the individual involved. The patient gave explicit permission for the publication of this case report, including any relevant clinical details.

**Fig. 1 (Abstract LP041):**
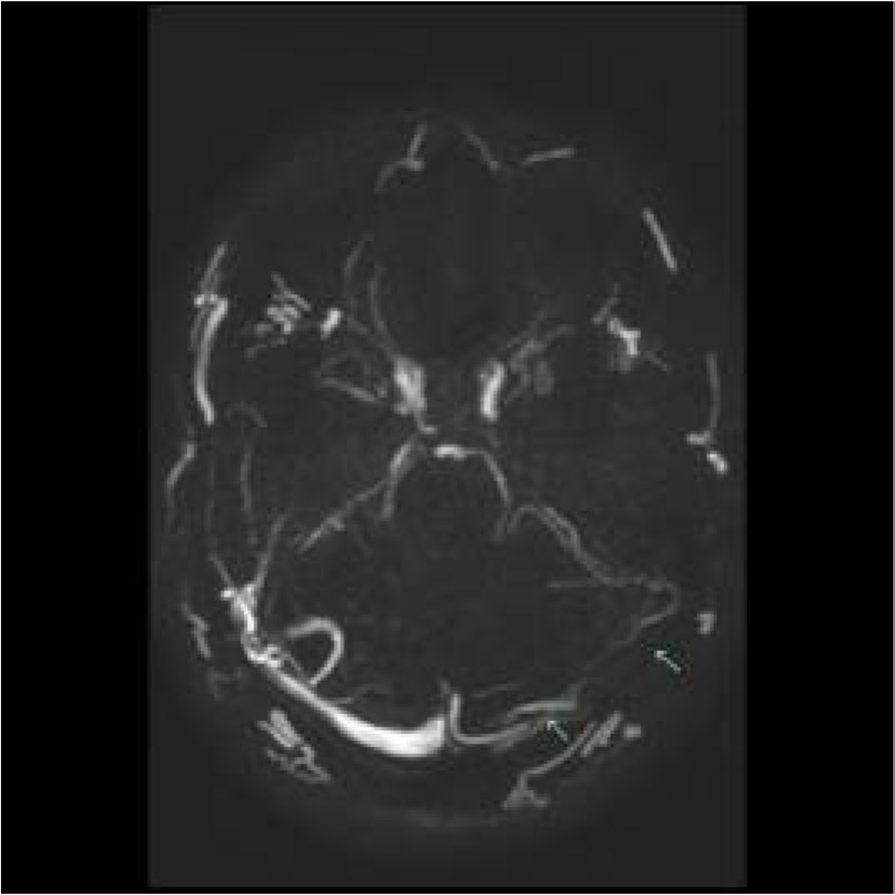
See text for description

**Fig. 2 (Abstract LP041):**
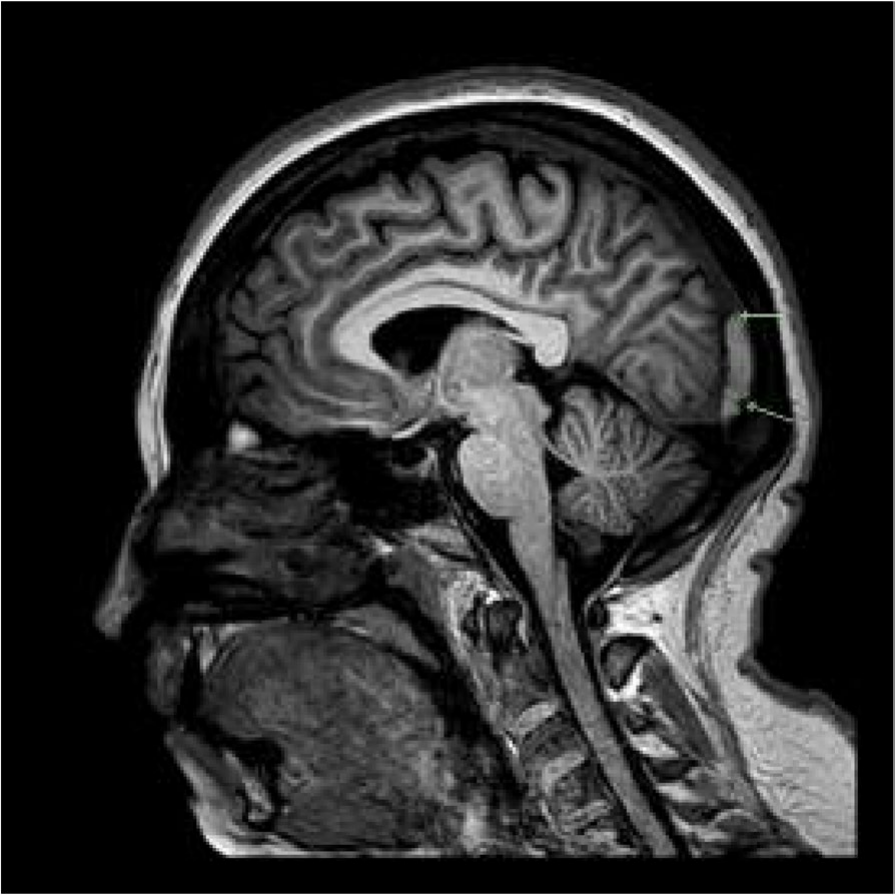
See text for description

## LP042 Premonitory symptoms in migraine: A REFORM study

### J. Thuraiaiyah^1^, H. Ashina^1^, R. H. Christensen^1^, H. Al-Khazali^1^, A. Wiggers^1^, F. M. Amin^1^, T. J. Steiner^2^, M. Ashina^1^

#### ^1^Copenhagen University Hospital - Rigshospitalet, Department of Neurology, Danish Headache Center, Glostrup, Denmark; ^2^Imperial College London, Division of Brain Sciences, London, United Kingdom

##### **Correspondence:** J. Thuraiaiyah

*The Journal of Headache and Pain 2024*, **25(Suppl 1):**LP042

**Objective:** We aimed to establish the proportion of patients reporting premonitory symptoms based on two enquiry methods. Additionally, we investigated the impact of premonitory symptoms on disease burden using Headache Impact Test (HIT-6), Migraine Disability Assessment (MIDAS) and World Health Organization Disability Assessment 2.0 (WHODAS 2.0).

**Methods:** In a cross-sectional study, patients with migraine from a tertiary clinic were asked to list premonitory symptoms (experienced within 2 to 48 hours before headache or aura onset). Firstly, they were asked to list any symptom, followed by a checklist of 17 items. Clinical characteristics were elicited through a semi-structured interview. HIT-6, MIDAS and WHODAS were assessed using electronic questionnaires.

**Results:** We included 632 individuals with migraine. Prompted enquiry resulted in a substantially greater proportion of patients reporting premonitory symptoms compared to the unprompted enquiry (69.9% vs. 43.0%; *P<0.001*), and a higher number of premonitory symptoms were reported with the promopted methods compared to unprompted (medians 2 [IQR 0-6] vs 1 [IQR 0-1]; *P*<0.001). Number of reported premonitory symptoms and measures of disease-attributed burden correlated weakly for HIT-6 (*ρ* = 0.14; *P*<0.001) and WHODAS scores (*ρ* = 0.09; *P*=0.041), but not for MIDAS score (*ρ* = -0.05; *P* = 0.24).

**Conclusion:** The use of a standardized and optimized method for assessing premonitory symptoms is necessary to estimate their probability and to understand whether and how they contribute to disease burden. While the impact of premonitory symptoms on migraine-attributed burden has previously been overlooked, our findings suggest it is of less importance, relative to the impact of headache, both in clinical practice and to public health. This needs verification in population-based studies.

## LP043 Migraine headache phase prediction using machine learning based on the Migrebot E-diary: prospective collection and analysis of more than 50 parameters 3 times a day

### K. Skorobogatykh^1^, J. Azimova^1,2^, O. Cherednichenko^3^, N. Vashchenko^1^, D. Korobkova^1^, E. Mamkhegov^1^

#### ^1^University Headache Clinic, Moscow, Russian Federation; ^2^Institute of General Pathology and Pathophysiology, Moscow, Russian Federation; ^3^Moscow Institute of Physics and Technology, Moscow, Russian Federation

##### **Correspondence:** K. Skorobogatykh

*The Journal of Headache and Pain 2024*, **25(Suppl 1):**LP043

**Objective:** The aim of our study was to prospectively evaluate the symptoms of the premonitory phase in order to find predictors of migraine attacks using a machine learning approach

**Methods: **This was a prospective observational cohort study. The Migrebot E-diary database was used to select subjects with migraine and headache frequency 3-8 days per month. Patients completed a special version of the diary (ProdromaBot) to assess the premonitory phase. Participants were required to complete three time points (TP) each day (9:00, 15:00 and 21:00). At each TP, the participant answered 51 questions about well-being, potential triggers, premonitory symptoms, and a headache. We used logistic regression,random forest, SVM, gradient boosting (XGBoost),KNN as baseline models and LightGBM as final model to find predictors of attack. Each model was trained on a training dataset and evaluated using cross-validation techniques to ensure robustness and mitigate over-fitting. Model performance metrics such as accuracy, precision, recall and F1-score were calculated to show the advantages of each approach. LightGBM showed the best performance so far in terms of the listed metrics

**Results: **The study included a total of 667 subjects. The total number of diary days was 45111, number of TPs with new headache episodes was 18614, total number of valid TPs with the new headache that preceded or followed the fully completed TP was 4308 and it was selected for further analysis. To test the performance of the model, we used a validation dataset that was not included in the original training set. Thus, we had 6621 premonitory TPs for the validation set. Using SHAP analysis through bee swarm plots for feature importance methods, we found that the most common premonitory symptoms were nausea, scalp sensitivity, neck pain, yawning, and frequent urination. The machine learning approach was able to predict the new headache episode with an avearage **75% sensitivity,92% specificity** and 91% for ROC-AUC for the LightGBM boosting algorithm

**Conclusion:** ProdromaBot and a certain set of premonitory symptoms are reliable instruments to prospectively gather clinical information and predict migraine attacks with good sensitivity

**Table 1 (Abstract LP043):**
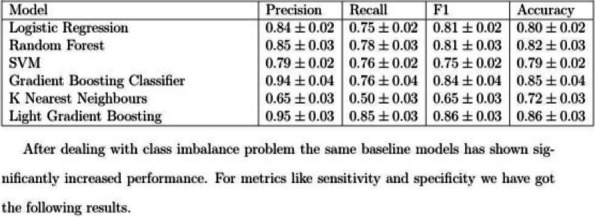
Baseline classification report results for balanced dataset

**Table 2 (Abstract LP043):**
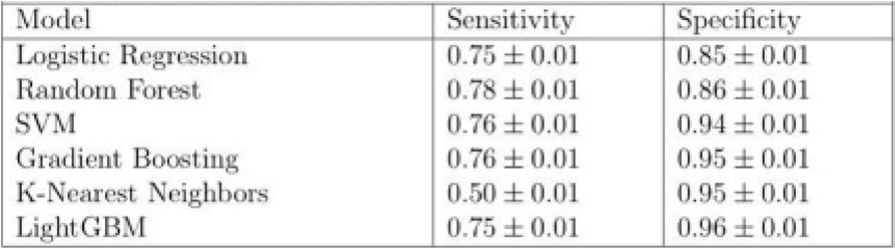
Classification results with sensitivity, specificity

**Fig. 1 (Abstract LP043):**
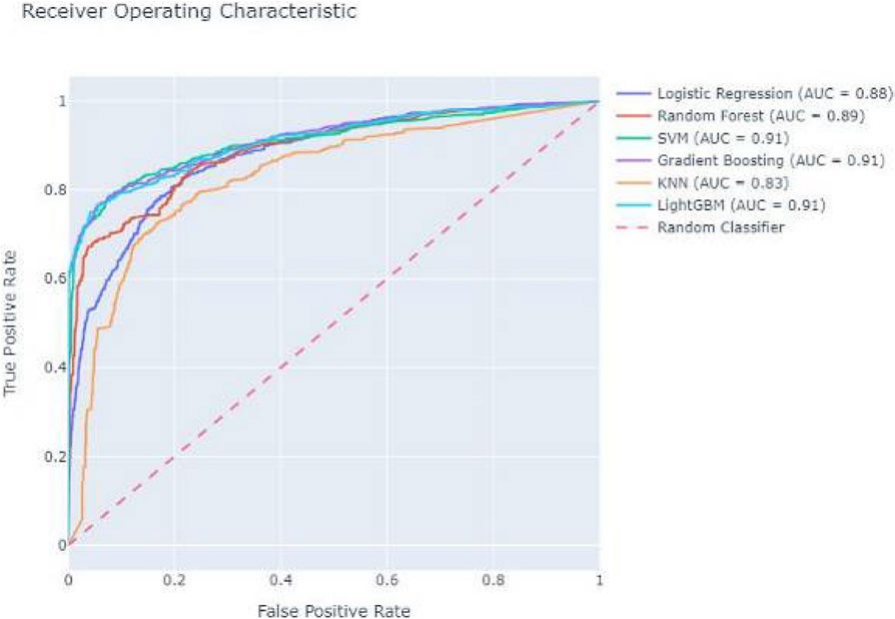
See text for description

**Fig. 2 (Abstract LP043):**
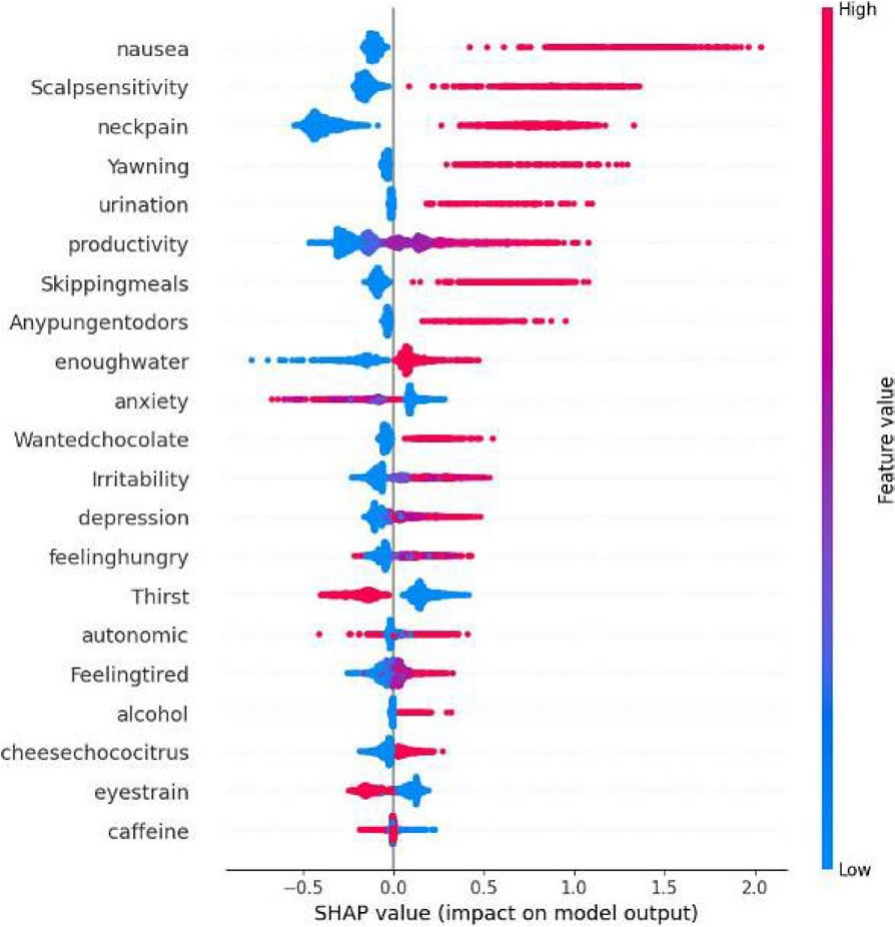
See text for description

## LP044 Vestibular migraine is a real entity far from a form of episodic migraine?

### R. Ghouri^1^, E. Uludüz^2^, N. Öksüz^1^, S. Eyüpoğlu^3^, D. Uludüz^3,4^, A. A. Özdemir^5^, A. Özge^1^

#### ^1^Mersin University School of Medicine, Neurology, Mersin, Turkey; ^2^Koç University School of Medicine, Istanbul, Turkey; ^3^Brain 360 Integrative Center, Neurology, Istanbul, Turkey; ^4^Cerrahpaşa University School of Medicine, Neurology, Istanbul, Turkey; ^5^Mersin University School of Medicine, Medical Education, Mersin, Turkey

##### **Correspondence:** R. Ghouri

*The Journal of Headache and Pain 2024*, **25(Suppl 1):**LP044

**Objective:** Migraine is a complex neurological disorder that can manifest in various forms, including vestibular migraine (VM). VM manifests as episodes of sudden or positional vertigo lasting from seconds to days, and these episodes are accompanied by symptoms commonly associated with migraines. Since headaches are frequently absent during these acute attacks, a comprehensive patient history is necessary to identify other migraine-related characteristics. In this study, we planned to investigate the headache characteristics and accompanying comorbidities of patients with episodic migraine (EM), excluding VM, and patients with VM.

**Methods:** A retrospective analysis was conducted utilizing the comprehensive Mersin University headache database and Brain 360 Integrative center database, which encompassed a total of 334 patients meeting the diagnostic criteria for EM and VM according to the International Classification of Headache Disorders, 3rd edition (ICHD-3). These patients were closely monitored between the years 2021 and 2023. Among the study group, 248 individuals were with EM and the rest with VM. Migrainous features such as throbbing pattern, photophobia, phonophobia, nausea, vomiting and accompanying comorbidities were questioned and analyzed accordingly.

**Results:** Patients with EM exhibited a higher likelihood of experiencing migraine-associated symptoms, including nausea, photophobia and phonophobia (*p*<0.05).

**Conclusiun:** In our study, migraine-like features and comorbidities were generally more common in the EM group and showed different pattern from VM sufferers. It was interesting that motion sickness and vomiting rates did not show a significant difference between the groups. Our results created a questioning real pattern of VM unrestricted to current diagnostic criteria and an emerging topic merit to more comprehensive longitudinal researches.

## LP045 Monoclonal antibodies against calcitonin gene-related peptide for the prophylaxis of migraine with aura

### B. K. Kim^1^, H. C. Lee^1^, S. Cho^2^

#### ^1^Nowon Eulji Medical Center, Eulji University School of Medicine, Department of Neurology, Seoul, South Korea; ^2^Uijeongbu Eulji Medical Center, Eulji University School of Medicine, Department of Neurology, Uijeongbu, South Korea

##### **Correspondence:** B. K. Kim

*The Journal of Headache and Pain 2024*, **25(Suppl 1):**LP045

**Objective:** Monoclonal antibodies to CGRP or its receptor (anti-CGRP mAbs) are a novel class of migraine-specific prophylaxis. Theoretically, the high molecular weight of anti-CGRP mAbs prevents them from crossing the blood-brain barrier and does not inhibit cortical spreading depression, which is the underlying mechanism of a migraine aura. Furthermore, no randomized controlled trials have evaluated their efficacy in migraine with aura (MA), which occurs in up to one-thirds of migraine patients. Here, we prospectively evaluated the changes in days with aura after 3 months of anti-CGRP mAb treatment.

**Methods:** Patients with migraine according to ICHD-3 who had been treated with anti-CGRP mAbs for 3 months were included in the study. Patients were included if they had at least one aura attack per month. Data were collected at baseline and at the third month of treatment. The efficacy of anti-CGRP mAbs in MA was compared with that in migraine without aura (MO). The number of headache and MA days was assessed using a headache diary.

**Results:** Of the 332 patients with migraine who were treated with anti-CGRP mAbs (galcanezumab or frenezumab), 26 had MA. All patients reported visual aura and two of them also had sensory and aphasic aura. The mean age of the patients was 33.7 ± 13.0 years and 69.2% were female. The mean age at the onset of the MA was 24.8 ± 13.3 years. At month 3, 65.4% and 42.3% of patients achieved at least a 50% and 75% reduction in monthly MA days, respectively, which was not statistically different from the monthly headache days in the MO group (75.2% and 46.4%). No serious adverse events were observed regardless of the presence of MA.

**Conclusion:** This study supports that the efficacy of anti-CGRP mAbs is not limited to the incidence of headache, but is also effective in MA attacks that precede headache.

## LP046 Identification of trigger factors of migraine and other primary headaches among migraine sufferers in Azerbaijan population

### I. Azizova^1,2^, R. Hasanov^3^

#### ^1^Neurology Clinic "New Medical Technologies", Baku, Azerbaijan; ^2^Medipoint, Baku, Azerbaijan; ^3^Azerbaijan State Advanced Training Institute for Doctors named after A. Aliyev, Department of Neurology and Clinical Neurophysiology, Baku, Azerbaijan

##### **Correspondence:** I. Azizova

*The Journal of Headache and Pain 2024*, **25(Suppl 1):**LP046

**Objective:** Migraine is a debilitating neurological disorder associated with significant disability. However, its diagnosis can be complicated when coexisting with other types of headaches. Neurologists often focus solely on migraine characteristics and may overlook concurrent headaches. This study aimed to distinguish between triggers that specifically provoke migraines and those that induce other types of headaches among migraine sufferers in Azerbaijan.

**Methods:** We enrolled 144 migraine patients from July 1, 2022, to December 30, 2022. Participants were asked to identify their headache triggers and specify whether each trigger provoked their migraine or another headache type. Common triggers examined included stress, sleep disturbances, exercise, fatigue, hormonal changes, weather fluctuations, sensory stimuli, dietary factors, and various others.

**Results:** The results revealed that stress (68.1%), missed meals (46.5%), traveling (40.1%), sleep disturbances (45.8%), and loud noise (45.1%) were the most frequentmigraine triggers. Cold weather (52.1%) was a shared trigger between tension-type headache and migraine. Surprisingly, most patients experienced more than two triggers, with only a small percentage having a single trigger before a migraine attack. 27.1% of migraineurs also had other primary headache disorders such as primary stabbing headache, primary cough headache, cold-stimulus headache, primary exercise headache, and external traction headache, often sharing triggers with migraines.

**Conclusion:** In summary, patients often conflate triggers for their migraines with those of other headache types, likely due to the debilitating nature of migraines. Thorough examination and emphasis on differentiating headache types are essential for accurate diagnosis and treatment. Our findings not only identified migraine-associated triggers but also revealed misdiagnosed triggers for other co-occurring headache types. This study underscores the importance of individualized care and a nuanced understanding of headache triggers in managing migraine and related conditions.

## LP048 Human RAMP1 (hRAMP1) overexpressing mice are resistant to migraine therapies for motion sensitivity: a mouse model of vestibular migraine

### S. Rahman, A. Luebke

#### University of Rochester, Rochester, NY, United States

##### **Correspondence:** A. Luebke

*The Journal of Headache and Pain 2024*, **25(Suppl 1):**LP048

**Objective:** Increased motion sensitivity correlates with the severity of vestibular migraine symptoms. Systemic CGRP has previously been shown to exacerbate motion-induced nausea in wild-type C57B6/J mice and postural sway in female mice. Olcegepant, a CGRP receptor antagonist, can reverse both effects. In this study, nestin/hRAMP1, a mouse model with elevated human RAMP1 expression that enhances CGRP signaling in the nervous system, was used to investigate motion-induced nausea and static imbalance.

**Methods:** As behavioral surrogates, we performed motion-induced thermoregulation and postural sway center of pressure (CoP) experiments in male and female nestin/hRAMP1 mice and compared them to unaffected littermates. Mice were evaluated in these assays following intraperitoneal (IP) injections of i) vehicle control, ii) CGRP (0.1 mg/kg), or iii) CGRP (0.1 mg/kg) co-administered with either olcegepant (1.0 mg/kg CGRP receptor antagonist) or rizatriptan (1.0 mg/kg selective serotonin receptor agonist).

**Results:** We show that overexpression of hRAMP1 in the nervous system causes increased postural sway in both female and male mice at baseline and that systemic CGRP has no additive effect. We also show that in these RAMP1-overexpressing mice, olcegepant no longer rescues the increased postural sway and motion-induced nausea.

**Conclusion:** This study suggests that hypersensitivity to CGRP may model the clinical symptoms of vestibular migraine. This work was supported by R01 DC017261 (AEL).

## LP049 Open label experience of repeated Onabotulinum Toxin A injections towards the sphenopalatine ganglion in patients with chronic cluster headache and chronic migraine

### L. Simmonds^1^, I. Aschehoug^2^, S. Hara^2,3^, T. Meisingset^2,4,5^, M. Matharu^1,4^, E. Tronvik^2,4,5^, D. Fossum Bratbak^2,3^

#### ^1^Headache and Facial Pain Group, University College London Queen Square Institute of Neurology and National Hospital for Neurology and Neurosurgery, London, United Kingdom; ^2^NTNU Norwegian University of Science and Technology, Neuromedicine, Trondheim, Norway; ^3^St Olav's University Hospital, Neurosurgery, Trondheim, Norway; ^4^Norwegian Headache Research Centre, NorHEAD, Trondheim, Norway; ^5^St Olav's University Hospital, National Advisory Unit on Headaches, Department of Neurology and Clinical Neurophysiology, Trondheim, Norway

##### **Correspondence:** L. Simmonds

*The Journal of Headache and Pain 2024*, **25(Suppl 1):**LP049

**Objective:** A novel technique for the injection of Onabotulinum Toxin A (BTA) towards the sphenopalatine ganglion (SPG) has shown promise in refractory chronic migraine (CM) and chronic cluster headache (CCH). This open label study aims to report the efficacy and safety of this technique in a single centre.

**Methods:** Patients with refractory CM or CCH who had received at least one injection and completed headache diaries were included. Efficacy was defined as ≥50% reduction in moderate-to-severe headache days for CM, or ≥50% reduction in attack frequency for CCH, at weeks five to eight compared to baseline.

**Results:** Twelve patients with CM and 31 with CCH were eligible for efficacy analysis. The 50% response rate for a single injection was 81% for CM and 69% for CCH. Mean reduction of 8.9 MSHDs was seen in the CM group, and 8.9 attacks per month in the CCH group (*p*<0.001). The 50% response rates remained high over four consecutive injections in both groups, ranging between 67-81% in CM and 69-89% in CCH. CCH attack intensity (1-4 scale) reduced by 2.48 (*p*<0.001). For the safety analysis, 261 injections were reviewed, resulting in 123 adverse events (AEs), 93% of which were mild. Common AEs included jaw pain, pain or swelling at the injection site and mild visual disturbance. Four patients had facial weakness, and one was admitted for investigation of this. All AEs resolved, most within 12 weeks.

**Conclusion:** BTA injection towards the SPG is an effective treatment for both resistant CM and CCH. It is likely to remain effective for at least three months. Injections can be repeated with consistently good effect, although criteria for selecting patients most likely to benefit require refinement. The safety profile is acceptable. Incomplete diary monitoring and a relatively small number of patients are limiting factors in this study. A multi-centre, randomised, placebo-controlled trial is now open to patients with refractory CCH which aims to provide higher quality evidence in support of this treatment.

**Fig 1 (Abstract LP049):**
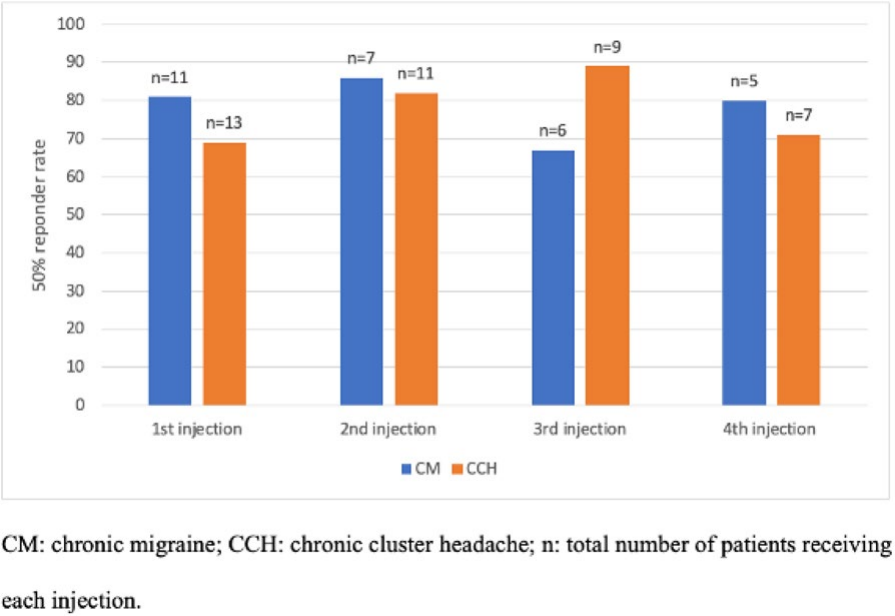
50% response rates at 5-8 weeks after injection, over 4 consencutive injections for chronic migraine and chronic cluster headache patients

**Fig 2 (Abstract LP049):**
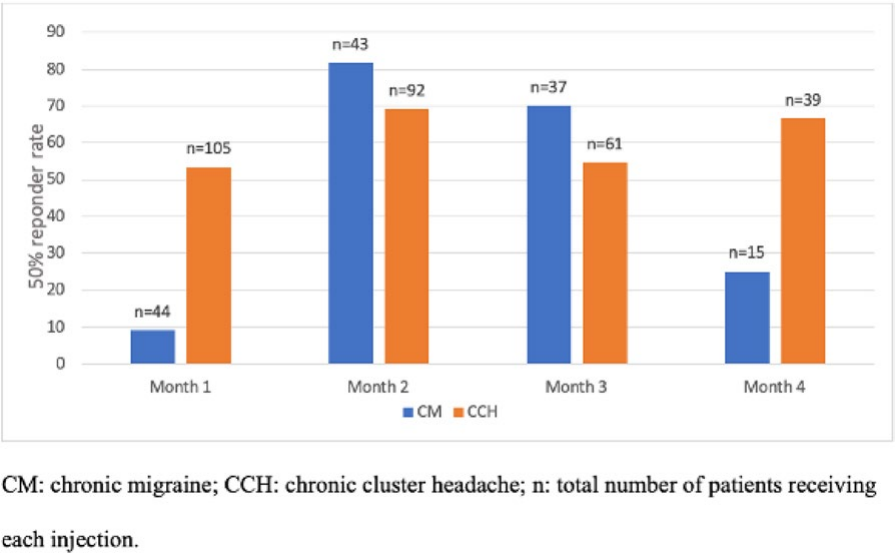
Monthly 50% response rates following a single injection

## LP050 Expression of calcitonin receptor, amylin and CGRP in C1/C2 spinal cord

### T. Rees^1^, Z. Tasma^1^, C. Walker^1^, D. Hay^2^

#### ^1^University of Auckland, School of Biological Sciences, Auckland, New Zealand; ^2^University of Otago, Department of Pharmacology and Toxicology, Dunedin, New Zealand

##### **Correspondence:** T. Rees

*The Journal of Headache and Pain 2024*, **25(Suppl 1):**LP050

**Objective:** The cervical spinal cord (C1-3) receives inputs from the trigeminal ganglia (TG) and dorsal root ganglia (DRG), transmitting sensory information through the ascending pain pathway to the brain. A role for amylin receptors (AMY) and the associated ligands, amylin and calcitonin gene-related peptide (CGRP), in migraine and pain are beginning to emerge. However, the relative distribution of receptor and ligand at relevant anatomical sites is unclear. Hence, it is difficult to determine what contribution AMY receptors and ligands may make to migraine and pain. Therefore, this study aimed to explore the relative distribution of the AMY receptor subunit, the calcitonin receptor (CTR), CGRP and amylin in the upper cervical (C1/2) spinal cord.

**Methods:** Immunohistochemistry was performed with well-characterised and validated antibodies against CTR, CGRP, amylin and neural markers in mouse, rat, and human C1/2 spinal cord.

**Results:** CTR immunoreactivity was observed in the soma of smaller neurons in laminae I-II, as determined by NF200, and in larger neurons in laminae III-V. Colocalisation with calbindin, an interneuron marker, was observed for a subset of CTR immunopositive neurons in laminae I-III. Fibres immunopositive for CGRP and amylin were observed in laminae I-II. Immunoreactivity for CTR, CGRP and amylin was observed together in the soma of a subset of laminae III-V neurons and large motor neuron-like cells in the ventral horn.

**Conclusion:** CGRP and amylin may be present in dorsal horn fibres that project from the TG (CGRP) and DRG (CGRP and amylin) and could activate CTR-based receptors in laminae I-V neurons. These may be interneurons and 2nd-order neurons. This suggests that CTR-based receptors may contribute to the transmission and modulation of sensory information from the peripheral to the central nervous system. In addition, the expression of CTR and ligands in motor neuron-like cells indicate possible involvement in motor function.

## LP051 Reimbursement rules for CGRP mAbs throughout Europe: an overview of the various constraints used

### J. Versijpt^1^, F. Amin^2^, C. Deligianni^3^, R. Gil-Gouveia^4^, P. Martelletti^5^, D. Uludüz^6^, U. Reuter^7^, S. Sacco^8^, M. Sanchez del Rio^9^, A. Maassen van den Brink^10^, C. Lampl^11^

#### ^1^Vrije Universiteit Brussel (VUB), Universitair Ziekenhuis Brussel (UZ Brussel), Department of Neurology, Brussels, Belgium; ^2^Danish Headache Center, Copenhagen University Hospital - Rigshospitalet, Department of Neurology, Copenhagen, Denmark; ^3^Athens Naval Hospital, Department of Neurology, Athens, Greece; ^4^Hospital da Luz Headache Center, Hospital da Luz Lisboa, Neurology Department, Lisbon, Portugal; ^5^Sapienza University, Department of Clinical and Molecular Medicine, Rome, Italy; ^6^Istanbul Cerrahpasa Medical Faculty, Department of Neurology , Istanbul, Turkey; ^7^Charité Universitätsmedizin, Department of Neurology, Berlin, Germany; ^8^University of L´Aquila, Department of Biotechnological and Applied Clinical Sciences, L’Aquila, Italy; ^9^Clinica Universidad de Navarra, Department of Neurology, Madrid, Spain; ^10^Erasmus MC Medical Center, Department of Internal Medicine, Rotterdam, Netherlands; ^11^Konventhospital Barmherzige Brüder Linz, Department of Neurology, Linz, Austria

##### **Correspondence:** J. Versijpt

*The Journal of Headache and Pain 2024*, **25(Suppl 1):**LP051

**Objective:** Although recent guidelines of the European Headache Federation recommend CGRP mAbs as a *first line treatment option* for the prophylactic treatment of migraine, most if not all European countries build in restrictions to limit the number of eligible patients and the ensuing costs. The present study seeks to describe the eligibility criteria that have been implemented in different European countries.

**Methods:** Representatives of European countries were inquired by mail about the current reimbursement policy of CGRP mAbs in their country.

**Results:** Only 16 out of 29 countries inquired provide a reimbursement for CGRP mAbs. Mostly (hospital based) neurologists are allowed to prescribe CGRP mAbs, sometimes also headache or pain specialists. Eligible patients need monthly migraine days varying from at least 4 to sometimes having to fulfill criteria for chronic migraine. In most countries, 3 drug(s) (classes) should be failed before CGRP mAbs are reimbursed. In 3 countries, medication overuse should be addressed before CGRP mAbs can be prescribed. In 4 countries, onabotulinum toxin A must be failed before prescribing CGRP mAbs for chronic migraine. In contrast, in approximately half of the countries, the combination of onabotulinum toxin A and CGRP mAbs is viable and reimbursable. In the majority of countries, an evaluation of treatment efficacy is required after 3 months, confirming at least a 50% reduction in monthly migraine days before extending the treatment period. Subsequent evaluations are generally foreseen on a yearly basis. In 4 countries a mandatory drug holiday (varying between 1 to 3 months) is required after 12-18 months. In most countries, switching between mAbs is allowed.

**Conclusion:** More than half of the countries inquired provide a reimbursement when prescribing CGRP mAbs. Constraints used before CGRP mAbs are reimbursed vary widely across countries and are not always in line with the most recent guidelines from the European Headache Federation concerning their use.

## LP052 Systemic PACAP and CGRP have comparable effects on motion-induced nausea and postural sway measures in preclinical models

### S. Rahman, A. Luebke

#### University of Rochester, Rochester, NY, United States

##### **Correspondence:** A. Luebke

*The Journal of Headache and Pain 2024*, **25(Suppl 1):**LP052

**Objective:** Migraine and vestibular migraine (VM) are disorders associated with increased motion sensitivity leading to symptoms of motion-induced nausea and static imbalance. Antagonism of CGRP signaling is an established therapy, but not all patients respond. In this study, we compared systemic PACAP with systemic CGRP to investigate motion-induced nausea and static imbalance in mice.

**Methods:** As behavioral surrogates, we performed motion-induced nausea and center of pressure (CoP) postural sway experiments in male and female C57B/6 mice. Mice were evaluated in these assays following intraperitoneal (IP) injections of i) vehicle control, ii) CGRP (0.1 mg/kg) (Sigma-Aldrich), or iii) 0.3 mg/kg PACAP-38 (Bachem). These studies were performed in 80 wildtype C57BL/6J (JAX 664) mice (40F/40M). For the motion-induced nausea test, head and tail temperatures were measured with a FLIR E60 IR camera before, during, and after 20 min of orbital rotation (0.75 Hz to 4 cm displacement). For postural sway testing, mice were placed on a force platform and forces and moments were recorded before and after a brief vestibular challenge.

**Results:** We confirmed that in both female and male mice during provocative motion, there is a decrease in head temperature which recovers, and an associated short-lasting tail skin vasodilation . Interestingly, both systemic CGRP and systemic PACAP-38 injections caused similar reductions in head temperature, but there was no associated tail skin vasodilation, suggesting severe motion-induced nausea. In addition, mice showed increased postural sway after either systemic CGRP or PACAP-38 injection.

**Conclusion:** Our finding that systemic CGRP and PACAP have comparable effects on motion-induced nausea and measures of postural sway in mice suggests that PACAP-targeted drugs may be effective in patients who do not respond to CGRP-based therapeutics. This work was supported by R01 DC017261(AEL).

## LP053 Atypical headache in a patient with fibrous dysplasia of the sphenoid bone: a case report

### Y. Cohen, A. A. Ashkenazi

#### Shaare Zedek Medical Center, Neurology, Jerusalem, Israel

##### **Correspondence:** A. A. Ashkenazi

*The Journal of Headache and Pain 2024*, **25(Suppl 1):**LP053

**Objective:** Fibrous dysplasia is a rare benign bone disorder known for its potential to affect craniofacial structures. This report highlights a 25-year-old male who presented with a new daily persistant headache and was found to have craniofacial fibrous dysplasia. Fibrous dysplasia, stemming from presence of activating mutations involving G-nucleotide binding protein alpha subunit (GNAS), results in abnormal fibrous tissue replacing bone. It can occur in various bones, including the skull, occasionally causing pain and deformities.

**Methods:** This is a case report of a patient presented to our outpatient clinic.

**Results:** A 25-year-old male presented to our clinic with new-onset headache of six-week duration. The pain localized to his right periorbital and temporal area. Pain was daily and worsened with eye movements. He had right eye tearing during the pain, but no conjunctival hyperemia or droopy eyelid. There was no history of trauma or systemic illness. Physical examination revealed right periorbital and temporal bone tenderness. Neurological examination was normal. Ophthalmological examination revealed mild decreased visual acuity of the right eye. Head CT and MRI scans showed a lytic lesion in the right sphenoidal wing bone with a ground-glass appearance, extending into adjacent structures and involving the foramen rotundum, findings characteristic of fibrous dysplasia.

**Conclusion:** Although uncommon, fibrous dysplasia should be considered when a localized atypical craniofacial pain is present. We hypothesize that the patient`s headache resulted from the expanding fibrous dysplastic lesion, that involved the foramen rotundum through which the maxillary branch of the trigeminal nerve exits the skull. His visual deficit may have been caused by involvement of the orbit as well.

In conclusion, for atypical headaches in young adults, fibrous dysplasia should be considered, especially when craniofacial tenderness and characteristic radiographic findings are noted. A multidisciplinary approach to management, including surgical intervention when required, can lead to favorable outcomes in such cases

*Disclosure statement*: Informed consent to publish this case study and its potentially identifiable information of the patient was obtained from the individual involved. The patient gave explicit permission for the publication of this case report, including any relevant clinical details.

**Fig. 1 (Abstract LP053):**
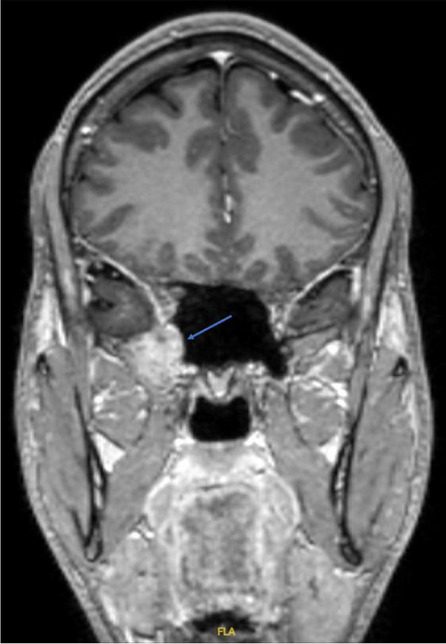
See text for description

**Fig. 2 (Abstract LP053):**
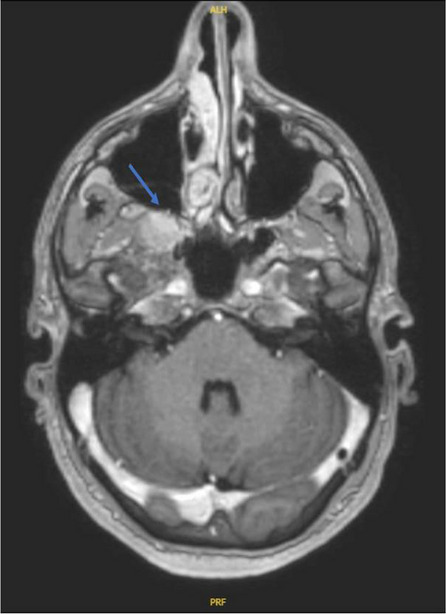
See text for description

## LP054 Impact of primary headache disorders on the quality of life among University students in health profession fields

### D. Mahović^1,2^, L. Jakuš^3^, M. Bracic^4^, M. Horvat Tišlar^3^

#### ^1^University Hospital Centre Zagreb, Department of Neurology, Zagreb, Croatia; ^2^School of Medicine Zagreb, Zagreb, Croatia; ^3^University of Applied Health Sciences, Zagreb, Croatia; ^4^Andrija Štampar Teaching Institute of Public Health, Department of School and Adolescent Medicine, Zagreb, Croatia

##### **Correspondence:** D. Mahović

*The Journal of Headache and Pain 2024*, **25(Suppl 1):**LP054

**Objective:** Primary headache disorders are increasingly prevalent among young adults and have substantial implications for their quality of life and academic or professional achievements. This sub-study was designed to evaluate the impact of primary headache disorders on the quality of life of university students majoring in health profession fields.

**Methods:** A cross-sectional epidemiological study was conducted among university students enrolled in various health profession programs at the University of Applied Health Sciences in Zagreb, Croatia. The Croatian version of the Headache-Attributed Restriction, Disability, Social Handicap, and Impaired Participation (HARDSHIP) questionnaire was employed to determine the prevalence of primary headache cases and to assess their quality of life (measured using the WHOQoL-8 score incorporated into the HARDSHIP questionnaire). The Kruskal-Wallis nonparametric statistical test was utilized to investigate differences among independently sampled groups with respect to a single, non-normally distributed continuous variable.

**Results:** A total of 1350 full-time students participated in this study, comprising 253 (18.7%) males and 1097 (81.3%) females, with an average age of 22 years (M ± SD: 21.9 ± 2.27). In the sample, 525 (38.9%) respondents tested positive for migraine, 481 (35.6%) for tension-type headaches, 223 (16.5%) met the criteria for undifferentiated headaches, and 121 (9%) reported having no headaches. The mean WHOQoL-8 score for migraine was 30.9, for tension-type headaches 32.4, for undifferentiated headaches 31.6, and for no headaches 33.6 (a higher score indicating a better quality of life). Statistically significant differences were observed between the groups (H = 27.3; *p* < 0.01; η^2^ = 0.021).

**Conclusion:** These findings suggest that primary headache disorders can exert a substantial impact on the quality of life of university students in health profession fields.

## LP055 Post hoc analysis of the APPRAISE study: evaluating treatment satisfaction and quality of life improvement in patients with episodic migraine-erenumab vs standard of care

### P. Pozo-Rosich^1^, D. Dolezil^2^, K. Paemeleire^3^, A. Stepien^4^, P. Stude^5^, J. Snellman^6^, M. Arkuszewski^6^, T. Stites^6^, T. Maio-Twofoot^6^, C. Babanrao Pisal^7^, R. Gil-Gouveia^8^

#### ^1^Vall d'Hebron University Hospital, Neurology, Barcelona, Spain; ^2^Headache Center, DADO Medical sro, Prague, Czech Republic; ^3^Ghent University Hospital, Ghent, Belgium; ^4^WIM, Warsaw, Poland; ^5^Praxis Dr. Stude, Bochum, Germany; ^6^Novartis Pharma AG, Basel, Switzerland; ^7^Novartis Healthcare Pvt. Ltd., Hyderabad, India; ^8^Hospital da Luz, Lisban, Portugal

##### **Correspondence:** P. Pozo-Rosich

*The Journal of Headache and Pain 2024*, **25(Suppl 1):**LP055

**Objective:** To compare the effect of erenumab and oral standard of care (SoC) prophylactics on patient-reported outcomes (PROs) in patients with episodic migraine (EM) who completed a 52-week treatment phase on initially assigned treatment.

**Methods:** APPRAISE was a phase 4, global, 12-month study including adult patients with 1 or 2 prior prophylactic treatment failures in the last six months that were randomized (2:1) to receive monthly subcutaneous erenumab (70 mg/140 mg; *n*=413) or daily oral SoC (*n*=208). Dose adjustment and treatment switching to SoC was allowed for both arms to obtain the best possible outcome for patients. This post hoc analysis evaluated the following PROs: PGIC, HIT-6, MIDAS, TSQM-14, and SF-36 after 52 weeks of treatment.

**Results:** A total of 86.9% (359/413) of patients in the erenumab group vs 37.5% (78/208) in the SoC group completed 52 weeks on initially assigned treatment. Among the patients remaining on initial treatment, 88% of erenumab-treated patients vs 50% in the SoC group achieved relevant clinical improvement in PGIC score (5-7) at week 52. Patients treated with erenumab had greater reductions from baseline in disability scores including HIT-6 (−10.1 vs −4.9), MIDAS (−29.2 vs −11.9) and improvement in SF-36 scores (8.5 vs 4.1). The mean change from baseline in total TSQM score (74.4 vs 42.8), domain score for effectiveness (38.0 vs 13.4), and global satisfaction (41.7 vs 10.8), was higher in the erenumab group vs SoC group and was comparable for convenience domain (13.0 vs 11.9) (Table).

**Conclusion:** This study demonstrated greater improvement in clinical and quality of life outcomes in patients receiving erenumab compared to SoC, who completed 52 weeks on initially assigned treatment. Albeit randomised treatment was well tolerated and considered beneficial until the end of treatment phase, erenumab substantially alleviated illness burden and increased treatment satisfaction compared to SoC, as indicated by improvement in PROs.

**ClinicalTrials.gov Identifier: NCT03927144**

**Table 1 (Abstract LP055):**
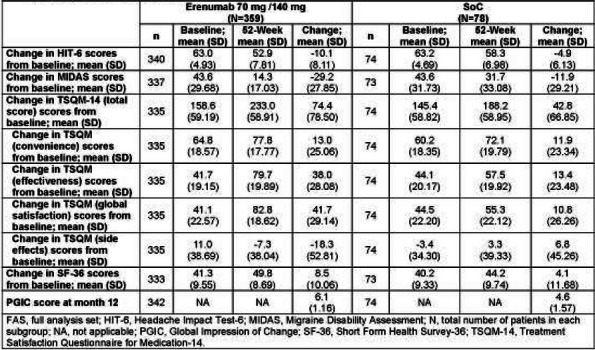
Summary of patient-reported outcomes at Month 12 FOR patients who completed 52 weeks on the initially assigned treatment (FAS population)

## LP056 Postdromal symptoms in migraine: a REFORM study

### J. Thuraiaiyah, H. Ashina, R. H. Christensen, H. Al-Khazali, M. Ashina

#### Copenhagen University Hospital - Rigshospitalet, Department of Neurology, Danish Headache Center, Glostrup, Denmark

##### **Correspondence:** J. Thuraiaiyah

*The Journal of Headache and Pain 2024*, **25(Suppl 1):**LP056

**Objective:** To investigate the proportion of individuals with migraine from a tertiary care unit reporting postdromal symptoms in adherence with the ICHD-3 definition, and to examine how the means of enquiry influences the findings. Finally, we assessed to what extend the postdromal symptoms might impact the disease burden.

**Methods:** In a cross-sectional study, participants diagnosed with migraine were questioned about their postdromal symptoms. Free recall was used first, and subsequently a list of 16 potential postdromal symptoms were used to assess the symptoms. A semi-structured interview was used to obtain clinical characteristic, while electronic questionnaires were used to assess the disease burden, i.e., the Headache Impact Test (HIT-6), Migraine Disability Assessment (MIDAS), and the World Health Organization Disability Assessment 2.0 (WHODAS 2.0).

**Results:** A total of 631 individuals were included. Using the list was associated with a greater proportion of participants reporting postdromal symptoms (≥1 symptom) compared to free recall (504 (79.9%) vs. 421 (66.7%), *P* < 0.001). Similarly, the number of symptoms reported was greater with the list (medians 3 [IQR 1 – 6] versus 1 [IQR 0 – 2]; *P* < 0.001). The number of postdromal symptoms was correlated with HIT-6 (*ρ* = 0.14; *P* < 0.001) and WHODAS scores (*ρ* = 0.15; *P* < 0.001), but no correlation was observed with MIDAS (*ρ* = 0.08; *P* = 0.054).

**Conclusion:** Postdromal symptoms are higher prevalent in individuals with migraine. However, our findings underscore the importance of the means of enquiry and suggests the use of standardized assessments. Additionally, it appears that postdromal symptoms are weakly correlated with both HIT-6 and WHODAS scores, which indicate its potential influence on the disease burden.

## LP057 Improvement of comorbid anxiety and depression in migraine patients with the use of injectable preventive calcitonin gene-related peptide (CGRP) antagonists; Review of clinical evidence

### A. Albilali^1^, A. Omaer^2^, R. Bamogaddam^2^, F. Almutairi^2^, R. Alsaif^2^, O. Almohammadi^3^, A. Alhifany^4^

#### ^1^King Saud University, Neurology Unit, Department of Internal Medicine, Riyadh, Saudi Arabia; ^2^King Saud Medical City, Riyadh, Saudi Arabia; ^3^International Medical Center, Jeddah, Saudi Arabia; ^4^Umm Al-Qura University, Clinical Pharmacy Department, College of Pharmacy, Makkah, Saudi Arabia

##### **Correspondence:** A. Albilali

*The Journal of Headache and Pain 2024*, **25(Suppl 1):**LP059

**Objective:** The objective of this study is to review the available evidence regarding the benefit of using CGRP-targeted mAbs for treating patients with migraine with comorbid anxiety and depression to reveal possible correlations between improved comorbid anxiety and depression and improvements in terms of MMDs.

**Methods:** A literature review was conducted on clinicaltrials.gov, PubMed, Ovid Medline, and EMBASE focusing on phase 3 clinical trials, post-hoc analysis studies, and real-world evidence (RWE) from the past five years. The review primarily utilized patient-reported outcomes (PROs) tools such as PHQ-9, HDRS, BDI-II, GAD-7, and HARS to assess anxiety and depression in relation to CGRP-targeted monoclonal antibodies.

**Results:** Out of 260 studies, 17 met the inclusion criteria. Eptinezumab lacked sufficient evidence regarding its impact on depression and anxiety. While sufficient evidence on its effect on comorbid anxiety was not available, Fremanezumab was shown to significantly improve comorbid depression in one study. Both, Erenumab and galcanezumab showed significant improvement in comorbid depression. Galcanezumab showed faster relief from depressive symptoms compared to the other injectable CGRP antagonists. Galcanezumab also exhibited improvements in GAD-7 scores for anxiety, although not statistically significant, while RWE showed promising HARS scores for both galcanezumab and erenumab.

**Conclusion:** Galcanezumab and erenumab appear to be more effective in improving concurrent depressive and anxiety symptoms in migraine patients compared to fremanezumab. However, it is important to note that these psychometric questionnaires were not the primary outcome measures in the trials and were not specifically designed to investigate the effects of these medications on depression or anxiety. Further research is needed to fully understand the impact of CGRP antagonists on mental health disorders associated with migraines. These findings have implications for enhancing the overall well-being and quality of life for individuals with migraines and comorbid psychiatric conditions.

## LP058 Telemedicine for headache management in Lithuania

### A. Dapkute^1^, S. Andruskevicius^1^, D. Petrosian^2^, K. Ryliskiene^1^

#### ^1^Vilnius University, Centre of Neurology, Vilnius, Lithuania; ^2^Vilnius University, Faculty of Medicine, Vilnius, Lithuania

##### **Correspondence:** A. Dapkute

*The Journal of Headache and Pain 2024,* **25(Suppl 1):**LP058

**Objective:** COVID-19 restrictions exacerbated challenges in accessing specialized headache care, prompting telemedicine as a promising solution. We conducted a national survey in Lithuania to assess the impact of telehealth on headache management and identify implementation barriers and facilitators.

**Methods:** An anonymous e-survey was conducted in January-February 2023 via the Migraine Association of Lithuania. It covered patient sociodemographics, migraine characteristics, prior remote consultation experience, telehealth advantages/disadvantages and future consultation preferences.

**Results:** Among 1046 survey participants, 81% had a confirmed migraine diagnosis. The majority were females (97%) with an average age of 36.7 years. Average monthly headache days (MHDs) were 6.7. 7% had chronic migraine (CM).

35% had prior teleconsultations for headaches, mainly with GPs (26%) over neurologists (17%) (*p*<0.001). Neurologist teleconsultations correlated with a greater distance to a specialist (*p*=0.032), CM (*p*=0.007) and more MHDs (*p*=0.006). Teleconsultation outcomes included treatment extensions (83%), prescriptions for new prophylactic treatment (28%, effective for 74% of patients), and new acute treatments (27%, effective for 77%). Reasons for not seeking teleconsultations (*n*=704) included a lack of awareness that teleconsultations were offered (70%) and specialists not providing such consultations (18%).

67% preferred mixed-type consultations in the future. In-person-only preference (29%) correlated with lower education (*p*<0.001), on-site work (*p*=0.005), lower self-evaluated digital literacy (*p*=0.018), physically demanding work (*p*<0.001), retirement (*p*=0.017), absence of prior treatment extension during teleconsultations (*p*=0.008) and dissatisfaction with remotely prescribed treatment (*p*=0.005).

**Conclusion:** This study reveals higher telehealth utilization by patients with severe migraines in Lithuania. Key barriers include insufficient digital literacy, prior dissatisfaction, and low healthcare provider engagement. A preference for mixed appointments indicates a promising strategy for integrating remote consultations into routine migraine management, facilitating care access without compromising its quality.

## LP059 Clinical characteristics and diagnostic delay in Cluster headache in Egypt

### M. Nada, S. Abualazayem, M. Kamel

#### Cairo University, Neurology, Cairo, Egypt

##### **Correspondence:** M. Nada

*The Journal of Headache and Pain 2024*, **25(Suppl 1):**LP059

**Objective:** We aim to present for the first time in Egypt, the clinical characteristics of cluster headache and assess the diagnostic delay.

**Methods:** A cross sectional study that included all patients presenting with cluster headache diagnosed according to ICHD-III from two large centers from 2 governorates in Egypt over 1 year. Demographic and clinical characteristics were collected. Rate of diagnostic delay in diagnosis was calculated. The protocol was approved by Cairo university ethical committee.

**Results:** Our registry data included 1187 patients with primary headaches who presented in the two headache centers over 1 year. The majority of patients had migraine (81.5%), tension type headache (14.9%), while patients diagnosed as cluster headache represents 1.9% of all patients. The majority of patients were males (82%) male to female ratio 4.75:1. The mean age was 37.9 ± 10 years, while the mean age of disease onset was 25 ± 8 years. Sixteen patients (69.6%) were smoker while 6 patients (26.1%) had comorbidities (hypertension, ischemic heart disease and diabetes). Sixty-five percent of patients had episodic CH, while 34.8% had chronic CH. Most of patients (95.7%) had strictly unilateral pain (right side 52.2%, left side 43.5%), while only one patient had alternating pain. The most common autonomic features were rhinorrhea (91%), ptosis (87%), and lacrimation (78%). Migrainous features as photophobia, phonophobia, nausea and vomiting were found in 26% of patients more in males (4 out of 6). Most of the attacks were nocturnal with infrequent attacks occurring in early morning. The median attack severity by Visual Analog Scale (VAS) was 10 (8-10). The bout duration of CH lasted average 1-4 months duration. The time interval of diagnostic delay ranged from 0.5-29 years, with mean diagnostic delay 9.8 ± 7.9 years. The longer diagnostic delay was associated with poorer response to prophylactic treatment and required polytherapy

**Conclusion:** Our study showed that cluster headache presents 1.9% of primary headache disorders in Egypt. A wide range of diagnostic delay was found that mandate the importance of diagnostic awareness.

**Fig. 1 (Abstract LP059):**
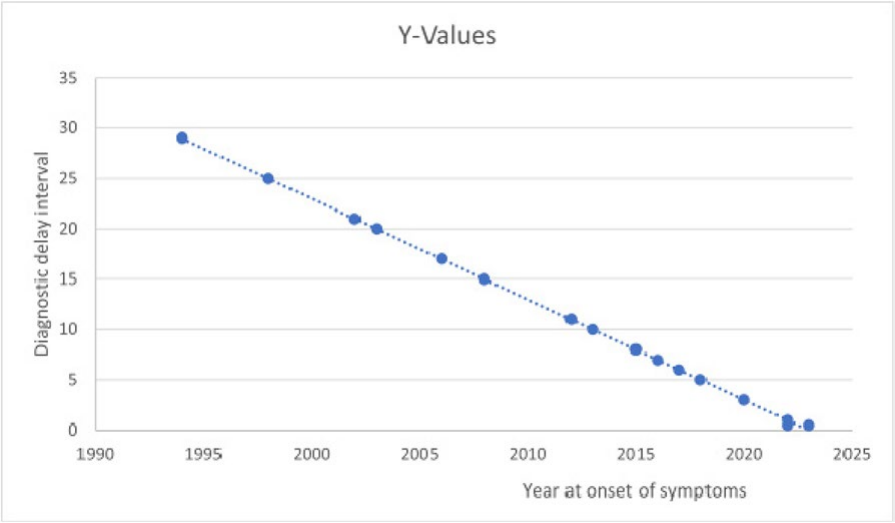
See text for description

**Fig. 2 (Abstract LP059):**
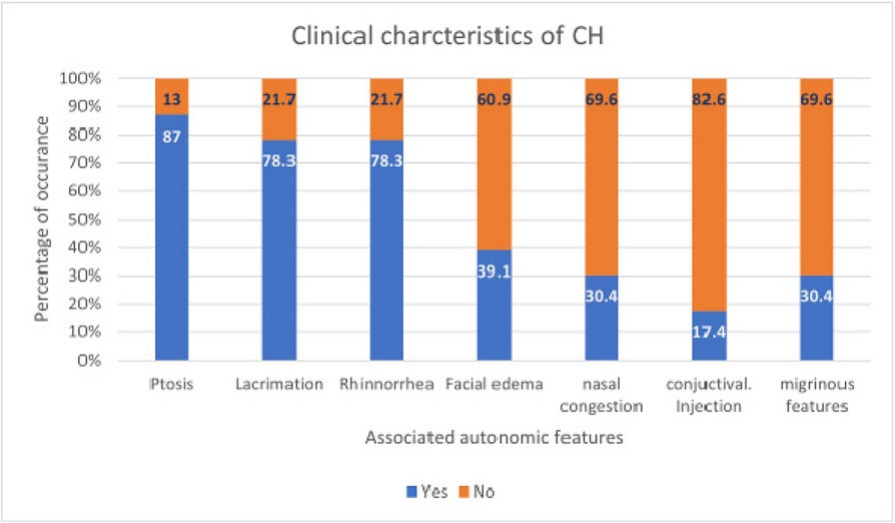
See text for description

## LP060 Episodic status migrainosus: a follow-up of a Mexican series

### A. Marfil Rivera, M. A. Cansino Torres, D. Ortiz Zacarias, A. Martínez Rodríguez

#### Hospital Universitario de Nuevo Leon, Neurology, MONTERREY, Mexico

##### **Correspondence:** A. Marfil Rivera; M. A. Cansino Torres

*The Journal of Headache and Pain 2024*, **25(Suppl 1):**LP060

**Objective:** Status migrainosus is defined as >72 hours of debilitating pain attack. Its prevalence has been estimated between 3-24.5% one third presenting a subsequent relapse. Recently a new variety of migraine has been identified baptized as episodic status migrainosus, usually in migraineurs without aura. Most patients are women with history of chronic migraine and depression. The aim of this study is to communicate the clinical characteristics of series of patients with episodic status migrainosus in a Mexican population.

**Methods:** PREMECEF (First Mexican Headache Registry) is a registry of headaches in Mexican population. We analyzed the clinical characteristics, treatment and evolution of people with migraine attacks fulfilling duration >72 hours.

**Results:** 14 cases were identified. All were female. The main duration of headaches was 72 hours. The current frequency is between 3 to 5 months. Pain quality was pulsatile, mean pain intensity 7/10. The most prevalent accompanying symptom was photophobia. Associated triggers were menstruation, alcohol intake, emotional stress, smoke and sweet flavors. Depression and anxiety were also described. The treatment to terminate the status with intramuscular betamethasone in four patients and triptans in the rest, all of them with a good symptomatic response.

**Conclusion:** The mechanisms that terminate a migraine attack are not known. Whatever they are, in these patients they are ineffective. It could be a special variety of migraine that tends to have a different evolution, as well as a lesser response to abortive treatment. Given the frequency of attacks in our patients, they were successfully managed with abortive treatment so preventive treatment may not be necessary. Unlike the original case series, our patients did not tend to develop chronic migraine. The episodic status migrainosus could constitute a new variety of migraine. Diagnostic criteria have been proposed, but not yet accepted. Abortive treatment should be individualized.

## LP061 Treatment of hypnic headache: a single centre experience

### M. Abu Lafi, A. A. Ashkenazi

#### Sharee Zedek Medical Center, Neurology Department, Jerusalem, Israel

##### **Correspondence:** M. Abu Lafi

*The Journal of Headache and Pain 2024*, **25(Suppl 1):**LP061

**Objective:** Hypnic Headache (HH) is a rare primary headache disorder characterized by headache attacks that occur exclusively during sleep, awakening the patient. HH typically affects individuals older than 50 years. Its pathophysiology is incompletely understood, however, the strong association with sleep suggests hypothalamic involvement. To date, treatment options for HH have been evaluated in case reports and case series only, with various medications showing efficacy. Here we report on our experience with HH treatment.

**Methods:** This is a case series of HH patients seen at the Shaare Zedek Medical Center Headache Clinic. Patients met the ICHD-3 criteria for HH. Data was collected from the patients' electronic medical records and by phone calls. We analyzed headache clinical characteristics and response to treatment in each patient.

**Results:** Six patients were included. The mean age at symptom onset was 63.8 years (SD: 9.93, range: 50—80), with a male-to-female ratio of 2:1. Headache was bilateral in 100% of patients, with a predilection for the vertex (83% of cases). Headache occurred exclusively during nighttime sleep in all patients. None of the patients exhibited autonomic manifestations or migrainous features during attacks, although 33% had previously experienced migraine. Two patients who were initially given verapamil showed an excellent response, with an 87% reduction in headache days (from 30 to 4 days per month). Similarly, two patients initially treated with caffeine had an excellent response, with a 73.4% reduction in headache days (from 30 to 8 days per month). Two other patients who were given melatonin showed mild or no improvement; subsequently, indomethacin was prescribed, still with only mild improvement.

**Conclusion:** In this case series, verapamil and caffeine demonstrated efficacy in the treatment of HH, whereas indomethacin and melatonin yielded less favorable results. Our findings suggest that verapamil, for which there are few reports as a treatment for HH, may be effective for this disease.

## LP062 Headache prevalence and associated factors among delta University students: a cross-sectional study

### A. Abdulatif Mosa^1^, A. Soliman^1,2^, M. Eltantawy^1,2,3^, M. Ismail^1,2,3,4^

#### ^1^Delta University for science and technology, NEUROLOGY, Mansoura, Egypt; ^2^Delta University for science and technology, community, Damitta, Egypt; ^3^Delta University for science and technology, Neurology, Almansourah, Egypt; ^4^Delta University for science and technology, Almansourah, Egypt

##### **Correspondence:** A. Abdulatif Mosa

*The Journal of Headache and Pain 2024*, **25(Suppl 1):**LP062

**Objective:** Headache is a common neurological disorder that affects individuals across various age groups. Among university students, we try to measure the burden of these factors like academic stress, lifestyle changes, and irregular sleep patterns may contribute to the occurrence of headaches.

**Methods:** In this study, a scientific approach was employed to explore the prevalence and impact of headaches among students at Delta University, Egypt. The study utilized a cross-sectional survey methodology, specifically the Headache Impact Test questionnaire, to collect data from a sample of 515 participants. The primary aim was to assess the prevalence of headache impact and to identify the factors that contribute to its occurrence. Additionally, to gain further insight into the potential triggers of headaches, a separate Headache questionnaire cross-sectional survey was conducted.

**Results:** 375 participants (72.8%) experienced a severe impact of headaches, 61 participants (11.8%) had a substantial impact of headaches, 60 participants (11.7%) reported some impact of headaches, and 19 participants (3.7%) experienced little to no impact from headaches.

In the headache questionnaire, the predominant response, as indicated by 92.8% of the participating students, was that their headaches were associated with stress. The mean age of onset for these headaches was found to be 15.75 ± 2.76 years, with a range spanning from 6 to 23 years.

**Conclusion:** Most of participants experienced a severe impact of headaches,11.8% of them had a substantial impact of headaches,11.7% reported some impact of headaches, and 3.7% experienced little to no impact from headaches, the predominant response was that their headaches were associated with stress.

**Fig. 1 (Abstract LP062):**
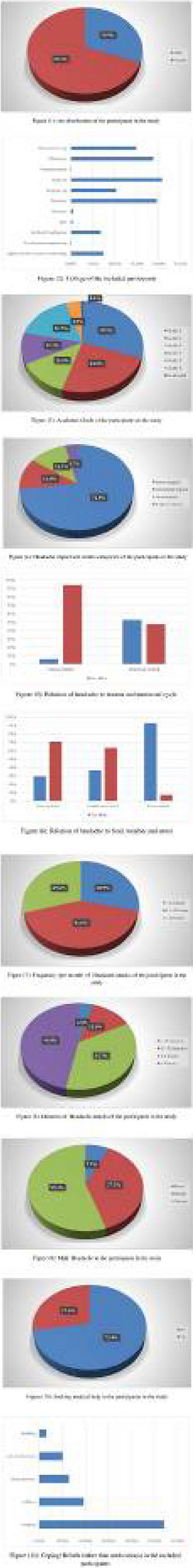
See text for description

**Fig. 2 (Abstract LP062):**
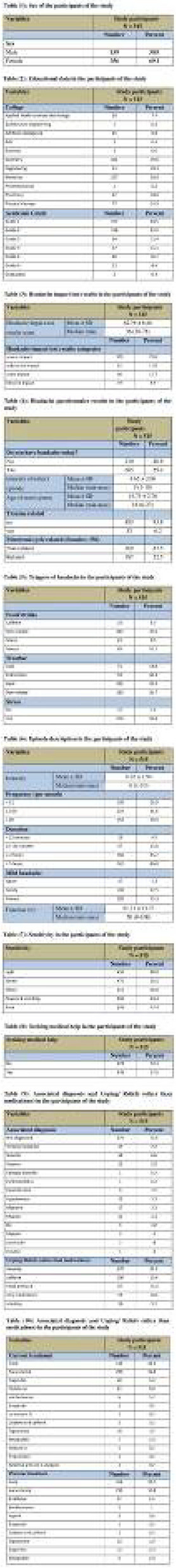
See text for description

**Fig. 3 (Abstract LP062):**
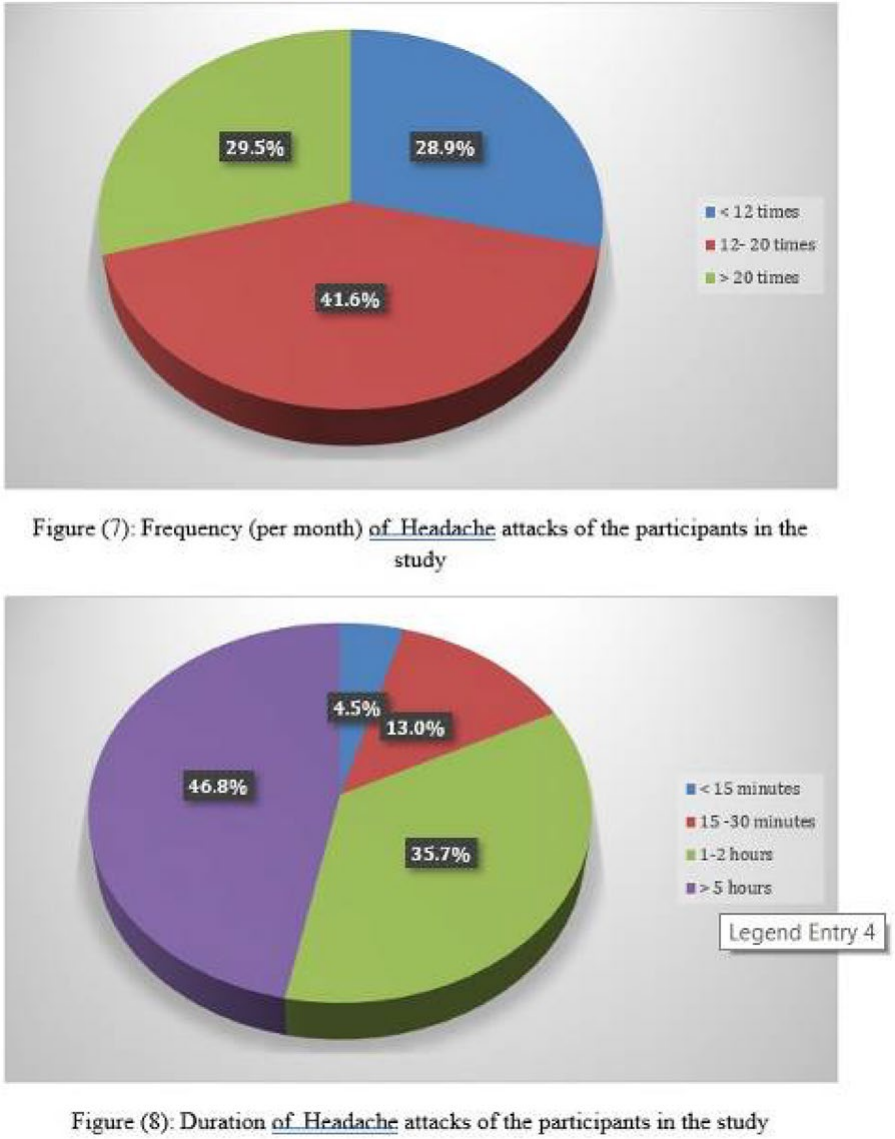
See text for description

## LP063 The comparative safety and efficacy of anti-migraine medications and devices in migraine prophylaxis: a network meta-analysis

### R. B. Nieto^1^, M. J. Láinez^2^, M. Huth^3^, P. Francis^4^, S. Singh^5^, A. McMaster^6^, S. N. Hasham^7^, B. Suthar^5^, P. K. Srivastava^8^

#### ^1^Hospital de La Santa Creu, Department of Neurology, Barcelona, Spain; ^2^Hospital Clínico Universitario de Valencia, Department of Neurology, Valencia, Spain; ^3^Netcare Linksfield Hospital, Neurology, Johannesburg, South Africa; ^4^Private Neurology Practice, Neurology, uMhlanga, South Africa; ^5^Dr. Reddy's Laboratories, Clinical Innovations- Neurology, Hyderabad, India; ^6^Dr. Reddy's Laboratories, Medical Affairs, Johannesburg, South Africa; ^7^Dr. Reddy's Laboratories, Global strategic Portfolio, Hyderabad, India; ^8^Mediception Science Pvt Ltd, Medico-marketing and Clinical Research, Gurgaon, India

##### **Correspondence:** S. Singh

*The Journal of Headache and Pain 2024*, **25(Suppl 1):**LP063

**Objective:** To enable a safety and efficacy comparison of diverse migraine prophylaxis options, including medications and devices, using Network Meta-analysis (NMA), incorporating direct and indirect evidence.

**Methods:** A systematic literature search was conducted in the PubMed, Cochrane & ClinicalTrials.gov databases upto 20 April 2023, to identify randomized placebo-controlled trials on medications & devices for migraine prophylaxis in adults. NMA was conducted using the Bayesian framework in Winbugs software platform & Surface Under Curve Ranking Area (SUCRA) scores were created to rank the interventions.

**Results:** 28 eligible trials involving 13,112 patients were identified. Studies having the Confidence Interval data of their end-points were considered. Atogepant & Eptinezumab demonstrated favorable outcomes in exploratory quality-of-life assessments. On safety, all interventions were comparable to placebo, except Amitriptyline, Topiramate, Fremanezumab, and Galcanezumab. Devices (Nerivio, Genesis, gammaCore) & gepants were the safest interventions. Atogepant & devices like Cefaly & Nerivio had the major proportion of subjects experiencing ≥50% reduction in Monthly Migraine Days (MMDs). Calcitonin gene-related peptide monoclonal antibodies (CGRP-mAbs) and non-pharmacological therapies like Nerivio, displayed high SUCRA scores for difference with placebo in mean MMDs reduction; however, for Nerivio the sample size was smaller. Nerivio exhibited superior SUCRA scores over placebo for reducing the mean monthly headache days among all evaluated interventions. Nerivio and Galcanezumab exhibited higher efficacy in reducing acute medication usage days vs placebo.

**Conclusion:** Our NMA results suggest that neuromodulation devices (Nerivio), CGRP-mAbs, and Atogepant offer promising safety and key efficacy profiles for migraine prophylaxis. Studies with larger sample sizes and head-to-head comparisons are needed.

## LP064 Effect of median nerve stimulation on headache frequency and cortical activity in patients with migraine without aura

### Y. W. Lin^1^, L. L. H. Pan^2^, S. J. Wang^3^, L. W. Chou^1^

#### ^1^National Yang Ming Chiao Tung University, Physical Therapy and Assistive Technology, Taipei City, Taiwan; ^2^National Yang Ming Chiao Tung University, Brain Research Center, Taipei City, Taiwan; ^3^Taipei Veterans General Hospital, Department of Neurology, Neurological Institute, Taipei City, Taiwan

##### **Correspondence:** Y. W. Lin

*The Journal of Headache and Pain 2024*, **25(Suppl 1):**LP064

**Objective:** Investigate the effect of median nerve stimulation (MNS) on headache frequency and cortical activities in migraine without aura (MwoA), and identify potential electroencephalogram (EEG) biomarkers for long-term MNS intervention.

**Methods:** Twenty patients with MwoA received daily 30-minute sensory-level MNS on the left volar forearm for 8 weeks. The participants used an electronic headache diary to record monthly headache days (MHD) as the primary outcomes. Eye-opened EEG was recorded using 32-channel actiCHamp EEG system (Brain Product, Germany) at the beginning, 8th week (Posttest), and 12th week (Follow-up) after the intervention started. EEG before and after a single session MNS was also recorded before the 8-week intervention. The relative power spectral density (rPSD) of EEG was analyzed for the prefrontal, frontal, parietal, temporal, and occipital regions.

**Results:** After the 8-week MNS intervention, MHD significantly decreased at Posttest (-1.8 d/M) and Follow-up (-1.4 d/M). Significantly decreased occipital rPSD in the delta band was observed at Posttest. In addition, increased occipital rPSD within beta, low gamma, and high gamma bands were also noted. Our correlation analysis revealed that MwoA with greater decrement in delta rPSD in the frontal and temporal regions after a single session MNS at baseline showed a greater reduction in the MHD after intervention.

**Conclusion:** Our preliminary findings suggest that eight weeks of MNS can reduce MHD and lead to consistent changes in cortical activity among patients with MwoA. The decrease in delta rPSD at the occipital region may indicate a reduction in migraine severity (Bjørk, M. H. et al., 2009), while the increase in beta rPSD after treatment suggests that brain activity may become more similar to that of healthy individuals (Cao, Z. et al., 2016). Furthermore, changes in rPSD following a single session of MNS may serve as a predictor of the long-term effects of MNS in migraine prevention.

**Fig. 1 (Abstract LP064):**
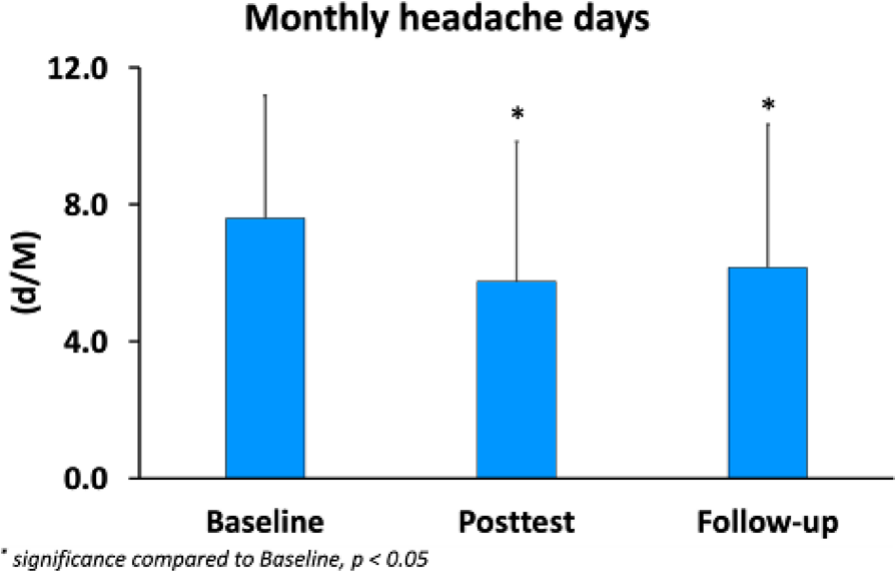
The monthly headache days at three time points

**Fig. 2 (Abstract LP064):**
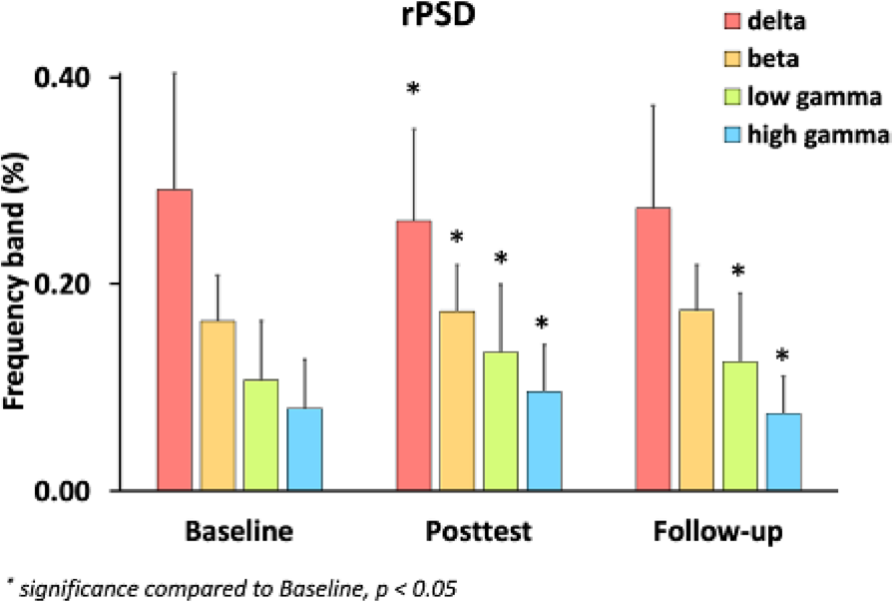
The occipital rPSD within different frequency bands at three time points

**Fig. 3 (Abstract LP064):**
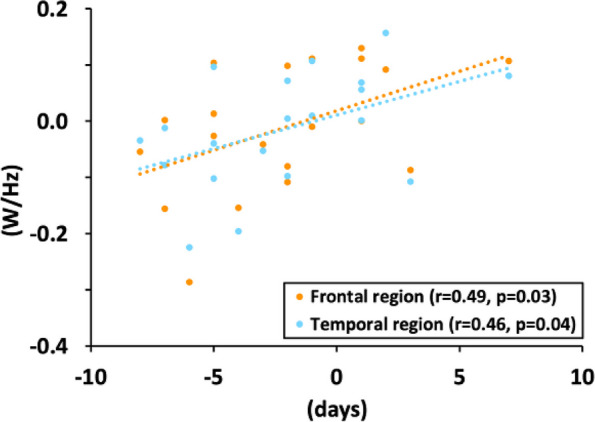
The correlation between delta rPSD changes induced by a single MNS session and MHD reduction following an 8-week intervention

## LP065 Case report: Kinetic oscillation stimulation for the therapy of chronic migraine with limited therapy options in a patient with systemic mastocytosis

### F. Rimmele, P. Kropp, T. P. Jürgens

#### University Medical Centre Rostock, Department of Neurology, Rostock, Germany

##### **Correspondence:** F. Rimmele

*The Journal of Headache and Pain 2024*, **25(Suppl 1):**LP065

**Objective:** Intranasal kinetic oscillatory stimulation (K.O.S.) is a new prophylactic therapy for chronic migraine that has shown good tolerability and efficacy in a recent study. Systemic mastocytosis is a rare disease that occurs in the form of indolent systemic mastocytosis (ISM) most often in younger adults between the ages of 20 and 40. Various stimuli such as allergens, drugs, etc. can lead to uncontrolled mast cell degeneration, which can result in a variety of symptoms, including anaphylactic shock.

**Methods:** A 45-year-old female patient with a history of chronic migraine with visual auras presented to our headache centre. In addition to systemic mastocytosis with evidence of a KIT-D816V mutation, the patient suffers from bronchial asthma, recent depression and anxiety disorder. On average, the patient had migraines on 18 days/month. Due to the mastocytosis and allergic reaction to multiple substances, acute medication was only possible with tramadol, which had only a moderate effect. The established medicinal migraine prophylactics could not be used because of various contraindications; the patient reacted allergically to the amitriptyline considered and the multiple injections of prophylaxis with onabotulinumtoxinA are also contraindicated in mastocytosis.

**Results:** The patient received kinetic oscillation stimulation (K.O.S.) in a double-blind, randomised placebo-controlled study. Over a period of 6 weeks, weekly stimulation of 10 minutes per nostril was performed, during which the migraine improved very well and a reduction to an average of 8 migraine days/month was achieved. The patient reported no side effects during the stimulation therapy. After completion of the study and unblinding, the patient could be assigned to the test (verum) group. The improvement effect lasted for 4 months.

**Conclusion:** In this particular case, established migraine prophylactic medication was not possible in a patient with chronic migraine due to her other illnesses, especially systemic mastocytosis. The K.O.S. carried out within the framework of a study turned out to be a well-tolerated and long-lasting prophylaxis with good efficacy.

*Disclosure statement*: Informed consent to publish this case study and its potentially identifiable information of the patient was obtained from the individual involved. The patient gave explicit permission for the publication of this case report, including any relevant clinical details.

## LP066 Resting-state EEG power analysis in patients with chronic and high-frequency episodic migraine

### J. Fransen^1^, N. Ikumi^1^, A. Marti-Marca^1^, V. J. Gallardo^1^, A. Vilà-Balló^1^, E. Caronna^2^, X. Cerda-Company^2^, M. Torres^2^, A. Alpuente^2^, P. Pozo-Rosich^2,1^

#### ^1^Vall d’Hebron Hospital & Research Institute, Universitat Autonoma de Barcelona, Headache and Neurological Pain Research Group, Barcelona, Spain; ^2^Hospital Universitari Vall d'Hebron, Department of Neurology, Barcelona, Spain

##### **Correspondence:** J. Fransen

*The Journal of Headache and Pain 2024*, **25(Suppl 1):**LP066

**Objective:** This study aimed to compare the frequency band activity of the delta, theta, alpha and beta between headache-free, healthy controls (HC), and patients with high-frequency episodic (HFEM) and chronic migraine (CM), both interictally and ictally. In addition, we assessed the reliability of these frequency bands in HC.

**Methods:** To study this, five minutes of resting state EEG were recorded during two sessions in 66 migraine patients (MP) (15.65 ± 5.1 headache days per month) and 30 HC. The mean time between the first and second session was 10.4 ± 15.43 days. Patients were diagnosed with HFEM or CM according to the International Classification of Headache Disorders (ICHD-3). Only EEG recordings of MP in the interictal and ictal phase were used for the statistical analysis. Clinical and sociodemographic data were collected. The mean relative power of the delta (1-3,9 Hz), theta (4-7,9 Hz), alpha (8-12,9 Hz) and beta (13-30 Hz) frequency in the fronto-central, temporal and occipito-parietal regions were computed. Mixed-effect linear regression models (LMM) were used for comparison between groups. The p-value is adjusted with the fdr method.

**Results:** EEG recordings of 63 MP (mean 15.79±5.1 headache days per month, 54 females, and 40.1±8.9 years old) and 25 gender- and age-matched HC (20 females, 38,6±9.1 years old, p>0.47) were included in the statistical analyses. An intraclass correlation (ICC) analysis in HC confirmed the consistency of power for the delta (ICC>0.79), theta (ICC>0.88), alpha (ICC>0.89) and beta band (ICC>0.82) between the two EEG sessions. The results of the LMM showed no significant main effect of group (interictal, ictal and HC) on the power of delta (*p*>0.61), theta (*p*>0.61), alpha (*p*>0.13) and beta (*p*>0.16) band.

**Conclusion:** Our results demonstrate that the power activity of all four frequency bands are reliable individual measurements of brain activity but do not differ between migraine patients (both interictally and ictally) and healthy controls.

